# Searching for genes determining the APR phenotype in rye

**DOI:** 10.1186/s12870-025-06920-0

**Published:** 2025-07-19

**Authors:** Joanna Szewińska, Mateusz Matuszkiewicz, Monika Rakoczy-Trojanowska, Magdalena Święcicka, Marianna Krysińska, Wojciech Wakuliński

**Affiliations:** 1 Institute of Biology, Warsaw University of Life Sciences, Nowoursynowska 159, Warsaw, 02-776 Poland; 2 Institute of Horticultural Sciences, Warsaw University of Life Sciences, Nowoursynowska 159, Warsaw, 02-776 Poland

**Keywords:** *Secale cereale*, Leaf rust, Adult plant resistance, Phylogenetic analysis, *Puccinia recondita* f. sp. *secalis*, ABC transporters, Sugar transporters

## Abstract

**Background:**

Adult-plant resistance (APR) is a type of genetic resistance of cereals against a range of disease-causing pathogens including leaf rust (LR). In rye, APR to LR although known, is poorly understood, especially at the molecular level. Recently, numerous variants of genes encoding ATP-binding cassette (ABC) and sugar transporters, have been identified in the rye transcriptome. In these two pools of genes, we decided to find genes determining APR using both nucleotide and amino acid sequence similarity to the *Lr34* and *Lr67* genes carrying the APR to LR in wheat as the main selection criterion and as an additional criterion - expression profiles of chosen variants in seedlings infected with LR.

**Results:**

The phylogenetic analysis of chosen genes *ScLr_ABC* and *ScLr_SUG* encoding, respectively, ABC and sugar transporters revealed that a lack of polymorphisms responsible for APR in wheat. However, *ScLr_SUG1*, a putative ortholog of *Lr67*, and *ScLr_ABC25*, which shows high 3D structural similarity to Lr34, could potentially be involved in APR of rye.

The analysis of the expression of selected *ScLr_ABC* and *ScLr_SUG* genes carried out on plants infected with fungal spores collected from locations where phenotypic assessments were performed. Most of the analyzed genes did not show any clear association between APR to LR. Only S*cLr_ABC25* gene seems to determine APR-type immunity against LR.

**Conclusions:**

This work is the first attempt to find genetic determinants of APR resistance to LR in common rye. Our studies show that the mechanism of this type of resistance is different in rye than in other cereals studied in this respect (mainly wheat and barley). However, our findings are a good starting point for further research, and, as in the case of the S*cLr_ABC25* gene - they can be the basis for creating a molecular resistance breeding program focused on selecting forms characterized by APR to LR.

**Supplementary Information:**

The online version contains supplementary material available at 10.1186/s12870-025-06920-0.

## Background

Adult-plant resistance (APR) is a type of genetic resistance of cereals against a range of disease-causing pathogens acting, by definition, at the mature plant stage. APR can be both multi-pathogen resistance and, in very few cases - pathogen-specific [1, 2]. Moreover, it provides long-term durable resistance by slowing the rate of pathogen sporulation [3]. Although APR is best known in wheat, this type of resistance has been described in other cereal species such as barley, rice, maize, sorghum and rye [4, 5]. APR mainly concerns rust fungi, comprising leaf rust, LR [2], but this type of resistance has also been found against other pathogens, including hemibiotrophs, e.g. *Magnaporthe oryzae* in rice [6] or *Colletotrichum sublineolum* in sorghum [7].

In wheat, the species best known in terms of APR and at the same time the closest relative of rye, nearly 30 genes determining APR (and, usually seedling stage resistance, SR) [8, 9] have been identified in different plant resources. However, only two genes – *Leaf rust resistance (Lr)34* and *Lr67* have been deeply characterized on molecular level so far. Both genes confer APR to many diseases, in a non-race-specific manner and code for cellular transporters [10]. Moreover, according to some authors, both *Lr34* and *Lr67* genes also determine seedling resistance to LR in wheat [11–13] and barley [14].

The gene *Lr34*, located on 7DS chromosome, codes for a membrane protein - an ATP-binding cassette (ABC) transporter - for which ABA is the substrate [15–17]. The second gene, *Lr67*, located on chromosome 4D (with homeologous on 4 A and 4B), encodes a hexose transporter associated with anion fluxes [18–22].

Both genes are complex loci that confer resistance to stripe rust, stem rust, powdery mildew, and barley yellow dwarf virus. Due to their pleiotropic function, they are referred to as *Lr34/Sr57/Yr18/Pm38* and *Lr67/Sr55/Yr46/Pm46* [1, 19, 23–25].

Discreet structural changes in both genes determine the APR phenotype. For *Lr34*, a 3-nucleotide indel causes phenylalanine deletion at position 546 and an SNP leads to the substitution of conserved tyrosine to histidine at position 634. These changes are present in resistant (*Lr34res)* allelic variants, found only in cultivated bread wheat so far [6, 15, 16, 26]. Importantly, both amino acid changes are in the first transmembrane domain of the ABC transporter [6]. In the later work, Krattinger et al. [17] suggested that LR34res-mediated ABA redistribution has a major effect on the transcriptional response and physiology in plants carrying *Lr34res*-allele which results in the induction of a constitutive defense response to pathogen attack.

Additionally, as it was showed by Rinaldo et al. [11], the over-expression of the gene *Lr34* and increasing day length cause seedling resistance.

In case of *Lr67* gene, the resistant (*Lr67res*) haplotype is related to the presence of two amino acids, arginine in position 144 and leucine in position 387 instead of glycine and valine in susceptible haplotypes. This change translates into a reduction in glucose uptake, which in turn results in limited growth of multiple biotrophic pathogens [19].

The wheat *Lr34* gene was introduced via genetic transformation to other cereal species, such as barley [27–29], rice [6], maize [30] and sorghum [7]. In all cases, transgenic plants were resistant to rust fungi and, like wheat, to powdery mildew. However, some differences and specificity of the function of the *Lr34* transgene compared to wheat were found. Transgenic barley with *Lr34* gene showed resistance at only seedling stage. In rice, maize and sorghum, *Lr34* transgene conferred resistance not only against biotrophic fungi but also against hemibiotrophs [6].

Similarly, as for *Lr34*, the introduction of the wheat *Lr67* gene into barley resulted in gaining APR against leaf rust and powdery mildew [14]. In addition to LR APR-related genes and QTLs, three miRNAs - tae-miR9653b, tae-miR9773 and tae-miR9677 have been recently identified in common wheat by Spychała et al. [31]. The authors showed potential downregulatory link between one of these genes - tae-miR9653b and *Lr34* gene expression hypothesizing that tae-miR9653b may control the degradation of *Lr34* gene transcripts.

In rye, LR caused by *Puccinia recondita* Roberge ex Desm. f. sp. *secalis* is one of the most damaging diseases [32–34]. The genetic basis of resistance to LR has been relatively well known: (1) including mapping 16 dominant *Pr* genes: *Pr1–5*,* Pr-d–f*,* Pr-i–l*,* Pr-n*,* Pr-p*,* Pr-r*,* Pr-t* [33–35], (2) identification of hundreds of differentially expressed genes (of which the largest groups were genes encoding cytochrome P450, receptor-like kinases, methylesterases, pathogenesis-related protein-1, xyloglucan endotransglucosylases/hydrolases, 1-deoxy-D-xylulose 5-phosphate synthases, β−1,3-glucanases, glycosyltransferases and peroxidases), (3) dozens of differentially accumulated metabolites (including metabolites related to phenylpropanoid and diterpenoid biosynthesis and thiamine metabolism) in response to the infection with LR [36, 37], and (4) detection of two QTLs on chromosome 1R composed of DArTseq and silicoDArT markers, many of which were located in genes encoding proteins with a known function in response to LR, such as NBS-LRR disease resistance protein-like protein and carboxyl-terminal peptidase [38].

Knowledge about APR resistance to LR in rye is still fragmentary compared to the relatively extensive knowledge about seedling-stage resistance, in particular – in terms of molecular determinants. To the best of our knowledge, so far only several QTLs and markers (including highly significant markers at 175.2 cM on chromosome 2R) associated with the true APR against stem rust (but not LR) have been identified [5]. At the same time, the authors found that the most significant markers for adult-plant and seedling resistance were different. None of them co-localized with the genes encoding ABC transporters and/or hexose transporters.

Recently, Krępski et al. [37] identified numerous variants of genes encoding ABC and sugar transporters in the rye transcriptome; some of them were differentially expressed after infection with *Prs*, mainly in compatible interaction, but only at the seedling stage. In these two pools of genes (hereafter referred to as *ScLr_ABC* and *ScLr_SUG*) we decided to find genes determining APR using both nucleotide and amino acid sequence similarity to the *Lr34* and *Lr67* genes as the main selection criterion and as an additional criterion - expression profiles in seedlings infected with *Prs*. We assumed that the most appropriate approach to find the “res” alleles would be mining the gene sequences most closely related to Lr67 and Lr34 in different rye collections. The results of these studies are the subject of the work presented here.

## Methods

### Plant material

In the study, 402 and 192 rye inbred lines belonging to the breeding materials of two Polish Breeding companies: DANKO Plant Breeding (Choryń, Poland; 52°03’49.2"N, 16°78’05.8"E) and Poznań Plant Breeding, PHR (Wiatrowo, Poland; 52°75’34.4"N, 17°14’94.4"E), respectively, were phenotyped in terms of expression of the APR trait. These materials represented a broad phenotypic diversity with respect to resistance against LR. Hereinafter the abbreviated names of these companies will be used: DANKO and PHR.

### Field phenotyping of APR to leaf rust

Phenotyping of rye inbred lines for LR resistance was carried out in 2020 (268 lines in DANKO and 42 lines in PHR) and 2021 (134 lines from DANKO and 150 lines from PHR). The experiments were arranged in a randomized complete block design with two replications. Each microplot consisted of one row with ten plants spaced 20 cm apart. The source of inoculum was the natural field population of *Prs*, which developed on rye plants near the plots containing the tested genotypes. During the cropping seasons of 2020 and 2021, disease severity (DS) was evaluated twice on the leaves of 10 randomly selected rye tillers. The same tillers were assessed during both the first and second disease evaluations, and the results were expressed as mean values for each leaf separately. The infection level was rated using a modified Cobb’s scale, focusing on the percentage of leaf surface infection area: − 1 point: up to 5%, − 2 points: 5–10%, − 3 points: 10–20%, − 4 points: 20–40%, − 5 points: 40–60%, − 6 points: 60–100%. The first observation was made as leaf rust outbreaks began to accelerate, and most of the tested genotypes were in the beginning of the flowering phase (GS 60/69 according to the Zadoks growth stage). The second evaluation was conducted within a 10-day interval. Adult plant resistance (APR) to leaf rust was estimated by measuring the increase of disease severity (IDS) on individual leaves (F, F-1, F-2) and the whole plants, according to the following formula: [(DSF-i)t2- (DSF-i)t1]. During field phenotyping, leaf tip necrosis (LTN) was observed, but this trait was not included in the APR genotype assessment. Agroclimatology data from fields where phenotyping was conducted such as: temperature at 2 m above ground level, temperature at 2 m above ground level range, relative humidity at 2 m above ground level, wind speed at 2 m above ground level, precipitation corrected were obtained from the POWER Project’s Hourly 2.4.2 version on 2024.11.13 (Figure S1).

### Identification of wheat orthologs Lr34 and Lr67 in rye

The nucleotide and amino acid sequences of Lr34 (TraesCS7D03G0183600) and Lr67 (TraesCS4D03G0585200) from wheat (Chinese Spring genome v2.1) were used as queries to identify orthologs in the rye Lo7 genome. BLASTP searches were conducted on the WheatOmics platform [39] to find the best matches for further analysis. To identify putative Lr34 orthologs, we used less stringent parametres: e-value ≤ 1 × 10^−6^, sequence identity ≥ 40% for amino acid sequences, and query cover ≥ 60%. This decision was necessitated by the inability to detect potential Lr34 orthologs under highly restrictive search criteria. For Lr67, we applied more stringent parameters: e-value ≤ 1 × 10^−6^, sequence identity ≥ 50% for amino acid sequences, and query cover ≥ 75%. In addition, conserved domains of each candidate gene were identified using the NCBI Conserved Domains Database [40]. Based on these analyses, rye amino acid sequences homologous to wheat Lr34 and Lr67 were identified as ABC transporters from the ABC G superfamily named ScLr_ABC and sugar transporter protein named ScLr_SUG, respectively.

### Multiple sequence alignment and phylogenetic tree construction

Putative rye orthologs of wheat Lr34 and Lr67 were used to identify the most similar proteins from *Triticum aestivum* (v2.1), *Aegilops tauschii* (Aet v4.0), *Oryza sativa* (IRGSP 1.0), *Brachypodium distachyon* (IBI v3.0), *Hordeum vulgare* (Morex v3), and *Sorghum bicolor* (NCBI v3) using the Triticeae-GeneTribe homology database (http://wheat.cau.edu.cn/TGT; [41]). Amino acid sequences were extracted from the Ensembl Plants Database and NCBI database. Multiple sequence alignments were performed using Clustal Omega [42]. Phylogenetic trees were constructed using IQ-TREE [43] with the Maximum likelihood method. The bootstrap values were calculated from 1000 replicates [44]. Phylogenetic trees were visualized using iTOL (version 6.0). List of gene identificators of found orthologues used in phylogenetic analysis is in supplement (Table S1).

### Comparison of predicted protein structural models

Protein structure models for the reference sequences Lr34res (acn41354_1) and Lr34sus (acl36478) and the test sequences ScLr_ABC1 and ScLr_ABC25 downloaded from the WheatOmics platform [39] were made with the AlphaFold3 tool with default parameters [45]. Visualization of the protein models performed in PyMOL (PyMOL Molecular Graphics System, Version 2.0 Schrödinger, LLC).

### Amplicon sequencing and contig assembly

To investigate the nucleotide sequence of the *ScLr_SUG1* gene in different inbred lines with APR and non-APR phenotypes, we designed seven pairs of PCR primers that cover the entire gene sequence. Amplicon sequencing was performed by Genomed S.A. (Warsaw, Poland). To obtain the complete sequence, contig assembly was performed using CAP3 software [46]. To elucidate gene structure of *ScLr_SUG1* in different inbred lines we used FGENESH with two organism specific gene-finding parameters - wheat and monocot plants [47]. A similar approach was used to investigate the polymorphic regions associated with the APR phenotype in ScLr_SUG4 and ScLr_SUG6. However, sequencing was performed exclusively on the coding regions of the gene and was limited to eight selected inbred lines. Primers were designed to flank potential nonsynonymous single-nucleotide polymorphisms (SNPs) in exons 2 and 3, which are implicated in the APR phenotype in wheat. The list of primers utilized in this study is provided in the Figure S2.

### Analysis of expression of *ScLr_ABC* and *ScLr_SUG* genes

To perform expression analysis of chosen *ScLr*_*ABC* and *ScLr_SUG* genes, four lines were selected. For each company we chose two APR and two non-APR lines. The lines from DANKO were numbered 118 and 120 (APR lines), and 59 and 61 (non-APR lines). In the case of PHR, the selected APR lines were numbered 71 and 149, and the non-APR lines – 52 and 105.

Seeds of these lines were surfaced sterilized by rinsing in commercial bleach (1 part to 8 parts of water) for 15 min and then in distilled water, and then they germinated in Petri dishes in dark at 22 °C. After two days all seedlings were placed into plastic pots (diameter 12 cm) containing previously sterilized peat substrate and incubated in a growth chamber for 26 days at 22 °C and the light intensity of 60 µmol m^−2^ s^−1^, in 16-h light/8-h dark photoperiod. The experiment was performed in three biological replicates, each consisting of three individual plants.

### Infection of APR and non-APR rye plants with *Prs*

Four-week-old seedlings with 3–4 leaves (Zadoks scale: 13–14) were inoculated with *Prs* spores. This developmental stage was chosen based on methodology described previously by Rinaldo et al. [11] and Milne et al. [14] who investigated *Lr34* gene and *Lr67* gene in barley, respectively.

Prior to inoculation, spores of *Prs* collected in DANKO (then used for infection of plants of lines 118, 120, 59 and 61) and PHR (then used for infection of plants of lines 71, 149, 52 and 105) were suspended in Novec-7100 engineered fluid (at a density of 1 mg cm^−3^) in brown glass diffusers (Roth, Basel, Switzerland). Control plants were sprayed with Novec-7100 engineered fluid alone. Immediately after the infection, plants were placed in black boxes and incubated at 18 °C, with 100% humidity, for 24 h. Then plants were transferred into growing chambers and incubated under the same conditions as before the inoculation. Plant tissue was collected 5 days after infection (dai), frozen immediately in liquid nitrogen, and stored at − 80 °C. The tissue samples were collected at this time point based on the preliminary data of *ScLr_SUG1* and *ScLr_SUG6* expression profiles in two inbreed line L318 and D39 (Figure S3).

### Macroscopic observations

To evaluate the *Prs* infection type, 10 dai a macroscopic observation were completed using the following 6 point scale [48]: 0 = immune (no visible reaction), 0; = resistant (chlorotic or necrotic flecking), 1 = resistant (minore uredinia surrounded by chlorosis or necrosis), 2 = moderately resistant (small pustules surrounded by chlorosis), 3 = moderately susceptible (moderately large pustules surrounded by chlorosis), and 4 = susceptible (moderately large to large pustules with little or no chlorosis).

### RNA extraction

Total RNA was isolated from liquid N2-frozen rye leaves using the Universal RNA Purification kit (EURx, Gdańsk, Poland) following the manufacturer’s instruction. To remove genomic DNA contamination, during isolation RNA was treated with RNase-free DNase I (EURx, Gdańsk, Poland). RNA was diluted in 40 µl of RNase-free water. The RNA quantity was measured spectrophotometrically (NanoDrop ND-1000; Thermo Scientific, Wilmington, DE, USA) and its integrity and purity were tested on a 1.2% (w/v) agarose gel.

### cDNA synthesis and qRT-PCR analysis

The relative expression levels of *ScLr_ABC1*,* ScLr_ABC25*, *ScLr_SUG1* and *ScLr_SUG6* were determined by an qRT-PCR analysis. These four gene variants were selected based on bioinformatic analysis.

Purified total RNA (1000 ng) was reverse transcribed using RevertAid First Strand cDNA Synthesis Kit (Thermo Fisher Scientific, Waltham, MA, USA), according to the manufacturer’s instruction.

Quantitative real-time PCR (qRT-PCR) was carried out by using a LightCycler 96 Real Time System (Roche, Basel, Switzerland). Reactions were completed using a 96-well plate. Each reaction volume of 20 µl contained 10 µl FastStart Essential DNA Green Master (Roche), 5 µl cDNA (4 ng/µl), 1 µl 10 µM forward primer, 1 µl 10 µM reverse primer, and 3 µl ddH2O. The PCR cycle used was as follows: 95 °C for 600 s, 40 cycles of 95 °C for 10 s, 60 °C for 10 s, and 72 °C for 10 s. qPCR reactions were performed with three biological replicates and two technical replicates. Specific primers of all used genes were listed in Table S2. Gene expression levels were normalized against the expression levels of the two reference genes Actin and ADP-ribosylation factor according to the 2^−ΔΔCt^ method [49]. The statistical analysis of differentially expressed genes was performed using the REST software [50], with *p* ≤ 0.05 set as the threshold for significance.

## Results

### Phenotyping of APR to leaf rust under field conditions

**Fig. 1:**
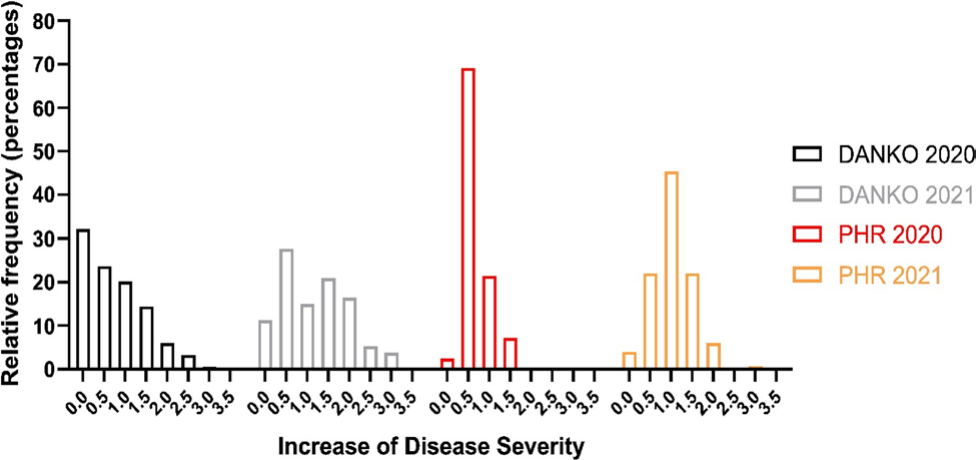
Histogram of Increase of Disease Severity measured on infected leaves (F, F-1, F-2) of rye inbred lines from DANKO and PHR

Phenotypic assessments of rust intensity were conducted twice during each cropping season in 2020 and 2021, allowing for the monitoring of disease progression dynamics. Over these two years, a total of 594 rye genotypes (402 from DANKO and 192 from PHR) were evaluated for slow rusting resistance. Infected plants exhibited a full spectrum of disease symptoms, including uredia covering the phyllosphere and chlorotic or necrotic discoloration of leaf tissue. The percentage of leaf area covered by rust pustules served as the basis for calculating the disease severity and the increase of this index (Fig. 1). The IDS ranged from 0 to 4.5 points on the Cobb’s scale (Table S3, Figure S4). Analysis of infection progression across leaves of the stems (F, F-1, F-2) revealed that very low [0–1] IDS values predominated among the tested genotypes, regardless of location and growing season (Table S3; Figure S4). In contrast, when considering IDS values of the whole plant leaves, complete inhibition of LR development (null IDS), was rare, observed, except for DANKO in 2020, in less than 10% of the lines, specifically 9%, 2,4% and 3,3% in DANKO, 2021, PHR, 2020 and PHR, 2021, respectively. The share of lines characterized by null IDS in DANKO, 2020 was significantly higher and amounted to 23.6% (Fig. 1).

### Identification and characterization of ScLr_ABCs proteins in the Lo7 genome

To find potential rye orthologues of wheat Lr34 we conducted a BLASTP analysis using the amino acid sequence of Lr34 as a query. We identified 29 sequences in the Lo7 genome, called ScLr_ABCs, that show relatively high similarity (42-57%) to Lr34, but not so high as to be considered orthologs of this protein. Results were ordered in descending order of the sequence identity parameter. The highest prefix of the ScLr_ABC gene is the closest homology to the wheat Lr34 protein (Table 1).

**Table 1:** Identification of wheat Lr34 gene putative orthologs in rye Lo7 genome. The amino acid sequence of wheat Lr34 was the query to find putative rye orthologues (ScLr_ABCs) by BLASTP analysis. Results were ordered in descending order of the sequence identity parameter. The highest prefix of the *ScLr_ABC* gene is the closest homology to the wheat Lr34 protein

Name	Accession	Max score	Total score	Query cover	E value	Ident	Description
ScLr_ABC1	SECCE4Rv1G0261990.1	1580.5	1580.5	95%	0	57%	ABC transporter G family member 41
ScLr_ABC2	SECCE5Rv1G0320680.1	1617.0	1617.0	99%	0	56%	ABC transporter G family member 41
ScLr_ABC3	SECCE5Rv1G0320700.1	1583.9	1583.9	97%	0	56%	ABC transporter G family member 41
ScLr_ABC4	SECCE7Rv1G0516320.1	1579.3	1579.3	101%	0	54%	ABC transporter G family member 41
ScLr_ABC5	SECCE3Rv1G0188760.1	1359.4	1359.4	99%	0	49%	ABC transporter G family member 38
ScLr_ABC6	SECCE3Rv1G0180110.1	1360.1	1360.1	101%	0	48%	ABC transporter G family member 37
ScLr_ABC7	SECCE3Rv1G0187220.1	1343.6	1343.6	101%	0	48%	ABC transporter G family member 32
ScLr_ABC8	SECCE3Rv1G0180080.1	1332.0	1332.0	99%	0	48%	ABC transporter G family member 36
ScLr_ABC9	SECCE7Rv1G0503690.1	1303.5	1303.5	98%	0	48%	ABC transporter G family member 44
ScLr_ABC10	SECCE7Rv1G0500880.1	1299.3	1299.3	100%	0	48%	ABC transporter G family member 42
ScLr_ABC11	SECCE7Rv1G0471570.1	1272.7	1272.7	96%	0	48%	ABC transporter G family member 48
ScLr_ABC12	SECCEUnv1G0527620.1	1264.6	1264.6	96%	0	48%	ABC transporter G family member 48
ScLr_ABC13	SECCE5Rv1G0360170.1	1258.0	1258.0	96%	0	48%	ABC transporter G family member 48
ScLr_ABC14	SECCE2Rv1G0126220.1	1218.8	1364.0	92%	0	48%	ABC transporter G family member 37
ScLr_ABC15	SECCE7Rv1G0500890.1	1341.6	1341.6	102%	0	47%	ABC transporter G family member 42
ScLr_ABC16	SECCE7Rv1G0503680.1	1312.4	1312.4	102%	0	47%	ABC transporter G family member 44
ScLr_ABC17	SECCE6Rv1G0389550.1	1297.3	1297.3	102%	0	47%	ABC transporter G family member 39
ScLr_ABC18	SECCE5Rv1G0360180.1	1265.0	1265.0	96%	0	47%	ABC transporter G family member 48
ScLr_ABC19	SECCE6Rv1G0429220.1	1260.7	1260.7	99%	0	47%	ABC transporter G family member 53
ScLr_ABC20	SECCE3Rv1G0163690.1	1232.2	1232.2	98%	0	47%	ABC transporter G family member 31
ScLr_ABC21	SECCE5Rv1G0324050.1	1295.0	1295.0	103%	0	46%	ABC transporter G family member 53
ScLr_ABC22	SECCE4Rv1G0252410.1	1243.8	1243.8	102%	0	46%	ABC transporter G family member 48
ScLr_ABC23	SECCE7Rv1G0483740.1	1147.5	1147.5	96%	0	44%	ABC transporter G family member 45
ScLr_ABC24	SECCE5Rv1G0368180.1	1146.7	1146.7	95%	0	44%	ABC transporter G family member 45
ScLr_ABC25	SECCE5Rv1G0377090.1	1145.6	1145.6	95%	0	44%	ABC transporter G family member 45
ScLr_ABC26	SECCE5Rv1G0368300.1	716.8	854.7	94%	0	44%	ABC transporter G family member 45
ScLr_ABC27	SECCE5Rv1G0368310.1	399.8	489.2	62%	3.65e-126	44%	ABC transporter G family member 45
ScLr_ABC28	SECCE4Rv1G0271130.1	1172.9	1172.9	101%	0	43%	ABC transporter G family member 51
ScLr_ABC29	SECCE4Rv1G0264660.1	1047.7	1047.7	95%	0	42%	ABC transporter G family member 45

All these sequences are members of fourteen clades of ABC transporters, called subfamily ABCG (Table 1). Our bioinformatic analyses showed that the identified transporters have different sequence lengths ranging from 459 (ScLr_ABC27) to 1511 (ScLr_ABC15) amino acid residues, molecular weights ranging between 52.72 and 164.70 kDa, and theoretical isoelectric points (pI) ranging from 6.05 (ScLr_ABC27) to 9.0 (ScLr_ABC26) (Table S4).

**Fig. 2:**
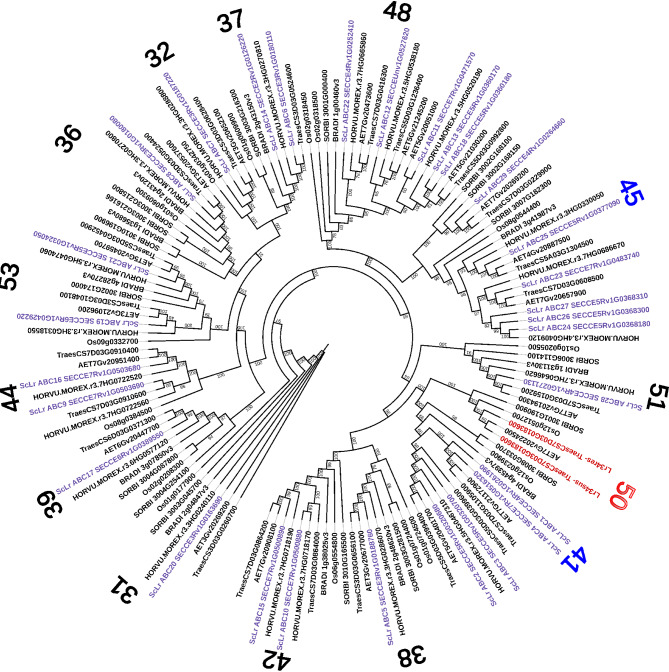
The phylogenetic tree of ABCG transporters. Rye transporters were classified into 14 clades. Wheat Lr34 proteins are depicted in red, rye proteins are depicted in purple, while barley, aegilops, sorghum and rice proteins are depicted in black. The numbers around the phylogenetic tree marked in red, blue, and black represent clades of the ABCG transporter subfamily. The protein tree was constructed using the Maximum likelihood method. The bootstrap consensus tree was generated using 1000 replicates

We selected and further analyzed two out of 29 rye ABCG transporters: ScLr_ABC1 and ScLr_ABC25. We chose ScLr_ABC1 due to its highest similarity (57%) to wheat Lr34, while the reason for selecting ScLr_ABC25 was the presence of a Cys residue at position 634. All the studied rye ABCG transporters, except for ScLr_ABC25, have an aromatic amino acid, Phe or Tyr, at this position (Fig. 2). According to Cloutier et al. [51], the amino acid at position 634 may be crucial for substrate recognition and binding.

### Identification of rye ABCG transporters and analysis of their phylogenetic relationship

To better understand the functions of different ABCG transporters found in the Lo7 genome a phylogenetic analysis was done. All 29 ABCG transporters identified in rye were used to find their orthologs in 6 cereal species such as: *Triticum aestivum*, *Hordeum vulgare*, *Brachypodium distachyon*, *Aegilops tauchii*, *Oryza sativa* and *Sorghum bicolor*.

Our analysis showed that rye ABCG transporters were classified into 14 clades: 31, 32, 36, 37, 38, 39, 41, 42, 44, 45, 48, 50, 51 and 53. We observed that both variants, of wheat Lr34, resistant and susceptible were present in clade 50 while no rye ABCG transporter was found in this clade. Six rye sequences were present in clade 45; five sequences were in clade 48; four transporters belonged to clade 41, two sequences were in clades 37, 42, 44, and 53, and single sequences were in the remaining clades: 31, 32, 36, 38, 39 and 51. Our selected rye ABCG transporters: ScLr_ABC1 and ScL_ABC25 belonged to clades 41 and 45, respectively.

### Comparison of rye and wheat ABCG transporters belonging to clades 41, 45, and 50

**Fig. 3:**
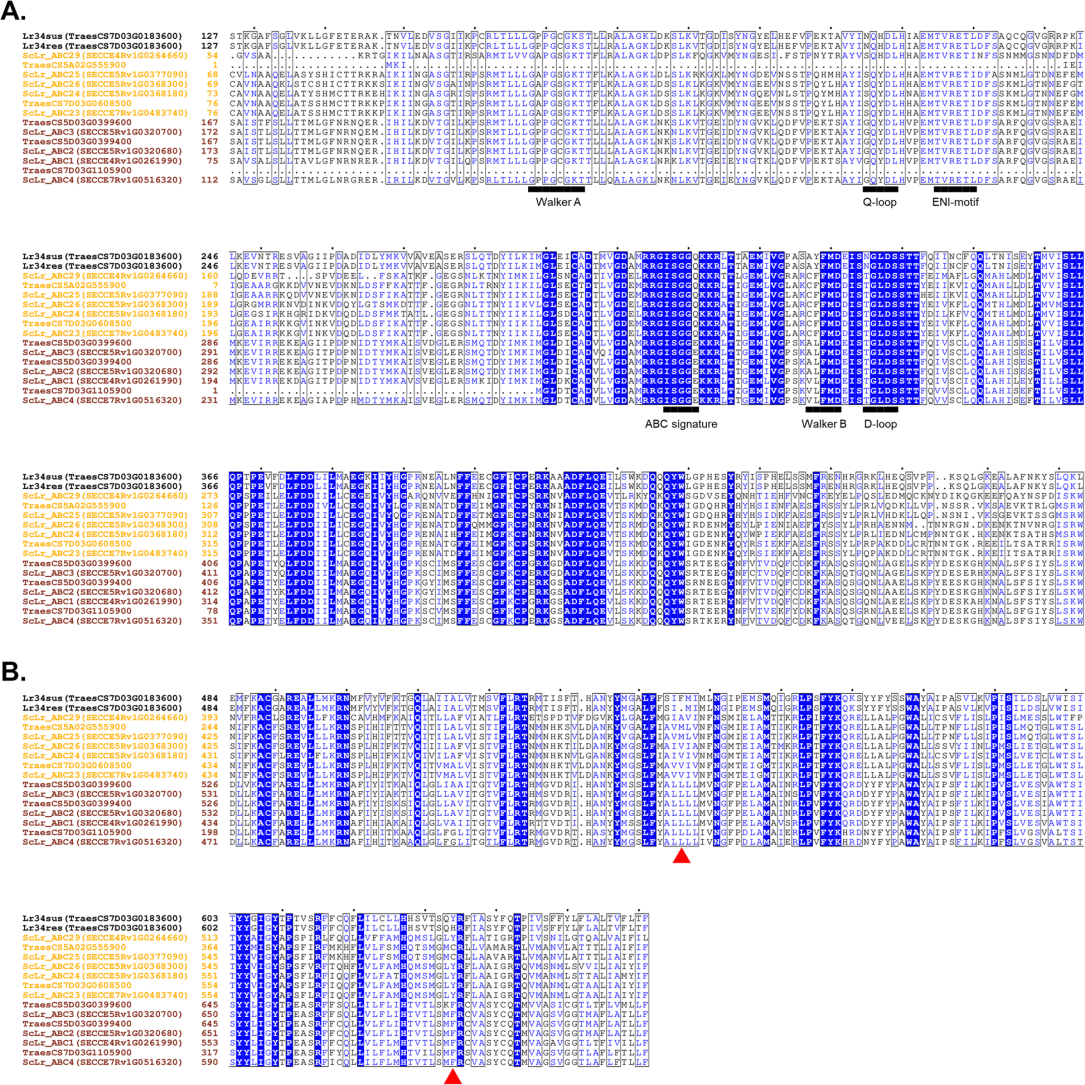
The alignment of selected fragments of rye and wheat ABCG proteins from 41, 45 and 50 clades. **A** NBD1s alignment of ABCG transporters. The deduced amino acid sequences of the “Walker A”, “Q-loop”, “ENI-motif”, “ABC signature”, “Walker B” and D-loop were marked.; **B** TMD1s alignment of ABCG transporters. Two regions surrounding the amino acids at positions 546 and 634, distinguishing the resistant and susceptible Lr34 variants, were highlighted

To investigate the differences in amino acid structure between clades 41 and 45, from which the transporters ScLr_ABC1 and ScLrABC_25 were selected for further study, we compared rye proteins belonging to clades 41 and 45 with their wheat homologs and with the Lr34 variants (resistant and susceptible) from clade 50 (Fig. 3). The sequence analysis revealed that nucleotide-binding domain 1 (NBD1s) of the examined ABC transporters include three characteristic motifs: Walker A, Walker B, and the ABC signature. The Walker A motif is a glycine-rich sequence involved in ATP phosphate binding. A typical sequence is GXXXXGK(S/T), where ‘X’ can be any amino acid, and ‘K’ (lysine) plays a key role in ATP binding [52]. The Walker B motif (ΦΦΦΦD), where Φ represents a hydrophobic amino acid, and ‘D’ (aspartic acid) is involved in coordinating the Mg²⁺ ions necessary for ATP hydrolytic activity [53]. The ABC signature motif, unique to ABC proteins and called the C-loop is responsible for transmitting conformational signals related to ATP hydrolysis [54]. In addition to these motifs, the NBDs contain three other characteristic sequences: the Q-loop, the ENI motif, and the D-loop. The Q-loop is named after its nearly invariable Q residue [55]. The highly conserved “ENI motif” (S/TФXD/ENФ; where X is any amino acid and Ф is a hydrophobic amino acid), also known as the “E-helix,” contains a highly conserved glutamate and functions in coupling between the NBD and transmembrane domains (TMD). Finally, a few residues downstream of the Walker B motif, many NBDs contain a D-loop, which, though quite diagnostic, has largely been ignored thus far. The observed subtle differences within some motifs of the NBD1s may explain the membership of these ABC transporters in different clades. Therefore, the amino acid residue N of Q-loop in clade 50 is replaced by G in clade 41 and S in clade 45. Q residue of the ABC signature in clades 50 and 45 is replaced by E residue in clade 41. In the D-loop of clade 50, N residue is found while in clades 41 and 45 residue T is present (Fig. 3A).

In addition to two highly conserved nucleotide-binding domains, ABCG transporters also contain two less conserved transmembrane domains. To investigate whether and to what extent the regions surrounding the amino acid residues positions 546 and 634, determining the Lr34res phenotype, have been conserved, we compared the TMD1 fragments of the chosen ABC transporters. We observed variability in these regions between chosen clades and relatively high conservativeness within individual clades (Fig. 3B).

In none of the studied clades, including the presented clades 41 and 45, we did not observe a deletion of F at position 546. However, we observed that the sequences of both rye and wheat from clade 41 around position 546 are highly conserved. We found much greater variability in clade 45. The greatest differences were observed between ScLr_ABC29 and the other members of this clade, followed by 100% identity between ScL_ABC25 and its wheat homolog. The sequence was highly conserved in ScLr_ABC28, ScLr_ABC24, and ScLr_ABC23 and their wheat homolog. The only difference was the presence of I residue in ScLr_ABC28 and ScLr_ABC24 instead of the V residue.

At position 634, there is an H residue in Lr34res, which is replaced by Y residue in Lr34sus. In the transporters from clade 41, an aromatic F residue is found at this position, while in the proteins from clade 45, a Y residue is present. The exception is ScLr_ABC25 and its wheat homolog, where a C residue is found.

### The comparison of predicted protein structural models

**Fig. 4:**
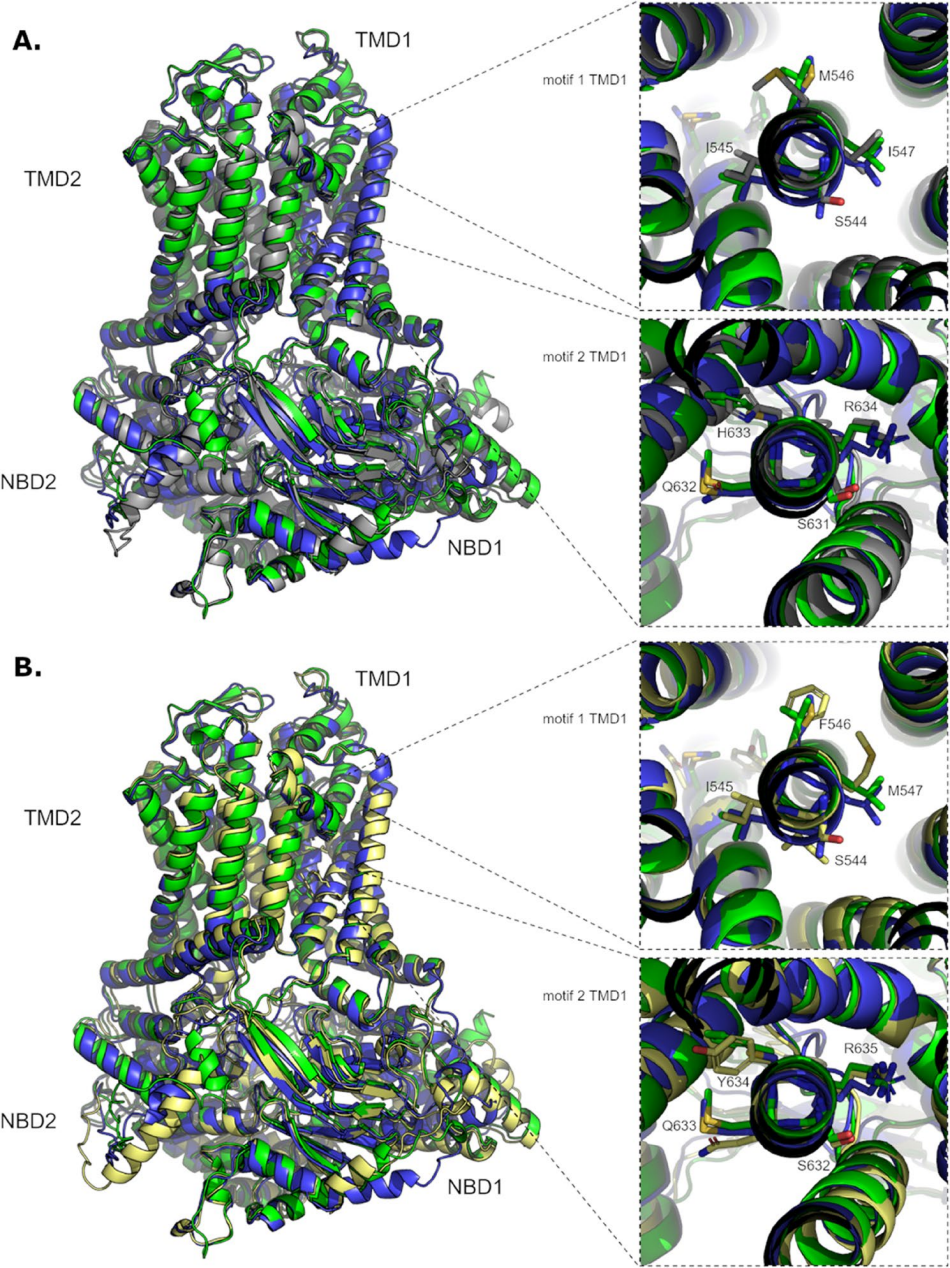
The predicted structural models of the selected rye ABC proteins with wheat Lr34. **A** Structural models of ScLr_ABC1 (green), ScLr_ABC25 (blue) with the reference sequences LR34res (gray). **B** Structural models of ScLr_ABC1 (green), and ScLr_ABC25 (blue) with the reference sequences LR34sus (yellow)

Bioinformatics analysis of wheat and rye proteins showed high conservation of the amino acid sequence of the proteins studied. Furthermore, the presence of mutations of Lr34res in a key substrate-binding region was identified: H633 corresponding to F584 in the ScLr_ABC1 protein, and C576 in ScLr_ABC25. The mutations found were identified in model structures (AlphaFold3) - and found to have no discernible effect on protein folding in this region. All six protein structure models are of high quality, with high confidence (pLDDT) and reliability (pTM) parameters: acn41354_1 (reference, grey) majority model at least pLDDT > 70 and pTM = 0.89; ScLr_ABC1 (green) majority pLDDT > 90, at least pLDDT > 70 and pTM = 0.89; ScLr_ABC25 (blue) majority pLDDT > 90, at least pLDDT > 70 and pTM = 0.88. Mer and the domain containing the key amino acids is of exceptionally high quality - the best part of the models. When 3 models with the reference sequences LR34 res (Fig. 4A) and with Lr34 sus (Fig. 4B) are superimposed, the key regions match almost perfectly despite mutations in the position of key amino acids for activity.

### Identification and characterization of Lr67 orthologs in rye Lo7 genome

The second APR gene we decided to investigate was Lr67. We performed a BLASTP analysis using the amino acid sequence of Lr67 as a query. That analysis allowed us to find 20 putative orthologs, which were annotated in Lo7 genome as “sugar transporters”. Deeper sequence analysis showed that those genes were involved in sugar transport and belong to 10 different protein families (Table 2). This diversity is reflected in the amino acid sequence similarity (from 50 to 99%) and query cover (78 −100%) predicted by BLASTP analysis. Further analysis of the sequence characteristics of the 20 rye sugar transporters proteins revealed that their sequence lengths ranged between 407 (ScLr_SUG15) and 529 (ScLr_SUG4) amino acid residues. The molecular weights of these proteins ranged between 43.83 and 57.83 kDa, while their theoretical isoelectric points (pI) ranged between 8.33 and 10.42, with an average pI value of 9.4 (Table S5).

**Table 2:** Identification of wheat Lr67 gene putative orthologs in rye Lo7 genome. Amino acid sequence of wheat Lr67 was the query to find putative rye orthologues (ScLr_SUG) by BLASTP analysis. Results were ordered in descending order of the sequence identity parameter. The highest prefix of *ScLr_SUG* gene the closest homology to wheat Lr67 protein

Name	Accession	Max score	Total score	Query cover	E value	Ident	Description
ScLr_SUG1	SECCE7Rv1G0472490.1	1029.6	1029.6	100%	0	99%	Sugar transport protein MST4
ScLr_SUG2	SECCE6Rv1G0415700.1	823.2	823.2	100%	0	75%	Sugar transport protein MST4
ScLr_SUG3	SECCE1Rv1G0048590.1	605.1	605.1	98%	0	62%	Sugar transport protein 8
ScLr_SUG4	SECCE2Rv1G0088070.1	623.6	623.6	98%	0	60%	Sugar transport protein MST6
ScLr_SUG5	SECCE5Rv1G0329650.1	611.7	611.7	98%	0	60%	Sugar transport protein 7
ScLr_SUG6	SECCE2Rv1G0097870.1	640.2	640.2	100%	0	59%	Sugar transport protein MST3
ScLr_SUG7	SECCE6Rv1G0428500.1	604.0	604.0	98%	0	58%	Sugar transport protein MST3
ScLr_SUG8	SECCE5Rv1G0329680.1	574.3	574.3	96%	0	57%	Sugar transport protein 7
ScLr_SUG9	SECCE5Rv1G0323150.1	565.8	565.8	97%	0	57%	Sugar transport protein MST3
ScLr_SUG10	SECCEUnv1G0529890.1	555.1	555.1	96%	0	57%	Sugar transport protein 7
ScLr_SUG11	SECCE5Rv1G0329670.1	538.1	538.1	97%	0	57%	Sugar transport protein 7
ScLr_SUG12	SECCEUnv1G0535310.1	597.0	597.0	99%	0	56%	Sugar transport protein MST5
ScLr_SUG13	SECCEUnv1G0548660.1	584.7	584.7	99%	0	56%	Sugar transport protein MST8
ScLr_SUG14	SECCE5Rv1G0323750.1	561.2	561.2	100%	0	54%	Sugar transport protein 14
ScLr_SUG15	SECCE6Rv1G0428600.1	443.0	443.0	78%	7.503e-153	54%	Sugar transport protein MST3
ScLr_SUG16	SECCE4Rv1G0268810.1	485.0	485.0	95%	1.074e-167	53%	Hexose carrier protein HEX6
ScLr_SUG17	SECCE5Rv1G0374180.1	548.9	548.9	99%	0	51%	Hexose carrier protein HEX6
ScLr_SUG18	SECCE2Rv1G0105390.1	501.5	501.5	99%	2.626e-174	51%	Sugar transport protein MST1
ScLr_SUG19	SECCE1Rv1G0032970.1	526.2	526.2	96%	0	50%	Hexose carrier protein HEX6
ScLr_SUG20	SECCE3Rv1G0160530.1	491.5	491.5	96%	2.254e-170	50%	Hexose carrier protein HEX6

Importantly in our dataset, only two proteins, ScLr_SUG1 and ScLr_SUG2, exhibited high sequence similarity (99% and 75%, respectively). The wheat Lr67 gene has 3 exons and 2 introns, and the protein is 514 amino acids long. Comparing this information with our results, ScLr_SUG1 had a similar organization but was shorter by 2 amino acids. The second candidate, ScLr_SUG2, had 4 exons and 3 introns, and the protein was 521 amino acids long (Table S5). Both proteins belong to the family “sugar transport protein MST4”, which is in line with NCBI gene annotation for Lr67. Interestingly, in the work of Moore and colleagues [19], Lr67 is identified as belonging to the sugar transport protein 13 based on similarity to *Arabidopsis thaliana* transporters. Our candidates ScLr_SUG1 and ScLr_SUG2 also showed similarity to the same group of transporters.

### Identification of rye sugar transport proteins and analysis of their phylogenetic relationship

**Fig. 5:**
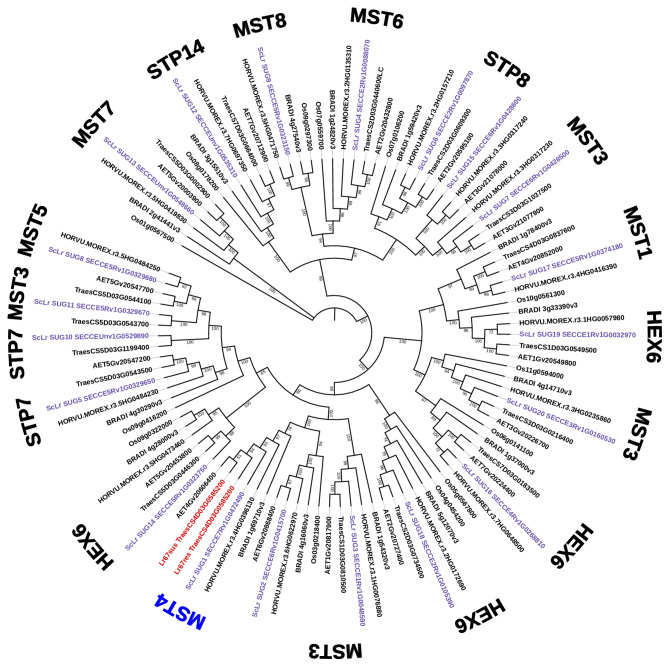
An unrooted phylogenetic tree of sugar transporters. The protein tree was constructed using the Maximum likelihood method. The bootstrap consensus tree was generated using 1000 replicates. Rye putative orthologues of Lr67 were classified into 10 subfamilies based on conserved domains. Wheat Lr67 proteins are depicted in red, rye proteins are depicted in purple, while barley, aegilops, sorghum and rice proteins are depicted in black

Homology comparisons were conducted with identified rye sugar transport proteins found by BLASTP analysis. The candidate proteins were further confirmed using the NCBI Conserved Domains Database to classify protein families (Table S6). A total of 20 rye sugar transport genes were used to find orthologues in 6 different species such as: *T. aestivum*,* H. vulgare*,* B. distachyon*,* A. tauchii*,* O. sativa* and *S. bicolor* to gain insights into their classification and evolutionary relationships. The phylogeny results revealed that analyzed gene family could be categorized into ten subfamilies: Sugar transport protein MST1, Sugar transport protein MST3-6, Sugar transport protein MST8, Sugar transport protein 7–8, Sugar transport protein 14 and Hexose carrier protein HEX6 (Fig. 5, Table S1). Notably, when we looked at APR gene Lr67 both variants susceptible and resistance were positioned on the same clade as ScLr_SUG1, with ScLr_SUG2 located in proximity. Identification of phylogenetic relationship inside sugar transporters prompted us to undertake more detailed analyses on ScLr_SUG1.

### Detailed gene structure of ScLr_SUG1 in APR and non-APR inbred lines

BLAST and phylogenetic analysis allowed us to find a good candidate to be orthologue of Lr67 in rye – ScLr_SUG1. Moore and co-authors [19] described two nonsynonymous single-nucleotide polymorphisms (SNPs) in exons 2 and 3, which were responsible for APR phenotype in wheat. In susceptible forms (Lr67sus) Gly144 and Val387 residues were present. However, in resistance form (Lr67res) that SNP caused mutation to Arg144 and Leu387. To investigate whether similar mutations were present in our studied populations in gene ScLr_SUG1 we decided to perform detailed gene structure analysis of lines with clear APR and non-APR phenotype. Seven different primer pairs were designed to sequence whole gene sequence including parts of 5’ and 3’ UTRs. Position of those primers were presented in Figure S2.

**Fig. 6:**
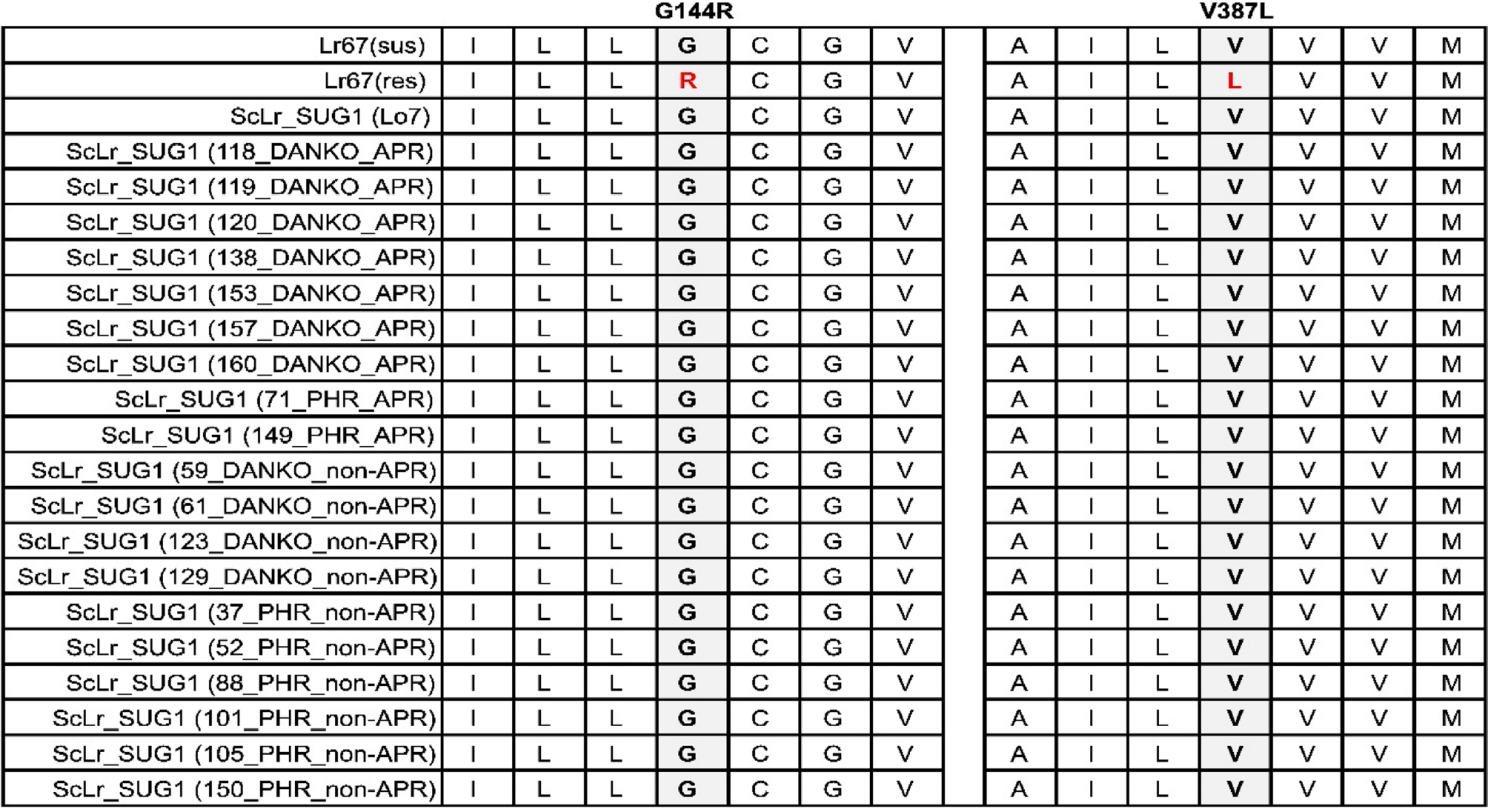
The sequence alignment of ScLr_SUG1 reveals mutations associated with the APR phenotype. A multiple sequence alignment and the regions containing the G144R and V387L mutations were schematically represented and highlighted with gray boxes. The alignment was generated using MUSCLE and subsequently edited with Jalview

Out of 25 lines provided by breeding companies, we were able to assemble the *ScLr_SUG1* gene structure for only 19 lines. Six inbred lines exhibited high polymorphism in regions where primers bind, resulting in non-specific PCR products or lack of amplicon (Table S7). Among the 19 fully sequenced inbred lines, 9 exhibited the APR phenotype (7 from DANKO and 2 from PHR), while the remaining 10 exhibited the non-APR phenotype (4 from DANKO and 6 from PHR). Contig assembly revealed sequence lengths ranging from 5290 nucleotides (line 138; APR DANKO) to 5880 nucleotides (line 52; PHR non-APR), with an average length of 5456 nucleotides. Interestingly, a detailed analysis of the obtained contigs indicated that FGENESH gene structure prediction for rye was slightly more accurate when using monocot-specific gene-finding parameters. Based on that, of the 19 lines, only two - line 119 (APR DANKO) and line 150 (non-APR PHR) exhibited a different gene structure with four exons. The remaining lines had 3 exons of similar length to the Lr67 gene (Table S8). After gene prediction analysis, the coding sequences were extracted. In wheat Lr67 resistance forms, two mutations were present: Gly144Arg and Val387Leu. However, neither of these mutations was found in any of the 19 inbred lines. (Fig. 6, Figure S5A, B). Additionally, no SNPs were found in the intron sequences that could be associated with the APR or non-APR phenotype (Figure S6).

### New candidates for the Lr67 ortholog in rye

**Fig. 7:**
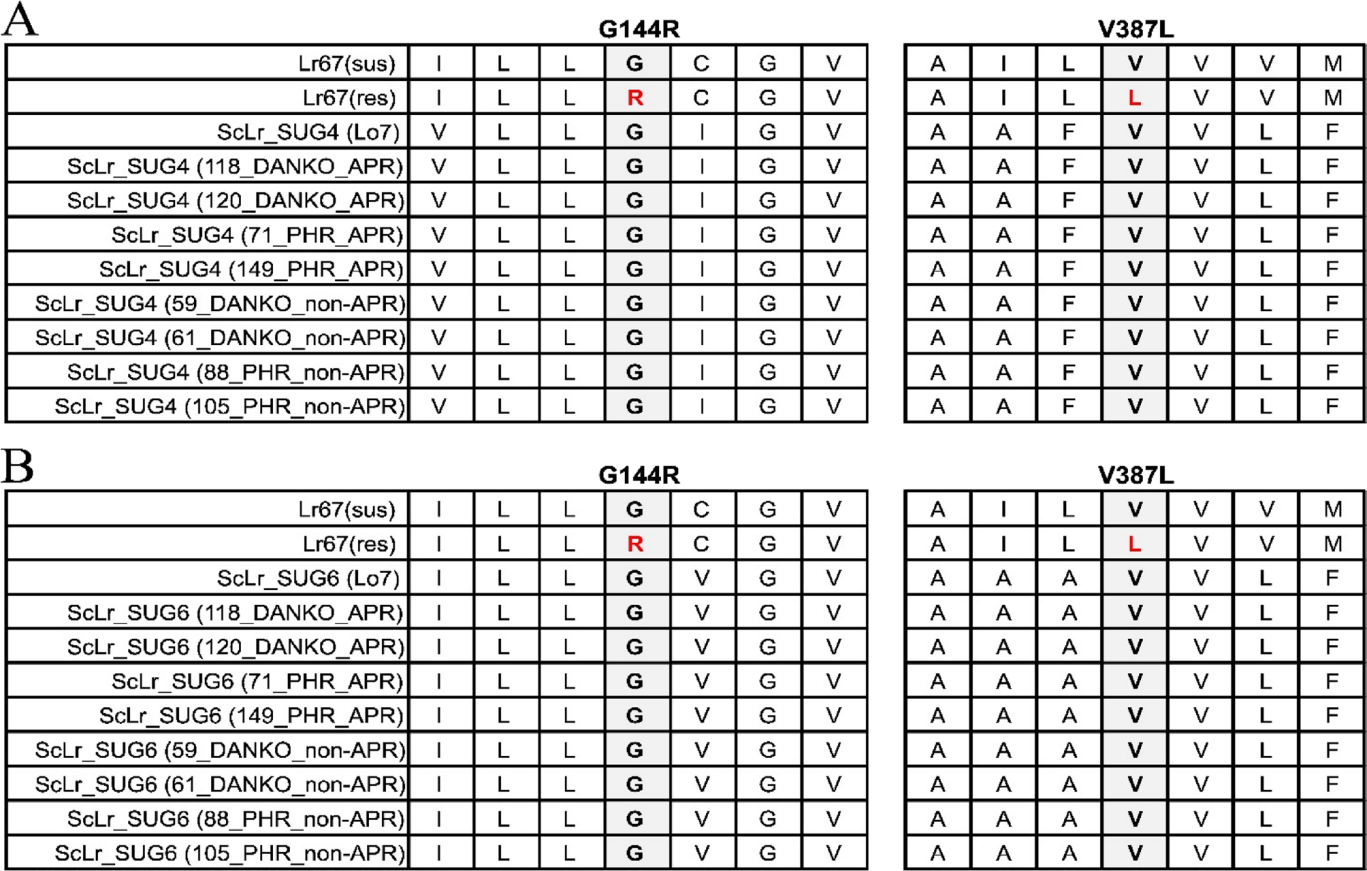
The sequence alignment of ScLr_SUG4 and ScLr_SUG6 reveals mutations associated with the APR phenotype. A multiple sequence alignment of two candidate genes ScLr_SUG4 (**A**) and ScLr_SUG6 (**B**) with the regions containing the G144R and V387L mutations were schematically represented and highlighted with gray boxes. The alignment was generated using MUSCLE and subsequently edited with Jalview

Due to the absence of polymorphisms associated with the APR phenotype in the *ScLr_SUG1* gene across the analyzed lines, we extended our search for additional candidate genes. Further structural analysis was narrowed down to 8 lines - two APR and two non-APR from each of the two breeding companies. Due to the high sequence similarity to other sugar transporter family genes, designing specific primers for sequencing the SNP-containing regions of the *ScLr_SUG2* gene was unsuccessful. Therefore, we decided to reanalyze our transcriptomic data obtained from three inbred lines infected with compatible and incompatible *Prs* isolates for further investigation [37]. This approach led to the identification of nine differentially expressed genes (*ScSUG-1*; *ScSUG-4*; *ScSUG-5*, *ScSUG-6*; *ScSUG-14*; *ScSUG-16*; *ScSUG-17*; *ScSUG-18*; *ScSUG-20*). By integrating these findings with sequence similarity data (Table 2), we identified two additional candidate genes, *ScLr_SUG4* and *ScLr_SUG6*, for subsequent sequencing analysis. *ScLr_SUG6* belongs to the “Sugar Transport Protein MST3” family, while *ScLr_SUG4* is a member of the “Sugar Transport Protein MST6” family, with sequence similarities to Lr67 of 60% and 59%, respectively (Table 2). Interestingly, both genes exhibit increased expression at 36 h post-inoculation in our RNA-seq data [37]. The expression of *ScSUG-4* is induced exclusively in response to infection by the compatible *Prs* isolate (line L318: 0.76 log2FC; line D39: 0.92 log2FC). In contrast, *ScSUG-6* shows up-regulation in response to both the compatible isolate (line L318: 1.03 log2FC) and the incompatible isolate (line D33: 0.59 log2FC). Despite the positive indications from RNA-seq analysis, no specific SNPs associated with the APR phenotype were identified among the analyzed lines. (Fig. 7; Figure S7A, B).

### Analysis of expression of chosen *ScLr_ABC* and *ScLr_SUG* genes

**Fig. 8:**
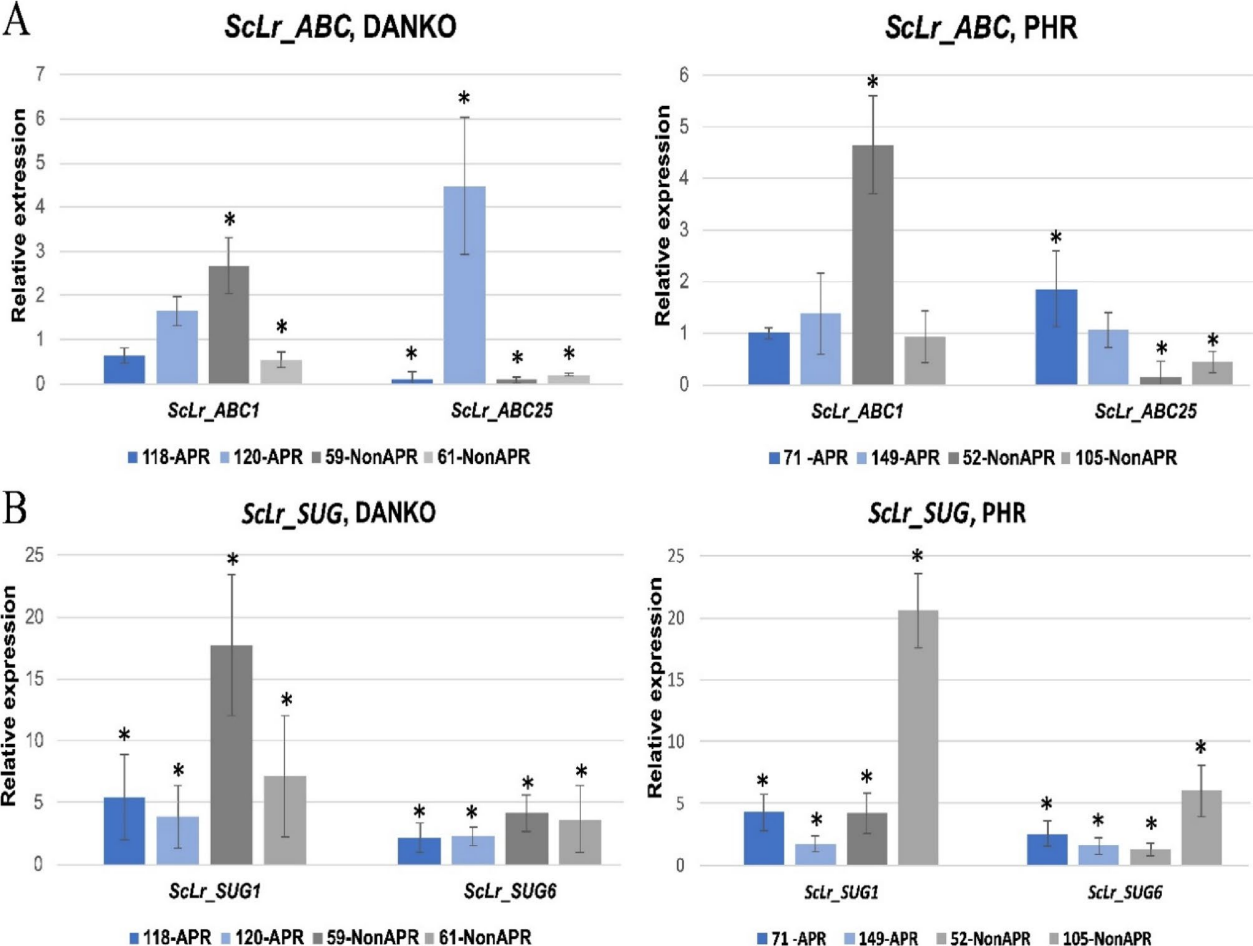
Analysis of the expression patterns of chosen *ScLr_ABC* and *ScLrSUG* genes. Relative expression patterns of *ScLr_ABC1* and *ScLr_ABC25* (**A**) and *ScLr_SUG1* and *ScLr_SUG6* (**B**) in APR and non-APR rye lines from DANKO and PHR Polish Breeding companies infected with the mixture of *Prs* spores performed using RT-qPCR

In the RT-qPCR experiment, we examined the expression of four genes, *ScLr_ABC1* and *ScLr_ABC25* and *ScLr_SUG1* and *ScLr_SUG6* in APR and non-APR lines derived from DANKO and PHR companies. With a few exceptions, namely *ScLr_ABC25* in line 120 from DANKO (especially) and in line 71 from PHR both genes and both variants of them were characterized by higher expression in plants of the non-APR lines. The highest expression level among plants of the APR lines was measured for *ScLr_SUG1* gene in line 118 from DANKO and PHR, and for *ScLr_ABC25* gene in line 120 from DANKO. Among non-APR plants, the highest level of expression was recorded for *ScLr_SUG1* in line 59 from DANKO and in line 105 from PHR (Fig. 8). In turn, the lowest expression levels were found for *ScLr_ABC25* gene in one APR line, 118 from DANKO and three non-APR lines, 59 and 61 from DANKO, and 52 from PHR.

### Phenotyping of APR at late seedling stage

**Fig. 9:**
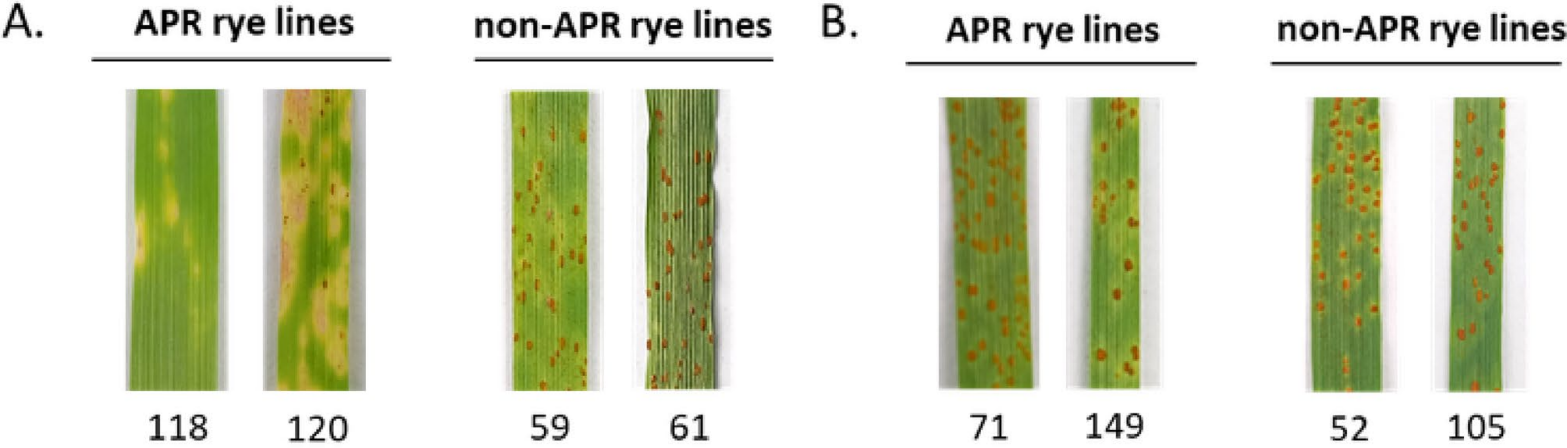
Analysis of disease symptoms of *Puccinia recondita* f. sp. *secalis*. Macroscope observation of the type of infection on APR and non-APR rye lines from two Polish Breeding companies: (**A**) DANKO Plant Breeding and (**B**) Poznań Plant Breeding, PHR at 10 days after inoculation

We were unable to detect LTN in all investigated lines at 10 dai, which is one of the characteristics of the APR phenotype. However, LR symptoms were observed in all lines at 10 dai (Fig. 9) and evaluated using the 6-point Murphy’s scale [48]. In case of materials from DANKO, the infection types of both inoculated APR lines were scored as 0; (chlorotic or necrotic flecking) and as 1 (minore uredinia surrounded by chlorosis or necrosis) for lines 118 and 120, respectively. The infection type of both non-APR lines (59, 61) was rated as 4 (large-sized uredinia with little or no chlorosis) (Fig. 9A). The disease symptoms in all PHR-derived lines, both APR (71, 149) and non-APR (52, 105) were very similar after 10 dai and the corresponding infection type was rated as 4 (Fig. 9B). Phenotyping of APR lines from DANKO and PHR indicated potentially different mechanisms of rye response to *Prs* at late seedling stage.

## Discussion

APR, as a type of resistance to a usually wider spectrum of fungal pathogens of cereals, mainly fungi of the genus *Puccinia*, and additionally providing long-term resistance [1–3], is a very desirable trait for breeders and producers. An institution as important as CIMMYT strongly recommends APR as an effective strategy for breeding wheat varieties that have durable resistance to rusts (https://www.cimmyt.org/content/uploads/2021/06/CIMMYT-Strategy-for-Adult-Plant-Resistance-APR.pdf)*.*

Nevertheless, apart from the very well-known genetic determinant of this resistance in wheat, in other cereals, such as barley, rice, maize and sorghum knowledge on this subject is limited and all results come from the analysis of transgenic forms with introduced wheat *Lr34res* and *Lr67res* genes [6, 7, 14, 27–30]. It is known well that in rye the APR phenotype also occurs [4, 5, 56]. It should be added, however, that in the second cited work [7], the adult plant partial resistance was analyzed, not the true APR. Until the research presented in this paper was undertaken, we knew practically nothing about the molecular basis of APR in rye. So far, only Gruner et al. [5] has performed molecular analyses that led him to identify several QTL for APR, but these loci were associated with resistance to stem rust, not LR. Therefore, we decided to undertake the in-depth molecular studies including structural, phylogenetic and expression analyses of two groups of genes: the first were the genes encoding ABC transporters (*ScLr_ABC*), and the second - sugar transporters (*ScLr_SUG*) supported by precise phenotyping of numerous collections of rye inbred lines from two Polish breeding companies for the APR trait.

### Phenotyping APR to LR in rye

Although no reliable and comprehensive studies on APR in rye have been conducted to date, our findings also suggest that this type of resistance to LR occurs in the species at a very low frequency. We focused on the increase of disease severity (IDS). This approach assessed the increase in disease severity within a 10-day interval across all tested genotypes and due to constant period between disease rating was directly related to area under the disease-progress curve (AUDPC). Under conditions of natural infection by LR, less than 10% of the lines of all lines evaluated showed slow rusting with a null value of IDS. The exception was the lines phenotyped in DANKO in 2020, for which IDS was 23,6%. A similar share of lines with the APR phenotype (25%) was observed by Miedaner et al. [4]. However, it is difficult to compare these results with ours because the cited authors assessed a much smaller number of genotypes (that is 16) in this respect.

Despite the generally low frequency of lines with the APR phenotype, the expression of the APR trait varied both between locations and between years. The course and development of LR is influenced by the host genotype, the pathogen variation and location conditions, as it was shown in our work and in the research of other authors dealing with rust fungi (for review see: Kolmer et al. [57]). Miedaner et al. [58] demonstrated that the virulence structure of *Prs* evolved over a long-term period, with increasing complexity observed within a single cropping season. Over the three-year experiment, 93, 201, and 125 pathotypes were identified, and the Evenness index values, ranging from 0.88 to 0.92, stressed the absence of a dominant pathotype in the fungal population. The variability may arise directly from the pathogen’s biology, including the potential for sexual recombination. Within the Pucciniomycota, less commonly acknowledged processes, such as somatic fusion [59, 60], hybridization [61, 62] as well as factors affecting the structure of genetic population, such as migration, can also contribute to this variability. The migration of *Puccinia recondita* f. sp. *secalis* can occur through long-distance spore dispersal [58]. This mechanism has been shown to introduce unexpected new virulence in brown rust [63] and yellow rust [64] pathogens.

The severity of LR infection is clearly influenced by the host plant [58, 65–67]. Research on the susceptibility of Polish rye breeding lines has demonstrated that the origin of the plant material, particularly its gene pool, significantly impacts the extent and severity of infection [67]. Phenotyping of inbred lines for LR severity performed in DANKO and PHR, presented in this work, further support these findings.

As discussed above, environmental factors play a crucial role in the development of the *Prs*, however Racca et al. [68] highlighted that, even under the same conditions, the dynamics of epidemic progression can differ significantly due to the varying stages of uredospore ontogenesis.

### Phylogenetic analysis of *ScLr_ABC* and *ScLr_SUG* genes

We initially assumed that orthologs of genes *Lr34* and *Lr67* with an already known function in conferring APR to LR in close related species, primarily wheat and barley would be found among them. Thus, the first step in our research was a detailed bioinformatic phylogenetic analysis of both genes.

### ScLr_ABC

We conducted a BLAST search to identify genes that encode amino acid sequences similar to *Lr34* in the rye genome Lo7 published by Rabanus-Wallace et al. [69]. All 29 rye-identified sequences, just like wheat *Lr34*, belong to the subfamily ABCG of ATP-binding cassette transporters. However, the similarity of these sequences was not very high (from 42% for ScLr_ABC29 to 57% for ScLr_ABC1). Southern blot and EST sequence analysis of Krattinger et al. [70], even though the rye genome was not known at the time, suggested the absence of *Lr34* orthologs in the rye. The authors showed that alleles of resistant (Lr34res-D) and susceptible (Lr34sus-D) wheat cultivars differed by only three polymorphisms, two of which involve helix 2 (TMX2) and 4 (TMX4) of the first transmembrane domain (TMD1). TMDs of ABCG transporters are less conserved than NBDs however their structure is a key in the transport across the cell membrane. We did not observe these polymorphisms in predicted rye proteins. In the selected sequences of rye and wheat from 50, 41, and 45 clades we did not detect the deletion of F residue, but the regions around this position were highly conserved among the sequences of individual clades. In the case of position 633 (in Lr34res), instead of H residue, we found F residue in members of 41 clad (ScLr_ABC1, 2, 3, and 4), Y residue in members from 45 clad however in ScLr_ABC25 and its homolog from wheat A genome we found C residue. The 3D structural locations of two mutations in LR34res and LR34sus were analyzed by Cloutier et al. [51] who showed that they are located in the TMD1 close to the putative substrate binding sites. The authors suggest that the aromatic side chain of the phenylalanine (F546) residue in LR34sus, positioned in TMH-2, acts as a structural barrier within the substrate translocation channel. The second mutation involves a histidine (H) in LR34res being replaced by a tyrosine (Y) in LR34sus, located in TMX4 of TMD1, close to the substrate binding sites. Tyrosine residues help stabilize positive charges within the membrane’s electric field and play a role in facilitating substrate transport [71, 72]. On the other hand, histidine residues are crucial in transmembrane domains (TMDs) of transmembrane proteins [73–75]. Histidines are known for undergoing pH-dependent protonation changes, which can lead to alterations in transmembrane helix conformation, modification of pore size, and influence substrate transport activity [76–78]. Thus, it is reasonable to speculate that substituting tyrosine with histidine in LR34sus may enhance structural stability, flexibility, and trigger conformational changes affecting substrate translocation activities. Our observations, i.e. the relatively low level of similarity, the absence of rye representatives in the 50th clade, to which Lr34 belongs and the lack of key polymorphisms characteristic of Lr34res allele (the F deletion and the presence of a conserved H residue in TMD1), lead us to the conclusion confirming the findings of Krattinger et al. [70], that the *ScLr_ABC* transporters in rye are not orthologs of wheat *Lr34*. Accordingly, our results can be considered decisive in this matter, because we based them on the already known rye genome, while the above-cited authors did not have such knowledge.

Nevertheless, we cannot rule out that among the numerous rye genes encoding transporters, there are genes related to APR. One of them: the gene encoding the ScLr_ABC25 transporter could be a good candidate. The bioinformatic analyses, the observed increased level of *ScLr_ABC25* expression in APR rye lines, and a reduced expression in non-APR lines support this hypothesis. Our phylogenetic analysis showed that 29 rye transporters were found in 14 ABCG subfamily clades. The members of ABCG are characterized to have two cytosolic domains known as the ATP-binding cassettes or the nucleotide-binding domains (NBDs) and two less conserved trans-membrane domains (TMD) [43]. We found characteristic motifs of NBD in identified rye sequences and analyzed them in selected rye and wheat transporters from 50, 41, and 45 clades. The observed differences in NBD motifs indicate variations between the transporters of the studied clades. Hwang et al. [79] suggest that genes encoding ABC transporters may have experienced multiplication and diversification throughout evolution, enabling plants to better adapt to terrestrial environments. Alternative splicing (AS) is a crucial posttranscriptional regulatory mechanism that produces multiple transcript and protein isoforms from a single gene [80]. The ABC transporter in rice, known as OsPDR1 (OsABCG45), generates three splice isoforms: OsPDR1.2, OsPDR1.3, and OsPDR1.1. Interestingly, OsPDR1.2 and OsPDR1.3 contain a conserved glutamate residue within the “ENI-motif” of the first nucleotide-binding domain, while this feature is absent in OsPDR1.1. Mutations affecting this conserved residue in the “ENI-motif” are expected to impair transport function by disrupting the interaction between the NBD and TMD domains. Plants overexpressing OsPDR1.2 and OsPDR1.3 showed enhanced disease resistance compared to those overexpressing OsPDR1.1. We observed that all analyzed ABC transporters contain a conserved glutamate residue in the „ENI-motif” meaning that crosstalk between NBD and TMD works smoothly.

We did not observe the key polymorphisms of Lr34res in rye transporters but there was no discernible effect on protein folding in this region. The high 3D structural similarity of the selected rye proteins (ScLr_ABC1, ScLr_ABC25) to those from wheat confirms observations of Cloutier et al. [51] that the properties of amino acids residue in substrate binding site determine the functions of ABC proteins. Due to their hydrophobic nature, aromatic amino acids present at the substrate binding site (F and Y residue in members of 41 and 45 clade respectively) may participate in interactions with hydrophobic ligands such as lipids and sterols. These amino acids may stabilize the binding of these substrates through these interactions. In contrast, the cysteine residue in ScLr_ABC25 differs from phenylalanine (F) and tyrosine (Y) in its chemical properties. It is smaller and contains a reactive thiol group (-SH), which can form disulfide bridges or react with ligands containing reactive groups. The substitution of Y or F with a C residue in key positions may alter the way in which protein binds the substrate, affecting substrate specificity, affinity, and binding stability. Cysteine, with its thiol group.

(-SH), such as histidine with imidazole group in Lr34res, can potentially engage in specific interactions with certain ligands that require more reactive amino acid residues for binding. In cases where the ABCG protein’s substrate requires interactions with thiol groups, cysteine could enhance the ability to transport these specific molecules that may be crucial for APR in the rye.

To sum up the above considerations, the results presented in this paper did not reveal sufficiently high similarities for none of the analyzed *ScLr_ABC* sequences to the *Lr34* gene. Nevertheless, our search led to the identification of several sequence variants of the *ScLr_ABC* gene that show relatively high similarity to *Lr34* (but not high enough to be considered orthologs of this gene) and their expression profiles (discussed later in this section) may indicate their role in the immune response to LR; including the APR-type response.

### ScLr_SUG

Another well-known multi-pathogen resistance gene is the *Lr67* locus (Lr67/Yr46/Sr55/Pm46), which, like Lr34, confers resistance to leaf rust, stripe rust, stem rust, and powdery mildew in wheat [22]. To find putative orthologues of Lr67 in rye we performed BLAST search against Lo7 genome published by Rabanus-Wallace et al. [69]. All 20 rye-identified sequences, just like wheat Lr67, belong to the sugar transporters family. However, the similarity of those sequences ranged from 99% (ScLr_SUG1) to 50% (ScLr_SUG20). ScLr_SUG1 and ScLr_SUG2, the most similar proteins, were classified as Sugar Transport Protein 13 (STP13), which has highly conserved orthologs across plant species [19]. When examining the APR gene *Lr67*, both the susceptible and resistant variants were positioned within the same clade as *ScLr_SUG1*, with *ScLr_SUG2* located nearby. By comparing sequence similarity between the Lr67 protein and ScLr_SUG1, as well as the phylogenetic distance between these sequences, we postulate that ScLr_SUG1 is an ortholog of Lr67. Consequently, we pursued an in-depth structural analysis of rye *ScLr_SUG1* in lines exhibiting APR and non-APR phenotypes.

Successful assemble of *ScLr_SUG1* gene structure was achieved for 19 lines, of which 9 exhibited the APR phenotype, while the remaining 10 were non-APR. Analysis of canonical SNPs associated with the APR phenotype in Lr67 - specifically, Gly144Arg and Val387Leu - revealed no APR-linked polymorphisms in rye. None of the analyzed lines carried mutations in regions encompassing the G144R or V387L variants, nor were any intronic SNPs detected that could distinguish APR from non-APR phenotypes.

Interestingly another loss-of-resistance mutations in Lr67res such as C75Y, E160K, G208D, and G217D described by Moore et al. [19] and functionally characterized in *X. laevis* oocytes system and in yeast [22, 81] suggest a role for altered anion fluxes in the Lr67res phenotype were absent in ScLr_SUG1 in our studied lines. Moreover, Milne et al. [81] postulate that a disulfide bond between Cys77 and Cys449 in the lid domain, identified in the AtSTP10 crystal structure [82] may be also required for the Lr67res function. However, in this case as well, we did not observe any differences in the lines we studied.

Due to the absence of polymorphisms associated with the APR phenotype in the ScLr_SUG1 gene across the analyzed lines, we broadened our search to additional candidate genes. High sequence similarity among rye sugar transporter genes complicated primer design for SNP-containing regions in ScLr_SUG2, preventing effective sequencing.

Therefore, we sought to identify potential orthologs of Lr67 responsible for the APR phenotype by examining gene expression changes during infection by *Prs*. To this end, we analyzed our transcriptomic data from three inbred lines infected with both compatible and incompatible *Prs* isolates [37]. This unique dataset al.lowed us to assess the expression changes of genes within the ScLr_SUG family at early stages of *Prs* development - specifically at 20- and 36-hours post-infection. Among the 20 potential Lr67 orthologs, only 9 sugar transporters exhibited differentially expressed genes level: *ScSUG-1*,* ScSUG-4*,* ScSUG-5*,* ScSUG-6*,* ScSUG-14*,* ScSUG-16*,* ScSUG-17*,* ScSUG-18*, and *ScSUG-20*. Phylogenetic analysis and BLAST analyses highlighted, ScLr_SUG4 and ScLr_SUG6 as additional candidate genes, prompting further structural investigation in a subset of APR and non-APR lines selected from various breeding sources. The expression of *ScLr_SUG4* appears to be exclusively induced during compatible reaction (line L318 and D39). In contrast, *ScLr_SUG6* exhibits up-regulation in response to both the compatible isolate (line L318) and the incompatible isolate (line D33). Although RNA-seq analysis suggests potential relevance, no APR-specific SNPs were identified across examined lines. These findings raise the possibility of additional genetic factors influencing the observed expression patterns and their association with the APR phenotype. Milne et al. [22, 81] proposed that changes in ion fluxes may play a critical role in Lr67res pathogen resistance, with Lr67res plants displaying increased Cl⁻ uptake and concurrent Na⁺ accumulation, maintaining electroneutrality and exhibiting a leaf-tip necrosis phenotype. While similar roles may be filled by other rye sugar transporters, validation of this hypothesis will require further investigation.

Notably, ABA the transport substrate for Lr34 [17], has been shown to induce Cl⁻ fluxes through channel mediation in guard cells [83]. This multi-dimensional nature of the APR response further complicates understanding the mechanism of this reaction in rye.

Moreover, race-specific R genes have also been associated with altered ion fluxes in plant cells, albeit via a mechanism distinct from both Lr67res and Lr34res-associated ion transport. In this case the assembly of the ZAR1 resistosome at the plasma membrane enables Ca²⁺ fluxes, which trigger hypersensitive responses [84].

Interestingly, Lr34 and Lr67 exhibit cross-species functionality and, when expressed as transgenes in other species such as barley, rice, maize and sorghum, confer resistance to pathogens that do not infect wheat [14]. This cross-functionality is highly valuable for creating novel resistance sources in crops with limited or underdeveloped germplasm and genetic resources. Usage of non-canonical resistance genes in breeding programs could be beneficial due to rapid pathogen evolution, which can overcome major resistance genes in commercial cultivars and become a threat for cereals production [85, 86].

### Macroscopic observations of LR symptoms at late seedling stage

To complement the analysis of expression of *ScLr_ABC* and *ScLr_SUG* genes at the seedling stage, we also performed macroscopic observations to detect differences in the response to *Prs* infection of young rye plants.

Considering the specificity of APR, we would expect the symptoms of this immune response to LR to occur in the adult plant stage. On the other hand, based on the results of the expression analysis (discussed above), it could be expected that the APR phenotype would become apparent much earlier. Meanwhile, lines from DANKO and PHR reacted differently. In the case of both APR lines originating from DANKO, disease development was suppressed already on four weeks old seedlings, what was detected 10 days after infection. To clarify whether this is a genotype-dependent reaction, we infected these lines with a mixture of spores from another location, i.e. Wiatrowo (PHR) and we observed a very similar response as in the case of using the spore mixture from DANKO (Figure S8). Plant materials from PHR reacted in a completely different way - in this case, the reaction of four-week-old seedlings to LR was the same in APR and non-APR lines; this concerned both the number of uredinia produced as well as the presence of chlorosis. However, based on field observations based on field observations all PHR lines had a clearly determined phenotype - APR or non-APR at the adult plant stage.

The different dynamics of activating the APR immune response in materials bred in both companies can be explained by their relatively high genetic diversity (as we showed in our previous works [87, 88]), and therefore by the different way and time of expressing the APR trait - at the late seedling stage vs. at the mature plant stage in the case of rye inbred lines from DANKO and PHR, respectively.

### Analysis of expression of *ScLr_ABC* and *ScLr_SUG* gene variants

To verify the potential role of *ScLr_ABC* and *ScLr_SUG* genes in APR, but related to their expression and not structure, we performed the qRT-PCR analysis. Based on the results of the previous works of Milne et al. [14], Rinaldo et al. [11], McCallum et al. [12] and Omara et al. [13] who showed that the APR related genes also confer seedling resistance in rye relatives, namely wheat and barley, we performed these analyzes on young, 5-week-old plants of four APR (two from each company) and four non-APR lines (two from each company). The results of this analysis, however, are not fully clear. Although in all cases *Prs* infection resulted in an increase in the relative expression of *ScLr_ABC* and *ScLr_SUG* genes, especially *ScLr_SUG1*, it is difficult to find an unambiguous relationship between the gene expression profile and the type of resistance to LR. Nevertheless, our results with respect to *ScLr_SUG1* gene suggest its association with non-APR phenotype. In turn, *ScLr_ABC25* seems to determine APR-type immunity. This variant could be the candidate providing the basis for elaboration of marker assisted selection (MAS) aimed at the selection of rye forms with the APR phenotype so sought after by breeders. In our previous work [37] we showed that the latter variant, *ScLr_ABC25* gene, is upregulated after LR infection of young rye seedlings, in a compatible plant-pathogen interaction. This may indicate an important role of this gene in multi-stage resistance to LR.

## Conclusions

The results presented in this paper are the first attempt to find genetic determinants of APR resistance to LR in common rye. They show that the mechanism of this type of resistance is different in rye than in other cereals studied in this respect (mainly wheat and barley). This is especially true for genes *ScLr_ABC*, whose sequence and lack of polymorphisms translating into the APR phenotype allow us to state that rye does not possess orthologs of the wheat Lr34 gene. Conversely, in the case of genes encoding sugar transporters, we believe that rye possesses ortholog(s) of the Lr67 gene, although, similarly to the previous group of genes, we did not find any polymorphisms identified in the wheat gene. The obtained results are a good starting point for further works which because of advancements in high-throughput technologies such as RNA-seq, sRNA-seq, and MutRenSeq, exploring the diversity and durability of LR resistance could become significantly easier and efficient.

From the more practical point of view, at the moment, our best APR-conferring candidate is gene ScLr_ABC25, which could be the basis for creating breeding programs aimed at selecting forms with the APR resistance type - so sought after by breeders. It is so important and necessary that, unfortunately, to the best of our knowledge, there are currently no specific breeding programs in rye aimed at developing forms with APR-type resistance to rust diseases.

To sum up, we hope that our work has provided insights into APR in rye and indicated potential directions for further basic and applied research.

## Supplementary Information

Supplementary Material 1.

Supplementary Material 2.

Supplementary Material 3.

Supplementary Material 4.

Supplementary Material 5.

Supplementary Material 6.

Supplementary Material 7.

Supplementary Material 8.

Supplementary Material 9.

Supplementary Material 10.

Supplementary Material 11.

Supplementary Material 12.

Supplementary Material 13.

Supplementary Material 14.

Supplementary Material 15.

Supplementary Material 16.

Supplementary Material 17.

Supplementary Material 18.

## Data Availability

All data generated or analyzed during this study are included in this published article and its supplementary information files. The raw RNA-seq (fastq) data are deposited in the NCBI database (BioProject: PRJNA888031).
