# Supplementary material for: Searching for genes determining the APR phenotype in rye
Source: BMC Plant Biol. 2025 Jul 19;25:935. doi: 10.1186/s12870-025-06920-0 (PMC12275401; doi:10.1186/s12870-025-06920-0)
Supplement: Supplementary file 1 — Supplementary Material 1. [file 12870_2025_6920_MOESM1_ESM.pdf]

DANKO

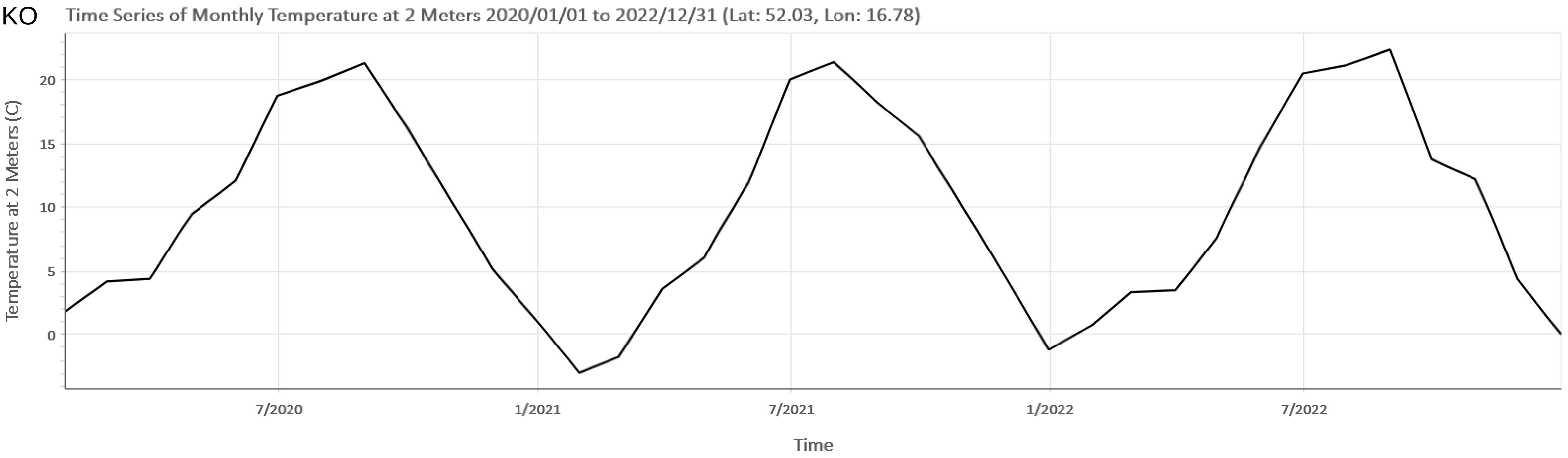
Time Series of Monthly Temperature at 2 Meters 2020/01/01 to 2022/12/31 (Lat: 52.03, Lon: 16.78)
Temperature at 2 Meters (C)
20
15
10
5
0
7/2020
1/2021
7/2021
1/2022
7/2022
Time

PHR

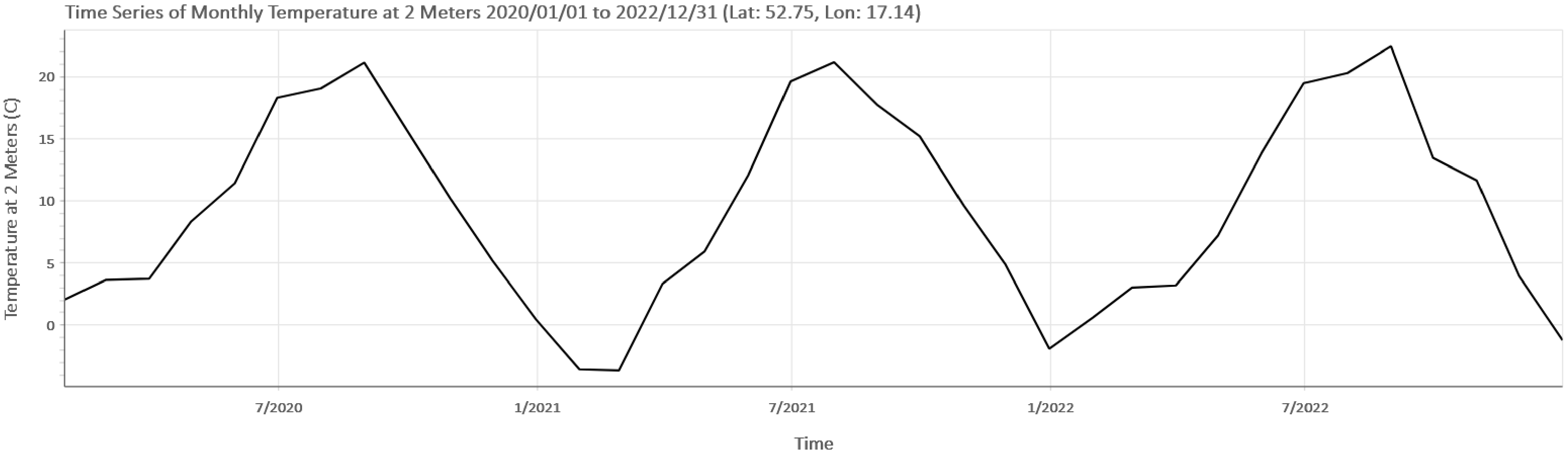
Time Series of Monthly Temperature at 2 Meters 2020/01/01 to 2022/12/31 (Lat: 52.75, Lon: 17.14)
Temperature at 2 Meters (C)
20
15
10
5
0
7/2020
1/2021
7/2021
1/2022
7/2022
Time

DANKO

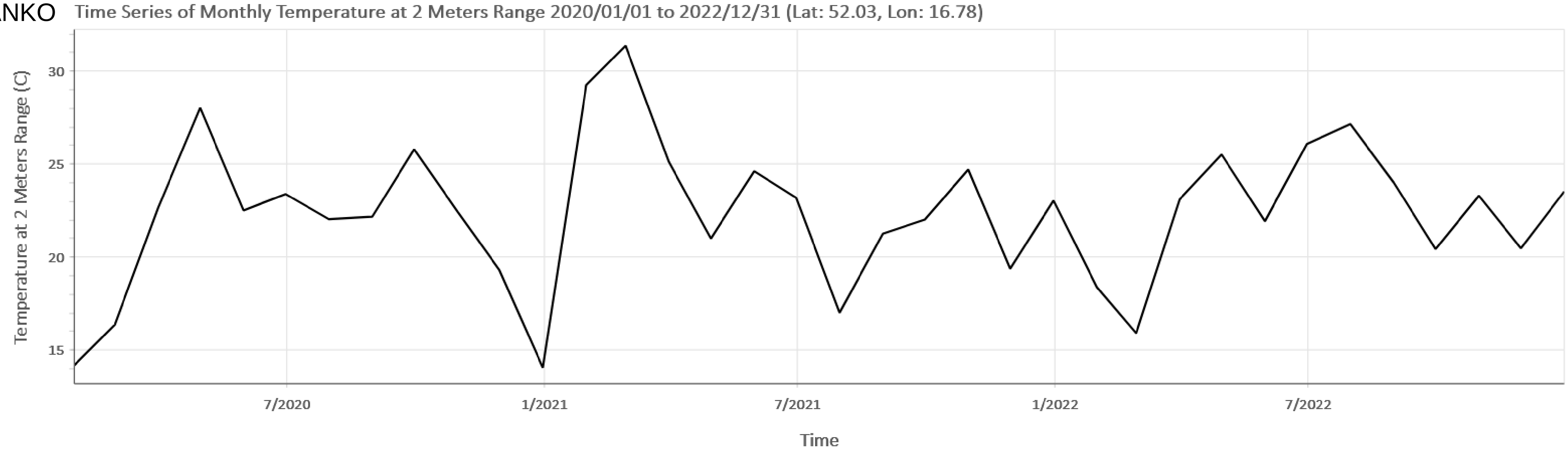
Time Series of Monthly Temperature at 2 Meters Range 2020/01/01 to 2022/12/31 (Lat: 52.03, Lon: 16.78)
Temperature at 2 Meters Range (C)
30
25
20
15
7/2020
1/2021
7/2021
1/2022
7/2022
Time

PHR

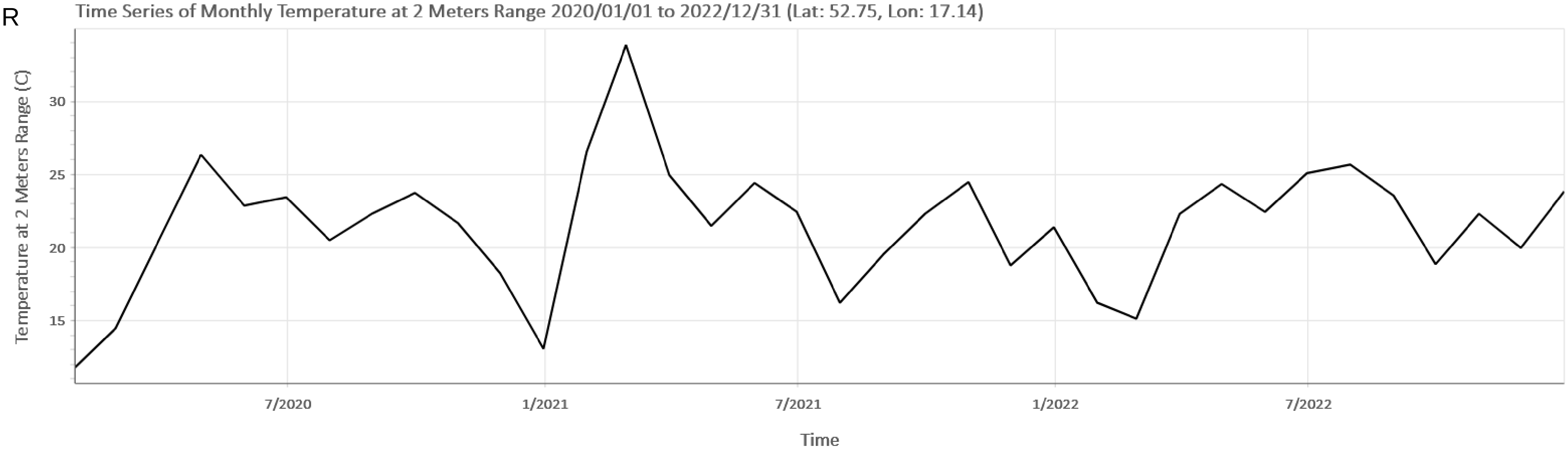
Time Series of Monthly Temperature at 2 Meters Range 2020/01/01 to 2022/12/31 (Lat: 52.75, Lon: 17.14)
Temperature at 2 Meters Range (C)
30
25
20
15
7/2020
1/2021
7/2021
1/2022
7/2022
Time

DANKO

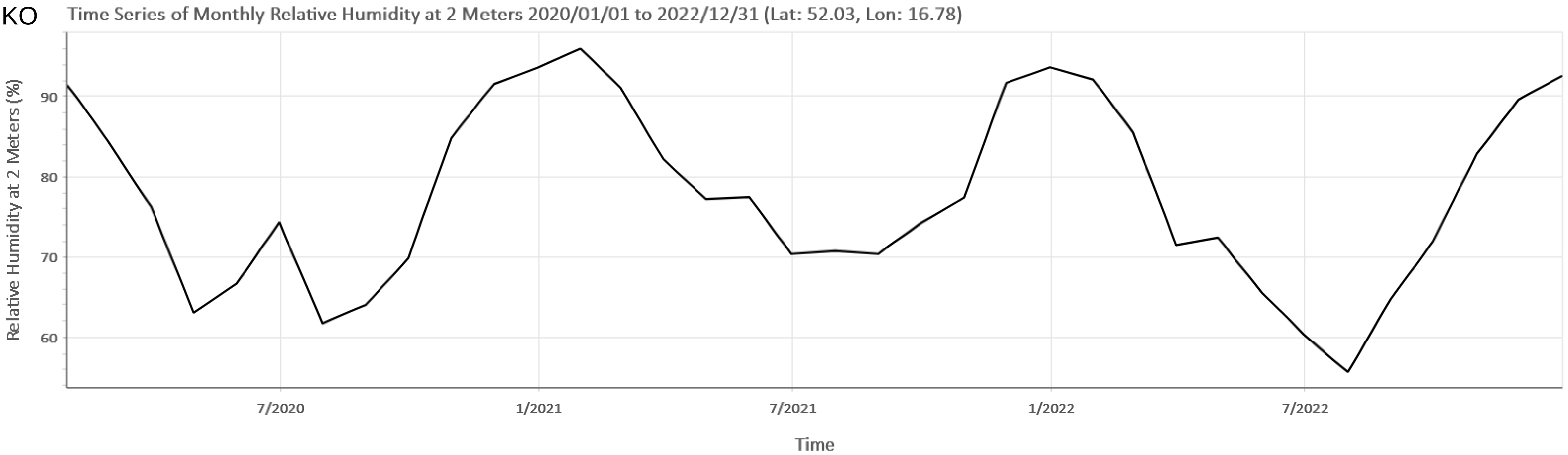
Time Series of Monthly Relative Humidity at 2 Meters 2020/01/01 to 2022/12/31 (Lat: 52.03, Lon: 16.78)
Relative Humidity at 2 Meters (%)
90
80
70
60
7/2020
1/2021
7/2021
1/2022
7/2022
Time

PHR

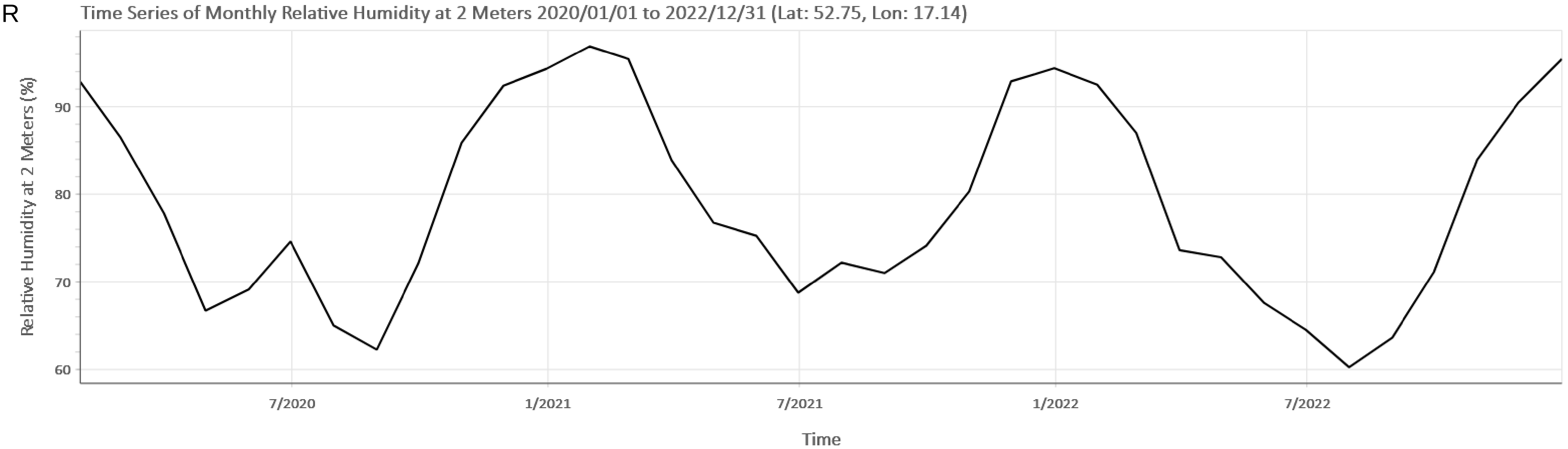
Time Series of Monthly Relative Humidity at 2 Meters 2020/01/01 to 2022/12/31 (Lat: 52.75, Lon: 17.14)
Relative Humidity at 2 Meters (%)
90
80
70
60
7/2020
1/2021
7/2021
1/2022
7/2022
Time

DANKO

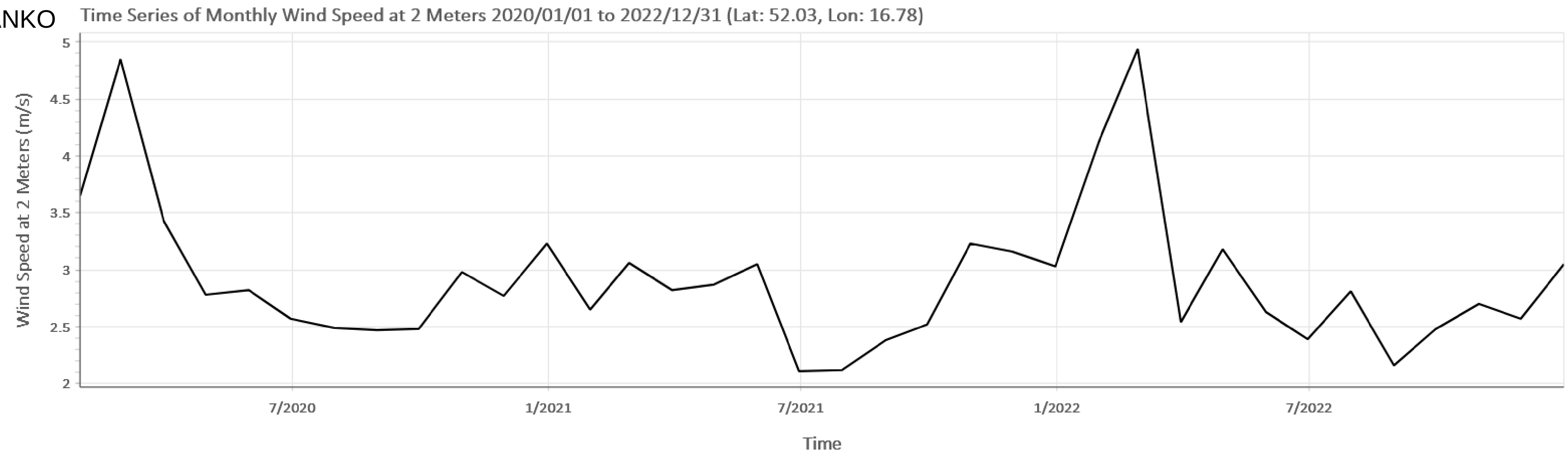
Time Series of Monthly Wind Speed at 2 Meters 2020/01/01 to 2022/12/31 (Lat: 52.03, Lon: 16.78)
Wind Speed at 2 Meters (m/s)
5
4.5
4
3.5
3
2.5
2
7/2020
1/2021
7/2021
1/2022
7/2022
Time

PHR

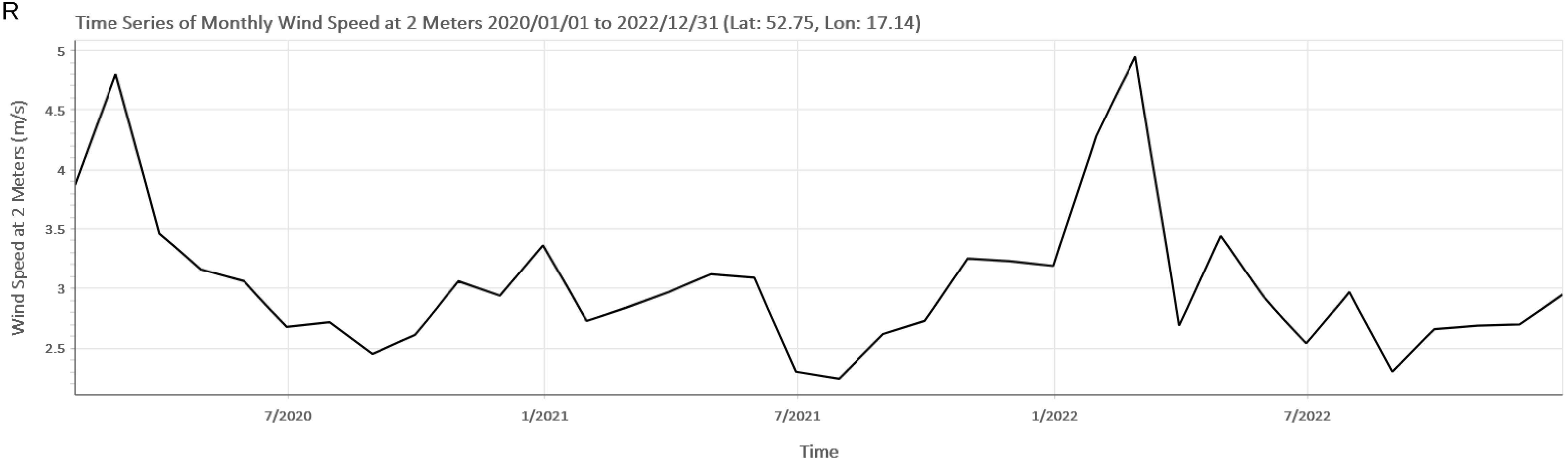
Time Series of Monthly Wind Speed at 2 Meters 2020/01/01 to 2022/12/31 (Lat: 52.75, Lon: 17.14)
Wind Speed at 2 Meters (m/s)
5
4.5
4
3.5
3
2.5
7/2020
1/2021
7/2021
1/2022
7/2022
Time

DANKO

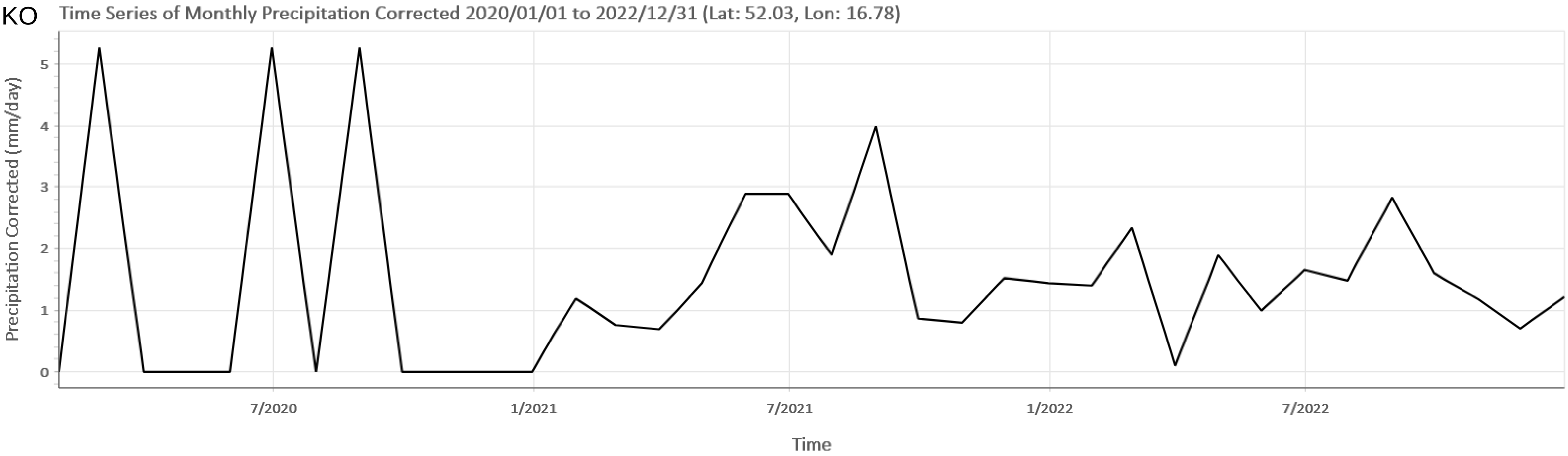
Time Series of Monthly Precipitation Corrected 2020/01/01 to 2022/12/31 (Lat: 52.03, Lon: 16.78)
Precipitation Corrected (mm/day)
0
1
2
3
4
5
7/2020
1/2021
7/2021
1/2022
7/2022
Time

PHR

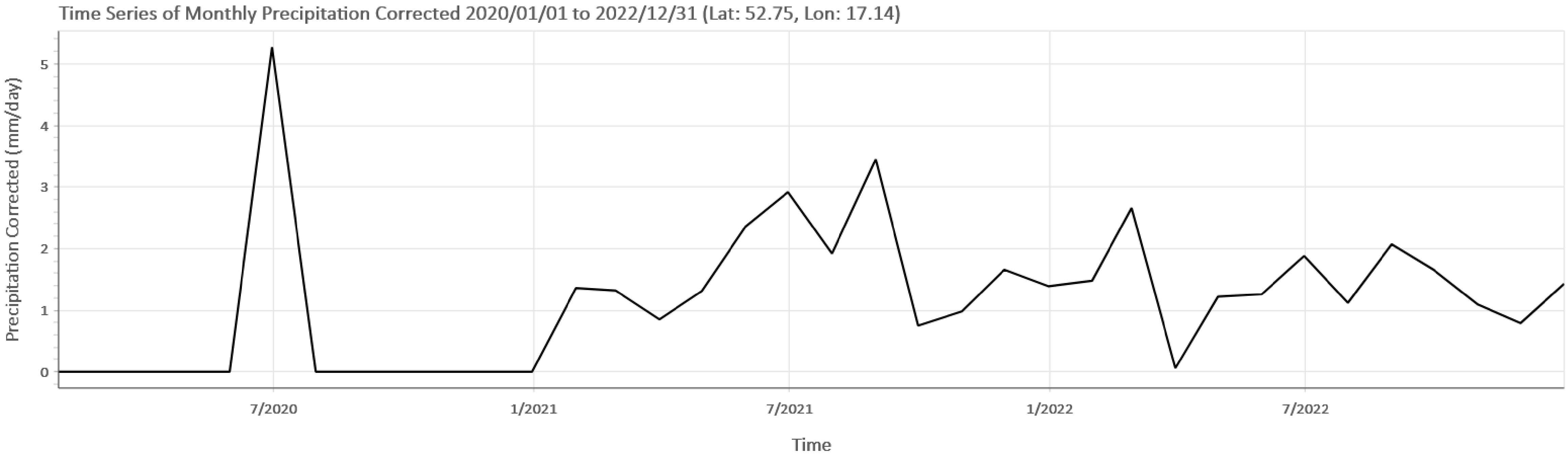
Time Series of Monthly Precipitation Corrected 2020/01/01 to 2022/12/31 (Lat: 52.75, Lon: 17.14)
Precipitation Corrected (mm/day)
0
1
2
3
4
5
7/2020
1/2021
7/2021
1/2022
7/2022
Time
