# Supplementary material for: Searching for genes determining the APR phenotype in rye
Source: BMC Plant Biol. 2025 Jul 19;25:935. doi: 10.1186/s12870-025-06920-0 (PMC12275401; doi:10.1186/s12870-025-06920-0)
Supplement: Supplementary file 2 — Supplementary Material 2. [file 12870_2025_6920_MOESM2_ESM.docx]

Supplementary figure 2. **Primers for amplicon sequencing of different ScLr_SUG genes.**


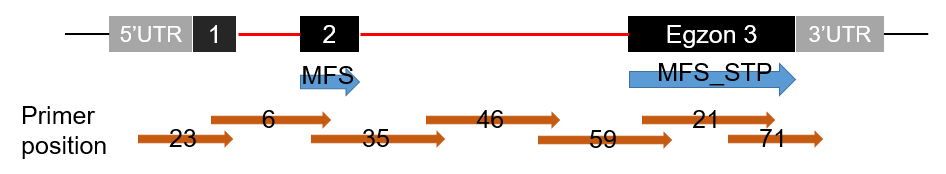


**ScLr_SUG1 (ID: SECCE7Rv1G0472490.1) DNA sequence**

ACCTTTCAGAAGGTGACAACCGCTTTGCGTATGTTGGCATACGGTATCCCGGCTGATCTAGTTGATGATCACTTGGCCATGGGTGAAATCCAAGCCATCATGTGTGTTAAGCGCTTCGCAGTCGGAATTGTGTAAGTGTTTGGCCAGGAGTACTTGGAGAATCCCAATGCTGAAGACGCCGCAAGGCTACTGAAGATGAACAAAGCTAGCGGCTTCCCAGGTATGCTTAGCTCAATAGATTGCATGCACTGAAGTTGGAAGAATTGTCCAAAGGCATGGCATGATGATCTTCAGAATGATCTCATTGAGGAGTGATGGGTTTGGAAAGACCGCCAAAGAGCATCATGATTTGTGCGTTTGATGTTGTATTGTTGAACTATTTATTATATTTGAAAGATAAACTATTTATTTGAGTTGTAATAACGGAATTAAACTATTTTATTGTTGATTATTTTGTTTGTGTTTGATCTTCTTGCTTATGTTTGGAGCGCATATGTTGTTTGTGCGAGAGCGCGCGCTGCTGGAGTTAGCGCTACCCGCCGCGTCAAACCAAACGATGGACGCGCTGCAAACTGATTTTTAGCGCGTCGCGCGTTGGCCGGCTGTTAAAGATGCTATAAGCAGTTTGCCGGTGGGGGGAGAGACAGCGTGTTTACGTTATCAGTTAAGTACTGTACCTCAGAGTCAAAGCCCAGCCAGGACCGGACGTGTCTGGGAGTAGTAGTTTCGGGCTTTGCCCGACGGCGACCGGCCGTTGGTTCAGACACAGCGAAACGCAAATCAAACCCAGAGATCGTTAGGGAGTTCCCCACGGAAAACGGTGCTCTTTTTTCAGAGATCATACGGAAAACCGGTGCTGTGCGTACGTGAGACATCCATGCGTGGCGTTGGCTGCGTACTCGCAGCATATTTTACTACGGTATATAAGCATACATGGAGTGAAAAGGATCCTCACGTAACACGTACGTTGTCGTCGTGGCACTCTGTGCTGCGCACAAGTGCTGCTCTCGTTCGTAAGCTCGCTGAAGGAAGTTCCGCAGAAGTCTTGGCCTGCCCTGGACAAACGCTCCAGCTCGCTCGTGTTCCTTTCTGCTTTTGCGAGCACGTCGCGGTCGGCCATCGGGTTGAACCAAGACGGCAAATTGCGGCGTCGCACGCCGTTTAAAATTCCCAAACGGGTGGGAGCTCTCGCGTGCAAACCGTGCCGAATCATGCATTTGAAATTCTTTTCAGCATAGGTGTATTTTCTTTTCGAGGGAACCATCGATTCCCCTGACGGCATTATTCGGGGCCGCGGCTGCAGGGTTTTTGGGGTGTTGAATATTCAGGCGATAAGATGAGTTGACTTATACTTATACTACCTCCTTTCCGGTTTATAAGGCTTGCGCGTATTCCTAGGTCTACAATTTGACCAACATAAAATGAGTTGTGTAGTCTAAAAATTACATCATTAAAAAGTAGAAGATATGAAGTTTCTAATGATATATTTTGTGTGACACATAACTTGTATTACGTTGGTTTATTTGTCAACCTAGATATACGCGCAAGCCTTATAAACCGAAAAGGAGGTAGTAGCATGACAGTATGAACTGAGCATCGTCGGTCGGTGAGAACGTGCAAACGGGCTTGGGTTGCCGGATACATAAAGATCAAAGCAAAATTCGATTTCGCGCAGATTTGTTATTTGGAGTGGCGACCGGTCGTTAGCCCACATGCTCTAATTCCACGGCTTGGTTCGTTTCCCGTGAGCCTAACGTGTATCATTAGGCGCGTTGTTTAGTACTCCTGGCTGCGGCGAGATCGAACTGATTACAGAAATAATGGAGAAATTTAGAGTACCACTGATTTGTAACGACTAACGATACAATATGTTGTGTCATCAGGTCGACCAGGCTAGCTGTAAGTGGGATTTTATTTAGCCCGCTCTTCGTCGACATCGTGACAGTGGTATATTTATTTATACAGCCATATGTTGTACGATCGTATCTCGACCAAATCGCTAATGGTACAAAAGCTAGACGTACATAAACTAGCACCACTTGGCCAACACCACTTTCCCCTGTGGAAAAGTACACGCACGCATGCACTGTGGTAATCGCCGGGGAACGCCGACCGGGAGTCAAATTTCTTGTGCTTGCATGTATACTGCTATCCACATCGACACATAATTTTTCCCAAGCGAATCCATCCCCACGTGACGTATAAATTTCGTTAAAATTGTATTCAAAGTCAAGGTCCTCCATTCACATGAACCAAGCAAGCAACAAGGAAGCTGCGCCCGTTGTTGACACTCCGAAAATTCAGAAGGATTGTTCACTGACGTGTGGGCCCATCCAGCGATCTCATTGTCAAGAAGGTCGGCGTCCGATAAAAGAAATATCCTCAAGGTTTTCCGAACCACTCTCCAGTTTTCCAAAAAAGGAGCCCATAAAGGCTTACTACAAGAAGGGTTTGGTTTGGTTTGGATAAGCCATAATTTGTGGGAGGGACGGAAAAGTCGTCGTGCTTATGCACACAGTGGTGCGTGGTCTGGCCCGTGGGCCGTGGAGCCGTCCGTTGACTGGGCAGCCAGCGAGCTGCGGGCGAGAGAGCCGTCGTGCGGGTCCCACCGGAGGCGACGTGGCCGCATCTGGCCGCTCGTTTGCATTTGAAATTTCTTAGTCGAGTGGGCTTCCCTCCCTATATATACCCAGGGACTTGGAGCCTTCTGTCCCATCTCCAATCCCCTTCCTTTGGCTGCTCCCTCGTGTGGCTCTGGAGAAACACTCGCTGCTTGTCTAGCTTCCATTATATCGGCGTAGCTTGACCGGCCGGCCTGCGAAG**ATG**CCGGGCGGAGGGTTCGCCGTGTCGGCGCCGTCCGGCGTGGAGTTCGAGGCCAAGATCACGCCCATCGTCATCATCTCCTGCATCATGGCGGCCACCGGCGGCCTGATGTTCGGCTACGACGTCGGCATCTCAGGTAACCCGGCCGATACTCTACATGTCCATGCAGTTCTTCCTGCCATTGATCATCTCCATTCATGCATGTGCGTGCGTGCGTTGCGTACGTGCTCCCGTGGTAGCTCTGGGAAATCATGTCAAATCTGCCTTACATAGTTACATGCATGGCTTAGGGATCCGTAACGTCGCCGTCCGTGCGTATGTTCATCCGTGTGCTCGCCTCCCATGATCCCGTTGGAACCACACGCATGGGAGAGAGGAGTTTAATTGACTCCATGGATATGGATATGGTGTTTACTTGTGGCAGCCGTCTCTCGTCTTCATATGACGAAGCAGATAACCACAGATAATTGGAACGAAGTTAATGCAATTGATCCTTCTCATTGACTACCACGCACCGGCCAGCTGATGATTTAACTCTTACCTATTTAAGCCCATTGTGAACAATATCCAGTTTTCTGGCCAGGATCATATATACTCGGTGCTAGCTAGCTGTATGATTCTGTGACTGATACCCGGTACTACGTAAGAATTAATATTATACTGTATTGGGAAGAAAATAGATAACTGCTGCCATCTTGCCACCTACTTTGACAGGCTATTAAGTCAACCCATTTGTACACCTTCGGATCACGTTGGATTCAACAAAGCTGTCTTTTGTGTGGACACGTTGCCTTTTGGTTTAGCGTAGTATTAAATCTATTCTTACTACAACTTATTTTTGCGAGATGAGTTCTTACTAATACTAATTAATTAAATATTATCCATTTCTTTTACAGCGTCCAGCTTGATTAACAACCAGTTCTAAGGTCTAAAAACTGTTTGTTTCTGCAGGCGGAGTGACGTCGATGGACGATTTCCTGCGTGAGTTCTTCCCGGCGGTGCTGCAGCGGAAGAACCAGGACAAGGAGAGCAACTACTGCAAGTATGACAACCAGGGCCTCCAGCTCTTCACCTCGTCGCTCTACCTCGCCGGCCTCACCGCCACCTTCTTCGCCTCCTACACCACCCGCCGCCTCGGCCGCCGCCTCACCATGCTCATCGCCGGCGTCTTCTTCATCATCGGCGTCATCTTCAACGGGGCCGCCCAGAACCTCGCCATGCTCATCATCGGCAGGATCCTGCTTGGTTGCGGCGTCGGTTTCGCCAACCAGGTTAGCAAGAATTCCCAGCTTCAGAGTTATATAACTATATAAGTACATTTTTTAACCTAGTATAAGCAAGATTACGTACGTTTGCGCAGCGGCATTTGGGTCAAAAAAGTCTTGGTAGAAACCGAAGTCAAACATGTAAAGCTAGCTAGCGGTGGCTGGTTAGAGAAAATATGTAGTAGCACGCATGCACGAGACGTAATTTTCCGCGTCTACGAAAATGACGTAACCCGTGTGCAGTTTCTTCCTTGTTTGCTGGCAATCAGTCACCGTCCACGTCCACGGTAGTATCAAAGAATCTTTGGACGACCAAACTAAACTTACTGGTCCTACCATTGTTTACGTCCAGATTAATAATACCATAAATATAAAATATTGTTGTATTTTATACGTATTAGCTGCAGTACGTATGTAGGCACATCGTATCCCTCTTGTGTTTCCCGGAATTTAGGGGTCAAATGTACAAACCATGTGACGGTGCATGACTCGTGTTGGCGCACGAGGACACCTTCCATTGGTGGCATCGCCTCACCTCTCGCGATAAGCTTGTTAAGTTCCAAGATTCCGGTGCTGGCCGCGACTCCCAGGCTTAGAAATTACTGGACCAAATGGTAGGAGAAGCTTGGGCCAACCAACAGGTGATAGGTGTACGCTTTGCAGCTTATCTCCTTGGTATCTCTTGGTTTTACTAGTCTGCTGGTGTGGCTGCCCTCCTCAGTTCTCTCTAGTTGTCCCAAGTTGCTATCAACTTGGACTAGTTGTAGAATATACTATCACAGCGGCTTTGGATTCTTCTGTTTTTGGCTGTCGAAACTTCGATTTTAGTAGTCGCAAGCAGGCGCGGTTGTTCACGCGTAGGCCACACGGGTCGCAATATGAGGCGGGCCTAGCTAGCTGATCGAGTAGTTGCACATAAACTTAGAGGCTTGCACCAAAATTGTAACTTGGATGCTTGACTTGGAGGTGTGCTTCGATTGGATTCTTCTGGTTCCTTCCTTGTCGAACAGTGAAAAAAACGATGCGTCTATGGACTCTAGACCTCAGAGCGGGGAGCTTTCTCGATTGAAAATGCAAAGAATTTGTGCACCAGTGACGCGTTAGATTACAAAAAATCATGGATCAACGCACTGATTGAGCATTACTTTGCAAATTATGCACACACTAACGTACAAAAATAATAACATAACGTACTTGTGCATAAATATATATAAGGGAGTCTAGACTCAATTATACGAAGCAGGCAAGACAAACATGCATGTGTGCCAGAACTAGCTCGAGTAGGAGTAGATCATTAGTCCAACAGTTTTTTATTCAGTTAATTTATCCGCTTCTGATTAGACCCACTGATTAAGAGATTCCGATGGTGATAGGTTGGTGCGGTTCTGACACTTTATTTGTACGGTCACTGATTATTTTTCTTGACAGTAGGAAAGCATGCTAGATGATTTGTGTTAGGGTGTATATAGGTCTCAGTCGAGTGAGACTTAATCAAGTCTCAGTCAAATGATATACTAGTATATAAGAAGAAAAAAAATAATTTTTTTTACACATCTTCATGCAAGATCAAAAAAATATAGCATCCATTGAGACATAACGAAGTTTAAGTTAACTCAGATTTAGCAAAACTGTTTGTTGTTTTTTTTTTGTTAGTGTAGTCACATGCTAGTAATGTCTCAAGAAAAGGAAGAAAACCCGGAAGAGAACCTTCTTGGCCCAATGGGTCTCCTTAAGGCTATCTGCATTTGACGGCCCATTTCGTGATTTCAAAATAAAAATGTTGATTTCACATTTCTCAATTTCATCGTTTATTATTTACATTTTGCTGAAAAAAACCTTTATTATTGATATACATCTCTAACGAAACGAACTGTGGTTTACGCAGGCCGTTCCCCTGTTCCTGTCTGAGATCGCGCCGACGAGGATCCGCGGCGGGCTGAACATCCTGTTCCAGCTGAACGTGACCATCGGCATCCTGTTCGCGAACTTGGTGAACTCCGGCACCAGCAAGATCCACCCGTGGGGCTGGCGGCTGTCGCTGTCGCTGGCCGGCATCCCGGCGGCGATGCTCACCCTGGGCGCGCTCTTCGTCACCGACACCCCCAACAGCCTCATCGAGCGCGGCCACCTGGAGGAGGGCAAGGCGGTGCTGAAGCGGATCCGCGGCACCGACAACGTGGAGCCCGAGTTCAACGAGATCGTGGAGGCGAGCCGCATCGCGCAGGAGGTGAAGCACCCGTTCCGGAACCTGCTCCAGCGCCGCAACCGCCCGCAGCTGGTCATCGCCGTGCTCCTCCAGATCTTCCAGCAGTTCACCGGGATCAACGCCATCATGTTCTACGCCCCCGTGCTGTTCAACACGCTCGGGTTCAAGAGCGACGCGTCGCTCTACTCGGCGGTGATCACGGGCGCCGTCAACGTGCTGGCCACGCTGGTGTCGGTGTACGCCGTGGACCGCGCCGGGCGGCGGGCGCTGCTGCTGGAGGCCGGCGTGCAGATGTTCGTGTCGCAGGTGGTGATCGCCGTGGTGCTGGGCATCAAGGTGACGGACAAGTCGGACAACCTGGGCCACGGGTGGGCCATCCTGGTGGTGGTCATGGTGTGCACCTACGTCGCCTCCTTCGCATGGTCCTGGGGCCCGCTGGGGTGGCTCATCCCCAGCGAGACGTTCCCGCTGGAGACGCGGTCGGCCGGGCAGAGCGTGACGGTGTGCGTCAACCTACTCTTCACCTTCCTCATCGCGCAGGCCTTCCTCTCCATGCTCTGCCACCTCAAGTTCGCCATCTTCATCTTCTTCTCCGCGTGGGTGCTCGTCATGTCCGTCTTCGTGCTCTTCTTCCTCCCGGAGACCAAGAACGTGCCCATCGAGGAGATGACCGACAAGGTGTGGAAGCAGCACTGGTTCTGGAAGAGATACATGGACGACGACGACCACCACATCGCCAACGGCAAGAACGTCACCGTC**TAA**AAAGTGTTACTACCACGTAACGCTGCCTGCCTGCAGAGCTCGACTTAGAAAGAAGAAAGATGGATACTGAGCAGCTTTATCGATCGATCTCGACATCTCGTCTCGTCTCGTCTGTAACATATGCAGACACTTTCGCAGTCGCGAGCTCAACTATTACACTGCTGACATGCCAAGAGGGAACAGCAGGCAGTGCGGCAGTAAACCTTGCTGTTCTATACTTCTATATATAGGTAATAATAGCTAAGAGTGTTTTGGTGTTTCTTGTTCATTAGTATATCCTTTACGTGTCTATTTGTTTGGAATCGGTCCCTGACAATGCGAGCCTACCTGTATTATACAGCACCTTGTAAAACTGATGTTGCGATTCCTTAATGCACAGCAGTAATGTTCCACAGTCGCCTCTCTTTTAGTCAGCTATATTTCATCATCCGCCCTTCTAAAGTGTTAACAGGTTTTTGCCTACCGAACACTTGCTTGAAACGGAGGCAATTTTTTTTTGTCTCATCCATTAAATAAGTGAAAGGCTTCTTTGATCGAGCGGTGCTTCAGCTTACACATTGATGACAGTGGATCTCGGCGGCATTGGCGCAGTGGAGACTTGGCGTCCGATGCGCGGACATGGACTCGCGCAGGAGGAGGAGGCTGTCTGTCGTCATGGCGACGTCAATGGCAGAGAGGACTGGCAAGGTCGGTGCATCAGTTCTGCTCCGAGGATGGTTCGATGGAAGATGAAGGTGACGGCCCTTGCAGCGTGCGGTGCTCACTGAGAGTGTGCCGGACCGCTGTGTGACCCAATCCAGGTAGTGGCTTTGATGGGGCATCCGACTTTAGATGTTAGGCTTTGGTGCGATGTAATATTTGGTATTAGGCCCGGATATTCGGCACACCTTCATCAAGGGGATAGGAGTAGCAACAATGTTGCCAAGATGGTGGCTTCAGGCTTATTGATGTATCACTTTGTATGGTCTTTGGGAATAATTAATAAAATGGCTGCATGCATCGTCCAGATGTAGAGGCCAACCGTTACAAACCGTCGAGCAACACACAAAAAATACCCCCCACACACCTCAACAAGGCAGGGTCGCATCAAGATACATTAGGACGTCATCGTCAGCACTTCTCCCCAACATGCGTCATCGCCCTCCCTAGACTCCGAGCAAGCGACTTCGACCTCGCCAAAGACAAACCTCGAGCCTACCCTTCTCGTAGAGGCTATGTGGAAACACAGGCAGAACCGTGCCATGCCCAGCCTCCTGCACCGAAGGCGGCGCAACTCCGACTCTGATGGAGAGCTCTAAAGGAGAACTATGTGTCGTGGGAGTGAGACTGATAGTTAGAGTAGGGGGTACGTAAGAGGAGGCAAGGTCCTAGCTACGGTGAGGTTGTACACGCAAGTTTTACGAGTTCATGCCATTCTCGGAGGAGGTAACAACCCTACGTCTCGGCGCCTTGGAGGCTTGTCGACTGGATTATGCGTGAAAGTTACAGGGGGTGCGAACCCCAGTGCCAGAGGAGGGGGTGGCTGTAACGACCAAACCTCGAAGGATTTGAGTCTCTGTGCTTACCGTGCTAGTCCCTGGATCGAACTCGCTAGCACACACAGTATAGATGAATACCAGAATACAAGTGCATCATTTATTACAACGTATGATCCAGAGTATATAAAATAAAACAATCCGCATGGCTCATGGCTAGCTATTATTCAATCACACAACGGAAAATAGCAGAAAATAACGATGAGTCCCATCATAGCCCAACTGGCGATGCTGAGTGAAGACTCGGGACCCTAACGGTACCTTACTCCTCGTCTGAAAATCCTGCAACATGAGACGTTGCAGCCGTATAGGTCAATACATTGAATGTATTGGCAAGTTACACAGGAGAAGTAATAAAGCAGACCTACATGTATATGCATAGAACAACAAAGGAAGGCTTGAGGGTTTACTTTGCAGAAAGCTGATTTTGTCCTCATAACTACCTAACAAATAGTCAATTTTTCATTTAGTTCTTTTATTATTACAATTGAATGCACAAGGTTGAGTTGGCAAAATCAACTCCAACCTACCCATTTATTAAGTTGCCCACAAATATTATTAAGTATCACATCTGTCAAGATCACCCAACAACTGTAATGGCATAGTCGCTGAAATTTACCCATAATCGGGGGCACGGCTAATCATGATTAGTTTTATTACTCTGCAGAGTTTTGCACACTTTACCCACTAGACTCAACCCGAAGATTGAGACGAAGCCTTTCAGAAGTAGTCACCTCCACCCCCGGCAGCCGGTACACCTATCCATATCTACATCTGCTAGCATACCTGGGCGAGGATCCCACAACCTACTCAACTAAGCCAGAGCCCATTTAGCTAGTGGTCGCGCATGGAAGCTACTAGTCATTAAGTCTGTCTGATCTTTTTGAGCCTGGGCGGCCGTCCACTTACATTCAGAGGGTATAAACCATGATGTTCTTAAGACCACCCATCAATACCACTTCCGCCCAGAGGTGAGTTAAGTTCTGAATTACTACTCAGTTTGTTATATATTAATAATCCTCACATAAGCTCAATGTATACAGGAACCAACCCGTCTACAAGCATAGCAAAACTATACCTACCACGATTTGCAAAAGACGGGAGATAGTTCAACAATATAGCTCAAATTATAACAATCCTATAATGCATAAGTTTATAGTGTGGCATAACGTACAATGCATAGAAAAACAATAGGTAAAGGATGATCAACATGTAACTTGCCTTGGTTCTGGTTCAAAAAGTCACACTCCTGACAATAGCTCGCGTCGCACTCCGGACAATCTACACGTTTCACACAATATAACAAATCAATCACCGAACTCAAATAAAACCAAAATAAAGCCAACAGCAAGAAAACCATTTTAAAACTCAAAAACAGCAGCAAAACAACATGCACAGATCATCTATATTGCACTACGGTCACAATGCAAAAAGAATCGCTTGATTCCGATAAACGGTTTAAAAGATACGGCCTCCGGAAGGTATAATTCAAATTCAAATGAATTCAAATTTGTATTGTGTAAACATGTGAAAAATTGGT

Legend:

black – UTR

yellow – CDS

red – Intons

underline – primers

**Primer list:**

| **Name** | **Sequence** |
| --- | --- |
| ScLr_SUG1_Fwd_23 | GTCGACATCGTGACAGTGGT |
| ScLr_SUG1_Rev_23 | GGGTTACCTGAGATGCCGAC |
| ScLr_SUG1_Fwd_6 | CACGCCCATCGTCATCATCT |
| ScLr_SUG1_Rev_6 | TTGTCCTGGTTCTTCCGCTG |
| ScLr_SUG1_Fwd_35 | ACTGTTTGTTTCTGCAGGCGG |
| ScLr_SUG1_Rev_35 | CCAGCACCGGAATCTTGGAA |
| ScLr_SUG1_Fwd_46 | GGCACATCGTATCCCTCTTGT |
| ScLr_SUG1_Rev_46 | AAAGTGTCAGAACCGCACCA |
| ScLr_SUG1_Fwd_59 | CGAAGCAGGCAAGACAAACA |
| ScLr_SUG1_Rev_59 | GATCTCGTTGAACTCGGGCT |
| ScLr_SUG1_Fwd_21 | CTGAACGTGACCATCGGCAT |
| ScLr_SUG1_Rev_21 | CAGAGCATGGAGAGGAAGGC |
| ScLr_SUG1_Fwd_71 | GGACAAGTCGGACAACCTGG |
| ScLr_SUG1_Rev_71 | CACCGCTCGATCAAAGAAGC |

**ScLr_SUG4 (SECCE2Rv1G0088070.1) coding sequence**

ATGGCCGGCGGCGCGGTTGTTAACACCGGAGGGGGGAAGGACTACCCCGGCAAGCTCACCATGTTCGTGCTCTTCGCCTGCATCGTCGCCGCCACCGGCGGCCTCATCTTCGGATACGACATAGGCATCTCCGGTGGCGTGACCTCGATGAACCCCTTCTTGATGAAGTTCTTCCCGGGGGTGTACCACCAGGAGCAGGAGGCGGAGCGGAACCAGAGCAACCAGTACTGCAAGTTCGACAGCCAGCTGCTCACCATGTTCACCTCCTCGCTCTACCTCGCCGCGCTCGTCGCTTCCTTCTTCGCCGCCACCGTCACCCGCGTCGCCGGCCGCAAGTGGTCCATGTTCGCCGGCGGGGTCACCTTCCTCGTCGGCGCCGCGCTCAACGGCGCCGCCAAGAACGTCCTCATGCTCATCCTCGGGCGCGTCCTGCTCGGCATCGGGGTCGGCTTCGCCAACCAGTCGGTGCCGGTTTACCTGTCGGAGATGGCGCCGGCGAGGCTGAGGGGGATGCTCAACATCGGGTTCCAGCTGATGGTCACGATCGGCATCCTGTGCGCGAACCTGATCAACTACGGGACGGCTAAGATCAAGGGCGGGTGGGGCTGGCGCGTGAGCCTCGCGCTGGCCGCGGTGCCGGCCGGAATCATCGCCATCGGCGCGCTCTTCCTCCCCGACACCCCCAACTCCCTCATCGACCGCGGCTACACCGACGACGCCAAGAAGATGCTCCGGCGCGTGCGCGGCACGGACGACGTCGAGGAGGAGTACAGCGACCTGGTGGCCGCCAGCGAGGAGTCCAAGCTCGTGAGCCATCCGTGGCGCAACATCCTCCAGCGCCGGTACCGGCCGCAGCTCACCTTCGCGATCGCCATCCCCTTCTTCCAGCAGCTCACCGGCATCAACGTCATCATGTTCTACGCGCCCGTGCTCTTCAAGACGCTCGGGTTCGCGGACGACGCGTCCCTCATGTCCGCCGTCATCACCGGCCTCGTCAACGTCTTCGCCACCTTCGTGTCCATCGTGACCGTGGACCGGCTGGGCCGGCGCAAGCTCTTCCTGCAGGGCGGCACCCAGATGCTGGCCTGCCAGATCGTGGTGGGCAGCCTGATCGGCGCCAAGTTCGGGTTCACCGGCGTGGCCGACATCCCCAAGGGGTACGCGGCGTTCGTGGTGCTCTTCATCTGCGCGTACGTGGCCGGGTTCGCGTGGTCCTGGGGCCCGCTGGGGTGGCTCGTCCCCAGCGAGATCTTCCCGCTGGAGATCAGGTCGGCGGGGCAGAGCATCACCGTGTCCATGAACATGCTGTGCACCTTCATCATCGCGCAGGCGTTTCTCCCCATGCTCTGCCGCTTGAAGTTCATGCTCTTCTTCTTCTTCGGCGCGTGGGTGATCGTCATGACCCTCTTCGTTGCCTTCTTCCTGCCGGAGACCAAGAACGTGCCCATCGAGGAGATGGTGCTCGTGTGGAAGGCGCACTGGTACTGGAGCCGCTTCATCCGCGACGAGGACGTGCACGTCGGCGGCGGCGCAGACGTCGAGATGCGCTCCAACGGCAAGGTCCAGGCTGCCAAGCTCCCGTGA

| **Name** | **Sequence** |
| --- | --- |
| ScLr_SUG4_Fwd | CACCATGTTCGTGCTCTTCG |
| ScLr_SUG4_Rev | TTCCACACGAGCACCATCTC |
| ScLr_SUG4_Fwd2 | ATGTTCGTGCTCTTCGCCTG |
| ScLr_SUG4_Rev2 | AGAAGGCAACGAAGAGGGTC |

**ScLr_SUG6 (SECCE2Rv1G0097870.1) coding sequence**
ATGGCGGGGGGCGCCGTCGTCAACACGTCCGGCGGCAAGGACTACCCCGGCAGACTCACCCTCTTCGTCTTCTTCACCTGCGTCGTCGCCGCCACCGGCGGCCTCATCTTCGGATACGATATCGGCATCTCAGGGGGCGTGACGTCCATGAACCCTTTCCTGAAGAAGTTCTTCCCGGAGGTGTACCAGAAGAAGCAGATGGAGAGCTCGGCCAACCAGTACTGCAAGTACGACAACCAGCTGCTCCAGACCTTCACCTCTTCCCTCTACCTCGCGGCGCTTGTCTCCTCCTTTTTCGCCGCCACCGTCACACGCGTCGTGGGCCGCAAGTGGTCCATGTTCACCGGAGGGCTCACCTTCCTCATCGGCGCCGCGCTCAACGGGGCGGCGGAGAACATCGCCATGCTCATCGTCGGACGCATCCTCCTCGGTGTCGGCGTCGGCTTCGCCAATCAGTCTGTGCCGGTGTACCTGTCGGAGATGGCGCCCGCGCGTCTCCGGGGCATGCTCAACATCGGGTTCCAGCTCATGATCACCATCGGCATCCTGGCTGCGGCGCTCATCAACTACGGCACCAACAAGATCAAGGCCGGGTACGGGTGGCGCATCAGCCTGGCCCTGGCGGCCGTCCCCGCGGGCATCATCACCCTCGGCTCCCTCTTCCTCCCCGACACCCCCAACTCCCTCATCGAGCGTGGCCACCCGGAGGCGGCGCGCCGCATGCTCAACCGCATCCGCGGCAACGACGTGGACATCAGCGAGGAGTACGCGGACCTGGTGGTGGCGAGCGAGGAGTCCAAGCTGGTGCAGCACCCGTGGCGCAACATCCTGCAGCGCAAGTACCGGCCACAGCTGACCATGGCGATCATGATCCCCTTCTTCCAGCAGCTGACGGGCATCAACGTCATCATGTTCTACGCGCCGGTGCTGTTCGAGACGCTGGGGTTCAAGGGCGACGCGTCGCTCATGTCGGCCGTCATCACGGGCCTGGTCAACGTGTTCGCGACGCTGGTGTCCGTGTTCACCGTGGACCGGCTGGGCCGGCGGAAGCTGTTCCTGCAGGGCGGCACGCAGATGCTGATGAGCCAGCTGGTGGTGGGCACCCTGATCGCGGTCAAGTTCGGGACGAGCGGCGTGGGGGAGATGCCCAAGGGGTACGCGGCGGCGGTGGTGCTCTTCATCTGCCTGTACGTGGCCGGGTTCGCCTGGTCGTGGGGGCCCCTGGGGTGGCTGGTGCCCAGCGAGATCTTCCCGCTGGAGATCAGGCCGGCCGGGCAGAGCATCAACGTGTCGGTGAACATGCTCTTCACCTTCGTCATCGCGCAGGCGTTCCTCACCATGCTGTGCCACATGAAGTTCGGCCTCTTCTACTTCTTCGCCGGCTGGGTGGTGATCATGACCGTCTTCATCGCGCTCTTCCTGCCCGAGACCAAGAACGTGCCCATCGAAGAGATGGTGCTCGTCTGGAAGGGGCACTGGTTCTGGCGCAGGTACATCGGAGACGCTGACGTCCACGTCGGCGCCAACAACGGCAAGGGCGCCGCCATTGCATAG

| **Name** | **Sequence** |
| --- | --- |
| ScLr_SUG6_Fwd | GCAGACTCACCCTCTTCGTC |
| ScLr_SUG6_Rev | CTCTTCGATGGGCACGTTCT |
| ScLr_SUG6_Fwd2 | TCTTCGTCTTCTTCACCTGCG |
| ScLr_SUG6_Rev2 | ATGGGCACGTTCTTGGTCTC |
