# Supplementary material for: Searching for genes determining the APR phenotype in rye
Source: BMC Plant Biol. 2025 Jul 19;25:935. doi: 10.1186/s12870-025-06920-0 (PMC12275401; doi:10.1186/s12870-025-06920-0)
Supplement: Supplementary file 3 — Supplementary Material 3. [file 12870_2025_6920_MOESM3_ESM.pdf]

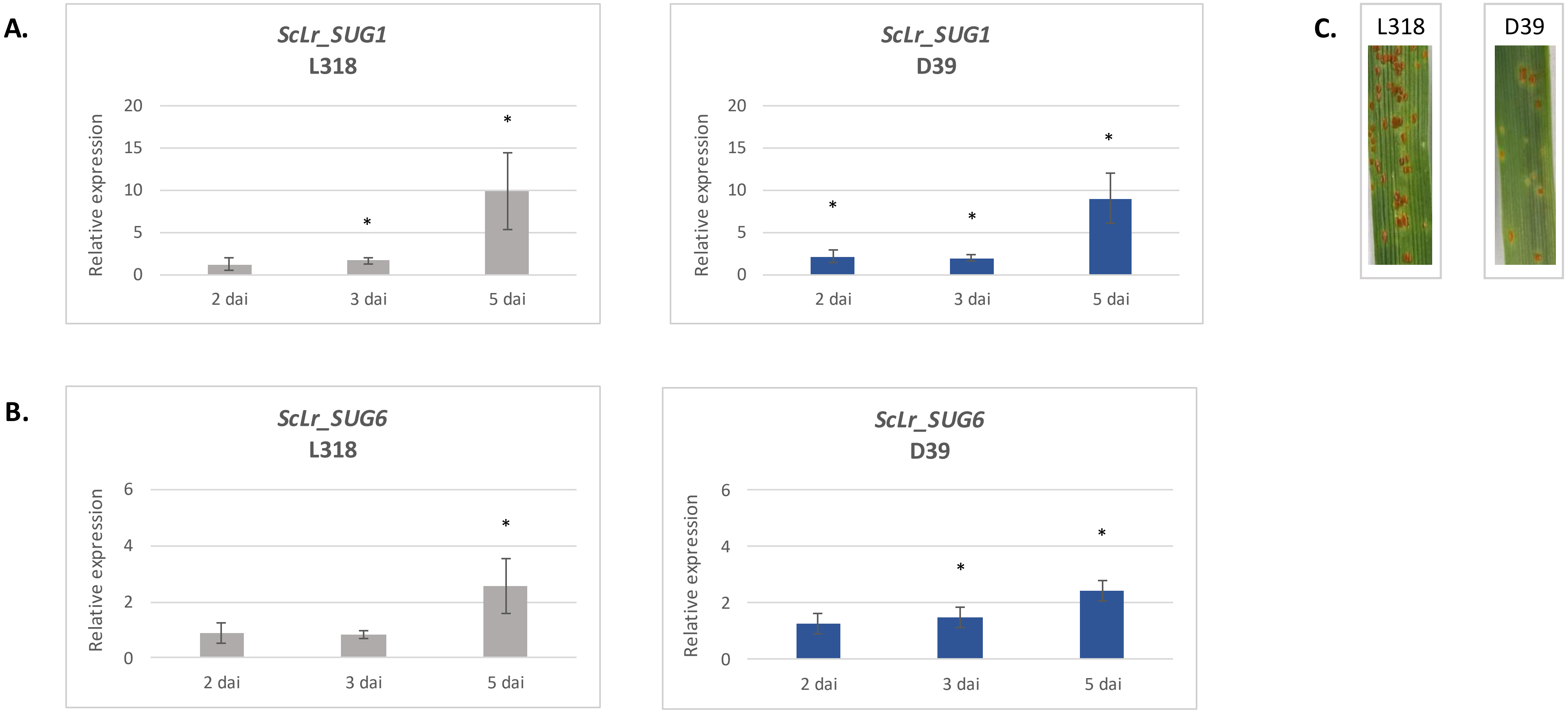

Fig. S3. Relative expression of *ScLr_SUG1* **(A.)** and *ScLr_SUG6* **(B.)** genes in two rye inbreed lines, L318 and D39, 2, 3 and 5 days upon *Puccinia recondita* f. sp. *secalis* infection (isolates from Polish Breeding company – Danko Plant Breeding). **C.** Leaf rust symptoms on L318 and D39 rye inbreed lines 10 dai.
