# Supplementary material for: Searching for genes determining the APR phenotype in rye
Source: BMC Plant Biol. 2025 Jul 19;25:935. doi: 10.1186/s12870-025-06920-0 (PMC12275401; doi:10.1186/s12870-025-06920-0)

Supplementary Figure S4. **Distribution of increase disease severity in rye inbred line leaves.** Calculation was made after phenotypic assessments of LR intensity in A. DANKO 2020, B. DANKO 2021, C. PHR 2020, D. PHR 2021. F, F-1, F-2 denotes a flag leaf and leaves underneath.


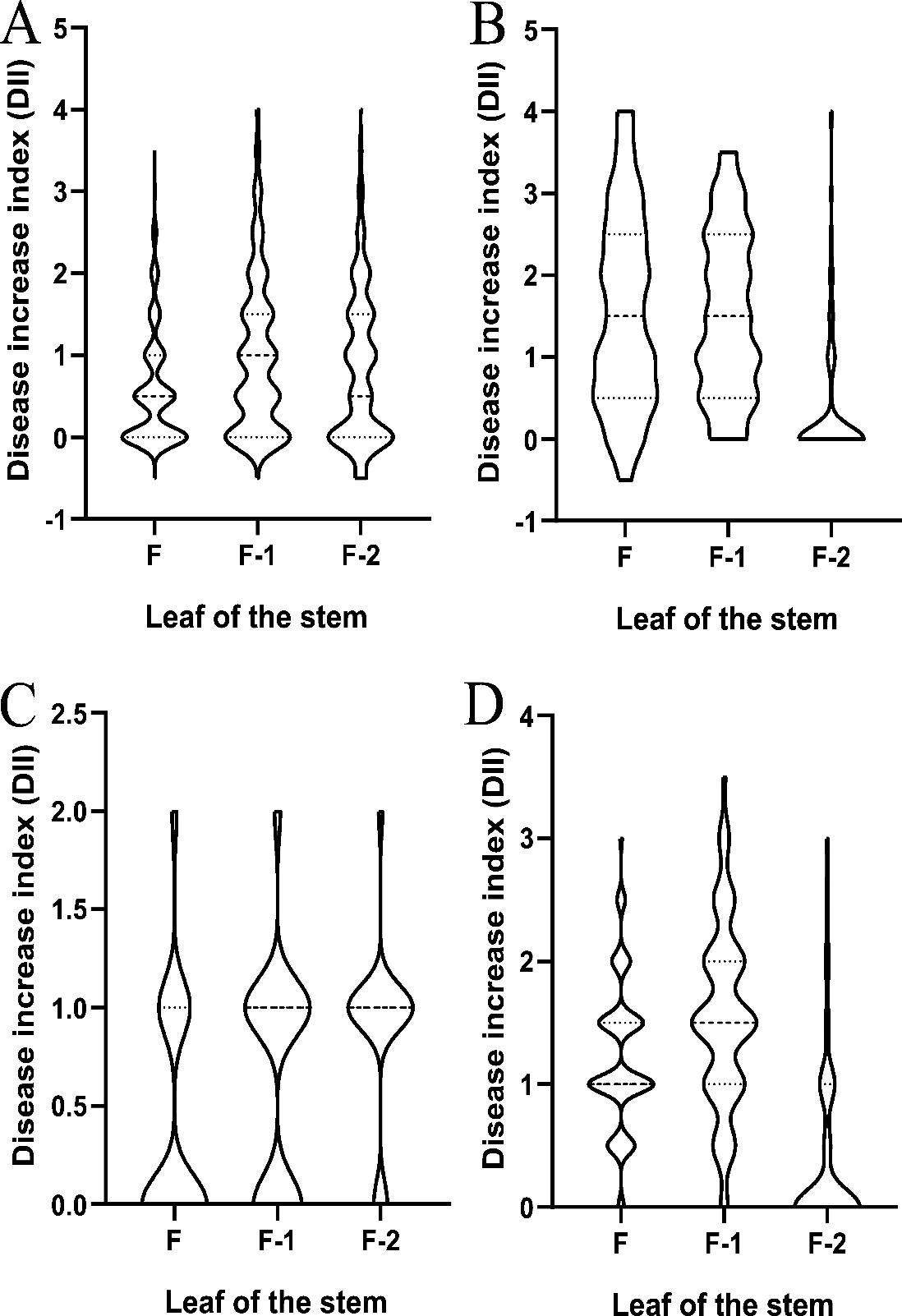

Supplement: Supplementary file 4 — Supplementary Material 4. [file 12870_2025_6920_MOESM4_ESM.docx]
