# Supplementary material for: Searching for genes determining the APR phenotype in rye
Source: BMC Plant Biol. 2025 Jul 19;25:935. doi: 10.1186/s12870-025-06920-0 (PMC12275401; doi:10.1186/s12870-025-06920-0)
Supplement: Supplementary file 5 — Supplementary Material 5. [file 12870_2025_6920_MOESM5_ESM.pdf]

```
Consensus           MPGGGFAVSAPSGVEFEAKITPIVIISCIMAATGGLMFG--------YDVGI-SGGVTSMDDFLREFFPA
                             10        20        30        40        50        60        70
Lr67(sus)           MPGGGFAVSAPSGVEFEAKITPIVIISCIMAATGGLMFG--------YDVGI-SGGVTSMDDFLREFFPA  61
Lr67(res)           MPGGGFAVSAPSGVEFEAKITPIVIISCIMAATGGLMFG--------YDVGI-SGGVTSMDDFLREFFPA  61
ScLr67-1(Lo7)       MPGGGFAVSAPSGVEFEAKITPIVIISCIMAATGGLMFG--------YDVGI-SGGVTSMDDFLREFFPA  61
118_DANKO_APR       MPGGGFAVSAPSGVEFEAKITPIVIISCIMAATGGLMFG--------YDVGI-SGGVTSMDDFLREFFPA  61
119_DANKO_APR       MPGGGFAVSAPSGVEFEAKITPIVIISCIMAATGGLMFG--------YDVGI-SGGVTSMDDFLREFFPA  61
119_DANKO_APR2      MPGGGFAVSAPSGVEFEAKITPIVIISCIMAATGGLMFG--------YDVGI-SGGVTSMDDFLREFFPA  61
120_DANKO_APR       MPGGGFAVSAPSGVEFEAKITPIVIISCIMAATGGLMFG--------YDVGI-SGGVTSMDDFLREFFPA  61
123_DANKO_non-APR   MPGGGFAVSAPSGVEFEAKITPIVIISCIMAATGGLMFG--------YDVGI-SGGVTSMDDFLREFFPA  61
138_DANKO_APR       MPGGGFAVSAPSGVEFEAKITPIVIISCIMAATGGLMFG--------YDVGI-SGGVTSMDDFLREFFPA  61
153_DANKO_APR       MPGGGFAVSAPSGVEFEAKITPIVIISCIMAATGGLMFG--------YDVGI-SGGVTSMDDFLREFFPA  61
157_DANKO_APR       MPGGGFAVSAPSGVEFEAKITPIVIISCIMAATGGLMFG--------YDVGI-SGGVTSMDDFLREFFPA  61
160_DANKO_APR       MPGGGFAVSAPSGVEFEAKITPIVIISCIMAATGGLMFG--------YDVGI-SGGVTSMDDFLREFFPA  61
71_PHR_APR          MPGGGFAVSAPSGVEFEAKITPIVIISCIMAATGGLMFG--------YDVGI-SGGVTSMDDFLREFFPA  61
149_PHR_APR         MPGGGFAVSAPSGVEFEAKITPIVIISCIMAATGGLMFG--------YDVGI-SGGVTSMDDFLREFFPA  61
59_DANKO_non-APR    MPGGGFAVSAPSGVEFEAKITPIVIISCIMAATGGLMFG--------YDVGI-SGGVTSMDDFLREFFPA  61
61_DANKO_non-APR    MPGGGFAVSAPSGVEFEAKITPIVIISCIMAATGGLMFG--------YDVGI-SGGVTSMDDFLREFFPA  61
129_DANKO_non-APR   MPGGGFAVSAPSGVEFEAKITPIVIISCIMAATGGLMFG--------YDVGI-SGGVTSMDDFLREFFPA  61
150_PHR_non-APR     MPGGGFAVSAPSGVEFEAKITPIVIISCIRHLSIWKKIDNCCHLATYFDRLL-S-GVTSMDDFLREFFPA  68
150_PHR_non-APR2    MPGGGFAVSAPSGVEFEAKITPIVIISCIRHLSFLARII--------YTRSCASCGVTSMDDFLREFFPA  62
37_PHR_non-APR      MPGGGFAVSAPSGVEFEAKITPIVIISCIMAATGGLMFG--------YDVGI-SGGVTSMDDFLREFFPA  61
52_PHR_non-APR      MPGGGFAVSAPSGVEFEAKITPIVIISCIMAATGGLMFG--------YDVGI-SGGVTSMDDFLREFFPA  61
88_PHR_non-APR      MPGGGFAVSAPSGVEFEAKITPIVIISCIMAATGGLMFG--------YDVGI-SGGVTSMDDFLREFFPA  61
101_PHR_non-APR     MPGGGFAVSAPSGVEFEAKITPIVIISCIMAATGGLMFG--------YDVGI-SGGVTSMDDFLREFFPA  61
105_PHR_non-APR     MPGGGFAVSAPSGVEFEAKITPIVIISCIMAATGGLMFG--------YDVGI-SGGVTSMDDFLREFFPA  61

Consensus           VLQRKNQDKESNYCKYDNQGLQLFTSSLYLAGLTATFFASYTTRRLGRRLTMLIAGVFFIIGVIFNGAAQ
                             80        90        100       110       120       130       140
Lr67(sus)           VLRRKNQDKESNYCKYDNQGLQLFTSSLYLAGLTATFFASYTTRRLGRRLTMLIAGVFFIIGVIFNGAAQ  131
Lr67(res)           VLRRKNQDKESNYCKYDNQGLQLFTSSLYLAGLTATFFASYTTRRLGRRLTMLIAGVFFIIGVIFNGAAQ  131
ScLr67-1(Lo7)       VLQRKNQDKESNYCKYDNQGLQLFTSSLYLAGLTATFFASYTTRRLGRRLTMLIAGVFFIIGVIFNGAAQ  131
118_DANKO_APR       VLQRKNQDKESNYCKYDNQGLQLFTSSLYLAGLTATFFASYTTRRLGRRLTMLIAGVFFIIGVIFNGAAQ  131
119_DANKO_APR       VLQRKNQDKESNYCKYDNQGLQLFTSSLYLAGLTATFFASYTTRRLGRRLTMLIAGVFFIIGVIFNGAAQ  131
119_DANKO_APR2      VLQRKNQDKESNYCKYDNQGLQLFTSSLYLAGLTATFFASYTTRRLGRRLTMLIAGVFFIIGVIFNGAAQ  131
120_DANKO_APR       VLQRKNQDKESNYCKYDNQGLQLFTSSLYLAGLTATFFASYTTRRLGRRLTMLIAGVFFIIGVIFNGAAQ  131
123_DANKO_non-APR   VLQRKNQDKESNYCKYDNQGLQLFTSSLYLAGLTATFFASYTTRRLGRRLTMLIAGVFFIIGVIFNGAAQ  131
138_DANKO_APR       VLQRKNQDKESNYCKYDNQGLQLFTSSLYLAGLTATFFASYTTRRLGRRLTMLIAGVFFIIGVIFNGAAQ  131
153_DANKO_APR       VLQRKNQDKESNYCKYDNQGLQLFTSSLYLAGLTATFFASYTTRRLGRRLTMLIAGVFFIIGVIFNGAAQ  131
157_DANKO_APR       VLQRKNQDKESNYCKYDNQGLQLFTSSLYLAGLTATFFASYTTRRLGRRLTMLIAGVFFIIGVIFNGAAQ  131
160_DANKO_APR       VLQRKNQDKESNYCKYDNQGLQLFTSSLYLAGLTATFFASYTTRRLGRRLTMLIAGVFFIIGVIFNGAAQ  131
71_PHR_APR          VLQRKNQDKESNYCKYDNQGLQLFTSSLYLAGLTATFFASYTTRRLGRRLTMLIAGVFFIIGVIFNGAAQ  131
149_PHR_APR         VLQRKNQDKESNYCKYDNQGLQLFTSSLYLAGLTATFFASYTTRRLGRRLTMLIAGVFFIIGVIFNGAAQ  131
59_DANKO_non-APR    VLQRKNQDKESNYCKYDNQGLQLFTSSLYLAGLTATFFASYTTRRLGRRLTMLIAGVFFIIGVIFNGAAQ  131
61_DANKO_non-APR    VLQRKNQDKESNYCKYDNQGLQLFTSSLYLAGLTATFFASYTTRRLGRRLTMLIAGVFFIIGVIFNGAAQ  131
129_DANKO_non-APR   VLQRKNQDKESNYCKYDNQGLQLFTSSLYLAGLTATFFASYTTRRLGRRLTMLIAGVFFIIGVIFNGAAQ  131
150_PHR_non-APR     VLQRKNQDKESNYCKYDNQGLQLFTSSLYLAGLTATFFASYTTRRLGRRLTMLIAGVFFIIGVIFNGAAQ  138
150_PHR_non-APR2    VLQRKNQDKESNYCKYDNQGLQLFTSSLYLAGLTATFFASYTTRRLGRRLTMLIAGVFFIIGVIFNGAAQ  132
37_PHR_non-APR      VLQRKNQDKESNYCKYDNQGLQLFTSSLYLAGLTATFFASYTTRRLGRRLTMLIAGVFFIIGVIFNGAAQ  131
52_PHR_non-APR      VLHRKNQDKESNYCKYDNQGLQLFTSSLYLAGLTATFFASYTTRRLGRRLTMLIAGVFFIIGVIFNGAAQ  131
88_PHR_non-APR      VLQRKNQDKESNYCKYDNQGLQLFTSSLYLAGLTATFFASYTTRRLGRRLTMLIAGVFFIIGVIFNGAAQ  131
101_PHR_non-APR     VLQRKNQDKESNYCKYDNQGLQLFTSSLYLAGLTATFFASYTTRRLGRRLTMLIAGVFFIIGVIFNGAAQ  131
105_PHR_non-APR     VLQRKNQDKESNYCKYDNQGLQLFTSSLYLAGLTATFFASYTTRRLGRRLTMLIAGVFFIIGVIFNGAAQ  131
```

Sequence	150 / 160 / 170 / 180 / 190 / 200 / 210	
**Consensus**	**NLAMLIIGRILLGCGVGFANQAVPLFLSEIAPTRIRGGLNILFQLNVTIGILFANLVNSGTSKIH-----**	
Lr67(sus)	NLAMLIIGRILLGCGVGFANQAVPLFLSEIAPTRIRGGLNILFQLNVTIGILFANLVNYGTSKIH-----	196
Lr67(res)	NLAMLIIGRILLRCGVGFANQAVPLFLSEIAPTRIRGGLNILFQLNVTIGILFANLVNYGTSKIH-----	196
ScLr67-1(Lo7)	NLAMLIIGRILLGCGVGFANQAVPLFLSEIAPTRIRGGLNILFQLNVTIGILFANLVNSGTSKIH-----	196
118_DANKO_APR	NLAMLIIGRILLGCGVGFANQAVPLFLSEIAPTRIRGGLNILFQLNVTIGILFANLVNSGTSKIH-----	196
119_DANKO_APR	NLAMLIIGRILLGCGVGFANQAVPLFLSEIAPTRIRGGLNILFQLNVTIGILFANLVNSGTSKIH-----	196
119_DANKO_APR2	NLAMLIIGRILLGCGVGFANQAVPLFLSEIAPTRIRGGLNILFQLNVTIGILFANLVNSGTSKIHPWGWR	201
120_DANKO_APR	NLAMLIIGRILLGCGVGFANQAVPLFLSEIAPTRIRGGLNILFQLNVTIGILFANLVNSGTSKIH-----	196
123_DANKO_non-APR	NLAMLIIGRILLGCGVGFANQAVPLFLSEIAPTRIRGGLNILFQLNVTIGILFANLVNSGTSKIH-----	196
138_DANKO_APR	NLAMLIIGRILLGCGVGFANQAVPLFLSEIAPTRIRGGLNILFQLNVTIGILFANLVNSGTSKIH-----	196
153_DANKO_APR	NLAMLIIGRILLGCGVGFANQAVPLFLSEIAPTRIRGGLNILFQLNVTIGILFANLVNSGTSKIH-----	196
157_DANKO_APR	NLAMLIIGRILLGCGVGFANQAVPLFLSEIAPTRIRGGLNILFQLNVTIGILFANLVNSGTSKIH-----	196
160_DANKO_APR	NLAMLIIGRILLGCGVGFANQAVPLFLSEIAPTRIRGGLNILFQLNVTIGILFANLVNSGTSKIH-----	196
71_PHR_APR	NLAMLIIGRILLGCGVGFANQAVPLFLSEIAPTRIRGGLNILFQLNVTIGILFANLVNSGTSKIH-----	196
149_PHR_APR	NLAMLIIGRILLGCGVGFANQAVPLFLSEIAPTRIRGGLNILFQLNVTIGILFANLVNSGTSKIH-----	196
59_DANKO_non-APR	NLAMLIIGRILLGCGVGFANQAVPLFLSEIAPTRIRGGLNILFQLNVTIGILFANLVNSGTSKIH-----	196
61_DANKO_non-APR	NLAMLIIGRILLGCGVGFANQAVPLFLSEIAPTRIRGGLNILFQLNVTIGILFANLVNSGTSKIH-----	196
129_DANKO_non-APR	NLAMLIIGRILLGCGVGFANQAVPLFLSEIAPTRIRGGLNILFQLNVTIGILFANLVNSGTSKIH-----	196
150_PHR_non-APR	NLAMLIIGRILLGCGVGFANQAVPLFLSEIAPTRIRGGLNILFQLNVTIGILFANLVNSGTSKIH-----	203
150_PHR_non-APR2	NLAMLIIGRILLGCGVGFANQAVPLFLSEIAPTRIRGGLNILFQLNVTIGILFANLVNSGTSKIH-----	197
37_PHR_non-APR	NLAMLIIGRILLGCGVGFANQAVPLFLSEIAPTRIRGGLNILFQLNVTIGILFANLVNSGTSKIH-----	196
52_PHR_non-APR	NLAMLIIGRILLGCGVGFANQAVPLFLSEIAPTRIRGGLNILFQLNVTIGILFANLVNSGTSKIH-----	196
88_PHR_non-APR	NLAMLIIGRILLGCGVGFANQAVPLFLSEIAPTRIRGGLNILFQLNVTIGILFANLVNSGTSKIH-----	196
101_PHR_non-APR	NLAMLIIGRILLGCGVGFANQAVPLFLSEIAPTRIRGGLNILFQLNVTIGILFANLVNSGTSKIH-----	196
105_PHR_non-APR	NLAMLIIGRILLGCGVGFANQAVPLFLSEIAPTRIRGGLNILFQLNVTIGILFANLVNSGTSKIH-----	196

Sequence	220 / 230 / 240 / 250 / 260 / 270 / 280	
**Consensus**	**--PWGWRLSLSLAGIPAAMLTL**	
Lr67(sus)	--PWGWRLSLSLAGIPAAMLTL	216
Lr67(res)	--PWGWRLSLSLAGIPAAMLTL	216
ScLr67-1(Lo7)	--PWGWRLSLSLAGIPAAMLTL	216
118_DANKO_APR	--PWGWRLSLSLAGIPAAMLTL	216
119_DANKO_APR	--PWGWRLSLSLAGIPAAMLTL	216
119_DANKO_APR2	LSLSLAGIPAAMLTLGALFVTDTPNSLIERGHLEEGKAVLKRIRGTDNIHPWGWRLSLSLAGIPAAMLTL	271
120_DANKO_APR	--PWGWRLSLSLAGIPAAMLTL	216
123_DANKO_non-APR	--PWGWRLSLSLAGIPAAMLTL	216
138_DANKO_APR	--PWGWRLSLSLAGIPAAMLTL	216
153_DANKO_APR	--PWGWRLSLSLAGIPAAMLTL	216
157_DANKO_APR	--PWGWRLSLSLAGIPAAMLTL	216
160_DANKO_APR	--PWGWRLSLSLAGIPAAMLTL	216
71_PHR_APR	--PWGWRLSLSLAGIPAAMLTL	216
149_PHR_APR	--PWGWRLSLSLAGIPAAMLTL	216
59_DANKO_non-APR	--PWGWRLSLSLAGIPAAMLTL	216
61_DANKO_non-APR	--PWGWRLSLSLAGIPAAMLTL	216
129_DANKO_non-APR	--PWGWRLSLSLAGIPAAMLTL	216
150_PHR_non-APR	--PWGWRLSLSLAGIPAAMLTL	223
150_PHR_non-APR2	--PWGWRLSLSLAGIPAAMLTL	217
37_PHR_non-APR	--PWGWRLSLSLAGIPAAMLTL	216
52_PHR_non-APR	--PWGWRLSLSLAGIPAAMLTL	216
88_PHR_non-APR	--PWGWRLSLSLAGIPAAMLTL	216
101_PHR_non-APR	--PWGWRLSLSLAGIPAAMLTL	216
105_PHR_non-APR	--PWGWRLSLSLAGIPAAMLTL	216

**Consensus**	**GALFVTDTPNSLIERGHLE------------------------------EGKAVLKRIRGTDNVEPEFNE**	
	290 300 310 320 330 340 350	
Lr67(sus)	GALFVTDTPNSLIERGHLE------------------------------EGKAVLKRIRGTDNVEPEFNE	256
Lr67(res)	GALFVTDTPNSLIERGHLE------------------------------EGKAVLKRIRGTDNVEPEFNE	256
ScLr67-1(Lo7)	GALFVTDTPNSLIERGHLE------------------------------EGKAVLKRIRGTDNVEPEFNE	256
118_DANKO_APR	GALFVTDTPNSLIERGHLE------------------------------EGKAVLKRIRGTDNVEPEFNE	256
119_DANKO_APR	GALFVTDTPNSLIERGHLEEGKAVLKRIRGTDNVPVREPGELRHQQDPPEGKAVLKRIRGTDNVEPEFNE	286
119_DANKO_APR2	GALFVTDTPNSLIERGHLE------------------------------EGKAVLKRIRGTDNVEPEFNE	311
120_DANKO_APR	GALFVTDTPNSLIERGHLE------------------------------EGKAVLKRIRGTDNVEPEFNE	256
123_DANKO_non-APR	GALFVTDTPNSLIERGHLE------------------------------EGKAVLKRIRGTDNVEPEFNE	256
138_DANKO_APR	GALFVTDTPNSLIERGHLE------------------------------EGKAVLKRIRGTDNVEPEFNE	256
153_DANKO_APR	GALFVTDTPNSLIERGHLE------------------------------EGKAVLKRIRGTDNVEPEFNE	256
157_DANKO_APR	GALFVTDTPNSLIERGHLE------------------------------EGKAVLKRIRGTDNVEPEFNE	256
160_DANKO_APR	GALFVTDTPNSLIERGHLE------------------------------EGKAVLKRIRGTDNVEPEFNE	256
71_PHR_APR	GALFVTDTPNSLIERGHLE------------------------------EGKAVLKRIRGTDNVEPEFNE	256
149_PHR_APR	GALFVTDTPNSLIERGHLE------------------------------EGKAVLKRIRGTDNVEPEFNE	256
59_DANKO_non-APR	GALFVTDTPNSLIERGHLE------------------------------EGKAVLKRIRGTDNVEPEFNE	256
61_DANKO_non-APR	GALFVTDTPNSLIERGHLE------------------------------EGKAVLKRIRGTDNVEPEFNE	256
129_DANKO_non-APR	GALFVTDTPNSLIERGHLE------------------------------EGKAVLKRIRGTDNVEPEFNE	256
150_PHR_non-APR	GALFVTDTPNSLIERGHLE------------------------------EGKAVLKRIRGTDNVEPEFNE	263
150_PHR_non-APR2	GALFVTDTPNSLIERGHLE------------------------------EGKAVLKRIRGTDNVEPEFNE	257
37_PHR_non-APR	GALFVTDTPNSLIERGHLE------------------------------EGKAVLKRIRGTDNVEPEFNE	256
52_PHR_non-APR	GALFVTDTPNSLIERGHLE------------------------------EGKAVLKRIRGTDNVEPEFNE	256
88_PHR_non-APR	GALFVTDTPNSLIERGHLE------------------------------EGKAVLKRIRGTDNVEPEFNE	256
101_PHR_non-APR	GALFVTDTPNSLIERGHLE------------------------------EGKAVLKRIRGTDNVEPEFNE	256
105_PHR_non-APR	GALFVTDTPNSLIERGHLE------------------------------EGKAVLKRIRGTDNVEPEFNE	256

**Consensus**	**IVEASRIAQEVKHPFRNLLQRRNRPQLVIAVLLQIFQQFTGINAIMFYAPVLFNTLGFKSDASLYSAVIT**	
	360 370 380 390 400 410 420	
Lr67(sus)	IVEASRIAQEVKHPFRNLLQRRNRPQLVIAVLLQIFQQFTGINAIMFYAPVLFNTLGFKSDASLYSAVIT	326
Lr67(res)	IVEASRIAQEVKHPFRNLLQRRNRPQLVIAVLLQIFQQFTGINAIMFYAPVLFNTLGFKSDASLYSAVIT	326
ScLr67-1(Lo7)	IVEASRIAQEVKHPFRNLLQRRNRPQLVIAVLLQIFQQFTGINAIMFYAPVLFNTLGFKSDASLYSAVIT	326
118_DANKO_APR	IVEASRIAQEVKHPFRNLLQRRNRPQLVIAVLLQIFQQFTGINAIMFYAPVLFNTLGFKSDASLYSAVIT	326
119_DANKO_APR	IVEASRIAQEVKHPFRNLLQRRNRPQLVIAVLLQIFQQFTGINAIMFYAPVLFNTLGFKSDASLYSAVIT	356
119_DANKO_APR2	IVEASRIAQEVKHPFRNLLQRRNRPQLVIAVLLQIFQQFTGINAIMFYAPVLFNTLGFKSDASLYSAVIT	381
120_DANKO_APR	IVEASRIAQEVKHPFRNLLQRRNRPQLVIAVLLQIFQQFTGINAIMFYAPVLFNTLGFKSDASLYSAVIT	326
123_DANKO_non-APR	IVEASRIAQEVKHPFRNLLQRRNRPQLVIAVLLQIFQQFTGINAIMFYAPVLFNTLGFKSDASLYSAVIT	326
138_DANKO_APR	IVEASRIAQEVKHPFRNLLQRRNRPQLVIAVLLQIFQQFTGINAIMFYAPVLFNTLGFKSDASLYSAVIT	326
153_DANKO_APR	IVEASRIAQEVKHPFRNLLQRRNRPQLVIAVLLQIFQQFTGINAIMFYAPVLFNTLGFKSDASLYSAVIT	326
157_DANKO_APR	IVEASRIAQEVKHPFRNLLQRRNRPQLVIAVLLQIFQQFTGINAIMFYAPVLFNTLGFKSDASLYSAVIT	326
160_DANKO_APR	IVEASRIAQEVKHPFRNLLQRRNRPQLVIAVLLQIFQQFTGINAIMFYAPVLFNTLGFKSDASLYSAVIT	326
71_PHR_APR	IVEASRIAQEVKHPFRNLLQRRNRPQLVIAVLLQIFQQFTGINAIMFYAPVLFNTLGFKSDASLYSAVIT	326
149_PHR_APR	IVEASRIAQEVKHPFRNLLQRRNRPQLVIAVLLQIFQQFTGINAIMFYAPVLFNTLGFKSDASLYSAVIT	326
59_DANKO_non-APR	IVEASRIAQEVKHPFRNLLQRRNRPQLVIAVLLQIFQQFTGINAIMFYAPVLFNTLGFKSDASLYSAVIT	326
61_DANKO_non-APR	IVEASRIAQEVKHPFRNLLQRRNRPQLVIAVLLQIFQQFTGINAIMFYAPVLFNTLGFKSDASLYSAVIT	326
129_DANKO_non-APR	IVEASRIAQEVKHPFRNLLQRRNRPQLVIAVLLQIFQQFTGINAIMFYAPVLFNTLGFKSDASLYSAVIT	326
150_PHR_non-APR	IVEASRIAQEVKHPFRNLLQRRNRPQLVIAVLLQIFQQFTGINAIMFYAPVLFNTLGFKSDASLYSAVIT	333
150_PHR_non-APR2	IVEASRIAQEVKHPFRNLLQRRNRPQLVIAVLLQIFQQFTGINAIMFYAPVLFNTLGFKSDASLYSAVIT	327
37_PHR_non-APR	IVEASRIAQEVKHPFRNLLQRRNRPQLVIAVLLQIFQQFTGINAIMFYAPVLFNTLGFKSDASLYSAVIT	326
52_PHR_non-APR	IVEASRIAQEVKHPFRNLLQRRNRPQLVIAVLLQIFQQFTGINAIMFYAPVLFNTLGFKSDASLYSAVIT	326
88_PHR_non-APR	IVEASRIAQEVKHPFRNLLQRRNRPQLVIAVLLQIFQQFTGINAIMFYAPVLFNTLGFKSDASLYSAVIT	326
101_PHR_non-APR	IVEASRIAQEVKHPFRNLLQRRNRPQLVIAVLLQIFQQFTGINAIMFYAPVLFNTLGFKSDASLYSAVIT	326
105_PHR_non-APR	IVEASRIAQEVKHPFRNLLQRRNRPQLVIAVLLQIFQQFTGINAIMFYAPVLFNTLGFKSDASLYSAVIT	326

Consensus	GAVNVLATLVSVYAVDRAGRRALLLEAGVQMFVSQVVIAVVLGIKVTDKSDNLGHGWAILVVVMVCTYVA	
	430 440 450 460 470 480 490	
Lr67(sus)	GAVNVLATLVSVYAVDRAGRRALLLEAGVQMFLSQVVIAVVLGIKVTDKSDNLGHGWAILVVVMVCTYVA	396
Lr67(res)	GAVNVLATLVSVYAVDRAGRRALLLEAGVQMFLSQVVIAVVLGIKVTDKSDNLGHGWAILLVVMVCTYVA	396
ScLr67-1(Lo7)	GAVNVLATLVSVYAVDRAGRRALLLEAGVQMFVSQVVIAVVLGIKVTDKSDNLGHGWAILVVVMVCTYVA	396
118_DANKO_APR	GAVNVLATLVSVYAVDRAGRRALLLEAGVQMFVSQVVIAVVLGIKVTDKSDNLGHGWAILVVVMVCTYVA	396
119_DANKO_APR	GAVNVLATLVSVYAVDRAGRRALLLEAGVQMFVSQVVIAVVLGIKVTDKSDNLGHGWAILVVVMVCTYVA	426
119_DANKO_APR2	GAVNVLATLVSVYAVDRAGRRALLLEAGVQMFVSQVVIAVVLGIKVTDKSDNLGHGWAILVVVMVCTYVA	451
120_DANKO_APR	GAVNVLATLVSVYAVDRAGRRALLLEAGVQMFVSQVVIAVVLGIKVTDKSDNLGHGWAILVVVMVCTYVA	396
123_DANKO_non-APR	GAVNVLATLVSVYAVDRAGRRALLLEAGVQMFVSQVVIAVVLGIKVTDKSDNLGHGWAILVVVMVCTYVA	396
138_DANKO_APR	GAVNVLATLVSVYAVDRAGRRALLLEAGVQMFVSQVVIAVVLGIKVTDKSDNLGHGWAILVVVMVCTYVA	396
153_DANKO_APR	GAVNVLATLVSVYAVDRAGRRALLLEAGVQMFVSQVVIAVVLGIKVTDKSDNLGHGWAILVVVMVCTYVA	396
157_DANKO_APR	GAVNVLATLVSVYAVDRAGRRALLLEAGVQMFVSQVVIAVVLGIKVTDKSDNLGHGWAILVVVMVCTYVA	396
160_DANKO_APR	GAVNVLATLVSVYAVDRAGRRALLLEAGVQMFVSQVVIAVVLGIKVTDKSDNLGHGWAILVVVMVCTYVA	396
71_PHR_APR	GAVNVLATLVSVYAVDRAGRRALLLEAGVQMFVSQVVIAVVLGIKVTDKSDNLGHGWAILVVVMVCTYVA	396
149_PHR_APR	GAVNVLATLVSVYAVDRAGRRALLLEAGVQMFVSQVVIAVVLGIKVTDKSDNLGHGWAILVVVMVCTYVA	396
59_DANKO_non-APR	GAVNVLATLVSVYAVDRAGRRALLLEAGVQMFVSQVVIAVVLGIKVTDKSDNLGHGWAILVVVMVCTYVA	396
61_DANKO_non-APR	GAVNVLATLVSVYAVDRAGRRALLLEAGVQMFVSQVVIAVVLGIKVTDKSDNLGHGWAILVVVMVCTYVA	396
129_DANKO_non-APR	GAVNVLATLVSVYAVDRAGRRALLLEAGVQMFVSQVVIAVVLGIKVTDKSDNLGHGWAILVVVMVCTYVA	396
150_PHR_non-APR	GAVNVLATLVSVYAVDRAGRRALLLEAGVQMFVSQVVIAVVLGIKVTDKSDNLGHGWAILVVVMVCTYVA	403
150_PHR_non-APR2	GAVNVLATLVSVYAVDRAGRRALLLEAGVQMFVSQVVIAVVLGIKVTDKSDNLGHGWAILVVVMVCTYVA	397
37_PHR_non-APR	GAVNVLATLVSVYAVDRAGRRALLLEAGVQMFVSQVVIAVVLGIKVTDKSDNLGHGWAILVVVMVCTYVA	396
52_PHR_non-APR	GAVNVLATLVSVYAVDRAGRRALLLEAGVQMFVSQVVIAVVLGIKVTDKSDNLGHGWAILVVVMVCTYVA	396
88_PHR_non-APR	GAVNVLATLVSVYAVDRAGRRALLLEAGVQMFVSQVVIAVVLGIKVTDKSDNLGHGWAILVVVMVCTYVA	396
101_PHR_non-APR	GAVNVLATLVSVYAVDRAGRRALLLEAGVQMFVSQVVIAVVLGIKVTDKSDNLGHGWAILVVVMVCTYVA	396
105_PHR_non-APR	GAVNVLATLVSVYAVDRAGRRALLLEAGVQMFVSQVVIAVVLGIKVTDKSDNLGHGWAILVVVMVCTYVA	396

Consensus	SFAWSWGPLGWLIPSETFPLETRSAGQSVTVCVNLLFTFLIAQAFLSMLCHLKFAIFIFFSAWVLVMSVF	
	500 510 520 530 540 550 560	
Lr67(sus)	SFAWSWGPLGWLIPSETFPLETRSAGQSVTVCVNLLFTFLIAQAFLSMLCHLKFAIFIFFSAWVLVMSVF	466
Lr67(res)	SFAWSWGPLGWLIPSETFPLETRSAGQSVTVCVNLLFTFLIAQAFLSMLCHLKFAIFIFFSAWVLVMSVF	466
ScLr67-1(Lo7)	SFAWSWGPLGWLIPSETFPLETRSAGQSVTVCVNLLFTFLIAQAFLSMLCHLKFAIFIFFSAWVLVMSVF	466
118_DANKO_APR	SFAWSWGPLGWLIPSETFPLETRSAGQSVTVCVNLLFTFLIAQAFLSMLCHLKFAIFIFFSAWVLVMSVF	466
119_DANKO_APR	SFAWSWGPLGWLIPSETFPLETRSAGQSVTVCVNLLFTFLIAQAFLSMLCHLKFAIFIFFSAWVLVMSVF	496
119_DANKO_APR2	SFAWSWGPLGWLIPSETFPLETRSAGQSVTVCVNLLFTFLIAQAFLSMLCHLKFAIFIFFSAWVLVMSVF	521
120_DANKO_APR	SFAWSWGPLGWLIPSETFPLETRSAGQSVTVCVNLLFTFLIAQAFLSMLCHLKFAIFIFFSAWVLVMSVF	466
123_DANKO_non-APR	SFAWSWGPLGWLIPSETFPLETRSAGQSVTVCVNLLFTFLIAQAFLSMLCHLKFAIFIFFSAWVLVMSVF	466
138_DANKO_APR	SFAWSWGPLGWLIPSETFPLETRSAGQSVTVCVNLLFTFLIAQAFLSMLCHLKFAIFIFFSAWVLVMSVF	466
153_DANKO_APR	SFAWSWGPLGWLIPSDTFPLETRSAGQSVTVCVNLLFTFLIAQAFLSMLCHLKFAIFIFFSAWVLVMSVF	466
157_DANKO_APR	SFAWSWGPLGWLIPSETFPLETRSAGQSVTVCVNLLFTFLIAQAFLSMLCHLKFAIFIFFSAWVLVMSVF	466
160_DANKO_APR	SFAWSWGPLGWLIPSETFPLETRSAGQSVTVCVNLLFTFLIAQAFLSMLCHLKFAIFIFFSAWVLVMSVF	466
71_PHR_APR	SFAWSWGPLGWLIPSETFPLETRSAGQSVTVCVNLLFTFLIAQAFLSMLCHLKFAIFIFFSAWVLVMSVF	466
149_PHR_APR	SFAWSWGPLGWLIPSETFPLETRSAGQSVTVCVNLLFTFLIAQAFLSMLCHLKFAIFIFFSAWVLVMSVF	466
59_DANKO_non-APR	SFAWSWGPLGWLIPSETFPLETRSAGQSVTVCVNLLFTFLIAQAFLSMLCHLKFAIFIFFSAWVLVMSVF	466
61_DANKO_non-APR	SFAWSWGPLGWLIPSETFPLETRSAGQSVTVCVNLLFTFLIAQAFLSMLCHLKFAIFIFFSAWVLVMSVF	466
129_DANKO_non-APR	SFAWSWGPLGWLIPSETFPLETRSAGQSVTVCVNLLFTFLIAQAFLSMLCHLKFAIFIFFSAWVLVMSVF	466
150_PHR_non-APR	SFAWSWGPLGWLIPSETFPLETRSAGQSVTVCVNLLFTFLIAQAFLSMLCHLKFAIFIFFSAWVLVMSVF	473
150_PHR_non-APR2	SFAWSWGPLGWLIPSETFPLETRSAGQSVTVCVNLLFTFLIAQAFLSMLCHLKFAIFIFFSAWVLVMSVF	467
37_PHR_non-APR	SFAWSWGPLGWLIPSETFPLETRSAGQSVTVCVNLLFTFLIAQAFLSMLCHLKFAIFIFFSAWVLVMSVF	466
52_PHR_non-APR	SFAWSWGPLGWLIPSETFPLETRSAGQSVTVCVNLLFTFLIAQAFLSMLCHLKFAIFIFFSAWVLVMSVF	466
88_PHR_non-APR	SFAWSWGPLGWLIPSETFPLETRSAGQSVTVCVNLLFTFLIAQAFLSMLCHLKFAIFIFFSAWVLVMSVF	466
101_PHR_non-APR	SFAWSWGPLGWLIPSETFPLETRSAGQSVTVCVNLLFTFLIAQAFLSMLCHLKFAIFIFFSAWVLVMSVF	466
105_PHR_non-APR	SFAWSWGPLGWLIPSETFPLETRSAGQSVTVCVNLLFTFLIAQAFLSMLCHLKFAIFIFFSAWVLVMSVF	466

**Consensus**	**VLFFLPETKNVPIEEMTDKVWKQHWFWKRYMDDDDHH--IANGKNVTV**	
	570 580 590 600	
Lr67(sus)	VLFFLPETKNVPIEEMTDKVWKQHWFWKRFMDDDDHHHNIANGKNATV	514
Lr67(res)	VLFFLPETKNVPIEEMTDKVWKQHWFWKRFMDDDDHHHNIANGKNATV	514
ScLr67-1(Lo7)	VLFFLPETKNVPIEEMTDKVWKQHWFWKRYMDDDDHH--IANGKNVTV	512
118_DANKO_APR	VLFFLPETKNVPIEEMTDKVWKQHWFWKRYMDDDDHH--IANGKNVTV	512
119_DANKO_APR	VLFFLPETKNVPIEEMTDKVWKQHWFWKRYMDDDDHH--IANGKNVTV	542
119_DANKO_APR2	VLFFLPETKNVPIEEMTDKVWKQHWFWKRYMDDDDHH--IANGKNVTV	567
120_DANKO_APR	VLFFLPETKNVPIEEMTDKVWKQHWFWKRYMDDDDHH--IANGKNVTV	512
123_DANKO_non-APR	VLFFLPETKNVPIEEMTDKVWKQHWFWKRYMDDDDHH--IANGKNVTV	512
138_DANKO_APR	VLFFLPETKNVPIEEMTDKVWKQHWFWKRYMDDDDHH--IANGKNVTV	512
153_DANKO_APR	VLFFLPETKNVPIEEMTDKVWKQHWFWKRYMDDDDHH--IANGKNVTV	512
157_DANKO_APR	VLFFLPETKNVPIEEMTDKVWKQHWFWKRYMDDDDHH--IANGKNVTV	512
160_DANKO_APR	VLFFLPETKNVPIEEMTDKVWKQHWFWKRYMDDDDHH--IANGKNVTV	512
71_PHR_APR	VLFFLPETKNVPIEEMTDKVWKQHWFWKRYMDDDDHH--IANGKNVTV	512
149_PHR_APR	VLFFLPETKNVPIEEMTDKVWKQHWFWKRYMDDDDHH--IANGKNVTV	512
59_DANKO_non-APR	VLFFLPETKNVPIEEMTDKVWKQHWFWKRYMDDDDHH--IANGKNVTV	512
61_DANKO_non-APR	VLFFLPETKNVPIEEMTDKVWKQHWFWKRYMDDDDHH--IANGKNVTV	512
129_DANKO_non-APR	VLFFLPETKNVPIEEMTDKVWKQHWFWKRYMDDDDHH--IANGKNVTV	512
150_PHR_non-APR	VLFFLPETKNVPIEEMTDKVWKQHWFWKRYMDDDDHH--IANGKNVTV	519
150_PHR_non-APR2	VLFFLPETKNVPIEEMTDKVWKQHWFWKRYMDDDDHH--IANGKNVTV	513
37_PHR_non-APR	VLFFLPETKNVPIEEMTDKVWKQHWFWKRYMDDDDHH--IANGKNVTV	512
52_PHR_non-APR	VLFFLPETKNVPIEEMTDKVWKQHWFWKRYMDDDDHH--IANGKNVTV	512
88_PHR_non-APR	VLFFLPETKNVPIEEMTDKVWKQHWFWKRYMDDDDHH--IANGKNVTV	512
101_PHR_non-APR	VLFFLPETKNVPIEEMTDKVWKQHWFWKRYMDDDDHH--IANGKNVTV	512
105_PHR_non-APR	VLFFLPETKNVPIEEMTDKVWKQHWFWKRYMDDDDHH--IANGKNVTV	512

Consensus Threshold: >50%

Colors: properties + conservation (Clustal X)

Created: 26 lis 2024

Last Modified: 26 lis 2024

Feature	Location	Size	⇄	Type
