# Supplementary material for: Searching for genes determining the APR phenotype in rye
Source: BMC Plant Biol. 2025 Jul 19;25:935. doi: 10.1186/s12870-025-06920-0 (PMC12275401; doi:10.1186/s12870-025-06920-0)
Supplement: Supplementary file 6 — Supplementary Material 6. [file 12870_2025_6920_MOESM6_ESM.pdf]

Aligned using MUSCLE

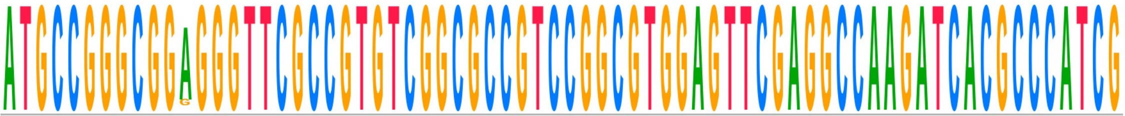

**Consensus**	ATGCCGGGCGGAGGGTTCGCCGTGTCGGCGCCGTCCGGCGTGGAGTTCGAGGCCAAGATCACGCCCATCG	
	10 20 30 40 50 60 70	
Lr67(sus)	ATGCCGGGCGGGGGGTTCGCCGTGTCGGCGCCGTCCGGCGTGGAGTTCGAGGCCAAGATCACGCCCATCG	70
Lr67(res)	ATGCCGGGCGGGGGGTTCGCCGTGTCGGCGCCGTCCGGCGTGGAGTTCGAGGCCAAGATCACGCCCATCG	70
ScLr67_1 (Lo7)	ATGCCGGGCGGAGGGTTCGCCGTGTCGGCGCCGTCCGGCGTGGAGTTCGAGGCCAAGATCACGCCCATCG	70
118_Danko_APR	ATGCCGGGCGGAGGGTTCGCCGTGTCGGCGCCGTCCGGCGTGGAGTTCGAGGCCAAGATCACGCCCATCG	70
119_Danko_APR	ATGCCGGGCGGAGGGTTCGCCGTGTCGGCGCCGTCCGGCGTGGAGTTCGAGGCCAAGATCACGCCCATCG	70
119_Danko_APR2	ATGCCGGGCGGAGGGTTCGCCGTGTCGGCGCCGTCCGGCGTGGAGTTCGAGGCCAAGATCACGCCCATCG	70
120_Danko_APR	ATGCCGGGCGGAGGGTTCGCCGTGTCGGCGCCGTCCGGCGTGGAGTTCGAGGCCAAGATCACGCCCATCG	70
138_Danko_APR	ATGCCGGGCGGAGGGTTCGCCGTGTCGGCGCCGTCCGGCGTGGAGTTCGAGGCCAAGATCACGCCCATCG	70
153_Danko_APR	ATGCCGGGCGGAGGGTTCGCCGTGTCGGCGCCGTCCGGCGTGGAGTTCGAGGCCAAGATCACGCCCATCG	70
157_Danko_APR	ATGCCGGGCGGAGGGTTCGCCGTGTCGGCGCCGTCCGGCGTGGAGTTCGAGGCCAAGATCACGCCCATCG	70
160_Danko_APR	ATGCCGGGCGGAGGGTTCGCCGTGTCGGCGCCGTCCGGCGTGGAGTTCGAGGCCAAGATCACGCCCATCG	70
71_PHR_APR	ATGCCGGGCGGAGGGTTCGCCGTGTCGGCGCCGTCCGGCGTGGAGTTCGAGGCCAAGATCACGCCCATCG	70
149_PHR_APR	ATGCCGGGCGGAGGGTTCGCCGTGTCGGCGCCGTCCGGCGTGGAGTTCGAGGCCAAGATCACGCCCATCG	70
59_Danko_non-APR	ATGCCGGGCGGAGGGTTCGCCGTGTCGGCGCCGTCCGGCGTGGAGTTCGAGGCCAAGATCACGCCCATCG	70
61_Danko_non-APR	ATGCCGGGCGGAGGGTTCGCCGTGTCGGCGCCGTCCGGCGTGGAGTTCGAGGCCAAGATCACGCCCATCG	70
123_Danko_non-APR	ATGCCGGGCGGAGGGTTCGCCGTGTCGGCGCCGTCCGGCGTGGAGTTCGAGGCCAAGATCACGCCCATCG	70
129_Danko_non-APR	ATGCCGGGCGGAGGGTTCGCCGTGTCGGCGCCGTCCGGCGTGGAGTTCGAGGCCAAGATCACGCCCATCG	70
37_PHR_non-APR	ATGCCGGGCGGAGGGTTCGCCGTGTCGGCGCCGTCCGGCGTGGAGTTCGAGGCCAAGATCACGCCCATCG	70
52_PHR_non-APR	ATGCCGGGCGGAGGGTTCGCCGTGTCGGCGCCGTCCGGCGTGGAGTTCGAGGCCAAGATCACGCCCATCG	70
88_PHR_non-APR	ATGCCGGGCGGAGGGTTCGCCGTGTCGGCGCCGTCCGGCGTGGAGTTCGAGGCCAAGATCACGCCCATCG	70
101_PHR_non-APR	ATGCCGGGCGGAGGGTTCGCCGTGTCGGCGCCGTCCGGCGTGGAGTTCGAGGCCAAGATCACGCCCATCG	70
105_PHR_non-APR	ATGCCGGGCGGAGGGTTCGCCGTGTCGGCGCCGTCCGGCGTGGAGTTCGAGGCCAAGATCACGCCCATCG	70
150_PHR_non-APR	ATGCCGGGCGGAGGGTTCGCCGTGTCGGCGCCGTCCGGCGTGGAGTTCGAGGCCAAGATCACGCCCATCG	70
150_PHR_non-APR2	ATGCCGGGCGGAGGGTTCGCCGTGTCGGCGCCGTCCGGCGTGGAGTTCGAGGCCAAGATCACGCCCATCG	70

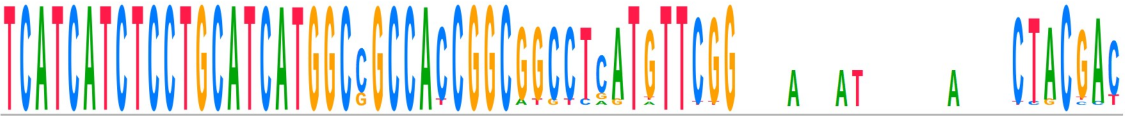

**Consensus**	TCATCATCTCCTGCATCATGGCCGCCACCGGCGGCCTCATGTTCGG-----------------CTACGAC	
	80 90 100 110 120 130 140	
Lr67(sus)	TCATCATCTCCTGCATCATGGCGGCCACCGGCGGCCTCATGTTCGG-----------------CTACGAC	123
Lr67(res)	TCATCATCTCCTGCATCATGGCGGCCACCGGCGGCCTCATGTTCGG-----------------CTACGAC	123
ScLr67_1 (Lo7)	TCATCATCTCCTGCATCATGGCGGCCACCGGCGGCCTGATGTTCGG-----------------CTACGAC	123
118_Danko_APR	TCATCATCTCCTGCATCATGGCCGCCACCGGCGGCCTCATGTTCGG-----------------CTACGAC	123
119_Danko_APR	TCATCATCTCCTGCATCATGGCCGCCACCGGCGGCCTCATGTTCGG-----------------CTACGAC	123
119_Danko_APR2	TCATCATCTCCTGCATCATGGCCGCCACCGGCGGCCTCATGTTCGG-----------------CTACGAC	123
120_Danko_APR	TCATCATCTCCTGCATCATGGCCGCCACCGGCGGCCTCATGTTCGG-----------------CTACGAC	123
138_Danko_APR	TCATCATCTCCTGCATCATGGCCGCCACCGGCGGCCTCATGTTCGG-----------------CTACGAC	123
153_Danko_APR	TCATCATCTCCTGCATCATGGCCGCCACCGGCGGCCTCATGTTCGG-----------------CTACGAC	123
157_Danko_APR	TCATCATCTCCTGCATCATGGCCGCCACCGGCGGCCTCATGTTCGG-----------------CTACGAC	123
160_Danko_APR	TCATCATCTCCTGCATCATGGCCGCCACCGGCGGCCTCATGTTCGG-----------------CTACGAC	123
71_PHR_APR	TCATCATCTCCTGCATCATGGCCGCCACCGGCGGGCTCATGTTCGG-----------------CTACGAT	123
149_PHR_APR	TCATCATCTCCTGCATCATGGCCGCCACCGGCGGCCTCATGTTCGG-----------------CTACGAC	123
59_Danko_non-APR	TCATCATCTCCTGCATCATGGCCGCCACCGGCGGGCTCATGTTCGG-----------------CTACGAT	123
61_Danko_non-APR	TCATCATCTCCTGCATCATGGCGGCCACCGGCGGCCTGATGTTCGG-----------------CTACGAC	123
123_Danko_non-APR	TCATCATCTCCTGCATCATGGCCGCCACCGGCGGCCTCATGTTCGG-----------------CTACGAC	123
129_Danko_non-APR	TCATCATCTCCTGCATCATGGCCGCCACCGGCGGCCTCATGTTCGG-----------------CTACGAC	123
37_PHR_non-APR	TCATCATCTCCTGCATCATGGCCGCCACCGGCGGCCTCATGTTCGG-----------------CTACGAC	123
52_PHR_non-APR	TCATCATCTCCTGCATCATGGCGGCCACCGGCGGCCTGATGTTCGG-----------------CTACGAC	123
88_PHR_non-APR	TCATCATCTCCTGCATCATGGCCGCCACCGGCGGCCTCATGTTCGG-----------------CTACGAC	123
101_PHR_non-APR	TCATCATCTCCTGCATCATGGCCGCCACCGGCGGCCTCATGTTCGG-----------------CTACGAC	123
105_PHR_non-APR	TCATCATCTCCTGCATCATGGCCGCCACCGGCGGCCTCATGTTCGG-----------------CTACGAC	123
150_PHR_non-APR	TCATCATCTCCTGCAT-----------TCGGCATCTCAGTATTTGGAAGAAAATAGATAACTGCTGCCAT	129
150_PHR_non-APR2	TCATCATCTCCTGCAT-----------TCGGCATCTCAGTTTTCTGGCCAGGATCATATATACTCGCTCT	129

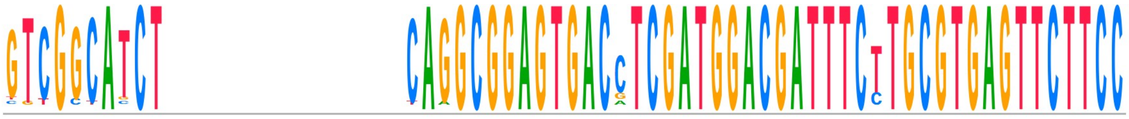

**Consensus**	GTCGGCATCT---------------CAGGCGGAGTGACCTCGATGGACGATTTCTTGCGTGAGTTCTTCC	
	150 160 170 180 190 200 210	
Lr67(sus)	GTCGGCATCT---------------CAGGCGGAGTGACATCGATGGACGATTTCCTGCGTGAGTTCTTCC	178
Lr67(res)	GTCGGCATCT---------------CAGGCGGAGTGACATCGATGGACGATTTCCTGCGTGAGTTCTTCC	178
ScLr67_1 (Lo7)	GTCGGCATCT---------------CAGGCGGAGTGACGTCGATGGACGATTTCCTGCGTGAGTTCTTCC	178
118_Danko_APR	GTCGGCATCT---------------CAGGCGGAGTGACCTCGATGGACGATTTCTTGCGTGAGTTCTTCC	178
119_Danko_APR	GTCGGCATCT---------------CAGGCGGAGTGACCTCGATGGACGATTTCTTGCGTGAGTTCTTCC	178
119_Danko_APR2	GTCGGCATCT---------------CAGGCGGAGTGACCTCGATGGACGATTTCTTGCGTGAGTTCTTCC	178
120_Danko_APR	GTCGGCATCT---------------CAGGCGGAGTGACCTCGATGGACGATTTCTTGCGTGAGTTCTTCC	178
138_Danko_APR	GTCGGCATCT---------------CAGGCGGAGTGACCTCGATGGACGATTTCTTGCGTGAGTTCTTCC	178
153_Danko_APR	GTCGGCATCT---------------CAGGCGGAGTGACCTCGATGGACGATTTCTTGCGTGAGTTCTTCC	178
157_Danko_APR	GTCGGCATCT---------------CAGGCGGAGTGACCTCGATGGACGATTTCTTGCGTGAGTTCTTCC	178
160_Danko_APR	GTCGGCATCT---------------CAGGCGGAGTGACCTCGATGGACGATTTCTTGCGTGAGTTCTTCC	178
71_PHR_APR	GTCGGCATCT---------------CAGGCGGAGTGACCTCGATGGACGATTTCTTGCGTGAGTTCTTCC	178
149_PHR_APR	GTCGGCATCT---------------CAGGCGGAGTGACCTCGATGGACGATTTCTTGCGTGAGTTCTTCC	178
59_Danko_non-APR	GTCGGCATCT---------------CAGGCGGAGTGACCTCGATGGACGATTTCTTGCGTGAGTTCTTCC	178
61_Danko_non-APR	GTCGGCATCT---------------CAGGCGGAGTGACGTCGATGGACGATTTCCTGCGTGAGTTCTTCC	178
123_Danko_non-APR	GTCGGCATCT---------------CAGGCGGAGTGACCTCGATGGACGATTTCTTGCGTGAGTTCTTCC	178
129_Danko_non-APR	GTCGGCATCT---------------CAGGCGGAGTGACCTCGATGGACGATTTCTTGCGTGAGTTCTTCC	178
37_PHR_non-APR	GTCGGCATCT---------------CAGGCGGAGTGACCTCGATGGACGATTTCTTGCGTGAGTTCTTCC	178
52_PHR_non-APR	GTCGGCATCT---------------CAGGCGGAGTGACGTCGATGGACGATTTCCTGCGTGAGTTCTTCC	178
88_PHR_non-APR	GTCGGCATCT---------------CAGGCGGAGTGACCTCGATGGACGATTTCTTGCGTGAGTTCTTCC	178
101_PHR_non-APR	GTCGGCATCT---------------CAGGCGGAGTGACCTCGATGGACGATTTCTTGCGTGAGTTCTTCC	178
105_PHR_non-APR	GTCGGCATCT---------------CAGGCGGAGTGACCTCGATGGACGATTTCTTGCGTGAGTTCTTCC	178
150_PHR_non-APR	CTTGCCACCTACTTTGATAGGCTGTTAAGCGGAGTGACCTCGATGGACGATTTCTTGCGTGAGTTCTTCC	199
150_PHR_non-APR2	TGTGCTAGCT------------------GCGGAGTGACCTCGATGGACGATTTCTTGCGTGAGTTCTTCC	181

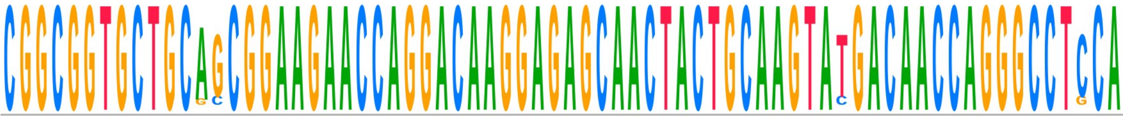

**Consensus**	CGGCGGTGCTGCAGCGGAAGAACCAGGACAAGGAGAGCAACTACTGCAAGTATGACAACCAGGGCCTCCA	
	220 230 240 250 260 270 280	
Lr67(sus)	CGGCGGTGCTGCGCCGGAAGAACCAGGACAAGGAGAGCAACTACTGCAAGTACGACAACCAGGGCCTGCA	248
Lr67(res)	CGGCGGTGCTGCGCCGGAAGAACCAGGACAAGGAGAGCAACTACTGCAAGTACGACAACCAGGGCCTGCA	248
ScLr67_1 (Lo7)	CGGCGGTGCTGCAGCGGAAGAACCAGGACAAGGAGAGCAACTACTGCAAGTATGACAACCAGGGCCTCCA	248
118_Danko_APR	CGGCGGTGCTGCAGCGGAAGAACCAGGACAAGGAGAGCAACTACTGCAAGTATGACAACCAGGGCCTCCA	248
119_Danko_APR	CGGCGGTGCTGCAGCGGAAGAACCAGGACAAGGAGAGCAACTACTGCAAGTATGACAACCAGGGCCTCCA	248
119_Danko_APR2	CGGCGGTGCTGCAGCGGAAGAACCAGGACAAGGAGAGCAACTACTGCAAGTATGACAACCAGGGCCTCCA	248
120_Danko_APR	CGGCGGTGCTGCAGCGGAAGAACCAGGACAAGGAGAGCAACTACTGCAAGTATGACAACCAGGGCCTCCA	248
138_Danko_APR	CGGCGGTGCTGCAGCGGAAGAACCAGGACAAGGAGAGCAACTACTGCAAGTATGACAACCAGGGCCTCCA	248
153_Danko_APR	CGGCGGTGCTGCAGCGGAAGAACCAGGACAAGGAGAGCAACTACTGCAAGTATGACAACCAGGGCCTCCA	248
157_Danko_APR	CGGCGGTGCTGCAGCGGAAGAACCAGGACAAGGAGAGCAACTACTGCAAGTATGACAACCAGGGCCTCCA	248
160_Danko_APR	CGGCGGTGCTGCAGCGGAAGAACCAGGACAAGGAGAGCAACTACTGCAAGTATGACAACCAGGGCCTCCA	248
71_PHR_APR	CGGCGGTGCTGCAGCGGAAGAACCAGGACAAGGAGAGCAACTACTGCAAGTATGACAACCAGGGCCTCCA	248
149_PHR_APR	CGGCGGTGCTGCAGCGGAAGAACCAGGACAAGGAGAGCAACTACTGCAAGTATGACAACCAGGGCCTCCA	248
59_Danko_non-APR	CGGCGGTGCTGCAGCGGAAGAACCAGGACAAGGAGAGCAACTACTGCAAGTATGACAACCAGGGCCTCCA	248
61_Danko_non-APR	CGGCGGTGCTGCAGCGGAAGAACCAGGACAAGGAGAGCAACTACTGCAAGTATGACAACCAGGGCCTCCA	248
123_Danko_non-APR	CGGCGGTGCTGCAGCGGAAGAACCAGGACAAGGAGAGCAACTACTGCAAGTATGACAACCAGGGCCTCCA	248
129_Danko_non-APR	CGGCGGTGCTGCAGCGGAAGAACCAGGACAAGGAGAGCAACTACTGCAAGTATGACAACCAGGGCCTCCA	248
37_PHR_non-APR	CGGCGGTGCTGCAGCGGAAGAACCAGGACAAGGAGAGCAACTACTGCAAGTATGACAACCAGGGCCTCCA	248
52_PHR_non-APR	CGGCGGTGCTGCACCGGAAGAACCAGGACAAGGAGAGCAACTACTGCAAGTACGACAACCAGGGCCTGCA	248
88_PHR_non-APR	CGGCGGTGCTGCAGCGGAAGAACCAGGACAAGGAGAGCAACTACTGCAAGTATGACAACCAGGGCCTCCA	248
101_PHR_non-APR	CGGCGGTGCTGCAGCGGAAGAACCAGGACAAGGAGAGCAACTACTGCAAGTATGACAACCAGGGCCTCCA	248
105_PHR_non-APR	CGGCGGTGCTGCAGCGGAAGAACCAGGACAAGGAGAGCAACTACTGCAAGTATGACAACCAGGGCCTCCA	248
150_PHR_non-APR	CGGCGGTGCTGCAGCGGAAGAACCAGGACAAGGAGAGCAACTACTGCAAGTATGACAACCAGGGCCTCCA	269
150_PHR_non-APR2	CGGCGGTGCTGCAGCGGAAGAACCAGGACAAGGAGAGCAACTACTGCAAGTATGACAACCAGGGCCTCCA	251

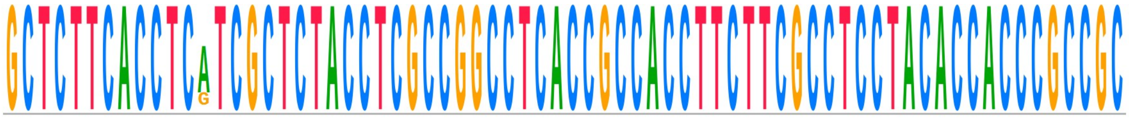

**Consensus**	GCTCTTCACCTCATCGCTCTACCTCGCCGGCCTCACCGCCACCTTCTTCGCCTCCTACACCACCCGCCGC	
	290 300 310 320 330 340 350	
Lr67(sus)	GCTCTTCACCTCGTCGCTCTACCTCGCCGGCCTCACCGCCACCTTCTTCGCCTCCTACACCACCCGCCGC	318
Lr67(res)	GCTCTTCACCTCGTCGCTCTACCTCGCCGGCCTCACCGCCACCTTCTTCGCCTCCTACACCACCCGCCGC	318
ScLr67_1 (Lo7)	GCTCTTCACCTCGTCGCTCTACCTCGCCGGCCTCACCGCCACCTTCTTCGCCTCCTACACCACCCGCCGC	318
118_Danko_APR	GCTCTTCACCTCATCGCTCTACCTCGCCGGCCTCACCGCCACCTTCTTCGCCTCCTACACCACCCGCCGC	318
119_Danko_APR	GCTCTTCACCTCATCGCTCTACCTCGCCGGCCTCACCGCCACCTTCTTCGCCTCCTACACCACCCGCCGC	318
119_Danko_APR2	GCTCTTCACCTCATCGCTCTACCTCGCCGGCCTCACCGCCACCTTCTTCGCCTCCTACACCACCCGCCGC	318
120_Danko_APR	GCTCTTCACCTCATCGCTCTACCTCGCCGGCCTCACCGCCACCTTCTTCGCCTCCTACACCACCCGCCGC	318
138_Danko_APR	GCTCTTCACCTCATCGCTCTACCTCGCCGGCCTCACCGCCACCTTCTTCGCCTCCTACACCACCCGCCGC	318
153_Danko_APR	GCTCTTCACCTCATCGCTCTACCTCGCCGGCCTCACCGCCACCTTCTTCGCCTCCTACACCACCCGCCGC	318
157_Danko_APR	GCTCTTCACCTCATCGCTCTACCTCGCCGGCCTCACCGCCACCTTCTTCGCCTCCTACACCACCCGCCGC	318
160_Danko_APR	GCTCTTCACCTCATCGCTCTACCTCGCCGGCCTCACCGCCACCTTCTTCGCCTCCTACACCACCCGCCGC	318
71_PHR_APR	GCTCTTCACCTCATCGCTCTACCTCGCCGGCCTCACCGCCACCTTCTTCGCCTCCTACACCACCCGCCGC	318
149_PHR_APR	GCTCTTCACCTCATCGCTCTACCTCGCCGGCCTCACCGCCACCTTCTTCGCCTCCTACACCACCCGCCGC	318
59_Danko_non-APR	GCTCTTCACCTCATCGCTCTACCTCGCCGGCCTCACCGCCACCTTCTTCGCCTCCTACACCACCCGCCGC	318
61_Danko_non-APR	GCTCTTCACCTCGTCGCTCTACCTCGCCGGCCTCACCGCCACCTTCTTCGCCTCCTACACCACCCGCCGC	318
123_Danko_non-APR	GCTCTTCACCTCATCGCTCTACCTCGCCGGCCTCACCGCCACCTTCTTCGCCTCCTACACCACCCGCCGC	318
129_Danko_non-APR	GCTCTTCACCTCATCGCTCTACCTCGCCGGCCTCACCGCCACCTTCTTCGCCTCCTACACCACCCGCCGC	318
37_PHR_non-APR	GCTCTTCACCTCATCGCTCTACCTCGCCGGCCTCACCGCCACCTTCTTCGCCTCCTACACCACCCGCCGC	318
52_PHR_non-APR	GCTCTTCACCTCGTCGCTCTACCTCGCCGGCCTCACCGCCACCTTCTTCGCCTCCTACACCACCCGCCGC	318
88_PHR_non-APR	GCTCTTCACCTCATCGCTCTACCTCGCCGGCCTCACCGCCACCTTCTTCGCCTCCTACACCACCCGCCGC	318
101_PHR_non-APR	GCTCTTCACCTCATCGCTCTACCTCGCCGGCCTCACCGCCACCTTCTTCGCCTCCTACACCACCCGCCGC	318
105_PHR_non-APR	GCTCTTCACCTCATCGCTCTACCTCGCCGGCCTCACCGCCACCTTCTTCGCCTCCTACACCACCCGCCGC	318
150_PHR_non-APR	GCTCTTCACCTCATCGCTCTACCTCGCCGGCCTCACCGCCACCTTCTTCGCCTCCTACACCACCCGCCGC	339
150_PHR_non-APR2	GCTCTTCACCTCATCGCTCTACCTCGCCGGCCTCACCGCCACCTTCTTCGCCTCCTACACCACCCGCCGC	321

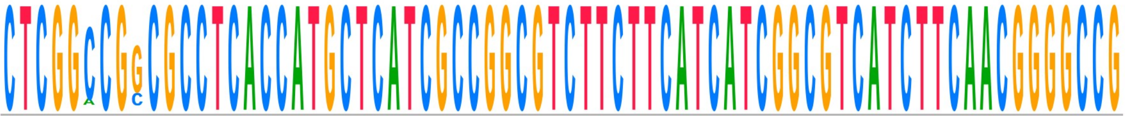

**Consensus**	CTCGGCCGGCGCCTCACCATGCTCATCGCCGGCGTCTTCTTCATCATCGGCGTCATCTTCAACGGGGCCG	
	360 370 380 390 400 410 420	
Lr67(sus)	CTCGGACGCCGCCTCACCATGCTCATCGCCGGCGTCTTCTTCATCATCGGCGTCATCTTCAACGGGGCCG	388
Lr67(res)	CTCGGACGCCGCCTCACCATGCTCATCGCCGGCGTCTTCTTCATCATCGGCGTCATCTTCAACGGGGCCG	388
ScLr67_1 (Lo7)	CTCGGCCGCCGCCTCACCATGCTCATCGCCGGCGTCTTCTTCATCATCGGCGTCATCTTCAACGGGGCCG	388
118_Danko_APR	CTCGGCCGGCGCCTCACCATGCTCATCGCCGGCGTCTTCTTCATCATCGGCGTCATCTTCAACGGGGCCG	388
119_Danko_APR	CTCGGCCGGCGCCTCACCATGCTCATCGCCGGCGTCTTCTTCATCATCGGCGTCATCTTCAACGGGGCCG	388
119_Danko_APR2	CTCGGCCGGCGCCTCACCATGCTCATCGCCGGCGTCTTCTTCATCATCGGCGTCATCTTCAACGGGGCCG	388
120_Danko_APR	CTCGGCCGGCGCCTCACCATGCTCATCGCCGGCGTCTTCTTCATCATCGGCGTCATCTTCAACGGGGCCG	388
138_Danko_APR	CTCGGCCGGCGCCTCACCATGCTCATCGCCGGCGTCTTCTTCATCATCGGCGTCATCTTCAACGGGGCCG	388
153_Danko_APR	CTCGGCCGGCGCCTCACCATGCTCATCGCCGGCGTCTTCTTCATCATCGGCGTCATCTTCAACGGGGCCG	388
157_Danko_APR	CTCGGCCGGCGCCTCACCATGCTCATCGCCGGCGTCTTCTTCATCATCGGCGTCATCTTCAACGGGGCCG	388
160_Danko_APR	CTCGGCCGGCGCCTCACCATGCTCATCGCCGGCGTCTTCTTCATCATCGGCGTCATCTTCAACGGGGCCG	388
71_PHR_APR	CTCGGCCGGCGCCTCACCATGCTCATCGCCGGCGTCTTCTTCATCATCGGCGTCATCTTCAACGGGGCCG	388
149_PHR_APR	CTCGGCCGGCGCCTCACCATGCTCATCGCCGGCGTCTTCTTCATCATCGGCGTCATCTTCAACGGGGCCG	388
59_Danko_non-APR	CTCGGCCGGCGCCTCACCATGCTCATCGCCGGCGTCTTCTTCATCATCGGCGTCATCTTCAACGGGGCCG	388
61_Danko_non-APR	CTCGGCCGCCGCCTCACCATGCTCATCGCCGGCGTCTTCTTCATCATCGGCGTCATCTTCAACGGGGCCG	388
123_Danko_non-APR	CTCGGCCGGCGCCTCACCATGCTCATCGCCGGCGTCTTCTTCATCATCGGCGTCATCTTCAACGGGGCCG	388
129_Danko_non-APR	CTCGGCCGGCGCCTCACCATGCTCATCGCCGGCGTCTTCTTCATCATCGGCGTCATCTTCAACGGGGCCG	388
37_PHR_non-APR	CTCGGCCGGCGCCTCACCATGCTCATCGCCGGCGTCTTCTTCATCATCGGCGTCATCTTCAACGGGGCCG	388
52_PHR_non-APR	CTCGGCCGCCGCCTCACCATGCTCATCGCCGGCGTCTTCTTCATCATCGGCGTCATCTTCAACGGGGCCG	388
88_PHR_non-APR	CTCGGCCGGCGCCTCACCATGCTCATCGCCGGCGTCTTCTTCATCATCGGCGTCATCTTCAACGGGGCCG	388
101_PHR_non-APR	CTCGGCCGGCGCCTCACCATGCTCATCGCCGGCGTCTTCTTCATCATCGGCGTCATCTTCAACGGGGCCG	388
105_PHR_non-APR	CTCGGCCGGCGCCTCACCATGCTCATCGCCGGCGTCTTCTTCATCATCGGCGTCATCTTCAACGGGGCCG	388
150_PHR_non-APR	CTCGGCCGGCGCCTCACCATGCTCATCGCCGGCGTCTTCTTCATCATCGGCGTCATCTTCAACGGGGCCG	409
150_PHR_non-APR2	CTCGGCCGGCGCCTCACCATGCTCATCGCCGGCGTCTTCTTCATCATCGGCGTCATCTTCAACGGGGCCG	391

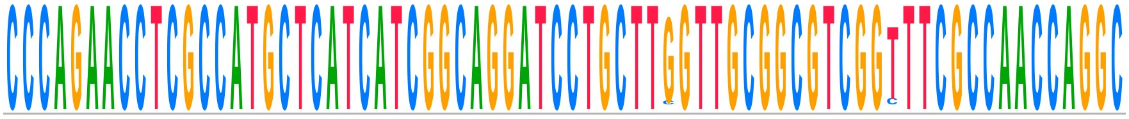

**Consensus**	CCCAGAACCTCGCCATGCTCATCATCGGCAGGATCCTGCTTGGTTGCGGCGTCGGTTTCGCCAACCAGGC	
	430 440 450 460 470 480 490	
Lr67(sus)	CCCAGAACCTCGCCATGCTCATCATCGGCAGGATCCTGCTTGGTTGCGGCGTCGGCTTCGCCAACCAGGC	458
Lr67(res)	CCCAGAACCTCGCCATGCTCATCATCGGCAGGATCCTGCTTCGTTGCGGCGTCGGCTTCGCCAACCAGGC	458
ScLr67_1 (Lo7)	CCCAGAACCTCGCCATGCTCATCATCGGCAGGATCCTGCTTGGTTGCGGCGTCGGTTTCGCCAACCAGGC	458
118_Danko_APR	CCCAGAACCTCGCCATGCTCATCATCGGCAGGATCCTGCTTGGTTGCGGCGTCGGTTTCGCCAACCAGGC	458
119_Danko_APR	CCCAGAACCTCGCCATGCTCATCATCGGCAGGATCCTGCTTGGTTGCGGCGTCGGTTTCGCCAACCAGGC	458
119_Danko_APR2	CCCAGAACCTCGCCATGCTCATCATCGGCAGGATCCTGCTTGGTTGCGGCGTCGGTTTCGCCAACCAGGC	458
120_Danko_APR	CCCAGAACCTCGCCATGCTCATCATCGGCAGGATCCTGCTTGGTTGCGGCGTCGGTTTCGCCAACCAGGC	458
138_Danko_APR	CCCAGAACCTCGCCATGCTCATCATCGGCAGGATCCTGCTTGGTTGCGGCGTCGGTTTCGCCAACCAGGC	458
153_Danko_APR	CCCAGAACCTCGCCATGCTCATCATCGGCAGGATCCTGCTTGGTTGCGGCGTCGGTTTCGCCAACCAGGC	458
157_Danko_APR	CCCAGAACCTCGCCATGCTCATCATCGGCAGGATCCTGCTTGGTTGCGGCGTCGGTTTCGCCAACCAGGC	458
160_Danko_APR	CCCAGAACCTCGCCATGCTCATCATCGGCAGGATCCTGCTTGGTTGCGGCGTCGGTTTCGCCAACCAGGC	458
71_PHR_APR	CCCAGAACCTCGCCATGCTCATCATCGGCAGGATCCTGCTTGGTTGCGGCGTCGGTTTCGCCAACCAGGC	458
149_PHR_APR	CCCAGAACCTCGCCATGCTCATCATCGGCAGGATCCTGCTTGGTTGCGGCGTCGGTTTCGCCAACCAGGC	458
59_Danko_non-APR	CCCAGAACCTCGCCATGCTCATCATCGGCAGGATCCTGCTTGGTTGCGGCGTCGGTTTCGCCAACCAGGC	458
61_Danko_non-APR	CCCAGAACCTCGCCATGCTCATCATCGGCAGGATCCTGCTTGGTTGCGGCGTCGGTTTCGCCAACCAGGC	458
123_Danko_non-APR	CCCAGAACCTCGCCATGCTCATCATCGGCAGGATCCTGCTTGGTTGCGGCGTCGGTTTCGCCAACCAGGC	458
129_Danko_non-APR	CCCAGAACCTCGCCATGCTCATCATCGGCAGGATCCTGCTTGGTTGCGGCGTCGGTTTCGCCAACCAGGC	458
37_PHR_non-APR	CCCAGAACCTCGCCATGCTCATCATCGGCAGGATCCTGCTTGGTTGCGGCGTCGGTTTCGCCAACCAGGC	458
52_PHR_non-APR	CCCAGAACCTCGCCATGCTCATCATCGGCAGGATCCTGCTTGGTTGCGGCGTCGGTTTCGCCAACCAGGC	458
88_PHR_non-APR	CCCAGAACCTCGCCATGCTCATCATCGGCAGGATCCTGCTTGGTTGCGGCGTCGGTTTCGCCAACCAGGC	458
101_PHR_non-APR	CCCAGAACCTCGCCATGCTCATCATCGGCAGGATCCTGCTTGGTTGCGGCGTCGGTTTCGCCAACCAGGC	458
105_PHR_non-APR	CCCAGAACCTCGCCATGCTCATCATCGGCAGGATCCTGCTTGGTTGCGGCGTCGGTTTCGCCAACCAGGC	458
150_PHR_non-APR	CCCAGAACCTCGCCATGCTCATCATCGGCAGGATCCTGCTTGGTTGCGGCGTCGGTTTCGCCAACCAGGC	479
150_PHR_non-APR2	CCCAGAACCTCGCCATGCTCATCATCGGCAGGATCCTGCTTGGTTGCGGCGTCGGTTTCGCCAACCAGGC	461

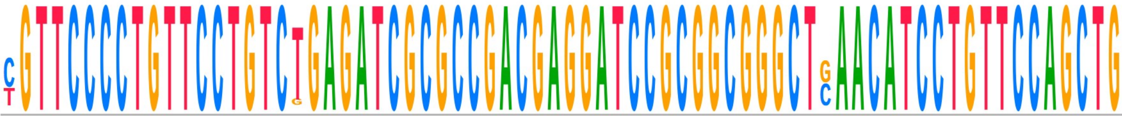

**Consensus**	CGTTCCCCTGTTCCTGTCTGAGATCGCGCCGACGAGGATCCGCGGCGGGCTNAACATCCTGTTCCAGCTG	
	500 510 520 530 540 550 560	
Lr67(sus)	CGTTCCCCTGTTCCTGTCGGAGATCGCGCCGACGAGGATCCGCGGCGGGCTCAACATCCTGTTCCAGCTG	528
Lr67(res)	CGTTCCCCTGTTCCTGTCGGAGATCGCGCCGACGAGGATCCGCGGCGGGCTCAACATCCTGTTCCAGCTG	528
ScLr67_1 (Lo7)	CGTTCCCCTGTTCCTGTCTGAGATCGCGCCGACGAGGATCCGCGGCGGGCTGAACATCCTGTTCCAGCTG	528
118_Danko_APR	CGTTCCCCTGTTCCTGTCTGAGATCGCGCCGACGAGGATCCGCGGCGGGCTGAACATCCTGTTCCAGCTG	528
119_Danko_APR	TGTTCCCCTGTTCCTGTCTGAGATCGCGCCGACGAGGATCCGCGGCGGGCTCAACATCCTGTTCCAGCTG	528
119_Danko_APR2	TGTTCCCCTGTTCCTGTCTGAGATCGCGCCGACGAGGATCCGCGGCGGGCTCAACATCCTGTTCCAGCTG	528
120_Danko_APR	TGTTCCCCTGTTCCTGTCTGAGATCGCGCCGACGAGGATCCGCGGCGGGCTCAACATCCTGTTCCAGCTG	528
138_Danko_APR	TGTTCCCCTGTTCCTGTCTGAGATCGCGCCGACGAGGATCCGCGGCGGGCTCAACATCCTGTTCCAGCTG	528
153_Danko_APR	CGTTCCCCTGTTCCTGTCTGAGATCGCGCCGACGAGGATCCGCGGCGGGCTGAACATCCTGTTCCAGCTG	528
157_Danko_APR	TGTTCCCCTGTTCCTGTCTGAGATCGCGCCGACGAGGATCCGCGGCGGGCTCAACATCCTGTTCCAGCTG	528
160_Danko_APR	TGTTCCCCTGTTCCTGTCTGAGATCGCGCCGACGAGGATCCGCGGCGGGCTCAACATCCTGTTCCAGCTG	528
71_PHR_APR	TGTTCCCCTGTTCCTGTCTGAGATCGCGCCGACGAGGATCCGCGGCGGGCTCAACATCCTGTTCCAGCTG	528
149_PHR_APR	CGTTCCCCTGTTCCTGTCTGAGATCGCGCCGACGAGGATCCGCGGCGGGCTGAACATCCTGTTCCAGCTG	528
59_Danko_non-APR	TGTTCCCCTGTTCCTGTCTGAGATCGCGCCGACGAGGATCCGCGGCGGGCTCAACATCCTGTTCCAGCTG	528
61_Danko_non-APR	CGTTCCCCTGTTCCTGTCTGAGATCGCGCCGACGAGGATCCGCGGCGGGCTGAACATCCTGTTCCAGCTG	528
123_Danko_non-APR	CGTTCCCCTGTTCCTGTCTGAGATCGCGCCGACGAGGATCCGCGGCGGGCTGAACATCCTGTTCCAGCTG	528
129_Danko_non-APR	CGTTCCCCTGTTCCTGTCTGAGATCGCGCCGACGAGGATCCGCGGCGGGCTGAACATCCTGTTCCAGCTG	528
37_PHR_non-APR	TGTTCCCCTGTTCCTGTCTGAGATCGCGCCGACGAGGATCCGCGGCGGGCTCAACATCCTGTTCCAGCTG	528
52_PHR_non-APR	CGTTCCCCTGTTCCTGTCTGAGATCGCGCCGACGAGGATCCGCGGCGGGCTCAACATCCTGTTCCAGCTG	528
88_PHR_non-APR	CGTTCCCCTGTTCCTGTCTGAGATCGCGCCGACGAGGATCCGCGGCGGGCTGAACATCCTGTTCCAGCTG	528
101_PHR_non-APR	CGTTCCCCTGTTCCTGTCTGAGATCGCGCCGACGAGGATCCGCGGCGGGCTGAACATCCTGTTCCAGCTG	528
105_PHR_non-APR	CGTTCCCCTGTTCCTGTCTGAGATCGCGCCGACGAGGATCCGCGGCGGGCTGAACATCCTGTTCCAGCTG	528
150_PHR_non-APR	CGTTCCCCTGTTCCTGTCTGAGATCGCGCCGACGAGGATCCGCGGCGGGCTGAACATCCTGTTCCAGCTG	549
150_PHR_non-APR2	CGTTCCCCTGTTCCTGTCTGAGATCGCGCCGACGAGGATCCGCGGCGGGCTGAACATCCTGTTCCAGCTG	531

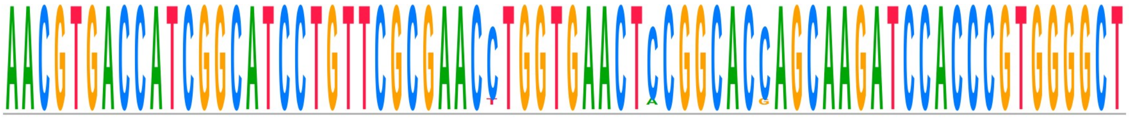

**Consensus**	AACGTGACCATCGGCATCCTGTTCGCGAACCTGGTGAACTCCGGCACCAGCAAGATCCACCCGTGGGGCT	
	570 580 590 600 610 620 630	
Lr67(sus)	AACGTGACCATCGGCATCCTGTTCGCGAACCTGGTGAACTACGGCACGAGCAAGATCCACCCGTGGGGCT	598
Lr67(res)	AACGTGACCATCGGCATCCTGTTCGCGAACCTGGTGAACTACGGCACGAGCAAGATCCACCCGTGGGGCT	598
ScLr67_1 (Lo7)	AACGTGACCATCGGCATCCTGTTCGCGAACTTGGTGAACTCCGGCACCAGCAAGATCCACCCGTGGGGCT	598
118_Danko_APR	AACGTGACCATCGGCATCCTGTTCGCGAACCTGGTGAACTCCGGCACCAGCAAGATCCACCCGTGGGGCT	598
119_Danko_APR	AACGTGACCATCGGCATCCTGTTCGCGAACCTGGTGAACTCCGGCACCAGCAAGATCCACCCGTGGGGCT	598
119_Danko_APR2	AACGTGACCATCGGCATCCTGTTCGCGAACCTGGTGAACTCCGGCACCAGCAAGATCCACCCGTGGGGCT	598
120_Danko_APR	AACGTGACCATCGGCATCCTGTTCGCGAACCTGGTGAACTCCGGCACCAGCAAGATCCACCCGTGGGGCT	598
138_Danko_APR	AACGTGACCATCGGCATCCTGTTCGCGAACCTGGTGAACTCCGGCACCAGCAAGATCCACCCGTGGGGCT	598
153_Danko_APR	AACGTGACCATCGGCATCCTGTTCGCGAACCTGGTGAACTCCGGCACCAGCAAGATCCACCCGTGGGGCT	598
157_Danko_APR	AACGTGACCATCGGCATCCTGTTCGCGAACCTGGTGAACTCCGGCACCAGCAAGATCCACCCGTGGGGCT	598
160_Danko_APR	AACGTGACCATCGGCATCCTGTTCGCGAACCTGGTGAACTCCGGCACCAGCAAGATCCACCCGTGGGGCT	598
71_PHR_APR	AACGTGACCATCGGCATCCTGTTCGCGAACCTGGTGAACTCCGGCACCAGCAAGATCCACCCGTGGGGCT	598
149_PHR_APR	AACGTGACCATCGGCATCCTGTTCGCGAACCTGGTGAACTCCGGCACCAGCAAGATCCACCCGTGGGGCT	598
59_Danko_non-APR	AACGTGACCATCGGCATCCTGTTCGCGAACCTGGTGAACTCCGGCACCAGCAAGATCCACCCGTGGGGCT	598
61_Danko_non-APR	AACGTGACCATCGGCATCCTGTTCGCGAACTTGGTGAACTCCGGCACCAGCAAGATCCACCCGTGGGGCT	598
123_Danko_non-APR	AACGTGACCATCGGCATCCTGTTCGCGAACCTGGTGAACTCCGGCACCAGCAAGATCCACCCGTGGGGCT	598
129_Danko_non-APR	AACGTGACCATCGGCATCCTGTTCGCGAACCTGGTGAACTCCGGCACCAGCAAGATCCACCCGTGGGGCT	598
37_PHR_non-APR	AACGTGACCATCGGCATCCTGTTCGCGAACCTGGTGAACTCCGGCACCAGCAAGATCCACCCGTGGGGCT	598
52_PHR_non-APR	AACGTGACCATCGGCATCCTGTTCGCGAACCTGGTGAACTCCGGCACCAGCAAGATCCACCCGTGGGGCT	598
88_PHR_non-APR	AACGTGACCATCGGCATCCTGTTCGCGAACCTGGTGAACTCCGGCACCAGCAAGATCCACCCGTGGGGCT	598
101_PHR_non-APR	AACGTGACCATCGGCATCCTGTTCGCGAACCTGGTGAACTCCGGCACCAGCAAGATCCACCCGTGGGGCT	598
105_PHR_non-APR	AACGTGACCATCGGCATCCTGTTCGCGAACCTGGTGAACTCCGGCACCAGCAAGATCCACCCGTGGGGCT	598
150_PHR_non-APR	AACGTGACCATCGGCATCCTGTTCGCGAACCTGGTGAACTCCGGCACCAGCAAGATCCACCCGTGGGGCT	619
150_PHR_non-APR2	AACGTGACCATCGGCATCCTGTTCGCGAACCTGGTGAACTCCGGCACCAGCAAGATCCACCCGTGGGGCT	601

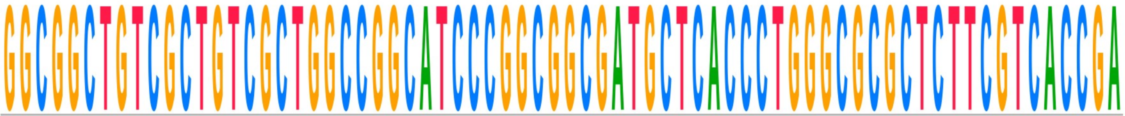

**Consensus**	GGCGGCTGTCGCTGTCGCTGGCCGGCATCCCGGCGGCGATGCTCACCCTGGGCGCGCTCTTCGTCACCGA	
	640 650 660 670 680 690 700	
Lr67(sus)	GGCGGCTGTCGCTGTCGCTGGCCGGCATCCCGGCGGCGATGCTCACCCTGGGCGCGCTCTTCGTCACCGA	668
Lr67(res)	GGCGGCTGTCGCTGTCGCTGGCCGGCATCCCGGCGGCGATGCTCACCCTGGGCGCGCTCTTCGTCACCGA	668
ScLr67_1 (Lo7)	GGCGGCTGTCGCTGTCGCTGGCCGGCATCCCGGCGGCGATGCTCACCCTGGGCGCGCTCTTCGTCACCGA	668
118_Danko_APR	GGCGGCTGTCGCTGTCGCTGGCCGGCATCCCGGCGGCGATGCTCACCCTGGGCGCGCTCTTCGTCACCGA	668
119_Danko_APR	GGCGGCTGTCGCTGTCGCTGGCCGGCATCCCGGCGGCGATGCTCACCCTGGGCGCGCTCTTCGTCACCGA	668
119_Danko_APR2	GGCGGCTGTCGCTGTCGCTGGCCGGCATCCCGGCGGCGATGCTCACCCTGGGCGCGCTCTTCGTCACCGA	668
120_Danko_APR	GGCGGCTGTCGCTGTCGCTGGCCGGCATCCCGGCGGCGATGCTCACCCTGGGCGCGCTCTTCGTCACCGA	668
138_Danko_APR	GGCGGCTGTCGCTGTCGCTGGCCGGCATCCCGGCGGCGATGCTCACCCTGGGCGCGCTCTTCGTCACCGA	668
153_Danko_APR	GGCGGCTGTCGCTGTCGCTGGCCGGCATCCCGGCGGCGATGCTCACCCTGGGCGCGCTCTTCGTCACCGA	668
157_Danko_APR	GGCGGCTGTCGCTGTCGCTGGCCGGCATCCCGGCGGCGATGCTCACCCTGGGCGCGCTCTTCGTCACCGA	668
160_Danko_APR	GGCGGCTGTCGCTGTCGCTGGCCGGCATCCCGGCGGCGATGCTCACCCTGGGCGCGCTCTTCGTCACCGA	668
71_PHR_APR	GGCGGCTGTCGCTGTCGCTGGCCGGCATCCCGGCGGCGATGCTCACCCTGGGCGCGCTCTTCGTCACCGA	668
149_PHR_APR	GGCGGCTGTCGCTGTCGCTGGCCGGCATCCCGGCGGCGATGCTCACCCTGGGCGCGCTCTTCGTCACCGA	668
59_Danko_non-APR	GGCGGCTGTCGCTGTCGCTGGCCGGCATCCCGGCGGCGATGCTCACCCTGGGCGCGCTCTTCGTCACCGA	668
61_Danko_non-APR	GGCGGCTGTCGCTGTCGCTGGCCGGCATCCCGGCGGCGATGCTCACCCTGGGCGCGCTCTTCGTCACCGA	668
123_Danko_non-APR	GGCGGCTGTCGCTGTCGCTGGCCGGCATCCCGGCGGCGATGCTCACCCTGGGCGCGCTCTTCGTCACCGA	668
129_Danko_non-APR	GGCGGCTGTCGCTGTCGCTGGCCGGCATCCCGGCGGCGATGCTCACCCTGGGCGCGCTCTTCGTCACCGA	668
37_PHR_non-APR	GGCGGCTGTCGCTGTCGCTGGCCGGCATCCCGGCGGCGATGCTCACCCTGGGCGCGCTCTTCGTCACCGA	668
52_PHR_non-APR	GGCGGCTGTCGCTGTCGCTGGCCGGCATCCCGGCGGCGATGCTCACCCTGGGCGCGCTCTTCGTCACCGA	668
88_PHR_non-APR	GGCGGCTGTCGCTGTCGCTGGCCGGCATCCCGGCGGCGATGCTCACCCTGGGCGCGCTCTTCGTCACCGA	668
101_PHR_non-APR	GGCGGCTGTCGCTGTCGCTGGCCGGCATCCCGGCGGCGATGCTCACCCTGGGCGCGCTCTTCGTCACCGA	668
105_PHR_non-APR	GGCGGCTGTCGCTGTCGCTGGCCGGCATCCCGGCGGCGATGCTCACCCTGGGCGCGCTCTTCGTCACCGA	668
150_PHR_non-APR	GGCGGCTGTCGCTGTCGCTGGCCGGCATCCCGGCGGCGATGCTCACCCTGGGCGCGCTCTTCGTCACCGA	689
150_PHR_non-APR2	GGCGGCTGTCGCTGTCGCTGGCCGGCATCCCGGCGGCGATGCTCACCCTGGGCGCGCTCTTCGTCACCGA	671

```
                     CACCCCCAACAGCCTCATCGAGCGCGGCCACCTGGAGGAGGGCAAGGCGGTGCTGAAGCGGATCCGCGGC
Consensus            CACCCCCAACAGCCTCATCGAGCGCGGCCACCTGGAG---------------------------------
                              710       720       730       740       750       760       770
Lr67(sus)            CACCCCCAACAGCCTCATCGAGCGCGGCCACCTGGAG---------------------------------  705
Lr67(res)            CACCCCCAACAGCCTCATCGAGCGCGGCCACCTGGAG---------------------------------  705
ScLr67_1 (Lo7)       CACCCCCAACAGCCTCATCGAGCGCGGCCACCTGGAG---------------------------------  705
118_Danko_APR        CACCCCCAACAGCCTCATCGAGCGCGGCCACCTGGAG---------------------------------  705
119_Danko_APR        CACCCCCAACAGCCTCATCGAGCGCGGCCACCTGGAGGAGGGCAAGGCGGTGCTGAAGCGGATCCGCGGC  738
119_Danko_APR2       CACCCCCAACAGCCTCATCGAGCGCGGCCACCTGGAGGAGGGCAAGGCGGTGCTGAAGCGGATCCGCGGC  738
120_Danko_APR        CACCCCCAACAGCCTCATCGAGCGCGGCCACCTGGAG---------------------------------  705
138_Danko_APR        CACCCCCAACAGCCTCATCGAGCGCGGCCACCTGGAG---------------------------------  705
153_Danko_APR        CACCCCCAACAGCCTCATCGAGCGCGGCCACCTGGAG---------------------------------  705
157_Danko_APR        CACCCCCAACAGCCTCATCGAGCGCGGCCACCTGGAG---------------------------------  705
160_Danko_APR        CACCCCCAACAGCCTCATCGAGCGCGGCCACCTGGAG---------------------------------  705
71_PHR_APR           CACCCCCAACAGCCTCATCGAGCGCGGCCACCTGGAG---------------------------------  705
149_PHR_APR          CACCCCCAACAGCCTCATCGAGCGCGGCCACCTGGAG---------------------------------  705
59_Danko_non-APR     CACCCCCAACAGCCTCATCGAGCGCGGCCACCTGGAG---------------------------------  705
61_Danko_non-APR     CACCCCCAACAGCCTCATCGAGCGCGGCCACCTGGAG---------------------------------  705
123_Danko_non-APR    CACCCCCAACAGCCTCATCGAGCGCGGCCACCTGGAG---------------------------------  705
129_Danko_non-APR    CACCCCCAACAGCCTCATCGAGCGCGGCCACCTGGAG---------------------------------  705
37_PHR_non-APR       CACCCCCAACAGCCTCATCGAGCGCGGCCACCTGGAG---------------------------------  705
52_PHR_non-APR       CACCCCCAACAGCCTCATCGAGCGCGGCCACCTGGAG---------------------------------  705
88_PHR_non-APR       CACCCCCAACAGCCTCATCGAGCGCGGCCACCTGGAG---------------------------------  705
101_PHR_non-APR      CACCCCCAACAGCCTCATCGAGCGCGGCCACCTGGAG---------------------------------  705
105_PHR_non-APR      CACCCCCAACAGCCTCATCGAGCGCGGCCACCTGGAG---------------------------------  705
150_PHR_non-APR      CACCCCCAACAGCCTCATCGAGCGCGGCCACCTGGAG---------------------------------  726
150_PHR_non-APR2     CACCCCCAACAGCCTCATCGAGCGCGGCCACCTGGAG---------------------------------  708
```

```
                     ACCGACAAC T C
Consensus            ----------------------------------------------------------------------
                              780       790       800       810       820       830       840
Lr67(sus)            ----------------------------------------------------------------------  705
Lr67(res)            ----------------------------------------------------------------------  705
ScLr67_1 (Lo7)       ----------------------------------------------------------------------  705
118_Danko_APR        ----------------------------------------------------------------------  705
119_Danko_APR        ACCGACAACGTGC---------------------------------------------------------  751
119_Danko_APR2       ACCGACAACATCCACCCGTGGGGCTGGCGGCTGTCGCTGTCGCTGGCCGGCATCCCGGCGGCGATGCTCA  808
120_Danko_APR        ----------------------------------------------------------------------  705
138_Danko_APR        ----------------------------------------------------------------------  705
153_Danko_APR        ----------------------------------------------------------------------  705
157_Danko_APR        ----------------------------------------------------------------------  705
160_Danko_APR        ----------------------------------------------------------------------  705
71_PHR_APR           ----------------------------------------------------------------------  705
149_PHR_APR          ----------------------------------------------------------------------  705
59_Danko_non-APR     ----------------------------------------------------------------------  705
61_Danko_non-APR     ----------------------------------------------------------------------  705
123_Danko_non-APR    ----------------------------------------------------------------------  705
129_Danko_non-APR    ----------------------------------------------------------------------  705
37_PHR_non-APR       ----------------------------------------------------------------------  705
52_PHR_non-APR       ----------------------------------------------------------------------  705
88_PHR_non-APR       ----------------------------------------------------------------------  705
101_PHR_non-APR      ----------------------------------------------------------------------  705
105_PHR_non-APR      ----------------------------------------------------------------------  705
150_PHR_non-APR      ----------------------------------------------------------------------  726
150_PHR_non-APR2     ----------------------------------------------------------------------  708
```

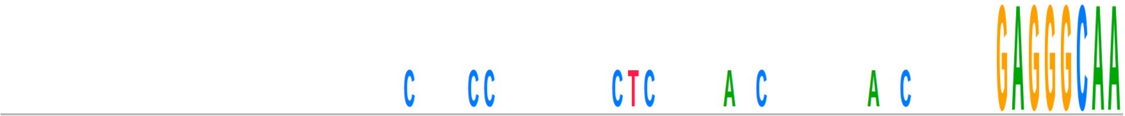

```
Consensus            --------------------------------------------------------------GAGGGCAA
                         850       860       870       880       890       900       910
Lr67(sus)            --------------------------------------------------------------GAGGGCAA   713
Lr67(res)            --------------------------------------------------------------GAGGGCAA   713
ScLr67_1 (Lo7)       --------------------------------------------------------------GAGGGCAA   713
118_Danko_APR        --------------------------------------------------------------GAGGGCAA   713
119_Danko_APR        ------------------CTGTTCGCGAACCTGGTGAACTCCGGCACCAGCAAGATCCACCCGAGGGCAA   803
119_Danko_APR2       CCCTGGGCGCGCTCTTCGTCACCGACACCCCCAACAGCCTCATCGAGCGCGGCCACCTGGAGGAGGGCAA   878
120_Danko_APR        --------------------------------------------------------------GAGGGCAA   713
138_Danko_APR        --------------------------------------------------------------GAGGGCAA   713
153_Danko_APR        --------------------------------------------------------------GAGGGCAA   713
157_Danko_APR        --------------------------------------------------------------GAGGGCAA   713
160_Danko_APR        --------------------------------------------------------------GAGGGCAA   713
71_PHR_APR           --------------------------------------------------------------GAGGGCAA   713
149_PHR_APR          --------------------------------------------------------------GAGGGCAA   713
59_Danko_non-APR     --------------------------------------------------------------GAGGGCAA   713
61_Danko_non-APR     --------------------------------------------------------------GAGGGCAA   713
123_Danko_non-APR    --------------------------------------------------------------GAGGGCAA   713
129_Danko_non-APR    --------------------------------------------------------------GAGGGCAA   713
37_PHR_non-APR       --------------------------------------------------------------GAGGGCAA   713
52_PHR_non-APR       --------------------------------------------------------------GAGGGCAA   713
88_PHR_non-APR       --------------------------------------------------------------GAGGGCAA   713
101_PHR_non-APR      --------------------------------------------------------------GAGGGCAA   713
105_PHR_non-APR      --------------------------------------------------------------GAGGGCAA   713
150_PHR_non-APR      --------------------------------------------------------------GAGGGCAA   734
150_PHR_non-APR2     --------------------------------------------------------------GAGGGCAA   716
```

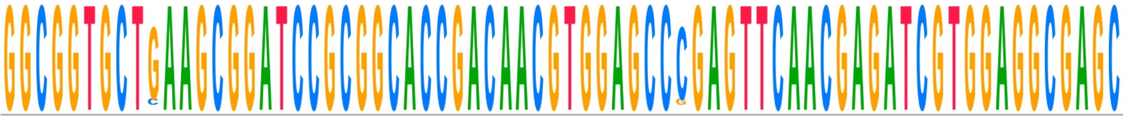

```
Consensus            GGCGGTGCTGAAGCGGATCCGCGGCACCGACAACGTGGAGCCCGAGTTCAACGAGATCGTGGAGGCGAGC
                             920       930       940       950       960       970       980
Lr67(sus)            GGCGGTGCTCAAGCGGATCCGCGGCACCGACAACGTGGAGCCGGAGTTCAACGAGATCGTGGAGGCGAGC   783
Lr67(res)            GGCGGTGCTCAAGCGGATCCGCGGCACCGACAACGTGGAGCCGGAGTTCAACGAGATCGTGGAGGCGAGC   783
ScLr67_1 (Lo7)       GGCGGTGCTGAAGCGGATCCGCGGCACCGACAACGTGGAGCCCGAGTTCAACGAGATCGTGGAGGCGAGC   783
118_Danko_APR        GGCGGTGCTGAAGCGGATCCGCGGCACCGACAACGTGGAGCCCGAGTTCAACGAGATCGTGGAGGCGAGC   783
119_Danko_APR        GGCGGTGCTGAAGCGGATCCGCGGCACCGACAACGTGGAGCCCGAGTTCAACGAGATCGTGGAGGCGAGC   873
119_Danko_APR2       GGCGGTGCTGAAGCGGATCCGCGGCACCGACAACGTGGAGCCCGAGTTCAACGAGATCGTGGAGGCGAGC   948
120_Danko_APR        GGCGGTGCTGAAGCGGATCCGCGGCACCGACAACGTGGAGCCCGAGTTCAACGAGATCGTGGAGGCGAGC   783
138_Danko_APR        GGCGGTGCTGAAGCGGATCCGCGGCACCGACAACGTGGAGCCCGAGTTCAACGAGATCGTGGAGGCGAGC   783
153_Danko_APR        GGCGGTGCTGAAGCGGATCCGCGGCACCGACAACGTGGAGCCCGAGTTCAACGAGATCGTGGAGGCGAGC   783
157_Danko_APR        GGCGGTGCTGAAGCGGATCCGCGGCACCGACAACGTGGAGCCCGAGTTCAACGAGATCGTGGAGGCGAGC   783
160_Danko_APR        GGCGGTGCTGAAGCGGATCCGCGGCACCGACAACGTGGAGCCCGAGTTCAACGAGATCGTGGAGGCGAGC   783
71_PHR_APR           GGCGGTGCTGAAGCGGATCCGCGGCACCGACAACGTGGAGCCCGAGTTCAACGAGATCGTGGAGGCGAGC   783
149_PHR_APR          GGCGGTGCTGAAGCGGATCCGCGGCACCGACAACGTGGAGCCCGAGTTCAACGAGATCGTGGAGGCGAGC   783
59_Danko_non-APR     GGCGGTGCTGAAGCGGATCCGCGGCACCGACAACGTGGAGCCCGAGTTCAACGAGATCGTGGAGGCGAGC   783
61_Danko_non-APR     GGCGGTGCTGAAGCGGATCCGCGGCACCGACAACGTGGAGCCCGAGTTCAACGAGATCGTGGAGGCGAGC   783
123_Danko_non-APR    GGCGGTGCTGAAGCGGATCCGCGGCACCGACAACGTGGAGCCCGAGTTCAACGAGATCGTGGAGGCGAGC   783
129_Danko_non-APR    GGCGGTGCTGAAGCGGATCCGCGGCACCGACAACGTGGAGCCCGAGTTCAACGAGATCGTGGAGGCGAGC   783
37_PHR_non-APR       GGCGGTGCTGAAGCGGATCCGCGGCACCGACAACGTGGAGCCCGAGTTCAACGAGATCGTGGAGGCGAGC   783
52_PHR_non-APR       GGCGGTGCTGAAGCGGATCCGCGGCACCGACAACGTGGAGCCCGAGTTCAACGAGATCGTGGAGGCGAGC   783
88_PHR_non-APR       GGCGGTGCTGAAGCGGATCCGCGGCACCGACAACGTGGAGCCCGAGTTCAACGAGATCGTGGAGGCGAGC   783
101_PHR_non-APR      GGCGGTGCTGAAGCGGATCCGCGGCACCGACAACGTGGAGCCCGAGTTCAACGAGATCGTGGAGGCGAGC   783
105_PHR_non-APR      GGCGGTGCTGAAGCGGATCCGCGGCACCGACAACGTGGAGCCCGAGTTCAACGAGATCGTGGAGGCGAGC   783
150_PHR_non-APR      GGCGGTGCTGAAGCGGATCCGCGGCACCGACAACGTGGAGCCCGAGTTCAACGAGATCGTGGAGGCGAGC   804
150_PHR_non-APR2     GGCGGTGCTGAAGCGGATCCGCGGCACCGACAACGTGGAGCCCGAGTTCAACGAGATCGTGGAGGCGAGC   786
```

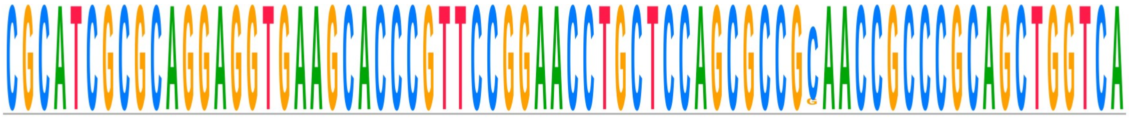

Sequence	990–1050	
**Consensus**	CGCATCGCGCAGGAGGTGAAGCACCCGTTCCGGAACCTGCTCCAGCGCCGCAACCGCCCGCAGCTGGTCA	
Lr67(sus)	CGCATCGCGCAGGAGGTGAAGCACCCGTTCCGGAACCTGCTCCAGCGCCGGAACCGCCCGCAGCTGGTCA	853
Lr67(res)	CGCATCGCGCAGGAGGTGAAGCACCCGTTCCGGAACCTGCTCCAGCGCCGGAACCGCCCGCAGCTGGTCA	853
ScLr67_1 (Lo7)	CGCATCGCGCAGGAGGTGAAGCACCCGTTCCGGAACCTGCTCCAGCGCCGCAACCGCCCGCAGCTGGTCA	853
118_Danko_APR	CGCATCGCGCAGGAGGTGAAGCACCCGTTCCGGAACCTGCTCCAGCGCCGCAACCGCCCGCAGCTGGTCA	853
119_Danko_APR	CGCATCGCGCAGGAGGTGAAGCACCCGTTCCGGAACCTGCTCCAGCGCCGCAACCGCCCGCAGCTGGTCA	943
119_Danko_APR2	CGCATCGCGCAGGAGGTGAAGCACCCGTTCCGGAACCTGCTCCAGCGCCGCAACCGCCCGCAGCTGGTCA	1018
120_Danko_APR	CGCATCGCGCAGGAGGTGAAGCACCCGTTCCGGAACCTGCTCCAGCGCCGCAACCGCCCGCAGCTGGTCA	853
138_Danko_APR	CGCATCGCGCAGGAGGTGAAGCACCCGTTCCGGAACCTGCTCCAGCGCCGCAACCGCCCGCAGCTGGTCA	853
153_Danko_APR	CGCATCGCGCAGGAGGTGAAGCACCCGTTCCGGAACCTGCTCCAGCGCCGCAACCGCCCGCAGCTGGTCA	853
157_Danko_APR	CGCATCGCGCAGGAGGTGAAGCACCCGTTCCGGAACCTGCTCCAGCGCCGCAACCGCCCGCAGCTGGTCA	853
160_Danko_APR	CGCATCGCGCAGGAGGTGAAGCACCCGTTCCGGAACCTGCTCCAGCGCCGCAACCGCCCGCAGCTGGTCA	853
71_PHR_APR	CGCATCGCGCAGGAGGTGAAGCACCCGTTCCGGAACCTGCTCCAGCGCCGCAACCGCCCGCAGCTGGTCA	853
149_PHR_APR	CGCATCGCGCAGGAGGTGAAGCACCCGTTCCGGAACCTGCTCCAGCGCCGCAACCGCCCGCAGCTGGTCA	853
59_Danko_non-APR	CGCATCGCGCAGGAGGTGAAGCACCCGTTCCGGAACCTGCTCCAGCGCCGCAACCGCCCGCAGCTGGTCA	853
61_Danko_non-APR	CGCATCGCGCAGGAGGTGAAGCACCCGTTCCGGAACCTGCTCCAGCGCCGCAACCGCCCGCAGCTGGTCA	853
123_Danko_non-APR	CGCATCGCGCAGGAGGTGAAGCACCCGTTCCGGAACCTGCTCCAGCGCCGCAACCGCCCGCAGCTGGTCA	853
129_Danko_non-APR	CGCATCGCGCAGGAGGTGAAGCACCCGTTCCGGAACCTGCTCCAGCGCCGCAACCGCCCGCAGCTGGTCA	853
37_PHR_non-APR	CGCATCGCGCAGGAGGTGAAGCACCCGTTCCGGAACCTGCTCCAGCGCCGCAACCGCCCGCAGCTGGTCA	853
52_PHR_non-APR	CGCATCGCGCAGGAGGTGAAGCACCCGTTCCGGAACCTGCTCCAGCGCCGCAACCGCCCGCAGCTGGTCA	853
88_PHR_non-APR	CGCATCGCGCAGGAGGTGAAGCACCCGTTCCGGAACCTGCTCCAGCGCCGCAACCGCCCGCAGCTGGTCA	853
101_PHR_non-APR	CGCATCGCGCAGGAGGTGAAGCACCCGTTCCGGAACCTGCTCCAGCGCCGCAACCGCCCGCAGCTGGTCA	853
105_PHR_non-APR	CGCATCGCGCAGGAGGTGAAGCACCCGTTCCGGAACCTGCTCCAGCGCCGCAACCGCCCGCAGCTGGTCA	853
150_PHR_non-APR	CGCATCGCGCAGGAGGTGAAGCACCCGTTCCGGAACCTGCTCCAGCGCCGCAACCGCCCGCAGCTGGTCA	874
150_PHR_non-APR2	CGCATCGCGCAGGAGGTGAAGCACCCGTTCCGGAACCTGCTCCAGCGCCGCAACCGCCCGCAGCTGGTCA	856

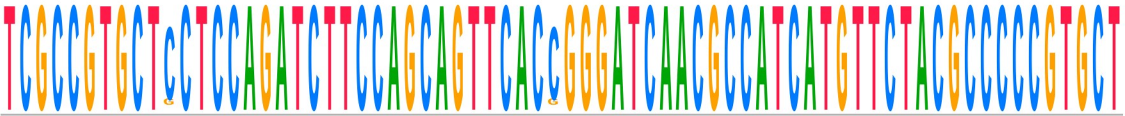

Sequence	1060–1120	
**Consensus**	TCGCCGTGCTCCTCCAGATCTTCCAGCAGTTCACCGGGATCAACGCCATCATGTTCTACGCCCCCGTGCT	
Lr67(sus)	TCGCCGTGCTGCTCCAGATCTTCCAGCAGTTCACGGGGATCAACGCCATCATGTTCTACGCCCCCGTGCT	923
Lr67(res)	TCGCCGTGCTGCTCCAGATCTTCCAGCAGTTCACGGGGATCAACGCCATCATGTTCTACGCCCCCGTGCT	923
ScLr67_1 (Lo7)	TCGCCGTGCTCCTCCAGATCTTCCAGCAGTTCACCGGGATCAACGCCATCATGTTCTACGCCCCCGTGCT	923
118_Danko_APR	TCGCCGTGCTCCTCCAGATCTTCCAGCAGTTCACCGGGATCAACGCCATCATGTTCTACGCCCCCGTGCT	923
119_Danko_APR	TCGCCGTGCTCCTCCAGATCTTCCAGCAGTTCACCGGGATCAACGCCATCATGTTCTACGCCCCCGTGCT	1013
119_Danko_APR2	TCGCCGTGCTCCTCCAGATCTTCCAGCAGTTCACCGGGATCAACGCCATCATGTTCTACGCCCCCGTGCT	1088
120_Danko_APR	TCGCCGTGCTCCTCCAGATCTTCCAGCAGTTCACCGGGATCAACGCCATCATGTTCTACGCCCCCGTGCT	923
138_Danko_APR	TCGCCGTGCTCCTCCAGATCTTCCAGCAGTTCACCGGGATCAACGCCATCATGTTCTACGCCCCCGTGCT	923
153_Danko_APR	TCGCCGTGCTCCTCCAGATCTTCCAGCAGTTCACCGGGATCAACGCCATCATGTTCTACGCCCCCGTGCT	923
157_Danko_APR	TCGCCGTGCTCCTCCAGATCTTCCAGCAGTTCACCGGGATCAACGCCATCATGTTCTACGCCCCCGTGCT	923
160_Danko_APR	TCGCCGTGCTCCTCCAGATCTTCCAGCAGTTCACCGGGATCAACGCCATCATGTTCTACGCCCCCGTGCT	923
71_PHR_APR	TCGCCGTGCTCCTCCAGATCTTCCAGCAGTTCACCGGGATCAACGCCATCATGTTCTACGCCCCCGTGCT	923
149_PHR_APR	TCGCCGTGCTCCTCCAGATCTTCCAGCAGTTCACCGGGATCAACGCCATCATGTTCTACGCCCCCGTGCT	923
59_Danko_non-APR	TCGCCGTGCTCCTCCAGATCTTCCAGCAGTTCACCGGGATCAACGCCATCATGTTCTACGCCCCCGTGCT	923
61_Danko_non-APR	TCGCCGTGCTCCTCCAGATCTTCCAGCAGTTCACCGGGATCAACGCCATCATGTTCTACGCCCCCGTGCT	923
123_Danko_non-APR	TCGCCGTGCTCCTCCAGATCTTCCAGCAGTTCACCGGGATCAACGCCATCATGTTCTACGCCCCCGTGCT	923
129_Danko_non-APR	TCGCCGTGCTCCTCCAGATCTTCCAGCAGTTCACCGGGATCAACGCCATCATGTTCTACGCCCCCGTGCT	923
37_PHR_non-APR	TCGCCGTGCTCCTCCAGATCTTCCAGCAGTTCACCGGGATCAACGCCATCATGTTCTACGCCCCCGTGCT	923
52_PHR_non-APR	TCGCCGTGCTCCTCCAGATCTTCCAGCAGTTCACCGGGATCAACGCCATCATGTTCTACGCCCCCGTGCT	923
88_PHR_non-APR	TCGCCGTGCTCCTCCAGATCTTCCAGCAGTTCACCGGGATCAACGCCATCATGTTCTACGCCCCCGTGCT	923
101_PHR_non-APR	TCGCCGTGCTCCTCCAGATCTTCCAGCAGTTCACCGGGATCAACGCCATCATGTTCTACGCCCCCGTGCT	923
105_PHR_non-APR	TCGCCGTGCTCCTCCAGATCTTCCAGCAGTTCACCGGGATCAACGCCATCATGTTCTACGCCCCCGTGCT	923
150_PHR_non-APR	TCGCCGTGCTCCTCCAGATCTTCCAGCAGTTCACCGGGATCAACGCCATCATGTTCTACGCCCCCGTGCT	944
150_PHR_non-APR2	TCGCCGTGCTCCTCCAGATCTTCCAGCAGTTCACCGGGATCAACGCCATCATGTTCTACGCCCCCGTGCT	926

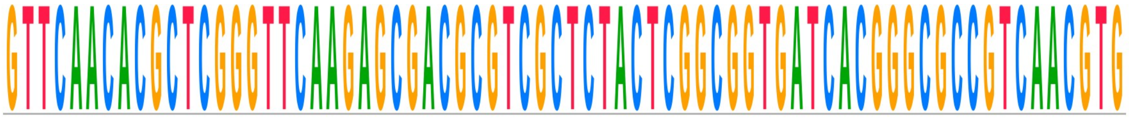

Consensus	GTTCAACACGCTCGGGTTCAAGAGCGACGCGTCGCTCTACTCGGCGGTGATCACGGGCGCCGTCAACGTG	
	1130 1140 1150 1160 1170 1180 1190	
Lr67(sus)	GTTCAACACGCTCGGGTTCAAGAGCGACGCGTCGCTCTACTCGGCGGTGATCACGGGCGCCGTCAACGTG	993
Lr67(res)	GTTCAACACGCTCGGGTTCAAGAGCGACGCGTCGCTCTACTCGGCGGTGATCACGGGCGCCGTCAACGTG	993
ScLr67_1 (Lo7)	GTTCAACACGCTCGGGTTCAAGAGCGACGCGTCGCTCTACTCGGCGGTGATCACGGGCGCCGTCAACGTG	993
118_Danko_APR	GTTCAACACGCTCGGGTTCAAGAGCGACGCGTCGCTCTACTCGGCGGTGATCACGGGCGCCGTCAACGTG	993
119_Danko_APR	GTTCAACACGCTCGGGTTCAAGAGCGACGCGTCGCTCTACTCGGCGGTGATCACGGGCGCCGTCAACGTG	1083
119_Danko_APR2	GTTCAACACGCTCGGGTTCAAGAGCGACGCGTCGCTCTACTCGGCGGTGATCACGGGCGCCGTCAACGTG	1158
120_Danko_APR	GTTCAACACGCTCGGGTTCAAGAGCGACGCGTCGCTCTACTCGGCGGTGATCACGGGCGCCGTCAACGTG	993
138_Danko_APR	GTTCAACACGCTCGGGTTCAAGAGCGACGCGTCGCTCTACTCGGCGGTGATCACGGGCGCCGTCAACGTG	993
153_Danko_APR	GTTCAACACGCTCGGGTTCAAGAGCGACGCGTCGCTCTACTCGGCGGTGATCACGGGCGCCGTCAACGTG	993
157_Danko_APR	GTTCAACACGCTCGGGTTCAAGAGCGACGCGTCGCTCTACTCGGCGGTGATCACGGGCGCCGTCAACGTG	993
160_Danko_APR	GTTCAACACGCTCGGGTTCAAGAGCGACGCGTCGCTCTACTCGGCGGTGATCACGGGCGCCGTCAACGTG	993
71_PHR_APR	GTTCAACACGCTCGGGTTCAAGAGCGACGCGTCGCTCTACTCGGCGGTGATCACGGGCGCCGTCAACGTG	993
149_PHR_APR	GTTCAACACGCTCGGGTTCAAGAGCGACGCGTCGCTCTACTCGGCGGTGATCACGGGCGCCGTCAACGTG	993
59_Danko_non-APR	GTTCAACACGCTCGGGTTCAAGAGCGACGCGTCGCTCTACTCGGCGGTGATCACGGGCGCCGTCAACGTG	993
61_Danko_non-APR	GTTCAACACGCTCGGGTTCAAGAGCGACGCGTCGCTCTACTCGGCGGTGATCACGGGCGCCGTCAACGTG	993
123_Danko_non-APR	GTTCAACACGCTCGGGTTCAAGAGCGACGCGTCGCTCTACTCGGCGGTGATCACGGGCGCCGTCAACGTG	993
129_Danko_non-APR	GTTCAACACGCTCGGGTTCAAGAGCGACGCGTCGCTCTACTCGGCGGTGATCACGGGCGCCGTCAACGTG	993
37_PHR_non-APR	GTTCAACACGCTCGGGTTCAAGAGCGACGCGTCGCTCTACTCGGCGGTGATCACGGGCGCCGTCAACGTG	993
52_PHR_non-APR	GTTCAACACGCTCGGGTTCAAGAGCGACGCGTCGCTCTACTCGGCGGTGATCACGGGCGCCGTCAACGTG	993
88_PHR_non-APR	GTTCAACACGCTCGGGTTCAAGAGCGACGCGTCGCTCTACTCGGCGGTGATCACGGGCGCCGTCAACGTG	993
101_PHR_non-APR	GTTCAACACGCTCGGGTTCAAGAGCGACGCGTCGCTCTACTCGGCGGTGATCACGGGCGCCGTCAACGTG	993
105_PHR_non-APR	GTTCAACACGCTCGGGTTCAAGAGCGACGCGTCGCTCTACTCGGCGGTGATCACGGGCGCCGTCAACGTG	993
150_PHR_non-APR	GTTCAACACGCTCGGGTTCAAGAGCGACGCGTCGCTCTACTCGGCGGTGATCACGGGCGCCGTCAACGTG	1014
150_PHR_non-APR2	GTTCAACACGCTCGGGTTCAAGAGCGACGCGTCGCTCTACTCGGCGGTGATCACGGGCGCCGTCAACGTG	996

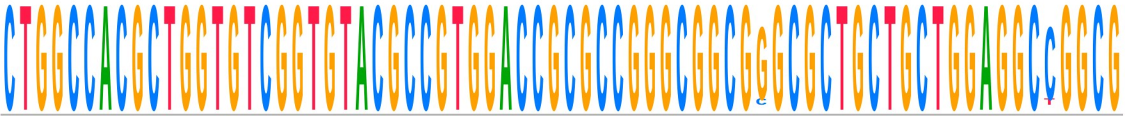

Consensus	CTGGCCACGCTGGTGTCGGTGTACGCCGTGGACCGCGCCGGGCGGCGGGCGCTGCTGCTGGAGGCCGGCG	
	1200 1210 1220 1230 1240 1250 1260	
Lr67(sus)	CTGGCCACGCTGGTGTCGGTGTACGCCGTGGACCGCGCCGGGCGGCGCGCGCTGCTGCTGGAGGCTGGCG	1063
Lr67(res)	CTGGCCACGCTGGTGTCGGTGTACGCCGTGGACCGCGCCGGGCGGCGCGCGCTGCTGCTGGAGGCTGGCG	1063
ScLr67_1 (Lo7)	CTGGCCACGCTGGTGTCGGTGTACGCCGTGGACCGCGCCGGGCGGCGGGCGCTGCTGCTGGAGGCCGGCG	1063
118_Danko_APR	CTGGCCACGCTGGTGTCGGTGTACGCCGTGGACCGCGCCGGGCGGCGGGCGCTGCTGCTGGAGGCCGGCG	1063
119_Danko_APR	CTGGCCACGCTGGTGTCGGTGTACGCCGTGGACCGCGCCGGGCGGCGGGCGCTGCTGCTGGAGGCCGGCG	1153
119_Danko_APR2	CTGGCCACGCTGGTGTCGGTGTACGCCGTGGACCGCGCCGGGCGGCGGGCGCTGCTGCTGGAGGCCGGCG	1228
120_Danko_APR	CTGGCCACGCTGGTGTCGGTGTACGCCGTGGACCGCGCCGGGCGGCGGGCGCTGCTGCTGGAGGCCGGCG	1063
138_Danko_APR	CTGGCCACGCTGGTGTCGGTGTACGCCGTGGACCGCGCCGGGCGGCGGGCGCTGCTGCTGGAGGCCGGCG	1063
153_Danko_APR	CTGGCCACGCTGGTGTCGGTGTACGCCGTGGACCGCGCCGGGCGGCGGGCGCTGCTGCTGGAGGCCGGCG	1063
157_Danko_APR	CTGGCCACGCTGGTGTCGGTGTACGCCGTGGACCGCGCCGGGCGGCGGGCGCTGCTGCTGGAGGCCGGCG	1063
160_Danko_APR	CTGGCCACGCTGGTGTCGGTGTACGCCGTGGACCGCGCCGGGCGGCGGGCGCTGCTGCTGGAGGCCGGCG	1063
71_PHR_APR	CTGGCCACGCTGGTGTCGGTGTACGCCGTGGACCGCGCCGGGCGGCGGGCGCTGCTGCTGGAGGCCGGCG	1063
149_PHR_APR	CTGGCCACGCTGGTGTCGGTGTACGCCGTGGACCGCGCCGGGCGGCGGGCGCTGCTGCTGGAGGCCGGCG	1063
59_Danko_non-APR	CTGGCCACGCTGGTGTCGGTGTACGCCGTGGACCGCGCCGGGCGGCGGGCGCTGCTGCTGGAGGCCGGCG	1063
61_Danko_non-APR	CTGGCCACGCTGGTGTCGGTGTACGCCGTGGACCGCGCCGGGCGGCGGGCGCTGCTGCTGGAGGCCGGCG	1063
123_Danko_non-APR	CTGGCCACGCTGGTGTCGGTGTACGCCGTGGACCGCGCCGGGCGGCGGGCGCTGCTGCTGGAGGCCGGCG	1063
129_Danko_non-APR	CTGGCCACGCTGGTGTCGGTGTACGCCGTGGACCGCGCCGGGCGGCGGGCGCTGCTGCTGGAGGCCGGCG	1063
37_PHR_non-APR	CTGGCCACGCTGGTGTCGGTGTACGCCGTGGACCGCGCCGGGCGGCGGGCGCTGCTGCTGGAGGCCGGCG	1063
52_PHR_non-APR	CTGGCCACGCTGGTGTCGGTGTACGCCGTGGACCGCGCCGGGCGGCGGGCGCTGCTGCTGGAGGCCGGCG	1063
88_PHR_non-APR	CTGGCCACGCTGGTGTCGGTGTACGCCGTGGACCGCGCCGGGCGGCGGGCGCTGCTGCTGGAGGCCGGCG	1063
101_PHR_non-APR	CTGGCCACGCTGGTGTCGGTGTACGCCGTGGACCGCGCCGGGCGGCGGGCGCTGCTGCTGGAGGCCGGCG	1063
105_PHR_non-APR	CTGGCCACGCTGGTGTCGGTGTACGCCGTGGACCGCGCCGGGCGGCGGGCGCTGCTGCTGGAGGCCGGCG	1063
150_PHR_non-APR	CTGGCCACGCTGGTGTCGGTGTACGCCGTGGACCGCGCCGGGCGGCGGGCGCTGCTGCTGGAGGCCGGCG	1084
150_PHR_non-APR2	CTGGCCACGCTGGTGTCGGTGTACGCCGTGGACCGCGCCGGGCGGCGGGCGCTGCTGCTGGAGGCCGGCG	1066

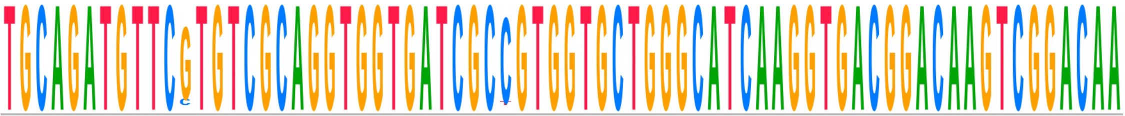

```
Consensus            TGCAGATGTTCGTGTCGCAGGTGGTGATCGCCGTGGTGCTGGGCATCAAGGTGACGGACAAGTCGGACAA
                              1270      1280      1290      1300      1310      1320      1330
Lr67(sus)            TGCAGATGTTCCTGTCGCAGGTGGTGATCGCCGTGGTGCTGGGCATCAAGGTGACGGACAAGTCGGACAA  1133
Lr67(res)            TGCAGATGTTCCTGTCGCAGGTGGTGATCGCCGTGGTGCTGGGCATCAAGGTGACGGACAAGTCGGACAA  1133
ScLr67_1 (Lo7)       TGCAGATGTTCGTGTCGCAGGTGGTGATCGCCGTGGTGCTGGGCATCAAGGTGACGGACAAGTCGGACAA  1133
118_Danko_APR        TGCAGATGTTCGTGTCGCAGGTGGTGATCGCCGTGGTGCTGGGCATCAAGGTGACGGACAAGTCGGACAA  1133
119_Danko_APR        TGCAGATGTTCGTGTCGCAGGTGGTGATCGCCGTGGTGCTGGGCATCAAGGTGACGGACAAGTCGGACAA  1223
119_Danko_APR2       TGCAGATGTTCGTGTCGCAGGTGGTGATCGCCGTGGTGCTGGGCATCAAGGTGACGGACAAGTCGGACAA  1298
120_Danko_APR        TGCAGATGTTCGTGTCGCAGGTGGTGATCGCCGTGGTGCTGGGCATCAAGGTGACGGACAAGTCGGACAA  1133
138_Danko_APR        TGCAGATGTTCGTGTCGCAGGTGGTGATCGCCGTGGTGCTGGGCATCAAGGTGACGGACAAGTCGGACAA  1133
153_Danko_APR        TGCAGATGTTCGTGTCGCAGGTGGTGATCGCCGTGGTGCTGGGCATCAAGGTGACGGACAAGTCGGACAA  1133
157_Danko_APR        TGCAGATGTTCGTGTCGCAGGTGGTGATCGCCGTGGTGCTGGGCATCAAGGTGACGGACAAGTCGGACAA  1133
160_Danko_APR        TGCAGATGTTCGTGTCGCAGGTGGTGATCGCCGTGGTGCTGGGCATCAAGGTGACGGACAAGTCGGACAA  1133
71_PHR_APR           TGCAGATGTTCGTGTCGCAGGTGGTGATCGCCGTGGTGCTGGGCATCAAGGTGACGGACAAGTCGGACAA  1133
149_PHR_APR          TGCAGATGTTCGTGTCGCAGGTGGTGATCGCCGTGGTGCTGGGCATCAAGGTGACGGACAAGTCGGACAA  1133
59_Danko_non-APR     TGCAGATGTTCGTGTCGCAGGTGGTGATCGCTGTGGTGCTGGGCATCAAGGTGACGGACAAGTCGGACAA  1133
61_Danko_non-APR     TGCAGATGTTCGTGTCGCAGGTGGTGATCGCCGTGGTGCTGGGCATCAAGGTGACGGACAAGTCGGACAA  1133
123_Danko_non-APR    TGCAGATGTTCGTGTCGCAGGTGGTGATCGCCGTGGTGCTGGGCATCAAGGTGACGGACAAGTCGGACAA  1133
129_Danko_non-APR    TGCAGATGTTCGTGTCGCAGGTGGTGATCGCCGTGGTGCTGGGCATCAAGGTGACGGACAAGTCGGACAA  1133
37_PHR_non-APR       TGCAGATGTTCGTGTCGCAGGTGGTGATCGCCGTGGTGCTGGGCATCAAGGTGACGGACAAGTCGGACAA  1133
52_PHR_non-APR       TGCAGATGTTCGTGTCGCAGGTGGTGATCGCCGTGGTGCTGGGCATCAAGGTGACGGACAAGTCGGACAA  1133
88_PHR_non-APR       TGCAGATGTTCGTGTCGCAGGTGGTGATCGCCGTGGTGCTGGGCATCAAGGTGACGGACAAGTCGGACAA  1133
101_PHR_non-APR      TGCAGATGTTCGTGTCGCAGGTGGTGATCGCCGTGGTGCTGGGCATCAAGGTGACGGACAAGTCGGACAA  1133
105_PHR_non-APR      TGCAGATGTTCGTGTCGCAGGTGGTGATCGCCGTGGTGCTGGGCATCAAGGTGACGGACAAGTCGGACAA  1133
150_PHR_non-APR      TGCAGATGTTCGTGTCGCAGGTGGTGATCGCCGTGGTGCTGGGCATCAAGGTGACGGACAAGTCGGACAA  1154
150_PHR_non-APR2     TGCAGATGTTCGTGTCGCAGGTGGTGATCGCCGTGGTGCTGGGCATCAAGGTGACGGACAAGTCGGACAA  1136
```

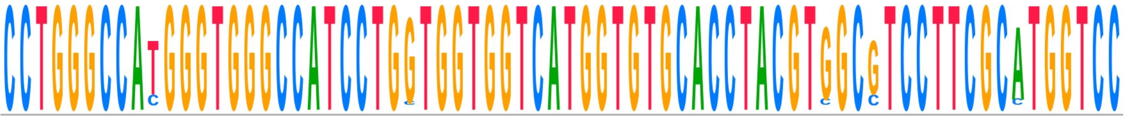

```
Consensus            CCTGGGCCATGGGTGGGCCATCCTGGTGGTGGTCATGGTGTGCACCTACGTGGCGTCCTTCGCATGGTCC
                              1340      1350      1360      1370      1380      1390      1400
Lr67(sus)            CCTGGGCCACGGGTGGGCCATCCTGGTGGTGGTCATGGTGTGCACCTACGTGGCCTCCTTCGCCTGGTCC  1203
Lr67(res)            CCTGGGCCACGGGTGGGCCATCCTGCTGGTGGTCATGGTGTGCACCTACGTGGCCTCCTTCGCCTGGTCC  1203
ScLr67_1 (Lo7)       CCTGGGCCACGGGTGGGCCATCCTGGTGGTGGTCATGGTGTGCACCTACGTCGCCTCCTTCGCATGGTCC  1203
118_Danko_APR        CCTGGGCCATGGGTGGGCCATCCTGGTGGTGGTCATGGTGTGCACCTACGTGGCGTCCTTCGCATGGTCC  1203
119_Danko_APR        CCTGGGCCATGGGTGGGCCATCCTGGTGGTGGTCATGGTGTGCACCTACGTGGCGTCCTTCGCATGGTCC  1293
119_Danko_APR2       CCTGGGCCATGGGTGGGCCATCCTGGTGGTGGTCATGGTGTGCACCTACGTGGCGTCCTTCGCATGGTCC  1368
120_Danko_APR        CCTGGGCCATGGGTGGGCCATCCTGGTGGTGGTCATGGTGTGCACCTACGTGGCGTCCTTCGCATGGTCC  1203
138_Danko_APR        CCTGGGCCATGGGTGGGCCATCCTGGTGGTGGTCATGGTGTGCACCTACGTGGCGTCCTTCGCATGGTCC  1203
153_Danko_APR        CCTGGGCCATGGGTGGGCCATCCTGGTGGTGGTCATGGTGTGCACCTACGTGGCGTCCTTCGCATGGTCC  1203
157_Danko_APR        CCTGGGCCATGGGTGGGCCATCCTGGTGGTGGTCATGGTGTGCACCTACGTGGCGTCCTTCGCATGGTCC  1203
160_Danko_APR        CCTGGGCCATGGGTGGGCCATCCTGGTGGTGGTCATGGTGTGCACCTACGTGGCGTCCTTCGCATGGTCC  1203
71_PHR_APR           CCTGGGCCATGGGTGGGCCATCCTGGTGGTGGTCATGGTGTGCACCTACGTGGCGTCCTTCGCATGGTCC  1203
149_PHR_APR          CCTGGGCCATGGGTGGGCCATCCTGGTGGTGGTCATGGTGTGCACCTACGTGGCGTCCTTCGCATGGTCC  1203
59_Danko_non-APR     CCTGGGCCATGGGTGGGCCATCCTGGTGGTGGTCATGGTGTGCACCTACGTGGCGTCCTTCGCATGGTCC  1203
61_Danko_non-APR     CCTGGGCCACGGGTGGGCCATCCTGGTGGTGGTCATGGTGTGCACCTACGTCGCCTCCTTCGCATGGTCC  1203
123_Danko_non-APR    CCTGGGCCATGGGTGGGCCATCCTGGTGGTGGTCATGGTGTGCACCTACGTGGCGTCCTTCGCATGGTCC  1203
129_Danko_non-APR    CCTGGGCCATGGGTGGGCCATCCTGGTGGTGGTCATGGTGTGCACCTACGTGGCGTCCTTCGCATGGTCC  1203
37_PHR_non-APR       CCTGGGCCATGGGTGGGCCATCCTGGTGGTGGTCATGGTGTGCACCTACGTGGCGTCCTTCGCATGGTCC  1203
52_PHR_non-APR       CCTGGGCCATGGGTGGGCCATCCTGGTGGTGGTCATGGTGTGCACCTACGTGGCGTCCTTCGCATGGTCC  1203
88_PHR_non-APR       CCTGGGCCATGGGTGGGCCATCCTGGTGGTGGTCATGGTGTGCACCTACGTGGCGTCCTTCGCATGGTCC  1203
101_PHR_non-APR      CCTGGGCCATGGGTGGGCCATCCTGGTGGTGGTCATGGTGTGCACCTACGTGGCGTCCTTCGCATGGTCC  1203
105_PHR_non-APR      CCTGGGCCATGGGTGGGCCATCCTGGTGGTGGTCATGGTGTGCACCTACGTGGCGTCCTTCGCATGGTCC  1203
150_PHR_non-APR      CCTGGGCCATGGGTGGGCCATCCTGGTGGTGGTCATGGTGTGCACCTACGTGGCGTCCTTCGCATGGTCC  1224
150_PHR_non-APR2     CCTGGGCCATGGGTGGGCCATCCTGGTGGTGGTCATGGTGTGCACCTACGTGGCGTCCTTCGCATGGTCC  1206
```

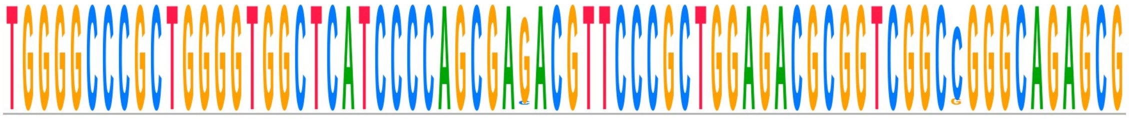

```
Consensus            TGGGGCCCGCTGGGGTGGCTCATCCCCAGCGAGACGTTCCCGCTGGAGACGCGGTCGGCCGGGCAGAGCG
                               1410      1420      1430      1440      1450      1460      1470
Lr67(sus)            TGGGGCCCGCTGGGGTGGCTCATCCCCAGCGAGACGTTCCCGCTGGAGACGCGGTCGGCGGGGCAGAGCG  1273
Lr67(res)            TGGGGCCCGCTGGGGTGGCTCATCCCCAGCGAGACGTTCCCGCTGGAGACGCGGTCGGCGGGGCAGAGCG  1273
ScLr67_1 (Lo7)       TGGGGCCCGCTGGGGTGGCTCATCCCCAGCGAGACGTTCCCGCTGGAGACGCGGTCGGCCGGGCAGAGCG  1273
118_Danko_APR        TGGGGCCCGCTGGGGTGGCTCATCCCCAGCGAGACGTTCCCGCTGGAGACGCGGTCGGCCGGGCAGAGCG  1273
119_Danko_APR        TGGGGCCCGCTGGGGTGGCTCATCCCCAGCGAGACGTTCCCGCTGGAGACGCGGTCGGCCGGGCAGAGCG  1363
119_Danko_APR2       TGGGGCCCGCTGGGGTGGCTCATCCCCAGCGAGACGTTCCCGCTGGAGACGCGGTCGGCCGGGCAGAGCG  1438
120_Danko_APR        TGGGGCCCGCTGGGGTGGCTCATCCCCAGCGAGACGTTCCCGCTGGAGACGCGGTCGGCCGGGCAGAGCG  1273
138_Danko_APR        TGGGGCCCGCTGGGGTGGCTCATCCCCAGCGAGACGTTCCCGCTGGAGACGCGGTCGGCCGGGCAGAGCG  1273
153_Danko_APR        TGGGGCCCGCTGGGGTGGCTCATCCCCAGCGACACGTTCCCGCTGGAGACGCGGTCGGCCGGGCAGAGCG  1273
157_Danko_APR        TGGGGCCCGCTGGGGTGGCTCATCCCCAGCGAGACGTTCCCGCTGGAGACGCGGTCGGCCGGGCAGAGCG  1273
160_Danko_APR        TGGGGCCCGCTGGGGTGGCTCATCCCCAGCGAGACGTTCCCGCTGGAGACGCGGTCGGCCGGGCAGAGCG  1273
71_PHR_APR           TGGGGCCCGCTGGGGTGGCTCATCCCCAGCGAGACGTTCCCGCTGGAGACGCGGTCGGCCGGGCAGAGCG  1273
149_PHR_APR          TGGGGCCCGCTGGGGTGGCTCATCCCCAGCGAGACGTTCCCGCTGGAGACGCGGTCGGCCGGGCAGAGCG  1273
59_Danko_non-APR     TGGGGCCCGCTGGGGTGGCTCATCCCCAGCGAGACGTTCCCGCTGGAGACGCGGTCGGCCGGGCAGAGCG  1273
61_Danko_non-APR     TGGGGCCCGCTGGGGTGGCTCATCCCCAGCGAGACGTTCCCGCTGGAGACGCGGTCGGCCGGGCAGAGCG  1273
123_Danko_non-APR    TGGGGCCCGCTGGGGTGGCTCATCCCCAGCGAGACGTTCCCGCTGGAGACGCGGTCGGCCGGGCAGAGCG  1273
129_Danko_non-APR    TGGGGCCCGCTGGGGTGGCTCATCCCCAGCGAGACGTTCCCGCTGGAGACGCGGTCGGCCGGGCAGAGCG  1273
37_PHR_non-APR       TGGGGCCCGCTGGGGTGGCTCATCCCCAGCGAGACGTTCCCGCTGGAGACGCGGTCGGCCGGGCAGAGCG  1273
52_PHR_non-APR       TGGGGCCCGCTGGGGTGGCTCATCCCCAGCGAGACGTTCCCGCTGGAGACGCGGTCGGCCGGGCAGAGCG  1273
88_PHR_non-APR       TGGGGCCCGCTGGGGTGGCTCATCCCCAGCGAGACGTTCCCGCTGGAGACGCGGTCGGCCGGGCAGAGCG  1273
101_PHR_non-APR      TGGGGCCCGCTGGGGTGGCTCATCCCCAGCGAGACGTTCCCGCTGGAGACGCGGTCGGCCGGGCAGAGCG  1273
105_PHR_non-APR      TGGGGCCCGCTGGGGTGGCTCATCCCCAGCGAGACGTTCCCGCTGGAGACGCGGTCGGCCGGGCAGAGCG  1273
150_PHR_non-APR      TGGGGCCCGCTGGGGTGGCTCATCCCCAGCGAGACGTTCCCGCTGGAGACGCGGTCGGCCGGGCAGAGCG  1294
150_PHR_non-APR2     TGGGGCCCGCTGGGGTGGCTCATCCCCAGCGAGACGTTCCCGCTGGAGACGCGGTCGGCCGGGCAGAGCG  1276
```

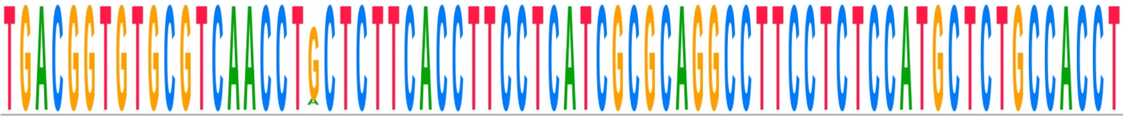

```
Consensus            TGACGGTGTGCGTCAACCTGCTCTTCACCTTCCTCATCGCGCAGGCCTTCCTCTCCATGCTCTGCCACCT
                              1480      1490      1500      1510      1520      1530      1540
Lr67(sus)            TGACGGTGTGCGTCAACCTGCTCTTCACCTTCCTCATCGCGCAGGCCTTCCTCTCCATGCTCTGCCACCT  1343
Lr67(res)            TGACGGTGTGCGTCAACCTGCTCTTCACCTTCCTCATCGCGCAGGCCTTCCTCTCCATGCTCTGCCACCT  1343
ScLr67_1 (Lo7)       TGACGGTGTGCGTCAACCTACTCTTCACCTTCCTCATCGCGCAGGCCTTCCTCTCCATGCTCTGCCACCT  1343
118_Danko_APR        TGACGGTGTGCGTCAACCTGCTCTTCACCTTCCTCATCGCGCAGGCCTTCCTCTCCATGCTCTGCCACCT  1343
119_Danko_APR        TGACGGTGTGCGTCAACCTGCTCTTCACCTTCCTCATCGCGCAGGCCTTCCTCTCCATGCTCTGCCACCT  1433
119_Danko_APR2       TGACGGTGTGCGTCAACCTGCTCTTCACCTTCCTCATCGCGCAGGCCTTCCTCTCCATGCTCTGCCACCT  1508
120_Danko_APR        TGACGGTGTGCGTCAACCTGCTCTTCACCTTCCTCATCGCGCAGGCCTTCCTCTCCATGCTCTGCCACCT  1343
138_Danko_APR        TGACGGTGTGCGTCAACCTGCTCTTCACCTTCCTCATCGCGCAGGCCTTCCTCTCCATGCTCTGCCACCT  1343
153_Danko_APR        TGACGGTGTGCGTCAACCTGCTCTTCACCTTCCTCATCGCGCAGGCCTTCCTCTCCATGCTCTGCCACCT  1343
157_Danko_APR        TGACGGTGTGCGTCAACCTGCTCTTCACCTTCCTCATCGCGCAGGCCTTCCTCTCCATGCTCTGCCACCT  1343
160_Danko_APR        TGACGGTGTGCGTCAACCTGCTCTTCACCTTCCTCATCGCGCAGGCCTTCCTCTCCATGCTCTGCCACCT  1343
71_PHR_APR           TGACGGTGTGCGTCAACCTGCTCTTCACCTTCCTCATCGCGCAGGCCTTCCTCTCCATGCTCTGCCACCT  1343
149_PHR_APR          TGACGGTGTGCGTCAACCTGCTCTTCACCTTCCTCATCGCGCAGGCCTTCCTCTCCATGCTCTGCCACCT  1343
59_Danko_non-APR     TGACGGTGTGCGTCAACCTGCTCTTCACCTTCCTCATCGCGCAGGCCTTCCTCTCCATGCTCTGCCACCT  1343
61_Danko_non-APR     TGACGGTGTGCGTCAACCTACTCTTCACCTTCCTCATCGCGCAGGCCTTCCTCTCCATGCTCTGCCACCT  1343
123_Danko_non-APR    TGACGGTGTGCGTCAACCTGCTCTTCACCTTCCTCATCGCGCAGGCCTTCCTCTCCATGCTCTGCCACCT  1343
129_Danko_non-APR    TGACGGTGTGCGTCAACCTGCTCTTCACCTTCCTCATCGCGCAGGCCTTCCTCTCCATGCTCTGCCACCT  1343
37_PHR_non-APR       TGACGGTGTGCGTCAACCTGCTCTTCACCTTCCTCATCGCGCAGGCCTTCCTCTCCATGCTCTGCCACCT  1343
52_PHR_non-APR       TGACGGTGTGCGTCAACCTGCTCTTCACCTTCCTCATCGCGCAGGCCTTCCTCTCCATGCTCTGCCACCT  1343
88_PHR_non-APR       TGACGGTGTGCGTCAACCTGCTCTTCACCTTCCTCATCGCGCAGGCCTTCCTCTCCATGCTCTGCCACCT  1343
101_PHR_non-APR      TGACGGTGTGCGTCAACCTGCTCTTCACCTTCCTCATCGCGCAGGCCTTCCTCTCCATGCTCTGCCACCT  1343
105_PHR_non-APR      TGACGGTGTGCGTCAACCTGCTCTTCACCTTCCTCATCGCGCAGGCCTTCCTCTCCATGCTCTGCCACCT  1343
150_PHR_non-APR      TGACGGTGTGCGTCAACCTGCTCTTCACCTTCCTCATCGCGCAGGCCTTCCTCTCCATGCTCTGCCACCT  1364
150_PHR_non-APR2     TGACGGTGTGCGTCAACCTGCTCTTCACCTTCCTCATCGCGCAGGCCTTCCTCTCCATGCTCTGCCACCT  1346
```

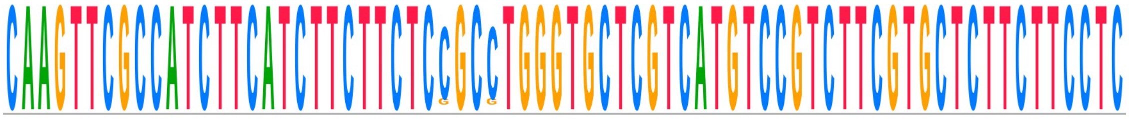

**Consensus**	CAAGTTCGCCATCTTCATCTTCTTCTCCGCCTGGGTGCTCGTCATGTCCGTCTTCGTGCTCTTCTTCCTC	
	1550 1560 1570 1580 1590 1600 1610	
Lr67(sus)	CAAGTTCGCCATCTTCATCTTCTTCTCGGCCTGGGTGCTCGTCATGTCCGTCTTCGTGCTCTTCTTCCTC	1413
Lr67(res)	CAAGTTCGCCATCTTCATCTTCTTCTCGGCCTGGGTGCTCGTCATGTCCGTCTTCGTGCTCTTCTTCCTC	1413
ScLr67_1 (Lo7)	CAAGTTCGCCATCTTCATCTTCTTCTCCGCGTGGGTGCTCGTCATGTCCGTCTTCGTGCTCTTCTTCCTC	1413
118_Danko_APR	CAAGTTCGCCATCTTCATCTTCTTCTCCGCCTGGGTGCTCGTCATGTCCGTCTTCGTGCTCTTCTTCCTC	1413
119_Danko_APR	CAAGTTCGCCATCTTCATCTTCTTCTCCGCCTGGGTGCTCGTCATGTCCGTCTTCGTGCTCTTCTTCCTC	1503
119_Danko_APR2	CAAGTTCGCCATCTTCATCTTCTTCTCCGCCTGGGTGCTCGTCATGTCCGTCTTCGTGCTCTTCTTCCTC	1578
120_Danko_APR	CAAGTTCGCCATCTTCATCTTCTTCTCCGCCTGGGTGCTCGTCATGTCCGTCTTCGTGCTCTTCTTCCTC	1413
138_Danko_APR	CAAGTTCGCCATCTTCATCTTCTTCTCCGCCTGGGTGCTCGTCATGTCCGTCTTCGTGCTCTTCTTCCTC	1413
153_Danko_APR	CAAGTTCGCCATCTTCATCTTCTTCTCCGCCTGGGTGCTCGTCATGTCCGTCTTCGTGCTCTTCTTCCTC	1413
157_Danko_APR	CAAGTTCGCCATCTTCATCTTCTTCTCCGCCTGGGTGCTCGTCATGTCCGTCTTCGTGCTCTTCTTCCTC	1413
160_Danko_APR	CAAGTTCGCCATCTTCATCTTCTTCTCCGCCTGGGTGCTCGTCATGTCCGTCTTCGTGCTCTTCTTCCTC	1413
71_PHR_APR	CAAGTTCGCCATCTTCATCTTCTTCTCCGCCTGGGTGCTCGTCATGTCCGTCTTCGTGCTCTTCTTCCTC	1413
149_PHR_APR	CAAGTTCGCCATCTTCATCTTCTTCTCCGCCTGGGTGCTCGTCATGTCCGTCTTCGTGCTCTTCTTCCTC	1413
59_Danko_non-APR	CAAGTTCGCCATCTTCATCTTCTTCTCCGCCTGGGTGCTCGTCATGTCCGTCTTCGTGCTCTTCTTCCTC	1413
61_Danko_non-APR	CAAGTTCGCCATCTTCATCTTCTTCTCCGCGTGGGTGCTCGTCATGTCCGTCTTCGTGCTCTTCTTCCTC	1413
123_Danko_non-APR	CAAGTTCGCCATCTTCATCTTCTTCTCCGCCTGGGTGCTCGTCATGTCCGTCTTCGTGCTCTTCTTCCTC	1413
129_Danko_non-APR	CAAGTTCGCCATCTTCATCTTCTTCTCCGCCTGGGTGCTCGTCATGTCCGTCTTCGTGCTCTTCTTCCTC	1413
37_PHR_non-APR	CAAGTTCGCCATCTTCATCTTCTTCTCCGCCTGGGTGCTCGTCATGTCCGTCTTCGTGCTCTTCTTCCTC	1413
52_PHR_non-APR	CAAGTTCGCCATCTTCATCTTCTTCTCCGCCTGGGTGCTCGTCATGTCCGTCTTCGTGCTCTTCTTCCTC	1413
88_PHR_non-APR	CAAGTTCGCCATCTTCATCTTCTTCTCCGCCTGGGTGCTCGTCATGTCCGTCTTCGTGCTCTTCTTCCTC	1413
101_PHR_non-APR	CAAGTTCGCCATCTTCATCTTCTTCTCCGCCTGGGTGCTCGTCATGTCCGTCTTCGTGCTCTTCTTCCTC	1413
105_PHR_non-APR	CAAGTTCGCCATCTTCATCTTCTTCTCCGCCTGGGTGCTCGTCATGTCCGTCTTCGTGCTCTTCTTCCTC	1413
150_PHR_non-APR	CAAGTTCGCCATCTTCATCTTCTTCTCCGCCTGGGTGCTCGTCATGTCCGTCTTCGTGCTCTTCTTCCTC	1434
150_PHR_non-APR2	CAAGTTCGCCATCTTCATCTTCTTCTCCGCCTGGGTGCTCGTCATGTCCGTCTTCGTGCTCTTCTTCCTC	1416

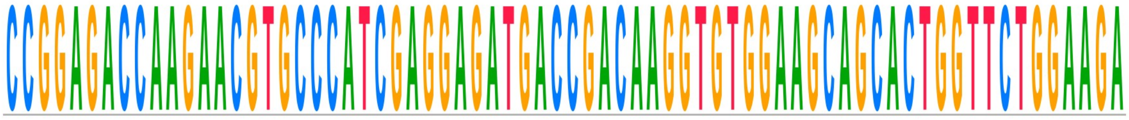

**Consensus**	CCGGAGACCAAGAACGTGCCCATCGAGGAGATGACCGACAAGGTGTGGAAGCAGCACTGGTTCTGGAAGA	
	1620 1630 1640 1650 1660 1670 1680	
Lr67(sus)	CCGGAGACCAAGAACGTGCCCATCGAGGAGATGACCGACAAGGTGTGGAAGCAGCACTGGTTCTGGAAGA	1483
Lr67(res)	CCGGAGACCAAGAACGTGCCCATCGAGGAGATGACCGACAAGGTGTGGAAGCAGCACTGGTTCTGGAAGA	1483
ScLr67_1 (Lo7)	CCGGAGACCAAGAACGTGCCCATCGAGGAGATGACCGACAAGGTGTGGAAGCAGCACTGGTTCTGGAAGA	1483
118_Danko_APR	CCGGAGACCAAGAACGTGCCCATCGAGGAGATGACCGACAAGGTGTGGAAGCAGCACTGGTTCTGGAAGA	1483
119_Danko_APR	CCGGAGACCAAGAACGTGCCCATCGAGGAGATGACCGACAAGGTGTGGAAGCAGCACTGGTTCTGGAAGA	1573
119_Danko_APR2	CCGGAGACCAAGAACGTGCCCATCGAGGAGATGACCGACAAGGTGTGGAAGCAGCACTGGTTCTGGAAGA	1648
120_Danko_APR	CCGGAGACCAAGAACGTGCCCATCGAGGAGATGACCGACAAGGTGTGGAAGCAGCACTGGTTCTGGAAGA	1483
138_Danko_APR	CCGGAGACCAAGAACGTGCCCATCGAGGAGATGACCGACAAGGTGTGGAAGCAGCACTGGTTCTGGAAGA	1483
153_Danko_APR	CCGGAGACCAAGAACGTGCCCATCGAGGAGATGACCGACAAGGTGTGGAAGCAGCACTGGTTCTGGAAGA	1483
157_Danko_APR	CCGGAGACCAAGAACGTGCCCATCGAGGAGATGACCGACAAGGTGTGGAAGCAGCACTGGTTCTGGAAGA	1483
160_Danko_APR	CCGGAGACCAAGAACGTGCCCATCGAGGAGATGACCGACAAGGTGTGGAAGCAGCACTGGTTCTGGAAGA	1483
71_PHR_APR	CCGGAGACCAAGAACGTGCCCATCGAGGAGATGACCGACAAGGTGTGGAAGCAGCACTGGTTCTGGAAGA	1483
149_PHR_APR	CCGGAGACCAAGAACGTGCCCATCGAGGAGATGACCGACAAGGTGTGGAAGCAGCACTGGTTCTGGAAGA	1483
59_Danko_non-APR	CCGGAGACCAAGAACGTGCCCATCGAGGAGATGACCGACAAGGTGTGGAAGCAGCACTGGTTCTGGAAGA	1483
61_Danko_non-APR	CCGGAGACCAAGAACGTGCCCATCGAGGAGATGACCGACAAGGTGTGGAAGCAGCACTGGTTCTGGAAGA	1483
123_Danko_non-APR	CCGGAGACCAAGAACGTGCCCATCGAGGAGATGACCGACAAGGTGTGGAAGCAGCACTGGTTCTGGAAGA	1483
129_Danko_non-APR	CCGGAGACCAAGAACGTGCCCATCGAGGAGATGACCGACAAGGTGTGGAAGCAGCACTGGTTCTGGAAGA	1483
37_PHR_non-APR	CCGGAGACCAAGAACGTGCCCATCGAGGAGATGACCGACAAGGTGTGGAAGCAGCACTGGTTCTGGAAGA	1483
52_PHR_non-APR	CCGGAGACCAAGAACGTGCCCATCGAGGAGATGACCGACAAGGTGTGGAAGCAGCACTGGTTCTGGAAGA	1483
88_PHR_non-APR	CCGGAGACCAAGAACGTGCCCATCGAGGAGATGACCGACAAGGTGTGGAAGCAGCACTGGTTCTGGAAGA	1483
101_PHR_non-APR	CCGGAGACCAAGAACGTGCCCATCGAGGAGATGACCGACAAGGTGTGGAAGCAGCACTGGTTCTGGAAGA	1483
105_PHR_non-APR	CCGGAGACCAAGAACGTGCCCATCGAGGAGATGACCGACAAGGTGTGGAAGCAGCACTGGTTCTGGAAGA	1483
150_PHR_non-APR	CCGGAGACCAAGAACGTGCCCATCGAGGAGATGACCGACAAGGTGTGGAAGCAGCACTGGTTCTGGAAGA	1504
150_PHR_non-APR2	CCGGAGACCAAGAACGTGCCCATCGAGGAGATGACCGACAAGGTGTGGAAGCAGCACTGGTTCTGGAAGA	1486

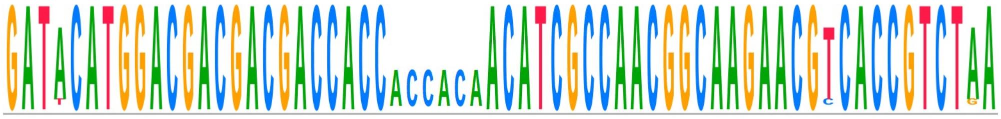

Sequence	Alignment (1690 – 1740)	
**Consensus**	GATACATGGACGACGACGACCACC------ACATCGCCAACGGCAAGAACGTCACCGTCTAA	
Lr67(sus)	GATTCATGGACGACGACGACCACCACCACAACATCGCCAACGGCAAGAACGCCACCGTCTGA	1545
Lr67(res)	GATTCATGGACGACGACGACCACCACCACAACATCGCCAACGGCAAGAACGCCACCGTCTGA	1545
ScLr67_1 (Lo7)	GATACATGGACGACGACGACCACC------ACATCGCCAACGGCAAGAACGTCACCGTCTAA	1539
118_Danko_APR	GATACATGGACGACGACGACCACC------ACATCGCCAACGGCAAGAACGTCACCGTCTAA	1539
119_Danko_APR	GATACATGGACGACGACGACCACC------ACATCGCCAACGGCAAGAACGTCACCGTCTAA	1629
119_Danko_APR2	GATACATGGACGACGACGACCACC------ACATCGCCAACGGCAAGAACGTCACCGTCTAA	1704
120_Danko_APR	GATACATGGACGACGACGACCACC------ACATCGCCAACGGCAAGAACGTCACCGTCTAA	1539
138_Danko_APR	GATACATGGACGACGACGACCACC------ACATCGCCAACGGCAAGAACGTCACCGTCTAA	1539
153_Danko_APR	GATACATGGACGACGACGACCACC------ACATCGCCAACGGCAAGAACGTCACCGTCTAA	1539
157_Danko_APR	GATACATGGACGACGACGACCACC------ACATCGCCAACGGCAAGAACGTCACCGTCTAA	1539
160_Danko_APR	GATACATGGACGACGACGACCACC------ACATCGCCAACGGCAAGAACGTCACCGTCTAA	1539
71_PHR_APR	GATACATGGACGACGACGACCACC------ACATCGCCAACGGCAAGAACGTCACCGTCTAA	1539
149_PHR_APR	GATACATGGACGACGACGACCACC------ACATCGCCAACGGCAAGAACGTCACCGTCTAA	1539
59_Danko_non-APR	GATACATGGACGACGACGACCACC------ACATCGCCAACGGCAAGAACGTCACCGTCTAA	1539
61_Danko_non-APR	GATACATGGACGACGACGACCACC------ACATCGCCAACGGCAAGAACGTCACCGTCTAA	1539
123_Danko_non-APR	GATACATGGACGACGACGACCACC------ACATCGCCAACGGCAAGAACGTCACCGTCTAA	1539
129_Danko_non-APR	GATACATGGACGACGACGACCACC------ACATCGCCAACGGCAAGAACGTCACCGTCTAA	1539
37_PHR_non-APR	GATACATGGACGACGACGACCACC------ACATCGCCAACGGCAAGAACGTCACCGTCTAA	1539
52_PHR_non-APR	GATACATGGACGACGACGACCACC------ACATCGCCAACGGCAAGAACGTCACCGTCTAA	1539
88_PHR_non-APR	GATACATGGACGACGACGACCACC------ACATCGCCAACGGCAAGAACGTCACCGTCTAA	1539
101_PHR_non-APR	GATACATGGACGACGACGACCACC------ACATCGCCAACGGCAAGAACGTCACCGTCTAA	1539
105_PHR_non-APR	GATACATGGACGACGACGACCACC------ACATCGCCAACGGCAAGAACGTCACCGTCTAA	1539
150_PHR_non-APR	GATACATGGACGACGACGACCACC------ACATCGCCAACGGCAAGAACGTCACCGTCTAA	1560
150_PHR_non-APR2	GATACATGGACGACGACGACCACC------ACATCGCCAACGGCAAGAACGTCACCGTCTAA	1542

Sequence Logo: 50% GC base composition

Consensus Threshold: >50%

Colors: 4-color highlighting

Created: 26 lis 2024

Last Modified: 26 lis 2024

Feature	Location	Size	⇄	Type
