# Supplementary material for: Searching for genes determining the APR phenotype in rye
Source: BMC Plant Biol. 2025 Jul 19;25:935. doi: 10.1186/s12870-025-06920-0 (PMC12275401; doi:10.1186/s12870-025-06920-0)
Supplement: Supplementary file 7 — Supplementary Material 7. [file 12870_2025_6920_MOESM7_ESM.pdf]

Aligned using MUSCLE

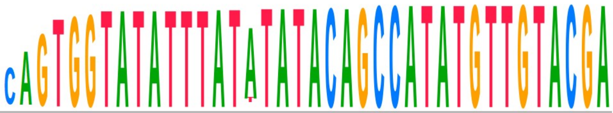

```
Consensus           --------------------------------------TATATTTATATATACAGCCATATGTTGTACGA
                             10        20        30        40        50        60        70
Lr67(sus)           ----------------------------------------------------------------------
Lr67(res)           ----------------------------------------------------------------------
ScLr67_1 (Lo7)      TTTATTTAGCCCGCTCTTCGTCGACATCGTGACAGTGGTATATTTATTTATACAGCCATATGTTGTACGA  70
118_Danko_APR       --------------------------------CAGTGGTATATTTATATATACAGCCATATGTTGTACGA  38
119_Danko_APR       -----------------------------------TGGTATATTTATATATACAGCCATATGTTGTACGA  35
119_Danko_APR2      -----------------------------------TGGTATATTTATATATACAGCCATATGTTGTACGA  35
120_Danko_APR       ---------------------------------AGTGGTATATTTATATATACAGCCATATGTTGTACGA  37
138_Danko_APR       -------------------------------------GTATATTTATATATACAGCCATATGTTGTACGA  33
153_Danko_APR       --------------------------------------TATATTTATATATACAGCCATATGTTGTACGA  32
157_Danko_APR       -----------------------------------TGGTATATTTATATATACAGCCATATGTTGTACGA  35
160_Danko_APR       ---------------------------------------------ATATATACAGCCATATGTTGTACGA  25
71_PHR_APR          --------------------------------------TATATTTATATATACAGCCATATGTTGTACGA  32
149_PHR_APR         ----------------------------------GTGGTATATTTATATATACAGCCATATGTTGTACGA  36
59_Danko_non-APR    ----------------------------------------------------------------------
61_Danko_non-APR    ------------------------------------------------TATACAGCCATATGTTGTACGA  22
123_Danko_non-APR   ----------------------------------------------------------------------
129_Danko_non-APR   ------------------------------------GGTATATTTATATATACAGCCATATGTTGTACGA  34
37_PHR_non-APR      ---------------------------------------------------------ATATGTTGTACGA  13
52_PHR_non-APR      --------------------------------------TATATTTATATATACAGCCATATGTTGTACGA  32
88_PHR_non-APR      -------------------------------------------------------CCATATGTTGTACGA  15
101_PHR_non-APR     ----------------------------------GTGGTATATTTATATATACAGCCATATGTTGTACGA  36
105_PHR_non-APR     -----------------------------------TGGTATATTTATATATACAGCCATATGTTGTACGA  35
150_PHR_non-APR     -------------------------------------------------------CCATATGTTGTACGA  15
150_PHR_non-APR2    -------------------------------------------------------CCATATGTTGTACGA  15
```

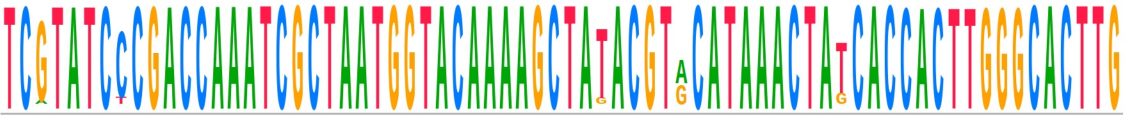

```
Consensus           TCGTATCCCGACCAAATCGCTAATGGTACAAAAGCTATACGTNCATAAACTATCACCACTTGGGCACTTG
                             80        90        100       110       120       130       140
Lr67(sus)           ----------------------------------------------------------------------
Lr67(res)           ----------------------------------------------------------------------
ScLr67_1 (Lo7)      TCGTATCTCGACCAAATCGCTAATGGTACAAAAGCTAGACGTACATAAACTAGCAC--------CACTTG  132
118_Danko_APR       TCGTATCCCGACCAAATCGCTAATGGTACAAAAGCTATACGTGCATAAACTATCACCACTTGGGCACTTG  108
119_Danko_APR       TCGTATCCCGACCAAATCGCTAATGGTACAAAAGCTATACGTACATAAACTATCACCACTTGGGCACTTG  105
119_Danko_APR2      TCGTATCCCGACCAAATCGCTAATGGTACAAAAGCTATACGTACATAAACTATCACCACTTGGGCACTTG  105
120_Danko_APR       TCGTATCCCGACCAAATCGCTAATGGTACAAAAGCTATACGTACATAAACTATCACCACTTGGGCACTTG  107
138_Danko_APR       TCGTATCCCGACCAAATCGCTAATGGTACAAAAGCTATACGTACATAAACTATCACCACTTGGGCACTTG  103
153_Danko_APR       TCGTATCCCGACCAAATCGCTAATGGTACAAAAGCTATACGTGCATAAACTATCACCACTTGGGCACTTG  102
157_Danko_APR       TCGTATCCCGACCAAATCGCTAATGGTACAAAAGCTATACGTACATAAACTATCACCACTTGGGCACTTG  105
160_Danko_APR       TCGTATCCCGACCAAATCGCTAATGGTACAAAAGCTATACGTACATAAACTATCACCACTTGGGCACTTG  95
71_PHR_APR          TCGTATCCCGACCAAATCGCTAATGGTACAAAAGCTATACGTACATAAACTAGCAC--------CACTTG  94
149_PHR_APR         TCGTATCCCGACCAAATCGCTAATGGTACAAAAGCTATACGTGCATAAACTATCACCACTTGGGCACTTG  106
59_Danko_non-APR    ----------------TCGCTAATGGTACAAAAGCTATACGTACATAAACTAGCAC--------CACTTG  46
61_Danko_non-APR    TCGTATCTCGACCAAATCGCTAATGGTACAAAAGCTAGACGTACATAAACTAGCAC--------CACTTG  84
123_Danko_non-APR   -------------AAATCGCTAATGGTACAAAAGCTATACGTGCATAAACTATCACCACTTGGGCACTTG  57
129_Danko_non-APR   TCGTATCCCGACCAAATCGCTAATGGTACAAAAGCTATACGTGCATAAACTATCACCACTTGGGCACTTG  104
37_PHR_non-APR      TCGTATCCCGACCAAATCGCTAATGGTACAAAAGCTATACGTACATAAACTATCACCACTTGGGCACTTG  83
52_PHR_non-APR      TCATATCCCGACCAAATCGCTAATGGTACAAAAGCTATACGTACATAAACTATCACCACTTGGGCACTTG  102
88_PHR_non-APR      TCGTATCCCGACCAAATCGCTAATGGTACAAAAGCTATACGTGCATAAACTATCACCACTTGGGCACTTG  85
101_PHR_non-APR     TCGTATCCCGACCAAATCGCTAATGGTACAAAAGCTATACGTGCATAAACTATCACCACTTGGGCACTTG  106
105_PHR_non-APR     TCGTATCCCGACCAAATCGCTAATGGTACAAAAGCTATACGTGCATAAACTATCACCACTTGGGCACTTG  105
150_PHR_non-APR     TCGTATCCCGACCAAATCGCTAATGGTACAAAAGCTATACGTGCATAAACTATCACCACTTGGGCACTTG  85
150_PHR_non-APR2    TCGTATCCCGACCAAATCGCTAATGGTACAAAAGCTATACGTGCATAAACTATCACCACTTGGGCACTTG  85
```

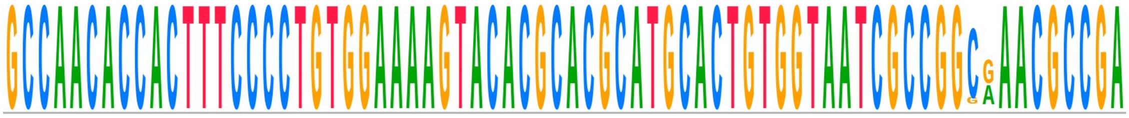

```
Consensus          GCCAACACCACTTTCCCCTGTGGAAAAGTACACGCACGCATGCACTGTGGTAATCGCCGGCNAACGCCGA
                        150       160       170       180       190       200       210
Lr67(sus)          ----------------------------------------------------------------------
Lr67(res)          ----------------------------------------------------------------------
ScLr67_1 (Lo7)     GCCAACACCACTTTCCCCTGTGGAAAAGTACACGCACGCATGCACTGTGGTAATCGCCGGGGAACGCCGA  202
118_Danko_APR      GCCAACACCACTTTCCCCTGTGGAAAAGTACACGCACGCATGCACTGTGGTAATCGCCGGCAAACGCCGA  178
119_Danko_APR      GCCAACACCACTTTCCCCTGTGGAAAAGTACACGCACGCATGCACTGTGGTAATCGCCGGCGAACGCCGA  175
119_Danko_APR2     GCCAACACCACTTTCCCCTGTGGAAAAGTACACGCACGCATGCACTGTGGTAATCGCCGGCGAACGCCGA  175
120_Danko_APR      GCCAACACCACTTTCCCCTGTGGAAAAGTACACGCACGCATGCACTGTGGTAATCGCCGGCGAACGCCGA  177
138_Danko_APR      GCCAACACCACTTTCCCCTGTGGAAAAGTACACGCACGCATGCACTGTGGTAATCGCCGGCGAACGCCGA  173
153_Danko_APR      GCCAACACCACTTTCCCCTGTGGAAAAGTACACGCACGCATGCACTGTGGTAATCGCCGGCAAACGCCGA  172
157_Danko_APR      GCCAACACCACTTTCCCCTGTGGAAAAGTACACGCACGCATGCACTGTGGTAATCGCCGGCGAACGCCGA  175
160_Danko_APR      GCCAACACCACTTTCCCCTGTGGAAAAGTACACGCACGCATGCACTGTGGTAATCGCCGGCGAACGCCGA  165
71_PHR_APR         GCCAACACCACTTTCCCCTGTGGAAAAGTACACGCACGCATGCACTGTGGTAATCGCCGGCGAACGCCGA  164
149_PHR_APR        GCCAACACCACTTTCCCCTGTGGAAAAGTACACGCACGCATGCACTGTGGTAATCGCCGGCAAACGCCGA  176
59_Danko_non-APR   GCCAACACCACTTTCCCCTGTGGAAAAGTACACGCACGCATGCACTGTGGTAATCGCCGGCGAACGCCGA  116
61_Danko_non-APR   GCCAACACCACTTTCCCCTGTGGAAAAGTACACGCACGCATGCACTGTGGTAATCGCCGGGGAACGCCGA  154
123_Danko_non-APR  GCCAACACCACTTTCCCCTGTGGAAAAGTACACGCACGCATGCACTGTGGTAATCGCCGGCAAACGCCGA  127
129_Danko_non-APR  GCCAACACCACTTTCCCCTGTGGAAAAGTACACGCACGCATGCACTGTGGTAATCGCCGGCAAACGCCGA  174
37_PHR_non-APR     GCCAACACCACTTTCCCCTGTGGAAAAGTACACGCACGCATGCACTGTGGTAATCGCCGGCGAACGCCGA  153
52_PHR_non-APR     GCCAACACCACTTTCCCCTGTGGAAAAGTACACGCACGCATGCACTGTGGTAATCGCCGGCGAACGCCGA  172
88_PHR_non-APR     GCCAACACCACTTTCCCCTGTGGAAAAGTACACGCACGCATGCACTGTGGTAATCGCCGGCAAACGCCGA  155
101_PHR_non-APR    GCCAACACCACTTTCCCCTGTGGAAAAGTACACGCACGCATGCACTGTGGTAATCGCCGGCAAACGCCGA  176
105_PHR_non-APR    GCCAACACCACTTTCCCCTGTGGAAAAGTACACGCACGCATGCACTGTGGTAATCGCCGGCAAACGCCGA  175
150_PHR_non-APR    GCCAACACCACTTTCCCCTGTGGAAAAGTACACGCACGCATGCACTGTGGTAATCGCCGGCAAACGCCGA  155
150_PHR_non-APR2   GCCAACACCACTTTCCCCTGTGGAAAAGTACACGCACGCATGCACTGTGGTAATCGCCGGCAAACGCCGA  155
```

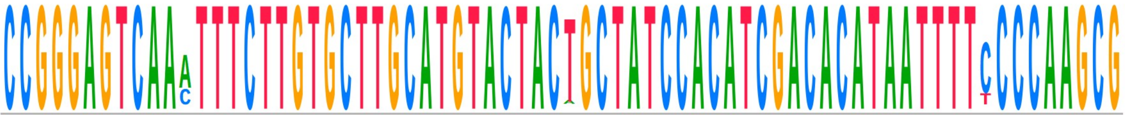

```
Consensus          CCGGGAGTCAAATTTCTTGTGCTTGCATGTACTACTGCTATCCACATCGACACATAATTTTCCCCAAGCG
                        220       230       240       250       260       270       280
Lr67(sus)          ----------------------------------------------------------------------
Lr67(res)          ----------------------------------------------------------------------
ScLr67_1 (Lo7)     CCGGGAGTCAAATTTCTTGTGCTTGCATGTA-TACTGCTATCCACATCGACACATAATTTTTCCCAAGCG  271
118_Danko_APR      CCGGGAGTCAAATTTCTTGTGCTTGCATGTACTACTGCTATCCACATCGACACATAATTTTCCCCAAGCG  248
119_Danko_APR      CCGGGAGTCAACTTTCTTGTGCTTGCATGTACTACTGCTATCCACATCGACACATAATTTTCCCCAAGCG  245
119_Danko_APR2     CCGGGAGTCAACTTTCTTGTGCTTGCATGTACTACTGCTATCCACATCGACACATAATTTTCCCCAAGCG  245
120_Danko_APR      CCGGGAGTCAACTTTCTTGTGCTTGCATGTACTACTGCTATCCACATCGACACATAATTTTCCCCAAGCG  247
138_Danko_APR      CCGGGAGTCAACTTTCTTGTGCTTGCATGTACTACTGCTATCCACATCGACACATAATTTTCCCCAAGCG  243
153_Danko_APR      CCGGGAGTCAAATTTCTTGTGCTTGCATGTACTACTGCTATCCACATCGACACATAATTTTCCCCAAGCG  242
157_Danko_APR      CCGGGAGTCAACTTTCTTGTGCTTGCATGTACTACTGCTATCCACATCGACACATAATTTTCCCCAAGCG  245
160_Danko_APR      CCGGGAGTCAACTTTCTTGTGCTTGCATGTACTACTGCTATCCACATCGACACATAATTTTCCCCAAGCG  235
71_PHR_APR         CCGGGAGTCAAATTTCTTGTGCTTGCATGTACTACTGCTATCCACATCGACACATAATTTTTCCCAAGCG  234
149_PHR_APR        CCGGGAGTCAAATTTCTTGTGCTTGCATGTACTACTGCTATCCACATCGACACATAATTTTCCCCAAGCG  246
59_Danko_non-APR   CCGGGAGTCAAATTTCTTGTGCTTGCATGTACTACAGCTATCCACATCGACACATAATTTTTCCCAAGCG  186
61_Danko_non-APR   CCGGGAGTCAAATTTCTTGTGCTTGCATGTA-TACTGCTATCCACATCGACACATAATTTTTCCCAAGCG  223
123_Danko_non-APR  CCGGGAGTCAAATTTCTTGTGCTTGCATGTACTACTGCTATCCACATCGACACATAATTTTCCCCAAGCG  197
129_Danko_non-APR  CCGGGAGTCAAATTTCTTGTGCTTGCATGTACTACTGCTATCCACATCGACACATAATTTTCCCCAAGCG  244
37_PHR_non-APR     CCGGGAGTCAACTTTCTTGTGCTTGCATGTACTACTGCTATCCACATCGACACATAATTTTCCCCAAGCG  223
52_PHR_non-APR     CCGGGAGTCAAATTTCTTGTGCTTGCATGTACTACTGCTATCCACATCGACACATAATTTTCCCCAAGCG  242
88_PHR_non-APR     CCGGGAGTCAAATTTCTTGTGCTTGCATGTACTACTGCTATCCACATCGACACATAATTTTCCCCAAGCG  225
101_PHR_non-APR    CCGGGAGTCAAATTTCTTGTGCTTGCATGTACTACTGCTATCCACATCGACACATAATTTTCCCCAAGCG  246
105_PHR_non-APR    CCGGGAGTCAAATTTCTTGTGCTTGCATGTACTACTGCTATCCACATCGACACATAATTTTCCCCAAGCG  245
150_PHR_non-APR    CCGGGAGTCAAATTTCTTGTGCTTGCATGTACTACTGCTATCCACATCGACACATAATTTTCCCCAAGCG  225
150_PHR_non-APR2   CCGGGAGTCAAATTTCTTGTGCTTGCATGTACTACTGCTATCCACATCGACACATAATTTTCCCCAAGCG  225
```

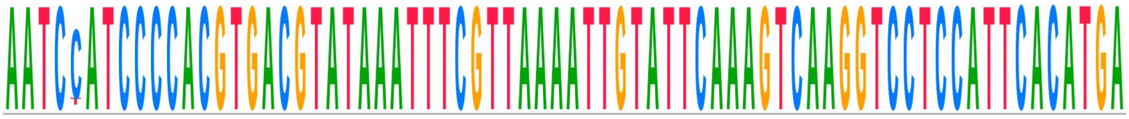

```
Consensus            AATCCATCCCCACGTGACGTATAAATTTCGTTAAAATTGTATTCAAAGTCAAGGTCCTCCATTCACATGA
                               290       300       310       320       330       340       350
Lr67(sus)            ---------------------------------------------------------------CACATGA  7
Lr67(res)            ---------------------------------------------------------------CACATGA  7
ScLr67_1 (Lo7)       AATCCATCCCCACGTGACGTATAAATTTCGTTAAAATTGTATTCAAAGTCAAGGTCCTCCATTCACATGA  341
118_Danko_APR        AATCCATCCCCACGTGACGTATAAATTTCGTTAAAATTGTATTCAAAGTCAAGGTCCTCCATTCACATGA  318
119_Danko_APR        AATCCATCCCCACGTGACGTATAAATTTCGTTAAAATTGTATTCAAAGTCAAGGTCCTCCATTCACATGA  315
119_Danko_APR2       AATCCATCCCCACGTGACGTATAAATTTCGTTAAAATTGTATTCAAAGTCAAGGTCCTCCATTCACATGA  315
120_Danko_APR        AATCCATCCCCACGTGACGTATAAATTTCGTTAAAATTGTATTCAAAGTCAAGGTCCTCCATTCACATGA  317
138_Danko_APR        AATCCATCCCCACGTGACGTATAAATTTCGTTAAAATTGTATTCAAAGTCAAGGTCCTCCATTCACATGA  313
153_Danko_APR        AATCCATCCCCACGTGACGTATAAATTTCGTTAAAATTGTATTCAAAGTCAAGGTCCTCCATTCACATGA  312
157_Danko_APR        AATCCATCCCCACGTGACGTATAAATTTCGTTAAAATTGTATTCAAAGTCAAGGTCCTCCATTCACATGA  315
160_Danko_APR        AATCCATCCCCACGTGACGTATAAATTTCGTTAAAATTGTATTCAAAGTCAAGGTCCTCCATTCACATGA  305
71_PHR_APR           AATCTATCCCCACGTGACGTATAAATTTCGTTAAAATTGTATTCAAAGTCAAGGTCCTCCATTCACATGA  304
149_PHR_APR          AATCCATCCCCACGTGACGTATAAATTTCGTTAAAATTGTATTCAAAGTCAAGGTCCTCCATTCACATGA  316
59_Danko_non-APR     AATCTATCCCCACGTGACGTATAAATTTCGTTAAAATTGTATTCAAAGTCAAGGTCCTCCATTCACATGA  256
61_Danko_non-APR     AATCCATCCCCACGTGACGTATAAATTTCGTTAAAATTGTATTCAAAGTCAAGGTCCTCCATTCACATGA  293
123_Danko_non-APR    AATCCATCCCCACGTGACGTATAAATTTCGTTAAAATTGTATTCAAAGTCAAGGTCCTCCATTCACATGA  267
129_Danko_non-APR    AATCCATCCCCACGTGACGTATAAATTTCGTTAAAATTGTATTCAAAGTCAAGGTCCTCCATTCACATGA  314
37_PHR_non-APR       AATCCATCCCCACGTGACGTATAAATTTCGTTAAAATTGTATTCAAAGTCAAGGTCCTCCATTCACATGA  293
52_PHR_non-APR       AATCCATCCCCACGTGACGTATAAATTTCGTTAAAATTGTATTCAAAGTCAAGGTCCTCCATTCACATGA  312
88_PHR_non-APR       AATCCATCCCCACGTGACGTATAAATTTCGTTAAAATTGTATTCAAAGTCAAGGTCCTCCATTCACATGA  295
101_PHR_non-APR      AATCCATCCCCACGTGACGTATAAATTTCGTTAAAATTGTATTCAAAGTCAAGGTCCTCCATTCACATGA  316
105_PHR_non-APR      AATCCATCCCCACGTGACGTATAAATTTCGTTAAAATTGTATTCAAAGTCAAGGTCCTCCATTCACATGA  315
150_PHR_non-APR      AATCCATCCCCACGTGACGTATAAATTTCGTTAAAATTGTATTCAAAGTCAAGGTCCTCCATTCACATGA  295
150_PHR_non-APR2     AATCCATCCCCACGTGACGTATAAATTTCGTTAAAATTGTATTCAAAGTCAAGGTCCTCCATTCACATGA  295
```

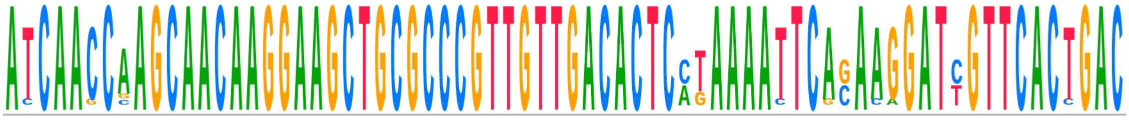

```
Consensus            ATCAACCAAGCAACAAGGAAGCTGCGCCCGTTGTTGACACTCCTAAAATTCAGAAGGATCGTTCACTGAC
                               360       370       380       390       400       410       420
Lr67(sus)            ATCAACCCAGCAACAAGGAAGCTGCGCCCGTTGTTGACACTCCGAAAACTCGGACAGATCGTTCACCGAC  77
Lr67(res)            ATCAACCCAGCAACAAGGAAGCTGCGCCCGTTGTTGACACTCCGAAAACTCGGACAGATCGTTCACCGAC  77
ScLr67_1 (Lo7)       ACCAAGCAAGCAACAAGGAAGCTGCGCCCGTTGTTGACACTCCGAAAATTCAGAAGGATTGTTCACTGAC  411
118_Danko_APR        ATCAACCAAGCAACAAGGAAGCTGCGCCCGTTGTTGACACTCATAAAATTCACAAGGATCGTTCACTGAC  388
119_Danko_APR        ATCAACCAAGCAACAAGGAAGCTGCGCCCGTTGTTGACACTCCTAAAATTCAGAAGGATTGTTCACTGAC  385
119_Danko_APR2       ATCAACCAAGCAACAAGGAAGCTGCGCCCGTTGTTGACACTCCTAAAATTCAGAAGGATTGTTCACTGAC  385
120_Danko_APR        ATCAACCAAGCAACAAGGAAGCTGCGCCCGTTGTTGACACTCCTAAAATTCAGAAGGATTGTTCACTGAC  387
138_Danko_APR        ATCAACCAAGCAACAAGGAAGCTGCGCCCGTTGTTGACACTCCTAAAATTCAGAAGGATTGTTCACTGAC  383
153_Danko_APR        ATCAACCAAGCAACAAGGAAGCTGCGCCCGTTGTTGACACTCATAAAATTCACAAGGATCGTTCACTGAC  382
157_Danko_APR        ATCAACCAAGCAACAAGGAAGCTGCGCCCGTTGTTGACACTCCTAAAATTCAGAAGGATTGTTCACTGAC  385
160_Danko_APR        ATCAACCAAGCAACAAGGAAGCTGCGCCCGTTGTTGACACTCCTAAAATTCAGAAGGATTGTTCACTGAC  375
71_PHR_APR           ATCAACCGAGCAACAAGGAAGCTGCGCCCGTTGTTGACACTCCGAAAATTCAGAAGGATTGTTCACTGAC  374
149_PHR_APR          ATCAACCAAGCAACAAGGAAGCTGCGCCCGTTGTTGACACTCATAAAATTCACAAGGATCGTTCACTGAC  386
59_Danko_non-APR     ATCAACCGAGCAACAAGGAAGCTGCGCCCGTTGTTGACACTCCGAAAATTCAGAAGGATTGTTCACTGAC  326
61_Danko_non-APR     ACCAAGCAAGCAACAAGGAAGCTGCGCCCGTTGTTGACACTCCGAAAATTCAGAAGGATTGTTCACTGAC  363
123_Danko_non-APR    ATCAACCAAGCAACAAGGAAGCTGCGCCCGTTGTTGACACTCATAAAATTCACAAGGATCGTTCACTGAC  337
129_Danko_non-APR    ATCAACCAAGCAACAAGGAAGCTGCGCCCGTTGTTGACACTCATAAAATTCACAAGGATCGTTCACTGAC  384
37_PHR_non-APR       ATCAACCAAGCAACAAGGAAGCTGCGCCCGTTGTTGACACTCCTAAAATTCAGAAGGATTGTTCACTGAC  363
52_PHR_non-APR       ATCAACCAAGCAACAAGGAAGCTGCGCCCGTTGTTGACACTCATAAAATTCACAAGGATCGTTCACTGAC  382
88_PHR_non-APR       ATCAACCAAGCAACAAGGAAGCTGCGCCCGTTGTTGACACTCATAAAATTCACAAGGATCGTTCACTGAC  365
101_PHR_non-APR      ATCAACCAAGCAACAAGGAAGCTGCGCCCGTTGTTGACACTCATAAAATTCACAAGGATCGTTCACTGAC  386
105_PHR_non-APR      ATCAACCAAGCAACAAGGAAGCTGCGCCCGTTGTTGACACTCATAAAATTCACAAGGATCGTTCACTGAC  385
150_PHR_non-APR      ATCAACCAAGCAACAAGGAAGCTGCGCCCGTTGTTGACACTCATAAAATTCACAAGGATCGTTCACTGAC  365
150_PHR_non-APR2     ATCAACCAAGCAACAAGGAAGCTGCGCCCGTTGTTGACACTCATAAAATTCACAAGGATCGTTCACTGAC  365
```

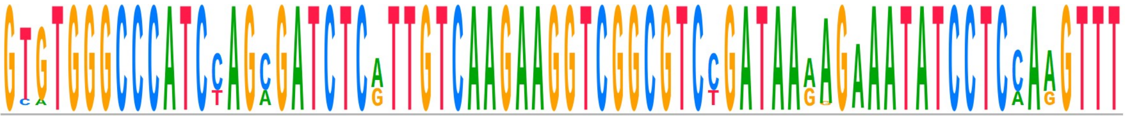

```
Consensus          GTGTGGGCCCATCCAGCGATCTCATTGTCAAGAAGGTCGGCGTCCGATAAAAG-AATATCCTCCAAGTTT
                            430       440       450       460       470       480       490
Lr67(sus)          GCATGGGCCCATCCAGCGATCTCGTTGTCAAGAAGGTCGGCGTCCGATAAGAG-AATATCCTCAAGGTTT  146
Lr67(res)          GCATGGGCCCATCCAGCGATCTCGTTGTCAAGAAGGTCGGCGTCCGATAAGAG-AATATCCTCAAGGTTT  146
ScLr67_1 (Lo7)     GTGTGGGCCCATCCAGCGATCTCATTGTCAAGAAGGTCGGCGTCCGATAAAAGAAATATCCTCAAGGTTT  481
118_Danko_APR      GTGTGGGCCCATCCAGCGATCTCATTGTCAAGAAGGTCGGCGTCCGATAAAAG-AATATCCTCCAAGTTT  457
119_Danko_APR      GTGTGGGCCCATCTAGAGATCTCGTTGTCAAGAAGGTCGGCGTCTGATAAGAG-AATATCCTCCAAGTTT  454
119_Danko_APR2     GTGTGGGCCCATCTAGAGATCTCGTTGTCAAGAAGGTCGGCGTCTGATAAGAG-AATATCCTCCAAGTTT  454
120_Danko_APR      GTGTGGGCCCATCTAGAGATCTCGTTGTCAAGAAGGTCGGCGTCTGATAAGAG-AATATCCTCCAAGTTT  456
138_Danko_APR      GTGTGGGCCCATCTAGAGATCTCGTTGTCAAGAAGGTCGGCGTCTGATAAGAG-AATATCCTCCAAGTTT  452
153_Danko_APR      GTGTGGGCCCATCCAGCGATCTCATTGTCAAGAAGGTCGGCGTCCGATAAAAG-AATATCCTCCAAGTTT  451
157_Danko_APR      GTGTGGGCCCATCTAGAGATCTCGTTGTCAAGAAGGTCGGCGTCTGATAAGAG-AATATCCTCCAAGTTT  454
160_Danko_APR      GTGTGGGCCCATCTAGAGATCTCGTTGTCAAGAAGGTCGGCGTCTGATAAGAG-AATATCCTCCAAGTTT  444
71_PHR_APR         GTGTGGGCCCATCCAGCGATCTCATTGTCAAGAAGGTCGGCGTCCGATAAAAGAAATATCCTCAAGGTTT  444
149_PHR_APR        GTGTGGGCCCATCCAGCGATCTCATTGTCAAGAAGGTCGGCGTCCGATAAAAG-AATATCCTCCAAGTTT  455
59_Danko_non-APR   GTGTGGGCCCATCCAGCGATCTCATTGTCAAGAAGGTCGGCGTCCGATAAAAGAAATATCCTCAAGGTTT  396
61_Danko_non-APR   GTGTGGGCCCATCCAGCGATCTCATTGTCAAGAAGGTCGGCGTCCGATAAAAGAAATATCCTCAAGGTTT  433
123_Danko_non-APR  GTGTGGGCCCATCCAGCGATCTCATTGTCAAGAAGGTCGGCGTCCGATAAAAG-AATATCCTCCAAGTTT  406
129_Danko_non-APR  GTGTGGGCCCATCCAGCGATCTCATTGTCAAGAAGGTCGGCGTCCGATAAAAG-AATATCCTCCAAGTTT  453
37_PHR_non-APR     GTGTGGGCCCATCTAGAGATCTCGTTGTCAAGAAGGTCGGCGTCTGATAAGAG-AATATCCTCCAAGTTT  432
52_PHR_non-APR     GTGTGGGCCCATCCAGCGATCTCATTGTCAAGAAGGTCGGCGTCCGATAAAGG-AATATCCTCCAAGTTT  451
88_PHR_non-APR     GTGTGGGCCCATCCAGCGATCTCATTGTCAAGAAGGTCGGCGTCCGATAAAAG-AATATCCTCCAAGTTT  434
101_PHR_non-APR    GTGTGGGCCCATCCAGCGATCTCATTGTCAAGAAGGTCGGCGTCCGATAAAAG-AATATCCTCCAAGTTT  455
105_PHR_non-APR    GTGTGGGCCCATCCAGCGATCTCATTGTCAAGAAGGTCGGCGTCCGATAAAAG-AATATCCTCCAAGTTT  454
150_PHR_non-APR    GTGTGGGCCCATCCAGCGATCTCATTGTCAAGAAGGTCGGCGTCCGATAAAAG-AATATCCTCCAAGTTT  434
150_PHR_non-APR2   GTGTGGGCCCATCCAGCGATCTCATTGTCAAGAAGGTCGGCGTCCGATAAAAG-AATATCCTCCAAGTTT  434
```

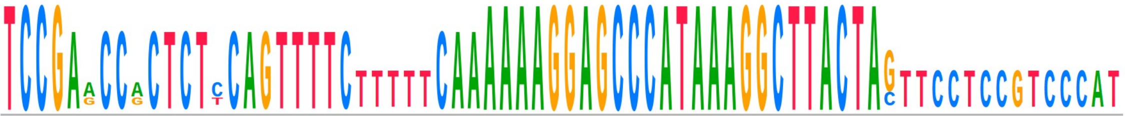

```
Consensus          TCCG--------------------------AAAAGGAGCCCATAAAGGCTTACTAG--------------
                            500       510       520       530       540       550       560
Lr67(sus)          TCCGAGCCGCTCTTCAGTTTTCTTTTTCAAAAAAGGAGCCCATAAAGGCTTACTACTTCCTCCGTCCCAT  216
Lr67(res)          TCCGAGCCGCTCTTCAGTTTTCTTTTTCAAAAAAGGAGCCCATAAAGGCTTACTACTTCCTCCGTCCCAT  216
ScLr67_1 (Lo7)     TCCGAACCACTCTCCAGTTTTC-----CAAAAAAGGAGCCCATAAAGGCTTACTAC--------------  532
118_Danko_APR      TCCG--------------------------AAAAGGAGCCCATAAAGGCTTACTAG--------------  487
119_Danko_APR      TCCG--------------------------AAAAGGAGCCCATAAAGGCTTACTAG--------------  484
119_Danko_APR2     TCCG--------------------------AAAAGGAGCCCATAAAGGCTTACTAG--------------  484
120_Danko_APR      TCCG--------------------------AAAAGGAGCCCATAAAGGCTTACTAG--------------  486
138_Danko_APR      TCCG--------------------------AAAAGGAGCCCATAAAGGCTTACTAG--------------  482
153_Danko_APR      TCCG--------------------------AAAAGGAGCCCATAAAGGCTTACTAG--------------  481
157_Danko_APR      TCCG--------------------------AAAAGGAGCCCATAAAGGCTTACTAG--------------  484
160_Danko_APR      TCCG--------------------------AAAAGGAGCCCATAAAGGCTTACTAG--------------  474
71_PHR_APR         TCCGAACCACTCTCCAGTTTTC-----CAAAAAAGGAGCCCATAAAGGCTTACTAC--------------  495
149_PHR_APR        TCCG--------------------------AAAAGGAGCCCATAAAGGCTTACTAG--------------  485
59_Danko_non-APR   TCCGAACCACTCTCCAGTTTTC-----CAAAAAAGGAGCCCATAAAGGCTTACTAC--------------  447
61_Danko_non-APR   TCCGAACCACTCTCCAGTTTTC-----CAAAAAAGGAGCCCATAAAGGCTTACTAC--------------  484
123_Danko_non-APR  TCCG--------------------------AAAAGGAGCCCATAAAGGCTTACTAG--------------  436
129_Danko_non-APR  TCCG--------------------------AAAAGGAGCCCATAAAGGCTTACTAG--------------  483
37_PHR_non-APR     TCCG--------------------------AAAAGGAGCCCATAAAGGCTTACTAG--------------  462
52_PHR_non-APR     TCCG--------------------------AAAAGGAGCCCATAAAGGCTTACTAG--------------  481
88_PHR_non-APR     TCCG--------------------------AAAAGGAGCCCATAAAGGCTTACTAG--------------  464
101_PHR_non-APR    TCCG--------------------------AAAAGGAGCCCATAAAGGCTTACTAG--------------  485
105_PHR_non-APR    TCCG--------------------------AAAAGGAGCCCATAAAGGCTTACTAG--------------  484
150_PHR_non-APR    TCCG--------------------------AAAAGGAGCCCATAAAGGCTTACTAG--------------  464
150_PHR_non-APR2   TCCG--------------------------AAAAGGAGCCCATAAAGGCTTACTAG--------------  464
```

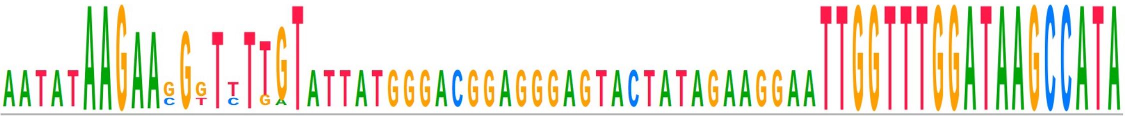

**Consensus**	-----AAG--------TGT--------------------------------TTGGTTTGGATAAGCCATA	
	570 580 590 600 610 620 630	
Lr67(sus)	AATATAAGAACGTTCTTATATTATGGGACGGAGGGAGTACTATAGAAGGAATTGGTTTGGATAAGCCATA	286
Lr67(res)	AATATAAGAACGTTCTTATATTATGGGACGGAGGGAGTACTATAGAAGGAATTGGTTTGGATAAGCCATA	286
ScLr67_1 (Lo7)	-----AAGAAGGGTTTGGT--------------------------------TTGGTTTGGATAAGCCATA	565
118_Danko_APR	-----AAG--------TGT--------------------------------TTGGTTTGGATAAGCCATA	512
119_Danko_APR	-----AAG--------TGT--------------------------------TTGGTTTGGATAAGCCATA	509
119_Danko_APR2	-----AAG--------TGT--------------------------------TTGGTTTGGATAAGCCATA	509
120_Danko_APR	-----AAG--------TGT--------------------------------TTGGTTTGGATAAGCCATA	511
138_Danko_APR	-----AAG--------TGT--------------------------------TTGGTTTGGATAAGCCATA	507
153_Danko_APR	-----AAG--------TGT--------------------------------TTGGTTTGGATAAGCCATA	506
157_Danko_APR	-----AAG--------TGT--------------------------------TTGGTTTGGATAAGCCATA	509
160_Danko_APR	-----AAG--------TGT--------------------------------TTGGTTTGGATAAGCCATA	499
71_PHR_APR	-----AAGAAGGGTTTGGT--------------------------------TTGGTTTGGATAAGCCATA	528
149_PHR_APR	-----AAG--------TGT--------------------------------TTGGTTTGGATAAGCCATA	510
59_Danko_non-APR	-----AAGAAGGGTTTGGT--------------------------------TTGGTTTGGATAAGCCATA	480
61_Danko_non-APR	-----AAGAAGGGTTTGGT--------------------------------TTGGTTTGGATAAGCCATA	517
123_Danko_non-APR	-----AAG--------TGT--------------------------------TTGGTTTGGATAAGCCATA	461
129_Danko_non-APR	-----AAG--------TGT--------------------------------TTGGTTTGGATAAGCCATA	508
37_PHR_non-APR	-----AAG--------TGT--------------------------------TTGGTTTGGATAAGCCATA	487
52_PHR_non-APR	-----AAG--------TGT--------------------------------TTGGTTTGGATAAGCCATA	506
88_PHR_non-APR	-----AAG--------TGT--------------------------------TTGGTTTGGATAAGCCATA	489
101_PHR_non-APR	-----AAG--------TGT--------------------------------TTGGTTTGGATAAGCCATA	510
105_PHR_non-APR	-----AAG--------TGT--------------------------------TTGGTTTGGATAAGCCATA	509
150_PHR_non-APR	-----AAG--------TGT--------------------------------TTGGTTTGGATAAGCCATA	489
150_PHR_non-APR2	-----AAG--------TGT--------------------------------TTGGTTTGGATAAGCCATA	489

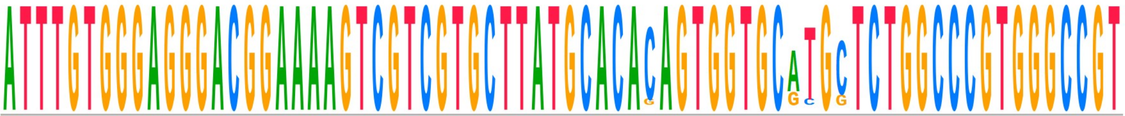

**Consensus**	ATTTGTGGGAGGGACGGAAAAGTCGTCGTGCTTATGCACACAGTGGTGCATGCTCTGGCCCGTGGGCCGT	
	640 650 660 670 680 690 700	
Lr67(sus)	ATTTGTGGGAGGGACGGAAAAGTCGTCGTGCTTATGCACAGAGTGGTGCGCGCTCTGGCCCGTGGGCCGT	356
Lr67(res)	ATTTGTGGGAGGGACGGAAAAGTCGTCGTGCTTATGCACAGAGTGGTGCGCGCTCTGGCCCGTGGGCCGT	356
ScLr67_1 (Lo7)	ATTTGTGGGAGGGACGGAAAAGTCGTCGTGCTTATGCACACAGTGGTGCGTGGTCTGGCCCGTGGGCCGT	635
118_Danko_APR	ATTTGTGGGAGGGACGGAAAAGTCGTCGTGCTTATGCACACAGTGGTGCATGCTCTGGCCCGTGGGCCGT	582
119_Danko_APR	ATTTGTGGGAGGGACGGAAAAGTCGTCGTGCTTATGCACACAGTGGTGCATGCTCTGGCCCGTGGGCCGT	579
119_Danko_APR2	ATTTGTGGGAGGGACGGAAAAGTCGTCGTGCTTATGCACACAGTGGTGCATGCTCTGGCCCGTGGGCCGT	579
120_Danko_APR	ATTTGTGGGAGGGACGGAAAAGTCGTCGTGCTTATGCACACAGTGGTGCATGCTCTGGCCCGTGGGCCGT	581
138_Danko_APR	ATTTGTGGGAGGGACGGAAAAGTCGTCGTGCTTATGCACACAGTGGTGCATGCTCTGGCCCGTGGGCCGT	577
153_Danko_APR	ATTTGTGGGAGGGACGGAAAAGTCGTCGTGCTTATGCACACAGTGGTGCATGCTCTGGCCCGTGGGCCGT	576
157_Danko_APR	ATTTGTGGGAGGGACGGAAAAGTCGTCGTGCTTATGCACACAGTGGTGCATGCTCTGGCCCGTGGGCCGT	579
160_Danko_APR	ATTTGTGGGAGGGACGGAAAAGTCGTCGTGCTTATGCACACAGTGGTGCATGCTCTGGCCCGTGGGCCGT	569
71_PHR_APR	ATTTGTGGGAGGGACGGAAAAGTCGTCGTGCTTATGCACACAGTGGTGCGTGGTCTGGCCCGTGGGCCGT	598
149_PHR_APR	ATTTGTGGGAGGGACGGAAAAGTCGTCGTGCTTATGCACACAGTGGTGCATGCTCTGGCCCGTGGGCCGT	580
59_Danko_non-APR	ATTTGTGGGAGGGACGGAAAAGTCGTCGTGCTTATGCACACAGTGGTGCGTGGTCTGGCCCGTGGGCCGT	550
61_Danko_non-APR	ATTTGTGGGAGGGACGGAAAAGTCGTCGTGCTTATGCACACAGTGGTGCGTGGTCTGGCCCGTGGGCCGT	587
123_Danko_non-APR	ATTTGTGGGAGGGACGGAAAAGTCGTCGTGCTTATGCACACAGTGGTGCATGCTCTGGCCCGTGGGCCGT	531
129_Danko_non-APR	ATTTGTGGGAGGGACGGAAAAGTCGTCGTGCTTATGCACACAGTGGTGCATGCTCTGGCCCGTGGGCCGT	578
37_PHR_non-APR	ATTTGTGGGAGGGACGGAAAAGTCGTCGTGCTTATGCACACAGTGGTGCATGCTCTGGCCCGTGGGCCGT	557
52_PHR_non-APR	ATTTGTGGGAGGGACGGAAAAGTCGTCGTGCTTATGCACACAGTGGTGCATGCTCTGGCCCGTGGGCCGT	576
88_PHR_non-APR	ATTTGTGGGAGGGACGGAAAAGTCGTCGTGCTTATGCACACAGTGGTGCATGCTCTGGCCCGTGGGCCGT	559
101_PHR_non-APR	ATTTGTGGGAGGGACGGAAAAGTCGTCGTGCTTATGCACACAGTGGTGCATGCTCTGGCCCGTGGGCCGT	580
105_PHR_non-APR	ATTTGTGGGAGGGACGGAAAAGTCGTCGTGCTTATGCACACAGTGGTGCATGCTCTGGCCCGTGGGCCGT	579
150_PHR_non-APR	ATTTGTGGGAGGGACGGAAAAGTCGTCGTGCTTATGCACACAGTGGTGCATGCTCTGGCCCGTGGGCCGT	559
150_PHR_non-APR2	ATTTGTGGGAGGGACGGAAAAGTCGTCGTGCTTATGCACACAGTGGTGCATGCTCTGGCCCGTGGGCCGT	559

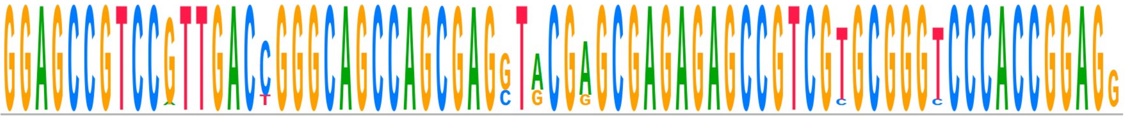

```
Consensus            GGAGCCGTCCGTTGACCGGGCAGCCAGCGAGGTACGAGCGAGAGAGCCGTCGTGCGGGTCCCACCGGAG-
                              710       720       730       740       750       760       770
Lr67(sus)            GGAGCCGTCCGTTGACCGGGCAGCC------CTGCGAGCGAGAGAGCCGTCGCGCGGGCCCCACCGGAGG  420
Lr67(res)            GGAGCCGTCCGTTGACCGGGCAGCC------CTGCGAGCGAGAGAGCCGTCGCGCGGGCCCCACCGGAGG  420
ScLr67_1 (Lo7)       GGAGCCGTCCGTTGACTGGGCAGCCAGCGAGCTGCGGGCGAGAGAGCCGTCGTGCGGGTCCCACCGGAG-  704
118_Danko_APR        GGAGCCGTCCGTTGACCGGGCAGCCAGCGAGGTACGAGCGAGAGAGCCGTCGTGCGGGTCCCACCGGAG-  651
119_Danko_APR        GGAGCCGTCCGTTGACCGGGCAGCCAGCGAGGTACGAGCGAGAGAGCCGTCGTGCGGGTCCCACCGGAG-  648
119_Danko_APR2       GGAGCCGTCCGTTGACCGGGCAGCCAGCGAGGTACGAGCGAGAGAGCCGTCGTGCGGGTCCCACCGGAG-  648
120_Danko_APR        GGAGCCGTCCGTTGACCGGGCAGCCAGCGAGGTACGAGCGAGAGAGCCGTCGTGCGGGTCCCACCGGAG-  650
138_Danko_APR        GGAGCCGTCCGTTGACCGGGCAGCCAGCGAGGTACGAGCGAGAGAGCCGTCGTGCGGGTCCCACCGGAG-  646
153_Danko_APR        GGAGCCGTCCGTTGACCGGGCAGCCAGCGAGGTACGAGCGAGAGAGCCGTCGTGCGGGTCCCACCGGAG-  645
157_Danko_APR        GGAGCCGTCCGTTGACCGGGCAGCCAGCGAGGTACGAGCGAGAGAGCCGTCGTGCGGGTCCCACCGGAG-  648
160_Danko_APR        GGAGCCGTCCGTTGACCGGGCAGCCAGCGAGGTACGAGCGAGAGAGCCGTCGTGCGGGTCCCACCGGAG-  638
71_PHR_APR           GGAGCCGTCCGTTGACTGGGCAGCCAGCGAGCTGCGGGCGAGAGAGCCGTCGTGCGGGTCCCACCGGAG-  667
149_PHR_APR          GGAGCCGTCCGTTGACCGGGCAGCCAGCGAGGTACGAGCGAGAGAGCCGTCGTGCGGGTCCCACCGGAG-  649
59_Danko_non-APR     GGAGCCGTCCGTTGACTGGGCAGCCAGCGAGCTGCGGGCGAGAGAGCCGTCGTGCGGGTCCCACCGGAG-  619
61_Danko_non-APR     GGAGCCGTCCGTTGACTGGGCAGCCAGCGAGCTGCGGGCGAGAGAGCCGTCGTGCGGGTCCCACCGGAG-  656
123_Danko_non-APR    GGAGCCGTCCGTTGACCGGGCAGCCAGCGAGGTACGAGCGAGAGAGCCGTCGTGCGGGTCCCACCGGAG-  600
129_Danko_non-APR    GGAGCCGTCCGTTGACCGGGCAGCCAGCGAGGTACGAGCGAGAGAGCCGTCGTGCGGGTCCCACCGGAG-  647
37_PHR_non-APR       GGAGCCGTCCGTTGACCGGGCAGCCAGCGAGGTACGAGCGAGAGAGCCGTCGTGCGGGTCCCACCGGAG-  626
52_PHR_non-APR       GGAGCCGTCCATTGACCGGGCAGCCAGCGAGCTGCGAGCGAGAGAGCCGTCGTGCGGGTCCCACCGGAG-  645
88_PHR_non-APR       GGAGCCGTCCGTTGACCGGGCAGCCAGCGAGGTACGAGCGAGAGAGCCGTCGTGCGGGTCCCACCGGAG-  628
101_PHR_non-APR      GGAGCCGTCCGTTGACCGGGCAGCCAGCGAGGTACGAGCGAGAGAGCCGTCGTGCGGGTCCCACCGGAG-  649
105_PHR_non-APR      GGAGCCGTCCGTTGACCGGGCAGCCAGCGAGGTACGAGCGAGAGAGCCGTCGTGCGGGTCCCACCGGAG-  648
150_PHR_non-APR      GGAGCCGTCCGTTGACCGGGCAGCCAGCGAGGTACGAGCGAGAGAGCCGTCGTGCGGGTCCCACCGGAG-  628
150_PHR_non-APR2     GGAGCCGTCCGTTGACCGGGCAGCCAGCGAGGTACGAGCGAGAGAGCCGTCGTGCGGGTCCCACCGGAG-  628
```

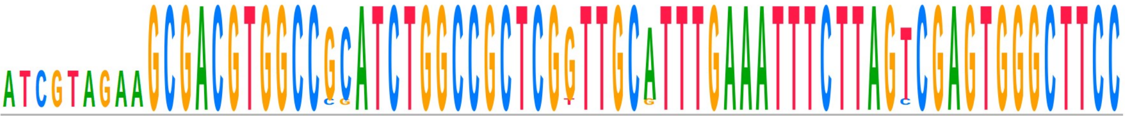

```
Consensus            ---------GCGACGTGGCCGCATCTGGCCGCTCGGTTGCATTTGAAATTTCTTAGTCGAGTGGGCTTCC
                              780       790       800       810       820       830       840
Lr67(sus)            ATCGTAGAAGCGACGTGGCCCGATCTGGCCGCTCGGTTGCGTTTGAAATTTCTTAGCCGAGTGGGCTTCC  490
Lr67(res)            ATCGTAGAAGCGACGTGGCCCGATCTGGCCGCTCGGTTGCGTTTGAAATTTCTTAGCCGAGTGGGCTTCC  490
ScLr67_1 (Lo7)       ---------GCGACGTGGCCGCATCTGGCCGCTCGTTTGCATTTGAAATTTCTTAGTCGAGTGGGCTTCC  765
118_Danko_APR        ---------GCGACGTGGCCGCATCTGGCCGCTCGGTTGCATTTGAAATTTCTTAGTCGAGTGGGCTTCC  712
119_Danko_APR        ---------GCGACGTGGCCGCATCTGGCCGCTCGGTTGCATTTGAAATTTCTTAGTCGAGTGGGCTTCC  709
119_Danko_APR2       ---------GCGACGTGGCCGCATCTGGCCGCTCGGTTGCATTTGAAATTTCTTAGTCGAGTGGGCTTCC  709
120_Danko_APR        ---------GCGACGTGGCCGCATCTGGCCGCTCGGTTGCATTTGAAATTTCTTAGTCGAGTGGGCTTCC  711
138_Danko_APR        ---------GCGACGTGGCCGCATCTGGCCGCTCGGTTGCATTTGAAATTTCTTAGTCGAGTGGGCTTCC  707
153_Danko_APR        ---------GCGACGTGGCCGCATCTGGCCGCTCGGTTGCATTTGAAATTTCTTAGTCGAGTGGGCTTCC  706
157_Danko_APR        ---------GCGACGTGGCCGCATCTGGCCGCTCGGTTGCATTTGAAATTTCTTAGTCGAGTGGGCTTCC  709
160_Danko_APR        ---------GCGACGTGGCCGCATCTGGCCGCTCGGTTGCATTTGAAATTTCTTAGTCGAGTGGGCTTCC  699
71_PHR_APR           ---------GCGACGTGGCCGCATCTGGCCGCTCGGTTGCATTTGAAATTTCTTAGTCGAGTGGGCTTCC  728
149_PHR_APR          ---------GCGACGTGGCCGCATCTGGCCGCTCGGTTGCATTTGAAATTTCTTAGTCGAGTGGGCTTCC  710
59_Danko_non-APR     ---------GCGACGTGGCCGCATCTGGCCGCTCGGTTGCATTTGAAATTTCTTAGTCGAGTGGGCTTCC  680
61_Danko_non-APR     ---------GCGACGTGGCCGCATCTGGCCGCTCGTTTGCATTTGAAATTTCTTAGTCGAGTGGGCTTCC  717
123_Danko_non-APR    ---------GCGACGTGGCCGCATCTGGCCGCTCGGTTGCATTTGAAATTTCTTAGTCGAGTGGGCTTCC  661
129_Danko_non-APR    ---------GCGACGTGGCCGCATCTGGCCGCTCGGTTGCATTTGAAATTTCTTAGTCGAGTGGGCTTCC  708
37_PHR_non-APR       ---------GCGACGTGGCCGCATCTGGCCGCTCGGTTGCATTTGAAATTTCTTAGTCGAGTGGGCTTCC  687
52_PHR_non-APR       ---------GCGACGTGGCCGCATCTGGCCGCTCGGTTGCATTTGAAATTTCTTAGTCGAGTGGGCTTCC  706
88_PHR_non-APR       ---------GCGACGTGGCCGCATCTGGCCGCTCGGTTGCATTTGAAATTTCTTAGTCGAGTGGGCTTCC  689
101_PHR_non-APR      ---------GCGACGTGGCCGCATCTGGCCGCTCGGTTGCATTTGAAATTTCTTAGTCGAGTGGGCTTCC  710
105_PHR_non-APR      ---------GCGACGTGGCCGCATCTGGCCGCTCGGTTGCATTTGAAATTTCTTAGTCGAGTGGGCTTCC  709
150_PHR_non-APR      ---------GCGACGTGGCCGCATCTGGCCGCTCGGTTGCATTTGAAATTTCTTAGTCGAGTGGGCTTCC  689
150_PHR_non-APR2     ---------GCGACGTGGCCGCATCTGGCCGCTCGGTTGCATTTGAAATTTCTTAGTCGAGTGGGCTTCC  689
```

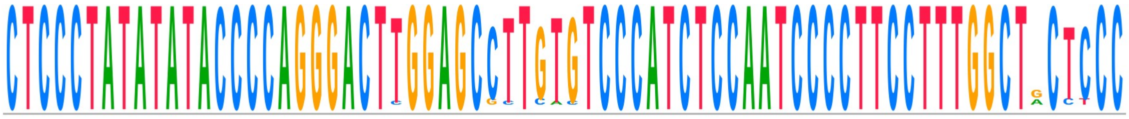

Name	Sequence	End
**Consensus**	CTCCCTATATATACCCCAGGGACTTGGAGCCTTGTGTCCCATCTCCAATCCCCTTCCTTTGGCT-CTCCC	
	850 860 870 880 890 900 910	
Lr67(sus)	CTCCCTATATATACCCCAGGGACTTGGAGCGTTGTGTCCCATCTCCAATCCCCTTCCTTTGGCT--CTCC	558
Lr67(res)	CTCCCTATATATACCCCAGGGACTTGGAGCGTTGTGTCCCATCTCCAATCCCCTTCCTTTGGCT--CTCC	558
ScLr67_1 (Lo7)	CTCCCTATATATA-CCCAGGGACTTGGAGCCTTCTGTCCCATCTCCAATCCCCTTCCTTTGGCTGCTCCC	834
118_Danko_APR	CTCCCTATATATACCCCAGGGACTTGGAGCCTTGTGTCCCATCTCCAATCCCCTTCCTTTGGCT-CTCCC	781
119_Danko_APR	CTCCCTATATATACCCCAGGGACTTGGAGCCTTGTGTCCCATCTCCAATCCCCTTCCTTTGGCT-CTCCC	778
119_Danko_APR2	CTCCCTATATATACCCCAGGGACTTGGAGCCTTGTGTCCCATCTCCAATCCCCTTCCTTTGGCT-CTCCC	778
120_Danko_APR	CTCCCTATATATACCCCAGGGACTTGGAGCCTTGTGTCCCATCTCCAATCCCCTTCCTTTGGCT-CTCCC	780
138_Danko_APR	CTCCCTATATATACCCCAGGGACTTGGAGCCTTGTGTCCCATCTCCAATCCCCTTCCTTTGGCT-CTCCC	776
153_Danko_APR	CTCCCTATATATACCCCAGGGACTTGGAGCCTTGTGTCCCATCTCCAATCCCCTTCCTTTGGCT-CTCCC	775
157_Danko_APR	CTCCCTATATATACCCCAGGGACTTGGAGCCTTGTGTCCCATCTCCAATCCCCTTCCTTTGGCT-CTCCC	778
160_Danko_APR	CTCCCTATATATACCCCAGGGACTTGGAGCCTTGTGTCCCATCTCCAATCCCCTTCCTTTGGCT-CTCCC	768
71_PHR_APR	CTCCCTATATATACCCCAGGGACTTGGAGCCTTGTGTCCCATCTCCAATCCCCTTCCTTTGGCTACTCCC	798
149_PHR_APR	CTCCCTATATATACCCCAGGGACTTGGAGCCTTGTGTCCCATCTCCAATCCCCTTCCTTTGGCT-CTCCC	779
59_Danko_non-APR	CTCCCTATATATACCCCAGGGACTTGGAGCCTTGTGTCCCATCTCCAATCCCCTTCCTTTGGCTACTCCC	750
61_Danko_non-APR	CTCCCTATATATA-CCCAGGGACTTGGAGCCTTCTGTCCCATCTCCAATCCCCTTCCTTTGGCTGCTCCC	786
123_Danko_non-APR	CTCCCTATATATACCCCAGGGACTTGGAGCCTTGTGTCCCATCTCCAATCCCCTTCCTTTGGCT-CTCCC	730
129_Danko_non-APR	CTCCCTATATATACCCCAGGGACTTGGAGCCTTGTGTCCCATCTCCAATCCCCTTCCTTTGGCT-CTCCC	777
37_PHR_non-APR	CTCCCTATATATACCCCAGGGACTTGGAGCCTTGTGTCCCATCTCCAATCCCCTTCCTTTGGCT-CTCCC	756
52_PHR_non-APR	CTCCCTATATATA-CCCAGGGACTCGGAGCCCTGACTCCCATCTCCAATCCCCTTCCTTTGGCTGCTCCC	775
88_PHR_non-APR	CTCCCTATATATACCCCAGGGACTTGGAGCCTTGTGTCCCATCTCCAATCCCCTTCCTTTGGCT-CTCCC	758
101_PHR_non-APR	CTCCCTATATATACCCCAGGGACTTGGAGCCTTGTGTCCCATCTCCAATCCCCTTCCTTTGGCT-CTCCC	779
105_PHR_non-APR	CTCCCTATATATACCCCAGGGACTTGGAGCCTTGTGTCCCATCTCCAATCCCCTTCCTTTGGCT-CTCCC	778
150_PHR_non-APR	CTCCCTATATATACCCCAGGGACTTGGAGCCTTGTGTCCCATCTCCAATCCCCTTCCTTTGGCT-CTCCC	758
150_PHR_non-APR2	CTCCCTATATATACCCCAGGGACTTGGAGCCTTGTGTCCCATCTCCAATCCCCTTCCTTTGGCT-CTCCC	758

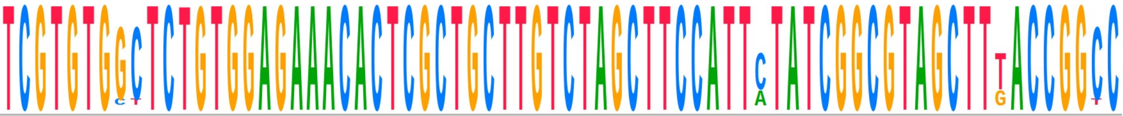

Name	Sequence	End
**Consensus**	TCGTGTGGCTCTGTGGAGAAACACTCGCTGCTTGTCTAGCTTCCATTCTATCGGCGTAGCTTTACCGGCC	
	920 930 940 950 960 970 980	
Lr67(sus)	TCGTGTGCTTCTGTGGAGAAACACTCGCTGCTTGTCTAGCTTCCATTATATCGGCGTAGCTTGACCGGCC	628
Lr67(res)	TCGTGTGCTTCTGTGGAGAAACACTCGCTGCTTGTCTAGCTTCCATTATATCGGCGTAGCTTGACCGGCC	628
ScLr67_1 (Lo7)	TCGTGTGGCTC--TGGAGAAACACTCGCTGCTTGTCTAGCTTCCATTATATCGGCGTAGCTTGACCGGCC	902
118_Danko_APR	TCGTGTGGCTCTGTGGAGAAACACTCGCTGCTTGTCTAGCTTCCATTCTATCGGCGTAGCTTTACCGGCC	851
119_Danko_APR	TCGTGTGGCTCTGTGGAGAAACACTCGCTGCTTGTCTAGCTTCCATTCTATCGGCGTAGCTTTACCGGCC	848
119_Danko_APR2	TCGTGTGGCTCTGTGGAGAAACACTCGCTGCTTGTCTAGCTTCCATTCTATCGGCGTAGCTTTACCGGCC	848
120_Danko_APR	TCGTGTGGCTCTGTGGAGAAACACTCGCTGCTTGTCTAGCTTCCATTCTATCGGCGTAGCTTTACCGGCC	850
138_Danko_APR	TCGTGTGGCTCTGTGGAGAAACACTCGCTGCTTGTCTAGCTTCCATTCTATCGGCGTAGCTTTACCGGCC	846
153_Danko_APR	TCGTGTGGCTCTGTGGAGAAACACTCGCTGCTTGTCTAGCTTCCATTCTATCGGCGTAGCTTTACCGGCC	845
157_Danko_APR	TCGTGTGGCTCTGTGGAGAAACACTCGCTGCTTGTCTAGCTTCCATTCTATCGGCGTAGCTTTACCGGCC	848
160_Danko_APR	TCGTGTGGCTCTGTGGAGAAACACTCGCTGCTTGTCTAGCTTCCATTCTATCGGCGTAGCTTTACCGGCC	838
71_PHR_APR	TCGTGTGGCTCTGTGGAGAAACACTCGCTGCTTGTCTAGCTTCCATTATATCGGCGTAGCTTGACCGGTC	868
149_PHR_APR	TCGTGTGGCTCTGTGGAGAAACACTCGCTGCTTGTCTAGCTTCCATTCTATCGGCGTAGCTTTACCGGCC	849
59_Danko_non-APR	TCGTGTGGCTCTGTGGAGAAACACTCGCTGCTTGTCTAGCTTCCATTATATCGGCGTAGCTTGACCGGTC	820
61_Danko_non-APR	TCGTGTGGCTC--TGGAGAAACACTCGCTGCTTGTCTAGCTTCCATTATATCGGCGTAGCTTGACCGGCC	854
123_Danko_non-APR	TCGTGTGGCTCTGTGGAGAAACACTCGCTGCTTGTCTAGCTTCCATTCTATCGGCGTAGCTTTACCGGCC	800
129_Danko_non-APR	TCGTGTGGCTCTGTGGAGAAACACTCGCTGCTTGTCTAGCTTCCATTCTATCGGCGTAGCTTTACCGGCC	847
37_PHR_non-APR	TCGTGTGGCTCTGTGGAGAAACACTCGCTGCTTGTCTAGCTTCCATTCTATCGGCGTAGCTTTACCGGCC	826
52_PHR_non-APR	TCGTGTGGCTCTGTGGAGAAACACTCGCTGCTTGTCTAGCTTCCATTATATCGGCGTAGCTTGACCGGCC	845
88_PHR_non-APR	TCGTGTGGCTCTGTGGAGAAACACTCGCTGCTTGTCTAGCTTCCATTCTATCGGCGTAGCTTTACCGGCC	828
101_PHR_non-APR	TCGTGTGGCTCTGTGGAGAAACACTCGCTGCTTGTCTAGCTTCCATTCTATCGGCGTAGCTTTACCGGCC	849
105_PHR_non-APR	TCGTGTGGCTCTGTGGAGAAACACTCGCTGCTTGTCTAGCTTCCATTCTATCGGCGTAGCTTTACCGGCC	848
150_PHR_non-APR	TCGTGTGGCTCTGTGGAGAAACACTCGCTGCTTGTCTAGCTTCCATTCTATCGGCGTAGCTTTACCGGCC	828
150_PHR_non-APR2	TCGTGTGGCTCTGTGGAGAAACACTCGCTGCTTGTCTAGCTTCCATTCTATCGGCGTAGCTTTACCGGCC	828

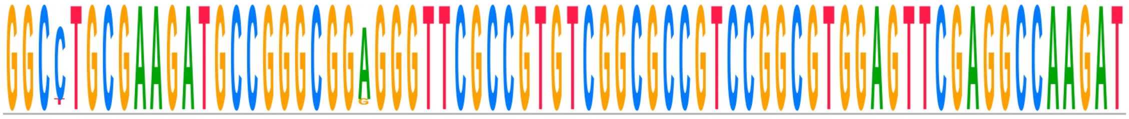

Consensus	GGCCTGCGAAGATGCCGGGCGGAGGGTTCGCCGTGTCGGCGCCGTCCGGCGTGGAGTTCGAGGCCAAGAT	
	990 1000 1010 1020 1030 1040 1050	
Lr67(sus)	GGCTTGCGAAGATGCCGGGCGGGGGGTTCGCCGTGTCGGCGCCGTCCGGCGTGGAGTTCGAGGCCAAGAT	698
Lr67(res)	GGCTTGCGAAGATGCCGGGCGGGGGGTTCGCCGTGTCGGCGCCGTCCGGCGTGGAGTTCGAGGCCAAGAT	698
ScLr67_1 (Lo7)	GGCCTGCGAAGATGCCGGGCGGAGGGTTCGCCGTGTCGGCGCCGTCCGGCGTGGAGTTCGAGGCCAAGAT	972
118_Danko_APR	GGCCTGCGAAGATGCCGGGCGGAGGGTTCGCCGTGTCGGCGCCGTCCGGCGTGGAGTTCGAGGCCAAGAT	921
119_Danko_APR	GGCCTGCGAAGATGCCGGGCGGAGGGTTCGCCGTGTCGGCGCCGTCCGGCGTGGAGTTCGAGGCCAAGAT	918
119_Danko_APR2	GGCCTGCGAAGATGCCGGGCGGAGGGTTCGCCGTGTCGGCGCCGTCCGGCGTGGAGTTCGAGGCCAAGAT	918
120_Danko_APR	GGCCTGCGAAGATGCCGGGCGGAGGGTTCGCCGTGTCGGCGCCGTCCGGCGTGGAGTTCGAGGCCAAGAT	920
138_Danko_APR	GGCCTGCGAAGATGCCGGGCGGAGGGTTCGCCGTGTCGGCGCCGTCCGGCGTGGAGTTCGAGGCCAAGAT	916
153_Danko_APR	GGCCTGCGAAGATGCCGGGCGGAGGGTTCGCCGTGTCGGCGCCGTCCGGCGTGGAGTTCGAGGCCAAGAT	915
157_Danko_APR	GGCCTGCGAAGATGCCGGGCGGAGGGTTCGCCGTGTCGGCGCCGTCCGGCGTGGAGTTCGAGGCCAAGAT	918
160_Danko_APR	GGCCTGCGAAGATGCCGGGCGGAGGGTTCGCCGTGTCGGCGCCGTCCGGCGTGGAGTTCGAGGCCAAGAT	908
71_PHR_APR	GGCCTGCGAAGATGCCGGGCGGAGGGTTCGCCGTGTCGGCGCCGTCCGGCGTGGAGTTCGAGGCCAAGAT	938
149_PHR_APR	GGCCTGCGAAGATGCCGGGCGGAGGGTTCGCCGTGTCGGCGCCGTCCGGCGTGGAGTTCGAGGCCAAGAT	919
59_Danko_non-APR	GGCCTGCGAAGATGCCGGGCGGAGGGTTCGCCGTGTCGGCGCCGTCCGGCGTGGAGTTCGAGGCCAAGAT	890
61_Danko_non-APR	GGCCTGCGAAGATGCCGGGCGGAGGGTTCGCCGTGTCGGCGCCGTCCGGCGTGGAGTTCGAGGCCAAGAT	924
123_Danko_non-APR	GGCCTGCGAAGATGCCGGGCGGAGGGTTCGCCGTGTCGGCGCCGTCCGGCGTGGAGTTCGAGGCCAAGAT	870
129_Danko_non-APR	GGCCTGCGAAGATGCCGGGCGGAGGGTTCGCCGTGTCGGCGCCGTCCGGCGTGGAGTTCGAGGCCAAGAT	917
37_PHR_non-APR	GGCCTGCGAAGATGCCGGGCGGAGGGTTCGCCGTGTCGGCGCCGTCCGGCGTGGAGTTCGAGGCCAAGAT	896
52_PHR_non-APR	GGCCTGCGAAGATGCCGGGCGGAGGGTTCGCCGTGTCGGCGCCGTCCGGCGTGGAGTTCGAGGCCAAGAT	915
88_PHR_non-APR	GGCCTGCGAAGATGCCGGGCGGAGGGTTCGCCGTGTCGGCGCCGTCCGGCGTGGAGTTCGAGGCCAAGAT	898
101_PHR_non-APR	GGCCTGCGAAGATGCCGGGCGGAGGGTTCGCCGTGTCGGCGCCGTCCGGCGTGGAGTTCGAGGCCAAGAT	919
105_PHR_non-APR	GGCCTGCGAAGATGCCGGGCGGAGGGTTCGCCGTGTCGGCGCCGTCCGGCGTGGAGTTCGAGGCCAAGAT	918
150_PHR_non-APR	GGCCTGCGAAGATGCCGGGCGGAGGGTTCGCCGTGTCGGCGCCGTCCGGCGTGGAGTTCGAGGCCAAGAT	898
150_PHR_non-APR2	GGCCTGCGAAGATGCCGGGCGGAGGGTTCGCCGTGTCGGCGCCGTCCGGCGTGGAGTTCGAGGCCAAGAT	898

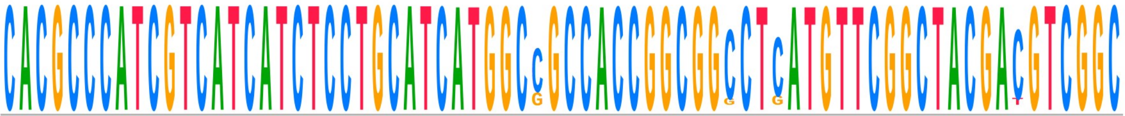

Consensus	CACGCCCATCGTCATCATCTCCTGCATCATGGCCGCCACCGGCGGCCTCATGTTCGGCTACGACGTCGGC	
	1060 1070 1080 1090 1100 1110 1120	
Lr67(sus)	CACGCCCATCGTCATCATCTCCTGCATCATGGCGGCCACCGGCGGCCTCATGTTCGGCTACGACGTCGGC	768
Lr67(res)	CACGCCCATCGTCATCATCTCCTGCATCATGGCGGCCACCGGCGGCCTCATGTTCGGCTACGACGTCGGC	768
ScLr67_1 (Lo7)	CACGCCCATCGTCATCATCTCCTGCATCATGGCGGCCACCGGCGGCCTGATGTTCGGCTACGACGTCGGC	1042
118_Danko_APR	CACGCCCATCGTCATCATCTCCTGCATCATGGCCGCCACCGGCGGCCTCATGTTCGGCTACGACGTCGGC	991
119_Danko_APR	CACGCCCATCGTCATCATCTCCTGCATCATGGCCGCCACCGGCGGCCTCATGTTCGGCTACGACGTCGGC	988
119_Danko_APR2	CACGCCCATCGTCATCATCTCCTGCATCATGGCCGCCACCGGCGGCCTCATGTTCGGCTACGACGTCGGC	988
120_Danko_APR	CACGCCCATCGTCATCATCTCCTGCATCATGGCCGCCACCGGCGGCCTCATGTTCGGCTACGACGTCGGC	990
138_Danko_APR	CACGCCCATCGTCATCATCTCCTGCATCATGGCCGCCACCGGCGGCCTCATGTTCGGCTACGACGTCGGC	986
153_Danko_APR	CACGCCCATCGTCATCATCTCCTGCATCATGGCCGCCACCGGCGGCCTCATGTTCGGCTACGACGTCGGC	985
157_Danko_APR	CACGCCCATCGTCATCATCTCCTGCATCATGGCCGCCACCGGCGGCCTCATGTTCGGCTACGACGTCGGC	988
160_Danko_APR	CACGCCCATCGTCATCATCTCCTGCATCATGGCCGCCACCGGCGGCCTCATGTTCGGCTACGACGTCGGC	978
71_PHR_APR	CACGCCCATCGTCATCATCTCCTGCATCATGGCCGCCACCGGCGGGCTCATGTTCGGCTACGATGTCGGC	1008
149_PHR_APR	CACGCCCATCGTCATCATCTCCTGCATCATGGCCGCCACCGGCGGCCTCATGTTCGGCTACGACGTCGGC	989
59_Danko_non-APR	CACGCCCATCGTCATCATCTCCTGCATCATGGCCGCCACCGGCGGGCTCATGTTCGGCTACGATGTCGGC	960
61_Danko_non-APR	CACGCCCATCGTCATCATCTCCTGCATCATGGCGGCCACCGGCGGCCTGATGTTCGGCTACGACGTCGGC	994
123_Danko_non-APR	CACGCCCATCGTCATCATCTCCTGCATCATGGCCGCCACCGGCGGCCTCATGTTCGGCTACGACGTCGGC	940
129_Danko_non-APR	CACGCCCATCGTCATCATCTCCTGCATCATGGCCGCCACCGGCGGCCTCATGTTCGGCTACGACGTCGGC	987
37_PHR_non-APR	CACGCCCATCGTCATCATCTCCTGCATCATGGCCGCCACCGGCGGCCTCATGTTCGGCTACGACGTCGGC	966
52_PHR_non-APR	CACGCCCATCGTCATCATCTCCTGCATCATGGCGGCCACCGGCGGCCTGATGTTCGGCTACGACGTCGGC	985
88_PHR_non-APR	CACGCCCATCGTCATCATCTCCTGCATCATGGCCGCCACCGGCGGCCTCATGTTCGGCTACGACGTCGGC	968
101_PHR_non-APR	CACGCCCATCGTCATCATCTCCTGCATCATGGCCGCCACCGGCGGCCTCATGTTCGGCTACGACGTCGGC	989
105_PHR_non-APR	CACGCCCATCGTCATCATCTCCTGCATCATGGCCGCCACCGGCGGCCTCATGTTCGGCTACGACGTCGGC	988
150_PHR_non-APR	CACGCCCATCGTCATCATCTCCTGCAT--------------------------------------TCGGC	930
150_PHR_non-APR2	CACGCCCATCGTCATCATCTCCTGCAT--------------------------------------TCGGC	930

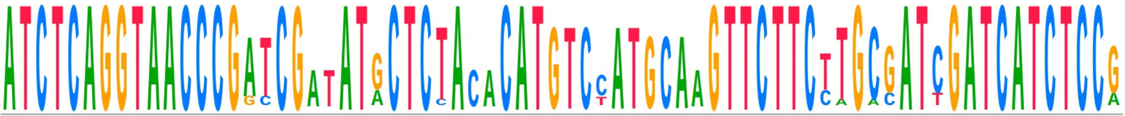

```
Consensus            ATCTCAGGTAACCCGATCG--ATGCTCTA--CAT----------GTTCTTCTTGCGATCGATCATCTCCG
                             1130      1140      1150      1160      1170      1180      1190
Lr67(sus)            ATCTCAGGTAACCCG------ATACTCCA--CATGTCTATGCAAGTTCTTCCAGAGATCGATCATCTCCG  830
Lr67(res)            ATCTCAGGTAACCCG------ATACTCCA--CATGTCTATGCAAGTTCTTCCAGAGATCGATCATCTCCG  830
ScLr67_1 (Lo7)       ATCTCAGGTAACCCGGCCG--ATACTCTA--CATGTCCATGCA-GTTCTTCCTGCCATTGATCATCTCCA  1107
118_Danko_APR        ATCTCAGGTAACCCGATCG--ATGCTCTA--CAT----------GTTCTTCTTGCGATCGATCATCTCCG  1047
119_Danko_APR        ATCTCAGGTAACCCGATCG--ATGCTCTA--CAT----------GTTCTTCTTGCGATCGATCATCTCCG  1044
119_Danko_APR2       ATCTCAGGTAACCCGATCG--ATGCTCTA--CAT----------GTTCTTCTTGCGATCGATCATCTCCG  1044
120_Danko_APR        ATCTCAGGTAACCCGATCG--ATGCTCTA--CAT----------GTTCTTCTTGCGATCGATCATCTCCG  1046
138_Danko_APR        ATCTCAGGTAACCCGATCG--ATGCTCTA--CAT----------GTTCTTCTTGCGATCGATCATCTCCG  1042
153_Danko_APR        ATCTCAGGTAACCCGATCG--ATGCTCTA--CAT----------GTTCTTCTTGCGATCGATCATCTCCG  1041
157_Danko_APR        ATCTCAGGTAACCCGATCG--ATGCTCTA--CAT----------GTTCTTCTTGCGATCGATCATCTCCG  1044
160_Danko_APR        ATCTCAGGTAACCCGATCG--ATGCTCTA--CAT----------GTTCTTCTTGCGATCGATCATCTCCG  1034
71_PHR_APR           ATCTCAGGTAACCCGATCGATATACTCTACACATGTCCAT----GTTCTTCCTGCCATTGATCATCTCCA  1074
149_PHR_APR          ATCTCAGGTAACCCGATCG--ATGCTCTA--CAT----------GTTCTTCTTGCGATCGATCATCTCCG  1045
59_Danko_non-APR     ATCTCAGGTAACCCGATCGATATACTCTACACATGTCCAT----GTTCTTCCTGCCATTGATCATCTCCA  1026
61_Danko_non-APR     ATCTCAGGTAACCCGGCCG--ATACTCTA--CATGTCCATGCA-GTTCTTCCTGCCATTGATCATCTCCA  1059
123_Danko_non-APR    ATCTCAGGTAACCCGATCG--ATGCTCTA--CAT----------GTTCTTCTTGCGATCGATCATCTCCG  996
129_Danko_non-APR    ATCTCAGGTAACCCGATCG--ATGCTCTA--CAT----------GTTCTTCTTGCGATCGATCATCTCCG  1043
37_PHR_non-APR       ATCTCAGGTAACCCGATCG--ATGCTCTA--CAT----------GTTCTTCTTGCGATCGATCATCTCCG  1022
52_PHR_non-APR       ATCTCAGGTAACCCGGCCG--ATACTCTA--CATGTCCATGCA-GTTCTTCCTGCCATTGATCATCTCCA  1050
88_PHR_non-APR       ATCTCAGGTAACCCGATCG--ATGCTCTA--CAT----------GTTCTTCTTGCGATCGATCATCTCCG  1024
101_PHR_non-APR      ATCTCAGGTAACCCGATCG--ATGCTCTA--CAT----------GTTCTTCTTGCGATCGATCATCTCCG  1045
105_PHR_non-APR      ATCTCAGGTAACCCGATCG--ATGCTCTA--CAT----------GTTCTTCTTGCGATCGATCATCTCCG  1044
150_PHR_non-APR      ATCTCAGGTAACCCGATCG--ATGCTCTA--CAT----------GTTCTTCTTGCGATCGATCATCTCCG  986
150_PHR_non-APR2     ATCTCAGGTAACCCGATCG--ATGCTCTA--CAT----------GTTCTTCTTGCGATCGATCATCTCCG  986
```

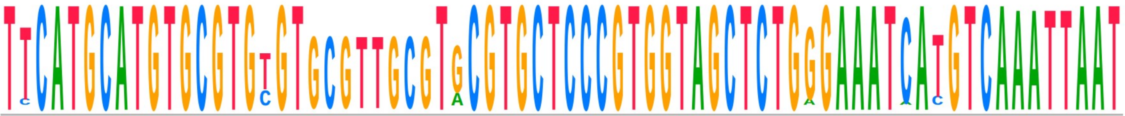

```
Consensus            TTCATGCATGTGCGTGTGT--------TGCGTGCTCCCGTGGTAGCTCTGGGAAATCATGTCAAATTAAT
                             1200      1210      1220      1230      1240      1250      1260
Lr67(sus)            TCCATGCATGTGCGTGCGT--------TGCGTGCTCCCGTGGTAGCTCTGAGAAATCATGTCA----AAT  888
Lr67(res)            TCCATGCATGTGCGTGCGT--------TGCGTGCTCCCGTGGTAGCTCTGAGAAATCATGTCA----AAT  888
ScLr67_1 (Lo7)       TTCATGCATGTGCGTGCGTGCGTTGCGTACGTGCTCCCGTGGTAGCTCTGGGAAATCATGTCA----AAT  1173
118_Danko_APR        TTCATGCATGTGCGTGTGT--------TGCGTGCTCCCGTGGTAGCTCTGGGAAATCATGTCAAATTAAT  1109
119_Danko_APR        TTCATGCATGTGCGTGTGT--------TGCGTGCTCCCGTGGTAGCTCTGGGAAATCATGTCAAATTAAT  1106
119_Danko_APR2       TTCATGCATGTGCGTGTGT--------TGCGTGCTCCCGTGGTAGCTCTGGGAAATCATGTCAAATTAAT  1106
120_Danko_APR        TTCATGCATGTGCGTGTGT--------TGCGTGCTCCCGTGGTAGCTCTGGGAAATCATGTCAAATTAAT  1108
138_Danko_APR        TTCATGCATGTGCGTGTGT--------TGCGTGCTCCCGTGGTAGCTCTGGGAAATCATGTCAAATTAAT  1104
153_Danko_APR        TTCATGCATGTGCGTGTGT--------TGCGTGCTCCCGTGGTAGCTCTGGGAAATCATGTCAAATTAAT  1103
157_Danko_APR        TTCATGCATGTGCGTGTGT--------TGCGTGCTCCCGTGGTAGCTCTGGGAAATCATGTCAAATTAAT  1106
160_Danko_APR        TTCATGCATGTGCGTGTGT--------TGCGTGCTCCCGTGGTAGCTCTGGGAAATCATGTCAAATTAAT  1096
71_PHR_APR           TTCATGCATGTGCGTGCGTGCGTTGCGTACGTGCTCCCGTGGTAGCTCTGGGAAATCACGTCA----AAT  1140
149_PHR_APR          TTCATGCATGTGCGTGTGT--------TGCGTGCTCCCGTGGTAGCTCTGGGAAATCATGTCAAATTAAT  1107
59_Danko_non-APR     TTCATGCATGTGCGTGCGTGCGTTGCGTACGTGCTCCCGTGGTAGCTCTGGGAAATCACGTCA----AAT  1092
61_Danko_non-APR     TTCATGCATGTGCGTGCGTGCGTTGCGTACGTGCTCCCGTGGTAGCTCTGGGAAATCATGTCA----AAT  1125
123_Danko_non-APR    TTCATGCATGTGCGTGTGT--------TGCGTGCTCCCGTGGTAGCTCTGGGAAATCATGTCAAATTAAT  1058
129_Danko_non-APR    TTCATGCATGTGCGTGTGT--------TGCGTGCTCCCGTGGTAGCTCTGGGAAATCATGTCAAATTAAT  1105
37_PHR_non-APR       TTCATGCATGTGCGTGTGT--------TGCGTGCTCCCGTGGTAGCTCTGGGAAATCATGTCAAATTAAT  1084
52_PHR_non-APR       TTCATGCATGTGCGTGCGTGCGTTGCGTACGTGCTCCCGTGGTAGCTCTGGGAAATAACGTCA----AAT  1116
88_PHR_non-APR       TTCATGCATGTGCGTGTGT--------TGCGTGCTCCCGTGGTAGCTCTGGGAAATCATGTCAAATTAAT  1086
101_PHR_non-APR      TTCATGCATGTGCGTGTGT--------TGCGTGCTCCCGTGGTAGCTCTGGGAAATCATGTCAAATTAAT  1107
105_PHR_non-APR      TTCATGCATGTGCGTGTGT--------TGCGTGCTCCCGTGGTAGCTCTGGGAAATCATGTCAAATTAAT  1106
150_PHR_non-APR      TTCATGCATGTGCGTGTGT--------TGCGTGCTCCCGTGGTAGCTCTGGGAAATCATGTCAAATTAAT  1048
150_PHR_non-APR2     TTCATGCATGTGCGTGTGT--------TGCGTGCTCCCGTGGTAGCTCTGGGAAATCATGTCAAATTAAT  1048
```

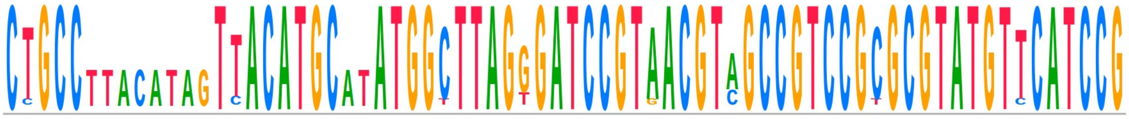

```
Consensus            CTGCC--------TTACATGC--ATGGCTTAGGGATCCGTAACGTAGCCGTCCGCGCGTATGTTCATCCG
                        1270      1280      1290      1300      1310      1320      1330
Lr67(sus)            CCGCC--------TTACATGC--ATGGTTTAGTGATCCGTGACGTCGCCGTCCGCGCGTATG--------  940
Lr67(res)            CCGCC--------TTACATGC--ATGGTTTAGTGATCCGTGACGTCGCCGTCCGCGCGTATG--------  940
ScLr67_1 (Lo7)       CTGCCTTACATAGTTACATGC--ATGGCTTAGGGATCCGTAACGTCGCCGTCCGTGCGTATGTTCATCCG  1241
118_Danko_APR        CTGCC--------TTACATGC--ATGGCTTAGGGATCCGTAACGTAGCCGTCCGCGCGTATGTTCATCCG  1169
119_Danko_APR        CTGCC--------TTACATGC--ATGGCTTAGGGATCCGTAACGTAGCCGTCCGCGCGTATGTTCATCCG  1166
119_Danko_APR2       CTGCC--------TTACATGC--ATGGCTTAGGGATCCGTAACGTAGCCGTCCGCGCGTATGTTCATCCG  1166
120_Danko_APR        CTGCC--------TTACATGC--ATGGCTTAGGGATCCGTAACGTAGCCGTCCGCGCGTATGTTCATCCG  1168
138_Danko_APR        CTGCC--------TTACATGC--ATGGCTTAGGGATCCGTAACGTAGCCGTCCGCGCGTATGTTCATCCG  1164
153_Danko_APR        CTGCC--------TTACATGC--ATGGCTTAGGGATCCGTAACGTAGCCGTCCGCGCGTATGTTCATCCG  1163
157_Danko_APR        CTGCC--------TTACATGC--ATGGCTTAGGGATCCGTAACGTAGCCGTCCGCGCGTATGTTCATCCG  1166
160_Danko_APR        CTGCC--------TTACATGC--ATGGCTTAGGGATCCGTAACGTAGCCGTCCGCGCGTATGTTCATCCG  1156
71_PHR_APR           CTGCC--------TCACATGCATATGGCTTAGTGATCCGTAACGTCGCCGTCCGCGCGTATGTCCATCCG  1202
149_PHR_APR          CTGCC--------TTACATGC--ATGGCTTAGGGATCCGTAACGTAGCCGTCCGCGCGTATGTTCATCCG  1167
59_Danko_non-APR     CTGCC--------TCACATGCATATGGCTTAGTGATCCGTAACGTCGCCGTCCGCGCGTATGTCCATCCG  1154
61_Danko_non-APR     CTGCCTTACATAGTTACATGC--ATGGCTTAGGGATCCGTAACGTCGCCGTCCGTGCGTATGTTCATCCG  1193
123_Danko_non-APR    CTGCC--------TTACATGC--ATGGCTTAGGGATCCGTAACGTAGCCGTCCGCGCGTATGTTCATCCG  1118
129_Danko_non-APR    CTGCC--------TTACATGC--ATGGCTTAGGGATCCGTAACGTAGCCGTCCGCGCGTATGTTCATCCG  1165
37_PHR_non-APR       CTGCC--------TTACATGC--ATGGCTTAGGGATCCGTAACGTAGCCGTCCGCGCGTATGTTCATCCG  1144
52_PHR_non-APR       CTGCC--------TCACATGC--ATGGCTTAGTGATCCGTAACGTCGCCGTCCGCGCGTATGTTCATCCG  1176
88_PHR_non-APR       CTGCC--------TTACATGC--ATGGCTTAGGGATCCGTAACGTAGCCGTCCGCGCGTATGTTCATCCG  1146
101_PHR_non-APR      CTGCC--------TTACATGC--ATGGCTTAGGGATCCGTAACGTAGCCGTCCGCGCGTATGTTCATCCG  1167
105_PHR_non-APR      CTGCC--------TTACATGC--ATGGCTTAGGGATCCGTAACGTAGCCGTCCGCGCGTATGTTCATCCG  1166
150_PHR_non-APR      CTGCC--------TTACATGC--ATGGCTTAGGGATCCGTAACGTAGCCGTCCGCGCGTATGTTCATCCG  1108
150_PHR_non-APR2     CTGCC--------TTACATGC--ATGGCTTAGGGATCCGTAACGTAGCCGTCCGCGCGTATGTTCATCCG  1108
```

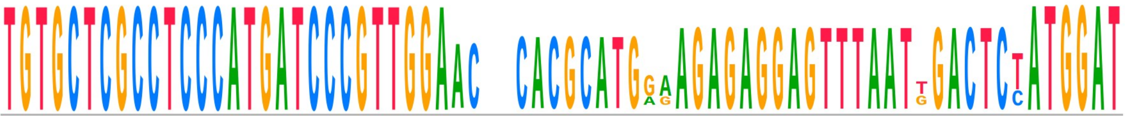

```
Consensus            TGTGCTCGCCTCCCATGATCCCGTTGGA-----------------------------------TATGGAT
                          1340      1350      1360      1370      1380      1390      1400
Lr67(sus)            ----------------------------------------AAAGAGAGGAGTTTAATTGACTCCATGGAT  970
Lr67(res)            ----------------------------------------AAAGAGAGGAGTTTAATTGACTCCATGGAT  970
ScLr67_1 (Lo7)       TGTGCTCGCCTCCCATGATCCCGTTGGAACCACACGCATGGGAGAGAGGAGTTTAATTGACTCCATGGAT  1311
118_Danko_APR        TGTGCTCGCCTCCCATGATCCCGTTGGA-----------------------------------TATGGAT  1204
119_Danko_APR        TGTGCTCGCCTCCCATGATCCCGTTGGA-----------------------------------TATGGAT  1201
119_Danko_APR2       TGTGCTCGCCTCCCATGATCCCGTTGGA-----------------------------------TATGGAT  1201
120_Danko_APR        TGTGCTCGCCTCCCATGATCCCGTTGGA-----------------------------------TATGGAT  1203
138_Danko_APR        TGTGCTCGCCTCCCATGATCCCGTTGGA-----------------------------------TATGGAT  1199
153_Danko_APR        TGTGCTCGCCTCCCATGATCCCGTTGGA-----------------------------------TATGGAT  1198
157_Danko_APR        TGTGCTCGCCTCCCATGATCCCGTTGGA-----------------------------------TATGGAT  1201
160_Danko_APR        TGTGCTCGCCTCCCATGATCCCGTTGGA-----------------------------------TATGGAT  1191
71_PHR_APR           TGTGCTCGCCTCCCATGATCCCGTTGGA--ACCACGCATGGAAGAGAGGAGTTTAATGGACTCCATGGAT  1270
149_PHR_APR          TGTGCTCGCCTCCCATGATCCCGTTGGA-----------------------------------TATGGAT  1202
59_Danko_non-APR     TGTGCTCGCCTCCCATGATCCCGTTGGAAC--CACGCATGGAAGAGAGGAGTTTAATGGACTCCATGGAT  1222
61_Danko_non-APR     TGTGCTCGCCTCCCATGATCCCGTTGGAACCACACGCATGGGAGAGAGGAGTTTAATTGACTCCATGGAT  1263
123_Danko_non-APR    TGTGCTCGCCTCCCATGATCCCGTTGGA-----------------------------------TATGGAT  1153
129_Danko_non-APR    TGTGCTCGCCTCCCATGATCCCGTTGGA-----------------------------------TATGGAT  1200
37_PHR_non-APR       TGTGCTCGCCTCCCATGATCCCGTTGGA-----------------------------------TATGGAT  1179
52_PHR_non-APR       TGTGCTCGCCTCCCATGATCCCGTTGGAAC--CACGCATGGAAGAGAGGAGTTTAATGGACTCCATGGAT  1244
88_PHR_non-APR       TGTGCTCGCCTCCCATGATCCCGTTGGA-----------------------------------TATGGAT  1181
101_PHR_non-APR      TGTGCTCGCCTCCCATGATCCCGTTGGA-----------------------------------TATGGAT  1202
105_PHR_non-APR      TGTGCTCGCCTCCCATGATCCCGTTGGA-----------------------------------TATGGAT  1201
150_PHR_non-APR      TGTGCTCGCCTCCCATGATCCCGTTGGA-----------------------------------TATGGAT  1143
150_PHR_non-APR2     TGTGCTCGCCTCCCATGATCCCGTTGGA-----------------------------------TATGGAT  1143
```

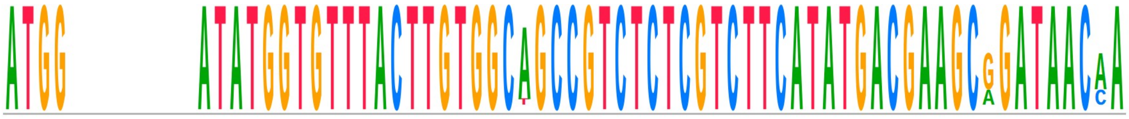

```
Consensus            ATGG--------ATATGGTGTTTACTTGTGGCAGCCGTCTCTCGTCTTCATATGACGAAGCGGATAACAA
                              1410      1420      1430      1440      1450      1460      1470
Lr67(sus)            ATGG--------ATATGGTGTTTACTTGTGGCTGCCGTCTCTCGTCTTCATATGACGAAGCAGATAACCA  1032
Lr67(res)            ATGG--------ATATGGTGTTTACTTGTGGCTGCCGTCTCTCGTCTTCATATGACGAAGCAGATAACCA  1032
ScLr67_1 (Lo7)       ATGG--------ATATGGTGTTTACTTGTGGCAGCCGTCTCTCGTCTTCATATGACGAAGCAGATAACCA  1373
118_Danko_APR        ATGG--------ATATGGTGTTTACTTGTGGCAGCCGTCTCTCGTCTTCATATGACGAAGCGGATAACAA  1266
119_Danko_APR        ATGG--------ATATGGTGTTTACTTGTGGCAGCCGTCTCTCGTCTTCATATGACGAAGCGGATAACAA  1263
119_Danko_APR2       ATGG--------ATATGGTGTTTACTTGTGGCAGCCGTCTCTCGTCTTCATATGACGAAGCGGATAACAA  1263
120_Danko_APR        ATGG--------ATATGGTGTTTACTTGTGGCAGCCGTCTCTCGTCTTCATATGACGAAGCGGATAACAA  1265
138_Danko_APR        ATGG--------ATATGGTGTTTACTTGTGGCAGCCGTCTCTCGTCTTCATATGACGAAGCGGATAACAA  1261
153_Danko_APR        ATGG--------ATATGGTGTTTACTTGTGGCAGCCGTCTCTCGTCTTCATATGACGAAGCGGATAACAA  1260
157_Danko_APR        ATGG--------ATATGGTGTTTACTTGTGGCAGCCGTCTCTCGTCTTCATATGACGAAGCGGATAACAA  1263
160_Danko_APR        ATGG--------ATATGGTGTTTACTTGTGGCAGCCGTCTCTCGTCTTCATATGACGAAGCGGATAACAA  1253
71_PHR_APR           ATGG--------ATATGGTGTTTACTTGTGGCAGCCGTCTCTCGTCTTCATATGACGAAGCAGATAACCA  1332
149_PHR_APR          ATGG--------ATATGGTGTTTACTTGTGGCAGCCGTCTCTCGTCTTCATATGACGAAGCGGATAACAA  1264
59_Danko_non-APR     ATGG--------ATATGGTGTTTACTTGTGGCAGCCGTCTCTCGTCTTCATATGACGAAGCAGATAACCA  1284
61_Danko_non-APR     ATGG--------ATATGGTGTTTACTTGTGGCAGCCGTCTCTCGTCTTCATATGACGAAGCAGATAACCA  1325
123_Danko_non-APR    ATGG--------ATATGGTGTTTACTTGTGGCAGCCGTCTCTCGTCTTCATATGACGAAGCGGATAACAA  1215
129_Danko_non-APR    ATGG--------ATATGGTGTTTACTTGTGGCAGCCGTCTCTCGTCTTCATATGACGAAGCGGATAACAA  1262
37_PHR_non-APR       ATGG--------ATATGGTGTTTACTTGTGGCAGCCGTCTCTCGTCTTCATATGACGAAGCGGATAACAA  1241
52_PHR_non-APR       ATGGATATGGACATATGGTGTTTACTTGTGGCAGCCGTCTCTCGTCTTCATATGACGAAGCAGATAACCA  1314
88_PHR_non-APR       ATGG--------ATATGGTGTTTACTTGTGGCAGCCGTCTCTCGTCTTCATATGACGAAGCGGATAACAA  1243
101_PHR_non-APR      ATGG--------ATATGGTGTTTACTTGTGGCAGCCGTCTCTCGTCTTCATATGACGAAGCGGATAACAA  1264
105_PHR_non-APR      ATGG--------ATATGGTGTTTACTTGTGGCAGCCGTCTCTCGTCTTCATATGACGAAGCGGATAACAA  1263
150_PHR_non-APR      ATGG--------ATATGGTGTTTACTTGTGGCAGCCGTCTCTCGTCTTCATATGACGAAGCGGATAACAA  1205
150_PHR_non-APR2     ATGG--------ATATGGTGTTTACTTGTGGCAGCCGTCTCTCGTCTTCATATGACGAAGCGGATAACAA  1205
```

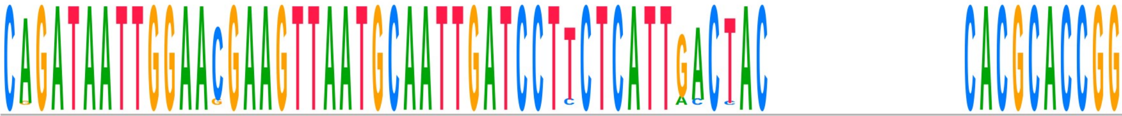

```
Consensus            CAGATAATTGGAACGAAGTTAATGCAATTGATCCTTCTCATTGACTAC------------CACGCACCGG
                              1480      1490      1500      1510      1520      1530      1540
Lr67(sus)            CAGATAATTGGAAGGAAGTTAATGCAATTGATCCTCCTCATTAACTAC------------CACGCACCGG  1090
Lr67(res)            CAGATAATTGGAAGGAAGTTAATGCAATTGATCCTCCTCATTAACTAC------------CACGCACCGG  1090
ScLr67_1 (Lo7)       CAGATAATTGGAACGAAGTTAATGCAATTGATCCTTCTCATTGACTAC------------CACGCACCGG  1431
118_Danko_APR        CAGATAATTGGAACGAAGTTAATGCAATTGATCCTTCTCATTGACTAC------------CACGCACCGG  1324
119_Danko_APR        CAGATAATTGGAACGAAGTTAATGCAATTGATCCTTCTCATTGACTAC------------CACGCACCGG  1321
119_Danko_APR2       CAGATAATTGGAACGAAGTTAATGCAATTGATCCTTCTCATTGACTAC------------CACGCACCGG  1321
120_Danko_APR        CAGATAATTGGAACGAAGTTAATGCAATTGATCCTTCTCATTGACTAC------------CACGCACCGG  1323
138_Danko_APR        CAGATAATTGGAACGAAGTTAATGCAATTGATCCTTCTCATTGACTAC------------CACGCACCGG  1319
153_Danko_APR        CAGATAATTGGAACGAAGTTAATGCAATTGATCCTTCTCATTGACTAC------------CACGCACCGG  1318
157_Danko_APR        CAGATAATTGGAACGAAGTTAATGCAATTGATCCTTCTCATTGACTAC------------CACGCACCGG  1321
160_Danko_APR        CAGATAATTGGAACGAAGTTAATGCAATTGATCCTTCTCATTGACTAC------------CACGCACCGG  1311
71_PHR_APR           CAGATAATTGGAACGAAGTTAATGCAATTGATCCTTCTCATTGCCTAC------------CACGCACCGG  1390
149_PHR_APR          CAGATAATTGGAACGAAGTTAATGCAATTGATCCTTCTCATTGACTAC------------CACGCACCGG  1322
59_Danko_non-APR     CAGATAATTGGAACGAAGTTAATGCAATTGATCCTTCTCATTGCCTAC------------CACGCACCGG  1342
61_Danko_non-APR     CAGATAATTGGAACGAAGTTAATGCAATTGATCCTTCTCATTGACTAC------------CACGCACCGG  1383
123_Danko_non-APR    CAGATAATTGGAACGAAGTTAATGCAATTGATCCTTCTCATTGACTAC------------CACGCACCGG  1273
129_Danko_non-APR    CAGATAATTGGAACGAAGTTAATGCAATTGATCCTTCTCATTGACTAC------------CACGCACCGG  1320
37_PHR_non-APR       CAGATAATTGGAACGAAGTTAATGCAATTGATCCTTCTCATTGACTAC------------CACGCACCGG  1299
52_PHR_non-APR       CGGATAATTGGAACGAAGTTAATGCAATTGATCCTTCTCATTAACCACATATAATTGTAGCACGCACCGG  1384
88_PHR_non-APR       CAGATAATTGGAACGAAGTTAATGCAATTGATCCTTCTCATTGACTAC------------CACGCACCGG  1301
101_PHR_non-APR      CAGATAATTGGAACGAAGTTAATGCAATTGATCCTTCTCATTGACTAC------------CACGCACCGG  1322
105_PHR_non-APR      CAGATAATTGGAACGAAGTTAATGCAATTGATCCTTCTCATTGACTAC------------CACGCACCGG  1321
150_PHR_non-APR      CAGATAATTGGAACGAAGTTAATGCAATTGATCCTTCTCATTGACTAC------------CACGCACCGG  1263
150_PHR_non-APR2     CAGATAATTGGAACGAAGTTAATGCAATTGATCCTTCTCATTGACTAC------------CACGCACCGG  1263
```

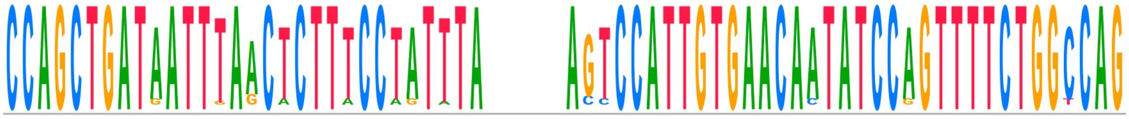

```
Consensus            CCAGCTGATAATTTAACTCTTTCCTATTTA-----AGTCCATTGTGAACAATATCCAGTTTTCTGGCCAG
                         1550      1560      1570      1580      1590      1600      1610
Lr67(sus)            CCAGCTGATAATTTAGCACTTTCCAGTTT------ACTCCATTGTGAACACTATCCGGTTTTCTGGCCAG  1154
Lr67(res)            CCAGCTGATAATTTAGCACTTTCCAGTTT------ACTCCATTGTGAACACTATCCGGTTTTCTGGCCAG  1154
ScLr67_1 (Lo7)       CCAGCTGATGATTTAACTCTTACCTATTTA-----AGCCCATTGTGAACAATATCCAGTTTTCTGGCCAG  1496
118_Danko_APR        CCAGCTGATAATTTAACTCTTTCCTATTTA-----AGTCCATTGTGAACAATATCCAGTTTTCTGGCCAG  1389
119_Danko_APR        CCAGCTGATAATTTAACTCTTTCCTATTTA-----AGTCCATTGTGAACAATATCCAGTTTTCTGGCCAG  1386
119_Danko_APR2       CCAGCTGATAATTTAACTCTTTCCTATTTA-----AGTCCATTGTGAACAATATCCAGTTTTCTGGCCAG  1386
120_Danko_APR        CCAGCTGATAATTTAACTCTTTCCTATTTA-----AGTCCATTGTGAACAATATCCAGTTTTCTGGCCAG  1388
138_Danko_APR        CCAGCTGATAATTTAACTCTTTCCTATTTA-----AGTCCATTGTGAACAATATCCAGTTTTCTGGCCAG  1384
153_Danko_APR        CCAGCTGATAATTTAACTCTTTCCTATTTA-----AGTCCATTGTGAACAATATCCAGTTTTCTGGCCAG  1383
157_Danko_APR        CCAGCTGATAATTTAACTCTTTCCTATTTA-----AGTCCATTGTGAACAATATCCAGTTTTCTGGCCAG  1386
160_Danko_APR        CCAGCTGATAATTTAACTCTTTCCTATTTA-----AGTCCATTGTGAACAATATCCAGTTTTCTGGCCAG  1376
71_PHR_APR           CCAGCTGATAATTTAGCTCTTTCCTATTTA-----AGTCCATTGTGAACAATATCCAGTTTTCTGGTCAG  1455
149_PHR_APR          CCAGCTGATAATTTAACTCTTTCCTATTTA-----AGTCCATTGTGAACAATATCCAGTTTTCTGGCCAG  1387
59_Danko_non-APR     CCAGCTGATAATTTAGCTCTTTCCTATTTA-----AGTCCATTGTGAACAATATCCAGTTTTCTGGTCAG  1407
61_Danko_non-APR     CCAGCTGATGATTTAACTCTTACCTATTTA-----AGCCCATTGTGAACAATATCCAGTTTTCTGGCCAG  1448
123_Danko_non-APR    CCAGCTGATAATTTAACTCTTTCCTATTTA-----AGTCCATTGTGAACAATATCCAGTTTTCTGGCCAG  1338
129_Danko_non-APR    CCAGCTGATAATTTAACTCTTTCCTATTTA-----AGTCCATTGTGAACAATATCCAGTTTTCTGGCCAG  1385
37_PHR_non-APR       CCAGCTGATAATTTAACTCTTTCCTATTTA-----AGTCCATTGTGAACAATATCCAGTTTTCTGGCCAG  1364
52_PHR_non-APR       CCAGCTGATAATTGAACTCTTTCCTATATATTTATACTCCATTGTGAACAATATCCAGTTTTCTGGCCAG  1454
88_PHR_non-APR       CCAGCTGATAATTTAACTCTTTCCTATTTA-----AGTCCATTGTGAACAATATCCAGTTTTCTGGCCAG  1366
101_PHR_non-APR      CCAGCTGATAATTTAACTCTTTCCTATTTA-----AGTCCATTGTGAACAATATCCAGTTTTCTGGCCAG  1387
105_PHR_non-APR      CCAGCTGATAATTTAACTCTTTCCTATTTA-----AGTCCATTGTGAACAATATCCAGTTTTCTGGCCAG  1386
150_PHR_non-APR      CCAGCTGATAATTTAACTCTTTCCTATTTA-----AGTCCATTGTGAACAATATCCAGTTTTCTGGCCAG  1328
150_PHR_non-APR2     CCAGCTGATAATTTAACTCTTTCCTATTTA-----AGTCCATTGTGAACAATATCCAGTTTTCTGGCCAG  1328
```

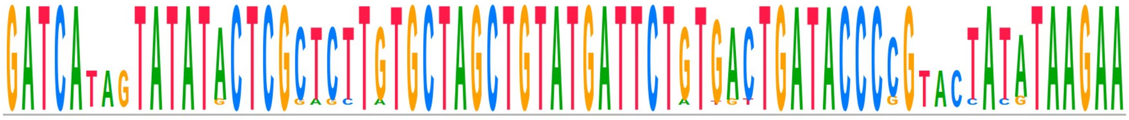

```
Consensus            GATCA---TATATACTCGCTCTTGTGCTAGCTGTATGATTCTGTGACTGATACCCCG---TATATAAGAA
                           1620      1630      1640      1650      1660      1670      1680
Lr67(sus)            GATCATAGTATATGCTCGCACTTGTGCTAGCTGTATGATTCTA--GCTG-----------CATATAAGAA  1211
Lr67(res)            GATCATAGTATATGCTCGCACTTGTGCTAGCTGTATGATTCTA--GCTG-----------CATATAAGAA  1211
ScLr67_1 (Lo7)       GATCA---TATATACTCGGTGCTA-GCTAGCTGTATGATTCTGTGACTGATACCCGGTACTACGTAAGAA  1562
118_Danko_APR        GATCA---TATATACTCGCTCTTGTGCTAGCTGTATGATTCTGTGACTGATACCCCG---TATATAAGAA  1453
119_Danko_APR        GATCA---TATATACTCGCTCTTGTGCTAGCTGTATGATTCTGTGACTGATACCCCG---TATATAAGAA  1450
119_Danko_APR2       GATCA---TATATACTCGCTCTTGTGCTAGCTGTATGATTCTGTGACTGATACCCCG---TATATAAGAA  1450
120_Danko_APR        GATCA---TATATACTCGCTCTTGTGCTAGCTGTATGATTCTGTGACTGATACCCCG---TATATAAGAA  1452
138_Danko_APR        GATCA---TATATACTCGCTCTTGTGCTAGCTGTATGATTCTGTGACTGATACCCCG---TATATAAGAA  1448
153_Danko_APR        GATCA---TATATACTCGCTCTTGTGCTAGCTGTATGATTCTGTGACTGATACCCCG---TATATAAGAA  1447
157_Danko_APR        GATCA---TATATACTCGCTCTTGTGCTAGCTGTATGATTCTGTGACTGATACCCCG---TATATAAGAA  1450
160_Danko_APR        GATCA---TATATACTCGCTCTTGTGCTAGCTGTATGATTCTGTGACTGATACCCCG---TATATAAGAA  1440
71_PHR_APR           GATCA---TATATACTCGCTCTTGTGCTAGCTGTATGATTCTGTGATTGATACCCCG---TATATAAGAA  1519
149_PHR_APR          GATCA---TATATACTCGCTCTTGTGCTAGCTGTATGATTCTGTGACTGATACCCCG---TATATAAGAA  1451
59_Danko_non-APR     GATCA---TATATACTCGCTCTTGTGCTAGCTGTATGATTCTGTGATTGATACCCCG---TATATAAGAA  1471
61_Danko_non-APR     GATCA---TATATACTCGGTGCTA-GCTAGCTGTATGATTCTGTGACTGATACCCGGTACTACGTAAGAA  1514
123_Danko_non-APR    GATCA---TATATACTCGCTCTTGTGCTAGCTGTATGATTCTGTGACTGATACCCCG---TATATAAGAA  1402
129_Danko_non-APR    GATCA---TATATACTCGCTCTTGTGCTAGCTGTATGATTCTGTGACTGATACCCCG---TATATAAGAA  1449
37_PHR_non-APR       GATCA---TATATACTCGCTCTTGTGCTAGCTGTATGATTCTGTGACTGATACCCCG---TATATAAGAA  1428
52_PHR_non-APR       GATCA---TATATACTCGCTCTTGTGCTAGCTGTATGATTCTGTTACTGATACCCGG---TATGTAAGAA  1518
88_PHR_non-APR       GATCA---TATATACTCGCTCTTGTGCTAGCTGTATGATTCTGTGACTGATACCCCG---TATATAAGAA  1430
101_PHR_non-APR      GATCA---TATATACTCGCTCTTGTGCTAGCTGTATGATTCTGTGACTGATACCCCG---TATATAAGAA  1451
105_PHR_non-APR      GATCA---TATATACTCGCTCTTGTGCTAGCTGTATGATTCTGTGACTGATACCCCG---TATATAAGAA  1450
150_PHR_non-APR      GATCA---TATATACTCGCTCTTGTGCTAGCTGTATGATTCTGTGACTGATACCCCG---TATATAAGAA  1392
150_PHR_non-APR2     GATCA---TATATACTCGCTCTTGTGCTAGCTGTATGATTCTGTGACTGATACCCCG---TATATAAGAA  1392
```

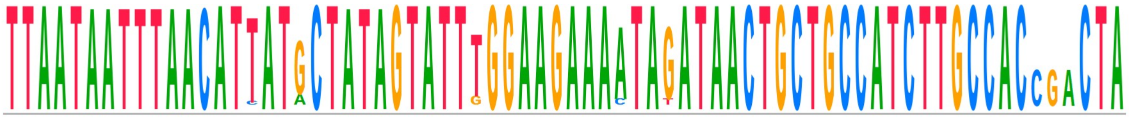

```
Consensus            TTAATAATTTAACATTATGCTATAGTATTTGGAAGAAAATAGATAACTGCTGCCATCTTGCCAC---CTA
                            1690      1700      1710      1720      1730      1740      1750
Lr67(sus)            TTAAT--------ATTATACT---GTATTTGGAAGAAACTATATAACTGCTGCCATCTTGCCACCGACTA 1270
Lr67(res)            TTAAT--------ATTATACT---GTATTTGGAAGAAACTATATAACTGCTGCCATCTTGCCACCGACTA 1270
ScLr67_1 (Lo7)       TTAAT--------ATTATACT---GTATTGGGAAGAAAATAGATAACTGCTGCCATCTTGCCAC---CTA 1618
118_Danko_APR        TTAATAATTTAACATTATGCTATAGTATTTGGAAGAAAATAGATAACTGCTGCCATCTTGCCAC---CTA 1520
119_Danko_APR        TTAATAATTTAACATTATGCTATAGTATTTGGAAGAAAATAGATAACTGCTGCCATCTTGCCAC---CTA 1517
119_Danko_APR2       TTAATAATTTAACATTATGCTATAGTATTTGGAAGAAAATAGATAACTGCTGCCATCTTGCCAC---CTA 1517
120_Danko_APR        TTAATAATTTAACATTATGCTATAGTATTTGGAAGAAAATAGATAACTGCTGCCATCTTGCCAC---CTA 1519
138_Danko_APR        TTAATAATTTAACATTATGCTATAGTATTTGGAAGAAAATAGATAACTGCTGCCATCTTGCCAC---CTA 1515
153_Danko_APR        TTAATAATTTAACATTATGCTATAGTATTTGGAAGAAAATAGATAACTGCTGCCATCTTGCCAC---CTA 1514
157_Danko_APR        TTAATAATTTAACATTATGCTATAGTATTTGGAAGAAAATAGATAACTGCTGCCATCTTGCCAC---CTA 1517
160_Danko_APR        TTAATAATTTAACATTATGCTATAGTATTTGGAAGAAAATAGATAACTGCTGCCATCTTGCCAC---CTA 1507
71_PHR_APR           TTAATAATTTAACATTATGCTATAGTATTTGGAAGAAAATAGATAACTGCTGCCATCTTGCCAC---CTA 1586
149_PHR_APR          TTAATAATTTAACATTATGCTATAGTATTTGGAAGAAAATAGATAACTGCTGCCATCTTGCCAC---CTA 1518
59_Danko_non-APR     TTAATAATTTAACATTATGCTATAGTATTTGGAAGAAAATAGATAACTGCTGCCATCTTGCCAC---CTA 1538
61_Danko_non-APR     TTAAT--------ATTATACT---GTATTGGGAAGAAAATAGATAACTGCTGCCATCTTGCCAC---CTA 1570
123_Danko_non-APR    TTAATAATTTAACATTATGCTATAGTATTTGGAAGAAAATAGATAACTGCTGCCATCTTGCCAC---CTA 1469
129_Danko_non-APR    TTAATAATTTAACATTATGCTATAGTATTTGGAAGAAAATAGATAACTGCTGCCATCTTGCCAC---CTA 1516
37_PHR_non-APR       TTAATAATTTAACATTATGCTATAGTATTTGGAAGAAAATAGATAACTGCTGCCATCTTGCCAC---CTA 1495
52_PHR_non-APR       TTAAT--------ATCATGCT---GTATTGGGAAGAAAATAGATAACTGCTGCCATCTTGCCAC---CTA 1574
88_PHR_non-APR       TTAATAATTTAACATTATGCTATAGTATTTGGAAGAAAATAGATAACTGCTGCCATCTTGCCAC---CTA 1497
101_PHR_non-APR      TTAATAATTTAACATTATGCTATAGTATTTGGAAGAAAATAGATAACTGCTGCCATCTTGCCAC---CTA 1518
105_PHR_non-APR      TTAATAATTTAACATTATGCTATAGTATTTGGAAGAAAATAGATAACTGCTGCCATCTTGCCAC---CTA 1517
150_PHR_non-APR      TTAATAATTTAACATTATGCTATAGTATTTGGAAGAAAATAGATAACTGCTGCCATCTTGCCAC---CTA 1459
150_PHR_non-APR2     TTAATAATTTAACATTATGCTATAGTATTTGGAAGAAAATAGATAACTGCTGCCATCTTGCCAC---CTA 1459
```

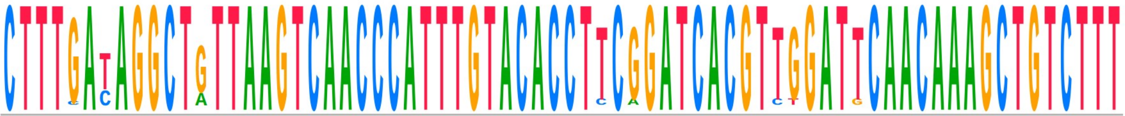

```
Consensus            CTTTGATAGGCTGTTAAGTCAACCCATTTGTACACCTTCGGATCACGTTGGATTCAACAAAGCTGTCTTT
                            1760      1770      1780      1790      1800      1810      1820
Lr67(sus)            CTTTGACAGGCTGTTAAGTCAACCCATTTGTACACCTCCAGATCACGTCTGATGCAACAAAGCTGTCTTT 1340
Lr67(res)            CTTTGACAGGCTGTTAAGTCAACCCATTTGTACACCTCCAGATCACGTCTGATGCAACAAAGCTGTCTTT 1340
ScLr67_1 (Lo7)       CTTTGACAGGCTATTAAGTCAACCCATTTGTACACCTTCGGATCACGTTGGATTCAACAAAGCTGTCTTT 1688
118_Danko_APR        CTTTGATAGGCTGTTAAGTCAACCCATTTGTACACCTTCGGATCACGTTGGATTCAACAAAGCTGTCTTT 1590
119_Danko_APR        CTTTGATAGGCTGTTAAGTCAACCCATTTGTACACCTTCGGATCACGTTGGATTCAACAAAGCTGTCTTT 1587
119_Danko_APR2       CTTTGATAGGCTGTTAAGTCAACCCATTTGTACACCTTCGGATCACGTTGGATTCAACAAAGCTGTCTTT 1587
120_Danko_APR        CTTTGATAGGCTGTTAAGTCAACCCATTTGTACACCTTCGGATCACGTTGGATTCAACAAAGCTGTCTTT 1589
138_Danko_APR        CTTTGATAGGCTGTTAAGTCAACCCATTTGTACACCTTCGGATCACGTTGGATTCAACAAAGCTGTCTTT 1585
153_Danko_APR        CTTTGATAGGCTGTTAAGTCAACCCATTTGTACACCTTCGGATCACGTTGGATTCAACAAAGCTGTCTTT 1584
157_Danko_APR        CTTTGATAGGCTGTTAAGTCAACCCATTTGTACACCTTCGGATCACGTTGGATTCAACAAAGCTGTCTTT 1587
160_Danko_APR        CTTTGATAGGCTGTTAAGTCAACCCATTTGTACACCTTCGGATCACGTTGGATTCAACAAAGCTGTCTTT 1577
71_PHR_APR           CTTTGACAGGCTATTAAGTCAACCCATTTGTACACCTTCGGATCACGTTGGATTCAACAAAGCTGTCTTT 1656
149_PHR_APR          CTTTGATAGGCTGTTAAGTCAACCCATTTGTACACCTTCGGATCACGTTGGATTCAACAAAGCTGTCTTT 1588
59_Danko_non-APR     CTTTGACAGGCTATTAAGTCAACCCATTTGTACACCTTCGGATCACGTTGGATTCAACAAAGCTGTCTTT 1608
61_Danko_non-APR     CTTTGACAGGCTATTAAGTCAACCCATTTGTACACCTTCGGATCACGTTGGATTCAACAAAGCTGTCTTT 1640
123_Danko_non-APR    CTTTGATAGGCTGTTAAGTCAACCCATTTGTACACCTTCGGATCACGTTGGATTCAACAAAGCTGTCTTT 1539
129_Danko_non-APR    CTTTGATAGGCTGTTAAGTCAACCCATTTGTACACCTTCGGATCACGTTGGATTCAACAAAGCTGTCTTT 1586
37_PHR_non-APR       CTTTGATAGGCTGTTAAGTCAACCCATTTGTACACCTTCGGATCACGTTGGATTCAACAAAGCTGTCTTT 1565
52_PHR_non-APR       CTTTCACAGGCTATTAAGTCAACCCATTTGTACACCTTCGGATCACGTTGGATTCAACAAAGCTGTCTTT 1644
88_PHR_non-APR       CTTTGATAGGCTGTTAAGTCAACCCATTTGTACACCTTCGGATCACGTTGGATTCAACAAAGCTGTCTTT 1567
101_PHR_non-APR      CTTTGATAGGCTGTTAAGTCAACCCATTTGTACACCTTCGGATCACGTTGGATTCAACAAAGCTGTCTTT 1588
105_PHR_non-APR      CTTTGATAGGCTGTTAAGTCAACCCATTTGTACACCTTCGGATCACGTTGGATTCAACAAAGCTGTCTTT 1587
150_PHR_non-APR      CTTTGATAGGCTGTTAAGTCAACCCATTTGTACACCTTCGGATCACGTTGGATTCAACAAAGCTGTCTTT 1529
150_PHR_non-APR2     CTTTGATAGGCTGTTAAGTCAACCCATTTGTACACCTTCGGATCACGTTGGATTCAACAAAGCTGTCTTT 1529
```

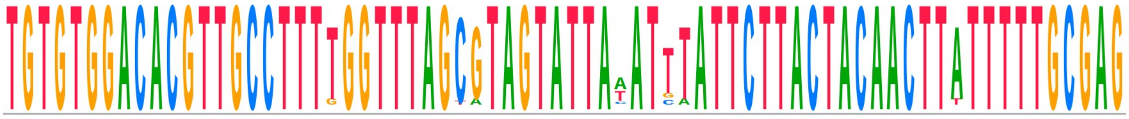

**Consensus**	TGTGTGGACACGTTGCCTTTTGGTTTAGCGTAGTATTANATTTATTCTTACTACAACTTATTTTTGCGAG	
	1830 1840 1850 1860 1870 1880 1890	
Lr67(sus)	TGTGTGGACACGTTGCCTTTGGGTTTAGCATAGTATTACATGAATTCTTACTA-AACTTTTTTTGCGAG	1409
Lr67(res)	TGTGTGGACACGTTGCCTTTGGGTTTAGCATAGTATTACATGAATTCTTACTA-AACTTTTTTTGCGAG	1409
ScLr67_1 (Lo7)	TGTGTGGACACGTTGCCTTTTGGTTTAGCGTAGTATTAAATCTATTCTTACTACAACTTATTTTTGCGAG	1758
118_Danko_APR	TGTGTGGACACGTTGCCTTTTGGTTTAGCGTAGTATTATATTTATTCTTACTACAACTTATTTTTGCGAG	1660
119_Danko_APR	TGTGTGGACACGTTGCCTTTTGGTTTAGCGTAGTATTAAATTTATTCTTACTACAACTTATTTTTGCGAG	1657
119_Danko_APR2	TGTGTGGACACGTTGCCTTTTGGTTTAGCGTAGTATTAAATTTATTCTTACTACAACTTATTTTTGCGAG	1657
120_Danko_APR	TGTGTGGACACGTTGCCTTTTGGTTTAGCGTAGTATTAAATTTATTCTTACTACAACTTATTTTTGCGAG	1659
138_Danko_APR	TGTGTGGACACGTTGCCTTTTGGTTTAGCGTAGTATTAAATTTATTCTTACTACAACTTATTTTTGCGAG	1655
153_Danko_APR	TGTGTGGACACGTTGCCTTTTGGTTTAGCGTAGTATTATATTTATTCTTACTACAACTTATTTTTGCGAG	1654
157_Danko_APR	TGTGTGGACACGTTGCCTTTTGGTTTAGCGTAGTATTAAATTTATTCTTACTACAACTTATTTTTGCGAG	1657
160_Danko_APR	TGTGTGGACACGTTGCCTTTTGGTTTAGCGTAGTATTAAATTTATTCTTACTACAACTTATTTTTGCGAG	1647
71_PHR_APR	TGTGTGGACACGTTGCCTTTTGGTTTAGCGTAGTATTAAATTTATTCTTACTACAACTTATTTTTGCGAG	1726
149_PHR_APR	TGTGTGGACACGTTGCCTTTTGGTTTAGCGTAGTATTATATTTATTCTTACTACAACTTATTTTTGCGAG	1658
59_Danko_non-APR	TGTGTGGACACGTTGCCTTTTGGTTTAGTGTAGTATTAAATTTATTCTTACTACAACTTATTTTTGCGAG	1678
61_Danko_non-APR	TGTGTGGACACGTTGCCTTTTGGTTTAGCGTAGTATTAAATCTATTCTTACTACAACTTATTTTTGCGAG	1710
123_Danko_non-APR	TGTGTGGACACGTTGCCTTTTGGTTTAGCGTAGTATTATATTTATTCTTACTACAACTTATTTTTGCGAG	1609
129_Danko_non-APR	TGTGTGGACACGTTGCCTTTTGGTTTAGCGTAGTATTATATTTATTCTTACTACAACTTATTTTTGCGAG	1656
37_PHR_non-APR	TGTGTGGACACGTTGCCTTTTGGTTTAGCGTAGTATTAAATTTATTCTTACTACAACTTATTTTTGCGAG	1635
52_PHR_non-APR	TGTGTGGACACGTTGCCTTTTGGTTTAGCGTAGTATTAAATTTATTCTTACTACAACTTATTTTTGCGAG	1714
88_PHR_non-APR	TGTGTGGACACGTTGCCTTTTGGTTTAGCGTAGTATTATATTTATTCTTACTACAACTTATTTTTGCGAG	1637
101_PHR_non-APR	TGTGTGGACACGTTGCCTTTTGGTTTAGCGTAGTATTATATTTATTCTTACTACAACTTATTTTTGCGAG	1658
105_PHR_non-APR	TGTGTGGACACGTTGCCTTTTGGTTTAGCGTAGTATTATATTTATTCTTACTACAACTTATTTTTGCGAG	1657
150_PHR_non-APR	TGTGTGGACACGTTGCCTTTTGGTTTAGCGTAGTATTATATTTATTCTTACTACAACTTATTTTTGCGAG	1599
150_PHR_non-APR2	TGTGTGGACACGTTGCCTTTTGGTTTAGCGTAGTATTATATTTATTCTTACTACAACTTATTTTTGCGAG	1599

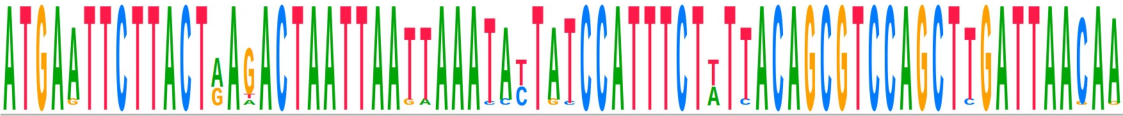

**Consensus**	ATGAATTCTTACTAAGACTAATTAATTAAATATTATCCATTTCTTTTACAGCGTCCAGCTTGATTAACAA	
	1900 1910 1920 1930 1940 1950 1960	
Lr67(sus)	ATGAATTCTTACTAAAACTAATTAAGAAAATCTTGTCCATTTCTTTCACAGCGTCCAGCTCGATTAACAA	1479
Lr67(res)	ATGAATTCTTACTAAAACTAATTAAGAAAATCTTGTCCATTTCTTTCACAGCGTCCAGCTCGATTAACAA	1479
ScLr67_1 (Lo7)	ATGAGTTCTTACTAATACTAATTAATTAAATATTATCCATTTCTTTTACAGCGTCCAGCTTGATTAACAA	1828
118_Danko_APR	ATGAATTCTTACTAAGACTAATTAATTAAATACTATCCATTTCTATTACAGCGTCCAGCTTGATTAACAA	1730
119_Danko_APR	ATGAATTCTTACTGAGACTAATTAATTAAATATTATCCATTTCTTTTACAGCGTCCAGCTTGATTAACAA	1727
119_Danko_APR2	ATGAATTCTTACTGAGACTAATTAATTAAATATTATCCATTTCTTTTACAGCGTCCAGCTTGATTAACAA	1727
120_Danko_APR	ATGAATTCTTACTGAGACTAATTAATTAAATATTATCCATTTCTTTTACAGCGTCCAGCTTGATTAACAA	1729
138_Danko_APR	ATGAATTCTTACTGAGACTAATTAATTAAATATTATCCATTTCTTTTACAGCGTCCAGCTTGATTAACAA	1725
153_Danko_APR	ATGAATTCTTACTAAGACTAATTAATTAAATACTATCCATTTCTATTACAGCGTCCAGCTTGATTAACAA	1724
157_Danko_APR	ATGAATTCTTACTGAGACTAATTAATTAAATATTATCCATTTCTTTTACAGCGTCCAGCTTGATTAACAA	1727
160_Danko_APR	ATGAATTCTTACTGAGACTAATTAATTAAATATTATCCATTTCTTTTACAGCGTCCAGCTTGATTAACAA	1717
71_PHR_APR	ATGAATTCTTACTGAGACTAATTAATTAAATATTATCCATTTCTTTTACAGCGTCCAGCTTGATTAACAA	1796
149_PHR_APR	ATGAATTCTTACTAAGACTAATTAATTAAATACTATCCATTTCTATTACAGCGTCCAGCTTGATTAACAA	1728
59_Danko_non-APR	ATGAATTCTTACTGAGACTAATTAATTAAATATTATCCATTTCTTTTACAGCGTCCAGCTTGATTAACAA	1748
61_Danko_non-APR	ATGAGTTCTTACTAATACTAATTAATTAAATATTATCCATTTCTTTTACAGCGTCCAGCTTGATTAACAA	1780
123_Danko_non-APR	ATGAATTCTTACTAAGACTAATTAATTAAATACTATCCATTTCTATTACAGCGTCCAGCTTGATTAACAA	1679
129_Danko_non-APR	ATGAATTCTTACTAAGACTAATTAATTAAATACTATCCATTTCTATTACAGCGTCCAGCTTGATTAACAA	1726
37_PHR_non-APR	ATGAATTCTTACTGAGACTAATTAATTAAATATTATCCATTTCTTTTACAGCGTCCAGCTTGATTAACAA	1705
52_PHR_non-APR	ATGAATTCTTACTAAGACTAATTAATTAAACATTACCCATTTCTTTTACAGCGTCCAGCTTGATTAAGAG	1784
88_PHR_non-APR	ATGAATTCTTACTAAGACTAATTAATTAAATACTATCCATTTCTATTACAGCGTCCAGCTTGATTAACAA	1707
101_PHR_non-APR	ATGAATTCTTACTAAGACTAATTAATTAAATACTATCCATTTCTATTACAGCGTCCAGCTTGATTAACAA	1728
105_PHR_non-APR	ATGAATTCTTACTAAGACTAATTAATTAAATACTATCCATTTCTATTACAGCGTCCAGCTTGATTAACAA	1727
150_PHR_non-APR	ATGAATTCTTACTAAGACTAATTAATTAAATACTATCCATTTCTATTACAGCGTCCAGCTTGATTAACAA	1669
150_PHR_non-APR2	ATGAATTCTTACTAAGACTAATTAATTAAATACTATCCATTTCTATTACAGCGTCCAGCTTGATTAACAA	1669

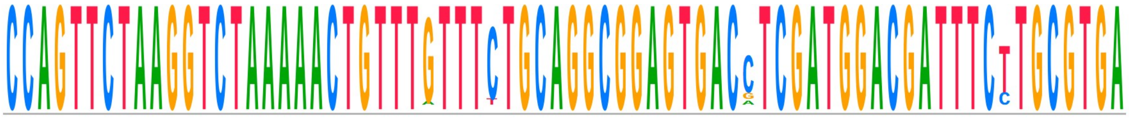

	Sequence (1970–2030)	
**Consensus**	CCAGTTCTAAGGTCTAAAAACTGTTTGTTTCTGCAGGCGGAGTGACCTCGATGGACGATTTCTTGCGTGA	
Lr67(sus)	CCAGTTCTAAGGTCTAAAAACTGTTTGTTTTTGCAGGCGGAGTGACATCGATGGACGATTTCCTGCGTGA	1549
Lr67(res)	CCAGTTCTAAGGTCTAAAAACTGTTTGTTTTTGCAGGCGGAGTGACATCGATGGACGATTTCCTGCGTGA	1549
ScLr67_1 (Lo7)	CCAGTTCTAAGGTCTAAAAACTGTTTGTTTCTGCAGGCGGAGTGACGTCGATGGACGATTTCCTGCGTGA	1898
118_Danko_APR	CCAGTTCTAAGGTCTAAAAACTGTTTGTTTCTGCAGGCGGAGTGACCTCGATGGACGATTTCTTGCGTGA	1800
119_Danko_APR	CCAGTTCTAAGGTCTAAAAACTGTTTGTTTCTGCAGGCGGAGTGACCTCGATGGACGATTTCTTGCGTGA	1797
119_Danko_APR2	CCAGTTCTAAGGTCTAAAAACTGTTTGTTTCTGCAGGCGGAGTGACCTCGATGGACGATTTCTTGCGTGA	1797
120_Danko_APR	CCAGTTCTAAGGTCTAAAAACTGTTTGTTTCTGCAGGCGGAGTGACCTCGATGGACGATTTCTTGCGTGA	1799
138_Danko_APR	CCAGTTCTAAGGTCTAAAAACTGTTTGTTTCTGCAGGCGGAGTGACCTCGATGGACGATTTCTTGCGTGA	1795
153_Danko_APR	CCAGTTCTAAGGTCTAAAAACTGTTTGTTTCTGCAGGCGGAGTGACCTCGATGGACGATTTCTTGCGTGA	1794
157_Danko_APR	CCAGTTCTAAGGTCTAAAAACTGTTTGTTTCTGCAGGCGGAGTGACCTCGATGGACGATTTCTTGCGTGA	1797
160_Danko_APR	CCAGTTCTAAGGTCTAAAAACTGTTTGTTTCTGCAGGCGGAGTGACCTCGATGGACGATTTCTTGCGTGA	1787
71_PHR_APR	CCAGTTCTAAGGTCTAAAAACTGTTTGTTTCTGCAGGCGGAGTGACCTCGATGGACGATTTCTTGCGTGA	1866
149_PHR_APR	CCAGTTCTAAGGTCTAAAAACTGTTTGTTTCTGCAGGCGGAGTGACCTCGATGGACGATTTCTTGCGTGA	1798
59_Danko_non-APR	CCAGTTCTAAGGTCTAAAAACTGTTTGTTTCTGCAGGCGGAGTGACCTCGATGGACGATTTCTTGCGTGA	1818
61_Danko_non-APR	CCAGTTCTAAGGTCTAAAAACTGTTTGTTTCTGCAGGCGGAGTGACGTCGATGGACGATTTCCTGCGTGA	1850
123_Danko_non-APR	CCAGTTCTAAGGTCTAAAAACTGTTTGTTTCTGCAGGCGGAGTGACCTCGATGGACGATTTCTTGCGTGA	1749
129_Danko_non-APR	CCAGTTCTAAGGTCTAAAAACTGTTTGTTTCTGCAGGCGGAGTGACCTCGATGGACGATTTCTTGCGTGA	1796
37_PHR_non-APR	CCAGTTCTAAGGTCTAAAAACTGTTTGTTTCTGCAGGCGGAGTGACCTCGATGGACGATTTCTTGCGTGA	1775
52_PHR_non-APR	CCAGTTCTAAGGTCTAAAAACTGTTTATTTCTGCAGGCGGAGTGACGTCGATGGACGATTTCCTGCGTGA	1854
88_PHR_non-APR	CCAGTTCTAAGGTCTAAAAACTGTTTGTTTCTGCAGGCGGAGTGACCTCGATGGACGATTTCTTGCGTGA	1777
101_PHR_non-APR	CCAGTTCTAAGGTCTAAAAACTGTTTGTTTCTGCAGGCGGAGTGACCTCGATGGACGATTTCTTGCGTGA	1798
105_PHR_non-APR	CCAGTTCTAAGGTCTAAAAACTGTTTGTTTCTGCAGGCGGAGTGACCTCGATGGACGATTTCTTGCGTGA	1797
150_PHR_non-APR	CCAGTTCTAAGGTCTAAAAACTGTTTGTTTCTGCAGGCGGAGTGACCTCGATGGACGATTTCTTGCGTGA	1739
150_PHR_non-APR2	CCAGTTCTAAGGTCTAAAAACTGTTTGTTTCTGCAGGCGGAGTGACCTCGATGGACGATTTCTTGCGTGA	1739

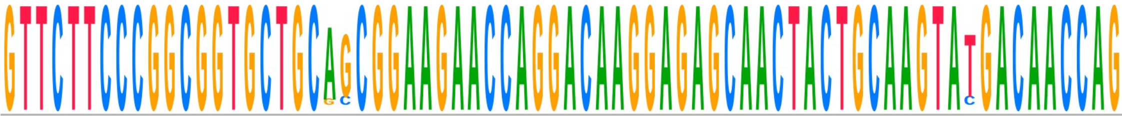

	Sequence (2040–2100)	
**Consensus**	GTTCTTCCCGGCGGTGCTGCAGCGGAAGAACCAGGACAAGGAGAGCAACTACTGCAAGTATGACAACCAG	
Lr67(sus)	GTTCTTCCCGGCGGTGCTGCGCCGGAAGAACCAGGACAAGGAGAGCAACTACTGCAAGTACGACAACCAG	1619
Lr67(res)	GTTCTTCCCGGCGGTGCTGCGCCGGAAGAACCAGGACAAGGAGAGCAACTACTGCAAGTACGACAACCAG	1619
ScLr67_1 (Lo7)	GTTCTTCCCGGCGGTGCTGCAGCGGAAGAACCAGGACAAGGAGAGCAACTACTGCAAGTATGACAACCAG	1968
118_Danko_APR	GTTCTTCCCGGCGGTGCTGCAGCGGAAGAACCAGGACAAGGAGAGCAACTACTGCAAGTATGACAACCAG	1870
119_Danko_APR	GTTCTTCCCGGCGGTGCTGCAGCGGAAGAACCAGGACAAGGAGAGCAACTACTGCAAGTATGACAACCAG	1867
119_Danko_APR2	GTTCTTCCCGGCGGTGCTGCAGCGGAAGAACCAGGACAAGGAGAGCAACTACTGCAAGTATGACAACCAG	1867
120_Danko_APR	GTTCTTCCCGGCGGTGCTGCAGCGGAAGAACCAGGACAAGGAGAGCAACTACTGCAAGTATGACAACCAG	1869
138_Danko_APR	GTTCTTCCCGGCGGTGCTGCAGCGGAAGAACCAGGACAAGGAGAGCAACTACTGCAAGTATGACAACCAG	1865
153_Danko_APR	GTTCTTCCCGGCGGTGCTGCAGCGGAAGAACCAGGACAAGGAGAGCAACTACTGCAAGTATGACAACCAG	1864
157_Danko_APR	GTTCTTCCCGGCGGTGCTGCAGCGGAAGAACCAGGACAAGGAGAGCAACTACTGCAAGTATGACAACCAG	1867
160_Danko_APR	GTTCTTCCCGGCGGTGCTGCAGCGGAAGAACCAGGACAAGGAGAGCAACTACTGCAAGTATGACAACCAG	1857
71_PHR_APR	GTTCTTCCCGGCGGTGCTGCAGCGGAAGAACCAGGACAAGGAGAGCAACTACTGCAAGTATGACAACCAG	1936
149_PHR_APR	GTTCTTCCCGGCGGTGCTGCAGCGGAAGAACCAGGACAAGGAGAGCAACTACTGCAAGTATGACAACCAG	1868
59_Danko_non-APR	GTTCTTCCCGGCGGTGCTGCAGCGGAAGAACCAGGACAAGGAGAGCAACTACTGCAAGTATGACAACCAG	1888
61_Danko_non-APR	GTTCTTCCCGGCGGTGCTGCAGCGGAAGAACCAGGACAAGGAGAGCAACTACTGCAAGTATGACAACCAG	1920
123_Danko_non-APR	GTTCTTCCCGGCGGTGCTGCAGCGGAAGAACCAGGACAAGGAGAGCAACTACTGCAAGTATGACAACCAG	1819
129_Danko_non-APR	GTTCTTCCCGGCGGTGCTGCAGCGGAAGAACCAGGACAAGGAGAGCAACTACTGCAAGTATGACAACCAG	1866
37_PHR_non-APR	GTTCTTCCCGGCGGTGCTGCAGCGGAAGAACCAGGACAAGGAGAGCAACTACTGCAAGTATGACAACCAG	1845
52_PHR_non-APR	GTTCTTCCCGGCGGTGCTGCACCGGAAGAACCAGGACAAGGAGAGCAACTACTGCAAGTACGACAACCAG	1924
88_PHR_non-APR	GTTCTTCCCGGCGGTGCTGCAGCGGAAGAACCAGGACAAGGAGAGCAACTACTGCAAGTATGACAACCAG	1847
101_PHR_non-APR	GTTCTTCCCGGCGGTGCTGCAGCGGAAGAACCAGGACAAGGAGAGCAACTACTGCAAGTATGACAACCAG	1868
105_PHR_non-APR	GTTCTTCCCGGCGGTGCTGCAGCGGAAGAACCAGGACAAGGAGAGCAACTACTGCAAGTATGACAACCAG	1867
150_PHR_non-APR	GTTCTTCCCGGCGGTGCTGCAGCGGAAGAACCAGGACAAGGAGAGCAACTACTGCAAGTATGACAACCAG	1809
150_PHR_non-APR2	GTTCTTCCCGGCGGTGCTGCAGCGGAAGAACCAGGACAAGGAGAGCAACTACTGCAAGTATGACAACCAG	1809

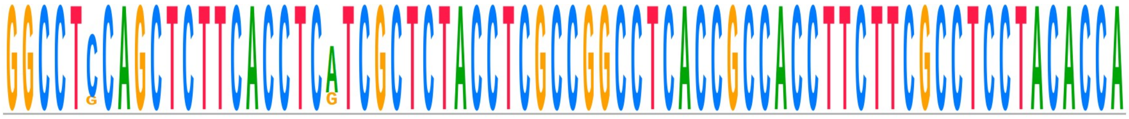

**Consensus**	GGCCTCCAGCTCTTCACCTCATCGCTCTACCTCGCCGGCCTCACCGCCACCTTCTTCGCCTCCTACACCA	
	2110 2120 2130 2140 2150 2160 2170	
Lr67(sus)	GGCCTGCAGCTCTTCACCTCGTCGCTCTACCTCGCCGGCCTCACCGCCACCTTCTTCGCCTCCTACACCA	1689
Lr67(res)	GGCCTGCAGCTCTTCACCTCGTCGCTCTACCTCGCCGGCCTCACCGCCACCTTCTTCGCCTCCTACACCA	1689
ScLr67_1 (Lo7)	GGCCTCCAGCTCTTCACCTCGTCGCTCTACCTCGCCGGCCTCACCGCCACCTTCTTCGCCTCCTACACCA	2038
118_Danko_APR	GGCCTCCAGCTCTTCACCTCATCGCTCTACCTCGCCGGCCTCACCGCCACCTTCTTCGCCTCCTACACCA	1940
119_Danko_APR	GGCCTCCAGCTCTTCACCTCATCGCTCTACCTCGCCGGCCTCACCGCCACCTTCTTCGCCTCCTACACCA	1937
119_Danko_APR2	GGCCTCCAGCTCTTCACCTCATCGCTCTACCTCGCCGGCCTCACCGCCACCTTCTTCGCCTCCTACACCA	1937
120_Danko_APR	GGCCTCCAGCTCTTCACCTCATCGCTCTACCTCGCCGGCCTCACCGCCACCTTCTTCGCCTCCTACACCA	1939
138_Danko_APR	GGCCTCCAGCTCTTCACCTCATCGCTCTACCTCGCCGGCCTCACCGCCACCTTCTTCGCCTCCTACACCA	1935
153_Danko_APR	GGCCTCCAGCTCTTCACCTCATCGCTCTACCTCGCCGGCCTCACCGCCACCTTCTTCGCCTCCTACACCA	1934
157_Danko_APR	GGCCTCCAGCTCTTCACCTCATCGCTCTACCTCGCCGGCCTCACCGCCACCTTCTTCGCCTCCTACACCA	1937
160_Danko_APR	GGCCTCCAGCTCTTCACCTCATCGCTCTACCTCGCCGGCCTCACCGCCACCTTCTTCGCCTCCTACACCA	1927
71_PHR_APR	GGCCTCCAGCTCTTCACCTCATCGCTCTACCTCGCCGGCCTCACCGCCACCTTCTTCGCCTCCTACACCA	2006
149_PHR_APR	GGCCTCCAGCTCTTCACCTCATCGCTCTACCTCGCCGGCCTCACCGCCACCTTCTTCGCCTCCTACACCA	1938
59_Danko_non-APR	GGCCTCCAGCTCTTCACCTCATCGCTCTACCTCGCCGGCCTCACCGCCACCTTCTTCGCCTCCTACACCA	1958
61_Danko_non-APR	GGCCTCCAGCTCTTCACCTCGTCGCTCTACCTCGCCGGCCTCACCGCCACCTTCTTCGCCTCCTACACCA	1990
123_Danko_non-APR	GGCCTCCAGCTCTTCACCTCATCGCTCTACCTCGCCGGCCTCACCGCCACCTTCTTCGCCTCCTACACCA	1889
129_Danko_non-APR	GGCCTCCAGCTCTTCACCTCATCGCTCTACCTCGCCGGCCTCACCGCCACCTTCTTCGCCTCCTACACCA	1936
37_PHR_non-APR	GGCCTCCAGCTCTTCACCTCATCGCTCTACCTCGCCGGCCTCACCGCCACCTTCTTCGCCTCCTACACCA	1915
52_PHR_non-APR	GGCCTGCAGCTCTTCACCTCGTCGCTCTACCTCGCCGGCCTCACCGCCACCTTCTTCGCCTCCTACACCA	1994
88_PHR_non-APR	GGCCTCCAGCTCTTCACCTCATCGCTCTACCTCGCCGGCCTCACCGCCACCTTCTTCGCCTCCTACACCA	1917
101_PHR_non-APR	GGCCTCCAGCTCTTCACCTCATCGCTCTACCTCGCCGGCCTCACCGCCACCTTCTTCGCCTCCTACACCA	1938
105_PHR_non-APR	GGCCTCCAGCTCTTCACCTCATCGCTCTACCTCGCCGGCCTCACCGCCACCTTCTTCGCCTCCTACACCA	1937
150_PHR_non-APR	GGCCTCCAGCTCTTCACCTCATCGCTCTACCTCGCCGGCCTCACCGCCACCTTCTTCGCCTCCTACACCA	1879
150_PHR_non-APR2	GGCCTCCAGCTCTTCACCTCATCGCTCTACCTCGCCGGCCTCACCGCCACCTTCTTCGCCTCCTACACCA	1879

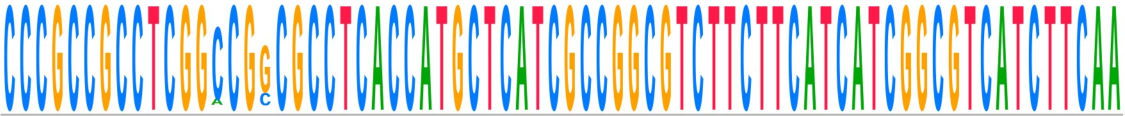

**Consensus**	CCCGCCGCCTCGGCCGGCGCCTCACCATGCTCATCGCCGGCGTCTTCTTCATCATCGGCGTCATCTTCAA	
	2180 2190 2200 2210 2220 2230 2240	
Lr67(sus)	CCCGCCGCCTCGGACGCCGCCTCACCATGCTCATCGCCGGCGTCTTCTTCATCATCGGCGTCATCTTCAA	1759
Lr67(res)	CCCGCCGCCTCGGACGCCGCCTCACCATGCTCATCGCCGGCGTCTTCTTCATCATCGGCGTCATCTTCAA	1759
ScLr67_1 (Lo7)	CCCGCCGCCTCGGCCGCCGCCTCACCATGCTCATCGCCGGCGTCTTCTTCATCATCGGCGTCATCTTCAA	2108
118_Danko_APR	CCCGCCGCCTCGGCCGGCGCCTCACCATGCTCATCGCCGGCGTCTTCTTCATCATCGGCGTCATCTTCAA	2010
119_Danko_APR	CCCGCCGCCTCGGCCGGCGCCTCACCATGCTCATCGCCGGCGTCTTCTTCATCATCGGCGTCATCTTCAA	2007
119_Danko_APR2	CCCGCCGCCTCGGCCGGCGCCTCACCATGCTCATCGCCGGCGTCTTCTTCATCATCGGCGTCATCTTCAA	2007
120_Danko_APR	CCCGCCGCCTCGGCCGGCGCCTCACCATGCTCATCGCCGGCGTCTTCTTCATCATCGGCGTCATCTTCAA	2009
138_Danko_APR	CCCGCCGCCTCGGCCGGCGCCTCACCATGCTCATCGCCGGCGTCTTCTTCATCATCGGCGTCATCTTCAA	2005
153_Danko_APR	CCCGCCGCCTCGGCCGGCGCCTCACCATGCTCATCGCCGGCGTCTTCTTCATCATCGGCGTCATCTTCAA	2004
157_Danko_APR	CCCGCCGCCTCGGCCGGCGCCTCACCATGCTCATCGCCGGCGTCTTCTTCATCATCGGCGTCATCTTCAA	2007
160_Danko_APR	CCCGCCGCCTCGGCCGGCGCCTCACCATGCTCATCGCCGGCGTCTTCTTCATCATCGGCGTCATCTTCAA	1997
71_PHR_APR	CCCGCCGCCTCGGCCGGCGCCTCACCATGCTCATCGCCGGCGTCTTCTTCATCATCGGCGTCATCTTCAA	2076
149_PHR_APR	CCCGCCGCCTCGGCCGGCGCCTCACCATGCTCATCGCCGGCGTCTTCTTCATCATCGGCGTCATCTTCAA	2008
59_Danko_non-APR	CCCGCCGCCTCGGCCGGCGCCTCACCATGCTCATCGCCGGCGTCTTCTTCATCATCGGCGTCATCTTCAA	2028
61_Danko_non-APR	CCCGCCGCCTCGGCCGCCGCCTCACCATGCTCATCGCCGGCGTCTTCTTCATCATCGGCGTCATCTTCAA	2060
123_Danko_non-APR	CCCGCCGCCTCGGCCGGCGCCTCACCATGCTCATCGCCGGCGTCTTCTTCATCATCGGCGTCATCTTCAA	1959
129_Danko_non-APR	CCCGCCGCCTCGGCCGGCGCCTCACCATGCTCATCGCCGGCGTCTTCTTCATCATCGGCGTCATCTTCAA	2006
37_PHR_non-APR	CCCGCCGCCTCGGCCGGCGCCTCACCATGCTCATCGCCGGCGTCTTCTTCATCATCGGCGTCATCTTCAA	1985
52_PHR_non-APR	CCCGCCGCCTCGGCCGCCGCCTCACCATGCTCATCGCCGGCGTCTTCTTCATCATCGGCGTCATCTTCAA	2064
88_PHR_non-APR	CCCGCCGCCTCGGCCGGCGCCTCACCATGCTCATCGCCGGCGTCTTCTTCATCATCGGCGTCATCTTCAA	1987
101_PHR_non-APR	CCCGCCGCCTCGGCCGGCGCCTCACCATGCTCATCGCCGGCGTCTTCTTCATCATCGGCGTCATCTTCAA	2008
105_PHR_non-APR	CCCGCCGCCTCGGCCGGCGCCTCACCATGCTCATCGCCGGCGTCTTCTTCATCATCGGCGTCATCTTCAA	2007
150_PHR_non-APR	CCCGCCGCCTCGGCCGGCGCCTCACCATGCTCATCGCCGGCGTCTTCTTCATCATCGGCGTCATCTTCAA	1949
150_PHR_non-APR2	CCCGCCGCCTCGGCCGGCGCCTCACCATGCTCATCGCCGGCGTCTTCTTCATCATCGGCGTCATCTTCAA	1949

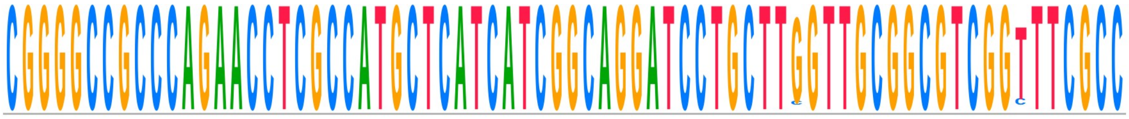

**Consensus**	CGGGGCCGCCCAGAACCTCGCCATGCTCATCATCGGCAGGATCCTGCTTGGTTGCGGCGTCGGTTTCGCC	
	2250 2260 2270 2280 2290 2300 2310	
Lr67(sus)	CGGGGCCGCCCAGAACCTCGCCATGCTCATCATCGGCAGGATCCTGCTTGGTTGCGGCGTCGGCTTCGCC	1829
Lr67(res)	CGGGGCCGCCCAGAACCTCGCCATGCTCATCATCGGCAGGATCCTGCTTCGTTGCGGCGTCGGCTTCGCC	1829
ScLr67_1 (Lo7)	CGGGGCCGCCCAGAACCTCGCCATGCTCATCATCGGCAGGATCCTGCTTGGTTGCGGCGTCGGTTTCGCC	2178
118_Danko_APR	CGGGGCCGCCCAGAACCTCGCCATGCTCATCATCGGCAGGATCCTGCTTGGTTGCGGCGTCGGTTTCGCC	2080
119_Danko_APR	CGGGGCCGCCCAGAACCTCGCCATGCTCATCATCGGCAGGATCCTGCTTGGTTGCGGCGTCGGTTTCGCC	2077
119_Danko_APR2	CGGGGCCGCCCAGAACCTCGCCATGCTCATCATCGGCAGGATCCTGCTTGGTTGCGGCGTCGGTTTCGCC	2077
120_Danko_APR	CGGGGCCGCCCAGAACCTCGCCATGCTCATCATCGGCAGGATCCTGCTTGGTTGCGGCGTCGGTTTCGCC	2079
138_Danko_APR	CGGGGCCGCCCAGAACCTCGCCATGCTCATCATCGGCAGGATCCTGCTTGGTTGCGGCGTCGGTTTCGCC	2075
153_Danko_APR	CGGGGCCGCCCAGAACCTCGCCATGCTCATCATCGGCAGGATCCTGCTTGGTTGCGGCGTCGGTTTCGCC	2074
157_Danko_APR	CGGGGCCGCCCAGAACCTCGCCATGCTCATCATCGGCAGGATCCTGCTTGGTTGCGGCGTCGGTTTCGCC	2077
160_Danko_APR	CGGGGCCGCCCAGAACCTCGCCATGCTCATCATCGGCAGGATCCTGCTTGGTTGCGGCGTCGGTTTCGCC	2067
71_PHR_APR	CGGGGCCGCCCAGAACCTCGCCATGCTCATCATCGGCAGGATCCTGCTTGGTTGCGGCGTCGGTTTCGCC	2146
149_PHR_APR	CGGGGCCGCCCAGAACCTCGCCATGCTCATCATCGGCAGGATCCTGCTTGGTTGCGGCGTCGGTTTCGCC	2078
59_Danko_non-APR	CGGGGCCGCCCAGAACCTCGCCATGCTCATCATCGGCAGGATCCTGCTTGGTTGCGGCGTCGGTTTCGCC	2098
61_Danko_non-APR	CGGGGCCGCCCAGAACCTCGCCATGCTCATCATCGGCAGGATCCTGCTTGGTTGCGGCGTCGGTTTCGCC	2130
123_Danko_non-APR	CGGGGCCGCCCAGAACCTCGCCATGCTCATCATCGGCAGGATCCTGCTTGGTTGCGGCGTCGGTTTCGCC	2029
129_Danko_non-APR	CGGGGCCGCCCAGAACCTCGCCATGCTCATCATCGGCAGGATCCTGCTTGGTTGCGGCGTCGGTTTCGCC	2076
37_PHR_non-APR	CGGGGCCGCCCAGAACCTCGCCATGCTCATCATCGGCAGGATCCTGCTTGGTTGCGGCGTCGGTTTCGCC	2055
52_PHR_non-APR	CGGGGCCGCCCAGAACCTCGCCATGCTCATCATCGGCAGGATCCTGCTTGGTTGCGGCGTCGGTTTCGCC	2134
88_PHR_non-APR	CGGGGCCGCCCAGAACCTCGCCATGCTCATCATCGGCAGGATCCTGCTTGGTTGCGGCGTCGGTTTCGCC	2057
101_PHR_non-APR	CGGGGCCGCCCAGAACCTCGCCATGCTCATCATCGGCAGGATCCTGCTTGGTTGCGGCGTCGGTTTCGCC	2078
105_PHR_non-APR	CGGGGCCGCCCAGAACCTCGCCATGCTCATCATCGGCAGGATCCTGCTTGGTTGCGGCGTCGGTTTCGCC	2077
150_PHR_non-APR	CGGGGCCGCCCAGAACCTCGCCATGCTCATCATCGGCAGGATCCTGCTTGGTTGCGGCGTCGGTTTCGCC	2019
150_PHR_non-APR2	CGGGGCCGCCCAGAACCTCGCCATGCTCATCATCGGCAGGATCCTGCTTGGTTGCGGCGTCGGTTTCGCC	2019

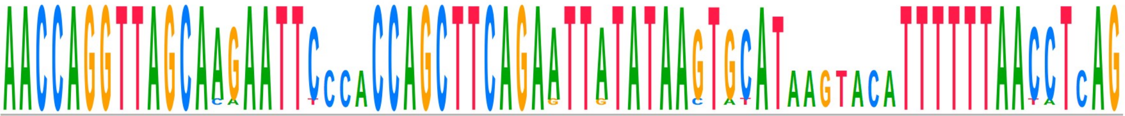

**Consensus**	AACCAGGTTAGCAAGAATTC---CCAGCTTCAGAATTATATAAGTGCAN-------TTTTTTAACCT-AG	
	2320 2330 2340 2350 2360 2370 2380	
Lr67(sus)	AACCAGGTTAGCACAAATTTCCACCAGCTTCAGAATTGTATA--------------TTTTTTAATATCAG	1885
Lr67(res)	AACCAGGTTAGCACAAATTTCCACCAGCTTCAGAATTGTATA--------------TTTTTTAATATCAG	1885
ScLr67_1 (Lo7)	AACCAGGTTAGCAAGAATTC---CCAGCTTCAGAGTTATATAACTATATAAGTACATTTTTTAACCT-AG	2244
118_Danko_APR	AACCAGGTTAGCAAGAATTC---CCAGCTTCAGAATTATATAAGTGCA--------TTTTTTAACCT-AG	2138
119_Danko_APR	AACCAGGTTAGCAAGAATTC---CCAGCTTCAGAATTATATAAGTGCAT-------TTTTTTAACCT-AG	2136
119_Danko_APR2	AACCAGGTTAGCAAGAATTC---CCAGCTTCAGAATTATATAAGTGCAT-------TTTTTTAACCT-AG	2136
120_Danko_APR	AACCAGGTTAGCAAGAATTC---CCAGCTTCAGAATTATATAAGTGCAT-------TTTTTTAACCT-AG	2138
138_Danko_APR	AACCAGGTTAGCAAGAATTC---CCAGCTTCAGAATTATATAAGTGCAT-------TTTTTTAACCT-AG	2134
153_Danko_APR	AACCAGGTTAGCAAGAATTC---CCAGCTTCAGAATTATATAAGTGCA--------TTTTTTAACCT-AG	2132
157_Danko_APR	AACCAGGTTAGCAAGAATTC---CCAGCTTCAGAATTATATAAGTGCAT-------TTTTTTAACCT-AG	2136
160_Danko_APR	AACCAGGTTAGCAAGAATTC---CCAGCTTCAGAATTATATAAGTGCAT-------TTTTTTAACCT-AG	2126
71_PHR_APR	AACCAGGTTAGCAAGAATTC---CCAGCTTCAGAATTATATAAGTGCAT-------TTTTTTAACCT-AG	2205
149_PHR_APR	AACCAGGTTAGCAAGAATTC---CCAGCTTCAGAATTATATAAGTGCA--------TTTTTTAACCT-AG	2136
59_Danko_non-APR	AACCAGGTTAGCAAGAATTC---CCAGCTTCAGAATTATATAAGTGCAT-------TTTTTTAACCT-AG	2157
61_Danko_non-APR	AACCAGGTTAGCAAGAATTC---CCAGCTTCAGAGTTATATAACTATATAAGTACATTTTTTAACCT-AG	2196
123_Danko_non-APR	AACCAGGTTAGCAAGAATTC---CCAGCTTCAGAATTATATAAGTGCA--------TTTTTTAACCT-AG	2087
129_Danko_non-APR	AACCAGGTTAGCAAGAATTC---CCAGCTTCAGAATTATATAAGTGCA--------TTTTTTAACCT-AG	2134
37_PHR_non-APR	AACCAGGTTAGCAAGAATTC---CCAGCTTCAGAATTATATAAGTGCAT-------TTTTTTAACCT-AG	2114
52_PHR_non-APR	AACCAGGTTAGCAAGAATTC---CCAGCTTCAGAATTATATAAGTGCAT-------TTTTTTAACCT-AG	2193
88_PHR_non-APR	AACCAGGTTAGCAAGAATTC---CCAGCTTCAGAATTATATAAGTGCA--------TTTTTTAACCT-AG	2115
101_PHR_non-APR	AACCAGGTTAGCAAGAATTC---CCAGCTTCAGAATTATATAAGTGCA--------TTTTTTAACCT-AG	2136
105_PHR_non-APR	AACCAGGTTAGCAAGAATTC---CCAGCTTCAGAATTATATAAGTGCA--------TTTTTTAACCT-AG	2135
150_PHR_non-APR	AACCAGGTTAGCAAGAATTC---CCAGCTTCAGAATTATATAAGTGCA--------TTTTTTAACCT-AG	2077
150_PHR_non-APR2	AACCAGGTTAGCAAGAATTC---CCAGCTTCAGAATTATATAAGTGCA--------TTTTTTAACCT-AG	2077

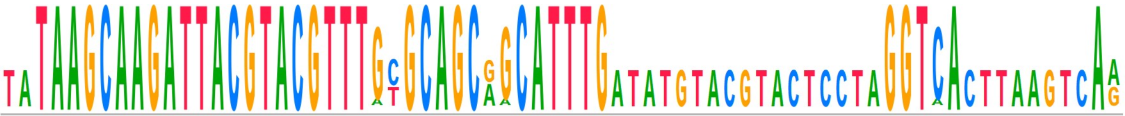

Sequence	Alignment	Position
**Consensus**	--TAAGCAAGATTACGTACGTTTGCGCAGCNGCATTTG-----------------GGTCA--------AA	
	2390 · 2400 · 2410 · 2420 · 2430 · 2440 · 2450	
Lr67(sus)	--TAAGCAAGATTACGTACGTTTACGCAGCAACATTTGATATGTACGTACTCCTAGGTAACTTAAGTCAA	1953
Lr67(res)	--TAAGCAAGATTACGTACGTTTACGCAGCAACATTTGATATGTACGTACTCCTAGGTAACTTAAGTCAA	1953
ScLr67_1 (Lo7)	TATAAGCAAGATTACGTACGTTTGCGCAGCGGCATTTG-----------------GGTCA--------AA	2289
118_Danko_APR	--TAAGCAAGATTACGTACGTTTGTGCAGCAGCATTTG-----------------GGTCA--------AG	2181
119_Danko_APR	--TAAGCAAGATTACGTACGTTTGCGCAGCGGCATTTG-----------------GGTCA--------AA	2179
119_Danko_APR2	--TAAGCAAGATTACGTACGTTTGCGCAGCGGCATTTG-----------------GGTCA--------AA	2179
120_Danko_APR	--TAAGCAAGATTACGTACGTTTGCGCAGCGGCATTTG-----------------GGTCA--------AA	2181
138_Danko_APR	--TAAGCAAGATTACGTACGTTTGCGCAGCGGCATTTG-----------------GGTCA--------AA	2177
153_Danko_APR	--TAAGCAAGATTACGTACGTTTGTGCAGCAGCATTTG-----------------GGTCA--------AG	2175
157_Danko_APR	--TAAGCAAGATTACGTACGTTTGCGCAGCGGCATTTG-----------------GGTCA--------AA	2179
160_Danko_APR	--TAAGCAAGATTACGTACGTTTGCGCAGCGGCATTTG-----------------GGTCA--------AA	2169
71_PHR_APR	--TAAGCAAGATTACGTACGTTTGCGCAGCGGCATTTG-----------------GGTCA--------AA	2248
149_PHR_APR	--TAAGCAAGATTACGTACGTTTGTGCAGCAGCATTTG-----------------GGTCA--------AG	2179
59_Danko_non-APR	--TAAGCAAGATTACGTACGTTTGCGCAGCGGCATTTG-----------------GGTCA--------AA	2200
61_Danko_non-APR	TATAAGCAAGATTACGTACGTTTGCGCAGCGGCATTTG-----------------GGTCA--------AA	2241
123_Danko_non-APR	--TAAGCAAGATTACGTACGTTTGTGCAGCAGCATTTG-----------------GGTCA--------AG	2130
129_Danko_non-APR	--TAAGCAAGATTACGTACGTTTGTGCAGCAGCATTTG-----------------GGTCA--------AG	2177
37_PHR_non-APR	--TAAGCAAGATTACGTACGTTTGCGCAGCGGCATTTG-----------------GGTCA--------AA	2157
52_PHR_non-APR	--TAAGCAAGATTACGTACGTTTGCGCAGCGGCATTTG-----------------GGTCA--------AA	2236
88_PHR_non-APR	--TAAGCAAGATTACGTACGTTTGTGCAGCAGCATTTG-----------------GGTCA--------AG	2158
101_PHR_non-APR	--TAAGCAAGATTACGTACGTTTGTGCAGCAGCATTTG-----------------GGTCA--------AG	2179
105_PHR_non-APR	--TAAGCAAGATTACGTACGTTTGTGCAGCAGCATTTG-----------------GGTCA--------AG	2178
150_PHR_non-APR	--TAAGCAAGATTACGTACGTTTGTGCAGCAGCATTTG-----------------GGTCA--------AG	2120
150_PHR_non-APR2	--TAAGCAAGATTACGTACGTTTGTGCAGCAGCATTTG-----------------GGTCA--------AG	2120

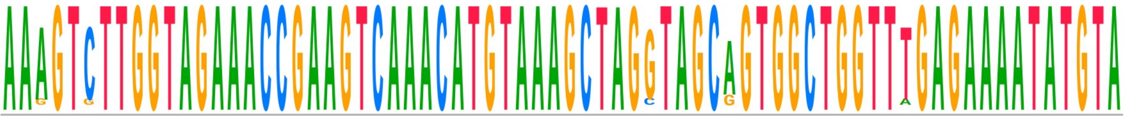

Sequence	Alignment	Position
**Consensus**	AAAGTCTTGGTAGAAACCGAAGTCAAACATGTAAAGCTAGGTAGCAGTGGCTGGTTTGAGAAAATATGTA	
	2460 · 2470 · 2480 · 2490 · 2500 · 2510 · 2520	
Lr67(sus)	AAGGTGTTGGTAGAAACCGAAGTCAAACATGTAAAGC----TAGCGGTGGCTGGTTTGAGAAAATATGTA	2019
Lr67(res)	AAGGTGTTGGTAGAAACCGAAGTCAAACATGTAAAGC----TAGCGGTGGCTGGTTTGAGAAAATATGTA	2019
ScLr67_1 (Lo7)	AAAGTCTTGGTAGAAACCGAAGTCAAACATGTAAAGCTAGCTAGCGGTGGCTGGTTAGAGAAAATATGTA	2359
118_Danko_APR	AAAGTCTTGGTAGAAACCGAAGTCAAACATGTAAAGCTAGGTAGCAGTGGCTGGTTTGAGAAAATATGTA	2251
119_Danko_APR	AAAGTCTTGGTAGAAACCGAAGTCAAACATGTAAAGCTAGGTAGCAGTGGCTGGTTTGAGAAAATATGTA	2249
119_Danko_APR2	AAAGTCTTGGTAGAAACCGAAGTCAAACATGTAAAGCTAGGTAGCAGTGGCTGGTTTGAGAAAATATGTA	2249
120_Danko_APR	AAAGTCTTGGTAGAAACCGAAGTCAAACATGTAAAGCTAGGTAGCAGTGGCTGGTTTGAGAAAATATGTA	2251
138_Danko_APR	AAAGTCTTGGTAGAAACCGAAGTCAAACATGTAAAGCTAGGTAGCAGTGGCTGGTTTGAGAAAATATGTA	2247
153_Danko_APR	AAAGTCTTGGTAGAAACCGAAGTCAAACATGTAAAGCTAGGTAGCAGTGGCTGGTTTGAGAAAATATGTA	2245
157_Danko_APR	AAAGTCTTGGTAGAAACCGAAGTCAAACATGTAAAGCTAGGTAGCAGTGGCTGGTTTGAGAAAATATGTA	2249
160_Danko_APR	AAAGTCTTGGTAGAAACCGAAGTCAAACATGTAAAGCTAGGTAGCAGTGGCTGGTTTGAGAAAATATGTA	2239
71_PHR_APR	AAAGTCTTGGTAGAAACCGAAGTCAAACATGTAAAGCTAGGTAGCAGTGGCTGGTTTGAGAAAATATGTA	2318
149_PHR_APR	AAAGTCTTGGTAGAAACCGAAGTCAAACATGTAAAGCTAGGTAGCAGTGGCTGGTTTGAGAAAATATGTA	2249
59_Danko_non-APR	AAAGTCTTGGTAGAAACCGAAGTCAAACATGTAAAGCTAGGTAGCAGTGGCTGGTTTGAGAAAATATGTA	2270
61_Danko_non-APR	AAAGTCTTGGTAGAAACCGAAGTCAAACATGTAAAGCTAGCTAGCGGTGGCTGGTTAGAGAAAATATGTA	2311
123_Danko_non-APR	AAAGTCTTGGTAGAAACCGAAGTCAAACATGTAAAGCTAGGTAGCAGTGGCTGGTTTGAGAAAATATGTA	2200
129_Danko_non-APR	AAAGTCTTGGTAGAAACCGAAGTCAAACATGTAAAGCTAGGTAGCAGTGGCTGGTTTGAGAAAATATGTA	2247
37_PHR_non-APR	AAAGTCTTGGTAGAAACCGAAGTCAAACATGTAAAGCTAGGTAGCAGTGGCTGGTTTGAGAAAATATGTA	2227
52_PHR_non-APR	AAAGTCTTGGTAGAAACCGAAGTCAAACATGTAAAGCTAGGTAGCAGTGGCTGGTTTGAGAAAATATGTA	2306
88_PHR_non-APR	AAAGTCTTGGTAGAAACCGAAGTCAAACATGTAAAGCTAGGTAGCAGTGGCTGGTTTGAGAAAATATGTA	2228
101_PHR_non-APR	AAAGTCTTGGTAGAAACCGAAGTCAAACATGTAAAGCTAGGTAGCAGTGGCTGGTTTGAGAAAATATGTA	2249
105_PHR_non-APR	AAAGTCTTGGTAGAAACCGAAGTCAAACATGTAAAGCTAGGTAGCAGTGGCTGGTTTGAGAAAATATGTA	2248
150_PHR_non-APR	AAAGTCTTGGTAGAAACCGAAGTCAAACATGTAAAGCTAGGTAGCAGTGGCTGGTTTGAGAAAATATGTA	2190
150_PHR_non-APR2	AAAGTCTTGGTAGAAACCGAAGTCAAACATGTAAAGCTAGGTAGCAGTGGCTGGTTTGAGAAAATATGTA	2190

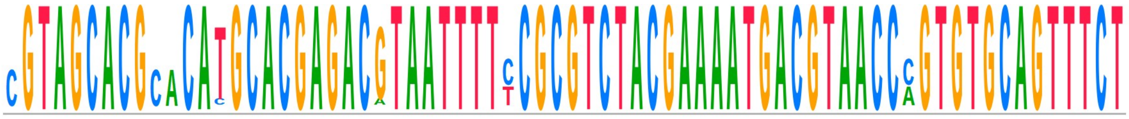

```
Consensus            -GTAGCACG--CATGCACGAGACGTAATTTTCCGCGTCTACGAAAATGACGTAACCCGTGTGCAGTTTCT
                              2530      2540      2550      2560      2570      2580      2590
Lr67(sus)            CGTAGCACGCACACGCACGAGACATAATTTTCCGCGTCTACGAAAATGACGTAACCCGTGTGCAGTTTCT  2089
Lr67(res)            CGTAGCACGCACACGCACGAGACATAATTTTCCGCGTCTACGAAAATGACGTAACCCGTGTGCAGTTTCT  2089
ScLr67_1 (Lo7)       -GTAGCACG--CATGCACGAGACGTAATTTTCCGCGTCTACGAAAATGACGTAACCCGTGTGCAGTTTCT  2426
118_Danko_APR        -GTAGCACG--CATGCACGAGACGTAATTTTTCGCGTCTACGAAAATGACGTAACCAGTGTGCAGTTTCT  2318
119_Danko_APR        -GTAGCACG--CATGCACGAGACGTAATTTTCCGCGTCTACGAAAATGACGTAACCCGTGTGCAGTTTCT  2316
119_Danko_APR2       -GTAGCACG--CATGCACGAGACGTAATTTTCCGCGTCTACGAAAATGACGTAACCCGTGTGCAGTTTCT  2316
120_Danko_APR        -GTAGCACG--CATGCACGAGACGTAATTTTCCGCGTCTACGAAAATGACGTAACCCGTGTGCAGTTTCT  2318
138_Danko_APR        -GTAGCACG--CATGCACGAGACGTAATTTTCCGCGTCTACGAAAATGACGTAACCCGTGTGCAGTTTCT  2314
153_Danko_APR        -GTAGCACG--CATGCACGAGACGTAATTTTTCGCGTCTACGAAAATGACGTAACCAGTGTGCAGTTTCT  2312
157_Danko_APR        -GTAGCACG--CATGCACGAGACGTAATTTTCCGCGTCTACGAAAATGACGTAACCCGTGTGCAGTTTCT  2316
160_Danko_APR        -GTAGCACG--CATGCACGAGACGTAATTTTCCGCGTCTACGAAAATGACGTAACCCGTGTGCAGTTTCT  2306
71_PHR_APR           -GTAGCACG--CATGCACGAGACGTAATTTTCCGCGTCTACGAAAATGACGTAACCCGTGTGCAGTTTCT  2385
149_PHR_APR          -GTAGCACG--CATGCACGAGACGTAATTTTTCGCGTCTACGAAAATGACGTAACCAGTGTGCAGTTTCT  2316
59_Danko_non-APR     -GTAGCACG--CATGCACGAGACGTAATTTTCCGCGTCTACGAAAATGACGTAACCCGTGTGCAGTTTCT  2337
61_Danko_non-APR     -GTAGCACG--CATGCACGAGACGTAATTTTCCGCGTCTACGAAAATGACGTAACCCGTGTGCAGTTTCT  2378
123_Danko_non-APR    -GTAGCACG--CATGCACGAGACGTAATTTTTCGCGTCTACGAAAATGACGTAACCAGTGTGCAGTTTCT  2267
129_Danko_non-APR    -GTAGCACG--CATGCACGAGACGTAATTTTTCGCGTCTACGAAAATGACGTAACCAGTGTGCAGTTTCT  2314
37_PHR_non-APR       -GTAGCACG--CATGCACGAGACGTAATTTTCCGCGTCTACGAAAATGACGTAACCCGTGTGCAGTTTCT  2294
52_PHR_non-APR       -GTAGCACG--CATGCACGAGACGTAATTTTCCGCGTCTACGAAAATGACGTAACCCGTGTGCAGTTTCT  2373
88_PHR_non-APR       -GTAGCACG--CATGCACGAGACGTAATTTTTCGCGTCTACGAAAATGACGTAACCAGTGTGCAGTTTCT  2295
101_PHR_non-APR      -GTAGCACG--CATGCACGAGACGTAATTTTTCGCGTCTACGAAAATGACGTAACCAGTGTGCAGTTTCT  2316
105_PHR_non-APR      -GTAGCACG--CATGCACGAGACGTAATTTTTCGCGTCTACGAAAATGACGTAACCAGTGTGCAGTTTCT  2315
150_PHR_non-APR      -GTAGCACG--CATGCACGAGACGTAATTTTTCGCGTCTACGAAAATGACGTAACCAGTGTGCAGTTTCT  2257
150_PHR_non-APR2     -GTAGCACG--CATGCACGAGACGTAATTTTTCGCGTCTACGAAAATGACGTAACCAGTGTGCAGTTTCT  2257
```

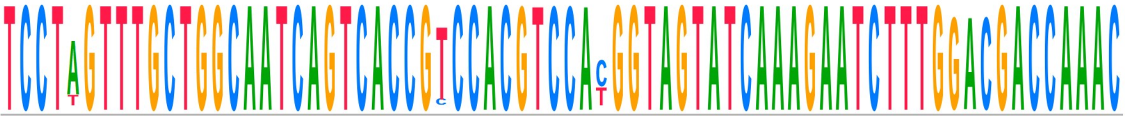

```
Consensus            TCCTAGTTTGCTGGCAATCAGTCACCGTCCACGTCCACGGTAGTATCAAAGAATCTTTG---GACCAAAC
                              2600      2610      2620      2630      2640      2650      2660
Lr67(sus)            TCCTTGTTTGCTGGCAATCAGTCACCGCCCAC------GGTAGTATCAAAGAATCTTTG---GACCAAAC  2150
Lr67(res)            TCCTTGTTTGCTGGCAATCAGTCACCGCCCAC------GGTAGTATCAAAGAATCTTTG---GACCAAAC  2150
ScLr67_1 (Lo7)       TCCTTGTTTGCTGGCAATCAGTCACCGTCCACGTCCACGGTAGTATCAAAGAATCTTTGGACGACCAAAC  2496
118_Danko_APR        TCCTAGTTTGCTGGCAATCAGTCACCGTCCACGTCCACGGTAGTATCAAAGAATCTTTG---GACCAAAC  2385
119_Danko_APR        TCCTAGTTTGCTGGCAATCAGTCACCGTCCACGTCCATGGTAGTATCAAAGAATCTTTGGACGACCAAAC  2386
119_Danko_APR2       TCCTAGTTTGCTGGCAATCAGTCACCGTCCACGTCCATGGTAGTATCAAAGAATCTTTGGACGACCAAAC  2386
120_Danko_APR        TCCTAGTTTGCTGGCAATCAGTCACCGTCCACGTCCATGGTAGTATCAAAGAATCTTTGGACGACCAAAC  2388
138_Danko_APR        TCCTAGTTTGCTGGCAATCAGTCACCGTCCACGTCCATGGTAGTATCAAAGAATCTTTGGACGACCAAAC  2384
153_Danko_APR        TCCTAGTTTGCTGGCAATCAGTCACCGTCCACGTCCACGGTAGTATCAAAGAATCTTTG---GACCAAAC  2379
157_Danko_APR        TCCTAGTTTGCTGGCAATCAGTCACCGTCCACGTCCATGGTAGTATCAAAGAATCTTTGGACGACCAAAC  2386
160_Danko_APR        TCCTAGTTTGCTGGCAATCAGTCACCGTCCACGTCCATGGTAGTATCAAAGAATCTTTGGACGACCAAAC  2376
71_PHR_APR           TCCTAGTTTGCTGGCAATCAGTCACCGTCCACGTCCATGGTAGTATCAAAGAATCTTTGGACGACCAAAC  2455
149_PHR_APR          TCCTAGTTTGCTGGCAATCAGTCACCGTCCACGTCCACGGTAGTATCAAAGAATCTTTG---GACCAAAC  2383
59_Danko_non-APR     TCCTAGTTTGCTGGCAATCAGTCACCGTCCACGTCCATGGTAGTATCAAAGAATCTTTGGACGACCAAAC  2407
61_Danko_non-APR     TCCTTGTTTGCTGGCAATCAGTCACCGTCCACGTCCACGGTAGTATCAAAGAATCTTTGGACGACCAAAC  2448
123_Danko_non-APR    TCCTAGTTTGCTGGCAATCAGTCACCGTCCACGTCCACGGTAGTATCAAAGAATCTTTG---GACCAAAC  2334
129_Danko_non-APR    TCCTAGTTTGCTGGCAATCAGTCACCGTCCACGTCCACGGTAGTATCAAAGAATCTTTG---GACCAAAC  2381
37_PHR_non-APR       TCCTAGTTTGCTGGCAATCAGTCACCGTCCACGTCCATGGTAGTATCAAAGAATCTTTGGACGACCAAAC  2364
52_PHR_non-APR       TCCTAGTTTGCTGGCAATCAGTCACCGTCCACGTCCACGGTAGTATCAAAGAATCTTTG---GACCAAAC  2440
88_PHR_non-APR       TCCTAGTTTGCTGGCAATCAGTCACCGTCCACGTCCACGGTAGTATCAAAGAATCTTTG---GACCAAAC  2362
101_PHR_non-APR      TCCTAGTTTGCTGGCAATCAGTCACCGTCCACGTCCACGGTAGTATCAAAGAATCTTTG---GACCAAAC  2383
105_PHR_non-APR      TCCTAGTTTGCTGGCAATCAGTCACCGTCCACGTCCACGGTAGTATCAAAGAATCTTTG---GACCAAAC  2382
150_PHR_non-APR      TCCTAGTTTGCTGGCAATCAGTCACCGTCCACGTCCACGGTAGTATCAAAGAATCTTTG---GACCAAAC  2324
150_PHR_non-APR2     TCCTAGTTTGCTGGCAATCAGTCACCGTCCACGTCCACGGTAGTATCAAAGAATCTTTG---GACCAAAC  2324
```

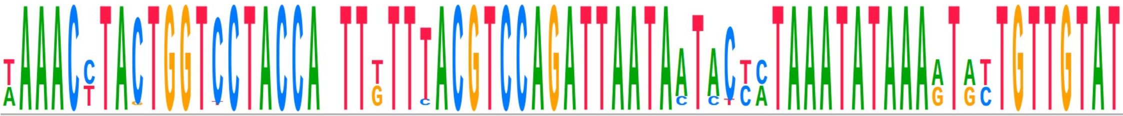

Consensus	TAAACCTACTGGTCCTACCA-TTTTTTACGTCCAGATTAATANTNCTCTAAATATAAAATATTGTTGTAT	
	2670 2680 2690 2700 2710 2720 2730	
Lr67(sus)	TAAACCTACTGGTCCTACCA-TTTTTCACGTCCAGATTAATACTCTTCTAAATATAAAATATTGTTGTAT	2219
Lr67(res)	TAAACCTACTGGTCCTACCA-TTTTTCACGTCCAGATTAATACTCTTCTAAATATAAAATATTGTTGTAT	2219
ScLr67_1 (Lo7)	TAAACTTACTGGTCCTACCA-TTGTTTACGTCCAGATTAATAATACCATAAATATAAAATATTGTTGTAT	2565
118_Danko_APR	AAAACCTACTGGTCCTACCA-TTTTTTACGTCCAGATTAATA---CTCTAAATATAAAGTGCTGTTGTAT	2451
119_Danko_APR	TAAACTTACTGGTCCTACCA-TTGTTTACGTCCAGATTAATAATACCATAAATATAAAATATTGTTGTAT	2455
119_Danko_APR2	TAAACTTACTGGTCCTACCA-TTGTTTACGTCCAGATTAATAATACCATAAATATAAAATATTGTTGTAT	2455
120_Danko_APR	TAAACTTACTGGTCCTACCA-TTGTTTACGTCCAGATTAATAATACCATAAATATAAAATATTGTTGTAT	2457
138_Danko_APR	TAAACTTACTGGTCCTACCA-TTGTTTACGTCCAGATTAATAATACCATAAATATAAAATATTGTTGTAT	2453
153_Danko_APR	AAAACCTACTGGTCCTACCA-TTTTTTACGTCCAGATTAATA---CTCTAAATATAAAGTGCTGTTGTAT	2445
157_Danko_APR	TAAACTTACTGGTCCTACCA-TTGTTTACGTCCAGATTAATAATACCATAAATATAAAATATTGTTGTAT	2455
160_Danko_APR	TAAACTTACTGGTCCTACCA-TTGTTTACGTCCAGATTAATAATACCATAAATATAAAATATTGTTGTAT	2445
71_PHR_APR	TAAACTTACTGGTCCTACCA-TTGTTTACGTCCAGATTAATAATACCATAAATATAAAATATTGTTGTAT	2524
149_PHR_APR	AAAACCTACTGGTCCTACCA-TTTTTTACGTCCAGATTAATA---CTCTAAATATAAAGTGCTGTTGTAT	2449
59_Danko_non-APR	TAAACTTACTGGTCCTACCA-TTGTTTACGTCCAGATTAATAATACCATAAATATAAAATATTGTTGTAT	2476
61_Danko_non-APR	TAAACTTACTGGTCCTACCA-TTGTTTACGTCCAGATTAATAATACCATAAATATAAAATATTGTTGTAT	2517
123_Danko_non-APR	AAAACCTACTGGTCCTACCA-TTTTTTACGTCCAGATTAATA---CTCTAAATATAAAGTGCTGTTGTAT	2400
129_Danko_non-APR	AAAACCTACTGGTCCTACCA-TTTTTTACGTCCAGATTAATA---CTCTAAATATAAAGTGCTGTTGTAT	2447
37_PHR_non-APR	TAAACTTACTGGTCCTACCA-TTGTTTACGTCCAGATTAATAATACCATAAATATAAAATATTGTTGTAT	2433
52_PHR_non-APR	TAAACCTAGTGGTTCTACCATTTTTTTACGTCCAGATTAATA---CTCTAAATATAAAATATTGTTGTAT	2507
88_PHR_non-APR	AAAACCTACTGGTCCTACCA-TTTTTTACGTCCAGATTAATA---CTCTAAATATAAAGTGCTGTTGTAT	2428
101_PHR_non-APR	AAAACCTACTGGTCCTACCA-TTTTTTACGTCCAGATTAATA---CTCTAAATATAAAGTGCTGTTGTAT	2449
105_PHR_non-APR	AAAACCTACTGGTCCTACCA-TTTTTTACGTCCAGATTAATA---CTCTAAATATAAAGTGCTGTTGTAT	2448
150_PHR_non-APR	AAAACCTACTGGTCCTACCA-TTTTTTACGTCCAGATTAATA---CTCTAAATATAAAGTGCTGTTGTAT	2390
150_PHR_non-APR2	AAAACCTACTGGTCCTACCA-TTTTTTACGTCCAGATTAATA---CTCTAAATATAAAGTGCTGTTGTAT	2390

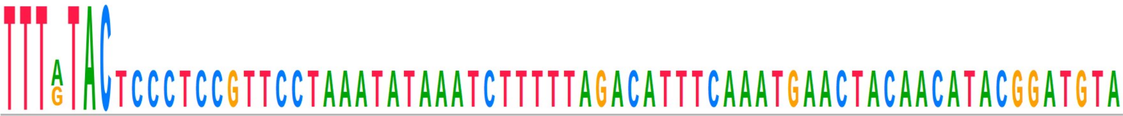

Consensus	TTTATAC---	
	2740 2750 2760 2770 2780 2790 2800	
Lr67(sus)	TTTATACTCCCTCCGTTCCTAAATATAAATCTTTTTAGACATTTCAAATGAACTACAACATACGGATGTA	2289
Lr67(res)	TTTATACTCCCTCCGTTCCTAAATATAAATCTTTTTAGACATTTCAAATGAACTACAACATACGGATGTA	2289
ScLr67_1 (Lo7)	TTTATAC---	2572
118_Danko_APR	TTTGTAC---	2458
119_Danko_APR	TTTATAC---	2462
119_Danko_APR2	TTTATAC---	2462
120_Danko_APR	TTTATAC---	2464
138_Danko_APR	TTTATAC---	2460
153_Danko_APR	TTTGTAC---	2452
157_Danko_APR	TTTATAC---	2462
160_Danko_APR	TTTATAC---	2452
71_PHR_APR	TTTATAC---	2531
149_PHR_APR	TTTGTAC---	2456
59_Danko_non-APR	TTTATAC---	2483
61_Danko_non-APR	TTTATAC---	2524
123_Danko_non-APR	TTTGTAC---	2407
129_Danko_non-APR	TTTGTAC---	2454
37_PHR_non-APR	TTTATAC---	2440
52_PHR_non-APR	TTTATAC---	2514
88_PHR_non-APR	TTTGTAC---	2435
101_PHR_non-APR	TTTGTAC---	2456
105_PHR_non-APR	TTTGTAC---	2455
150_PHR_non-APR	TTTGTAC---	2397
150_PHR_non-APR2	TTTGTAC---	2397

```
                   TGTAGGCATATTTTAGAGTGTAGATTCACTCATTTTGCTCCGTATTCGGTCACTTGTTGGAAAGACTTAT
Consensus          ----------------------------------------------------------------------
                            2810      2820      2830      2840      2850      2860      2870
Lr67(sus)          TGTAGGCATATTTTAGAGTGTAGATTCACTCATTTTGCTCCGTATTCGGTCACTTGTTGGAAAGACTTAT  2359
Lr67(res)          TGTAGGCATATTTTAGAGTGTAGATTCACTCATTTTGCTCCGTATTCGGTCACTTGTTGGAAAGACTTAT  2359
ScLr67_1 (Lo7)     ----------------------------------------------------------------------  2572
118_Danko_APR      ----------------------------------------------------------------------  2458
119_Danko_APR      ----------------------------------------------------------------------  2462
119_Danko_APR2     ----------------------------------------------------------------------  2462
120_Danko_APR      ----------------------------------------------------------------------  2464
138_Danko_APR      ----------------------------------------------------------------------  2460
153_Danko_APR      ----------------------------------------------------------------------  2452
157_Danko_APR      ----------------------------------------------------------------------  2462
160_Danko_APR      ----------------------------------------------------------------------  2452
71_PHR_APR         ----------------------------------------------------------------------  2531
149_PHR_APR        ----------------------------------------------------------------------  2456
59_Danko_non-APR   ----------------------------------------------------------------------  2483
61_Danko_non-APR   ----------------------------------------------------------------------  2524
123_Danko_non-APR  ----------------------------------------------------------------------  2407
129_Danko_non-APR  ----------------------------------------------------------------------  2454
37_PHR_non-APR     ----------------------------------------------------------------------  2440
52_PHR_non-APR     ----------------------------------------------------------------------  2514
88_PHR_non-APR     ----------------------------------------------------------------------  2435
101_PHR_non-APR    ----------------------------------------------------------------------  2456
105_PHR_non-APR    ----------------------------------------------------------------------  2455
150_PHR_non-APR    ----------------------------------------------------------------------  2397
150_PHR_non-APR2   ----------------------------------------------------------------------  2397

                   ATTTGGGAACGGAGGGAGTATGTACTAGCTGCAGTACGTATGTAGGTACATCGTATCCCTCTTGTGTTTC
Consensus          ---------------------GTACTAGCTGCAGTACGTATGTAGGTACATCGTATCCCTCTTGTGTTTC
                            2880      2890      2900      2910      2920      2930      2940
Lr67(sus)          ATTTGGGAACGGAGGGAGTATGTACTAGCTGCAGTACGTATGTAGGTACATCGTATCCCTCTTATATTTC  2429
Lr67(res)          ATTTGGGAACGGAGGGAGTATGTACTAGCTGCAGTACGTATGTAGGTACATCGTATCCCTCTTATATTTC  2429
ScLr67_1 (Lo7)     ---------------------GTATTAGCTGCAGTACGTATGTAGGCACATCGTATCCCTCTTGTGTTTC  2621
118_Danko_APR      ---------------------GTACTAGCTGCAGTACGTATGTAGGTAC-TCGTATCCCTCTTGTGTTTC  2506
119_Danko_APR      ---------------------GTACTAGCTGCAGTACGTATGTAGGTACATCGTATCCCTCTTGTGTTTC  2511
119_Danko_APR2     ---------------------GTACTAGCTGCAGTACGTATGTAGGTACATCGTATCCCTCTTGTGTTTC  2511
120_Danko_APR      ---------------------GTACTAGCTGCAGTACGTATGTAGGTACATCGTATCCCTCTTGTGTTTC  2513
138_Danko_APR      ---------------------GTACTAGCTGCAGTACGTATGTAGGTACATCGTATCCCTCTTGTGTTTC  2509
153_Danko_APR      ---------------------GTACTAGCTGCAGTACGTATGTAGGTAC-TCGTATCCCTCTTGTGTTTC  2500
157_Danko_APR      ---------------------GTACTAGCTGCAGTACGTATGTAGGTACATCGTATCCCTCTTGTGTTTC  2511
160_Danko_APR      ---------------------GTACTAGCTGCAGTACGTATGTAGGTACATCGTATCCCTCTTGTGTTTC  2501
71_PHR_APR         ---------------------GTACTAGCTGCAGTACGTATGTAGGTACATCGTATCCCTCTTGTGTTTC  2580
149_PHR_APR        ---------------------GTACTAGCTGCAGTACGTATGTAGGTAC-TCGTATCCCTCTTGTGTTTC  2504
59_Danko_non-APR   ---------------------GTACTAGCTGCAGTACGTATGTAGGTACATCGTATCCCTCTTGTGTTTC  2532
61_Danko_non-APR   ---------------------GTATTAGCTGCAGTACGTATGTAGGCACATCGTATCCCTCTTGTGTTTC  2573
123_Danko_non-APR  ---------------------GTACTAGCTGCAGTACGTATGTAGGTAC-TCGTATCCCTCTTGTGTTTC  2455
129_Danko_non-APR  ---------------------GTACTAGCTGCAGTACGTATGTAGGTAC-TCGTATCCCTCTTGTGTTTC  2502
37_PHR_non-APR     ---------------------GTACTAGCTGCAGTACGTATGTAGGTACATCGTATCCCTCTTGTGTTTC  2489
52_PHR_non-APR     ---------------------GTACTAGCTGCAGTACGTATGTAGGTACATCGTACCCCTCTTGTGTTTC  2563
88_PHR_non-APR     ---------------------GTACTAGCTGCAGTACGTATGTAGGTAC-TCGTATCCCTCTTGTGTTTC  2483
101_PHR_non-APR    ---------------------GTACTAGCTGCAGTACGTATGTAGGTAC-TCGTATCCCTCTTGTGTTTC  2504
105_PHR_non-APR    ---------------------GTACTAGCTGCAGTACGTATGTAGGTAC-TCGTATCCCTCTTGTGTTTC  2503
150_PHR_non-APR    ---------------------GTACTAGCTGCAGTACGTATGTAGGTAC-TCGTATCCCTCTTGTGTTTC  2445
150_PHR_non-APR2   ---------------------GTACTAGCTGCAGTACGTATGTAGGTAC-TCGTATCCCTCTTGTGTTTC  2445
```

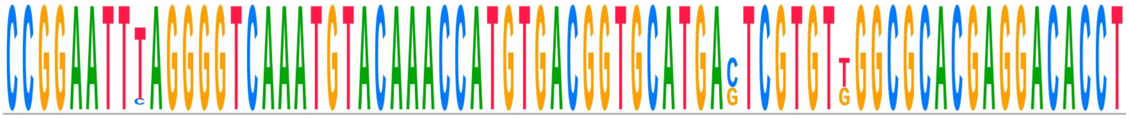

**Consensus**	CCGGAATTTAGGGGTCAAATGTACAAACCATGTGACGGTGCATGACTCGTGTTGGCGCACGAGGACACCT	
	2950 2960 2970 2980 2990 3000 3010	
Lr67(sus)	CCGGAATTCAGGGGTCAAATGTACAAACCATGTGACGGTGCATGACTCGTGTTGGCGCACGAGGACACCT	2499
Lr67(res)	CCGGAATTCAGGGGTCAAATGTACAAACCATGTGACGGTGCATGACTCGTGTTGGCGCACGAGGACACCT	2499
ScLr67_1 (Lo7)	CCGGAATTTAGGGGTCAAATGTACAAACCATGTGACGGTGCATGACTCGTGTTGGCGCACGAGGACACCT	2691
118_Danko_APR	CCGGAATTTAGGGGTCAAATGTACAAACCATGTGACGGTGCATGACTCGTGTTGGCGCACGAGGACACCT	2576
119_Danko_APR	CCGGAATTTAGGGGTCAAATGTACAAACCATGTGACGGTGCATGAGTCGTGTGGGCGCACGAGGACACCT	2581
119_Danko_APR2	CCGGAATTTAGGGGTCAAATGTACAAACCATGTGACGGTGCATGAGTCGTGTGGGCGCACGAGGACACCT	2581
120_Danko_APR	CCGGAATTTAGGGGTCAAATGTACAAACCATGTGACGGTGCATGAGTCGTGTGGGCGCACGAGGACACCT	2583
138_Danko_APR	CCGGAATTTAGGGGTCAAATGTACAAACCATGTGACGGTGCATGAGTCGTGTGGGCGCACGAGGACACCT	2579
153_Danko_APR	CCGGAATTTAGGGGTCAAATGTACAAACCATGTGACGGTGCATGACTCGTGTTGGCGCACGAGGACACCT	2570
157_Danko_APR	CCGGAATTTAGGGGTCAAATGTACAAACCATGTGACGGTGCATGAGTCGTGTGGGCGCACGAGGACACCT	2581
160_Danko_APR	CCGGAATTTAGGGGTCAAATGTACAAACCATGTGACGGTGCATGAGTCGTGTGGGCGCACGAGGACACCT	2571
71_PHR_APR	CCGGAATTTAGGGGTCAAATGTACAAACCATGTGACGGTGCATGAGTCGTGTGGGCGCACGAGGACACCT	2650
149_PHR_APR	CCGGAATTTAGGGGTCAAATGTACAAACCATGTGACGGTGCATGACTCGTGTTGGCGCACGAGGACACCT	2574
59_Danko_non-APR	CCGGAATTTAGGGGTCAAATGTACAAACCATGTGACGGTGCATGAGTCGTGTGGGCGCACGAGGACACCT	2602
61_Danko_non-APR	CCGGAATTTAGGGGTCAAATGTACAAACCATGTGACGGTGCATGACTCGTGTTGGCGCACGAGGACACCT	2643
123_Danko_non-APR	CCGGAATTTAGGGGTCAAATGTACAAACCATGTGACGGTGCATGACTCGTGTTGGCGCACGAGGACACCT	2525
129_Danko_non-APR	CCGGAATTTAGGGGTCAAATGTACAAACCATGTGACGGTGCATGACTCGTGTTGGCGCACGAGGACACCT	2572
37_PHR_non-APR	CCGGAATTTAGGGGTCAAATGTACAAACCATGTGACGGTGCATGAGTCGTGTGGGCGCACGAGGACACCT	2559
52_PHR_non-APR	CCGGAATTTAGGGGTCAAATGTACAAACCATGTGACGGTGCATGACTCGTGTTGGCGCACGAGGACACCT	2633
88_PHR_non-APR	CCGGAATTTAGGGGTCAAATGTACAAACCATGTGACGGTGCATGACTCGTGTTGGCGCACGAGGACACCT	2553
101_PHR_non-APR	CCGGAATTTAGGGGTCAAATGTACAAACCATGTGACGGTGCATGACTCGTGTTGGCGCACGAGGACACCT	2574
105_PHR_non-APR	CCGGAATTTAGGGGTCAAATGTACAAACCATGTGACGGTGCATGACTCGTGTTGGCGCACGAGGACACCT	2573
150_PHR_non-APR	CCGGAATTTAGGGGTCAAATGTACAAACCATGTGACGGTGCATGACTCGTGTTGGCGCACGAGGACACCT	2515
150_PHR_non-APR2	CCGGAATTTAGGGGTCAAATGTACAAACCATGTGACGGTGCATGACTCGTGTTGGCGCACGAGGACACCT	2515

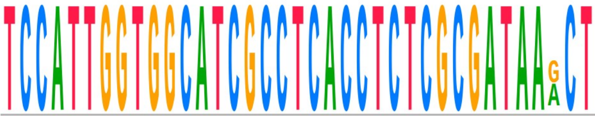

**Consensus**	TCCATTGGTGGCATCGCCTCACCTCTCGCGATAANCT---------------------------------	
	3020 3030 3040 3050 3060 3070 3080	
Lr67(sus)	TCCATTGGTGGCATCGCCTCACCTCTCGCGATAAACT---------------------------------	2536
Lr67(res)	TCCATTGGTGGCATCGCCTCACCTCTCGCGATAAACT---------------------------------	2536
ScLr67_1 (Lo7)	TCCATTGGTGGCATCGCCTCACCTCTCGCGATAAGCT---------------------------------	2728
118_Danko_APR	TCCATTGGTGGCATCGCCTCACCTCTCGCGATAAGCT---------------------------------	2613
119_Danko_APR	TCCATTGGTGGCATCGCCTCACCTCTCGCGATAAACT---------------------------------	2618
119_Danko_APR2	TCCATTGGTGGCATCGCCTCACCTCTCGCGATAAACT---------------------------------	2618
120_Danko_APR	TCCATTGGTGGCATCGCCTCACCTCTCGCGATAAACT---------------------------------	2620
138_Danko_APR	TCCATTGGTGGCATCGCCTCACCTCTCGCGATAAACT---------------------------------	2616
153_Danko_APR	TCCATTGGTGGCATCGCCTCACCTCTCGCGATAAGCT---------------------------------	2607
157_Danko_APR	TCCATTGGTGGCATCGCCTCACCTCTCGCGATAAACT---------------------------------	2618
160_Danko_APR	TCCATTGGTGGCATCGCCTCACCTCTCGCGATAAACT---------------------------------	2608
71_PHR_APR	TCCATTGGTGGCATCGCCTCACCTCTCGCGATAAACT---------------------------------	2687
149_PHR_APR	TCCATTGGTGGCATCGCCTCACCTCTCGCGATAAGCT---------------------------------	2611
59_Danko_non-APR	TCCATTGGTGGCATCGCCTCACCTCTCGCGATAAACT---------------------------------	2639
61_Danko_non-APR	TCCATTGGTGGCATCGCCTCACCTCTCGCGATAAGCT---------------------------------	2680
123_Danko_non-APR	TCCATTGGTGGCATCGCCTCACCTCTCGCGATAAGCT---------------------------------	2562
129_Danko_non-APR	TCCATTGGTGGCATCGCCTCACCTCTCGCGATAAGCT---------------------------------	2609
37_PHR_non-APR	TCCATTGGTGGCATCGCCTCACCTCTCGCGATAAACT---------------------------------	2596
52_PHR_non-APR	TCCATTGGTGGCATCGCCTCACCTCTCGCGATAAACTTGTTAGACTAGTCATAGTGGGGAGTAACTTACA	2703
88_PHR_non-APR	TCCATTGGTGGCATCGCCTCACCTCTCGCGATAAGCT---------------------------------	2590
101_PHR_non-APR	TCCATTGGTGGCATCGCCTCACCTCTCGCGATAAGCT---------------------------------	2611
105_PHR_non-APR	TCCATTGGTGGCATCGCCTCACCTCTCGCGATAAGCT---------------------------------	2610
150_PHR_non-APR	TCCATTGGTGGCATCGCCTCACCTCTCGCGATAAGCT---------------------------------	2552
150_PHR_non-APR2	TCCATTGGTGGCATCGCCTCACCTCTCGCGATAAGCT---------------------------------	2552

```
Consensus           ----------------------------------------------------------------------
                         3090      3100      3110      3120      3130      3140      3150
Lr67(sus)           ----------------------------------------------------------------------  2536
Lr67(res)           ----------------------------------------------------------------------  2536
ScLr67_1 (Lo7)      ----------------------------------------------------------------------  2728
118_Danko_APR       ----------------------------------------------------------------------  2613
119_Danko_APR       ----------------------------------------------------------------------  2618
119_Danko_APR2      ----------------------------------------------------------------------  2618
120_Danko_APR       ----------------------------------------------------------------------  2620
138_Danko_APR       ----------------------------------------------------------------------  2616
153_Danko_APR       ----------------------------------------------------------------------  2607
157_Danko_APR       ----------------------------------------------------------------------  2618
160_Danko_APR       ----------------------------------------------------------------------  2608
71_PHR_APR          ----------------------------------------------------------------------  2687
149_PHR_APR         ----------------------------------------------------------------------  2611
59_Danko_non-APR    ----------------------------------------------------------------------  2639
61_Danko_non-APR    ----------------------------------------------------------------------  2680
123_Danko_non-APR   ----------------------------------------------------------------------  2562
129_Danko_non-APR   ----------------------------------------------------------------------  2609
37_PHR_non-APR      ----------------------------------------------------------------------  2596
52_PHR_non-APR      CTAGTAACATACACGTATTACTAGACTATGTTACTACCTCCATAGTGGGTAGACTAGTCATAGTGGGGAG  2773
88_PHR_non-APR      ----------------------------------------------------------------------  2590
101_PHR_non-APR     ----------------------------------------------------------------------  2611
105_PHR_non-APR     ----------------------------------------------------------------------  2610
150_PHR_non-APR     ----------------------------------------------------------------------  2552
150_PHR_non-APR2    ----------------------------------------------------------------------  2552
```

```
Consensus           ----------------------------------------------------------------------
                         3160      3170      3180      3190      3200      3210      3220
Lr67(sus)           ----------------------------------------------------------------------  2536
Lr67(res)           ----------------------------------------------------------------------  2536
ScLr67_1 (Lo7)      ----------------------------------------------------------------------  2728
118_Danko_APR       ----------------------------------------------------------------------  2613
119_Danko_APR       ----------------------------------------------------------------------  2618
119_Danko_APR2      ----------------------------------------------------------------------  2618
120_Danko_APR       ----------------------------------------------------------------------  2620
138_Danko_APR       ----------------------------------------------------------------------  2616
153_Danko_APR       ----------------------------------------------------------------------  2607
157_Danko_APR       ----------------------------------------------------------------------  2618
160_Danko_APR       ----------------------------------------------------------------------  2608
71_PHR_APR          ----------------------------------------------------------------------  2687
149_PHR_APR         ----------------------------------------------------------------------  2611
59_Danko_non-APR    ----------------------------------------------------------------------  2639
61_Danko_non-APR    ----------------------------------------------------------------------  2680
123_Danko_non-APR   ----------------------------------------------------------------------  2562
129_Danko_non-APR   ----------------------------------------------------------------------  2609
37_PHR_non-APR      ----------------------------------------------------------------------  2596
52_PHR_non-APR      TAACTTACACTAGTAACATACACGTATTACTAGACTATGTTACTACCTCCATAGTGGGTAGAAACATAGG  2843
88_PHR_non-APR      ----------------------------------------------------------------------  2590
101_PHR_non-APR     ----------------------------------------------------------------------  2611
105_PHR_non-APR     ----------------------------------------------------------------------  2610
150_PHR_non-APR     ----------------------------------------------------------------------  2552
150_PHR_non-APR2    ----------------------------------------------------------------------  2552
```

```
Consensus            ----------------------------------------------------------------------
                               3230      3240      3250      3260      3270      3280      3290
Lr67(sus)            ----------------------------------------------------------------------  2536
Lr67(res)            ----------------------------------------------------------------------  2536
ScLr67_1 (Lo7)       ----------------------------------------------------------------------  2728
118_Danko_APR        ----------------------------------------------------------------------  2613
119_Danko_APR        ----------------------------------------------------------------------  2618
119_Danko_APR2       ----------------------------------------------------------------------  2618
120_Danko_APR        ----------------------------------------------------------------------  2620
138_Danko_APR        ----------------------------------------------------------------------  2616
153_Danko_APR        ----------------------------------------------------------------------  2607
157_Danko_APR        ----------------------------------------------------------------------  2618
160_Danko_APR        ----------------------------------------------------------------------  2608
71_PHR_APR           ----------------------------------------------------------------------  2687
149_PHR_APR          ----------------------------------------------------------------------  2611
59_Danko_non-APR     ----------------------------------------------------------------------  2639
61_Danko_non-APR     ----------------------------------------------------------------------  2680
123_Danko_non-APR    ----------------------------------------------------------------------  2562
129_Danko_non-APR    ----------------------------------------------------------------------  2609
37_PHR_non-APR       ----------------------------------------------------------------------  2596
52_PHR_non-APR       TGTGGTGTCATGCAAGAGTTCATTTATTAGTCTATAGACTCAATTTGCATTGGTATGTGTGATGTTACAG  2913
88_PHR_non-APR       ----------------------------------------------------------------------  2590
101_PHR_non-APR      ----------------------------------------------------------------------  2611
105_PHR_non-APR      ----------------------------------------------------------------------  2610
150_PHR_non-APR      ----------------------------------------------------------------------  2552
150_PHR_non-APR2     ----------------------------------------------------------------------  2552
```

```
Consensus            ----------------------------------------------------------------------
                               3300      3310      3320      3330      3340      3350      3360
Lr67(sus)            ----------------------------------------------------------------------  2536
Lr67(res)            ----------------------------------------------------------------------  2536
ScLr67_1 (Lo7)       ----------------------------------------------------------------------  2728
118_Danko_APR        ----------------------------------------------------------------------  2613
119_Danko_APR        ----------------------------------------------------------------------  2618
119_Danko_APR2       ----------------------------------------------------------------------  2618
120_Danko_APR        ----------------------------------------------------------------------  2620
138_Danko_APR        ----------------------------------------------------------------------  2616
153_Danko_APR        ----------------------------------------------------------------------  2607
157_Danko_APR        ----------------------------------------------------------------------  2618
160_Danko_APR        ----------------------------------------------------------------------  2608
71_PHR_APR           ----------------------------------------------------------------------  2687
149_PHR_APR          ----------------------------------------------------------------------  2611
59_Danko_non-APR     ----------------------------------------------------------------------  2639
61_Danko_non-APR     ----------------------------------------------------------------------  2680
123_Danko_non-APR    ----------------------------------------------------------------------  2562
129_Danko_non-APR    ----------------------------------------------------------------------  2609
37_PHR_non-APR       ----------------------------------------------------------------------  2596
52_PHR_non-APR       TAACATAGCTAGTTACCACAAGCACTTCTCTCCTCATTAATTACATGCCACATAAGCAAAACTGTGTTGG  2983
88_PHR_non-APR       ----------------------------------------------------------------------  2590
101_PHR_non-APR      ----------------------------------------------------------------------  2611
105_PHR_non-APR      ----------------------------------------------------------------------  2610
150_PHR_non-APR      ----------------------------------------------------------------------  2552
150_PHR_non-APR2     ----------------------------------------------------------------------  2552
```

Consensus	--	
	3370 3380 3390 3400 3410 3420 3430	
Lr67(sus)	--	2536
Lr67(res)	--	2536
ScLr67_1 (Lo7)	--	2728
118_Danko_APR	--	2613
119_Danko_APR	--	2618
119_Danko_APR2	--	2618
120_Danko_APR	--	2620
138_Danko_APR	--	2616
153_Danko_APR	--	2607
157_Danko_APR	--	2618
160_Danko_APR	--	2608
71_PHR_APR	--	2687
149_PHR_APR	--	2611
59_Danko_non-APR	--	2639
61_Danko_non-APR	--	2680
123_Danko_non-APR	--	2562
129_Danko_non-APR	--	2609
37_PHR_non-APR	--	2596
52_PHR_non-APR	GATGTGTGATGTTACTAGCTATGTTACTCCCACTATGGCCAGCCGTAGAAACATAGATGTGGTGTCATGC	3053
88_PHR_non-APR	--	2590
101_PHR_non-APR	--	2611
105_PHR_non-APR	--	2610
150_PHR_non-APR	--	2552
150_PHR_non-APR2	--	2552

Consensus	--	
	3440 3450 3460 3470 3480 3490 3500	
Lr67(sus)	--	2536
Lr67(res)	--	2536
ScLr67_1 (Lo7)	--	2728
118_Danko_APR	--	2613
119_Danko_APR	--	2618
119_Danko_APR2	--	2618
120_Danko_APR	--	2620
138_Danko_APR	--	2616
153_Danko_APR	--	2607
157_Danko_APR	--	2618
160_Danko_APR	--	2608
71_PHR_APR	--	2687
149_PHR_APR	--	2611
59_Danko_non-APR	--	2639
61_Danko_non-APR	--	2680
123_Danko_non-APR	--	2562
129_Danko_non-APR	--	2609
37_PHR_non-APR	--	2596
52_PHR_non-APR	AAGAGTTCATTTATTAGTCTATAGACTCAATTTGCATTGGTATGTGTGATGTTACAGTAACATAGCTAGT	3123
88_PHR_non-APR	--	2590
101_PHR_non-APR	--	2611
105_PHR_non-APR	--	2610
150_PHR_non-APR	--	2552
150_PHR_non-APR2	--	2552

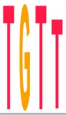

```
Consensus            ------------------------------------------------------------------TGT-
                             3510      3520      3530      3540      3550      3560      3570
Lr67(sus)            ------------------------------------------------------------------TGT-   2539
Lr67(res)            ------------------------------------------------------------------TGT-   2539
ScLr67_1 (Lo7)       ------------------------------------------------------------------TGT-   2731
118_Danko_APR        ------------------------------------------------------------------TGT-   2616
119_Danko_APR        ------------------------------------------------------------------TGTT   2622
119_Danko_APR2       ------------------------------------------------------------------TGTT   2622
120_Danko_APR        ------------------------------------------------------------------TGTT   2624
138_Danko_APR        ------------------------------------------------------------------TGTT   2620
153_Danko_APR        ------------------------------------------------------------------TGT-   2610
157_Danko_APR        ------------------------------------------------------------------TGTT   2622
160_Danko_APR        ------------------------------------------------------------------TGTT   2612
71_PHR_APR           ------------------------------------------------------------------TGTT   2691
149_PHR_APR          ------------------------------------------------------------------TGT-   2614
59_Danko_non-APR     ------------------------------------------------------------------TGTT   2643
61_Danko_non-APR     ------------------------------------------------------------------TGT-   2683
123_Danko_non-APR    ------------------------------------------------------------------TGT-   2565
129_Danko_non-APR    ------------------------------------------------------------------TGT-   2612
37_PHR_non-APR       ------------------------------------------------------------------TGTT   2600
52_PHR_non-APR       TACCACAAACACTTCTTTCCTCATTAATTACATGCCACATAAGCAAAATTGTGTTGGAATGTGTGATGTT   3193
88_PHR_non-APR       ------------------------------------------------------------------TGT-   2593
101_PHR_non-APR      ------------------------------------------------------------------TGT-   2614
105_PHR_non-APR      ------------------------------------------------------------------TGT-   2613
150_PHR_non-APR      ------------------------------------------------------------------TGT-   2555
150_PHR_non-APR2     ------------------------------------------------------------------TGT-   2555
```

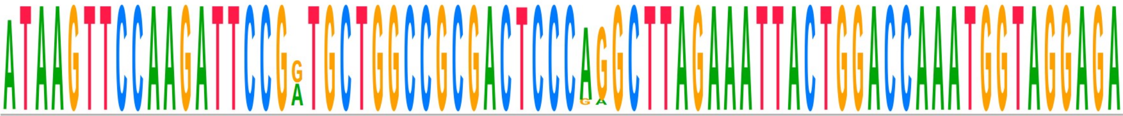

```
Consensus            -TAAGTTCCAAGATTCCGNTGCTGGCCGCGACTCCCAGGCTTAGAAATTACTGGACCAAATGGTAGGAGA
                             3580      3590      3600      3610      3620      3630      3640
Lr67(sus)            -TAAGTTCCAAGATTCCGATGCTGGCCGCGAC-CCCGAGCTTAGAAATTACTGGACCAAATGGTAGGAGA   2607
Lr67(res)            -TAAGTTCCAAGATTCCGATGCTGGCCGCGAC-CCCGAGCTTAGAAATTACTGGACCAAATGGTAGGAGA   2607
ScLr67_1 (Lo7)       -TAAGTTCCAAGATTCCGGTGCTGGCCGCGACTCCCAGGCTTAGAAATTACTGGACCAAATGGTAGGAGA   2800
118_Danko_APR        -TAAGTTCCAAGATTCCGGTGCTGGCCGCGACTCCCAGGCTTAGAAATTACTGGACCAAATGGTAGGAGA   2685
119_Danko_APR        ATAAGTTCCAAGATTCCGATGCTGGCCGCGACTCCCAGGCTTAGAAATTACTGGACCAAATGGTAGGAGA   2692
119_Danko_APR2       ATAAGTTCCAAGATTCCGATGCTGGCCGCGACTCCCAGGCTTAGAAATTACTGGACCAAATGGTAGGAGA   2692
120_Danko_APR        ATAAGTTCCAAGATTCCGATGCTGGCCGCGACTCCCAGGCTTAGAAATTACTGGACCAAATGGTAGGAGA   2694
138_Danko_APR        ATAAGTTCCAAGATTCCGATGCTGGCCGCGACTCCCAGGCTTAGAAATTACTGGACCAAATGGTAGGAGA   2690
153_Danko_APR        -TAAGTTCCAAGATTCCGGTGCTGGCCGCGACTCCCAGGCTTAGAAATTACTGGACCAAATGGTAGGAGA   2679
157_Danko_APR        ATAAGTTCCAAGATTCCGATGCTGGCCGCGACTCCCAGGCTTAGAAATTACTGGACCAAATGGTAGGAGA   2692
160_Danko_APR        ATAAGTTCCAAGATTCCGATGCTGGCCGCGACTCCCAGGCTTAGAAATTACTGGACCAAATGGTAGGAGA   2682
71_PHR_APR           ATAAGTTCCAAGATTCCGATGCTGGCCGCGACTCCCAGGCTTAGAAATTACTGGACCAAATGGTAGGAGA   2761
149_PHR_APR          -TAAGTTCCAAGATTCCGGTGCTGGCCGCGACTCCCAGGCTTAGAAATTACTGGACCAAATGGTAGGAGA   2683
59_Danko_non-APR     ATAAGTTCCAAGATTCCGATGCTGGCCGCGACTCCCAGGCTTAGAAATTACTGGACCAAATGGTAGGAGA   2713
61_Danko_non-APR     -TAAGTTCCAAGATTCCGGTGCTGGCCGCGACTCCCAGGCTTAGAAATTACTGGACCAAATGGTAGGAGA   2752
123_Danko_non-APR    -TAAGTTCCAAGATTCCGGTGCTGGCCGCGACTCCCAGGCTTAGAAATTACTGGACCAAATGGTAGGAGA   2634
129_Danko_non-APR    -TAAGTTCCAAGATTCCGGTGCTGGCCGCGACTCCCAGGCTTAGAAATTACTGGACCAAATGGTAGGAGA   2681
37_PHR_non-APR       ATAAGTTCCAAGATTCCGATGCTGGCCGCGACTCCCAGGCTTAGAAATTACTGGACCAAATGGTAGGAGA   2670
52_PHR_non-APR       ATAAGTTCCAAGATTCCGATGCTGGCCGCGACTCCCAGGCTTAGAAATTACTGGACCAAATGGTAGGAGA   3263
88_PHR_non-APR       -TAAGTTCCAAGATTCCGGTGCTGGCCGCGACTCCCAGGCTTAGAAATTACTGGACCAAATGGTAGGAGA   2662
101_PHR_non-APR      -TAAGTTCCAAGATTCCGGTGCTGGCCGCGACTCCCAGGCTTAGAAATTACTGGACCAAATGGTAGGAGA   2683
105_PHR_non-APR      -TAAGTTCCAAGATTCCGGTGCTGGCCGCGACTCCCAGGCTTAGAAATTACTGGACCAAATGGTAGGAGA   2682
150_PHR_non-APR      -TAAGTTCCAAGATTCCGGTGCTGGCCGCGACTCCCAGGCTTAGAAATTACTGGACCAAATGGTAGGAGA   2624
150_PHR_non-APR2     -TAAGTTCCAAGATTCCGGTGCTGGCCGCGACTCCCAGGCTTAGAAATTACTGGACCAAATGGTAGGAGA   2624
```

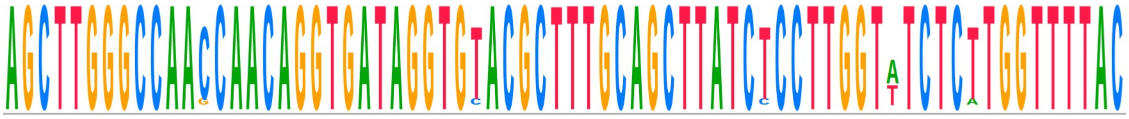

```
Consensus            AGCTTGGGCCAACCAACAGGTGATAGGTGTACGCTTTGCAGCTTATCTCCTTGGTATCTCTTGGTTTTAC
                              3650      3660      3670      3680      3690      3700      3710
Lr67(sus)            AGCTTGGGCCAAGCAACAGGTGATAGGTGCACGC-TTGCAGCTTATCCCCTTGGTTTCTCATGGTTTTAC  2676
Lr67(res)            AGCTTGGGCCAAGCAACAGGTGATAGGTGCACGC-TTGCAGCTTATCCCCTTGGTTTCTCATGGTTTTAC  2676
ScLr67_1 (Lo7)       AGCTTGGGCCAACCAACAGGTGATAGGTGTACGCTTTGCAGCTTATCTCCTTGGTATCTCTTGGTTTTAC  2870
118_Danko_APR        AGCTTGGGCCAACCAACAGGTGATAGGTGTACGCTTTGCAGCTTATCTCCTTGGTATCTCTTGGTTTTAC  2755
119_Danko_APR        AGCTTGGGCCAACCAACAGGTGATAGGTGTACGCTTTGCAGCTTATCTCCTTGGTTTCTCTTGGTTTTAC  2762
119_Danko_APR2       AGCTTGGGCCAACCAACAGGTGATAGGTGTACGCTTTGCAGCTTATCTCCTTGGTTTCTCTTGGTTTTAC  2762
120_Danko_APR        AGCTTGGGCCAACCAACAGGTGATAGGTGTACGCTTTGCAGCTTATCTCCTTGGTTTCTCTTGGTTTTAC  2764
138_Danko_APR        AGCTTGGGCCAACCAACAGGTGATAGGTGTACGCTTTGCAGCTTATCTCCTTGGTTTCTCTTGGTTTTAC  2760
153_Danko_APR        AGCTTGGGCCAACCAACAGGTGATAGGTGTACGCTTTGCAGCTTATCTCCTTGGTATCTCTTGGTTTTAC  2749
157_Danko_APR        AGCTTGGGCCAACCAACAGGTGATAGGTGTACGCTTTGCAGCTTATCTCCTTGGTTTCTCTTGGTTTTAC  2762
160_Danko_APR        AGCTTGGGCCAACCAACAGGTGATAGGTGTACGCTTTGCAGCTTATCTCCTTGGTTTCTCTTGGTTTTAC  2752
71_PHR_APR           AGCTTGGGCCAACCAACAGGTGATAGGTGTACGCTTTGCAGCTTATCTCCTTGGTTTCTCTTGGTTTTAC  2831
149_PHR_APR          AGCTTGGGCCAACCAACAGGTGATAGGTGTACGCTTTGCAGCTTATCTCCTTGGTATCTCTTGGTTTTAC  2753
59_Danko_non-APR     AGCTTGGGCCAACCAACAGGTGATAGGTGTACGCTTTGCAGCTTATCTCCTTGGTTTCTCTTGGTTTTAC  2783
61_Danko_non-APR     AGCTTGGGCCAACCAACAGGTGATAGGTGTACGCTTTGCAGCTTATCTCCTTGGTATCTCTTGGTTTTAC  2822
123_Danko_non-APR    AGCTTGGGCCAACCAACAGGTGATAGGTGTACGCTTTGCAGCTTATCTCCTTGGTATCTCTTGGTTTTAC  2704
129_Danko_non-APR    AGCTTGGGCCAACCAACAGGTGATAGGTGTACGCTTTGCAGCTTATCTCCTTGGTATCTCTTGGTTTTAC  2751
37_PHR_non-APR       AGCTTGGGCCAACCAACAGGTGATAGGTGTACGCTTTGCAGCTTATCTCCTTGGTTTCTCTTGGTTTTAC  2740
52_PHR_non-APR       AGCTTGGGCCAACCAACAGGTGATAGGTGTACGCTTTGCAGCTTATCTCCTTGGTATCTCTTGGTTTTAC  3333
88_PHR_non-APR       AGCTTGGGCCAACCAACAGGTGATAGGTGTACGCTTTGCAGCTTATCTCCTTGGTATCTCTTGGTTTTAC  2732
101_PHR_non-APR      AGCTTGGGCCAACCAACAGGTGATAGGTGTACGCTTTGCAGCTTATCTCCTTGGTATCTCTTGGTTTTAC  2753
105_PHR_non-APR      AGCTTGGGCCAACCAACAGGTGATAGGTGTACGCTTTGCAGCTTATCTCCTTGGTATCTCTTGGTTTTAC  2752
150_PHR_non-APR      AGCTTGGGCCAACCAACAGGTGATAGGTGTACGCTTTGCAGCTTATCTCCTTGGTATCTCTTGGTTTTAC  2694
150_PHR_non-APR2     AGCTTGGGCCAACCAACAGGTGATAGGTGTACGCTTTGCAGCTTATCTCCTTGGTATCTCTTGGTTTTAC  2694
```

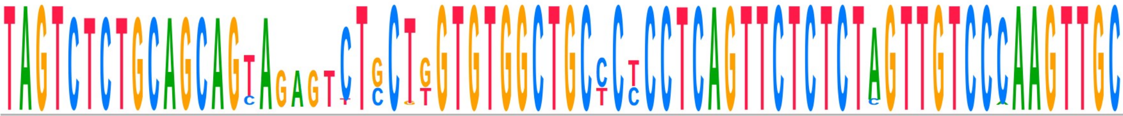

```
Consensus            TAGT-----------------CTGCTGGTGTGGCTGCCCTCCTCAGTTCTCTCTAGTTGTCCCAAGTTGC
                              3720      3730      3740      3750      3760      3770      3780
Lr67(sus)            TAGTCTCTGCAGCAGCAGAGTTTGCTGGTGTGGCTGCCC-------TTCTCTCTCGTTGTCCCAAGTTGC  2739
Lr67(res)            TAGTCTCTGCAGCAGCAGAGTTTGCTGGTGTGGCTGCCC-------TTCTCTCTCGTTGTCCCAAGTTGC  2739
ScLr67_1 (Lo7)       TAGT-----------------CTGCTGGTGTGGCTGCCCTCCTCAGTTCTCTCTAGTTGTCCCAAGTTGC  2923
118_Danko_APR        TAGT-----------------CTGCTGGTGTGGCTGCCCTCCTCAGTTCTCTCTAGTTGTCCCAAGTTGC  2808
119_Danko_APR        TAGTCTCTGCAGCAGTA----CTCCTTGTGTGGCTGCTCCCCTCAGTTCTCTCTAGTTGTCCCAAGTTGC  2828
119_Danko_APR2       TAGTCTCTGCAGCAGTA----CTCCTTGTGTGGCTGCTCCCCTCAGTTCTCTCTAGTTGTCCCAAGTTGC  2828
120_Danko_APR        TAGTCTCTGCAGCAGTA----CTCCTTGTGTGGCTGCTCCCCTCAGTTCTCTCTAGTTGTCCCAAGTTGC  2830
138_Danko_APR        TAGTCTCTGCAGCAGTA----CTCCTTGTGTGGCTGCTCCCCTCAGTTCTCTCTAGTTGTCCCAAGTTGC  2826
153_Danko_APR        TAGT-----------------CTGCTGGTGTGGCTGCCCTCCTCAGTTCTCTCTAGTTGTCCCAAGTTGC  2802
157_Danko_APR        TAGTCTCTGCAGCAGTA----CTCCTTGTGTGGCTGCTCCCCTCAGTTCTCTCTAGTTGTCCCAAGTTGC  2828
160_Danko_APR        TAGTCTCTGCAGCAGTA----CTCCTTGTGTGGCTGCTCCCCTCAGTTCTCTCTAGTTGTCCCAAGTTGC  2818
71_PHR_APR           TAGTCTCTGCAGCAGTA----CTCCGTGTGTGGCTGCTCCCCTCAGTTCTCTCTAGTTGTCCCAAGTTGC  2897
149_PHR_APR          TAGT-----------------CTGCTGGTGTGGCTGCCCTCCTCAGTTCTCTCTAGTTGTCCCAAGTTGC  2806
59_Danko_non-APR     TAGTCTCTGCAGCAGTA----CTCCTTGTGTGGCTGCTCCCCTCAGTTCTCTCTAGTTGTCCCAAGTTGC  2849
61_Danko_non-APR     TAGT-----------------CTGCTGGTGTGGCTGCCCTCCTCAGTTCTCTCTAGTTGTCCCAAGTTGC  2875
123_Danko_non-APR    TAGT-----------------CTGCTGGTGTGGCTGCCCTCCTCAGTTCTCTCTAGTTGTCCCAAGTTGC  2757
129_Danko_non-APR    TAGT-----------------CTGCTGGTGTGGCTGCCCTCCTCAGTTCTCTCTAGTTGTCCCAAGTTGC  2804
37_PHR_non-APR       TAGTCTCTGCAGCAGTA----CTCCTTGTGTGGCTGCTCCCCTCAGTTCTCTCTAGTTGTCCCAAGTTGC  2806
52_PHR_non-APR       TAGT-----------------CTGCTGGTGTGGCTGCCCTCCTCAGTTCTCTCTAGTTGTCCAAAGTTGC  3386
88_PHR_non-APR       TAGT-----------------CTGCTGGTGTGGCTGCCCTCCTCAGTTCTCTCTAGTTGTCCCAAGTTGC  2785
101_PHR_non-APR      TAGT-----------------CTGCTGGTGTGGCTGCCCTCCTCAGTTCTCTCTAGTTGTCCCAAGTTGC  2806
105_PHR_non-APR      TAGT-----------------CTGCTGGTGTGGCTGCCCTCCTCAGTTCTCTCTAGTTGTCCCAAGTTGC  2805
150_PHR_non-APR      TAGT-----------------CTGCTGGTGTGGCTGCCCTCCTCAGTTCTCTCTAGTTGTCCCAAGTTGC  2747
150_PHR_non-APR2     TAGT-----------------CTGCTGGTGTGGCTGCCCTCCTCAGTTCTCTCTAGTTGTCCCAAGTTGC  2747
```

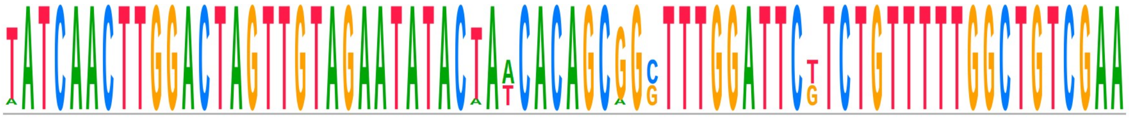

```
Consensus            TATCAACTTGGACTAGTTGTAGAATATACTAACACAGCGGCTTTGGATTCTTCTGTTTTTGGCTGTCGAA
                          3790      3800      3810      3820      3830      3840      3850

Lr67(sus)            AATCAACTTGGACTAGTTGTAGAATATACAAACACAGCAGCTTTGGATTCTTCTGTTTTTGGCTGTCGAA  2809
Lr67(res)            AATCAACTTGGACTAGTTGTAGAATATACAAACACAGCAGCTTTGGATTCTTCTGTTTTTGGCTGTCGAA  2809
ScLr67_1 (Lo7)       TATCAACTTGGACTAGTTGTAGAATATACTATCACAGCGGCTTTGGATTCTTCTGTTTTTGGCTGTCGAA  2993
118_Danko_APR        TATCAACTTGGACTAGTTGTAGAATATACTAACACAGCGGGTTTGGATTCGTCTGTTTTTGGCTGTCGAA  2878
119_Danko_APR        TATCAACTTGGACTAGTTGTAGAATATACTATCACAGCGGCTTTGGATTCTTCTGTTTTTGGCTGTCGAA  2898
119_Danko_APR2       TATCAACTTGGACTAGTTGTAGAATATACTATCACAGCGGCTTTGGATTCTTCTGTTTTTGGCTGTCGAA  2898
120_Danko_APR        TATCAACTTGGACTAGTTGTAGAATATACTATCACAGCGGCTTTGGATTCTTCTGTTTTTGGCTGTCGAA  2900
138_Danko_APR        TATCAACTTGGACTAGTTGTAGAATATACTATCACAGCGGCTTTGGATTCTTCTGTTTTTGGCTGTCGAA  2896
153_Danko_APR        TATCAACTTGGACTAGTTGTAGAATATACTAACACAGCGGGTTTGGATTCGTCTGTTTTTGGCTGTCGAA  2872
157_Danko_APR        TATCAACTTGGACTAGTTGTAGAATATACTATCACAGCGGCTTTGGATTCTTCTGTTTTTGGCTGTCGAA  2898
160_Danko_APR        TATCAACTTGGACTAGTTGTAGAATATACTATCACAGCGGCTTTGGATTCTTCTGTTTTTGGCTGTCGAA  2888
71_PHR_APR           TATCAACTTGGACTAGTTGTAGAATATACTATCACAGCGGCTTTGGATTCTTCTGTTTTTGGCTGTCGAA  2967
149_PHR_APR          TATCAACTTGGACTAGTTGTAGAATATACTAACACAGCGGGTTTGGATTCGTCTGTTTTTGGCTGTCGAA  2876
59_Danko_non-APR     TATCAACTTGGACTAGTTGTAGAATATACTATCACAGCGGCTTTGGATTCTTCTGTTTTTGGCTGTCGAA  2919
61_Danko_non-APR     TATCAACTTGGACTAGTTGTAGAATATACTATCACAGCGGCTTTGGATTCTTCTGTTTTTGGCTGTCGAA  2945
123_Danko_non-APR    TATCAACTTGGACTAGTTGTAGAATATACTAACACAGCGGGTTTGGATTCGTCTGTTTTTGGCTGTCGAA  2827
129_Danko_non-APR    TATCAACTTGGACTAGTTGTAGAATATACTAACACAGCGGGTTTGGATTCGTCTGTTTTTGGCTGTCGAA  2874
37_PHR_non-APR       TATCAACTTGGACTAGTTGTAGAATATACTATCACAGCGGCTTTGGATTCTTCTGTTTTTGGCTGTCGAA  2876
52_PHR_non-APR       TATCAACTTGGACTAGTTGTAGAATATACTAACACAGCGGGTTTGGATTCGTCTGTTTTTGGCTGTCGAA  3456
88_PHR_non-APR       TATCAACTTGGACTAGTTGTAGAATATACTAACACAGCGGGTTTGGATTCGTCTGTTTTTGGCTGTCGAA  2855
101_PHR_non-APR      TATCAACTTGGACTAGTTGTAGAATATACTAACACAGCGGGTTTGGATTCGTCTGTTTTTGGCTGTCGAA  2876
105_PHR_non-APR      TATCAACTTGGACTAGTTGTAGAATATACTAACACAGCGGGTTTGGATTCGTCTGTTTTTGGCTGTCGAA  2875
150_PHR_non-APR      TATCAACTTGGACTAGTTGTAGAATATACTAACACAGCGGGTTTGGATTCGTCTGTTTTTGGCTGTCGAA  2817
150_PHR_non-APR2     TATCAACTTGGACTAGTTGTAGAATATACTAACACAGCGGGTTTGGATTCGTCTGTTTTTGGCTGTCGAA  2817
```

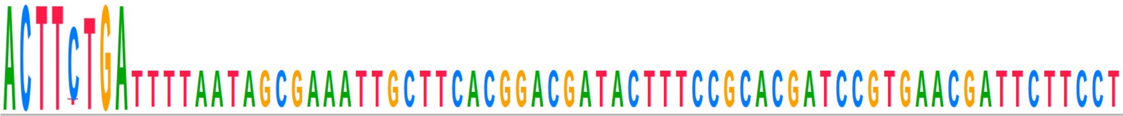

```
Consensus            ACTTC-GA--------------------------------------------------------------
                              3860      3870      3880      3890      3900      3910      3920

Lr67(sus)            ACTTT-GATTTTAATAGCGAAATTGCTTCACGGACGATACTTTCCGCACGATCCGTGAACGATTCTTCCT  2878
Lr67(res)            ACTTT-GATTTTAATAGCGAAATTGCTTCACGGACGATACTTTCCGCACGATCCGTGAACGATTCTTCCT  2878
ScLr67_1 (Lo7)       ACTTC-GA--------------------------------------------------------------  3000
118_Danko_APR        ACTTCTGA--------------------------------------------------------------  2886
119_Danko_APR        ACTTC-GA--------------------------------------------------------------  2905
119_Danko_APR2       ACTTC-GA--------------------------------------------------------------  2905
120_Danko_APR        ACTTC-GA--------------------------------------------------------------  2907
138_Danko_APR        ACTTC-GA--------------------------------------------------------------  2903
153_Danko_APR        ACTTCTGA--------------------------------------------------------------  2880
157_Danko_APR        ACTTC-GA--------------------------------------------------------------  2905
160_Danko_APR        ACTTC-GA--------------------------------------------------------------  2895
71_PHR_APR           ACTTC-GA--------------------------------------------------------------  2974
149_PHR_APR          ACTTCTGA--------------------------------------------------------------  2884
59_Danko_non-APR     ACTTC-GA--------------------------------------------------------------  2926
61_Danko_non-APR     ACTTC-GA--------------------------------------------------------------  2952
123_Danko_non-APR    ACTTCTGA--------------------------------------------------------------  2835
129_Danko_non-APR    ACTTCTGA--------------------------------------------------------------  2882
37_PHR_non-APR       ACTTC-GA--------------------------------------------------------------  2883
52_PHR_non-APR       ACTTCTGA--------------------------------------------------------------  3464
88_PHR_non-APR       ACTTCTGA--------------------------------------------------------------  2863
101_PHR_non-APR      ACTTCTGA--------------------------------------------------------------  2884
105_PHR_non-APR      ACTTCTGA--------------------------------------------------------------  2883
150_PHR_non-APR      ACTTCTGA--------------------------------------------------------------  2825
150_PHR_non-APR2     ACTTCTGA--------------------------------------------------------------  2825
```

```
                     TCGCGTCCAAATCTCATGCTTGCACGCACGCTGTGGCGCTGTCATGTAGGATCGTTCAGGCCTCGACGCA
Consensus            ----------------------------------------------------------------------
                              3930      3940      3950      3960      3970      3980      3990
Lr67(sus)            TCGCGTCCAAATCTCATGCTTGCACGCACGCTGTGGCGCTGTCATGTAGGATCGTTCAGGCCTCGACGCA 2948
Lr67(res)            TCGCGTCCAAATCTCATGCTTGCACGCACGCTGTGGCGCTGTCATGTAGGATCGTTCAGGCCTCGACGCA 2948
ScLr67_1 (Lo7)       ---------------------------------------------------------------------- 3000
118_Danko_APR        ---------------------------------------------------------------------- 2886
119_Danko_APR        ---------------------------------------------------------------------- 2905
119_Danko_APR2       ---------------------------------------------------------------------- 2905
120_Danko_APR        ---------------------------------------------------------------------- 2907
138_Danko_APR        ---------------------------------------------------------------------- 2903
153_Danko_APR        ---------------------------------------------------------------------- 2880
157_Danko_APR        ---------------------------------------------------------------------- 2905
160_Danko_APR        ---------------------------------------------------------------------- 2895
71_PHR_APR           ---------------------------------------------------------------------- 2974
149_PHR_APR          ---------------------------------------------------------------------- 2884
59_Danko_non-APR     ---------------------------------------------------------------------- 2926
61_Danko_non-APR     ---------------------------------------------------------------------- 2952
123_Danko_non-APR    ---------------------------------------------------------------------- 2835
129_Danko_non-APR    ---------------------------------------------------------------------- 2882
37_PHR_non-APR       ---------------------------------------------------------------------- 2883
52_PHR_non-APR       ---------------------------------------------------------------------- 3464
88_PHR_non-APR       ---------------------------------------------------------------------- 2863
101_PHR_non-APR      ---------------------------------------------------------------------- 2884
105_PHR_non-APR      ---------------------------------------------------------------------- 2883
150_PHR_non-APR      ---------------------------------------------------------------------- 2825
150_PHR_non-APR2     ---------------------------------------------------------------------- 2825

                     AATCTATCGTGTGTGTAGCAACACTTTTTTAGTAGTCGCAAGCAGGCGCGGTTGTTCACGCGTAGGCCAC
Consensus            --------------------------TTTTAGTAGTCGCAAGCAGGCGCGGTTGTTCACGCGTAGGCCAC
                              4000      4010      4020      4030      4040      4050      4060
Lr67(sus)            AATCTATCGTGTGTGTAGCAACACTTTTTTAGTAGTAGCAAGCAGGCGCGGTTGTTCACGTGTAGGCCAC 3018
Lr67(res)            AATCTATCGTGTGTGTAGCAACACTTTTTTAGTAGTAGCAAGCAGGCGCGGTTGTTCACGTGTAGGCCAC 3018
ScLr67_1 (Lo7)       --------------------------TTTTAGTAGTCGCAAGCAGGCGCGGTTGTTCACGCGTAGGCCAC 3044
118_Danko_APR        --------------------------TTTTAGTAGTCGCAAGCAGGCGCGGTTGTTCACGCGTAGGCCAC 2930
119_Danko_APR        --------------------------TTTTAGTAGTCGCAAGCAGGCGCGGTTGTTCACGCGTAGGCCAC 2949
119_Danko_APR2       --------------------------TTTTAGTAGTCGCAAGCAGGCGCGGTTGTTCACGCGTAGGCCAC 2949
120_Danko_APR        --------------------------TTTTAGTAGTCGCAAGCAGGCGCGGTTGTTCACGCGTAGGCCAC 2951
138_Danko_APR        --------------------------TTTTAGTAGTCGCAAGCAGGCGCGGTTGTTCACGCGTAGGCCAC 2947
153_Danko_APR        --------------------------TTTTAGTAGTCGCAAGCAGGCGCGGTTGTTCACGCGTAGGCCAC 2924
157_Danko_APR        --------------------------TTTTAGTAGTCGCAAGCAGGCGCGGTTGTTCACGCGTAGGCCAC 2949
160_Danko_APR        --------------------------TTTTAGTAGTCGCAAGCAGGCGCGGTTGTTCACGCGTAGGCCAC 2939
71_PHR_APR           --------------------------TTTTAGTAGTCGCAAGCAGGCGCGGTTGTTCACGCGTAGGCCAC 3018
149_PHR_APR          --------------------------TTTTAGTAGTCGCAAGCAGGCGCGGTTGTTCACGCGTAGGCCAC 2928
59_Danko_non-APR     --------------------------TTTTAGTAGTCGCAAGCAGGCGCGGTTGTTCACGCGTAGGCCAC 2970
61_Danko_non-APR     --------------------------TTTTAGTAGTCGCAAGCAGGCGCGGTTGTTCACGCGTAGGCCAC 2996
123_Danko_non-APR    --------------------------TTTTAGTAGTCGCAAGCAGGCGCGGTTGTTCACGCGTAGGCCAC 2879
129_Danko_non-APR    --------------------------TTTTAGTAGTCGCAAGCAGGCGCGGTTGTTCACGCGTAGGCCAC 2926
37_PHR_non-APR       --------------------------TTTTAGTAGTCGCAAGCAGGCGCGGTTGTTCACGCGTAGGCCAC 2927
52_PHR_non-APR       --------------------------TTTTAGTAGTCGCAAGCAGGCGCGGTTGTTCACGCGTAGGCCAC 3508
88_PHR_non-APR       --------------------------TTTTAGTAGTCGCAAGCAGGCGCGGTTGTTCACGCGTAGGCCAC 2907
101_PHR_non-APR      --------------------------TTTTAGTAGTCGCAAGCAGGCGCGGTTGTTCACGCGTAGGCCAC 2928
105_PHR_non-APR      --------------------------TTTTAGTAGTCGCAAGCAGGCGCGGTTGTTCACGCGTAGGCCAC 2927
150_PHR_non-APR      --------------------------TTTTAGTAGTCGCAAGCAGGCGCGGTTGTTCACGCGTAGGCCAC 2869
150_PHR_non-APR2     --------------------------TTTTAGTAGTCGCAAGCAGGCGCGGTTGTTCACGCGTAGGCCAC 2869
```

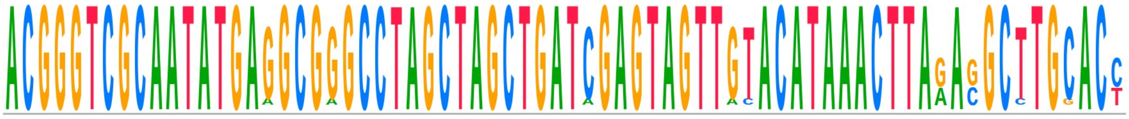

```
Consensus              ACGGGTCGCAATATGAGGCGGGCCTAGCTAGCTGATCGAGTAGTTGTACATAAACTTAGAGGCTTGCACC
                                4070      4080      4090      4100      4110      4120      4130
Lr67(sus)              ACGGGTCGCAATATGAAGCGAGC----CTAGCTGATAGAGTAGTTATACATAAACTTAG--GCCTGGACC  3082
Lr67(res)              ACGGGTCGCAATATGAAGCGAGC----CTAGCTGATAGAGTAGTTATACATAAACTTAG--GCCTGGACC  3082
ScLr67_1 (Lo7)         ACGGGTCGCAATATGAGGCGGGCCTAGCTAGCTGATCGAGTAGTTGCACATAAACTTAGAGGCTTGCACC  3114
118_Danko_APR          ACGGGTCGCAATATGAGGCGGGCCTAGCTAGCTGATCGAGTAGTTGTACATAAACTTAGAGGCTTGCACC  3000
119_Danko_APR          ACGGGTCGCAATATGAGGCGGGCCTAGCTAGCTGATCGAGTAGTTGTACATAAACTTAAACGCTTGCACT  3019
119_Danko_APR2         ACGGGTCGCAATATGAGGCGGGCCTAGCTAGCTGATCGAGTAGTTGTACATAAACTTAAACGCTTGCACT  3019
120_Danko_APR          ACGGGTCGCAATATGAGGCGGGCCTAGCTAGCTGATCGAGTAGTTGTACATAAACTTAAACGCTTGCACT  3021
138_Danko_APR          ACGGGTCGCAATATGAGGCGGGCCTAGCTAGCTGATCGAGTAGTTGTACATAAACTTAAACGCTTGCACT  3017
153_Danko_APR          ACGGGTCGCAATATGAGGCGGGCCTAGCTAGCTGATCGAGTAGTTGTACATAAACTTAGAGGCTTGCACC  2994
157_Danko_APR          ACGGGTCGCAATATGAGGCGGGCCTAGCTAGCTGATCGAGTAGTTGTACATAAACTTAAACGCTTGCACT  3019
160_Danko_APR          ACGGGTCGCAATATGAGGCGGGCCTAGCTAGCTGATCGAGTAGTTGTACATAAACTTAAACGCTTGCACT  3009
71_PHR_APR             ACGGGTCGCAATATGAGGCGGGCCTAGCTAGCTGATCGAGTAGTTGTACATAAACTTAAACGCTTGCACT  3088
149_PHR_APR            ACGGGTCGCAATATGAGGCGGGCCTAGCTAGCTGATCGAGTAGTTGTACATAAACTTAGAGGCTTGCACC  2998
59_Danko_non-APR       ACGGGTCGCAATATGAGGCGGGCCTAGCTAGCTGATCGAGTAGTTGTACATAAACTTAAACGCTTGCACT  3040
61_Danko_non-APR       ACGGGTCGCAATATGAGGCGGGCCTAGCTAGCTGATCGAGTAGTTGCACATAAACTTAGAGGCTTGCACC  3066
123_Danko_non-APR      ACGGGTCGCAATATGAGGCGGGCCTAGCTAGCTGATCGAGTAGTTGTACATAAACTTAGAGGCTTGCACC  2949
129_Danko_non-APR      ACGGGTCGCAATATGAGGCGGGCCTAGCTAGCTGATCGAGTAGTTGTACATAAACTTAGAGGCTTGCACC  2996
37_PHR_non-APR         ACGGGTCGCAATATGAGGCGGGCCTAGCTAGCTGATCGAGTAGTTGTACATAAACTTAAACGCTTGCACT  2997
52_PHR_non-APR         ACGGGTCGCAATATGAGGCGGGCCTAGCTAGCTGATCGAGTAGTTGTACATAAACTTAGAGGCTTGCACC  3578
88_PHR_non-APR         ACGGGTCGCAATATGAGGCGGGCCTAGCTAGCTGATCGAGTAGTTGTACATAAACTTAGAGGCTTGCACC  2977
101_PHR_non-APR        ACGGGTCGCAATATGAGGCGGGCCTAGCTAGCTGATCGAGTAGTTGTACATAAACTTAGAGGCTTGCACC  2998
105_PHR_non-APR        ACGGGTCGCAATATGAGGCGGGCCTAGCTAGCTGATCGAGTAGTTGTACATAAACTTAGAGGCTTGCACC  2997
150_PHR_non-APR        ACGGGTCGCAATATGAGGCGGGCCTAGCTAGCTGATCGAGTAGTTGTACATAAACTTAGAGGCTTGCACC  2939
150_PHR_non-APR2       ACGGGTCGCAATATGAGGCGGGCCTAGCTAGCTGATCGAGTAGTTGTACATAAACTTAGAGGCTTGCACC  2939
```

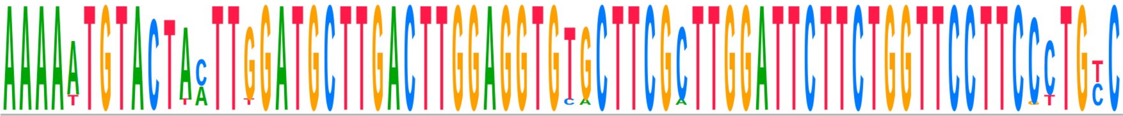

```
Consensus              AAAAATGTA--ACTTGGATGCTTGACTTGGAGGTGTGCTTCGCTTGGATTCTTCTGGTTCCTTCCCTGNC
                                4140      4150      4160      4170      4180      4190      4200
Lr67(sus)              AAAATTGTA--TCTTTGATGCTTGACTTGGAGGTGCACTTCGCTTGGATTCTTCTGGTTCCTTCCTTGCC  3150
Lr67(res)              AAAATTGTA--TCTTTGATGCTTGACTTGGAGGTGCACTTCGCTTGGATTCTTCTGGTTCCTTCCTTGCC  3150
ScLr67_1 (Lo7)         AAAATTGTA--ACTTGGATGCTTGACTTGGAGGTGTGCTTCGATTGGATTCTTCTGGTTCCTTCCTTGTC  3182
118_Danko_APR          AAAAATGTACTAATTGGATGCTTGACTTGGAGGTGTGCTTCGCTTGGATTCTTCTGGTTCCTTCCCTGCC  3070
119_Danko_APR          AAAAATGTA--ACTTGGATGCTTGACTTGGAGGTGTGCTTCGCTTGGATTCTTCTGGTTCCTTCCCTGTC  3087
119_Danko_APR2         AAAAATGTA--ACTTGGATGCTTGACTTGGAGGTGTGCTTCGCTTGGATTCTTCTGGTTCCTTCCCTGTC  3087
120_Danko_APR          AAAAATGTA--ACTTGGATGCTTGACTTGGAGGTGTGCTTCGCTTGGATTCTTCTGGTTCCTTCCCTGTC  3089
138_Danko_APR          AAAAATGTA--ACTTGGATGCTTGACTTGGAGGTGTGCTTCGCTTGGATTCTTCTGGTTCCTTCCCTGTC  3085
153_Danko_APR          AAAAATGTACTAATTGGATGCTTGACTTGGAGGTGTGCTTCGCTTGGATTCTTCTGGTTCCTTCCCTGCC  3064
157_Danko_APR          AAAAATGTA--ACTTGGATGCTTGACTTGGAGGTGTGCTTCGCTTGGATTCTTCTGGTTCCTTCCCTGTC  3087
160_Danko_APR          AAAAATGTA--ACTTGGATGCTTGACTTGGAGGTGTGCTTCGCTTGGATTCTTCTGGTTCCTTCCCTGTC  3077
71_PHR_APR             AAAAATGTA--ACTTGGATGCTTGACTTGGAGGTGTGCTTCGCTTGGATTCTTCTGGTTCCTTCCCTGTC  3156
149_PHR_APR            AAAAATGTACTAATTGGATGCTTGACTTGGAGGTGTGCTTCGCTTGGATTCTTCTGGTTCCTTCCCTGCC  3068
59_Danko_non-APR       AAAAATGTA--ACTTGGATGCTTGACTTGGAGGTGTGCTTCGCTTGGATTCTTCTGGTTCCTTCCCTGTC  3108
61_Danko_non-APR       AAAATTGTA--ACTTGGATGCTTGACTTGGAGGTGTGCTTCGATTGGATTCTTCTGGTTCCTTCCTTGTC  3134
123_Danko_non-APR      AAAAATGTACTAATTGGATGCTTGACTTGGAGGTGTGCTTCGCTTGGATTCTTCTGGTTCCTTCCCTGCC  3019
129_Danko_non-APR      AAAAATGTACTAATTGGATGCTTGACTTGGAGGTGTGCTTCGCTTGGATTCTTCTGGTTCCTTCCCTGCC  3066
37_PHR_non-APR         AAAAATGTA--ACTTGGATGCTTGACTTGGAGGTGTGCTTCGCTTGGATTCTTCTGGTTCCTTCCCTGTC  3065
52_PHR_non-APR         AAAAATGTA--ACTTGGATGCTTGACTTGGAGGTGTGCTTCGCTTGGATTCTTCTGGTTCCTTCGCTGTC  3646
88_PHR_non-APR         AAAAATGTACTAATTGGATGCTTGACTTGGAGGTGTGCTTCGCTTGGATTCTTCTGGTTCCTTCCCTGCC  3047
101_PHR_non-APR        AAAAATGTACTAATTGGATGCTTGACTTGGAGGTGTGCTTCGCTTGGATTCTTCTGGTTCCTTCCCTGCC  3068
105_PHR_non-APR        AAAAATGTACTAATTGGATGCTTGACTTGGAGGTGTGCTTCGCTTGGATTCTTCTGGTTCCTTCCCTGCC  3067
150_PHR_non-APR        AAAAATGTACTAATTGGATGCTTGACTTGGAGGTGTGCTTCGCTTGGATTCTTCTGGTTCCTTCCCTGCC  3009
150_PHR_non-APR2       AAAAATGTACTAATTGGATGCTTGACTTGGAGGTGTGCTTCGCTTGGATTCTTCTGGTTCCTTCCCTGCC  3009
```

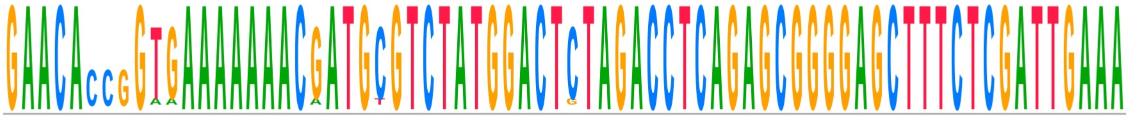

**Consensus**	GAACA---GTGAAAAAAACGATGCGTCTATGGACTCTAGACCTCAGAGCGGGGAGCTTTCTCGATTGAAA	
	4210 4220 4230 4240 4250 4260 4270	
Lr67(sus)	GAACACCGGAAAAAAAAACAATGTGTCTATGGACTGTAGACCTCAGAGCGGGGAGCTTTCTCGATTGAAA	3220
Lr67(res)	GAACACCGGAAAAAAAAACAATGTGTCTATGGACTGTAGACCTCAGAGCGGGGAGCTTTCTCGATTGAAA	3220
ScLr67_1 (Lo7)	GAACA---GTGAAAAAAACGATGCGTCTATGGACTCTAGACCTCAGAGCGGGGAGCTTTCTCGATTGAAA	3249
118_Danko_APR	GAACA---GTGAAAAAAACGATGCGTCTATGGACTCTAGACCTCAGAGCGGGGAGCTTTCTCGATTGAAA	3137
119_Danko_APR	GAACA---GTGAAAAAAACGATGCGTCTATGGACTCTAGACCTCAGAGCGGGGAGCTTTCTCGATTGAAA	3154
119_Danko_APR2	GAACA---GTGAAAAAAACGATGCGTCTATGGACTCTAGACCTCAGAGCGGGGAGCTTTCTCGATTGAAA	3154
120_Danko_APR	GAACA---GTGAAAAAAACGATGCGTCTATGGACTCTAGACCTCAGAGCGGGGAGCTTTCTCGATTGAAA	3156
138_Danko_APR	GAACA---GTGAAAAAAACGATGCGTCTATGGACTCTAGACCTCAGAGCGGGGAGCTTTCTCGATTGAAA	3152
153_Danko_APR	GAACA---GTGAAAAAAACGATGCGTCTATGGACTCTAGACCTCAGAGCGGGGAGCTTTCTCGATTGAAA	3131
157_Danko_APR	GAACA---GTGAAAAAAACGATGCGTCTATGGACTCTAGACCTCAGAGCGGGGAGCTTTCTCGATTGAAA	3154
160_Danko_APR	GAACA---GTGAAAAAAACGATGCGTCTATGGACTCTAGACCTCAGAGCGGGGAGCTTTCTCGATTGAAA	3144
71_PHR_APR	GAACA---GTGAAAAAAACGATGCGTCTATGGACTCTAGACCTCAGAGCGGGGAGCTTTCTCGATTGAAA	3223
149_PHR_APR	GAACA---GTGAAAAAAACGATGCGTCTATGGACTCTAGACCTCAGAGCGGGGAGCTTTCTCGATTGAAA	3135
59_Danko_non-APR	GAACA---GTGAAAAAAACGATGCGTCTATGGACTCTAGACCTCAGAGCGGGGAGCTTTCTCGATTGAAA	3175
61_Danko_non-APR	GAACA---GTGAAAAAAACGATGCGTCTATGGACTCTAGACCTCAGAGCGGGGAGCTTTCTCGATTGAAA	3201
123_Danko_non-APR	GAACA---GTGAAAAAAACGATGCGTCTATGGACTCTAGACCTCAGAGCGGGGAGCTTTCTCGATTGAAA	3086
129_Danko_non-APR	GAACA---GTGAAAAAAACGATGCGTCTATGGACTCTAGACCTCAGAGCGGGGAGCTTTCTCGATTGAAA	3133
37_PHR_non-APR	GAACA---GTGAAAAAAACGATGCGTCTATGGACTCTAGACCTCAGAGCGGGGAGCTTTCTCGATTGAAA	3132
52_PHR_non-APR	GAACA---GTGAAAAAAACGATGCGTCTATGGACTCTAGACCTCAGAGCGGGGAGCTTTCTCGATTGAAA	3713
88_PHR_non-APR	GAACA---GTGAAAAAAACGATGCGTCTATGGACTCTAGACCTCAGAGCGGGGAGCTTTCTCGATTGAAA	3114
101_PHR_non-APR	GAACA---GTGAAAAAAACGATGCGTCTATGGACTCTAGACCTCAGAGCGGGGAGCTTTCTCGATTGAAA	3135
105_PHR_non-APR	GAACA---GTGAAAAAAACGATGCGTCTATGGACTCTAGACCTCAGAGCGGGGAGCTTTCTCGATTGAAA	3134
150_PHR_non-APR	GAACA---GTGAAAAAAACGATGCGTCTATGGACTCTAGACCTCAGAGCGGGGAGCTTTCTCGATTGAAA	3076
150_PHR_non-APR2	GAACA---GTGAAAAAAACGATGCGTCTATGGACTCTAGACCTCAGAGCGGGGAGCTTTCTCGATTGAAA	3076

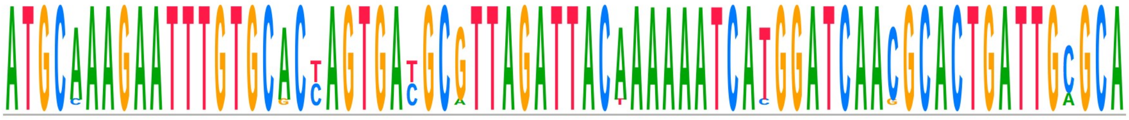

**Consensus**	ATGCAAAGAATTTGTGCACNAGTGANGCGTTAGATTACAAAAAATCATGGATCAACGCACTGATTGCGCA	
	4280 4290 4300 4310 4320 4330 4340	
Lr67(sus)	ATGCCAAGAATTTGTGCGCTAGTGATGCATTAGATTACTA------CACGGATCAAGGCACTGATTGAGCA	3285
Lr67(res)	ATGCCAAGAATTTGTGCGCTAGTGATGCATTAGATTACTA------CACGGATCAAGGCACTGATTGAGCA	3285
ScLr67_1 (Lo7)	ATGCAAAGAATTTGTGCACCAGTGACGCGTTAGATTACAAAAAATCATGGATCAACGCACTGATTGAGCA	3319
118_Danko_APR	ATGCAAAGAATTTGTGCACCAGTGACGCGTTAGATTACAAAAAATCATGGATCAACGCACTGATTGCGCA	3207
119_Danko_APR	ATGCAAAGAATTTGTGCACTAGTGATGCGTTAGATTACAAAAAATCATGGATCAACGCACTGATTGCGCA	3224
119_Danko_APR2	ATGCAAAGAATTTGTGCACTAGTGATGCGTTAGATTACAAAAAATCATGGATCAACGCACTGATTGCGCA	3224
120_Danko_APR	ATGCAAAGAATTTGTGCACTAGTGATGCGTTAGATTACAAAAAATCATGGATCAACGCACTGATTGCGCA	3226
138_Danko_APR	ATGCAAAGAATTTGTGCACTAGTGATGCGTTAGATTACAAAAAATCATGGATCAACGCACTGATTGCGCA	3222
153_Danko_APR	ATGCAAAGAATTTGTGCACCAGTGACGCGTTAGATTACAAAAAATCATGGATCAACGCACTGATTGCGCA	3201
157_Danko_APR	ATGCAAAGAATTTGTGCACTAGTGATGCGTTAGATTACAAAAAATCATGGATCAACGCACTGATTGCGCA	3224
160_Danko_APR	ATGCAAAGAATTTGTGCACTAGTGATGCGTTAGATTACAAAAAATCATGGATCAACGCACTGATTGCGCA	3214
71_PHR_APR	ATGCAAAGAATTTGTGCACTAGTGATGCGTTAGATTACAAAAAATCATGGATCAACGCACTGATTGCGCA	3293
149_PHR_APR	ATGCAAAGAATTTGTGCACCAGTGACGCGTTAGATTACAAAAAATCATGGATCAACGCACTGATTGCGCA	3205
59_Danko_non-APR	ATGCAAAGAATTTGTGCACTAGTGATGCGTTAGATTACAAAAAATCATGGATCAACGCACTGATTGCGCA	3245
61_Danko_non-APR	ATGCAAAGAATTTGTGCACCAGTGACGCGTTAGATTACAAAAAATCATGGATCAACGCACTGATTGAGCA	3271
123_Danko_non-APR	ATGCAAAGAATTTGTGCACCAGTGACGCGTTAGATTACAAAAAATCATGGATCAACGCACTGATTGCGCA	3156
129_Danko_non-APR	ATGCAAAGAATTTGTGCACCAGTGACGCGTTAGATTACAAAAAATCATGGATCAACGCACTGATTGCGCA	3203
37_PHR_non-APR	ATGCAAAGAATTTGTGCACTAGTGATGCGTTAGATTACAAAAAATCATGGATCAACGCACTGATTGCGCA	3202
52_PHR_non-APR	ATGCAAAGAATTTGTGCACTAGTGATGCGTTAGATTACAAAAAATCATGGATCAACGCACTGATTGAGCA	3783
88_PHR_non-APR	ATGCAAAGAATTTGTGCACCAGTGACGCGTTAGATTACAAAAAATCATGGATCAACGCACTGATTGCGCA	3184
101_PHR_non-APR	ATGCAAAGAATTTGTGCACCAGTGACGCGTTAGATTACAAAAAATCATGGATCAACGCACTGATTGCGCA	3205
105_PHR_non-APR	ATGCAAAGAATTTGTGCACCAGTGACGCGTTAGATTACAAAAAATCATGGATCAACGCACTGATTGCGCA	3204
150_PHR_non-APR	ATGCAAAGAATTTGTGCACCAGTGACGCGTTAGATTACAAAAAATCATGGATCAACGCACTGATTGCGCA	3146
150_PHR_non-APR2	ATGCAAAGAATTTGTGCACCAGTGACGCGTTAGATTACAAAAAATCATGGATCAACGCACTGATTGCGCA	3146

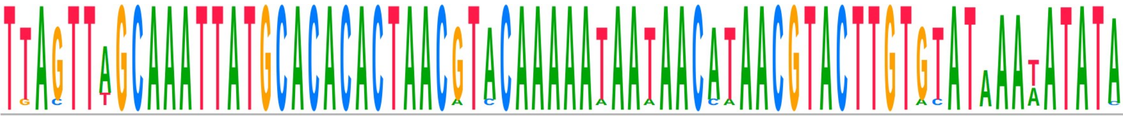

**Consensus**	TTAGTTAGCAAATTATGCACACTAACGTACAAAAATAATAACATAACGTACTTGTGTAT-AATATATA	
	4350 4360 4370 4380 4390 4400 4410	
Lr67(sus)	TGAGTTTGCAAATTATGCACACTAACATCCAAAAAAAAAAACCAAACGTACTTGTATA-------ATA	3348
Lr67(res)	TGAGTTTGCAAATTATGCACACTAACATCCAAAAAAAAAAACCAAACGTACTTGTATA-------ATA	3348
ScLr67_1 (Lo7)	TTACTTTGCAAATTATGCACACTAACGTACAAAAATAATAACATAACGTACTTGTGCATAAATATATA	3389
118_Danko_APR	TTAGTTAGCAAATTATGCACACTAACGTACAAAAATAATAACATAACGTACTTGTGTAT-AATATATA	3276
119_Danko_APR	TTAGTTAGCAAATTATGCACACTAACGTACAAAAATAATAACATAACGTACTTGTGTAT---AATATA	3291
119_Danko_APR2	TTAGTTAGCAAATTATGCACACTAACGTACAAAAATAATAACATAACGTACTTGTGTAT---AATATA	3291
120_Danko_APR	TTAGTTAGCAAATTATGCACACTAACGTACAAAAATAATAACATAACGTACTTGTGTAT---AATATA	3293
138_Danko_APR	TTAGTTAGCAAATTATGCACACTAACGTACAAAAATAATAACATAACGTACTTGTGTAT---AATATA	3289
153_Danko_APR	TTAGTTAGCAAATTATGCACACTAACGTACAAAAATAATAACATAACGTACTTGTGTAT-AATATATA	3270
157_Danko_APR	TTAGTTAGCAAATTATGCACACTAACGTACAAAAATAATAACATAACGTACTTGTGTAT---AATATA	3291
160_Danko_APR	TTAGTTAGCAAATTATGCACACTAACGTACAAAAATAATAACATAACGTACTTGTGTAT---AATATA	3281
71_PHR_APR	TTAGTTAGCAAATTATGCACACTAACGTACAAAAATAATAACATAACGTACTTGTGTAT---AATATA	3360
149_PHR_APR	TTAGTTAGCAAATTATGCACACTAACGTACAAAAATAATAACATAACGTACTTGTGTAT-AATATATA	3274
59_Danko_non-APR	TTAGTTAGCAAATTATGCACACTAACGTACAAAAATAATAACATAACGTACTTGTGTAT---AATATA	3312
61_Danko_non-APR	TTACTTTGCAAATTATGCACACTAACGTACAAAAATAATAACATAACGTACTTGTGCATAAATATATA	3341
123_Danko_non-APR	TTAGTTAGCAAATTATGCACACTAACGTACAAAAATAATAACATAACGTACTTGTGTAT-AATATATA	3225
129_Danko_non-APR	TTAGTTAGCAAATTATGCACACTAACGTACAAAAATAATAACATAACGTACTTGTGTAT-AATATATA	3272
37_PHR_non-APR	TTAGTTAGCAAATTATGCACACTAACGTACAAAAATAATAACATAACGTACTTGTGTAT---AATATA	3269
52_PHR_non-APR	TTAGTTTGCAAATTATGCACACTAACGTACAAAAATAATAACATAACGTACTTGTGTAT-AATATATC	3852
88_PHR_non-APR	TTAGTTAGCAAATTATGCACACTAACGTACAAAAATAATAACATAACGTACTTGTGTAT-AATATATA	3253
101_PHR_non-APR	TTAGTTAGCAAATTATGCACACTAACGTACAAAAATAATAACATAACGTACTTGTGTAT-AATATATA	3274
105_PHR_non-APR	TTAGTTAGCAAATTATGCACACTAACGTACAAAAATAATAACATAACGTACTTGTGTAT-AATATATA	3273
150_PHR_non-APR	TTAGTTAGCAAATTATGCACACTAACGTACAAAAATAATAACATAACGTACTTGTGTAT-AATATATA	3215
150_PHR_non-APR2	TTAGTTAGCAAATTATGCACACTAACGTACAAAAATAATAACATAACGTACTTGTGTAT-AATATATA	3215

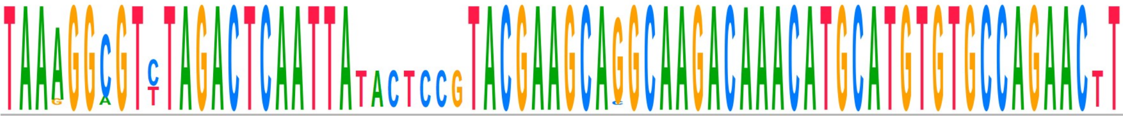

**Consensus**	TAAAGGCGTCTAGACTCAATTA-------TACGAAGCAGGCAAGACAAACATGCATGTGTGCCAGAAC-T	
	4420 4430 4440 4450 4460 4470 4480	
Lr67(sus)	TAAAGGCGTCTAGACTCAATTATACTCCGTACGAAGCAGGCAAGAC-AACATGCATGTGTGCCAGAACTT	3417
Lr67(res)	TAAAGGCGTCTAGACTCAATTATACTCCGTACGAAGCAGGCAAGAC-AACATGCATGTGTGCCAGAACTT	3417
ScLr67_1 (Lo7)	TAAGGGAGTCTAGACTCAATTA-------TACGAAGCAGGCAAGACAAACATGCATGTGTGCCAGAAC-T	3451
118_Danko_APR	TAAAGGCGTTTAGACTCAATTA-------TACGAAGCAGGCAAGACAAACATGCATGTGTGCCAGAAC-T	3338
119_Danko_APR	TAAAGGCGTCTAGACTCAATTA-------TACGAAGCAGGCAAGACAAACATGCATGTGTGCCAGAAC-T	3353
119_Danko_APR2	TAAAGGCGTCTAGACTCAATTA-------TACGAAGCAGGCAAGACAAACATGCATGTGTGCCAGAAC-T	3353
120_Danko_APR	TAAAGGCGTCTAGACTCAATTA-------TACGAAGCAGGCAAGACAAACATGCATGTGTGCCAGAAC-T	3355
138_Danko_APR	TAAAGGCGTCTAGACTCAATTA-------TACGAAGCAGGCAAGACAAACATGCATGTGTGCCAGAAC-T	3351
153_Danko_APR	TAAAGGCGTTTAGACTCAATTA-------TACGAAGCAGGCAAGACAAACATGCATGTGTGCCAGAAC-T	3332
157_Danko_APR	TAAAGGCGTCTAGACTCAATTA-------TACGAAGCAGGCAAGACAAACATGCATGTGTGCCAGAAC-T	3353
160_Danko_APR	TAAAGGCGTCTAGACTCAATTA-------TACGAAGCAGGCAAGACAAACATGCATGTGTGCCAGAAC-T	3343
71_PHR_APR	TAAAGGCGTCTAGACTCAATTA-------TACGAAGCAGGCAAGACAAACATGCATGTGTGCCAGAAC-T	3422
149_PHR_APR	TAAAGGCGTTTAGACTCAATTA-------TACGAAGCAGGCAAGACAAACATGCATGTGTGCCAGAAC-T	3336
59_Danko_non-APR	TAAAGGCGTCTAGACTCAATTA-------TACGAAGCAGGCAAGACAAACATGCATGTGTGCCAGAAC-T	3374
61_Danko_non-APR	TAAGGGAGTCTAGACTCAATTA-------TACGAAGCAGGCAAGACAAACATGCATGTGTGCCAGAAC-T	3403
123_Danko_non-APR	TAAAGGCGTTTAGACTCAATTA-------TACGAAGCAGGCAAGACAAACATGCATGTGTGCCAGAAC-T	3287
129_Danko_non-APR	TAAAGGCGTTTAGACTCAATTA-------TACGAAGCAGGCAAGACAAACATGCATGTGTGCCAGAAC-T	3334
37_PHR_non-APR	TAAAGGCGTCTAGACTCAATTA-------TACGAAGCAGGCAAGACAAACATGCATGTGTGCCAGAAC-T	3331
52_PHR_non-APR	TAAAGGAGTCTAGACTCAATTA-------TACGAAGCAGGCAAGACAAACATGCATGTGTGCCAGAAC-T	3914
88_PHR_non-APR	TAAAGGCGTTTAGACTCAATTA-------TACGAAGCACGCAAGACAAACATGCATGTGTGCCAGAAC-T	3315
101_PHR_non-APR	TAAAGGCGTTTAGACTCAATTA-------TACGAAGCAGGCAAGACAAACATGCATGTGTGCCAGAAC-T	3336
105_PHR_non-APR	TAAAGGCGTTTAGACTCAATTA-------TACGAAGCAGGCAAGACAAACATGCATGTGTGCCAGAAC-T	3335
150_PHR_non-APR	TAAAGGCGTTTAGACTCAATTA-------TACGAAGCAGGCAAGACAAACATGCATGTGTGCCAGAAC-T	3277
150_PHR_non-APR2	TAAAGGCGTTTAGACTCAATTA-------TACGAAGCAGGCAAGACAAACATGCATGTGTGCCAGAAC-T	3277

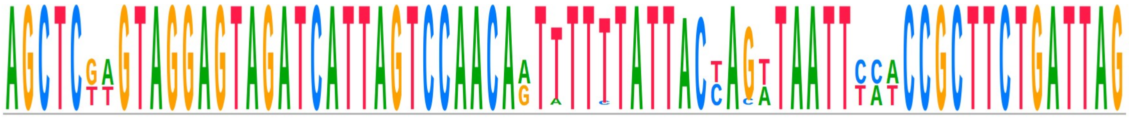

Name	Sequence (4481–4550)	End
**Consensus**	**AGCTCGAGTAGGAGTAGATCATTAGTCCAACAATTTTTTATT--NAGTTAATTCCACCGCTTCTGATTAG**	
	4490 … 4500 … 4510 … 4520 … 4530 … 4540 … 4550	
Lr67(sus)	AGCTCGAGTAGGAGTAGATCATTAGTCCAACAATATTTTATT---ACTTAATTCCACCGCTTCTGATTAG	3484
Lr67(res)	AGCTCGAGTAGGAGTAGATCATTAGTCCAACAATATTTTATT---ACTTAATTCCACCGCTTCTGATTAG	3484
ScLr67_1 (Lo7)	AGCTCGAGTAGGAGTAGATCATTAGTCCAACAGTTTTTTATT--CAGTTAATTTATCCGCTTCTGATTAG	3519
118_Danko_APR	AGCTCTTGTAGGAGTAGATCATTAGTCCAACAATTTTTTATTACTAGATAATTCCACCGCTTCTGATTAG	3408
119_Danko_APR	AGCTCGAGTAGGAGTAGATCATTAGTCCAACAGTTTTTTATT--CAGTTAATTTATCCGCTTCTGATTAG	3421
119_Danko_APR2	AGCTCGAGTAGGAGTAGATCATTAGTCCAACAGTTTTTTATT--CAGTTAATTTATCCGCTTCTGATTAG	3421
120_Danko_APR	AGCTCGAGTAGGAGTAGATCATTAGTCCAACAGTTTTTTATT--CAGTTAATTTATCCGCTTCTGATTAG	3423
138_Danko_APR	AGCTCGAGTAGGAGTAGATCATTAGTCCAACAGTTTTTTATT--CAGTTAATTTATCCGCTTCTGATTAG	3419
153_Danko_APR	AGCTCTTGTAGGAGTAGATCATTAGTCCAACAATTTTTTATTACTAGATAATTCCACCGCTTCTGATTAG	3402
157_Danko_APR	AGCTCGAGTAGGAGTAGATCATTAGTCCAACAGTTTTTTATT--CAGTTAATTTATCCGCTTCTGATTAG	3421
160_Danko_APR	AGCTCGAGTAGGAGTAGATCATTAGTCCAACAGTTTTTTATT--CAGTTAATTTATCCGCTTCTGATTAG	3411
71_PHR_APR	AGCTCGAGTAGGAGTAGATCATTAGTCCAACAGTTTTTTATT--CAGTTAATTTATCCGCTTCTGATTAG	3490
149_PHR_APR	AGCTCTTGTAGGAGTAGATCATTAGTCCAACAATTTTTTATTACTAGATAATTCCACCGCTTCTGATTAG	3406
59_Danko_non-APR	AGCTCGAGTAGGAGTAGATCATTAGTCCAACAGTTTTTTATT--CAGTTAATTTATCCGCTTCTGATTAG	3442
61_Danko_non-APR	AGCTCGAGTAGGAGTAGATCATTAGTCCAACAGTTTTTTATT--CAGTTAATTTATCCGCTTCTGATTAG	3471
123_Danko_non-APR	AGCTCTTGTAGGAGTAGATCATTAGTCCAACAATTTTTTATTACTAGATAATTCCACCGCTTCTGATTAG	3357
129_Danko_non-APR	AGCTCTTGTAGGAGTAGATCATTAGTCCAACAATTTTTTATTACTAGATAATTCCACCGCTTCTGATTAG	3404
37_PHR_non-APR	AGCTCGAGTAGGAGTAGATCATTAGTCCAACAGTTTTTTATT--CAGTTAATTTATCCGCTTCTGATTAG	3399
52_PHR_non-APR	AGCTCGAGTAGGAGTAGATCATTAGTCCAACAATTTTCTATTACTAGTTAATTCCACCGCTTCTGATTAG	3984
88_PHR_non-APR	AGCTCTTGTAGGAGTAGATCATTAGTCCAACAATTTTTTATTACTAGATAATTCCACCGCTTCTGATTAG	3385
101_PHR_non-APR	AGCTCTTGTAGGAGTAGATCATTAGTCCAACAATTTTTTATTACTAGATAATTCCACCGCTTCTGATTAG	3406
105_PHR_non-APR	AGCTCTTGTAGGAGTAGATCATTAGTCCAACAATTTTTTATTACTAGATAATTCCACCGCTTCTGATTAG	3405
150_PHR_non-APR	AGCTCTTGTAGGAGTAGATCATTAGTCCAACAATTTTTTATTACTAGATAATTCCACCGCTTCTGATTAG	3347
150_PHR_non-APR2	AGCTCTTGTAGGAGTAGATCATTAGTCCAACAATTTTTTATTACTAGATAATTCCACCGCTTCTGATTAG	3347

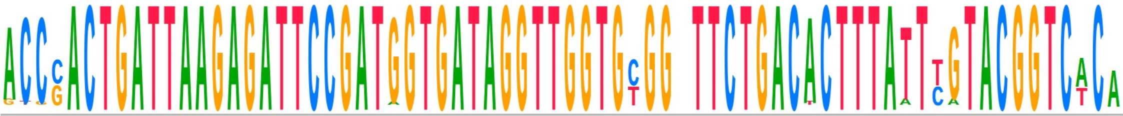

Name	Sequence (4551–4620)	End
**Consensus**	**ACCCACTGATTAAGAGATTCCGATGGTGATAGGTTGGTGCGG-TTCTGACACTTTATTTGTACGGTCAC-**	
	4560 … 4570 … 4580 … 4590 … 4600 … 4610 … 4620	
Lr67(sus)	GCCCACTGATTAAGAGATTCCGATGGTGATAGGTTGGTGCGG-TTCTGACACTTTAATTATACGGTCACA	3553
Lr67(res)	GCCCACTGATTAAGAGATTCCGATGGTGATAGGTTGGTGCGG-TTCTGACACTTTAATTATACGGTCACA	3553
ScLr67_1 (Lo7)	ACCCACTGATTAAGAGATTCCGATGGTGATAGGTTGGTGCGG-TTCTGACACTTTATTTGTACGGTCAC-	3587
118_Danko_APR	ACCGACTGATTAAGAGATTCCGATGGTGATAGGTTGGTGTGG-TTCTGACACTTTATTCGTACGGTCAC-	3476
119_Danko_APR	ACCCACTGATTAAGAGATTCCGATGGTGATAGGTTGGTGCGG-TTCTGACACTTTATTTGTACGGTCTC-	3489
119_Danko_APR2	ACCCACTGATTAAGAGATTCCGATGGTGATAGGTTGGTGCGG-TTCTGACACTTTATTTGTACGGTCTC-	3489
120_Danko_APR	ACCCACTGATTAAGAGATTCCGATGGTGATAGGTTGGTGCGG-TTCTGACACTTTATTTGTACGGTCTC-	3491
138_Danko_APR	ACCCACTGATTAAGAGATTCCGATGGTGATAGGTTGGTGCGG-TTCTGACACTTTATTTGTACGGTCTC-	3487
153_Danko_APR	ACCGACTGATTAAGAGATTCCGATGGTGATAGGTTGGTGTGG-TTCTGACACTTTATTCGTACGGTCAC-	3470
157_Danko_APR	ACCCACTGATTAAGAGATTCCGATGGTGATAGGTTGGTGCGG-TTCTGACACTTTATTTGTACGGTCTC-	3489
160_Danko_APR	ACCCACTGATTAAGAGATTCCGATGGTGATAGGTTGGTGCGG-TTCTGACACTTTATTTGTACGGTCTC-	3479
71_PHR_APR	ACCCACTGATTAAGAGATTCCGATGGTGATAGGTTGGTGCGG-TTCTGACACTTTATTTGTACGGTCTC-	3558
149_PHR_APR	ACCGACTGATTAAGAGATTCCGATGGTGATAGGTTGGTGTGGTTTCTGACACTTTATTCGTACGGTCAC-	3475
59_Danko_non-APR	ACCCACTGATTAAGAGATTCCGATGGTGATAGGTTGGTGCGG-TTCTGACACTTTATTTGTACGGTCTC-	3510
61_Danko_non-APR	ACCCACTGATTAAGAGATTCCGATGGTGATAGGTTGGTGCGG-TTCTGACACTTTATTTGTACGGTCAC-	3539
123_Danko_non-APR	ACCGACTGATTAAGAGATTCCGATGGTGATAGGTTGGTGTGG-TTCTGACACTTTATTCGTACGGTCAC-	3425
129_Danko_non-APR	ACCGACTGATTAAGAGATTCCGATGGTGATAGGTTGGTGTGG-TTCTGACACTTTATTCGTACGGTCAC-	3472
37_PHR_non-APR	ACCCACTGATTAAGAGATTCCGATGGTGATAGGTTGGTGCGG-TTCTGACACTTTATTTGTACGGTCTC-	3467
52_PHR_non-APR	ATGGACTGATTAAGAGATTCCGATAGTGATAGGTTGGTGCGG-TTCTGACTCTTTATTTGTACGGTCAC-	4052
88_PHR_non-APR	ACCGACTGATTAAGAGATTCCGATGGTGATAGGTTGGTGTGG-TTCTGACACTTTATTCGTACGGTCAC-	3453
101_PHR_non-APR	ACCGACTGATTAAGAGATTCCGATGGTGATAGGTTGGTGTGG-TTCTGACACTTTATTCGTACGGTCAC-	3474
105_PHR_non-APR	ACCGACTGATTAAGAGATTCCGATGGTGATAGGTTGGTGTGG-TTCTGACACTTTATTCGTACGGTCAC-	3473
150_PHR_non-APR	ACCGACTGATTAAGAGATTCCGATGGTGATAGGTTGGTGTGG-TTCTGACACTTTATTCGTACGGTCAC-	3415
150_PHR_non-APR2	ACCGACTGATTAAGAGATTCCGATGGTGATAGGTTGGTGTGG-TTCTGACACTTTATTCGTACGGTCAC-	3415

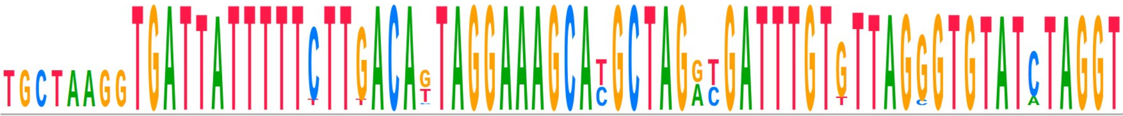

```
Consensus            --------TGATTNTTTTTCTTGACAGTAGGAAAGCATGCTAGNTGATTTGTGTTAGNGTNNNNNNNN
                             4630      4640      4650      4660      4670      4680      4690
Lr67(sus)            TGCTAAGGTGAT--TTTTTTTTTACAGTAGGAAAGCATGCTAGGTGATTTGTTT----------------  3605
Lr67(res)            TGCTAAGGTGAT--TTTTTTTTTACAGTAGGAAAGCATGCTAGGTGATTTGTTT----------------  3605
ScLr67_1 (Lo7)       --------TGATTATTTTTCTTGACAGTAGGAAAGCATGCTAGATGATTTGTGTTAGGGTGTATATAGGT  3649
118_Danko_APR        --------TGATTATTTTTCTTGACATTAGGAAAGCACGCTAGACGATTTGTGTTAGGGTGTATCTAGGT  3538
119_Danko_APR        --------TGAT--TTTTTCTTGACAGTAGGAAAGCATGCTAGGTGATTTGT------------------  3531
119_Danko_APR2       --------TGAT--TTTTTCTTGACAGTAGGAAAGCATGCTAGGTGATTTGT------------------  3531
120_Danko_APR        --------TGAT--TTTTTCTTGACAGTAGGAAAGCATGCTAGGTGATTTGT------------------  3533
138_Danko_APR        --------TGAT--TTTTTCTTGACAGTAGGAAAGCATGCTAGGTGATTTGT------------------  3529
153_Danko_APR        --------TGATTATTTTTCTTGACATTAGGAAAGCACGCTAGACGATTTGTGTTAGGGTGTATCTAGGT  3532
157_Danko_APR        --------TGAT--TTTTTCTTGACAGTAGGAAAGCATGCTAGGTGATTTGT------------------  3531
160_Danko_APR        --------TGAT--TTTTTCTTGACAGTAGGAAAGCATGCTAGGTGATTTGT------------------  3521
71_PHR_APR           --------TGAT--TTTTTCTTGACAGTAGGAAAGCATGCTAGGTGATTTGT------------------  3600
149_PHR_APR          --------TGATTATTTTTCTTGACATTAGGAAAGCACGCTAGACGATTTGTGTTAGGGTGTATCTAGGT  3537
59_Danko_non-APR     --------TGAT--TTTTTCTTGACAGTAGGAAAGCATGCTAGGTGATTTGT------------------  3552
61_Danko_non-APR     --------TGATTATTTTTCTTGACAGTAGGAAAGCATGCTAGATGATTTGTGTTAGGGTGTATATAGGT  3601
123_Danko_non-APR    --------TGATTATTTTTCTTGACATTAGGAAAGCACGCTAGACGATTTGTGTTAGGGTGTATCTAGGT  3487
129_Danko_non-APR    --------TGATTATTTTTCTTGACATTAGGAAAGCACGCTAGACGATTTGTGTTAGGGTGTATCTAGGT  3534
37_PHR_non-APR       --------TGAT--TTTTTCTTGACAGTAGGAAAGCATGCTAGGTGATTTGT------------------  3509
52_PHR_non-APR       --------TGATT-TTTTTCTTGACACTAGGAAAGCATGCTAGGTGATTTGTGTTAGCGT----------  4103
88_PHR_non-APR       --------TGATTATTTTTCTTGACATTAGGAAAGCACGCTAGACGATTTGTGTTAGGGTGTATCTAGGT  3515
101_PHR_non-APR      --------TGATTATTTTTCTTGACATTAGGAAAGCACGCTAGACGATTTGTGTTAGGGTGTATCTAGGT  3536
105_PHR_non-APR      --------TGATTATTTTTCTTGACATTAGGAAAGCACGCTAGACGATTTGTGTTAGGGTGTATCTAGGT  3535
150_PHR_non-APR      --------TGATTATTTTTCTTGACATTAGGAAAGCACGCTAGACGATTTGTGTTAGGGTGTATCTAGGT  3477
150_PHR_non-APR2     --------TGATTATTTTTCTTGACATTAGGAAAGCACGCTAGACGATTTGTGTTAGGGTGTATCTAGGT  3477
```

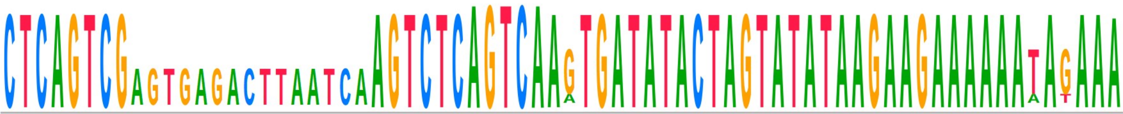

```
Consensus            NNNNNNNN---------------NNNNNNAGTCANNNNNNNNNNNNNNNNNNNNNNNNNNNNNNNNNN-
                             4700      4710      4720      4730      4740      4750      4760
Lr67(sus)            ----------------------------------------------------------------------  3605
Lr67(res)            ----------------------------------------------------------------------  3605
ScLr67_1 (Lo7)       CTCAGTCGAGTGAGACTTAATCAAGTCTCAGTCAAATGATATACTAGTATATAAGAAGAAAAAAAATAA-  3718
118_Danko_APR        CTCAGTCG---------------AGTCTCAGTCAAGTGATATACTAGTATATAAGAAGAAAAAAATAGAAA  3593
119_Danko_APR        -----------------------------AGTCA------------------------------------  3536
119_Danko_APR2       -----------------------------AGTCA------------------------------------  3536
120_Danko_APR        -----------------------------AGTCA------------------------------------  3538
138_Danko_APR        -----------------------------AGTCA------------------------------------  3534
153_Danko_APR        CTCAGTCG---------------AGTCTCAGTCAAGTGATATACTAGTATATAAGAAGAAAAAAATAGAAA  3587
157_Danko_APR        -----------------------------AGTCA------------------------------------  3536
160_Danko_APR        -----------------------------AGTCA------------------------------------  3526
71_PHR_APR           -----------------------------AGTCA------------------------------------  3605
149_PHR_APR          CTCAGTCG---------------AGTCTCAGTCAAGTGATATACTAGTATATAAGAAGAAAAAAATAGAAA  3592
59_Danko_non-APR     -----------------------------AGTCA------------------------------------  3557
61_Danko_non-APR     CTCAGTCGAGTGAGACTTAATCAAGTCTCAGTCAAATGATATACTAGTATATAAGAAGAAAAAAAATAA-  3670
123_Danko_non-APR    CTCAGTCG---------------AGTCTCAGTCAAGTGATATACTAGTATATAAGAAGAAAAAAATAGAAA  3542
129_Danko_non-APR    CTCAGTCG---------------AGTCTCAGTCAAGTGATATACTAGTATATAAGAAGAAAAAAATAGAAA  3589
37_PHR_non-APR       -----------------------------AGTCA------------------------------------  3514
52_PHR_non-APR       ----------------------------------------------------------------------  4103
88_PHR_non-APR       CTCAGTCG---------------AGTCTCAGTCAAGTGATATACTAGTATATAAGAAGAAAAAAATAGAAA  3570
101_PHR_non-APR      CTCAGTCG---------------AGTCTCAGTCAAGTGATATACTAGTATATAAGAAGAAAAAAATAGAAA  3591
105_PHR_non-APR      CTCAGTCG---------------AGTCTCAGTCAAGTGATATACTAGTATATAAGAAGAAAAAAATAGAAA  3590
150_PHR_non-APR      CTCAGTCG---------------AGTCTCAGTCAAGTGATATACTAGTATATAAGAAGAAAAAAATAGAAA  3532
150_PHR_non-APR2     CTCAGTCG---------------AGTCTCAGTCAAGTGATATACTAGTATATAAGAAGAAAAAAATAGAAA  3532
```

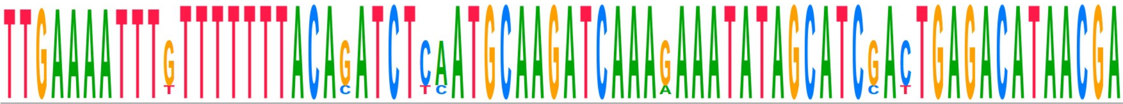

```
Consensus          ----------NNNNNNNNNNNNNNNNNNNNNNNNNNNNNNNNNNNNNNNNNNNNNNNNNNNNNNNNNNNNNN
                             4770      4780      4790      4800      4810      4820      4830

Lr67(sus)          ----------------------------------------------------------------------  3605
Lr67(res)          ----------------------------------------------------------------------  3605
ScLr67_1 (Lo7)     ----------TTTTTTTTACACATCTTCATGCAAGATCAAAAAAATATAGCATCCATTGAGACATAACGA  3778
118_Danko_APR      TTGAAAATTTGTTTTTTTACAGATCTCAATGCAAGATCAAAGAAATATAGCATCGACTGAGACATAACGA  3663
119_Danko_APR      ----------------------------------------------------------------------  3536
119_Danko_APR2     ----------------------------------------------------------------------  3536
120_Danko_APR      ----------------------------------------------------------------------  3538
138_Danko_APR      ----------------------------------------------------------------------  3534
153_Danko_APR      TTGAAAATTTGTTTTTTTACAGATCTCAATGCAAGATCAAAGAAATATAGCATCGACTGAGACATAACGA  3657
157_Danko_APR      ----------------------------------------------------------------------  3536
160_Danko_APR      ----------------------------------------------------------------------  3526
71_PHR_APR         ----------------------------------------------------------------------  3605
149_PHR_APR        TTGAAAATTTGTTTTTTTACAGATCTCAATGCAAGATCAAAGAAATATAGCATCGACTGAGACATAACGA  3662
59_Danko_non-APR   ----------------------------------------------------------------------  3557
61_Danko_non-APR   ----------TTTTTTTTACACATCTTCATGCAAGATCAAAAAAATATAGCATCCATTGAGACATAACGA  3730
123_Danko_non-APR  TTGAAAATTTGTTTTTTTACAGATCTCAATGCAAGATCAAAGAAATATAGCATCGACTGAGACATAACGA  3612
129_Danko_non-APR  TTGAAAATTTGTTTTTTTACAGATCTCAATGCAAGATCAAAGAAATATAGCATCGACTGAGACATAACGA  3659
37_PHR_non-APR     ----------------------------------------------------------------------  3514
52_PHR_non-APR     ----------------------------------------------------------------------  4103
88_PHR_non-APR     TTGAAAATTTGTTTTTTTACAGATCTCAATGCAAGATCAAAGAAATATAGCATCGACTGAGACATAACGA  3640
101_PHR_non-APR    TTGAAAATTTGTTTTTTTACAGATCTCAATGCAAGATCAAAGAAATATAGCATCGACTGAGACATAACGA  3661
105_PHR_non-APR    TTGAAAATTTGTTTTTTTACAGATCTCAATGCAAGATCAAAGAAATATAGCATCGACTGAGACATAACGA  3660
150_PHR_non-APR    TTGAAAATTTGTTTTTTTACAGATCTCAATGCAAGATCAAAGAAATATAGCATCGACTGAGACATAACGA  3602
150_PHR_non-APR2   TTGAAAATTTGTTTTTTTACAGATCTCAATGCAAGATCAAAGAAATATAGCATCGACTGAGACATAACGA  3602
```

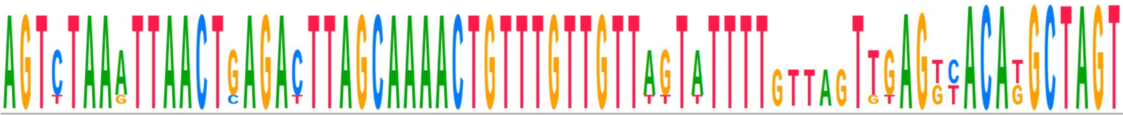

```
Consensus          NNNNNNNNNNNNNNNNNNNNNNNNNNNNNNNNNNNNNNNNNNNNNNNN-----NNNAGNNACANGCTAGT
                           4840      4850      4860      4870      4880      4890      4900

Lr67(sus)          ---------------------------------------------------------------------T  3606
Lr67(res)          ---------------------------------------------------------------------T  3606
ScLr67_1 (Lo7)     AGTTTAAGTTAACTCAGATTTAGCAAAACTGTTTGTTGTTTTTTTTTTGTTAGTGTAGTCACATGCTAGT  3848
118_Danko_APR      AGTCTAAATTAACTGAGACTTAGCAAAACTGTTTGTTGTTAGTATTTT-----TTGAGGTACAGGCTAGT  3728
119_Danko_APR      ----------------------------------------------------------TCACATGCTAGT  3548
119_Danko_APR2     ----------------------------------------------------------TCACATGCTAGT  3548
120_Danko_APR      ----------------------------------------------------------TCACATGCTAGT  3550
138_Danko_APR      ----------------------------------------------------------TCACATGCTAGT  3546
153_Danko_APR      AGTCTAAATTAACTGAGACTTAGCAAAACTGTTTGTTGTTAGTATTTT-----TTGAGGTACAGGCTAGT  3722
157_Danko_APR      ----------------------------------------------------------TCACATGCTAGT  3548
160_Danko_APR      ----------------------------------------------------------TCACATGCTAGT  3538
71_PHR_APR         ----------------------------------------------------------TCACATGCTAGT  3617
149_PHR_APR        AGTCTAAATTAACTGAGACTTAGCAAAACTGTTTGTTGTTAGTATTTT-----TTGAGGTACAGGCTAGT  3727
59_Danko_non-APR   ----------------------------------------------------------TCACATGCTAGT  3569
61_Danko_non-APR   AGTTTAAGTTAACTCAGATTTAGCAAAACTGTTTGTTGTTTTTTTTTTGTTAGTGTAGTCACATGCTAGT  3800
123_Danko_non-APR  AGTCTAAATTAACTGAGACTTAGCAAAACTGTTTGTTGTTAGTATTTT-----TTGAGGTACAGGCTAGT  3677
129_Danko_non-APR  AGTCTAAATTAACTGAGACTTAGCAAAACTGTTTGTTGTTAGTATTTT-----TTGAGGTACAGGCTAGT  3724
37_PHR_non-APR     ----------------------------------------------------------TCACATGCTAGT  3526
52_PHR_non-APR     --------------------------------------------------------AGTCACATGCTAGT  4117
88_PHR_non-APR     AGTCTAAATTAACTGAGACTTAGCAAAACTGTTTGTTGTTAGTATTTT-----TTGAGGTACAGGCTAGT  3705
101_PHR_non-APR    AGTCTAAATTAACTGAGACTTAGCAAAACTGTTTGTTGTTAGTATTTT-----TTGAGGTACAGGCTAGT  3726
105_PHR_non-APR    AGTCTAAATTAACTGAGACTTAGCAAAACTGTTTGTTGTTAGTATTTT-----TTGAGGTACAGGCTAGT  3725
150_PHR_non-APR    AGTCTAAATTAACTGAGACTTAGCAAAACTGTTTGTTGTTAGTATTTT-----TTGAGGTACAGGCTAGT  3667
150_PHR_non-APR2   AGTCTAAATTAACTGAGACTTAGCAAAACTGTTTGTTGTTAGTATTTT-----TTGAGGTACAGGCTAGT  3667
```

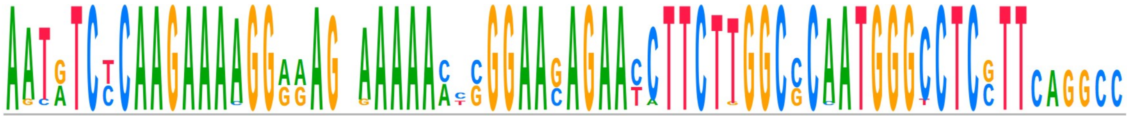

```
Consensus            AATGTCTCAAGAAAAGGAAAN-AAAAANNCGGAAGAGAACCTTCTTGGCCCAATGGGCCTCNTT------
                               4910      4920      4930      4940      4950      4960      4970
Lr67(sus)            AGCATCCCAAGAAAAGGAAAG-AAAAAAGGGGAAGAGAACATTC-TGGCCCAATGGGCCTCGTTCAGGCC  3674
Lr67(res)            AGCATCCCAAGAAAAGGAAAG-AAAAAAGGGGAAGAGAACATTC-TGGCCCAATGGGCCTCGTTCAGGCC  3674
ScLr67_1 (Lo7)       AATGTCTCAAGAAAAGGAA---GAAAACCCGGAAGAGAACCTTCTTGGCCCAATGGGTCTCCTT------  3909
118_Danko_APR        AATGTCTCAAGAAAAGGGGA--AAAAACCCGGAAGAGAATCTTCTTGGCCCAATGGGCCTCCTT------  3790
119_Danko_APR        AATATCCCAAGAAAAGGAAAG-AAAAAATGGGAACAGAACCTTC-TGGCGCAATGGGCCTCGTT------  3610
119_Danko_APR2       AATATCCCAAGAAAAGGAAAG-AAAAAATGGGAACAGAACCTTC-TGGCGCAATGGGCCTCGTT------  3610
120_Danko_APR        AATATCCCAAGAAAAGGAAAG-AAAAAATGGGAACAGAACCTTC-TGGCGCAATGGGCCTCGTT------  3612
138_Danko_APR        AATATCCCAAGAAAAGGAAAG-AAAAAATGGGAACAGAACCTTC-TGGCGCAATGGGCCTCGTT------  3608
153_Danko_APR        AATGTCTCAAGAAAAGGGGA--AAAAACCCGGAAGAGAATCTTCTTGGCCCAATGGGCCTCCTT------  3784
157_Danko_APR        AATATCCCAAGAAAAGGAAAG-AAAAAATGGGAACAGAACCTTC-TGGCGCAATGGGCCTCGTT------  3610
160_Danko_APR        AATATCCCAAGAAAAGGAAAG-AAAAAATGGGAACAGAACCTTC-TGGCGCAATGGGCCTCGTT------  3600
71_PHR_APR           AATATCCCAAGAAAAGGAAAG-AAAAAATGGGAACAGAACCTTC-TGGCGCAATGGGCCTCGTT------  3679
149_PHR_APR          AATGTCTCAAGAAAAGGGGA--AAAAACCCGGAAGAGAATCTTCTTGGCCCAATGGGCCTCCTT------  3789
59_Danko_non-APR     AATATCCCAAGAAAAGGAAAG-AAAAAATGGGAACAGAACCTTC-TGGCGCAATGGGCCTCGTT------  3631
61_Danko_non-APR     AATGTCTCAAGAAAAGGAA---GAAAACCCGGAAGAGAACCTTCTTGGCCCAATGGGTCTCCTT------  3861
123_Danko_non-APR    AATGTCTCAAGAAAAGGGGA--AAAAACCCGGAAGAGAATCTTCTTGGCCCAATGGGCCTCCTT------  3739
129_Danko_non-APR    AATGTCTCAAGAAAAGGGGA--AAAAACCCGGAAGAGAATCTTCTTGGCCCAATGGGCCTCCTT------  3786
37_PHR_non-APR       AATATCCCAAGAAAAGGAAAG-AAAAAATGGGAACAGAACCTTC-TGGCGCAATGGGCCTCGTT------  3588
52_PHR_non-APR       AATGTCTCAAGAAACGGAAAGAAAAAAAACGGAAGAGAACCTTCTGGGCCCCATGGGCCTCGTT------  4181
88_PHR_non-APR       AATGTCTCAAGAAAAGGGGA--AAAAACCCGGAAGAGAATCTTCTTGGCCCAATGGGCCTCCTT------  3767
101_PHR_non-APR      AATGTCTCAAGAAAAGGGGA--AAAAACCCGGAAGAGAATCTTCTTGGCCCAATGGGCCTCCTT------  3788
105_PHR_non-APR      AATGTCTCAAGAAAAGGGGA--AAAAACCCGGAAGAGAATCTTCTTGGCCCAATGGGCCTCCTT------  3787
150_PHR_non-APR      AATGTCTCAAGAAAAGGGGA--AAAAACCCGGAAGAGAATCTTCTTGGCCCAATGGGCCTCCTT------  3729
150_PHR_non-APR2     AATGTCTCAAGAAAAGGGGA--AAAAACCCGGAAGAGAATCTTCTTGGCCCAATGGGCCTCCTT------  3729
```

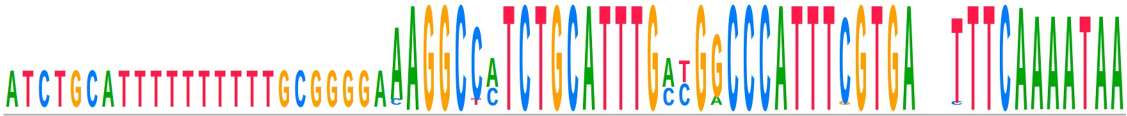

```
Consensus            ----------------------AAGGCCATCTGCATTTGANGGCCCATTTCGTGA--TTTCANNNNNN
                               4980      4990      5000      5010      5020      5030      5040
Lr67(sus)            ATCTGCATTTTTTTTTTGCGGGGACAGGCCATCTGCATTTGATGACCCATTTCGTGA--TTTC-------  3735
Lr67(res)            ATCTGCATTTTTTTTTTGCGGGGACAGGCCATCTGCATTTGATGACCCATTTCGTGA--TTTC-------  3735
ScLr67_1 (Lo7)       ----------------------AAGGCTATCTGCATTTGACGGCCCATTTCGTGA--TTTCAAAATAA  3953
118_Danko_APR        ----------------------AAGGCCATCTGCATTTGACGGCCCATTTCGTGA--TTTCAAAATAA  3834
119_Danko_APR        ----------------------AAGGCCCTCTGCATTTGCTGGCCCATTTCGTGA--TTTCA------  3648
119_Danko_APR2       ----------------------AAGGCCCTCTGCATTTGCTGGCCCATTTCGTGA--TTTCA------  3648
120_Danko_APR        ----------------------AAGGCCCTCTGCATTTGCTGGCCCATTTCGTGA--TTTCA------  3650
138_Danko_APR        ----------------------AAGGCCCTCTGCATTTGCTGGCCCATTTCGTGA--TTTCA------  3646
153_Danko_APR        ----------------------AAGGCCATCTGCATTTGACGGCCCATTTCGTGA--TTTCAAAATAA  3828
157_Danko_APR        ----------------------AAGGCCCTCTGCATTTGCTGGCCCATTTCGTGA--TTTCA------  3648
160_Danko_APR        ----------------------AAGGCCCTCTGCATTTGCTGGCCCATTTCGTGA--TTTCA------  3638
71_PHR_APR           ----------------------AAGGCCCTCTGCATTTGCTGGCCCATTTCGTGA--TTTCA------  3717
149_PHR_APR          ----------------------AAGGCCATCTGCATTTGACGGCCCATTTCGTGA--TTTCAAAATAA  3833
59_Danko_non-APR     ----------------------AAGGCCCTCTGCATTTGCTGGCCCATTTCGTGA--TTTCA------  3669
61_Danko_non-APR     ----------------------AAGGCTATCTGCATTTGACGGCCCATTTCGTGA--TTTCAAAATAA  3905
123_Danko_non-APR    ----------------------AAGGCCATCTGCATTTGACGGCCCATTTCGTGA--TTTCAAAATAA  3783
129_Danko_non-APR    ----------------------AAGGCCATCTGCATTTGACGGCCCATTTCGTGA--TTTCAAAATAA  3830
37_PHR_non-APR       ----------------------AAGGCCCTCTGCATTTGCTGGCCCATTTCGTGA--TTTCA------  3626
52_PHR_non-APR       ----------------------AAGGCCATCTGCATTTGATGACCCATTTGGTGAACCTTC-------  4220
88_PHR_non-APR       ----------------------AAGGCCATCTGCATTTGACGGCCCATTTCGTGA--TTTCAAAATAA  3811
101_PHR_non-APR      ----------------------AAGGCCATCTGCATTTGACGGCCCATTTCGTGA--TTTCAAAATAA  3832
105_PHR_non-APR      ----------------------AAGGCCATCTGCATTTGACGGCCCATTTCGTGA--TTTCAAAATAA  3831
150_PHR_non-APR      ----------------------AAGGCCATCTGCATTTGACGGCCCATTTCGTGA--TTTCAAAATAA  3773
150_PHR_non-APR2     ----------------------AAGGCCATCTGCATTTGACGGCCCATTTCGTGA--TTTCAAAATAA  3773
```

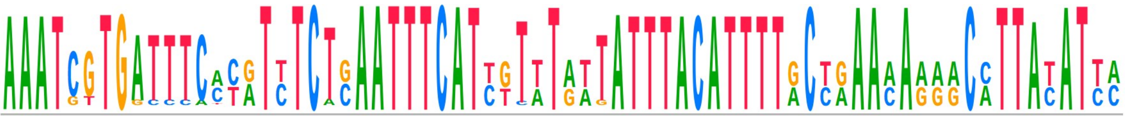

**Consensus**	NNNNNNTGNNNNNNCNTNTCTNAATTTCATNNTTTATNANNNNNNNNNNNCNNAANANNNCNTT-NATTN	
	5050 5060 5070 5080 5090 5100 5110	
Lr67(sus)	-------------CCGTCTCAGAATTTC---------------------------------------TTC	3753
Lr67(res)	-------------CCGTCTCAGAATTTC---------------------------------------TTC	3753
ScLr67_1 (Lo7)	AAATGTTGATTTCACATTTCTCAATTTCATCGTTTATTATTTACATTTTGCTGAAAAAAACCTT-TATTA	4022
118_Danko_APR	AAATCGTGATTTCACATTTCTCAATTTCATTGTTTATTATTTACATTTTGCTGAAAAAAACATT-TATTA	3903
119_Danko_APR	-------------CTGTCTCTGAATTTCATCTTATGA------------ACCAAACAGGGCCTTACATCC	3693
119_Danko_APR2	-------------CTGTCTCTGAATTTCATCTTATGA------------ACCAAACAGGGCCTTACATCC	3693
120_Danko_APR	-------------CTGTCTCTGAATTTCATCTTATGA------------ACCAAACAGGGCCTTACATCC	3695
138_Danko_APR	-------------CTGTCTCTGAATTTCATCTTATGA------------ACCAAACAGGGCCTTACATCC	3691
153_Danko_APR	AAATCGTGATTTCACATTTCTCAATTTCATTGTTTATTATTTACATTTTGCTGAAAAAAACATT-TATTA	3897
157_Danko_APR	-------------CTGTCTCTGAATTTCATCTTATGA------------ACCAAACAGGGCCTTACATCC	3693
160_Danko_APR	-------------CTGTCTCTGAATTTCATCTTATGA------------ACCAAACAGGGCCTTACATCC	3683
71_PHR_APR	-------------CTGTCTCTGAATTTCATCTTATGA------------ACCAAACAGGGCCTTACATCC	3762
149_PHR_APR	AAATCGTGATTTCACATTTCTCAATTTCATTGTTTATTATTTACATTTTGCTGAAAAAAACATT-TATTA	3902
59_Danko_non-APR	-------------CTGTCTCTGAATTTCAT--CTTATGA----------ACCAAACAGGGCCTTACATCC	3714
61_Danko_non-APR	AAATGTTGATTTCACATTTCTCAATTTCATCGTTTATTATTTACATTTTGCTGAAAAAAACCTT-TATTA	3974
123_Danko_non-APR	AAATCGTGATTTCACATTTCTCAATTTCATTGTTTATTATTTACATTTTGCTGAAAAAAACATT-TATTA	3852
129_Danko_non-APR	AAATCGTGATTTCACATTTCTCAATTTCATTGTTTATTATTTACATTTTGCTGAAAAAAACATT-TATTA	3899
37_PHR_non-APR	-------------CTGTCTCTGAATTTCATCTTATGA------------ACCAAACAGGGCCTTACATCC	3671
52_PHR_non-APR	------TGGCCCATTGTCTCTGAATTTCA---	4243
88_PHR_non-APR	AAATCGTGATTTCACATTTCTCAATTTCATTGTTTATTATTTACATTTTGCTGAAAAAAACATT-TATTA	3880
101_PHR_non-APR	AAATCGTGATTTCACATTTCTCAATTTCATTGTTTATTATTTACATTTTGCTGAAAAAAACATT-TATTA	3901
105_PHR_non-APR	AAATCGTGATTTCACATTTCTCAATTTCATTGTTTATTATTTACATTTTGCTGAAAAAAACATT-TATTA	3900
150_PHR_non-APR	AAATCGTGATTTCACATTTCTCAATTTCATTGTTTATTATTTACATTTTGCTGAAAAAAACATT-TATTA	3842
150_PHR_non-APR2	AAATCGTGATTTCACATTTCTCAATTTCATTGTTTATTATTTACATTTTGCTGAAAAAAACATT-TATTA	3842

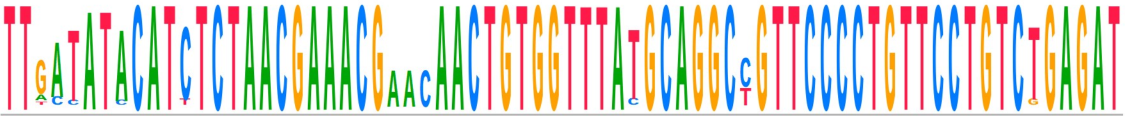

**Consensus**	TTNATATACATCTCTAACGAAACG---AACTGTGGTTTATGCAGGCCGTTCCCCTGTTCCTGTCTGAGAT	
	5120 5130 5140 5150 5160 5170 5180	
Lr67(sus)	TTACTATACATTTCTAACGAAACGAACAACTGTGGT----GCAGGCCGTTCCCCTGTTCCTGTCGGAGAT	3819
Lr67(res)	TTACTATACATTTCTAACGAAACGAACAACTGTGGT----GCAGGCCGTTCCCCTGTTCCTGTCGGAGAT	3819
ScLr67_1 (Lo7)	TTGATATACATCTCTAACGAAACG---AACTGTGGTTTACGCAGGCCGTTCCCCTGTTCCTGTCTGAGAT	4089
118_Danko_APR	TTGATATACATCTCTAACGAAACG---AACTGTGGTTTATGCAGGCCGTTCCCCTGTTCCTGTCTGAGAT	3970
119_Danko_APR	TT------CATCTCTAACGAAACG---AACTGTGGTTTATGCAGGCTGTTCCCCTGTTCCTGTCTGAGAT	3754
119_Danko_APR2	TT------CATCTCTAACGAAACG---AACTGTGGTTTATGCAGGCTGTTCCCCTGTTCCTGTCTGAGAT	3754
120_Danko_APR	TT------CATCTCTAACGAAACG---AACTGTGGTTTATGCAGGCTGTTCCCCTGTTCCTGTCTGAGAT	3756
138_Danko_APR	TT------CATCTCTAACGAAACG---AACTGTGGTTTATGCAGGCTGTTCCCCTGTTCCTGTCTGAGAT	3752
153_Danko_APR	TTGATATACATCTCTAACGAAACG---AACTGTGGTTTATGCAGGCCGTTCCCCTGTTCCTGTCTGAGAT	3964
157_Danko_APR	TT------CATCTCTAACGAAACG---AACTGTGGTTTATGCAGGCTGTTCCCCTGTTCCTGTCTGAGAT	3754
160_Danko_APR	TT------CATCTCTAACGAAACG---AACTGTGGTTTATGCAGGCTGTTCCCCTGTTCCTGTCTGAGAT	3744
71_PHR_APR	TT------CATCTCTAACGAAACG---AACTGTGGTTTATGCAGGCTGTTCCCCTGTTCCTGTCTGAGAT	3823
149_PHR_APR	TTGATATACATCTCTAACGAAACG---AACTGTGGTTTATGCAGGCCGTTCCCCTGTTCCTGTCTGAGAT	3969
59_Danko_non-APR	TT------CATCTCTAACGAAACG---AACTGTGGTTTATGCAGGCTGTTCCCCTGTTCCTGTCTGAGAT	3775
61_Danko_non-APR	TTGATATACATCTCTAACGAAACG---AACTGTGGTTTACGCAGGCCGTTCCCCTGTTCCTGTCTGAGAT	4041
123_Danko_non-APR	TTGATATACATCTCTAACGAAACG---AACTGTGGTTTATGCAGGCCGTTCCCCTGTTCCTGTCTGAGAT	3919
129_Danko_non-APR	TTGATATACATCTCTAACGAAACG---AACTGTGGTTTATGCAGGCCGTTCCCCTGTTCCTGTCTGAGAT	3966
37_PHR_non-APR	TT------CATCTCTAACGAAACG---AACTGTGGTTTATGCAGGCTGTTCCCCTGTTCCTGTCTGAGAT	3732
52_PHR_non-APR	TTTACATCCATCTCTAACGAAACG---AACTGTGGTTTATGCAGGCCGTTCCCCTGTTCCTGTCTGAGAT	4310
88_PHR_non-APR	TTGATATACATCTCTAACGAAACG---AACTGTGGTTTATGCAGGCCGTTCCCCTGTTCCTGTCTGAGAT	3947
101_PHR_non-APR	TTGATATACATCTCTAACGAAACG---AACTGTGGTTTATGCAGGCCGTTCCCCTGTTCCTGTCTGAGAT	3968
105_PHR_non-APR	TTGATATACATCTCTAACGAAACG---AACTGTGGTTTATGCAGGCCGTTCCCCTGTTCCTGTCTGAGAT	3967
150_PHR_non-APR	TTGATATACATCTCTAACGAAACG---AACTGTGGTTTATGCAGGCCGTTCCCCTGTTCCTGTCTGAGAT	3909
150_PHR_non-APR2	TTGATATACATCTCTAACGAAACG---AACTGTGGTTTATGCAGGCCGTTCCCCTGTTCCTGTCTGAGAT	3909

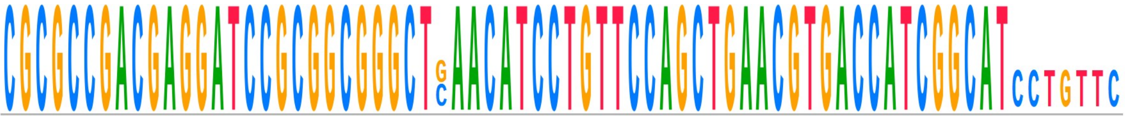

```
Consensus            CGCGCCGACGAGGATCCGCGGCGGGCTNAACATCCTGTTCCAGCTGAACGTGACCATCGGCAT-------
                               5190      5200      5210      5220      5230      5240      5250
Lr67(sus)            CGCGCCGACGAGGATCCGCGGCGGGCTCAACATCCTGTTCCAGCTGAACGTGACCATCGGCAT-------  3882
Lr67(res)            CGCGCCGACGAGGATCCGCGGCGGGCTCAACATCCTGTTCCAGCTGAACGTGACCATCGGCAT-------  3882
ScLr67_1 (Lo7)       CGCGCCGACGAGGATCCGCGGCGGGCTGAACATCCTGTTCCAGCTGAACGTGACCATCGGCAT-------  4152
118_Danko_APR        CGCGCCGACGAGGATCCGCGGCGGGCTGAACATCCTGTTCCAGCTGAACGTGACCATCGGCAT-------  4033
119_Danko_APR        CGCGCCGACGAGGATCCGCGGCGGGCTCAACATCCTGTTCCAGCTGAACGTGACCATCGGCATCCTGTTC  3824
119_Danko_APR2       CGCGCCGACGAGGATCCGCGGCGGGCTCAACATCCTGTTCCAGCTGAACGTGACCATCGGCATCCTGTTC  3824
120_Danko_APR        CGCGCCGACGAGGATCCGCGGCGGGCTCAACATCCTGTTCCAGCTGAACGTGACCATCGGCAT-------  3819
138_Danko_APR        CGCGCCGACGAGGATCCGCGGCGGGCTCAACATCCTGTTCCAGCTGAACGTGACCATCGGCAT-------  3815
153_Danko_APR        CGCGCCGACGAGGATCCGCGGCGGGCTGAACATCCTGTTCCAGCTGAACGTGACCATCGGCAT-------  4027
157_Danko_APR        CGCGCCGACGAGGATCCGCGGCGGGCTCAACATCCTGTTCCAGCTGAACGTGACCATCGGCAT-------  3817
160_Danko_APR        CGCGCCGACGAGGATCCGCGGCGGGCTCAACATCCTGTTCCAGCTGAACGTGACCATCGGCAT-------  3807
71_PHR_APR           CGCGCCGACGAGGATCCGCGGCGGGCTCAACATCCTGTTCCAGCTGAACGTGACCATCGGCAT-------  3886
149_PHR_APR          CGCGCCGACGAGGATCCGCGGCGGGCTGAACATCCTGTTCCAGCTGAACGTGACCATCGGCAT-------  4032
59_Danko_non-APR     CGCGCCGACGAGGATCCGCGGCGGGCTCAACATCCTGTTCCAGCTGAACGTGACCATCGGCAT-------  3838
61_Danko_non-APR     CGCGCCGACGAGGATCCGCGGCGGGCTGAACATCCTGTTCCAGCTGAACGTGACCATCGGCAT-------  4104
123_Danko_non-APR    CGCGCCGACGAGGATCCGCGGCGGGCTGAACATCCTGTTCCAGCTGAACGTGACCATCGGCAT-------  3982
129_Danko_non-APR    CGCGCCGACGAGGATCCGCGGCGGGCTGAACATCCTGTTCCAGCTGAACGTGACCATCGGCAT-------  4029
37_PHR_non-APR       CGCGCCGACGAGGATCCGCGGCGGGCTCAACATCCTGTTCCAGCTGAACGTGACCATCGGCAT-------  3795
52_PHR_non-APR       CGCGCCGACGAGGATCCGCGGCGGGCTCAACATCCTGTTCCAGCTGAACGTGACCATCGGCAT-------  4373
88_PHR_non-APR       CGCGCCGACGAGGATCCGCGGCGGGCTGAACATCCTGTTCCAGCTGAACGTGACCATCGGCAT-------  4010
101_PHR_non-APR      CGCGCCGACGAGGATCCGCGGCGGGCTGAACATCCTGTTCCAGCTGAACGTGACCATCGGCAT-------  4031
105_PHR_non-APR      CGCGCCGACGAGGATCCGCGGCGGGCTGAACATCCTGTTCCAGCTGAACGTGACCATCGGCAT-------  4030
150_PHR_non-APR      CGCGCCGACGAGGATCCGCGGCGGGCTGAACATCCTGTTCCAGCTGAACGTGACCATCGGCAT-------  3972
150_PHR_non-APR2     CGCGCCGACGAGGATCCGCGGCGGGCTGAACATCCTGTTCCAGCTGAACGTGACCATCGGCAT-------  3972
```

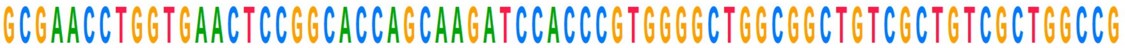

```
Consensus            ----------------------------------------------------------------------
                               5260      5270      5280      5290      5300      5310      5320
Lr67(sus)            ----------------------------------------------------------------------  3882
Lr67(res)            ----------------------------------------------------------------------  3882
ScLr67_1 (Lo7)       ----------------------------------------------------------------------  4152
118_Danko_APR        ----------------------------------------------------------------------  4033
119_Danko_APR        GCGAACCTGGTGAACTCCGGCACCAGCAAGATCCACCCGTGGGGCTGGCGGCTGTCGCTGTCGCTGGCCG  3894
119_Danko_APR2       GCGAACCTGGTGAACTCCGGCACCAGCAAGATCCACCCGTGGGGCTGGCGGCTGTCGCTGTCGCTGGCCG  3894
120_Danko_APR        ----------------------------------------------------------------------  3819
138_Danko_APR        ----------------------------------------------------------------------  3815
153_Danko_APR        ----------------------------------------------------------------------  4027
157_Danko_APR        ----------------------------------------------------------------------  3817
160_Danko_APR        ----------------------------------------------------------------------  3807
71_PHR_APR           ----------------------------------------------------------------------  3886
149_PHR_APR          ----------------------------------------------------------------------  4032
59_Danko_non-APR     ----------------------------------------------------------------------  3838
61_Danko_non-APR     ----------------------------------------------------------------------  4104
123_Danko_non-APR    ----------------------------------------------------------------------  3982
129_Danko_non-APR    ----------------------------------------------------------------------  4029
37_PHR_non-APR       ----------------------------------------------------------------------  3795
52_PHR_non-APR       ----------------------------------------------------------------------  4373
88_PHR_non-APR       ----------------------------------------------------------------------  4010
101_PHR_non-APR      ----------------------------------------------------------------------  4031
105_PHR_non-APR      ----------------------------------------------------------------------  4030
150_PHR_non-APR      ----------------------------------------------------------------------  3972
150_PHR_non-APR2     ----------------------------------------------------------------------  3972
```

	GCATCCCGGCGGCGATGCTCACCCTGGGCGCGCTCTTCGTCACCGACACCCCCAACAGCCTCATCGAGCG	
**Consensus**	--	
	5330 5340 5350 5360 5370 5380 5390	
Lr67(sus)	--	3882
Lr67(res)	--	3882
ScLr67_1 (Lo7)	--	4152
118_Danko_APR	--	4033
119_Danko_APR	GCATCCCGGCGGCGATGCTCACCCTGGGCGCGCTCTTCGTCACCGACACCCCCAACAGCCTCATCGAGCG	3964
119_Danko_APR2	GCATCCCGGCGGCGATGCTCACCCTGGGCGCGCTCTTCGTCACCGACACCCCCAACAGCCTCATCGAGCG	3964
120_Danko_APR	--	3819
138_Danko_APR	--	3815
153_Danko_APR	--	4027
157_Danko_APR	--	3817
160_Danko_APR	--	3807
71_PHR_APR	--	3886
149_PHR_APR	--	4032
59_Danko_non-APR	--	3838
61_Danko_non-APR	--	4104
123_Danko_non-APR	--	3982
129_Danko_non-APR	--	4029
37_PHR_non-APR	--	3795
52_PHR_non-APR	--	4373
88_PHR_non-APR	--	4010
101_PHR_non-APR	--	4031
105_PHR_non-APR	--	4030
150_PHR_non-APR	--	3972
150_PHR_non-APR2	--	3972

	CGGCCACCTGGAGGAGGGCAAGGCGGTGCTGAAGCGGATCCGCGGCACCGACAACGTGCCTGTTCGCGAA	
**Consensus**	--CCTGTTCGCGAA	
	5400 5410 5420 5430 5440 5450 5460	
Lr67(sus)	--CCTGTTCGCGAA	3894
Lr67(res)	--CCTGTTCGCGAA	3894
ScLr67_1 (Lo7)	--CCTGTTCGCGAA	4164
118_Danko_APR	--CCTGTTCGCGAA	4045
119_Danko_APR	CGGCCACCTGGAGGAGGGCAAGGCGGTGCTGAAGCGGATCCGCGGCACCGACAACGTGCCTGTTCGCGAA	4034
119_Danko_APR2	CGGCCACCTGGAGGAGGGCAAGGCGGTGCTGAAGCGGATCCGCGGCACCGACAACGTGCCTGTTCGCGAA	4034
120_Danko_APR	--CCTGTTCGCGAA	3831
138_Danko_APR	--CCTGTTCGCGAA	3827
153_Danko_APR	--CCTGTTCGCGAA	4039
157_Danko_APR	--CCTGTTCGCGAA	3829
160_Danko_APR	--CCTGTTCGCGAA	3819
71_PHR_APR	--CCTGTTCGCGAA	3898
149_PHR_APR	--CCTGTTCGCGAA	4044
59_Danko_non-APR	--CCTGTTCGCGAA	3850
61_Danko_non-APR	--CCTGTTCGCGAA	4116
123_Danko_non-APR	--CCTGTTCGCGAA	3994
129_Danko_non-APR	--CCTGTTCGCGAA	4041
37_PHR_non-APR	--CCTGTTCGCGAA	3807
52_PHR_non-APR	--CCTGTTCGCGAA	4385
88_PHR_non-APR	--CCTGTTCGCGAA	4022
101_PHR_non-APR	--CCTGTTCGCGAA	4043
105_PHR_non-APR	--CCTGTTCGCGAA	4042
150_PHR_non-APR	--CCTGTTCGCGAA	3984
150_PHR_non-APR2	--CCTGTTCGCGAA	3984

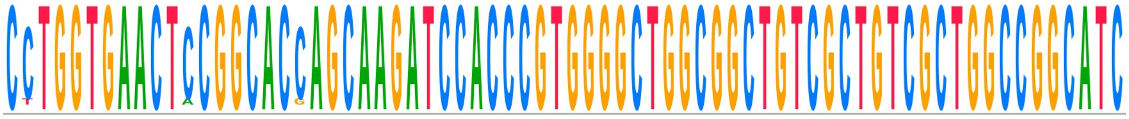

	Sequence	
**Consensus**	CCTGGTGAACTCCGGCACCAGCAAGATCCACCCGTGGGGCTGGCGGCTGTCGCTGTCGCTGGCCGGCATC	
	5470 5480 5490 5500 5510 5520 5530	
Lr67(sus)	CCTGGTGAACTACGGCACGAGCAAGATCCACCCGTGGGGCTGGCGGCTGTCGCTGTCGCTGGCCGGCATC	3964
Lr67(res)	CCTGGTGAACTACGGCACGAGCAAGATCCACCCGTGGGGCTGGCGGCTGTCGCTGTCGCTGGCCGGCATC	3964
ScLr67_1 (Lo7)	CTTGGTGAACTCCGGCACCAGCAAGATCCACCCGTGGGGCTGGCGGCTGTCGCTGTCGCTGGCCGGCATC	4234
118_Danko_APR	CCTGGTGAACTCCGGCACCAGCAAGATCCACCCGTGGGGCTGGCGGCTGTCGCTGTCGCTGGCCGGCATC	4115
119_Danko_APR	CCTGGTGAACTCCGGCACCAGCAAGATCCACCCGTGGGGCTGGCGGCTGTCGCTGTCGCTGGCCGGCATC	4104
119_Danko_APR2	CCTGGTGAACTCCGGCACCAGCAAGATCCACCCGTGGGGCTGGCGGCTGTCGCTGTCGCTGGCCGGCATC	4104
120_Danko_APR	CCTGGTGAACTCCGGCACCAGCAAGATCCACCCGTGGGGCTGGCGGCTGTCGCTGTCGCTGGCCGGCATC	3901
138_Danko_APR	CCTGGTGAACTCCGGCACCAGCAAGATCCACCCGTGGGGCTGGCGGCTGTCGCTGTCGCTGGCCGGCATC	3897
153_Danko_APR	CCTGGTGAACTCCGGCACCAGCAAGATCCACCCGTGGGGCTGGCGGCTGTCGCTGTCGCTGGCCGGCATC	4109
157_Danko_APR	CCTGGTGAACTCCGGCACCAGCAAGATCCACCCGTGGGGCTGGCGGCTGTCGCTGTCGCTGGCCGGCATC	3899
160_Danko_APR	CCTGGTGAACTCCGGCACCAGCAAGATCCACCCGTGGGGCTGGCGGCTGTCGCTGTCGCTGGCCGGCATC	3889
71_PHR_APR	CCTGGTGAACTCCGGCACCAGCAAGATCCACCCGTGGGGCTGGCGGCTGTCGCTGTCGCTGGCCGGCATC	3968
149_PHR_APR	CCTGGTGAACTCCGGCACCAGCAAGATCCACCCGTGGGGCTGGCGGCTGTCGCTGTCGCTGGCCGGCATC	4114
59_Danko_non-APR	CCTGGTGAACTCCGGCACCAGCAAGATCCACCCGTGGGGCTGGCGGCTGTCGCTGTCGCTGGCCGGCATC	3920
61_Danko_non-APR	CTTGGTGAACTCCGGCACCAGCAAGATCCACCCGTGGGGCTGGCGGCTGTCGCTGTCGCTGGCCGGCATC	4186
123_Danko_non-APR	CCTGGTGAACTCCGGCACCAGCAAGATCCACCCGTGGGGCTGGCGGCTGTCGCTGTCGCTGGCCGGCATC	4064
129_Danko_non-APR	CCTGGTGAACTCCGGCACCAGCAAGATCCACCCGTGGGGCTGGCGGCTGTCGCTGTCGCTGGCCGGCATC	4111
37_PHR_non-APR	CCTGGTGAACTCCGGCACCAGCAAGATCCACCCGTGGGGCTGGCGGCTGTCGCTGTCGCTGGCCGGCATC	3877
52_PHR_non-APR	CCTGGTGAACTCCGGCACCAGCAAGATCCACCCGTGGGGCTGGCGGCTGTCGCTGTCGCTGGCCGGCATC	4455
88_PHR_non-APR	CCTGGTGAACTCCGGCACCAGCAAGATCCACCCGTGGGGCTGGCGGCTGTCGCTGTCGCTGGCCGGCATC	4092
101_PHR_non-APR	CCTGGTGAACTCCGGCACCAGCAAGATCCACCCGTGGGGCTGGCGGCTGTCGCTGTCGCTGGCCGGCATC	4113
105_PHR_non-APR	CCTGGTGAACTCCGGCACCAGCAAGATCCACCCGTGGGGCTGGCGGCTGTCGCTGTCGCTGGCCGGCATC	4112
150_PHR_non-APR	CCTGGTGAACTCCGGCACCAGCAAGATCCACCCGTGGGGCTGGCGGCTGTCGCTGTCGCTGGCCGGCATC	4054
150_PHR_non-APR2	CCTGGTGAACTCCGGCACCAGCAAGATCCACCCGTGGGGCTGGCGGCTGTCGCTGTCGCTGGCCGGCATC	4054

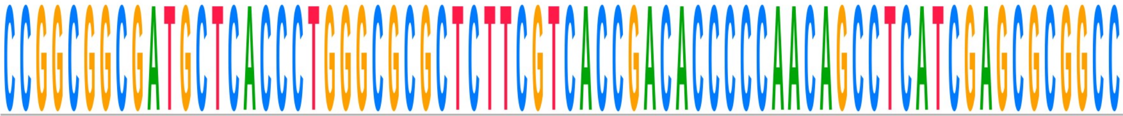

	Sequence	
**Consensus**	CCGGCGGCGATGCTCACCCTGGGCGCGCTCTTCGTCACCGACACCCCCAACAGCCTCATCGAGCGCGGCC	
	5540 5550 5560 5570 5580 5590 5600	
Lr67(sus)	CCGGCGGCGATGCTCACCCTGGGCGCGCTCTTCGTCACCGACACCCCCAACAGCCTCATCGAGCGCGGCC	4034
Lr67(res)	CCGGCGGCGATGCTCACCCTGGGCGCGCTCTTCGTCACCGACACCCCCAACAGCCTCATCGAGCGCGGCC	4034
ScLr67_1 (Lo7)	CCGGCGGCGATGCTCACCCTGGGCGCGCTCTTCGTCACCGACACCCCCAACAGCCTCATCGAGCGCGGCC	4304
118_Danko_APR	CCGGCGGCGATGCTCACCCTGGGCGCGCTCTTCGTCACCGACACCCCCAACAGCCTCATCGAGCGCGGCC	4185
119_Danko_APR	CCGGCGGCGATGCTCACCCTGGGCGCGCTCTTCGTCACCGACACCCCCAACAGCCTCATCGAGCGCGGCC	4174
119_Danko_APR2	CCGGCGGCGATGCTCACCCTGGGCGCGCTCTTCGTCACCGACACCCCCAACAGCCTCATCGAGCGCGGCC	4174
120_Danko_APR	CCGGCGGCGATGCTCACCCTGGGCGCGCTCTTCGTCACCGACACCCCCAACAGCCTCATCGAGCGCGGCC	3971
138_Danko_APR	CCGGCGGCGATGCTCACCCTGGGCGCGCTCTTCGTCACCGACACCCCCAACAGCCTCATCGAGCGCGGCC	3967
153_Danko_APR	CCGGCGGCGATGCTCACCCTGGGCGCGCTCTTCGTCACCGACACCCCCAACAGCCTCATCGAGCGCGGCC	4179
157_Danko_APR	CCGGCGGCGATGCTCACCCTGGGCGCGCTCTTCGTCACCGACACCCCCAACAGCCTCATCGAGCGCGGCC	3969
160_Danko_APR	CCGGCGGCGATGCTCACCCTGGGCGCGCTCTTCGTCACCGACACCCCCAACAGCCTCATCGAGCGCGGCC	3959
71_PHR_APR	CCGGCGGCGATGCTCACCCTGGGCGCGCTCTTCGTCACCGACACCCCCAACAGCCTCATCGAGCGCGGCC	4038
149_PHR_APR	CCGGCGGCGATGCTCACCCTGGGCGCGCTCTTCGTCACCGACACCCCCAACAGCCTCATCGAGCGCGGCC	4184
59_Danko_non-APR	CCGGCGGCGATGCTCACCCTGGGCGCGCTCTTCGTCACCGACACCCCCAACAGCCTCATCGAGCGCGGCC	3990
61_Danko_non-APR	CCGGCGGCGATGCTCACCCTGGGCGCGCTCTTCGTCACCGACACCCCCAACAGCCTCATCGAGCGCGGCC	4256
123_Danko_non-APR	CCGGCGGCGATGCTCACCCTGGGCGCGCTCTTCGTCACCGACACCCCCAACAGCCTCATCGAGCGCGGCC	4134
129_Danko_non-APR	CCGGCGGCGATGCTCACCCTGGGCGCGCTCTTCGTCACCGACACCCCCAACAGCCTCATCGAGCGCGGCC	4181
37_PHR_non-APR	CCGGCGGCGATGCTCACCCTGGGCGCGCTCTTCGTCACCGACACCCCCAACAGCCTCATCGAGCGCGGCC	3947
52_PHR_non-APR	CCGGCGGCGATGCTCACCCTGGGCGCGCTCTTCGTCACCGACACCCCCAACAGCCTCATCGAGCGCGGCC	4525
88_PHR_non-APR	CCGGCGGCGATGCTCACCCTGGGCGCGCTCTTCGTCACCGACACCCCCAACAGCCTCATCGAGCGCGGCC	4162
101_PHR_non-APR	CCGGCGGCGATGCTCACCCTGGGCGCGCTCTTCGTCACCGACACCCCCAACAGCCTCATCGAGCGCGGCC	4183
105_PHR_non-APR	CCGGCGGCGATGCTCACCCTGGGCGCGCTCTTCGTCACCGACACCCCCAACAGCCTCATCGAGCGCGGCC	4182
150_PHR_non-APR	CCGGCGGCGATGCTCACCCTGGGCGCGCTCTTCGTCACCGACACCCCCAACAGCCTCATCGAGCGCGGCC	4124
150_PHR_non-APR2	CCGGCGGCGATGCTCACCCTGGGCGCGCTCTTCGTCACCGACACCCCCAACAGCCTCATCGAGCGCGGCC	4124

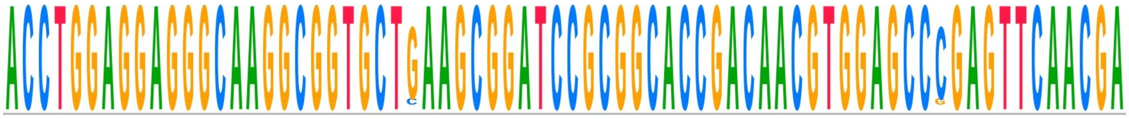

**Consensus**	ACCTGGAGGAGGGCAAGGCGGTGCTGAAGCGGATCCGCGGCACCGACAACGTGGAGCCCGAGTTCAACGA	
	5610 5620 5630 5640 5650 5660 5670	
Lr67(sus)	ACCTGGAGGAGGGCAAGGCGGTGCTCAAGCGGATCCGCGGCACCGACAACGTGGAGCCGGAGTTCAACGA	4104
Lr67(res)	ACCTGGAGGAGGGCAAGGCGGTGCTCAAGCGGATCCGCGGCACCGACAACGTGGAGCCGGAGTTCAACGA	4104
ScLr67_1 (Lo7)	ACCTGGAGGAGGGCAAGGCGGTGCTGAAGCGGATCCGCGGCACCGACAACGTGGAGCCCGAGTTCAACGA	4374
118_Danko_APR	ACCTGGAGGAGGGCAAGGCGGTGCTGAAGCGGATCCGCGGCACCGACAACGTGGAGCCCGAGTTCAACGA	4255
119_Danko_APR	ACCTGGAGGAGGGCAAGGCGGTGCTGAAGCGGATCCGCGGCACCGACAACGTGGAGCCCGAGTTCAACGA	4244
119_Danko_APR2	ACCTGGAGGAGGGCAAGGCGGTGCTGAAGCGGATCCGCGGCACCGACAACGTGGAGCCCGAGTTCAACGA	4244
120_Danko_APR	ACCTGGAGGAGGGCAAGGCGGTGCTGAAGCGGATCCGCGGCACCGACAACGTGGAGCCCGAGTTCAACGA	4041
138_Danko_APR	ACCTGGAGGAGGGCAAGGCGGTGCTGAAGCGGATCCGCGGCACCGACAACGTGGAGCCCGAGTTCAACGA	4037
153_Danko_APR	ACCTGGAGGAGGGCAAGGCGGTGCTGAAGCGGATCCGCGGCACCGACAACGTGGAGCCCGAGTTCAACGA	4249
157_Danko_APR	ACCTGGAGGAGGGCAAGGCGGTGCTGAAGCGGATCCGCGGCACCGACAACGTGGAGCCCGAGTTCAACGA	4039
160_Danko_APR	ACCTGGAGGAGGGCAAGGCGGTGCTGAAGCGGATCCGCGGCACCGACAACGTGGAGCCCGAGTTCAACGA	4029
71_PHR_APR	ACCTGGAGGAGGGCAAGGCGGTGCTGAAGCGGATCCGCGGCACCGACAACGTGGAGCCCGAGTTCAACGA	4108
149_PHR_APR	ACCTGGAGGAGGGCAAGGCGGTGCTGAAGCGGATCCGCGGCACCGACAACGTGGAGCCCGAGTTCAACGA	4254
59_Danko_non-APR	ACCTGGAGGAGGGCAAGGCGGTGCTGAAGCGGATCCGCGGCACCGACAACGTGGAGCCCGAGTTCAACGA	4060
61_Danko_non-APR	ACCTGGAGGAGGGCAAGGCGGTGCTGAAGCGGATCCGCGGCACCGACAACGTGGAGCCCGAGTTCAACGA	4326
123_Danko_non-APR	ACCTGGAGGAGGGCAAGGCGGTGCTGAAGCGGATCCGCGGCACCGACAACGTGGAGCCCGAGTTCAACGA	4204
129_Danko_non-APR	ACCTGGAGGAGGGCAAGGCGGTGCTGAAGCGGATCCGCGGCACCGACAACGTGGAGCCCGAGTTCAACGA	4251
37_PHR_non-APR	ACCTGGAGGAGGGCAAGGCGGTGCTGAAGCGGATCCGCGGCACCGACAACGTGGAGCCCGAGTTCAACGA	4017
52_PHR_non-APR	ACCTGGAGGAGGGCAAGGCGGTGCTGAAGCGGATCCGCGGCACCGACAACGTGGAGCCCGAGTTCAACGA	4595
88_PHR_non-APR	ACCTGGAGGAGGGCAAGGCGGTGCTGAAGCGGATCCGCGGCACCGACAACGTGGAGCCCGAGTTCAACGA	4232
101_PHR_non-APR	ACCTGGAGGAGGGCAAGGCGGTGCTGAAGCGGATCCGCGGCACCGACAACGTGGAGCCCGAGTTCAACGA	4253
105_PHR_non-APR	ACCTGGAGGAGGGCAAGGCGGTGCTGAAGCGGATCCGCGGCACCGACAACGTGGAGCCCGAGTTCAACGA	4252
150_PHR_non-APR	ACCTGGAGGAGGGCAAGGCGGTGCTGAAGCGGATCCGCGGCACCGACAACGTGGAGCCCGAGTTCAACGA	4194
150_PHR_non-APR2	ACCTGGAGGAGGGCAAGGCGGTGCTGAAGCGGATCCGCGGCACCGACAACGTGGAGCCCGAGTTCAACGA	4194

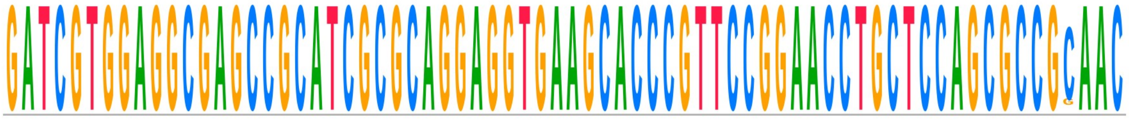

**Consensus**	GATCGTGGAGGCGAGCCGCATCGCGCAGGAGGTGAAGCACCCGTTCCGGAACCTGCTCCAGCGCCGCAAC	
	5680 5690 5700 5710 5720 5730 5740	
Lr67(sus)	GATCGTGGAGGCGAGCCGCATCGCGCAGGAGGTGAAGCACCCGTTCCGGAACCTGCTCCAGCGCCGGAAC	4174
Lr67(res)	GATCGTGGAGGCGAGCCGCATCGCGCAGGAGGTGAAGCACCCGTTCCGGAACCTGCTCCAGCGCCGGAAC	4174
ScLr67_1 (Lo7)	GATCGTGGAGGCGAGCCGCATCGCGCAGGAGGTGAAGCACCCGTTCCGGAACCTGCTCCAGCGCCGCAAC	4444
118_Danko_APR	GATCGTGGAGGCGAGCCGCATCGCGCAGGAGGTGAAGCACCCGTTCCGGAACCTGCTCCAGCGCCGCAAC	4325
119_Danko_APR	GATCGTGGAGGCGAGCCGCATCGCGCAGGAGGTGAAGCACCCGTTCCGGAACCTGCTCCAGCGCCGCAAC	4314
119_Danko_APR2	GATCGTGGAGGCGAGCCGCATCGCGCAGGAGGTGAAGCACCCGTTCCGGAACCTGCTCCAGCGCCGCAAC	4314
120_Danko_APR	GATCGTGGAGGCGAGCCGCATCGCGCAGGAGGTGAAGCACCCGTTCCGGAACCTGCTCCAGCGCCGCAAC	4111
138_Danko_APR	GATCGTGGAGGCGAGCCGCATCGCGCAGGAGGTGAAGCACCCGTTCCGGAACCTGCTCCAGCGCCGCAAC	4107
153_Danko_APR	GATCGTGGAGGCGAGCCGCATCGCGCAGGAGGTGAAGCACCCGTTCCGGAACCTGCTCCAGCGCCGCAAC	4319
157_Danko_APR	GATCGTGGAGGCGAGCCGCATCGCGCAGGAGGTGAAGCACCCGTTCCGGAACCTGCTCCAGCGCCGCAAC	4109
160_Danko_APR	GATCGTGGAGGCGAGCCGCATCGCGCAGGAGGTGAAGCACCCGTTCCGGAACCTGCTCCAGCGCCGCAAC	4099
71_PHR_APR	GATCGTGGAGGCGAGCCGCATCGCGCAGGAGGTGAAGCACCCGTTCCGGAACCTGCTCCAGCGCCGCAAC	4178
149_PHR_APR	GATCGTGGAGGCGAGCCGCATCGCGCAGGAGGTGAAGCACCCGTTCCGGAACCTGCTCCAGCGCCGCAAC	4324
59_Danko_non-APR	GATCGTGGAGGCGAGCCGCATCGCGCAGGAGGTGAAGCACCCGTTCCGGAACCTGCTCCAGCGCCGCAAC	4130
61_Danko_non-APR	GATCGTGGAGGCGAGCCGCATCGCGCAGGAGGTGAAGCACCCGTTCCGGAACCTGCTCCAGCGCCGCAAC	4396
123_Danko_non-APR	GATCGTGGAGGCGAGCCGCATCGCGCAGGAGGTGAAGCACCCGTTCCGGAACCTGCTCCAGCGCCGCAAC	4274
129_Danko_non-APR	GATCGTGGAGGCGAGCCGCATCGCGCAGGAGGTGAAGCACCCGTTCCGGAACCTGCTCCAGCGCCGCAAC	4321
37_PHR_non-APR	GATCGTGGAGGCGAGCCGCATCGCGCAGGAGGTGAAGCACCCGTTCCGGAACCTGCTCCAGCGCCGCAAC	4087
52_PHR_non-APR	GATCGTGGAGGCGAGCCGCATCGCGCAGGAGGTGAAGCACCCGTTCCGGAACCTGCTCCAGCGCCGCAAC	4665
88_PHR_non-APR	GATCGTGGAGGCGAGCCGCATCGCGCAGGAGGTGAAGCACCCGTTCCGGAACCTGCTCCAGCGCCGCAAC	4302
101_PHR_non-APR	GATCGTGGAGGCGAGCCGCATCGCGCAGGAGGTGAAGCACCCGTTCCGGAACCTGCTCCAGCGCCGCAAC	4323
105_PHR_non-APR	GATCGTGGAGGCGAGCCGCATCGCGCAGGAGGTGAAGCACCCGTTCCGGAACCTGCTCCAGCGCCGCAAC	4322
150_PHR_non-APR	GATCGTGGAGGCGAGCCGCATCGCGCAGGAGGTGAAGCACCCGTTCCGGAACCTGCTCCAGCGCCGCAAC	4264
150_PHR_non-APR2	GATCGTGGAGGCGAGCCGCATCGCGCAGGAGGTGAAGCACCCGTTCCGGAACCTGCTCCAGCGCCGCAAC	4264

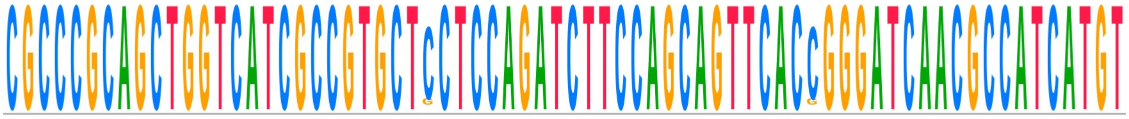

**Consensus**	CGCCCGCAGCTGGTCATCGCCGTGCTCCTCCAGATCTTCCAGCAGTTCACCGGGATCAACGCCATCATGT	
	5750 5760 5770 5780 5790 5800 5810	
Lr67(sus)	CGCCCGCAGCTGGTCATCGCCGTGCTGCTCCAGATCTTCCAGCAGTTCACGGGGATCAACGCCATCATGT	4244
Lr67(res)	CGCCCGCAGCTGGTCATCGCCGTGCTGCTCCAGATCTTCCAGCAGTTCACGGGGATCAACGCCATCATGT	4244
ScLr67_1 (Lo7)	CGCCCGCAGCTGGTCATCGCCGTGCTCCTCCAGATCTTCCAGCAGTTCACCGGGATCAACGCCATCATGT	4514
118_Danko_APR	CGCCCGCAGCTGGTCATCGCCGTGCTCCTCCAGATCTTCCAGCAGTTCACCGGGATCAACGCCATCATGT	4395
119_Danko_APR	CGCCCGCAGCTGGTCATCGCCGTGCTCCTCCAGATCTTCCAGCAGTTCACCGGGATCAACGCCATCATGT	4384
119_Danko_APR2	CGCCCGCAGCTGGTCATCGCCGTGCTCCTCCAGATCTTCCAGCAGTTCACCGGGATCAACGCCATCATGT	4384
120_Danko_APR	CGCCCGCAGCTGGTCATCGCCGTGCTCCTCCAGATCTTCCAGCAGTTCACCGGGATCAACGCCATCATGT	4181
138_Danko_APR	CGCCCGCAGCTGGTCATCGCCGTGCTCCTCCAGATCTTCCAGCAGTTCACCGGGATCAACGCCATCATGT	4177
153_Danko_APR	CGCCCGCAGCTGGTCATCGCCGTGCTCCTCCAGATCTTCCAGCAGTTCACCGGGATCAACGCCATCATGT	4389
157_Danko_APR	CGCCCGCAGCTGGTCATCGCCGTGCTCCTCCAGATCTTCCAGCAGTTCACCGGGATCAACGCCATCATGT	4179
160_Danko_APR	CGCCCGCAGCTGGTCATCGCCGTGCTCCTCCAGATCTTCCAGCAGTTCACCGGGATCAACGCCATCATGT	4169
71_PHR_APR	CGCCCGCAGCTGGTCATCGCCGTGCTCCTCCAGATCTTCCAGCAGTTCACCGGGATCAACGCCATCATGT	4248
149_PHR_APR	CGCCCGCAGCTGGTCATCGCCGTGCTCCTCCAGATCTTCCAGCAGTTCACCGGGATCAACGCCATCATGT	4394
59_Danko_non-APR	CGCCCGCAGCTGGTCATCGCCGTGCTCCTCCAGATCTTCCAGCAGTTCACCGGGATCAACGCCATCATGT	4200
61_Danko_non-APR	CGCCCGCAGCTGGTCATCGCCGTGCTCCTCCAGATCTTCCAGCAGTTCACCGGGATCAACGCCATCATGT	4466
123_Danko_non-APR	CGCCCGCAGCTGGTCATCGCCGTGCTCCTCCAGATCTTCCAGCAGTTCACCGGGATCAACGCCATCATGT	4344
129_Danko_non-APR	CGCCCGCAGCTGGTCATCGCCGTGCTCCTCCAGATCTTCCAGCAGTTCACCGGGATCAACGCCATCATGT	4391
37_PHR_non-APR	CGCCCGCAGCTGGTCATCGCCGTGCTCCTCCAGATCTTCCAGCAGTTCACCGGGATCAACGCCATCATGT	4157
52_PHR_non-APR	CGCCCGCAGCTGGTCATCGCCGTGCTCCTCCAGATCTTCCAGCAGTTCACCGGGATCAACGCCATCATGT	4735
88_PHR_non-APR	CGCCCGCAGCTGGTCATCGCCGTGCTCCTCCAGATCTTCCAGCAGTTCACCGGGATCAACGCCATCATGT	4372
101_PHR_non-APR	CGCCCGCAGCTGGTCATCGCCGTGCTCCTCCAGATCTTCCAGCAGTTCACCGGGATCAACGCCATCATGT	4393
105_PHR_non-APR	CGCCCGCAGCTGGTCATCGCCGTGCTCCTCCAGATCTTCCAGCAGTTCACCGGGATCAACGCCATCATGT	4392
150_PHR_non-APR	CGCCCGCAGCTGGTCATCGCCGTGCTCCTCCAGATCTTCCAGCAGTTCACCGGGATCAACGCCATCATGT	4334
150_PHR_non-APR2	CGCCCGCAGCTGGTCATCGCCGTGCTCCTCCAGATCTTCCAGCAGTTCACCGGGATCAACGCCATCATGT	4334

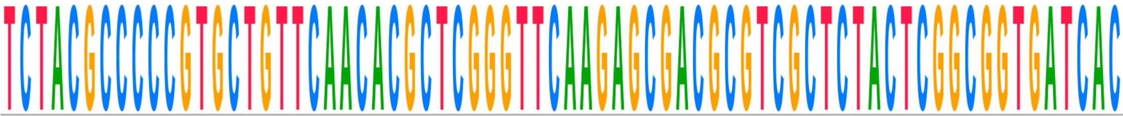

**Consensus**	TCTACGCCCCCGTGCTGTTCAACACGCTCGGGTTCAAGAGCGACGCGTCGCTCTACTCGGCGGTGATCAC	
	5820 5830 5840 5850 5860 5870 5880	
Lr67(sus)	TCTACGCCCCCGTGCTGTTCAACACGCTCGGGTTCAAGAGCGACGCGTCGCTCTACTCGGCGGTGATCAC	4314
Lr67(res)	TCTACGCCCCCGTGCTGTTCAACACGCTCGGGTTCAAGAGCGACGCGTCGCTCTACTCGGCGGTGATCAC	4314
ScLr67_1 (Lo7)	TCTACGCCCCCGTGCTGTTCAACACGCTCGGGTTCAAGAGCGACGCGTCGCTCTACTCGGCGGTGATCAC	4584
118_Danko_APR	TCTACGCCCCCGTGCTGTTCAACACGCTCGGGTTCAAGAGCGACGCGTCGCTCTACTCGGCGGTGATCAC	4465
119_Danko_APR	TCTACGCCCCCGTGCTGTTCAACACGCTCGGGTTCAAGAGCGACGCGTCGCTCTACTCGGCGGTGATCAC	4454
119_Danko_APR2	TCTACGCCCCCGTGCTGTTCAACACGCTCGGGTTCAAGAGCGACGCGTCGCTCTACTCGGCGGTGATCAC	4454
120_Danko_APR	TCTACGCCCCCGTGCTGTTCAACACGCTCGGGTTCAAGAGCGACGCGTCGCTCTACTCGGCGGTGATCAC	4251
138_Danko_APR	TCTACGCCCCCGTGCTGTTCAACACGCTCGGGTTCAAGAGCGACGCGTCGCTCTACTCGGCGGTGATCAC	4247
153_Danko_APR	TCTACGCCCCCGTGCTGTTCAACACGCTCGGGTTCAAGAGCGACGCGTCGCTCTACTCGGCGGTGATCAC	4459
157_Danko_APR	TCTACGCCCCCGTGCTGTTCAACACGCTCGGGTTCAAGAGCGACGCGTCGCTCTACTCGGCGGTGATCAC	4249
160_Danko_APR	TCTACGCCCCCGTGCTGTTCAACACGCTCGGGTTCAAGAGCGACGCGTCGCTCTACTCGGCGGTGATCAC	4239
71_PHR_APR	TCTACGCCCCCGTGCTGTTCAACACGCTCGGGTTCAAGAGCGACGCGTCGCTCTACTCGGCGGTGATCAC	4318
149_PHR_APR	TCTACGCCCCCGTGCTGTTCAACACGCTCGGGTTCAAGAGCGACGCGTCGCTCTACTCGGCGGTGATCAC	4464
59_Danko_non-APR	TCTACGCCCCCGTGCTGTTCAACACGCTCGGGTTCAAGAGCGACGCGTCGCTCTACTCGGCGGTGATCAC	4270
61_Danko_non-APR	TCTACGCCCCCGTGCTGTTCAACACGCTCGGGTTCAAGAGCGACGCGTCGCTCTACTCGGCGGTGATCAC	4536
123_Danko_non-APR	TCTACGCCCCCGTGCTGTTCAACACGCTCGGGTTCAAGAGCGACGCGTCGCTCTACTCGGCGGTGATCAC	4414
129_Danko_non-APR	TCTACGCCCCCGTGCTGTTCAACACGCTCGGGTTCAAGAGCGACGCGTCGCTCTACTCGGCGGTGATCAC	4461
37_PHR_non-APR	TCTACGCCCCCGTGCTGTTCAACACGCTCGGGTTCAAGAGCGACGCGTCGCTCTACTCGGCGGTGATCAC	4227
52_PHR_non-APR	TCTACGCCCCCGTGCTGTTCAACACGCTCGGGTTCAAGAGCGACGCGTCGCTCTACTCGGCGGTGATCAC	4805
88_PHR_non-APR	TCTACGCCCCCGTGCTGTTCAACACGCTCGGGTTCAAGAGCGACGCGTCGCTCTACTCGGCGGTGATCAC	4442
101_PHR_non-APR	TCTACGCCCCCGTGCTGTTCAACACGCTCGGGTTCAAGAGCGACGCGTCGCTCTACTCGGCGGTGATCAC	4463
105_PHR_non-APR	TCTACGCCCCCGTGCTGTTCAACACGCTCGGGTTCAAGAGCGACGCGTCGCTCTACTCGGCGGTGATCAC	4462
150_PHR_non-APR	TCTACGCCCCCGTGCTGTTCAACACGCTCGGGTTCAAGAGCGACGCGTCGCTCTACTCGGCGGTGATCAC	4404
150_PHR_non-APR2	TCTACGCCCCCGTGCTGTTCAACACGCTCGGGTTCAAGAGCGACGCGTCGCTCTACTCGGCGGTGATCAC	4404

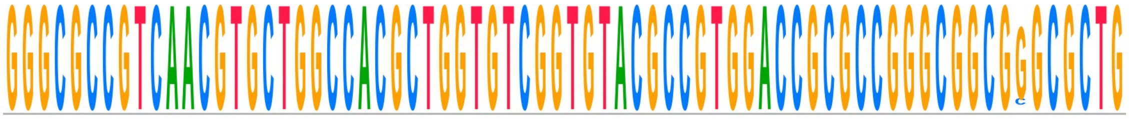

Sequence	Alignment (5881–5950)	End
**Consensus**	GGGCGCCGTCAACGTGCTGGCCACGCTGGTGTCGGTGTACGCCGTGGACCGCGCCGGGCGGCGGGCGCTG	
	5890 5900 5910 5920 5930 5940 5950	
Lr67(sus)	GGGCGCCGTCAACGTGCTGGCCACGCTGGTGTCGGTGTACGCCGTGGACCGCGCCGGGCGGCGCGCGCTG	4384
Lr67(res)	GGGCGCCGTCAACGTGCTGGCCACGCTGGTGTCGGTGTACGCCGTGGACCGCGCCGGGCGGCGCGCGCTG	4384
ScLr67_1 (Lo7)	GGGCGCCGTCAACGTGCTGGCCACGCTGGTGTCGGTGTACGCCGTGGACCGCGCCGGGCGGCGGGCGCTG	4654
118_Danko_APR	GGGCGCCGTCAACGTGCTGGCCACGCTGGTGTCGGTGTACGCCGTGGACCGCGCCGGGCGGCGGGCGCTG	4535
119_Danko_APR	GGGCGCCGTCAACGTGCTGGCCACGCTGGTGTCGGTGTACGCCGTGGACCGCGCCGGGCGGCGGGCGCTG	4524
119_Danko_APR2	GGGCGCCGTCAACGTGCTGGCCACGCTGGTGTCGGTGTACGCCGTGGACCGCGCCGGGCGGCGGGCGCTG	4524
120_Danko_APR	GGGCGCCGTCAACGTGCTGGCCACGCTGGTGTCGGTGTACGCCGTGGACCGCGCCGGGCGGCGGGCGCTG	4321
138_Danko_APR	GGGCGCCGTCAACGTGCTGGCCACGCTGGTGTCGGTGTACGCCGTGGACCGCGCCGGGCGGCGGGCGCTG	4317
153_Danko_APR	GGGCGCCGTCAACGTGCTGGCCACGCTGGTGTCGGTGTACGCCGTGGACCGCGCCGGGCGGCGGGCGCTG	4529
157_Danko_APR	GGGCGCCGTCAACGTGCTGGCCACGCTGGTGTCGGTGTACGCCGTGGACCGCGCCGGGCGGCGGGCGCTG	4319
160_Danko_APR	GGGCGCCGTCAACGTGCTGGCCACGCTGGTGTCGGTGTACGCCGTGGACCGCGCCGGGCGGCGGGCGCTG	4309
71_PHR_APR	GGGCGCCGTCAACGTGCTGGCCACGCTGGTGTCGGTGTACGCCGTGGACCGCGCCGGGCGGCGGGCGCTG	4388
149_PHR_APR	GGGCGCCGTCAACGTGCTGGCCACGCTGGTGTCGGTGTACGCCGTGGACCGCGCCGGGCGGCGGGCGCTG	4534
59_Danko_non-APR	GGGCGCCGTCAACGTGCTGGCCACGCTGGTGTCGGTGTACGCCGTGGACCGCGCCGGGCGGCGGGCGCTG	4340
61_Danko_non-APR	GGGCGCCGTCAACGTGCTGGCCACGCTGGTGTCGGTGTACGCCGTGGACCGCGCCGGGCGGCGGGCGCTG	4606
123_Danko_non-APR	GGGCGCCGTCAACGTGCTGGCCACGCTGGTGTCGGTGTACGCCGTGGACCGCGCCGGGCGGCGGGCGCTG	4484
129_Danko_non-APR	GGGCGCCGTCAACGTGCTGGCCACGCTGGTGTCGGTGTACGCCGTGGACCGCGCCGGGCGGCGGGCGCTG	4531
37_PHR_non-APR	GGGCGCCGTCAACGTGCTGGCCACGCTGGTGTCGGTGTACGCCGTGGACCGCGCCGGGCGGCGGGCGCTG	4297
52_PHR_non-APR	GGGCGCCGTCAACGTGCTGGCCACGCTGGTGTCGGTGTACGCCGTGGACCGCGCCGGGCGGCGGGCGCTG	4875
88_PHR_non-APR	GGGCGCCGTCAACGTGCTGGCCACGCTGGTGTCGGTGTACGCCGTGGACCGCGCCGGGCGGCGGGCGCTG	4512
101_PHR_non-APR	GGGCGCCGTCAACGTGCTGGCCACGCTGGTGTCGGTGTACGCCGTGGACCGCGCCGGGCGGCGGGCGCTG	4533
105_PHR_non-APR	GGGCGCCGTCAACGTGCTGGCCACGCTGGTGTCGGTGTACGCCGTGGACCGCGCCGGGCGGCGGGCGCTG	4532
150_PHR_non-APR	GGGCGCCGTCAACGTGCTGGCCACGCTGGTGTCGGTGTACGCCGTGGACCGCGCCGGGCGGCGGGCGCTG	4474
150_PHR_non-APR2	GGGCGCCGTCAACGTGCTGGCCACGCTGGTGTCGGTGTACGCCGTGGACCGCGCCGGGCGGCGGGCGCTG	4474

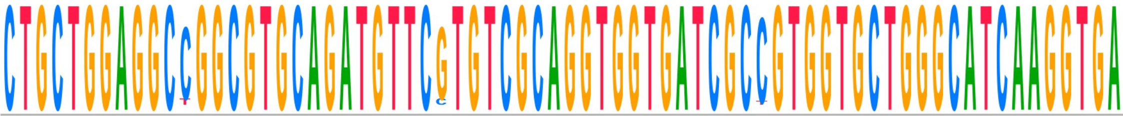

Sequence	Alignment (5951–6020)	End
**Consensus**	CTGCTGGAGGCCGGCGTGCAGATGTTCGTGTCGCAGGTGGTGATCGCCGTGGTGCTGGGCATCAAGGTGA	
	5960 5970 5980 5990 6000 6010 6020	
Lr67(sus)	CTGCTGGAGGCTGGCGTGCAGATGTTCCTGTCGCAGGTGGTGATCGCCGTGGTGCTGGGCATCAAGGTGA	4454
Lr67(res)	CTGCTGGAGGCTGGCGTGCAGATGTTCCTGTCGCAGGTGGTGATCGCCGTGGTGCTGGGCATCAAGGTGA	4454
ScLr67_1 (Lo7)	CTGCTGGAGGCCGGCGTGCAGATGTTCGTGTCGCAGGTGGTGATCGCCGTGGTGCTGGGCATCAAGGTGA	4724
118_Danko_APR	CTGCTGGAGGCCGGCGTGCAGATGTTCGTGTCGCAGGTGGTGATCGCCGTGGTGCTGGGCATCAAGGTGA	4605
119_Danko_APR	CTGCTGGAGGCCGGCGTGCAGATGTTCGTGTCGCAGGTGGTGATCGCCGTGGTGCTGGGCATCAAGGTGA	4594
119_Danko_APR2	CTGCTGGAGGCCGGCGTGCAGATGTTCGTGTCGCAGGTGGTGATCGCCGTGGTGCTGGGCATCAAGGTGA	4594
120_Danko_APR	CTGCTGGAGGCCGGCGTGCAGATGTTCGTGTCGCAGGTGGTGATCGCCGTGGTGCTGGGCATCAAGGTGA	4391
138_Danko_APR	CTGCTGGAGGCCGGCGTGCAGATGTTCGTGTCGCAGGTGGTGATCGCCGTGGTGCTGGGCATCAAGGTGA	4387
153_Danko_APR	CTGCTGGAGGCCGGCGTGCAGATGTTCGTGTCGCAGGTGGTGATCGCCGTGGTGCTGGGCATCAAGGTGA	4599
157_Danko_APR	CTGCTGGAGGCCGGCGTGCAGATGTTCGTGTCGCAGGTGGTGATCGCCGTGGTGCTGGGCATCAAGGTGA	4389
160_Danko_APR	CTGCTGGAGGCCGGCGTGCAGATGTTCGTGTCGCAGGTGGTGATCGCCGTGGTGCTGGGCATCAAGGTGA	4379
71_PHR_APR	CTGCTGGAGGCCGGCGTGCAGATGTTCGTGTCGCAGGTGGTGATCGCCGTGGTGCTGGGCATCAAGGTGA	4458
149_PHR_APR	CTGCTGGAGGCCGGCGTGCAGATGTTCGTGTCGCAGGTGGTGATCGCCGTGGTGCTGGGCATCAAGGTGA	4604
59_Danko_non-APR	CTGCTGGAGGCCGGCGTGCAGATGTTCGTGTCGCAGGTGGTGATCGCTGTGGTGCTGGGCATCAAGGTGA	4410
61_Danko_non-APR	CTGCTGGAGGCCGGCGTGCAGATGTTCGTGTCGCAGGTGGTGATCGCCGTGGTGCTGGGCATCAAGGTGA	4676
123_Danko_non-APR	CTGCTGGAGGCCGGCGTGCAGATGTTCGTGTCGCAGGTGGTGATCGCCGTGGTGCTGGGCATCAAGGTGA	4554
129_Danko_non-APR	CTGCTGGAGGCCGGCGTGCAGATGTTCGTGTCGCAGGTGGTGATCGCCGTGGTGCTGGGCATCAAGGTGA	4601
37_PHR_non-APR	CTGCTGGAGGCCGGCGTGCAGATGTTCGTGTCGCAGGTGGTGATCGCCGTGGTGCTGGGCATCAAGGTGA	4367
52_PHR_non-APR	CTGCTGGAGGCCGGCGTGCAGATGTTCGTGTCGCAGGTGGTGATCGCCGTGGTGCTGGGCATCAAGGTGA	4945
88_PHR_non-APR	CTGCTGGAGGCCGGCGTGCAGATGTTCGTGTCGCAGGTGGTGATCGCCGTGGTGCTGGGCATCAAGGTGA	4582
101_PHR_non-APR	CTGCTGGAGGCCGGCGTGCAGATGTTCGTGTCGCAGGTGGTGATCGCCGTGGTGCTGGGCATCAAGGTGA	4603
105_PHR_non-APR	CTGCTGGAGGCCGGCGTGCAGATGTTCGTGTCGCAGGTGGTGATCGCCGTGGTGCTGGGCATCAAGGTGA	4602
150_PHR_non-APR	CTGCTGGAGGCCGGCGTGCAGATGTTCGTGTCGCAGGTGGTGATCGCCGTGGTGCTGGGCATCAAGGTGA	4544
150_PHR_non-APR2	CTGCTGGAGGCCGGCGTGCAGATGTTCGTGTCGCAGGTGGTGATCGCCGTGGTGCTGGGCATCAAGGTGA	4544

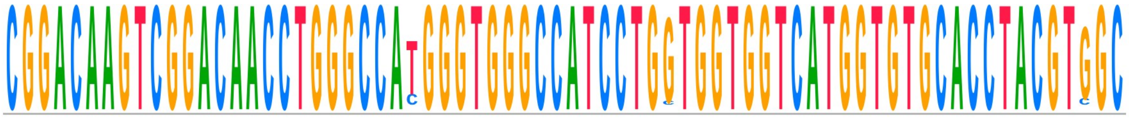

**Consensus**	CGGACAAGTCGGACAACCTGGGCCATGGGTGGGCCATCCTGGTGGTGGTCATGGTGTGCACCTACGTGGC	
	6030 6040 6050 6060 6070 6080 6090	
Lr67(sus)	CGGACAAGTCGGACAACCTGGGCCACGGGTGGGCCATCCTGGTGGTGGTCATGGTGTGCACCTACGTGGC	4524
Lr67(res)	CGGACAAGTCGGACAACCTGGGCCACGGGTGGGCCATCCTGCTGGTGGTCATGGTGTGCACCTACGTGGC	4524
ScLr67_1 (Lo7)	CGGACAAGTCGGACAACCTGGGCCACGGGTGGGCCATCCTGGTGGTGGTCATGGTGTGCACCTACGTCGC	4794
118_Danko_APR	CGGACAAGTCGGACAACCTGGGCCATGGGTGGGCCATCCTGGTGGTGGTCATGGTGTGCACCTACGTGGC	4675
119_Danko_APR	CGGACAAGTCGGACAACCTGGGCCATGGGTGGGCCATCCTGGTGGTGGTCATGGTGTGCACCTACGTGGC	4664
119_Danko_APR2	CGGACAAGTCGGACAACCTGGGCCATGGGTGGGCCATCCTGGTGGTGGTCATGGTGTGCACCTACGTGGC	4664
120_Danko_APR	CGGACAAGTCGGACAACCTGGGCCATGGGTGGGCCATCCTGGTGGTGGTCATGGTGTGCACCTACGTGGC	4461
138_Danko_APR	CGGACAAGTCGGACAACCTGGGCCATGGGTGGGCCATCCTGGTGGTGGTCATGGTGTGCACCTACGTGGC	4457
153_Danko_APR	CGGACAAGTCGGACAACCTGGGCCATGGGTGGGCCATCCTGGTGGTGGTCATGGTGTGCACCTACGTGGC	4669
157_Danko_APR	CGGACAAGTCGGACAACCTGGGCCATGGGTGGGCCATCCTGGTGGTGGTCATGGTGTGCACCTACGTGGC	4459
160_Danko_APR	CGGACAAGTCGGACAACCTGGGCCATGGGTGGGCCATCCTGGTGGTGGTCATGGTGTGCACCTACGTGGC	4449
71_PHR_APR	CGGACAAGTCGGACAACCTGGGCCATGGGTGGGCCATCCTGGTGGTGGTCATGGTGTGCACCTACGTGGC	4528
149_PHR_APR	CGGACAAGTCGGACAACCTGGGCCATGGGTGGGCCATCCTGGTGGTGGTCATGGTGTGCACCTACGTGGC	4674
59_Danko_non-APR	CGGACAAGTCGGACAACCTGGGCCATGGGTGGGCCATCCTGGTGGTGGTCATGGTGTGCACCTACGTGGC	4480
61_Danko_non-APR	CGGACAAGTCGGACAACCTGGGCCACGGGTGGGCCATCCTGGTGGTGGTCATGGTGTGCACCTACGTCGC	4746
123_Danko_non-APR	CGGACAAGTCGGACAACCTGGGCCATGGGTGGGCCATCCTGGTGGTGGTCATGGTGTGCACCTACGTGGC	4624
129_Danko_non-APR	CGGACAAGTCGGACAACCTGGGCCATGGGTGGGCCATCCTGGTGGTGGTCATGGTGTGCACCTACGTGGC	4671
37_PHR_non-APR	CGGACAAGTCGGACAACCTGGGCCATGGGTGGGCCATCCTGGTGGTGGTCATGGTGTGCACCTACGTGGC	4437
52_PHR_non-APR	CGGACAAGTCGGACAACCTGGGCCATGGGTGGGCCATCCTGGTGGTGGTCATGGTGTGCACCTACGTGGC	5015
88_PHR_non-APR	CGGACAAGTCGGACAACCTGGGCCATGGGTGGGCCATCCTGGTGGTGGTCATGGTGTGCACCTACGTGGC	4652
101_PHR_non-APR	CGGACAAGTCGGACAACCTGGGCCATGGGTGGGCCATCCTGGTGGTGGTCATGGTGTGCACCTACGTGGC	4673
105_PHR_non-APR	CGGACAAGTCGGACAACCTGGGCCATGGGTGGGCCATCCTGGTGGTGGTCATGGTGTGCACCTACGTGGC	4672
150_PHR_non-APR	CGGACAAGTCGGACAACCTGGGCCATGGGTGGGCCATCCTGGTGGTGGTCATGGTGTGCACCTACGTGGC	4614
150_PHR_non-APR2	CGGACAAGTCGGACAACCTGGGCCATGGGTGGGCCATCCTGGTGGTGGTCATGGTGTGCACCTACGTGGC	4614

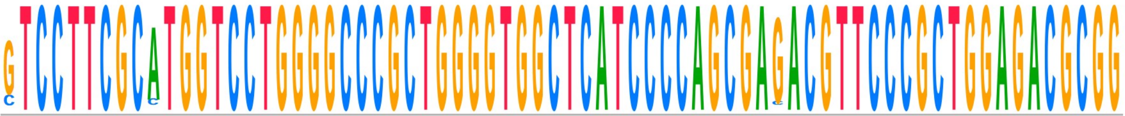

**Consensus**	GTCCTTCGCATGGTCCTGGGGCCCGCTGGGGTGGCTCATCCCAGCGAGACGTTCCCGCTGGAGACGCGG	
	6100 6110 6120 6130 6140 6150 6160	
Lr67(sus)	CTCCTTCGCCTGGTCCTGGGGCCCGCTGGGGTGGCTCATCCCAGCGAGACGTTCCCGCTGGAGACGCGG	4594
Lr67(res)	CTCCTTCGCCTGGTCCTGGGGCCCGCTGGGGTGGCTCATCCCAGCGAGACGTTCCCGCTGGAGACGCGG	4594
ScLr67_1 (Lo7)	CTCCTTCGCATGGTCCTGGGGCCCGCTGGGGTGGCTCATCCCAGCGAGACGTTCCCGCTGGAGACGCGG	4864
118_Danko_APR	GTCCTTCGCATGGTCCTGGGGCCCGCTGGGGTGGCTCATCCCAGCGAGACGTTCCCGCTGGAGACGCGG	4745
119_Danko_APR	GTCCTTCGCATGGTCCTGGGGCCCGCTGGGGTGGCTCATCCCAGCGAGACGTTCCCGCTGGAGACGCGG	4734
119_Danko_APR2	GTCCTTCGCATGGTCCTGGGGCCCGCTGGGGTGGCTCATCCCAGCGAGACGTTCCCGCTGGAGACGCGG	4734
120_Danko_APR	GTCCTTCGCATGGTCCTGGGGCCCGCTGGGGTGGCTCATCCCAGCGAGACGTTCCCGCTGGAGACGCGG	4531
138_Danko_APR	GTCCTTCGCATGGTCCTGGGGCCCGCTGGGGTGGCTCATCCCAGCGAGACGTTCCCGCTGGAGACGCGG	4527
153_Danko_APR	GTCCTTCGCATGGTCCTGGGGCCCGCTGGGGTGGCTCATCCCAGCGACACGTTCCCGCTGGAGACGCGG	4739
157_Danko_APR	GTCCTTCGCATGGTCCTGGGGCCCGCTGGGGTGGCTCATCCCAGCGAGACGTTCCCGCTGGAGACGCGG	4529
160_Danko_APR	GTCCTTCGCATGGTCCTGGGGCCCGCTGGGGTGGCTCATCCCAGCGAGACGTTCCCGCTGGAGACGCGG	4519
71_PHR_APR	GTCCTTCGCATGGTCCTGGGGCCCGCTGGGGTGGCTCATCCCAGCGAGACGTTCCCGCTGGAGACGCGG	4598
149_PHR_APR	GTCCTTCGCATGGTCCTGGGGCCCGCTGGGGTGGCTCATCCCAGCGAGACGTTCCCGCTGGAGACGCGG	4744
59_Danko_non-APR	GTCCTTCGCATGGTCCTGGGGCCCGCTGGGGTGGCTCATCCCAGCGAGACGTTCCCGCTGGAGACGCGG	4550
61_Danko_non-APR	CTCCTTCGCATGGTCCTGGGGCCCGCTGGGGTGGCTCATCCCAGCGAGACGTTCCCGCTGGAGACGCGG	4816
123_Danko_non-APR	GTCCTTCGCATGGTCCTGGGGCCCGCTGGGGTGGCTCATCCCAGCGAGACGTTCCCGCTGGAGACGCGG	4694
129_Danko_non-APR	GTCCTTCGCATGGTCCTGGGGCCCGCTGGGGTGGCTCATCCCAGCGAGACGTTCCCGCTGGAGACGCGG	4741
37_PHR_non-APR	GTCCTTCGCATGGTCCTGGGGCCCGCTGGGGTGGCTCATCCCAGCGAGACGTTCCCGCTGGAGACGCGG	4507
52_PHR_non-APR	GTCCTTCGCATGGTCCTGGGGCCCGCTGGGGTGGCTCATCCCAGCGAGACGTTCCCGCTGGAGACGCGG	5085
88_PHR_non-APR	GTCCTTCGCATGGTCCTGGGGCCCGCTGGGGTGGCTCATCCCAGCGAGACGTTCCCGCTGGAGACGCGG	4722
101_PHR_non-APR	GTCCTTCGCATGGTCCTGGGGCCCGCTGGGGTGGCTCATCCCAGCGAGACGTTCCCGCTGGAGACGCGG	4743
105_PHR_non-APR	GTCCTTCGCATGGTCCTGGGGCCCGCTGGGGTGGCTCATCCCAGCGAGACGTTCCCGCTGGAGACGCGG	4742
150_PHR_non-APR	GTCCTTCGCATGGTCCTGGGGCCCGCTGGGGTGGCTCATCCCAGCGAGACGTTCCCGCTGGAGACGCGG	4684
150_PHR_non-APR2	GTCCTTCGCATGGTCCTGGGGCCCGCTGGGGTGGCTCATCCCAGCGAGACGTTCCCGCTGGAGACGCGG	4684

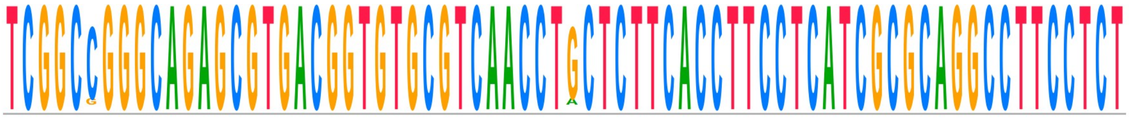

**Consensus**	TCGGCCGGGCAGAGCGTGACGGTGTGCGTCAACCTGCTCTTCACCTTCCTCATCGCGCAGGCCTTCCTCT	
	6170 6180 6190 6200 6210 6220 6230	
Lr67(sus)	TCGGCGGGGCAGAGCGTGACGGTGTGCGTCAACCTGCTCTTCACCTTCCTCATCGCGCAGGCCTTCCTCT	4664
Lr67(res)	TCGGCGGGGCAGAGCGTGACGGTGTGCGTCAACCTGCTCTTCACCTTCCTCATCGCGCAGGCCTTCCTCT	4664
ScLr67_1 (Lo7)	TCGGCCGGGCAGAGCGTGACGGTGTGCGTCAACCTACTCTTCACCTTCCTCATCGCGCAGGCCTTCCTCT	4934
118_Danko_APR	TCGGCCGGGCAGAGCGTGACGGTGTGCGTCAACCTGCTCTTCACCTTCCTCATCGCGCAGGCCTTCCTCT	4815
119_Danko_APR	TCGGCCGGGCAGAGCGTGACGGTGTGCGTCAACCTGCTCTTCACCTTCCTCATCGCGCAGGCCTTCCTCT	4804
119_Danko_APR2	TCGGCCGGGCAGAGCGTGACGGTGTGCGTCAACCTGCTCTTCACCTTCCTCATCGCGCAGGCCTTCCTCT	4804
120_Danko_APR	TCGGCCGGGCAGAGCGTGACGGTGTGCGTCAACCTGCTCTTCACCTTCCTCATCGCGCAGGCCTTCCTCT	4601
138_Danko_APR	TCGGCCGGGCAGAGCGTGACGGTGTGCGTCAACCTGCTCTTCACCTTCCTCATCGCGCAGGCCTTCCTCT	4597
153_Danko_APR	TCGGCCGGGCAGAGCGTGACGGTGTGCGTCAACCTGCTCTTCACCTTCCTCATCGCGCAGGCCTTCCTCT	4809
157_Danko_APR	TCGGCCGGGCAGAGCGTGACGGTGTGCGTCAACCTGCTCTTCACCTTCCTCATCGCGCAGGCCTTCCTCT	4599
160_Danko_APR	TCGGCCGGGCAGAGCGTGACGGTGTGCGTCAACCTGCTCTTCACCTTCCTCATCGCGCAGGCCTTCCTCT	4589
71_PHR_APR	TCGGCCGGGCAGAGCGTGACGGTGTGCGTCAACCTGCTCTTCACCTTCCTCATCGCGCAGGCCTTCCTCT	4668
149_PHR_APR	TCGGCCGGGCAGAGCGTGACGGTGTGCGTCAACCTGCTCTTCACCTTCCTCATCGCGCAGGCCTTCCTCT	4814
59_Danko_non-APR	TCGGCCGGGCAGAGCGTGACGGTGTGCGTCAACCTGCTCTTCACCTTCCTCATCGCGCAGGCCTTCCTCT	4620
61_Danko_non-APR	TCGGCCGGGCAGAGCGTGACGGTGTGCGTCAACCTACTCTTCACCTTCCTCATCGCGCAGGCCTTCCTCT	4886
123_Danko_non-APR	TCGGCCGGGCAGAGCGTGACGGTGTGCGTCAACCTGCTCTTCACCTTCCTCATCGCGCAGGCCTTCCTCT	4764
129_Danko_non-APR	TCGGCCGGGCAGAGCGTGACGGTGTGCGTCAACCTGCTCTTCACCTTCCTCATCGCGCAGGCCTTCCTCT	4811
37_PHR_non-APR	TCGGCCGGGCAGAGCGTGACGGTGTGCGTCAACCTGCTCTTCACCTTCCTCATCGCGCAGGCCTTCCTCT	4577
52_PHR_non-APR	TCGGCCGGGCAGAGCGTGACGGTGTGCGTCAACCTGCTCTTCACCTTCCTCATCGCGCAGGCCTTCCTCT	5155
88_PHR_non-APR	TCGGCCGGGCAGAGCGTGACGGTGTGCGTCAACCTGCTCTTCACCTTCCTCATCGCGCAGGCCTTCCTCT	4792
101_PHR_non-APR	TCGGCCGGGCAGAGCGTGACGGTGTGCGTCAACCTGCTCTTCACCTTCCTCATCGCGCAGGCCTTCCTCT	4813
105_PHR_non-APR	TCGGCCGGGCAGAGCGTGACGGTGTGCGTCAACCTGCTCTTCACCTTCCTCATCGCGCAGGCCTTCCTCT	4812
150_PHR_non-APR	TCGGCCGGGCAGAGCGTGACGGTGTGCGTCAACCTGCTCTTCACCTTCCTCATCGCGCAGGCCTTCCTCT	4754
150_PHR_non-APR2	TCGGCCGGGCAGAGCGTGACGGTGTGCGTCAACCTGCTCTTCACCTTCCTCATCGCGCAGGCCTTCCTCT	4754

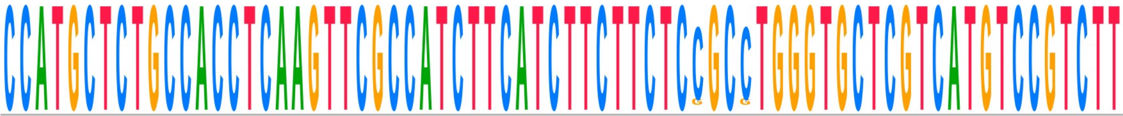

**Consensus**	CCATGCTCTGCCACCTCAAGTTCGCCATCTTCATCTTCTTCTCCGCCTGGGTGCTCGTCATGTCCGTCTT	
	6240 6250 6260 6270 6280 6290 6300	
Lr67(sus)	CCATGCTCTGCCACCTCAAGTTCGCCATCTTCATCTTCTTCTCGGCCTGGGTGCTCGTCATGTCCGTCTT	4734
Lr67(res)	CCATGCTCTGCCACCTCAAGTTCGCCATCTTCATCTTCTTCTCGGCCTGGGTGCTCGTCATGTCCGTCTT	4734
ScLr67_1 (Lo7)	CCATGCTCTGCCACCTCAAGTTCGCCATCTTCATCTTCTTCTCCGCGTGGGTGCTCGTCATGTCCGTCTT	5004
118_Danko_APR	CCATGCTCTGCCACCTCAAGTTCGCCATCTTCATCTTCTTCTCCGCCTGGGTGCTCGTCATGTCCGTCTT	4885
119_Danko_APR	CCATGCTCTGCCACCTCAAGTTCGCCATCTTCATCTTCTTCTCCGCCTGGGTGCTCGTCATGTCCGTCTT	4874
119_Danko_APR2	CCATGCTCTGCCACCTCAAGTTCGCCATCTTCATCTTCTTCTCCGCCTGGGTGCTCGTCATGTCCGTCTT	4874
120_Danko_APR	CCATGCTCTGCCACCTCAAGTTCGCCATCTTCATCTTCTTCTCCGCCTGGGTGCTCGTCATGTCCGTCTT	4671
138_Danko_APR	CCATGCTCTGCCACCTCAAGTTCGCCATCTTCATCTTCTTCTCCGCCTGGGTGCTCGTCATGTCCGTCTT	4667
153_Danko_APR	CCATGCTCTGCCACCTCAAGTTCGCCATCTTCATCTTCTTCTCCGCCTGGGTGCTCGTCATGTCCGTCTT	4879
157_Danko_APR	CCATGCTCTGCCACCTCAAGTTCGCCATCTTCATCTTCTTCTCCGCCTGGGTGCTCGTCATGTCCGTCTT	4669
160_Danko_APR	CCATGCTCTGCCACCTCAAGTTCGCCATCTTCATCTTCTTCTCCGCCTGGGTGCTCGTCATGTCCGTCTT	4659
71_PHR_APR	CCATGCTCTGCCACCTCAAGTTCGCCATCTTCATCTTCTTCTCCGCCTGGGTGCTCGTCATGTCCGTCTT	4738
149_PHR_APR	CCATGCTCTGCCACCTCAAGTTCGCCATCTTCATCTTCTTCTCCGCCTGGGTGCTCGTCATGTCCGTCTT	4884
59_Danko_non-APR	CCATGCTCTGCCACCTCAAGTTCGCCATCTTCATCTTCTTCTCCGCCTGGGTGCTCGTCATGTCCGTCTT	4690
61_Danko_non-APR	CCATGCTCTGCCACCTCAAGTTCGCCATCTTCATCTTCTTCTCCGCGTGGGTGCTCGTCATGTCCGTCTT	4956
123_Danko_non-APR	CCATGCTCTGCCACCTCAAGTTCGCCATCTTCATCTTCTTCTCCGCCTGGGTGCTCGTCATGTCCGTCTT	4834
129_Danko_non-APR	CCATGCTCTGCCACCTCAAGTTCGCCATCTTCATCTTCTTCTCCGCCTGGGTGCTCGTCATGTCCGTCTT	4881
37_PHR_non-APR	CCATGCTCTGCCACCTCAAGTTCGCCATCTTCATCTTCTTCTCCGCCTGGGTGCTCGTCATGTCCGTCTT	4647
52_PHR_non-APR	CCATGCTCTGCCACCTCAAGTTCGCCATCTTCATCTTCTTCTCCGCCTGGGTGCTCGTCATGTCCGTCTT	5225
88_PHR_non-APR	CCATGCTCTGCCACCTCAAGTTCGCCATCTTCATCTTCTTCTCCGCCTGGGTGCTCGTCATGTCCGTCTT	4862
101_PHR_non-APR	CCATGCTCTGCCACCTCAAGTTCGCCATCTTCATCTTCTTCTCCGCCTGGGTGCTCGTCATGTCCGTCTT	4883
105_PHR_non-APR	CCATGCTCTGCCACCTCAAGTTCGCCATCTTCATCTTCTTCTCCGCCTGGGTGCTCGTCATGTCCGTCTT	4882
150_PHR_non-APR	CCATGCTCTGCCACCTCAAGTTCGCCATCTTCATCTTCTTCTCCGCCTGGGTGCTCGTCATGTCCGTCTT	4824
150_PHR_non-APR2	CCATGCTCTGCCACCTCAAGTTCGCCATCTTCATCTTCTTCTCCGCCTGGGTGCTCGTCATGTCCGTCTT	4824

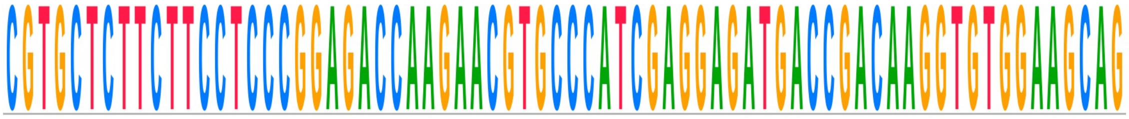

```
Consensus            CGTGCTCTTCTTCCTCCCGGAGACCAAGAACGTGCCCATCGAGGAGATGACCGACAAGGTGTGGAAGCAG
                             6310      6320      6330      6340      6350      6360      6370
Lr67(sus)            CGTGCTCTTCTTCCTCCCGGAGACCAAGAACGTGCCCATCGAGGAGATGACCGACAAGGTGTGGAAGCAG  4804
Lr67(res)            CGTGCTCTTCTTCCTCCCGGAGACCAAGAACGTGCCCATCGAGGAGATGACCGACAAGGTGTGGAAGCAG  4804
ScLr67_1 (Lo7)       CGTGCTCTTCTTCCTCCCGGAGACCAAGAACGTGCCCATCGAGGAGATGACCGACAAGGTGTGGAAGCAG  5074
118_Danko_APR        CGTGCTCTTCTTCCTCCCGGAGACCAAGAACGTGCCCATCGAGGAGATGACCGACAAGGTGTGGAAGCAG  4955
119_Danko_APR        CGTGCTCTTCTTCCTCCCGGAGACCAAGAACGTGCCCATCGAGGAGATGACCGACAAGGTGTGGAAGCAG  4944
119_Danko_APR2       CGTGCTCTTCTTCCTCCCGGAGACCAAGAACGTGCCCATCGAGGAGATGACCGACAAGGTGTGGAAGCAG  4944
120_Danko_APR        CGTGCTCTTCTTCCTCCCGGAGACCAAGAACGTGCCCATCGAGGAGATGACCGACAAGGTGTGGAAGCAG  4741
138_Danko_APR        CGTGCTCTTCTTCCTCCCGGAGACCAAGAACGTGCCCATCGAGGAGATGACCGACAAGGTGTGGAAGCAG  4737
153_Danko_APR        CGTGCTCTTCTTCCTCCCGGAGACCAAGAACGTGCCCATCGAGGAGATGACCGACAAGGTGTGGAAGCAG  4949
157_Danko_APR        CGTGCTCTTCTTCCTCCCGGAGACCAAGAACGTGCCCATCGAGGAGATGACCGACAAGGTGTGGAAGCAG  4739
160_Danko_APR        CGTGCTCTTCTTCCTCCCGGAGACCAAGAACGTGCCCATCGAGGAGATGACCGACAAGGTGTGGAAGCAG  4729
71_PHR_APR           CGTGCTCTTCTTCCTCCCGGAGACCAAGAACGTGCCCATCGAGGAGATGACCGACAAGGTGTGGAAGCAG  4808
149_PHR_APR          CGTGCTCTTCTTCCTCCCGGAGACCAAGAACGTGCCCATCGAGGAGATGACCGACAAGGTGTGGAAGCAG  4954
59_Danko_non-APR     CGTGCTCTTCTTCCTCCCGGAGACCAAGAACGTGCCCATCGAGGAGATGACCGACAAGGTGTGGAAGCAG  4760
61_Danko_non-APR     CGTGCTCTTCTTCCTCCCGGAGACCAAGAACGTGCCCATCGAGGAGATGACCGACAAGGTGTGGAAGCAG  5026
123_Danko_non-APR    CGTGCTCTTCTTCCTCCCGGAGACCAAGAACGTGCCCATCGAGGAGATGACCGACAAGGTGTGGAAGCAG  4904
129_Danko_non-APR    CGTGCTCTTCTTCCTCCCGGAGACCAAGAACGTGCCCATCGAGGAGATGACCGACAAGGTGTGGAAGCAG  4951
37_PHR_non-APR       CGTGCTCTTCTTCCTCCCGGAGACCAAGAACGTGCCCATCGAGGAGATGACCGACAAGGTGTGGAAGCAG  4717
52_PHR_non-APR       CGTGCTCTTCTTCCTCCCGGAGACCAAGAACGTGCCCATCGAGGAGATGACCGACAAGGTGTGGAAGCAG  5295
88_PHR_non-APR       CGTGCTCTTCTTCCTCCCGGAGACCAAGAACGTGCCCATCGAGGAGATGACCGACAAGGTGTGGAAGCAG  4932
101_PHR_non-APR      CGTGCTCTTCTTCCTCCCGGAGACCAAGAACGTGCCCATCGAGGAGATGACCGACAAGGTGTGGAAGCAG  4953
105_PHR_non-APR      CGTGCTCTTCTTCCTCCCGGAGACCAAGAACGTGCCCATCGAGGAGATGACCGACAAGGTGTGGAAGCAG  4952
150_PHR_non-APR      CGTGCTCTTCTTCCTCCCGGAGACCAAGAACGTGCCCATCGAGGAGATGACCGACAAGGTGTGGAAGCAG  4894
150_PHR_non-APR2     CGTGCTCTTCTTCCTCCCGGAGACCAAGAACGTGCCCATCGAGGAGATGACCGACAAGGTGTGGAAGCAG  4894
```

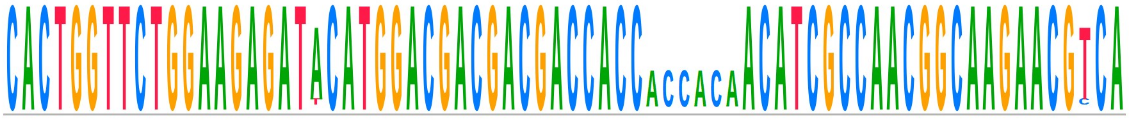

```
Consensus            CACTGGTTCTGGAAGAGATACATGGACGACGACGACCACC------ACATCGCCAACGGCAAGAACGTCA
                             6380      6390      6400      6410      6420      6430      6440
Lr67(sus)            CACTGGTTCTGGAAGAGATTCATGGACGACGACGACCACCACCACAACATCGCCAACGGCAAGAACGCCA  4874
Lr67(res)            CACTGGTTCTGGAAGAGATTCATGGACGACGACGACCACCACCACAACATCGCCAACGGCAAGAACGCCA  4874
ScLr67_1 (Lo7)       CACTGGTTCTGGAAGAGATACATGGACGACGACGACCACC------ACATCGCCAACGGCAAGAACGTCA  5138
118_Danko_APR        CACTGGTTCTGGAAGAGATACATGGACGACGACGACCACC------ACATCGCCAACGGCAAGAACGTCA  5019
119_Danko_APR        CACTGGTTCTGGAAGAGATACATGGACGACGACGACCACC------ACATCGCCAACGGCAAGAACGTCA  5008
119_Danko_APR2       CACTGGTTCTGGAAGAGATACATGGACGACGACGACCACC------ACATCGCCAACGGCAAGAACGTCA  5008
120_Danko_APR        CACTGGTTCTGGAAGAGATACATGGACGACGACGACCACC------ACATCGCCAACGGCAAGAACGTCA  4805
138_Danko_APR        CACTGGTTCTGGAAGAGATACATGGACGACGACGACCACC------ACATCGCCAACGGCAAGAACGTCA  4801
153_Danko_APR        CACTGGTTCTGGAAGAGATACATGGACGACGACGACCACC------ACATCGCCAACGGCAAGAACGTCA  5013
157_Danko_APR        CACTGGTTCTGGAAGAGATACATGGACGACGACGACCACC------ACATCGCCAACGGCAAGAACGTCA  4803
160_Danko_APR        CACTGGTTCTGGAAGAGATACATGGACGACGACGACCACC------ACATCGCCAACGGCAAGAACGTCA  4793
71_PHR_APR           CACTGGTTCTGGAAGAGATACATGGACGACGACGACCACC------ACATCGCCAACGGCAAGAACGTCA  4872
149_PHR_APR          CACTGGTTCTGGAAGAGATACATGGACGACGACGACCACC------ACATCGCCAACGGCAAGAACGTCA  5018
59_Danko_non-APR     CACTGGTTCTGGAAGAGATACATGGACGACGACGACCACC------ACATCGCCAACGGCAAGAACGTCA  4824
61_Danko_non-APR     CACTGGTTCTGGAAGAGATACATGGACGACGACGACCACC------ACATCGCCAACGGCAAGAACGTCA  5090
123_Danko_non-APR    CACTGGTTCTGGAAGAGATACATGGACGACGACGACCACC------ACATCGCCAACGGCAAGAACGTCA  4968
129_Danko_non-APR    CACTGGTTCTGGAAGAGATACATGGACGACGACGACCACC------ACATCGCCAACGGCAAGAACGTCA  5015
37_PHR_non-APR       CACTGGTTCTGGAAGAGATACATGGACGACGACGACCACC------ACATCGCCAACGGCAAGAACGTCA  4781
52_PHR_non-APR       CACTGGTTCTGGAAGAGATACATGGACGACGACGACCACC------ACATCGCCAACGGCAAGAACGTCA  5359
88_PHR_non-APR       CACTGGTTCTGGAAGAGATACATGGACGACGACGACCACC------ACATCGCCAACGGCAAGAACGTCA  4996
101_PHR_non-APR      CACTGGTTCTGGAAGAGATACATGGACGACGACGACCACC------ACATCGCCAACGGCAAGAACGTCA  5017
105_PHR_non-APR      CACTGGTTCTGGAAGAGATACATGGACGACGACGACCACC------ACATCGCCAACGGCAAGAACGTCA  5016
150_PHR_non-APR      CACTGGTTCTGGAAGAGATACATGGACGACGACGACCACC------ACATCGCCAACGGCAAGAACGTCA  4958
150_PHR_non-APR2     CACTGGTTCTGGAAGAGATACATGGACGACGACGACCACC------ACATCGCCAACGGCAAGAACGTCA  4958
```

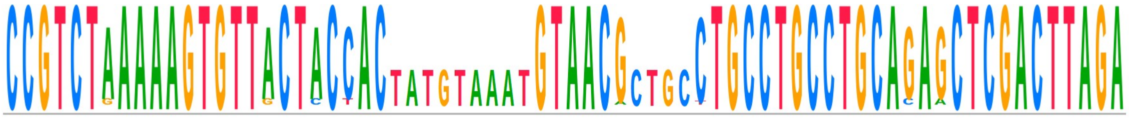

```
Consensus           CCGTCTAAAAAGTGTTACTACCAC---------GTAACG----CTGCCTGCCTGCAGAGCTCGACTTAGA
                                6450      6460      6470      6480      6490      6500      6510
Lr67(sus)           CCGTCTGAAAAGTGTTGCTCCTACTATGTAAATGTAACGCTGCCTGCCTGCCTGCACAACTCGACTTAGA  4944
Lr67(res)           CCGTCTGAAAAGTGTTGCTCCTACTATGTAAATGTAACGCTGCCTGCCTGCCTGCACAACTCGACTTAGA  4944
ScLr67_1 (Lo7)      CCGTCTAAAAAGTGTTACTACCAC---------GTAACG----CTGCCTGCCTGCAGAGCTCGACTTAGA  5195
118_Danko_APR       CCGTCTAAAAAGTGTTACTACCAC---------GTAACG----CTGCCTGCCTGCAGAGCTCGACTTAGA  5076
119_Danko_APR       CCGTCTAAAAAGTGTTACTACCAC---------GTAACG----CTGCCTGCCTGCAGAGCTCGACTTAGA  5065
119_Danko_APR2      CCGTCTAAAAAGTGTTACTACCAC---------GTAACG----CTGCCTGCCTGCAGAGCTCGACTTAGA  5065
120_Danko_APR       CCGTCTAAAAAGTGTTACTACCAC---------GTAACG----CTGCCTGCCTGCAGAGCTCGACTTAGA  4862
138_Danko_APR       CCGTCTAAAAAGTGTTACTACCAC---------GTAACG----CTGCCTGCCTGCAGAGCTCGACTTAGA  4858
153_Danko_APR       CCGTCTAAAAAGTGTTACTACCAC---------GTAACG----CTGCCTGCCTGCAGAGCTCGACTTAGA  5070
157_Danko_APR       CCGTCTAAAAAGTGTTACTACCAC---------GTAACG----CTGCCTGCCTGCAGAGCTCGACTTAGA  4860
160_Danko_APR       CCGTCTAAAAAGTGTTACTACCAC---------GTAACG----CTGCCTGCCTGCAGAGCTCGACTTAGA  4850
71_PHR_APR          CCGTCTAAAAAGTGTTACTACCAC---------GTAACG----CTGCCTGCCTGCAGAGCTCGACTTAGA  4929
149_PHR_APR         CCGTCTAAAAAGTGTTACTACCAC---------GTAACG----CTGCCTGCCTGCAGAGCTCGACTTAGA  5075
59_Danko_non-APR    CCGTCTAAAAAGTGTTACTACCAC---------GTAACA----TTGCCTGCCTGCAGAGCTCGACTTAGA  4881
61_Danko_non-APR    CCGTCTAAAAAGTGTTACTACCAC---------GTAACG----CTGCCTGCCTGCAGAGCTCGACTTAGA  5147
123_Danko_non-APR   CCGTCTAAAAAGTGTTACTACCAC---------GTAACG----CTGCCTGCCTGCAGAGCTCGACTTAGA  5025
129_Danko_non-APR   CCGTCTAAAAAGTGTTACTACCAC---------GTAACG----CTGCCTGCCTGCAGAGCTCGACTTAGA  5072
37_PHR_non-APR      CCGTCTAAAAAGTGTTACTACCAC---------GTAACG----CTGCCTGCCTGCAGAGCTCGACTTAGA  4838
52_PHR_non-APR      CCGTCTAAAAAGTGTTACTACCAC---------GTAACG----CTGCCTGCCTGCAGAGCTCGACTTAGA  5416
88_PHR_non-APR      CCGTCTAAAAAGTGTTACTACCAC---------GTAACG----CTGCCTGCCTGCAGAGCTCGACTTAGA  5053
101_PHR_non-APR     CCGTCTAAAAAGTGTTACTACCAC---------GTAACG----CTGCCTGCCTGCAGAGCTCGACTTAGA  5074
105_PHR_non-APR     CCGTCTAAAAAGTGTTACTACCAC---------GTAACG----CTGCCTGCCTGCAGAGCTCGACTTAGA  5073
150_PHR_non-APR     CCGTCTAAAAAGTGTTACTACCAC---------GTAACG----CTGCCTGCCTGCAGAGCTCGACTTAGA  5015
150_PHR_non-APR2    CCGTCTAAAAAGTGTTACTACCAC---------GTAACG----CTGCCTGCCTGCAGAGCTCGACTTAGA  5015
```

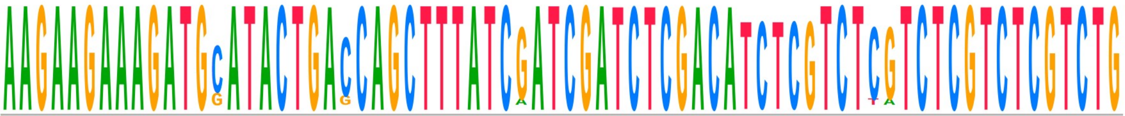

```
Consensus           AAGAAGAAAGATGCATACTGACCAGCTTTATCGATCGATCTCGACA-----TCTCGTCTCGTCTCGTCTG
                                6520      6530      6540      6550      6560      6570      6580
Lr67(sus)           AAGAAGAAAGATGGATACTG---AGCTTTATCAATCGA-------------TCTTATCTCG---------  4989
Lr67(res)           AAGAAGAAAGATGGATACTG---AGCTTTATCAATCGA-------------TCTTATCTCG---------  4989
ScLr67_1 (Lo7)      AAGAAGAAAGATGGATACTGAGCAGCTTTATCGATCGATCTCGACA-----TCTCGTCTCGTCTCGTCTG  5260
118_Danko_APR       AAGAAGAAAGATGCATACTGACCAGCTTTATCGATCGATCTCGACA-----TCTCGTCTCGTCTCGTCTG  5141
119_Danko_APR       AAGAAGAAAGATGCATACTGACCAGCTTTATCGATCGATCTCGACATCTCGTCTCGTCTCGTCTCGTCTG  5135
119_Danko_APR2      AAGAAGAAAGATGCATACTGACCAGCTTTATCGATCGATCTCGACATCTCGTCTCGTCTCGTCTCGTCTG  5135
120_Danko_APR       AAGAAGAAAGATGCATACTGACCAGCTTTATCGATCGATCTCGACATCTCGTCTCGTCTCGTCTCGTCTG  4932
138_Danko_APR       AAGAAGAAAGATGCATACTGACCAGCTTTATCGATCGATCTCGACATCTCGTCTCGTCTCGTCTCGTCTG  4928
153_Danko_APR       AAGAAGAAAGATGCATACTGACCAGCTTTATCGATCGATCTCGACA-----TCTCGTCTCGTCTCGTCTG  5135
157_Danko_APR       AAGAAGAAAGATGCATACTGACCAGCTTTATCGATCGATCTCGACATCTCGTCTCGTCTCGTCTCGTCTG  4930
160_Danko_APR       AAGAAGAAAGATGCATACTGACCAGCTTTATCGATCGATCTCGACATCTCGTCTCGTCTCGTCTCGTCTG  4920
71_PHR_APR          AAGAAGAAAGATGCATACTGACCAGCTTTATCGATCGATCTCGACATCTCGTCTCGTCTCGTCTCGTCTG  4999
149_PHR_APR         AAGAAGAAAGATGCATACTGACCAGCTTTATCGATCGATCTCGACA-----TCTCGTCTCGTCTCGTCTG  5140
59_Danko_non-APR    AAGAAGAAAGATGGATACTGAGCAGCTTTATCGATCGATCTCGACA-----TCTCGTCTCGTCTCGTCTG  4946
61_Danko_non-APR    AAGAAGAAAGATGGATACTGAGCAGCTTTATCGATCGATCTCGACA-----TCTCGTCTCGTCTCGTCTG  5212
123_Danko_non-APR   AAGAAGAAAGATGCATACTGACCAGCTTTATCGATCGATCTCGACA-----TCTCGTCTCGTCTCGTCTG  5090
129_Danko_non-APR   AAGAAGAAAGATGCATACTGACCAGCTTTATCGATCGATCTCGACA-----TCTCGTCTCGTCTCGTCTG  5137
37_PHR_non-APR      AAGAAGAAAGATGCATACTGACCAGCTTTATCGATCGATCTCGACATCTCGTCTCGTCTCGTCTCGTCTG  4908
52_PHR_non-APR      AAGAAGAAAGATGCATACTGACCAGCTTTATCGATCGATCTCGACATCTCGTCTCGTCTCGTCTCGTCTG  5486
88_PHR_non-APR      AAGAAGAAAGATGCATACTGACCAGCTTTATCGATCGATCTCGACA-----TCTCGTCTCGTCTCGTCTG  5118
101_PHR_non-APR     AAGAAGAAAGATGCATACTGACCAGCTTTATCGATCGATCTCGACA-----TCTCGTCTCGTCTCGTCTG  5139
105_PHR_non-APR     AAGAAGAAAGATGCATACTGACCAGCTTTATCGATCGATCTCGACA-----TCTCGTCTCGTCTCGTCTG  5138
150_PHR_non-APR     AAGAAGAAAGATGCATACTGACCAGCTTTATCGATCGATCTCGACA-----TCTCGTCTCGTCTCGTCTG  5080
150_PHR_non-APR2    AAGAAGAAAGATGCATACTGACCAGCTTTATCGATCGATCTCGACA-----TCTCGTCTCGTCTCGTCTG  5080
```

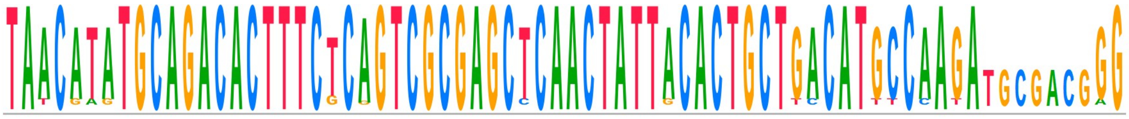

**Consensus**	TAACATATGCAGACACTTTCTCAGTCGCGAGCTCAACTATTACACTGCTGACATGCCAAGA-------GG	
	6590 6600 6610 6620 6630 6640 6650	
Lr67(sus)	-ATCGAGTGCAGACACTTT--CAGTCGCGAGCCCAACTATTGCACTGCTTCCATTTCCATATGCGACGAG	5056
Lr67(res)	-ATCGAGTGCAGACACTTT--CAGTCGCGAGCCCAACTATTGCACTGCTTCCATTTCCATATGCGACGAG	5056
ScLr67_1 (Lo7)	TAACATATGCAGACACTTTCGCAGTCGCGAGCTCAACTATTACACTGCTGACATGCCAAGA-------GG	5323
118_Danko_APR	TAACATATGCAGACACTTTCTCAGTCGCGAGCTCAACTATTACACTGCTGACATGCCAAGA-------GG	5204
119_Danko_APR	TAACATATGCAGACACTTTCTCAGTCGCGAGCTCAACTATTACACTGCTGACATGCCAAGA-------GG	5198
119_Danko_APR2	TAACATATGCAGACACTTTCTCAGTCGCGAGCTCAACTATTACACTGCTGACATGCCAAGA-------GG	5198
120_Danko_APR	TAACATATGCAGACACTTTCTCAGTCGCGAGCTCAACTATTACACTGCTGACATGCCAAGA-------GG	4995
138_Danko_APR	TAACATATGCAGACACTTTCTCAGTCGCGAGCTCAACTATTACACTGCTGACATGCCAAGA-------GG	4991
153_Danko_APR	TAACATATGCAGACACTTTCTCAGTCGCGAGCTCAACTATTACACTGCTGACATGCCAAGA-------GG	5198
157_Danko_APR	TAACATATGCAGACACTTTCTCAGTCGCGAGCTCAACTATTACACTGCTGACATGCCAAGA-------GG	4993
160_Danko_APR	TAACATATGCAGACACTTTCTCAGTCGCGAGCTCAACTATTACACTGCTGACATGCCAAGA-------GG	4983
71_PHR_APR	TAACATATGCAGACACTTTCTCAGTCGCGAGCTCAACTATTACACTGCTGACATGCCAAGA-------GG	5062
149_PHR_APR	TAACATATGCAGACACTTTCTCAGTCGCGAGCTCAACTATTACACTGCTGACATGCCAAGA-------GG	5203
59_Danko_non-APR	TAACATATGCAGACACTTTCGCGGTCGCGAGCTCAACTATTACACTGCTGACATGCCAAGA-------GG	5009
61_Danko_non-APR	TAACATATGCAGACACTTTCGCAGTCGCGAGCTCAACTATTACACTGCTGACATGCCAAGA-------GG	5275
123_Danko_non-APR	TAACATATGCAGACACTTTCTCAGTCGCGAGCTCAACTATTACACTGCTGACATGCCAAGA-------GG	5153
129_Danko_non-APR	TAACATATGCAGACACTTTCTCAGTCGCGAGCTCAACTATTACACTGCTGACATGCCAAGA-------GG	5200
37_PHR_non-APR	TAACATATGCAGACACTTTCTCAGTCGCGAGCTCAACTATTACACTGCTGACATGCCAAGA-------GG	4971
52_PHR_non-APR	TAACATATGCAGACACTTTCTCAGTCGCGAGCTCAACTATTACACTGCTGACATGCCAAGA-------GG	5549
88_PHR_non-APR	TAACATATGCAGACACTTTCTCAGTCGCGAGCTCAACTATTACACTGCTGACATGCCAAGA-------GG	5181
101_PHR_non-APR	TAACATATGCAGACACTTTCTCAGTCGCGAGCTCAACTATTACACTGCTGACATGCCAAGA-------GG	5202
105_PHR_non-APR	TAACATATGCAGACACTTTCTCAGTCGCGAGCTCAACTATTACACTGCTGACATGCCAAGA-------GG	5201
150_PHR_non-APR	TAACATATGCAGACACTTTCTCAGTCGCGAGCTCAACTATTACACTGCTGACATGCCAAGA-------GG	5143
150_PHR_non-APR2	TAACATATGCAGACACTTTCTCAGTCGCGAGCTCAACTATTACACTGCTGACATGCCAAGA-------GG	5143

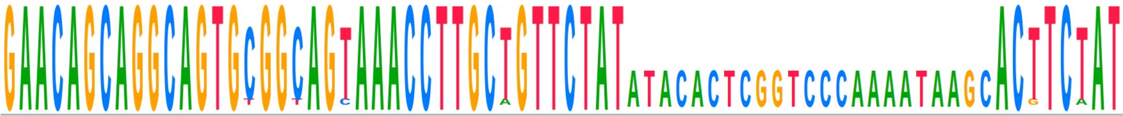

**Consensus**	GAACAGCAGGCAGTGCGGCAGTAAACCTTGCTGTTCTAT-----------------------ACTTCTAT	
	6660 6670 6680 6690 6700 6710 6720	
Lr67(sus)	GAACAGCAGGCAGTGTGGTAGCAAACCTTGCAGTTCTATATACACTCGGTCCCAAAATAAGCACGTCAAT	5126
Lr67(res)	GAACAGCAGGCAGTGTGGTAGCAAACCTTGCAGTTCTATATACACTCGGTCCCAAAATAAGCACGTCAAT	5126
ScLr67_1 (Lo7)	GAACAGCAGGCAGTGCGGCAGTAAACCTTGCTGTTCTAT-----------------------ACTTCTAT	5370
118_Danko_APR	GAACAGCAGGCAGTGCGGCAGTAAACCTTGCTGTTCTAT-----------------------ACTTCTAT	5251
119_Danko_APR	GAACAGCAGGCAGTGCGGCAGTAAACCTTGCTGTTCTAT-----------------------ACTTCTAT	5245
119_Danko_APR2	GAACAGCAGGCAGTGCGGCAGTAAACCTTGCTGTTCTAT-----------------------ACTTCTAT	5245
120_Danko_APR	GAACAGCAGGCAGTGCGGCAGTAAACCTTGCTGTTCTAT-----------------------ACTTCTAT	5042
138_Danko_APR	GAACAGCAGGCAGTGCGGCAGTAAACCTTGCTGTTCTAT-----------------------ACTTCTAT	5038
153_Danko_APR	GAACAGCAGGCAGTGCGGCAGTAAACCTTGCTGTTCTAT-----------------------ACTTCTAT	5245
157_Danko_APR	GAACAGCAGGCAGTGCGGCAGTAAACCTTGCTGTTCTAT-----------------------ACTTCTAT	5040
160_Danko_APR	GAACAGCAGGCAGTGCGGCAGTAAACCTTGCTGTTCTAT-----------------------ACTTCTAT	5030
71_PHR_APR	GAACAGCAGGCAGTGCGGCAGTAAACCTTGCTGTTCTAT-----------------------ACTTCTAT	5109
149_PHR_APR	GAACAGCAGGCAGTGCGGCAGTAAACCTTGCTGTTCTAT-----------------------ACTTCTAT	5250
59_Danko_non-APR	GAACAGCAGGCAGTGCGGCAGTAAACCTTGCTGTTCTAT-----------------------ACTTCTAT	5056
61_Danko_non-APR	GAACAGCAGGCAGTGCGGCAGTAAACCTTGCTGTTCTAT-----------------------ACTTCTAT	5322
123_Danko_non-APR	GAACAGCAGGCAGTGCGGCAGTAAACCTTGCTGTTCTAT-----------------------ACTTCTAT	5200
129_Danko_non-APR	GAACAGCAGGCAGTGCGGCAGTAAACCTTGCTGTTCTAT-----------------------ACTTCTAT	5247
37_PHR_non-APR	GAACAGCAGGCAGTGCGGCAGTAAACCTTGCTGTTCTAT-----------------------ACTTCTAT	5018
52_PHR_non-APR	GAACAGCAGGCAGTGCGGCAGTAAACCTTGCTGTTCTAT-----------------------ACTTCTAT	5596
88_PHR_non-APR	GAACAGCAGGCAGTGCGGCAGTAAACCTTGCTGTTCTAT-----------------------ACTTCTAT	5228
101_PHR_non-APR	GAACAGCAGGCAGTGCGGCAGTAAACCTTGCTGTTCTAT-----------------------ACTTCTAT	5249
105_PHR_non-APR	GAACAGCAGGCAGTGCGGCAGTAAACCTTGCTGTTCTAT-----------------------ACTTCTAT	5248
150_PHR_non-APR	GAACAGCAGGCAGTGCGGCAGTAAACCTTGCTGTTCTAT-----------------------ACTTCTAT	5190
150_PHR_non-APR2	GAACAGCAGGCAGTGCGGCAGTAAACCTTGCTGTTCTAT-----------------------ACTTCTAT	5190

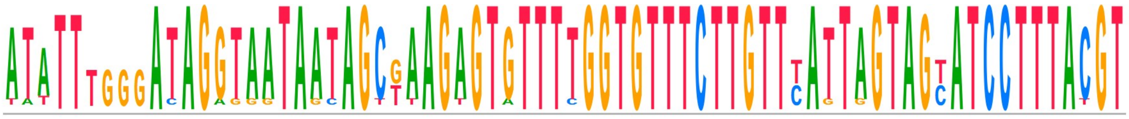

```
Consensus           ATNTT----ATAGGTAATAATAGCGAAGAGTGTTTTGGTGTTTCTTGTTTATTAGTAGTATCCTTTACGT
                            6730      6740      6750      6760      6770      6780      6790

Lr67(sus)           TATTTGGGACAGAGGGTAGCAGTGTAGTGTATTTCGGTGTTTCTTGTTTAGTGGTAGTATCCTTTATGT  5196
Lr67(res)           TATTTGGGACAGAGGGTAGCAGTGTAGTGTATTTCGGTGTTTCTTGTTTAGTGGTAGTATCCTTTATGT  5196
ScLr67_1 (Lo7)      AT-------ATAGGTAATAATAGCTAAGAGTGTTTTGGTGTTTCTTGTTCATTAGTA-TATCCTTTACGT  5432
118_Danko_APR       ATATT----ATAGGTAATAATAGCGAAGAGTGTTTTGGTGTTTCTTGTTTATTAGTAGCATCCTTTACGT  5317
119_Danko_APR       AT-------ATAGGTAATAATAGCTAAGAGTGTTTTGGTGTTTCTTGTTCATTAGTA-TATCCTTTACGT  5307
119_Danko_APR2      AT-------ATAGGTAATAATAGCTAAGAGTGTTTTGGTGTTTCTTGTTCATTAGTA-TATCCTTTACGT  5307
120_Danko_APR       AT-------ATAGGTAATAATAGCTAAGAGTGTTTTGGTGTTTCTTGTTCATTAGTA-TATCCTTTACGT  5104
138_Danko_APR       AT-------ATAGGTAATAATAGCTAAGAGTGTTTTGGTGTTTCTTGTTCATTAGTA-TATCCTTTACGT  5100
153_Danko_APR       ATATT----ATAGGTAATAATAGCGAAGAGTGTTTTGGTGTTTCTTGTTTATTAGTAGCATCCTTTACGT  5311
157_Danko_APR       AT-------ATAGGTAATAATAGCTAAGAGTGTTTTGGTGTTTCTTGTTCATTAGTA-TATCCTTTACGT  5102
160_Danko_APR       AT-------ATAGGTAATAATAGCTAAGAGTGTTTTGGTGTTTCTTGTTCATTAGTA-TATCCTTTACGT  5092
71_PHR_APR          AT-------ATAGGTAATAATAGCTAAGAGTGTTTTGGTGTTTCTTGTTCATTAGTA-TATCCTTTACGT  5171
149_PHR_APR         ATATT----ATAGGTAATAATAGCGAAGAGTGTTTTGGTGTTTCTTGTTTATTAGTAGCATCCTTTACGT  5316
59_Danko_non-APR    AT-------ATAGGTAATAATAGCTAAGAGTGTTTTGGTGTTTCTTGTTCATTAGTA-TATCCTTTACGT  5118
61_Danko_non-APR    AT-------ATAGGTAATAATAGCTAAGAGTGTTTTGGTGTTTCTTGTTCATTAGTA-TATCCTTTACGT  5384
123_Danko_non-APR   ATATT----ATAGGTAATAATAGCGAAGAGTGTTTTGGTGTTTCTTGTTTATTAGTAGCATCCTTTACGT  5266
129_Danko_non-APR   ATATT----ATAGGTAATAATAGCGAAGAGTGTTTTGGTGTTTCTTGTTTATTAGTAGCATCCTTTACGT  5313
37_PHR_non-APR      AT-------ATAGGTAATAATAGCTAAGAGTGTTTTGGTGTTTCTTGTTCATTAGTA-TATCCTTTACGT  5080
52_PHR_non-APR      ATATT----ATAGGTAATAATAGCGAAGAGTGTTTTGGTGTTTCTTGTTTATTAGTAGCATCCTTTACGT  5662
88_PHR_non-APR      ATATT----ATAGGTAATAATAGCGAAGAGTGTTTTGGTGTTTCTTGTTTATTAGTAGCATCCTTTACGT  5294
101_PHR_non-APR     ATATT----ATAGGTAATAATAGCGAAGAGTGTTTTGGTGTTTCTTGTTTATTAGTAGCATCCTTTACGT  5315
105_PHR_non-APR     ATATT----ATAGGTAATAATAGCGAAGAGTGTTTTGGTGTTTCTTGTTTATTAGTAGCATCCTTTACGT  5314
150_PHR_non-APR     ATATT----ATAGGTAATAATAGCGAAGAGTGTTTTGGTGTTTCTTGTTTATTAGTAGCATCCTTTACGT  5256
150_PHR_non-APR2    ATATT----ATAGGTAATAATAGCGAAGAGTGTTTTGGTGTTTCTTGTTTATTAGTAGCATCCTTTACGT  5256
```

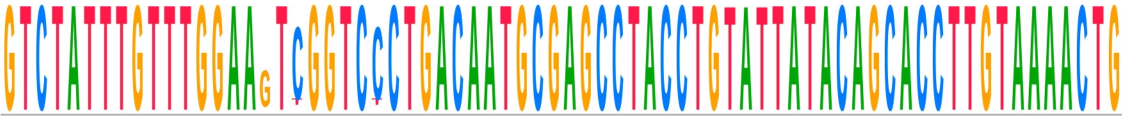

```
Consensus           GTCTATTTGTTTGGAA-TCGGTCCCTGACAATGCGAGCCTACCTGTATTATACAGCACCTTGTAAAACTG
                            6800      6810      6820      6830      6840      6850      6860

Lr67(sus)           GTCTATTTGTTTGGAAGTTGGTCTCTGACAATGCGAGCCTACCTG---TATACAGCACCTTGTAAAACTG  5263
Lr67(res)           GTCTATTTGTTTGGAAGTTGGTCTCTGACAATGCGAGCCTACCTG---TATACAGCACCTTGTAAAACTG  5263
ScLr67_1 (Lo7)      GTCTATTTGTTTGGAA-TCGGTCCCTGACAATGCGAGCCTACCTGTATTATACAGCACCTTGTAAAACTG  5501
118_Danko_APR       GTCTATTTGTTTGGAA-TCGGTCCCTGACAATGCGAGCCTACCTGTATTATACAGCACCTTGTAAAACTG  5386
119_Danko_APR       GTCTATTTGTTTGGAA-TCGGTCCCTGACAATGCGAGCCTACCTGTATTATACAGCACCTTGTAAAACTG  5376
119_Danko_APR2      GTCTATTTGTTTGGAA-TCGGTCCCTGACAATGCGAGCCTACCTGTATTATACAGCACCTTGTAAAACTG  5376
120_Danko_APR       GTCTATTTGTTTGGAA-TCGGTCCCTGACAATGCGAGCCTACCTGTATTATACAGCACCTTGTAAAACTG  5173
138_Danko_APR       GTCTATTTGTTTGGAA-TCGGTCCCTGACAATGCGAGCCTACCTGTATTATACAGCACCTTGTAAAACTG  5169
153_Danko_APR       GTCTATTTGTTTGGAA-TCGGTCCCTGACAATGCGAGCCTACCTGTATTATACAGCACCTTGTAAAACTG  5380
157_Danko_APR       GTCTATTTGTTTGGAA-TCGGTCCCTGACAATGCGAGCCTACCTGTATTATACAGCACCTTGTAAAACTG  5171
160_Danko_APR       GTCTATTTGTTTGGAA-TCGGTCCCTGACAATGCGAGCCTACCTGTATTATACAGCACCTTGTAAAACTG  5161
71_PHR_APR          GTCTATTTGTTTGGAA-TCGGTCCCTGACAATGCGAGCCTACCTGTATTATACAGCACCTTGTAAAACTG  5240
149_PHR_APR         GTCTATTTGTTTGGAA-TCGGTCCCTGACAATGCGAGCCTACCTGTATTATACAGCACCTTGTAAAACTG  5385
59_Danko_non-APR    GTCTATTTGTTTGGAA-TCGGTCCCTGACAATGCGAGCCTACCTGTATTATACAGCACCTTGTAAAACTG  5187
61_Danko_non-APR    GTCTATTTGTTTGGAA-TCGGTCCCTGACAATGCGAGCCTACCTGTATTATACAGCACCTTGTAAAACTG  5453
123_Danko_non-APR   GTCTATTTGTTTGGAA-TCGGTCCCTGACAATGCGAGCCTACCTGTATTATACAGCACCTTGTAAAACTG  5335
129_Danko_non-APR   GTCTATTTGTTTGGAA-TCGGTCCCTGACAATGCGAGCCTACCTGTATTATACAGCACCTTGTAAAACTG  5382
37_PHR_non-APR      GTCTATTTGTTTGGAA-TCGGTCCCTGACAATGCGAGCCTACCTGTATTATACAGCACCTTGTAAAACTG  5149
52_PHR_non-APR      GTCTATTTGTTTGGAA-TCGGTCCCTGACAATGCGAGCCTACCTGTATTATACAGCACCTTGTAAAACTG  5731
88_PHR_non-APR      GTCTATTTGTTTGGAA-TCGGTCCCTGACAATGCGAGCCTACCTGTATTATACAGCACCTTGTAAAACTG  5363
101_PHR_non-APR     GTCTATTTGTTTGGAA-TCGGTCCCTGACAATGCGAGCCTACCTGTATTATACAGCACCTTGTAAAACTG  5384
105_PHR_non-APR     GTCTATTTGTTTGGAA-TCGGTCCCTGACAATGCGAGCCTACCTGTATTATACAGCACCTTGTAAAACTG  5383
150_PHR_non-APR     GTCTATTTGTTTGGAA-TCGGTCCCTGACAATGCGAGCCTACCTGTATTATACAGCACCTTGTAAAACTG  5325
150_PHR_non-APR2    GTCTATTTGTTTGGAA-TCGGTCCCTGACAATGCGAGCCTACCTGTATTATACAGCACCTTGTAAAACTG  5325
```

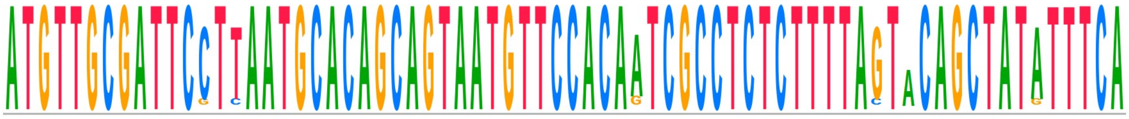

Sequence	6870 … 6930	
**Consensus**	ATGTTGCGATTCCTTAATGCACAGCAGTAATGTTCCACAATCGCCTCTCTTTTAGT-CAGCTATATTTCA	
Lr67(sus)	ATGTTGCGATTCGTCAATGCACAGCAGTAATGTTCCACAATCGCCTCTCTTTTACTACAGCTATG--TCA	5331
Lr67(res)	ATGTTGCGATTCGTCAATGCACAGCAGTAATGTTCCACAATCGCCTCTCTTTTACTACAGCTATG--TCA	5331
ScLr67_1 (Lo7)	ATGTTGCGATTCCTTAATGCACAGCAGTAATGTTCCACAGTCGCCTCTCTTTTAGT-CAGCTATATTTCA	5570
118_Danko_APR	ATGTTGCGATTCCTTAATGCACAGCAGTAATGTTCCACAATCGCCTCTCTTTTAGT-CAGCTATATTTCA	5455
119_Danko_APR	ATGTTGCGATTCCTTAATGCACAGCAGTAATGTTCCACAATCGCCTCTCTTTTAGT-CAGCTATATTTCA	5445
119_Danko_APR2	ATGTTGCGATTCCTTAATGCACAGCAGTAATGTTCCACAATCGCCTCTCTTTTAGT-CAGCTATATTTCA	5445
120_Danko_APR	ATGTTGCGATTCCTTAATGCACAGCAGTAATGTTCCACAATCGCCTCTCTTTTAGT-CAGCTATATTTCA	5242
138_Danko_APR	ATGTTGCGATTCCTTAATGCACAGCAGTAATGTTCCACAATCGCCTCTCTTTTAGT-CAGCTATATTTCA	5238
153_Danko_APR	ATGTTGCGATTCCTTAATGCACAGCAGTAATGTTCCACAATCGCCTCTCTTTTAGT-CAGCTATATTTCA	5449
157_Danko_APR	ATGTTGCGATTCCTTAATGCACAGCAGTAATGTTCCACAATCGCCTCTCTTTTAGT-CAGCTATATTTCA	5240
160_Danko_APR	ATGTTGCGATTCCTTAATGCACAGCAGTAATGTTCCACAATCGCCTCTCTTTTAGT-CAGCTATATTTCA	5230
71_PHR_APR	ATGTTGCGATTCCTTAATGCACAGCAGTAATGTTCCACAATCGCCTCTCTTTTAGT-CAGCTATATTTCA	5309
149_PHR_APR	ATGTTGCGATTCCTTAATGCACAGCAGTAATGTTCCACAATCGCCTCTCTTTTAGT-CAGCTATATTTCA	5454
59_Danko_non-APR	ATGTTGCGATTCCTTAATGCACAGCAGTAATGTTCCACAATCGCCTCTCTTTTAGT-CAGCTATATTTCA	5256
61_Danko_non-APR	ATGTTGCGATTCCTTAATGCACAGCAGTAATGTTCCACAGTCGCCTCTCTTTTAGT-CAGCTATATTTCA	5522
123_Danko_non-APR	ATGTTGCGATTCCTTAATGCACAGCAGTAATGTTCCACAATCGCCTCTCTTTTAGT-CAGCTATATTTCA	5404
129_Danko_non-APR	ATGTTGCGATTCCTTAATGCACAGCAGTAATGTTCCACAATCGCCTCTCTTTTAGT-CAGCTATATTTCA	5451
37_PHR_non-APR	ATGTTGCGATTCCTTAATGCACAGCAGTAATGTTCCACAATCGCCTCTCTTTTAGT-CAGCTATATTTCA	5218
52_PHR_non-APR	ATGTTGCGATTCCTTAATGCACAGCAGTAATGTTCCACAGTCGCCTCTCTTTTAGT-CAGCTATATTTCA	5800
88_PHR_non-APR	ATGTTGCGATTCCTTAATGCACAGCAGTAATGTTCCACAATCGCCTCTCTTTTAGT-CAGCTATATTTCA	5432
101_PHR_non-APR	ATGTTGCGATTCCTTAATGCACAGCAGTAATGTTCCACAATCGCCTCTCTTTTAGT-CAGCTATATTTCA	5453
105_PHR_non-APR	ATGTTGCGATTCCTTAATGCACAGCAGTAATGTTCCACAATCGCCTCTCTTTTAGT-CAGCTATATTTCA	5452
150_PHR_non-APR	ATGTTGCGATTCCTTAATGCACAGCAGTAATGTTCCACAATCGCCTCTCTTTTAGT-CAGCTATATTTCA	5394
150_PHR_non-APR2	ATGTTGCGATTCCTTAATGCACAGCAGTAATGTTCCACAATCGCCTCTCTTTTAGT-CAGCTATATTTCA	5394

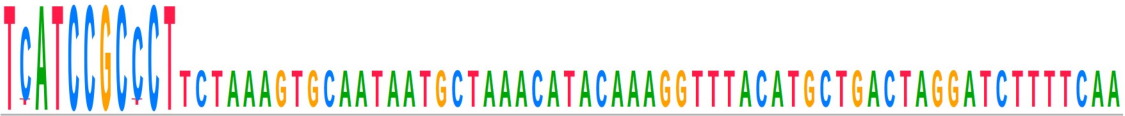

Sequence	6940 … 7000	
**Consensus**	TCATCCGCCCT---	
Lr67(sus)	TTATCCGCTCTTCTAAAGTGCAATAATGCTAAACATACAAAGGTTTACATGCTGACTAGGATCTTTTCAA	5401
Lr67(res)	TTATCCGCTCTTCTAAAGTGCAATAATGCTAAACATACAAAGGTTTACATGCTGACTAGGATCTTTTCAA	5401
ScLr67_1 (Lo7)	TCATCCGCCCT---	5581
118_Danko_APR	TCATCCGCCCT---	5466
119_Danko_APR	TCATCCGCCCT---	5456
119_Danko_APR2	TCATCCGCCCT---	5456
120_Danko_APR	TCATCCGCCCT---	5253
138_Danko_APR	TCATCCGCCCT---	5249
153_Danko_APR	TCATCCGCCCT---	5460
157_Danko_APR	TCATCCGCCCT---	5251
160_Danko_APR	TCATCCGCCCT---	5241
71_PHR_APR	TCATCCGCCCT---	5320
149_PHR_APR	TCATCCGCCCT---	5465
59_Danko_non-APR	TCATCCGCCCT---	5267
61_Danko_non-APR	TCATCCGCCCT---	5533
123_Danko_non-APR	TCATCCGCCCT---	5415
129_Danko_non-APR	TCATCCGCCCT---	5462
37_PHR_non-APR	TCATCCGCCCT---	5229
52_PHR_non-APR	TCATCCGCCCT---	5811
88_PHR_non-APR	TCATCCGCCCT---	5443
101_PHR_non-APR	TCATCCGCCCT---	5464
105_PHR_non-APR	TCATCCGCCCT---	5463
150_PHR_non-APR	TCATCCGCCCT---	5405
150_PHR_non-APR2	TCATCCGCCCT---	5405

```
                   ACTAACTAACCTCCCCCCACCCCCCCTATTCCCCCTGATTTTCATAAGGGTGGGCCCCTTCTACCCCTTA
Consensus          ----------------------------------------------------------------------
                            7010      7020      7030      7040      7050      7060      7070
Lr67(sus)          ACTAACTAACCTCCCCCCACCCCCCCTATTCCCCCTGATTTTCATAAGGGTGGGCCCCTTCTACCCCTTA 5471
Lr67(res)          ACTAACTAACCTCCCCCCACCCCCCCTATTCCCCCTGATTTTCATAAGGGTGGGCCCCTTCTACCCCTTA 5471
ScLr67_1 (Lo7)     ---------------------------------------------------------------------- 5581
118_Danko_APR      ---------------------------------------------------------------------- 5466
119_Danko_APR      ---------------------------------------------------------------------- 5456
119_Danko_APR2     ---------------------------------------------------------------------- 5456
120_Danko_APR      ---------------------------------------------------------------------- 5253
138_Danko_APR      ---------------------------------------------------------------------- 5249
153_Danko_APR      ---------------------------------------------------------------------- 5460
157_Danko_APR      ---------------------------------------------------------------------- 5251
160_Danko_APR      ---------------------------------------------------------------------- 5241
71_PHR_APR         ---------------------------------------------------------------------- 5320
149_PHR_APR        ---------------------------------------------------------------------- 5465
59_Danko_non-APR   ---------------------------------------------------------------------- 5267
61_Danko_non-APR   ---------------------------------------------------------------------- 5533
123_Danko_non-APR  ---------------------------------------------------------------------- 5415
129_Danko_non-APR  ---------------------------------------------------------------------- 5462
37_PHR_non-APR     ---------------------------------------------------------------------- 5229
52_PHR_non-APR     ---------------------------------------------------------------------- 5811
88_PHR_non-APR     ---------------------------------------------------------------------- 5443
101_PHR_non-APR    ---------------------------------------------------------------------- 5464
105_PHR_non-APR    ---------------------------------------------------------------------- 5463
150_PHR_non-APR    ---------------------------------------------------------------------- 5405
150_PHR_non-APR2   ---------------------------------------------------------------------- 5405
```

```
                   ATCTCCTATCAAAACTCACCTTTATGTAAAAGCCTCCTTTTGTACGTGTAGGATTACTCTCTAAAGTGTT
Consensus          -----------------------------------------------------------TCTAAAGTGTT
                            7080      7090      7100      7110      7120      7130      7140
Lr67(sus)          ATCTCCTATCAAAACTCACCTTTATGTAAAAGCCTCCTTTTGTACGTGTAGGATTACTCTCTAAAGTGTT 5541
Lr67(res)          ATCTCCTATCAAAACTCACCTTTATGTAAAAGCCTCCTTTTGTACGTGTAGGATTACTCTCTAAAGTGTT 5541
ScLr67_1 (Lo7)     -----------------------------------------------------------TCTAAAGTGTT 5592
118_Danko_APR      -----------------------------------------------------------TCTAAAGTGTT 5477
119_Danko_APR      -----------------------------------------------------------TCTAAAGTGTT 5467
119_Danko_APR2     -----------------------------------------------------------TCTAAAGTGTT 5467
120_Danko_APR      -----------------------------------------------------------TCTAAAGTGTT 5264
138_Danko_APR      -----------------------------------------------------------TCTAAAGTGTT 5260
153_Danko_APR      -----------------------------------------------------------TCTAAAGTGTT 5471
157_Danko_APR      -----------------------------------------------------------TCTAAAGTGTT 5262
160_Danko_APR      -----------------------------------------------------------TCTAAAGTGTT 5252
71_PHR_APR         -----------------------------------------------------------TCTAAAGTGTT 5331
149_PHR_APR        -----------------------------------------------------------TCTAAAGTGTT 5476
59_Danko_non-APR   -----------------------------------------------------------TCTAAAGTGTT 5278
61_Danko_non-APR   -----------------------------------------------------------TCTAAAGTGTT 5544
123_Danko_non-APR  -----------------------------------------------------------TCTAAAGTGTT 5426
129_Danko_non-APR  -----------------------------------------------------------TCTAAAGTGTT 5473
37_PHR_non-APR     -----------------------------------------------------------TCTAAAGTGTT 5240
52_PHR_non-APR     -----------------------------------------------------------TCTAAAGTGTT 5822
88_PHR_non-APR     -----------------------------------------------------------TCTAAAGTGTT 5454
101_PHR_non-APR    -----------------------------------------------------------TCTAAAGTGTT 5475
105_PHR_non-APR    -----------------------------------------------------------TCTAAAGTGTT 5474
150_PHR_non-APR    -----------------------------------------------------------TCTAAAGTGTT 5416
150_PHR_non-APR2   -----------------------------------------------------------TCTAAAGTGTT 5416
```

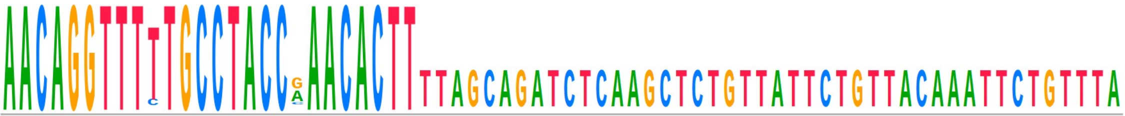

```
Consensus           AACAGGTTTTTGCCTACCNAACACTT--------------------------------------------
                             7150      7160      7170      7180      7190      7200      7210
Lr67(sus)           AACAGGTTTCTGCCTACCCAACACTTTTAGCAGATCTCAAGCTCTGTTATTCTGTTACAAATTCTGTTTA   5611
Lr67(res)           AACAGGTTTCTGCCTACCCAACACTTTTAGCAGATCTCAAGCTCTGTTATTCTGTTACAAATTCTGTTTA   5611
ScLr67_1 (Lo7)      AACAGGTTTTTGCCTACCGAACACTT--------------------------------------------   5618
118_Danko_APR       AACAGGTTTTTGCCTACCAAACAC----------------------------------------------   5501
119_Danko_APR       AACAGGTTTTTGCCTACCGAACACTT--------------------------------------------   5493
119_Danko_APR2      AACAGGTTTTTGCCTACCGAACACTT--------------------------------------------   5493
120_Danko_APR       AACAGGTTTTTGCCTACCGAACACTT--------------------------------------------   5290
138_Danko_APR       AACAGGTTTTTGCCTACCGAACACTT--------------------------------------------   5286
153_Danko_APR       AACAGGTTTTTGCCTACCAAACACTT--------------------------------------------   5497
157_Danko_APR       AACAGGTTTTTGCCTACCGAACACTT--------------------------------------------   5288
160_Danko_APR       AACAGGTTTTTGCCTACCGAACACTT--------------------------------------------   5278
71_PHR_APR          AACAGGTTTTTGCCTACCGAACACTT--------------------------------------------   5357
149_PHR_APR         AACAGGTTTTTGCCTACCAAACACTT--------------------------------------------   5502
59_Danko_non-APR    AACAGGTTTTTGCCTACCAAACACTT--------------------------------------------   5304
61_Danko_non-APR    AACAGGTTTTTGCCTACCGAACACTT--------------------------------------------   5570
123_Danko_non-APR   AACAGGTTTTTGCCTACCAAACACTT--------------------------------------------   5452
129_Danko_non-APR   AACAGGTTTTTGCCTACCAAACACTT--------------------------------------------   5499
37_PHR_non-APR      AACAGGTTTTTGCCTACCGAACACTT--------------------------------------------   5266
52_PHR_non-APR      AACAGGTTTTTGCCTACCGAACACTT--------------------------------------------   5848
88_PHR_non-APR      AACAGGTTTTTGCCTACCAAACACTT--------------------------------------------   5480
101_PHR_non-APR     AACAGGTTTTTGCCTACCAAACACTT--------------------------------------------   5501
105_PHR_non-APR     AACAGGTTTTTGCCTACCAAACACTT--------------------------------------------   5500
150_PHR_non-APR     AACAGGTTTTTGCCTACCAAACACTT--------------------------------------------   5442
150_PHR_non-APR2    AACAGGTTTTTGCCTACCAAACACTT--------------------------------------------   5442
```

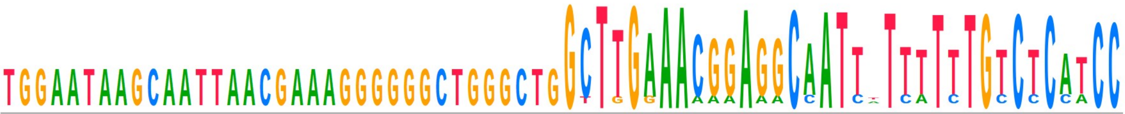

```
Consensus           -----------------------------------GCTTGAAACGGAGGCNATN-TNNNNNNNNN-----
                             7220      7230      7240      7250      7260      7270      7280
Lr67(sus)           TGGAATAAGCAATTAACGAAAGGGGGGCTGGGCTGGTTGGGAAAAAAAACCATCATCATCTGCCCCCACC   5681
Lr67(res)           TGGAATAAGCAATTAACGAAAGGGGGGCTGGGCTGGTTGGGAAAAAAAACCATCATCATCTGCCCCCACC   5681
ScLr67_1 (Lo7)      -----------------------------------GCTTGAAACGGAGGCAATTTTTTTTTGTCTCATCC   5653
118_Danko_APR       ----------------------------------------------------------------------   5501
119_Danko_APR       -----------------------------------GCTTGAAACGGAGGCAATT-TTTTTTGTCTCATCC   5527
119_Danko_APR2      -----------------------------------GCTTGAAACGGAGGCAATT-TTTTTTGTCTCATCC   5527
120_Danko_APR       ----------------------------------------------------------------------   5290
138_Danko_APR       -----------------------------------GCTT-------------------------------   5290
153_Danko_APR       -----------------------------------GCTTGAAA---------------------------   5505
157_Danko_APR       -----------------------------------GCTTGAAACGGAGGCAAT-----------------   5306
160_Danko_APR       -----------------------------------GCTTGAAACGGAGGCAATT-TTTTTTGTCTCATCC   5312
71_PHR_APR          -----------------------------------GCTTGAAACGGAGG---------------------   5371
149_PHR_APR         -----------------------------------GCTTGAAACGGAGGCAATT-TTTTTTGTCT-----   5531
59_Danko_non-APR    -----------------------------------GC---------------------------------   5306
61_Danko_non-APR    -----------------------------------GCTTG------------------------------   5575
123_Danko_non-APR   -----------------------------------GCTTGAAACGGAGGCAATT-TTTTTTGTCTCAT--   5484
129_Danko_non-APR   -----------------------------------GCTTGAAACGGAGGCAATT-TTTTTTGTCTCATCC   5533
37_PHR_non-APR      -----------------------------------GCTTGAAACGGAGGCAATT-TTTTTTGTCTCATCC   5300
52_PHR_non-APR      -----------------------------------GCTTGAAACGGAGGCAATT-TTTTTTGTCTCAT--   5880
88_PHR_non-APR      -----------------------------------GCTTGAAACGGAGGCAATT-TTTTTTGTCTCA---   5511
101_PHR_non-APR     -----------------------------------GCTTGAAACGGAGGC--------------------   5516
105_PHR_non-APR     -----------------------------------GCTTGAAACGGAGGCAATTTTT-------------   5522
150_PHR_non-APR     -----------------------------------GCTTGAAAC--------------------------   5451
150_PHR_non-APR2    -----------------------------------GCTTGAAAC--------------------------   5451
```

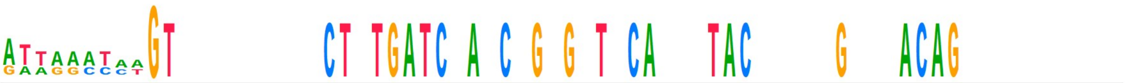

Name	Sequence (7290 – 7350)	
**Consensus**	--	
Lr67(sus)	GAAGGCCCTG--------CCCTGTGATCCATCAGCGTTCCAAGCTACCGCCAGTCCACAGCT--------	5735
Lr67(res)	GAAGGCCCTG--------CCCTGTGATCCATCAGCGTTCCAAGCTACCGCCAGTCCACAGCT--------	5735
ScLr67_1 (Lo7)	ATTAAATAAGTGAAAGGCTTCTTTGATCGAGCGGTGCTTCAGCTTACACATTGATGACAGTGGATCTCGG	5723
118_Danko_APR	--	5501
119_Danko_APR	ATTAAATAAGT---	5538
119_Danko_APR2	ATTAAATAAGT---	5538
120_Danko_APR	--	5290
138_Danko_APR	--	5290
153_Danko_APR	--	5505
157_Danko_APR	--	5306
160_Danko_APR	ATTAAAT---	5319
71_PHR_APR	--	5371
149_PHR_APR	--	5531
59_Danko_non-APR	--	5306
61_Danko_non-APR	--	5575
123_Danko_non-APR	--	5484
129_Danko_non-APR	A---	5534
37_PHR_non-APR	AT--	5302
52_PHR_non-APR	--	5880
88_PHR_non-APR	--	5511
101_PHR_non-APR	--	5516
105_PHR_non-APR	--	5522
150_PHR_non-APR	--	5451
150_PHR_non-APR2	--	5451

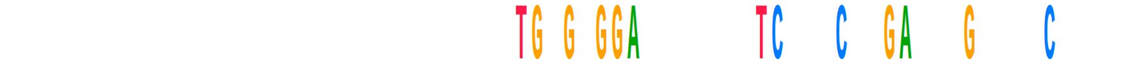

Name	Sequence (7360 – 7420)	
**Consensus**	--	
Lr67(sus)	--------------------------------TGTGAGGAACATTGTTCAACCCAGAATGGATACCCCCA	5773
Lr67(res)	--------------------------------TGTGAGGAACATTGTTCAACCCAGAATGGATACCCCCA	5773
ScLr67_1 (Lo7)	CGGCATTGGCGCAGTGGAGACTTGGCGTCCGATGCGCGGACATGGACTCGCGCAGGAGGAGGAGGCTGTC	5793
118_Danko_APR	--	5501
119_Danko_APR	--	5538
119_Danko_APR2	--	5538
120_Danko_APR	--	5290
138_Danko_APR	--	5290
153_Danko_APR	--	5505
157_Danko_APR	--	5306
160_Danko_APR	--	5319
71_PHR_APR	--	5371
149_PHR_APR	--	5531
59_Danko_non-APR	--	5306
61_Danko_non-APR	--	5575
123_Danko_non-APR	--	5484
129_Danko_non-APR	--	5534
37_PHR_non-APR	--	5302
52_PHR_non-APR	--	5880
88_PHR_non-APR	--	5511
101_PHR_non-APR	--	5516
105_PHR_non-APR	--	5522
150_PHR_non-APR	--	5451
150_PHR_non-APR2	--	5451

	T G T C ATGC A A	
**Consensus**	------------------------------	
	7430 7440 7450	
Lr67(sus)	TTAAGCATTACTCTCAGACATGCCCCACAC	5803
Lr67(res)	TTAAGCATTACTCTCAGACATGCCCCACAC	5803
ScLr67_1 (Lo7)	TGTCGTCATGGCGACGTCAATGGCAGAGAG	5823
118_Danko_APR	------------------------------	5501
119_Danko_APR	------------------------------	5538
119_Danko_APR2	------------------------------	5538
120_Danko_APR	------------------------------	5290
138_Danko_APR	------------------------------	5290
153_Danko_APR	------------------------------	5505
157_Danko_APR	------------------------------	5306
160_Danko_APR	------------------------------	5319
71_PHR_APR	------------------------------	5371
149_PHR_APR	------------------------------	5531
59_Danko_non-APR	------------------------------	5306
61_Danko_non-APR	------------------------------	5575
123_Danko_non-APR	------------------------------	5484
129_Danko_non-APR	------------------------------	5534
37_PHR_non-APR	------------------------------	5302
52_PHR_non-APR	------------------------------	5880
88_PHR_non-APR	------------------------------	5511
101_PHR_non-APR	------------------------------	5516
105_PHR_non-APR	------------------------------	5522
150_PHR_non-APR	------------------------------	5451
150_PHR_non-APR2	------------------------------	5451

Sequence Logo: 50% GC base composition

Consensus Threshold: >50%

Colors: 4-color highlighting

Created: 26 lis 2024

Last Modified: 26 lis 2024

Feature	Location	Size	⇄	Type
