# Supplementary material for: Searching for genes determining the APR phenotype in rye
Source: BMC Plant Biol. 2025 Jul 19;25:935. doi: 10.1186/s12870-025-06920-0 (PMC12275401; doi:10.1186/s12870-025-06920-0)
Supplement: Supplementary file 8 — Supplementary Material 8. [file 12870_2025_6920_MOESM8_ESM.pdf]

Aligned using MUSCLE

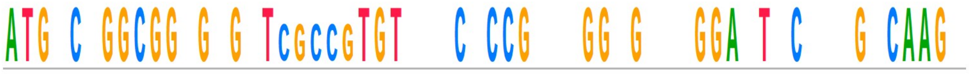

```
Consensus                   ------------------------------------------------------------
                                     10        20        30        40        50        60
Lr67(sus)                   ATGCCGGGCGGGGGGTTCGCCGTGTCGGCGCCGTCCGGCGT--GGAGTTCGAGGCCAAGA  58
Lr67(res)                   ATGCCGGGCGGGGGGTTCGCCGTGTCGGCGCCGTCCGGCGT--GGAGTTCGAGGCCAAGA  58
ScLr_SUG4 (Lo7)             ATGGCCGGCGGCGCGGT-----TGTTAACACCGGAGGGGGGAAGGACTACCCCGGCAAGC  55
ScLr_SUG4_118_DANKO_APR     ------------------------------------------------------------
ScLr_SUG4_120_DANKO_APR     ------------------------------------------------------------
ScLr_SUG4_71_PHR_APR        ------------------------------------------------------------
ScLr_SUG4_149_PHR_APR       ------------------------------------------------------------
ScLr_SUG4_59_DANKO_non-APR  ------------------------------------------------------------
ScLr_SUG4_61_DANKO_non-APR  ------------------------------------------------------------
ScLr_SUG4_88_PHR_non-APR    ------------------------------------------------------------
ScLr_SUG4_105_PHR_non-APR   ------------------------------------------------------------
```

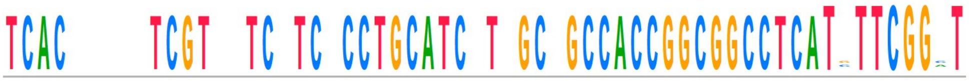

```
Consensus                   ------------------------------------------------------------
                                     70        80        90       100       110       120
Lr67(sus)                   TCACGCCCATCGTCATCATCTCCTGCATCATGGCGGCCACCGGCGGCCTCATGTTCGGCT  118
Lr67(res)                   TCACGCCCATCGTCATCATCTCCTGCATCATGGCGGCCACCGGCGGCCTCATGTTCGGCT  118
ScLr_SUG4 (Lo7)             TCACCATGTTCGTGCTCTTCGCCTGCATCGTCGCCGCCACCGGCGGCCTCATCTTCGGAT  115
ScLr_SUG4_118_DANKO_APR     ------------------------------------------------------------
ScLr_SUG4_120_DANKO_APR     ------------------------------------------------------------
ScLr_SUG4_71_PHR_APR        ------------------------------------------------------------
ScLr_SUG4_149_PHR_APR       ------------------------------------------------------------
ScLr_SUG4_59_DANKO_non-APR  ------------------------------------------------------------
ScLr_SUG4_61_DANKO_non-APR  ------------------------------------------------------------
ScLr_SUG4_88_PHR_non-APR    ------------------------------------------------------------
ScLr_SUG4_105_PHR_non-APR   ---------------------------------------------------TCTTCGGAT  9
```

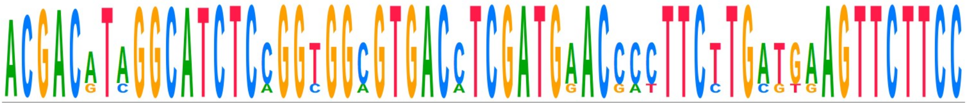

```
Consensus                   ACGACATAGGCATCTCCGGTGGCGTGACCTCGATGAACCCCTTCTTGATGAAGTTCTTCC
                                    130       140       150       160       170       180
Lr67(sus)                   ACGACGTCGGCATCTCAGGCGGAGTGACATCGATGGACGATTTCCTGCGTGAGTTCTTCC  178
Lr67(res)                   ACGACGTCGGCATCTCAGGCGGAGTGACATCGATGGACGATTTCCTGCGTGAGTTCTTCC  178
ScLr_SUG4 (Lo7)             ACGACATAGGCATCTCCGGTGGCGTGACCTCGATGAACCCCTTCTTGATGAAGTTCTTCC  175
ScLr_SUG4_118_DANKO_APR     ACGACATAGGCATCTCCGGTGGCGTGACCTCGATGAACCCCTTCTTGATGAAGTTCTTCC  60
ScLr_SUG4_120_DANKO_APR     -------------CTCCGGTGGCGTGACCTCGATGAACCCCTTCTTGATGAAGTTCTTCC  47
ScLr_SUG4_71_PHR_APR        ------TAGGCATCTCCGGTGGCGTGACCTCGATGAACCCCTTCTTGATGAAGTTCTTCC  54
ScLr_SUG4_149_PHR_APR       ACGACATAGGCATCTCCGGTGGCGTGACCTCGATGAACCCCTTCTTGATGAAGTTCTTCC  60
ScLr_SUG4_59_DANKO_non-APR  --GACATAGGCATCTCCGGTGGCGTGACCTCGATGAACCCCTTCTTGATGAAGTTCTTCC  58
ScLr_SUG4_61_DANKO_non-APR  --GACATAGGCATCTCCGGTGGCGTGACCTCGATGAACCCCTTCTTGATGAAGTTCTTCC  58
ScLr_SUG4_88_PHR_non-APR    -CGACATAGGCATCTCCGGTGGCGTGACCTCGATGAACCCCTTCTTGATGAAGTTCTTCC  59
ScLr_SUG4_105_PHR_non-APR   ACGACATAGGCATCTCCGGTGGCGTGACCTCGATGAACCCCTTCTTGATGAAGTTCTTCC  69
```

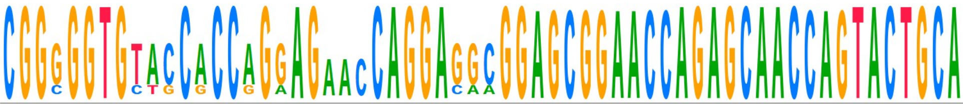

Sequence	Alignment (190–240)	End
**Consensus**	CGGGGGTGTACCACCAGGAG---CAGGAGGCGGAGCGGAACCAGAGCAACCAGTACTGCA	
Lr67(sus)	CGGCGGTGCTGCGCCGGAAGAACCAGGACAAGG---------AGAGCAAC---TACTGCA	226
Lr67(res)	CGGCGGTGCTGCGCCGGAAGAACCAGGACAAGG---------AGAGCAAC---TACTGCA	226
ScLr_SUG4 (Lo7)	CGGGGGTGTACCACCAGGAG---CAGGAGGCGGAGCGGAACCAGAGCAACCAGTACTGCA	232
ScLr_SUG4_118_DANKO_APR	CGGGGGTGTACCACCAGGAG---CAGGAGGCGGAGCGGAACCAGAGCAACCAGTACTGCA	117
ScLr_SUG4_120_DANKO_APR	CGGGGGTGTACCACCAGGAG---CAGGAGGCGGAGCGGAACCAGAGCAACCAGTACTGCA	104
ScLr_SUG4_71_PHR_APR	CGGGGGTGTACCACCAGGAG---CAGGAGGCGGAGCGGAACCAGAGCAACCAGTACTGCA	111
ScLr_SUG4_149_PHR_APR	CGGGGGTGTACCACCAGGAG---CAGGAGGCGGAGCGGAACCAGAGCAACCAGTACTGCA	117
ScLr_SUG4_59_DANKO_non-APR	CGGGGGTGTACCACCAGGAG---CAGGAGGCGGAGCGGAACCAGAGCAACCAGTACTGCA	115
ScLr_SUG4_61_DANKO_non-APR	CGGGGGTGTACCACCAGGAG---CAGGAGGCGGAGCGGAACCAGAGCAACCAGTACTGCA	115
ScLr_SUG4_88_PHR_non-APR	CGGGGGTGTACCACCAGGAG---CAGGAGGCGGAGCGGAACCAGAGCAACCAGTACTGCA	116
ScLr_SUG4_105_PHR_non-APR	CGGGGGTGTACCACCAGGAG---CAGGAGGCGGAGCGGAACCAGAGCAACCAGTACTGCA	126

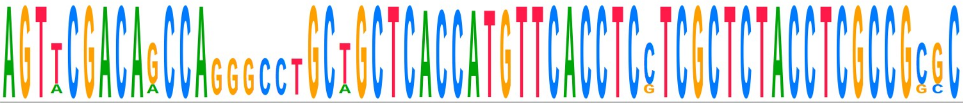

Sequence	Alignment (250–300)	End
**Consensus**	AGTTCGACAGCCA------GCTGCTCACCATGTTCACCTCCTCGCTCTACCTCGCCGCGC	
Lr67(sus)	AGTACGACAACCAGGGCCTGCAGCTC------TTCACCTCGTCGCTCTACCTCGCCGGCC	280
Lr67(res)	AGTACGACAACCAGGGCCTGCAGCTC------TTCACCTCGTCGCTCTACCTCGCCGGCC	280
ScLr_SUG4 (Lo7)	AGTTCGACAGCCA------GCTGCTCACCATGTTCACCTCCTCGCTCTACCTCGCCGCGC	286
ScLr_SUG4_118_DANKO_APR	AGTTCGACAGCCA------GCTGCTCACCATGTTCACCTCCTCGCTCTACCTCGCCGCGC	171
ScLr_SUG4_120_DANKO_APR	AGTTCGACAGCCA------GCTGCTCACCATGTTCACCTCCTCGCTCTACCTCGCCGCGC	158
ScLr_SUG4_71_PHR_APR	AGTTCGACAGCCA------GCTGCTCACCATGTTCACCTCCTCGCTCTACCTCGCCGCGC	165
ScLr_SUG4_149_PHR_APR	AGTTCGACAGCCA------GCTGCTCACCATGTTCACCTCCTCGCTCTACCTCGCCGCGC	171
ScLr_SUG4_59_DANKO_non-APR	AGTTCGACAGCCA------GCTGCTCACCATGTTCACCTCCTCGCTCTACCTCGCCGCGC	169
ScLr_SUG4_61_DANKO_non-APR	AGTTCGACAGCCA------GCTGCTCACCATGTTCACCTCCTCGCTCTACCTCGCCGCGC	169
ScLr_SUG4_88_PHR_non-APR	AGTTCGACAGCCA------GCTGCTCACCATGTTCACCTCCTCGCTCTACCTCGCCGCGC	170
ScLr_SUG4_105_PHR_non-APR	AGTTCGACAGCCA------GCTGCTCACCATGTTCACCTCCTCGCTCTACCTCGCCGCGC	180

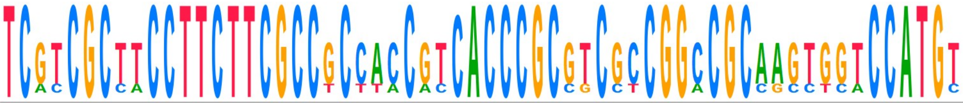

Sequence	Alignment (310–360)	End
**Consensus**	TCGTCGCTTCCTTCTTCGCCGCCACCGTCACCCGCGTCGCCGGCCGCAAGTGGTCCATGT	
Lr67(sus)	TCACCGCCACCTTCTTCGCCTCCTACACCACCCGCCGCCTCGGACGCCGCCTCACCATGC	340
Lr67(res)	TCACCGCCACCTTCTTCGCCTCCTACACCACCCGCCGCCTCGGACGCCGCCTCACCATGC	340
ScLr_SUG4 (Lo7)	TCGTCGCTTCCTTCTTCGCCGCCACCGTCACCCGCGTCGCCGGCCGCAAGTGGTCCATGT	346
ScLr_SUG4_118_DANKO_APR	TCGTCGCTTCCTTCTTCGCCGCTACCGTCACCCGCGTCGCCGGCCGCAAGTGGTCCATGT	231
ScLr_SUG4_120_DANKO_APR	TCGTCGCTTCCTTCTTCGCCGCTACCGTCACCCGCGTCGCCGGCCGCAAGTGGTCCATGT	218
ScLr_SUG4_71_PHR_APR	TCGTCGCTTCCTTCTTCGCCGCCACCGTCACCCGCGTCGCCGGCCGCAAGTGGTCCATGT	225
ScLr_SUG4_149_PHR_APR	TCGTCGCTTCCTTCTTCGCCGCCACCGTNACCCGCGTCGCCGGCCGCAAGTGGTCCATGT	231
ScLr_SUG4_59_DANKO_non-APR	TCGTCGCTTCCTTCTTCGCCGCCACCGTCACCCGCGTCGCCGGCCGCAAGTGGTCCATGT	229
ScLr_SUG4_61_DANKO_non-APR	TCGTCGCTTCCTTCTTCGCCGCCACCGTNACCCGCGTCGCCGGCCGCAAGTGGTCCATGT	229
ScLr_SUG4_88_PHR_non-APR	TCGTCGCTTCCTTCTTCGCCGCCACCGTNACCCGCGTCGCCGGCCGCAAGTGGTCCATGT	230
ScLr_SUG4_105_PHR_non-APR	TCGTCGCTTCCTTCTTCGCCGCCACCGTNACCCGCGTCGCCGGCCGCAAGTGGTCCATGT	240

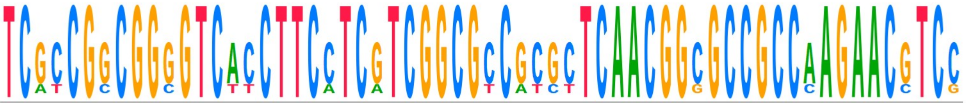

Consensus	TCGCCGGCGGGGTCACCTTCCTCGTCGGCGCCGCGCTCAACGGCGCCGCCAAGAACGTCC	
	370 380 390 400 410 420	
Lr67(sus)	TCATCGCCGGCGTCTTCTTCATCATCGGCGTCATCTTCAACGGGGCCGCCCAGAACCTCG	400
Lr67(res)	TCATCGCCGGCGTCTTCTTCATCATCGGCGTCATCTTCAACGGGGCCGCCCAGAACCTCG	400
ScLr_SUG4 (Lo7)	TCGCCGGCGGGGTCACCTTCCTCGTCGGCGCCGCGCTCAACGGCGCCGCCAAGAACGTCC	406
ScLr_SUG4_118_DANKO_APR	TCGCCGGCGGGGTCACCTTCCTCGTCGGCGCCGCGCTCAACGGCGCCGCCAAGAACGTCC	291
ScLr_SUG4_120_DANKO_APR	TCGCCGGCGGGGTCACCTTCCTCGTCGGCGCCGCGCTCAACGGCGCCGCCAAGAACGTCC	278
ScLr_SUG4_71_PHR_APR	TCGCCGGCGGGGTCACCTTCCTCGTCGGCGCCGCGCTCAACGGCGCCGCCAAGAACGTCC	285
ScLr_SUG4_149_PHR_APR	TCGCCGGCGGGGTCACCTTCCTCGTCGGCGCCGCGCTCAACGGCGCCGCCAAGAACGTCC	291
ScLr_SUG4_59_DANKO_non-APR	TCGCCGGCGGGGTCACCTTCCTCGTCGGCGCCGCGCTCAACGGCGCCGCCAAGAACGTCC	289
ScLr_SUG4_61_DANKO_non-APR	TCGCCGGCGGGGTCACCTTCCTCGTCGGCGCCGCGCTCAACGGCGCCGCCAAGAACGTCC	289
ScLr_SUG4_88_PHR_non-APR	TCGCCGGCGGGGTCACCTTCCTCGTCGGCGCCGCGCTCAACGGCGCCGCCAAGAACGTCC	290
ScLr_SUG4_105_PHR_non-APR	TCGCCGGCGGGGTCACCTTCCTCGTCGGCGCCGCGCTCAACGGCGCCGCCAAGAACGTCC	300

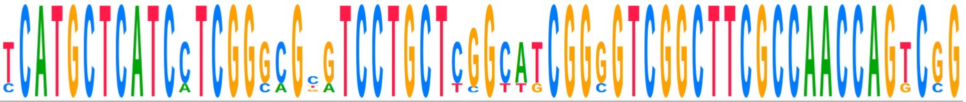

Consensus	TCATGCTCATCCTCGGGCGCGTCCTGCTCGGCATCGGGGTCGGCTTCGCCAACCAGTCGG	
	430 440 450 460 470 480	
Lr67(sus)	CCATGCTCATCATCGGCAGGATCCTGCTTGGTTGCGGCGTCGGCTTCGCCAACCAGGCCG	460
Lr67(res)	CCATGCTCATCATCGGCAGGATCCTGCTTCGTTGCGGCGTCGGCTTCGCCAACCAGGCCG	460
ScLr_SUG4 (Lo7)	TCATGCTCATCCTCGGGCGCGTCCTGCTCGGCATCGGGGTCGGCTTCGCCAACCAGTCGG	466
ScLr_SUG4_118_DANKO_APR	TCATGCTCATCCTCGGGCGTGTCCTGCTCGGCATCGGGGTCGGCTTCGCCAACCAGTCGG	351
ScLr_SUG4_120_DANKO_APR	TCATGCTCATCCTCGGGCGTGTCCTGCTCGGCATCGGGGTCGGCTTCGCCAACCAGTCGG	338
ScLr_SUG4_71_PHR_APR	TCATGCTCATCCTCGGGCGCGTCCTGCTCGGCATCGGGGTCGGCTTCGCCAACCAGTCGG	345
ScLr_SUG4_149_PHR_APR	TCATGCTCATCCTCGGGCGCGTCCTGCTCGGCATCGGGGTCGGCTTCGCCAACCAGTCGG	351
ScLr_SUG4_59_DANKO_non-APR	TCATGCTCATCCTCGGGCGCGTCCTGCTCGGCATCGGGGTCGGCTTCGCCAACCAGTCGG	349
ScLr_SUG4_61_DANKO_non-APR	TCATGCTCATCCTCGGGCGCGTCCTGCTCGGCATCGGGGTCGGCTTCGCCAACCAGTCGG	349
ScLr_SUG4_88_PHR_non-APR	TCATGCTCATCCTCGGGCGCGTCCTGCTCGGCATCGGGGTCGGCTTCGCCAACCAGTCGG	350
ScLr_SUG4_105_PHR_non-APR	TCATGCTCATCCTCGGGCGCGTCCTGCTCGGCATCGGGGTCGGCTTCGCCAACCAGTCGG	360

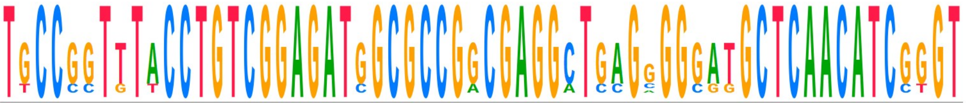

Consensus	TGCCGGTTTACCTGTCGGAGATGGCGCCGGCGAGGCTGAGGGGGATGCTCAACATCGGGT	
	490 500 510 520 530 540	
Lr67(sus)	TTCCCCTGTTCCTGTCGGAGATCGCGCCGACGAGGATCCGCGGCGGGCTCAACATCCTGT	520
Lr67(res)	TTCCCCTGTTCCTGTCGGAGATCGCGCCGACGAGGATCCGCGGCGGGCTCAACATCCTGT	520
ScLr_SUG4 (Lo7)	TGCCGGTTTACCTGTCGGAGATGGCGCCGGCGAGGCTGAGGGGGATGCTCAACATCGGGT	526
ScLr_SUG4_118_DANKO_APR	TGCCGGTTTACCTGTCGGAGATGGCGCCGGCGAGGCTGAGGGGGATGCTCAACATCGGGT	411
ScLr_SUG4_120_DANKO_APR	TGCCGGTTTACCTGTCGGAGATGGCGCCGGCGAGGCTGAGGGGGATGCTCAACATCGGGT	398
ScLr_SUG4_71_PHR_APR	TGCCGGTTTACCTGTCGGAGATGGCGCCGGCGAGGCTGAGGGGGATGCTCAACATCGGGT	405
ScLr_SUG4_149_PHR_APR	TGCCGGTTTACCTGTCGGAGATGGCGCCGGCGAGGCTGAGGGGGATGCTCAACATCGGGT	411
ScLr_SUG4_59_DANKO_non-APR	TGCCGGTTTACCTGTCGGAGATGGCGCCGGCGAGGCTGAGGGGGATGCTCAACATCGGGT	409
ScLr_SUG4_61_DANKO_non-APR	TGCCGGTTTACCTGTCGGAGATGGCGCCGGCGAGGCTGAGGGGGATGCTCAACATCGGGT	409
ScLr_SUG4_88_PHR_non-APR	TGCCGGTTTACCTGTCGGAGATGGCGCCGGCGAGGCTGAGAGGGGATGCTCAACATCGGGT	410
ScLr_SUG4_105_PHR_non-APR	TGCCGGTTTACCTGTCGGAGATGGCGCCGGCGAGGCTGAGGGGGATGCTCAACATCGGGT	420

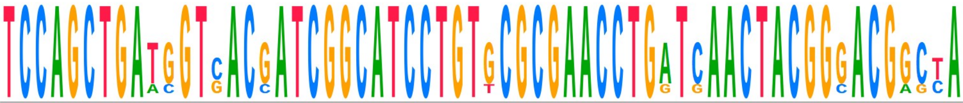

**Consensus**	TCCAGCTGATGGTCACGATCGGCATCCTGTGCGCGAACCTGATCAACTACGGGACGGCTA	
	550 560 570 580 590 600	
Lr67(sus)	TCCAGCTGAACGTGACCATCGGCATCCTGTTCGCGAACCTGGTGAACTACGGCACGAGCA	580
Lr67(res)	TCCAGCTGAACGTGACCATCGGCATCCTGTTCGCGAACCTGGTGAACTACGGCACGAGCA	580
ScLr_SUG4 (Lo7)	TCCAGCTGATGGTCACGATCGGCATCCTGTGCGCGAACCTGATCAACTACGGGACGGCTA	586
ScLr_SUG4_118_DANKO_APR	TCCAGCTGATGGTGACGATCGGCATCCTGTGCGCGAACCTGATCAACTACGGGACGGCCA	471
ScLr_SUG4_120_DANKO_APR	TCCAGCTGATGGTGACGATCGGCATCCTGTGCGCGAACCTGATCAACTACGGGACGGCCA	458
ScLr_SUG4_71_PHR_APR	TCCAGCTGATGGTCACGATCGGCATCCTGTGCGCGAACCTGATCAACTACGGGACGGCTA	465
ScLr_SUG4_149_PHR_APR	TCCAGCTGATGGTCACGATCGGCATCCTGTGCGCGAACCTGATCAACTACGGGACGGCTA	471
ScLr_SUG4_59_DANKO_non-APR	TCCAGCTGATGGTCACGATCGGCATCCTGTGCGCGAACCTGATCAACTACGGGACGGCTA	469
ScLr_SUG4_61_DANKO_non-APR	TCCAGCTGATGGTCACGATCGGCATCCTGTGCGCGAACCTGATCAACTACGGGACGGCTA	469
ScLr_SUG4_88_PHR_non-APR	TCCAGCTGATGGTCACGATCGGCATCCTGTGCGCGAACCTGATCAACTACGGGACGGCTA	470
ScLr_SUG4_105_PHR_non-APR	TCCAGCTGATGGTCACGATCGGCATCCTGTGCGCGAACCTGATCAACTACGGGACGGCTA	480

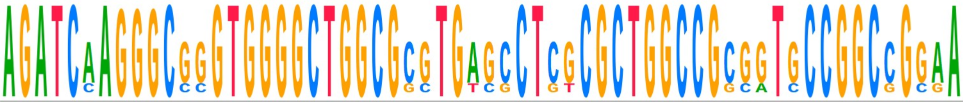

**Consensus**	AGATCAAGGGCGGGTGGGGCTGGCGCGTGAGCCTCGCGCTGGCCGCGGTGCCGGCCGGAA	
	610 620 630 640 650 660	
Lr67(sus)	AGATCCA---CCCGTGGGGCTGGCGGCTGTCGCTGTCGCTGGCCGGCATCCCGGCGGCGA	637
Lr67(res)	AGATCCA---CCCGTGGGGCTGGCGGCTGTCGCTGTCGCTGGCCGGCATCCCGGCGGCGA	637
ScLr_SUG4 (Lo7)	AGATCAAGGGCGGGTGGGGCTGGCGCGTGAGCCTCGCGCTGGCCGCGGTGCCGGCCGGAA	646
ScLr_SUG4_118_DANKO_APR	AGATCAAGGGCGGGTGGGGCTGGCGCGTGAGCCTCGCGCTGGCCGCGGTGCCGGCCGGAA	531
ScLr_SUG4_120_DANKO_APR	AGATCAAGGGCGGGTGGGGCTGGCGCGTGAGCCTCGCGCTGGCCGCGGTGCCGGCCGGAA	518
ScLr_SUG4_71_PHR_APR	AGATCAAGGGCGGGTGGGGCTGGCGCGTGAGCCTCGCGCTGGCCGCGGTGCCGGCCGGAA	525
ScLr_SUG4_149_PHR_APR	AGATCAAGGGCGGGTGGGGCTGGCGCGTGAGCCTCGCGCTGGCCGCGGTGCCGGCCGGAA	531
ScLr_SUG4_59_DANKO_non-APR	AGATCAAGGGCGGGTGGGGCTGGCGCGTGAGCCTCGCGCTGGCCGCGGTGCCGGCCGGAA	529
ScLr_SUG4_61_DANKO_non-APR	AGATCAAGGGCGGGTGGGGCTGGCGCGTGAGCCTCGCGCTGGCCGCGGTGCCGGCCGGAA	529
ScLr_SUG4_88_PHR_non-APR	AGATCAAGGGCGGGTGGGGCTGGCGCGTGAGCCTCGCGCTGGCCGCGGTGCCGGCCGGAA	530
ScLr_SUG4_105_PHR_non-APR	AGATCAAGGGCGGGTGGGGCTGGCGCGTGAGCCTCGCGCTGGCCGCGGTGCCGGCCGGAA	540

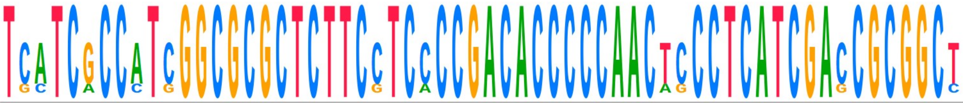

**Consensus**	TCATCGCCATCGGCGCGCTCTTCCTCCCCGACACCCCCAACTCCCTCATCGACCGCGGCT	
	670 680 690 700 710 720	
Lr67(sus)	TGCTCACCCTGGGCGCGCTCTTCGTCACCGACACCCCCAACAGCCTCATCGAGCGCGGCC	697
Lr67(res)	TGCTCACCCTGGGCGCGCTCTTCGTCACCGACACCCCCAACAGCCTCATCGAGCGCGGCC	697
ScLr_SUG4 (Lo7)	TCATCGCCATCGGCGCGCTCTTCCTCCCCGACACCCCCAACTCCCTCATCGACCGCGGCT	706
ScLr_SUG4_118_DANKO_APR	TCATCGCCATCGGCGCGCTCTTCCTCCCCGACACCCCCAACTCCCTCATCGACCGCGGCT	591
ScLr_SUG4_120_DANKO_APR	TCATCGCCATCGGCGCGCTCTTCCTCCCCGACACCCCCAACTCCCTCATCGACCGCGGCT	578
ScLr_SUG4_71_PHR_APR	TCATCGCCATCGGCGCGCTCTTCCTCCCCGACACCCCCAACTCCCTCATCGACCGCGGCT	585
ScLr_SUG4_149_PHR_APR	TCATCGCCATCGGCGCGCTCTTCCTCCCCGACACCCCCAACTCCCTCATCGACCGCGGCT	591
ScLr_SUG4_59_DANKO_non-APR	TCATCGCCATCGGCGCGCTCTTCCTCCCCGACACCCCCAACTCCCTCATCGACCGCGGCT	589
ScLr_SUG4_61_DANKO_non-APR	TCATCGCCATCGGCGCGCTCTTCCTCCCCGACACCCCCAACTCCCTCATCGACCGCGGCT	589
ScLr_SUG4_88_PHR_non-APR	TCATCGCCATCGGCGCGCTCTTCCTCCCCGACACCCCCAACTCCCTCATCGACCGCGGCT	590
ScLr_SUG4_105_PHR_non-APR	TCATCGCCATCGGCGCGCTCTTCCTCCCCGACACCCCCAACTCCCTCATCGACCGCGGCT	600

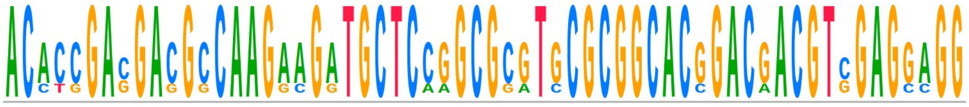

**Consensus**	ACACCGACGACGCCAAGAAGATGCTCCGGCGCGTGCGCGGCACGGACGACGTCGAGGAGG	
	730 740 750 760 770 780	
Lr67(sus)	ACCTGGAGGAGGGCAAGGCGGTGCTCAAGCGGATCCGCGGCACCGACAACGTGGAGCCGG	757
Lr67(res)	ACCTGGAGGAGGGCAAGGCGGTGCTCAAGCGGATCCGCGGCACCGACAACGTGGAGCCGG	757
ScLr_SUG4 (Lo7)	ACACCGACGACGCCAAGAAGATGCTCCGGCGCGTGCGCGGCACGGACGACGTCGAGGAGG	766
ScLr_SUG4_118_DANKO_APR	ACACCGAGGACGCCAAGAAGATGCTCCGGCGCGTGCGCGGCACGGACGACGTGGAGGAGG	651
ScLr_SUG4_120_DANKO_APR	ACACCGAGGACGCCAAGAAGATGCTCCGGCGCGTGCGCGGCACGGACGACGTGGAGGAGG	638
ScLr_SUG4_71_PHR_APR	ACACCGACGACGCCAAGAAGATGCTCCGGCGCGTGCGCGGCACGGACGACGTCGAGGAGG	645
ScLr_SUG4_149_PHR_APR	ACACCGACGACGCCAAGAAGATGCTCCGGCGCGTGCGCGGCACGGACGACGTCGAGGAGG	651
ScLr_SUG4_59_DANKO_non-APR	ACACCGACGACGCCAAGAAGATGCTCCGGCGCGTGCGCGGCACGGACGACGTCGAGGAGG	649
ScLr_SUG4_61_DANKO_non-APR	ACACCGACGACGCCAAGAAGATGCTCCGGCGCGTGCGCGGCACGGACGACGTCGAGGAGG	649
ScLr_SUG4_88_PHR_non-APR	ACACCGACGACGCCAAGAAGATGCTCCGGCGCGTGCGCGGCACGGACGACGTCGAGGAGG	650
ScLr_SUG4_105_PHR_non-APR	ACACCGACGACGCCAAGAAGATGCTCCGGCGCGTGCGCGGCACGGACGACGTCGAGGAGG	660

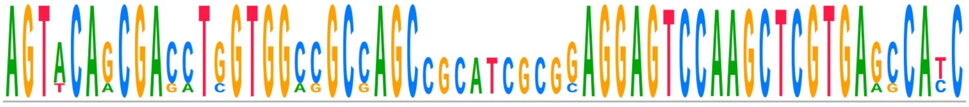

**Consensus**	AGTACAGCGACCTGGTGGCCGCCAGC---------GAGGAGTCCAAGCTCGTGAGCCATC	
	790 800 810 820 830 840	
Lr67(sus)	AGTTCAACGAGATCGTGGAGGCGAGCCGCATCGCGCAGGAG---------GTGAAGCACC	808
Lr67(res)	AGTTCAACGAGATCGTGGAGGCGAGCCGCATCGCGCAGGAG---------GTGAAGCACC	808
ScLr_SUG4 (Lo7)	AGTACAGCGACCTGGTGGCCGCCAGC---------GAGGAGTCCAAGCTCGTGAGCCATC	817
ScLr_SUG4_118_DANKO_APR	AGTACAGCGACCTGGTGGCCGCCAGC---------GAGGAGTCCAAGCTCGTGAGCCACC	702
ScLr_SUG4_120_DANKO_APR	AGTACAGCGACCTGGTGGCCGCCAGC---------GAGGAGTCCAAGCTCGTGAGCCACC	689
ScLr_SUG4_71_PHR_APR	AGTACAGCGACCTGGTGGCCGCCAGC---------GAGGAGTCCAAGCTCGTGAGCCATC	696
ScLr_SUG4_149_PHR_APR	AGTACAGCGACCTGGTGGCCGCCAGC---------GAGGAGTCCAAGCTCGTGAGCCATC	702
ScLr_SUG4_59_DANKO_non-APR	AGTACAGCGACCTGGTGGCCGCCAGC---------GAGGAGTCCAAGCTCGTGAGCCATC	700
ScLr_SUG4_61_DANKO_non-APR	AGTACAGCGACCTGGTGGCCGCCAGC---------GAGGAGTCCAAGCTCGTGAGCCATC	700
ScLr_SUG4_88_PHR_non-APR	AGTACAGCGACCTGGTGGCCGCCAGC---------GAGGAGTCCAAGCTCGTGAGCCATC	701
ScLr_SUG4_105_PHR_non-APR	AGTACAGCGACCTGGTGGCCGCCAGC---------GAGGAGTCCAAGCTCGTGAGCCATC	711

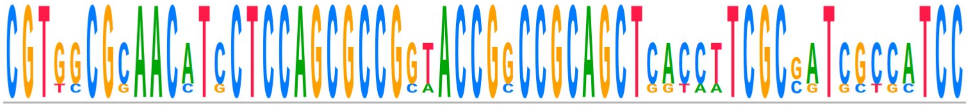

**Consensus**	CGTGGCGCAACATCCTCCAGCGCCGGTACCGGCCGCAGCTCACCTTCGCGATCGCCATCC	
	850 860 870 880 890 900	
Lr67(sus)	CGTTCCGGAACCTGCTCCAGCGCCGGAACCGCCCGCAGCTGGTCATCGCCGTGCTGCTCC	868
Lr67(res)	CGTTCCGGAACCTGCTCCAGCGCCGGAACCGCCCGCAGCTGGTCATCGCCGTGCTGCTCC	868
ScLr_SUG4 (Lo7)	CGTGGCGCAACATCCTCCAGCGCCGGTACCGGCCGCAGCTCACCTTCGCGATCGCCATCC	877
ScLr_SUG4_118_DANKO_APR	CGTGGCGCAACATCCTCCAGCGCCGCTACCGGCCGCAGCTCACATTCGCCATCGCCATCC	762
ScLr_SUG4_120_DANKO_APR	CGTGGCGCAACATCCTCCAGCGCCGCTACCGGCCGCAGCTCACATTCGCCATCGCCATCC	749
ScLr_SUG4_71_PHR_APR	CGTGGCGCAACATCCTCCAGCGCCGGTACCGGCCGCAGCTCACCTTCGCGATCGCCATCC	756
ScLr_SUG4_149_PHR_APR	CGTGGCGCAACATCCTCCAGCGCCGGTACCGGCCGCAGCTCACCTTCGCGATCGCCATCC	762
ScLr_SUG4_59_DANKO_non-APR	CGTGGCGCAACATCCTCCAGCGCCGGTACCGGCCGCAGCTCACCTTCGCGATCGCCATCC	760
ScLr_SUG4_61_DANKO_non-APR	CGTGGCGCAACATCCTCCAGCGCCGGTACCGGCCGCAGCTCACCTTCGCGATCGCCATCC	760
ScLr_SUG4_88_PHR_non-APR	CGTGGCGCAACATCCTCCAGCGCCGGTACCGGCCGCAGCTCACCTTCGCGATCGCCATCC	761
ScLr_SUG4_105_PHR_non-APR	CGTGGCGCAACATCCTCCAGCGCCGGTACCGGCCGCAGCTCACCTTCGCGATCGCCATCC	771

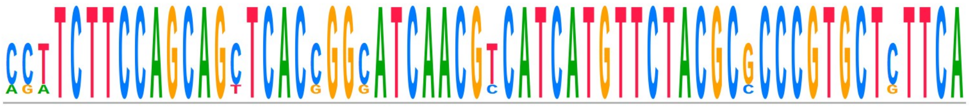

```
Consensus                      CCTTCTTCCAGCAGCTCACCGGCATCAACGTCATCATGTTCTACGCGCCCGTGCTCTTCA
                                        910       920       930       940       950       960
Lr67(sus)                      AGATCTTCCAGCAGTTCACGGGGATCAACGCCATCATGTTCTACGCCCCCGTGCTGTTCA  928
Lr67(res)                      AGATCTTCCAGCAGTTCACGGGGATCAACGCCATCATGTTCTACGCCCCCGTGCTGTTCA  928
ScLr_SUG4 (Lo7)                CCTTCTTCCAGCAGCTCACCGGCATCAACGTCATCATGTTCTACGCGCCCGTGCTCTTCA  937
ScLr_SUG4_118_DANKO_APR        CCTTCTTCCAGCAGCTCACCGGCATCAACGTCATCATGTTCTACGCGCCCGTGCTCTTCA  822
ScLr_SUG4_120_DANKO_APR        CCTTCTTCCAGCAGCTCACCGGCATCAACGTCATCATGTTCTACGCGCCCGTGCTCTTCA  809
ScLr_SUG4_71_PHR_APR           CCTTCTTCCAGCAGCTCACCGGCATCAACGTCATCATGTTCTACGCGCCCGTGCTCTTCA  816
ScLr_SUG4_149_PHR_APR          CCTTCTTCCAGCAGCTCACCGGCATCAACGTCATCATGTTCTACGCGCCCGTGCTCTTCA  822
ScLr_SUG4_59_DANKO_non-APR     CCTTCTTCCAGCAGCTCACCGGCATCAACGTCATCATGTTCTACGCGCCCGTGCTCTTCA  820
ScLr_SUG4_61_DANKO_non-APR     CCTTCTTCCAGCAGCTCACCGGCATCAACGTCATCATGTTCTACGCGCCCGTGCTCTTCA  820
ScLr_SUG4_88_PHR_non-APR       CCTTCTTCCAGCAGCTCACCGGCATCAACGTCATCATGTTCTACGCGCCCGTGCTCTTCA  821
ScLr_SUG4_105_PHR_non-APR      CCTTCTTCCAGCAGCTCACCGGCATCAACGTCATCATGTTCTACGCGCCCGTGCTCTTCA  831
```

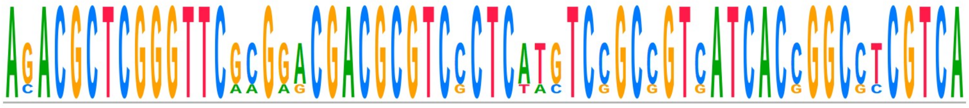

```
Consensus                      AGACGCTCGGGTTCGCGGACGACGCGTCCCTCATGTCCGCCGTCATCACCGGCCTCGTCA
                                        970       980       990      1000      1010      1020
Lr67(sus)                      ACACGCTCGGGTTCAAGAGCGACGCGTCGCTCTACTCGGCGGTGATCACGGGCGCCGTCA  988
Lr67(res)                      ACACGCTCGGGTTCAAGAGCGACGCGTCGCTCTACTCGGCGGTGATCACGGGCGCCGTCA  988
ScLr_SUG4 (Lo7)                AGACGCTCGGGTTCGCGGACGACGCGTCCCTCATGTCCGCCGTCATCACCGGCCTCGTCA  997
ScLr_SUG4_118_DANKO_APR        AGACGCTCGGGTTCGCGGACGACGCGTCCCTCATGTCCGCCGTCATCACCGGCCTCGTCA  882
ScLr_SUG4_120_DANKO_APR        AGACGCTCGGGTTCGCGGACGACGCGTCCCTCATGTCCGCCGTCATCACCGGCCTCGTCA  869
ScLr_SUG4_71_PHR_APR           AGACGCTCGGGTTCGCGGACGACGCGTCCCTCATGTCCGCCGTCATCACCGGCCTCGTCA  876
ScLr_SUG4_149_PHR_APR          AGACGCTCGGGTTCGCGGACGACGCGTCCCTCATGTCCGCCGTCATCACCGGCCTCGTCA  882
ScLr_SUG4_59_DANKO_non-APR     AGACGCTCGGGTTCGCGGACGACGCGTCCCTCATGTCCGCCGTCATCACCGGCCTCGTCA  880
ScLr_SUG4_61_DANKO_non-APR     AGACGCTCGGGTTCGCGGACGACGCGTCCCTCATGTCCGCCGTCATCACCGGCCTCGTCA  880
ScLr_SUG4_88_PHR_non-APR       AGACGCTCGGGTTCGCGGACGACGCGTCCCTCATGTCCGCCGTCATCACCGGCCTCGTCA  881
ScLr_SUG4_105_PHR_non-APR      AGACGCTCGGGTTCGCGGACGACGCGTCCCTCATGTCCGCCGTCATCACCGGCCTCGTCA  891
```

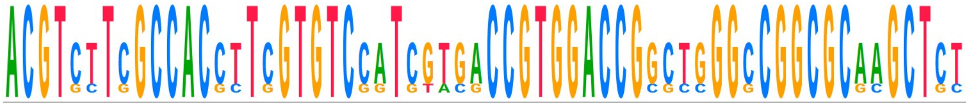

```
Consensus                      ACGTCTTCGCCACCTTCGTGTCCATCGTGACCGTGGACCGGCTGGGCCGGCGCAAGCTCT
                                       1030      1040      1050      1060      1070      1080
Lr67(sus)                      ACGTGCTGGCCACGCTGGTGTCGGTGTACGCCGTGGACCGCGCCGGGCGGCGCGCGCTGC  1048
Lr67(res)                      ACGTGCTGGCCACGCTGGTGTCGGTGTACGCCGTGGACCGCGCCGGGCGGCGCGCGCTGC  1048
ScLr_SUG4 (Lo7)                ACGTCTTCGCCACCTTCGTGTCCATCGTGACCGTGGACCGGCTGGGCCGGCGCAAGCTCT  1057
ScLr_SUG4_118_DANKO_APR        ACGTCTTCGCCACCTTCGTGTCCATCGTGACCGTGGACCGGCTGGGCCGGCGCAAGCTCT  942
ScLr_SUG4_120_DANKO_APR        ACGTCTTCGCCACCTTCGTGTCCATCGTGACCGTGGACCGGCTGGGCCGGCGCAAGCTCT  929
ScLr_SUG4_71_PHR_APR           ACGTCTTCGCCACCTTCGTGTCCATCGTGACCGTGGACCGGCTGGGCCGGCGCAAGCTCT  936
ScLr_SUG4_149_PHR_APR          ACGTCTTCGCCACCTTCGTGTCCATCGTGACCGTGGACCGGCTGGGCCGGCGCAAGCTCT  942
ScLr_SUG4_59_DANKO_non-APR     ACGTCTTCGCCACCTTCGTGTCCATCGTGACCGTGGACCGGCTGGGCCGGCGCAAGCTCT  940
ScLr_SUG4_61_DANKO_non-APR     ACGTCTTCGCCACCTTCGTGTCCATCGTGACCGTGGACCGGCTGGGCCGGCGCAAGCTCT  940
ScLr_SUG4_88_PHR_non-APR       ACGTCTTCGCCACCTTCGTGTCCATCGTGACCGTGGACCGGCTGGGCCGGCGCAAGCTCT  941
ScLr_SUG4_105_PHR_non-APR      ACGTCTTCGCCACCTTCGTGTCCATCGTGACCGTGGACCGGCTGGGCCGGCGCAAGCTCT  951
```

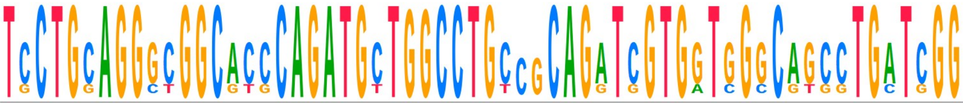

```
Consensus                     TCCTGCAGGGCGGCACCCAGATGCTGGCCTGC--CAGATCGTGGTGGGCAGCCTGATCGG
                                        1090      1100      1110      1120      1130      1140
Lr67(sus)                     TGCTGGAGGCTGGCGTGCAGATGTT--CCTGTCGCAGGTGGTGATCGCCGTGGTGCTGGG  1106
Lr67(res)                     TGCTGGAGGCTGGCGTGCAGATGTT--CCTGTCGCAGGTGGTGATCGCCGTGGTGCTGGG  1106
ScLr_SUG4 (Lo7)               TCCTGCAGGGCGGCACCCAGATGCTGGCCTGC--CAGATCGTGGTGGGCAGCCTGATCGG  1115
ScLr_SUG4_118_DANKO_APR       TCCTGCAGGGCGGCACCCAGATGCTGGCCTGC--CAGATCGTGGTGGGCAGCCTGATCGG  1000
ScLr_SUG4_120_DANKO_APR       TCCTGCAGGGCGGCACCCAGATGCTGGCCTGC--CAGATCGTGGTGGGCAGCCTGATCGG  987
ScLr_SUG4_71_PHR_APR          TCCTGCAGGGCGGCACCCAGATGCTGGCCTGC--CAGATCGTGGTGGGCAGCCTGATCGG  994
ScLr_SUG4_149_PHR_APR         TCCTGCAGGGCGGCACCCAGATGCTGGCCTGC--CAGATCGTGGTGGGCAGCCTGATCGG  1000
ScLr_SUG4_59_DANKO_non-APR    TCCTGCAGGGCGGCACCCAGATGCTGGCCTGC--CAGATCGTGGTGGGCAGCCTGATCGG  998
ScLr_SUG4_61_DANKO_non-APR    TCCTGCAGGGCGGCACCCAGATGCTGGCCTGC--CAGATCGTGGTGGGCAGCCTGATCGG  998
ScLr_SUG4_88_PHR_non-APR      TCCTGCAGGGCGGCACCCAGATGCTGGCCTGC--CAGATCGTGGTGGGCAGCCTGATCGG  999
ScLr_SUG4_105_PHR_non-APR     TCCTGCAGGGCGGCACCCAGATGCTGGCCTGC--CAGATCGTGGTGGGCAGCCTGATCGG  1009
```

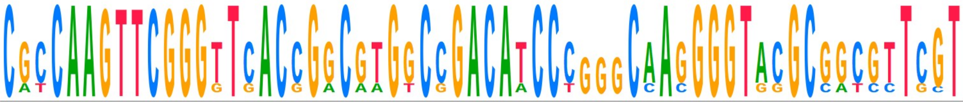

```
Consensus                     CGCCAAGTTCGGGTTCACCGGCGTGGCCGACATCCC---CAAGGGGTACGCGGCGTTCGT
                                        1150      1160      1170      1180      1190      1200
Lr67(sus)                     CATCAA------GGTGACGGACAAGTCGGACAACCTGGGCCACGGGTGGGCCATCCTGGT  1160
Lr67(res)                     CATCAA------GGTGACGGACAAGTCGGACAACCTGGGCCACGGGTGGGCCATCCTGCT  1160
ScLr_SUG4 (Lo7)               CGCCAAGTTCGGGTTCACCGGCGTGGCCGACATCCC---CAAGGGGTACGCGGCGTTCGT  1172
ScLr_SUG4_118_DANKO_APR       CGCCAAGTTCGGGTTCACCGGCGTGGCCGACATCCC---CAAGGGGTACGCGGCGTTCGT  1057
ScLr_SUG4_120_DANKO_APR       CGCCAAGTTCGGGTTCACCGGCGTGGCCGACATCCC---CAAGGGGTACGCGGCGTTCGT  1044
ScLr_SUG4_71_PHR_APR          CGCCAAGTTCGGGTTCACCGGCGTGGCCGACATCCC---CAAGGGGTACGCGGCGTTCGT  1051
ScLr_SUG4_149_PHR_APR         CGCCAAGTTCGGGTTCACCGGCGTGGCCGACATCCC---CAAGGGGTACGCGGCGTTCGT  1057
ScLr_SUG4_59_DANKO_non-APR    CGCCAAGTTCGGGTTCACCGGCGTGGCCGACATCCC---CAAGGGGTACGCGGCGTTCGT  1055
ScLr_SUG4_61_DANKO_non-APR    CGCCAAGTTCGGGTTCACCGGCGTGGCCGACATCCC---CAAGGGGTACGCGGCGTTCGT  1055
ScLr_SUG4_88_PHR_non-APR      CGCCAAGTTCGGGTTCACCGGCGTGGCCGACATCCC---CAAGGGGTACGCGGCGTTCGT  1056
ScLr_SUG4_105_PHR_non-APR     CGCCAAGTTCGGGTTCACCGGCGTGGCCGACATCCC---CAAGGGGTACGCGGCGTTCGT  1066
```

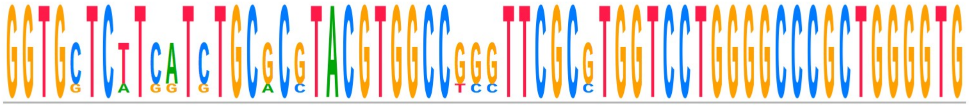

```
Consensus                     GGTGCTCTTCATCTGCGCGTACGTGGCCGGGTTCGCGTGGTCCTGGGGCCCGCTGGGGTG
                                        1210      1220      1230      1240      1250      1260
Lr67(sus)                     GGTGGTCATGGTGTGCACCTACGTGGCCTCCTTCGCCTGGTCCTGGGGCCCGCTGGGGTG  1220
Lr67(res)                     GGTGGTCATGGTGTGCACCTACGTGGCCTCCTTCGCCTGGTCCTGGGGCCCGCTGGGGTG  1220
ScLr_SUG4 (Lo7)               GGTGCTCTTCATCTGCGCGTACGTGGCCGGGTTCGCGTGGTCCTGGGGCCCGCTGGGGTG  1232
ScLr_SUG4_118_DANKO_APR       GGTGCTCTTCATCTGCGCGTACGTGGCCGGGTTCGCGTGGTCCTGGGGCCCGCTGGGGTG  1117
ScLr_SUG4_120_DANKO_APR       GGTGCTCTTCATCTGCGCGTACGTGGCCGGGTTCGCGTGGTCCTGGGGCCCGCTGGGGTG  1104
ScLr_SUG4_71_PHR_APR          GGTGCTCTTCATCTGCGCGTACGTGGCCGGGTTCGCGTGGTCCTGGGGCCCGCTGGGGTG  1111
ScLr_SUG4_149_PHR_APR         GGTGCTCTTCATCTGCGCGTACGTGGCCGGGTTCGCGTGGTCCTGGGGCCCGCTGGGGTG  1117
ScLr_SUG4_59_DANKO_non-APR    GGTGCTCTTCATCTGCGCGTACGTGGCCGGGTTCGCGTGGTCCTGGGGCCCGCTGGGGTG  1115
ScLr_SUG4_61_DANKO_non-APR    GGTGCTCTTCATCTGCGCGTACGTGGCCGGGTTCGCGTGGTCCTGGGGCCCGCTGGGGTG  1115
ScLr_SUG4_88_PHR_non-APR      GGTGCTCTTCATCTGCGCGTACGTGGCCGGGTTCGCGTGGTCCTGGGGCCCGCTGGGGTG  1116
ScLr_SUG4_105_PHR_non-APR     GGTGCTCTTCATCTGCGCGTACGTGGCCGGGTTCGCGTGGTCCTGGGGCCCGCTGGGGTG  1126
```

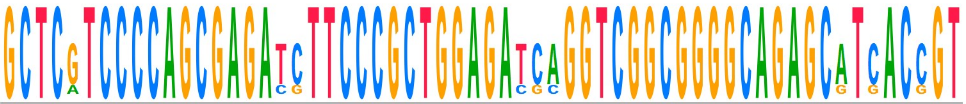

```
Consensus                   GCTCGTCCCCAGCGAGATCTTCCCGCTGGAGATCAGGTCGGCGGGGCAGAGCATCACCGT
                                      1270      1280      1290      1300      1310      1320
Lr67(sus)                   GCTCATCCCCAGCGAGACGTTCCCGCTGGAGACGCGGTCGGCGGGGCAGAGCGTGACGGT  1280
Lr67(res)                   GCTCATCCCCAGCGAGACGTTCCCGCTGGAGACGCGGTCGGCGGGGCAGAGCGTGACGGT  1280
ScLr_SUG4 (Lo7)             GCTCGTCCCCAGCGAGATCTTCCCGCTGGAGATCAGGTCGGCGGGGCAGAGCATCACCGT  1292
ScLr_SUG4_118_DANKO_APR     GCTCGTCCCCAGCGAGATCTTCCCGCTGGAGATCAGGTCGGCGGGGCAGAGCATCACCGT  1177
ScLr_SUG4_120_DANKO_APR     GCTCGTCCCCAGCGAGATCTTCCCGCTGGAGATCAGGTCGGCGGGGCAGAGCATCACCGT  1164
ScLr_SUG4_71_PHR_APR        GCTCGTCCCCAGCGAGATCTTCCCGCTGGAGATCAGGTCGGCGGGGCAGAGCATCACCGT  1171
ScLr_SUG4_149_PHR_APR       GCTCGTCCCCAGCGAGATCTTCCCGCTGGAGATCAGGTCGGCGGGGCAGAGCATCACCGT  1177
ScLr_SUG4_59_DANKO_non-APR  GCTCGTCCCCAGCGAGATCTTCCCGCTGGAGATCAGGTCGGCGGGGCAGAGCATCACCGT  1175
ScLr_SUG4_61_DANKO_non-APR  GCTCGTCCCCAGCGAGATCTTCCCGCTGGAGATCAGGTCGGCGGGGCAGAGCATCACCGT  1175
ScLr_SUG4_88_PHR_non-APR    GCTCGTCCCCAGCGAGATCTTCCCGCTGGAGATCAGGTCGGCGGGGCAGAGCATCACCGT  1176
ScLr_SUG4_105_PHR_non-APR   GCTCGTCCCCAGCGAGATCTTCCCGCTGGAGATCAGGTCGGCGGGGCAGAGCATCACCGT  1186
```

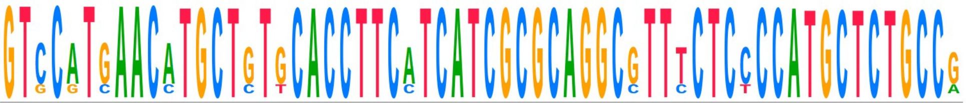

```
Consensus                   GTCCATGAACATGCTGTGCACCTTCATCATCGCGCAGGCGTTTCTCCCCATGCTCTGCCG
                                      1330      1340      1350      1360      1370      1380
Lr67(sus)                   GTGCGTCAACCTGCTCTTCACCTTCCTCATCGCGCAGGCCTTCCTCTCCATGCTCTGCCA  1340
Lr67(res)                   GTGCGTCAACCTGCTCTTCACCTTCCTCATCGCGCAGGCCTTCCTCTCCATGCTCTGCCA  1340
ScLr_SUG4 (Lo7)             GTCCATGAACATGCTGTGCACCTTCATCATCGCGCAGGCGTTTCTCCCCATGCTCTGCCG  1352
ScLr_SUG4_118_DANKO_APR     GTCCATGAACATGCTGTGCACCTTCATCATCGCGCAGGCGTTTCTCCCCATGCTCTGCCG  1237
ScLr_SUG4_120_DANKO_APR     GTCCATGAACATGCTGTGCACCTTCATCATCGCGCAGGCGTTTCTCCCCATGCTCTGCCG  1224
ScLr_SUG4_71_PHR_APR        GTCCATGAACA-------------------------------------------------  1182
ScLr_SUG4_149_PHR_APR       GTCCATGAACATGCTGTGCACCTTCATCATCGCGCAGGCGTTTCTCCCCATGCTCTGCCG  1237
ScLr_SUG4_59_DANKO_non-APR  GTCCATGAACATGCTGTGCACCTTCATCATCGCGCAGGCGTTTCTCCCCA----------  1225
ScLr_SUG4_61_DANKO_non-APR  GTCCATGAACATGCTGTGCACCTTCATCATCGCGCAGGCGTTTCTCCCCATGCTCTGCCG  1235
ScLr_SUG4_88_PHR_non-APR    GTCCATGAACATGCTGTGCACCTTCATCATCGCGCAGGCGTTTCTCCCCATGCTCTGCCG  1236
ScLr_SUG4_105_PHR_non-APR   GTCCATGAACATGCTGTGCACCTTCATCATCGCGCAGGCGTTTCTCCCCATGCTCTGCCG  1246
```

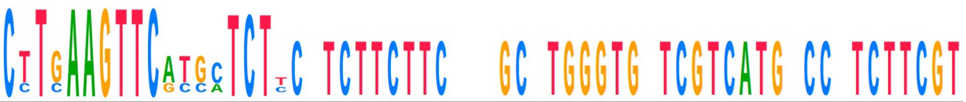

```
Consensus                   CTTGAAGTTCATGNTCT-------------------------------------------
                                      1390      1400      1410      1420      1430      1440
Lr67(sus)                   CCTCAAGTTCGCCATCTTCATCTTCTTCTCGGCCTGGGTGCTCGTCATGTCCGTCTTCGT  1400
Lr67(res)                   CCTCAAGTTCGCCATCTTCATCTTCTTCTCGGCCTGGGTGCTCGTCATGTCCGTCTTCGT  1400
ScLr_SUG4 (Lo7)             CTTGAAGTTCATGCTCTTCTTCTTCTTCGGCGCGTGGGTGATCGTCATGACCCTCTTCGT  1412
ScLr_SUG4_118_DANKO_APR     CTTGAAGTTCATG-----------------------------------------------  1250
ScLr_SUG4_120_DANKO_APR     CTTGAAGTTCATGCTCTC------------------------------------------  1242
ScLr_SUG4_71_PHR_APR        ------------------------------------------------------------  1182
ScLr_SUG4_149_PHR_APR       CTTGAAGTTCATGCTCTC------------------------------------------  1255
ScLr_SUG4_59_DANKO_non-APR  ------------------------------------------------------------  1225
ScLr_SUG4_61_DANKO_non-APR  CTTGAAGTTC--------------------------------------------------  1245
ScLr_SUG4_88_PHR_non-APR    CTTGAAGTTCATGCTCT-------------------------------------------  1253
ScLr_SUG4_105_PHR_non-APR   CTTGAAGTTCATGCTCT-------------------------------------------  1263
```

```
                              CTTCTTCCT CCGGAGACCAAGAACGTGCCCATCGAGGAGATG     CAAGGTGTG
Consensus                  ------------------------------------------------------------
                                    1450      1460      1470      1480      1490      1500
Lr67(sus)                  GCTCTTCTTCCTCCCGGAGACCAAGAACGTGCCCATCGAGGAGATGACCGACAAGGTGTG  1460
Lr67(res)                  GCTCTTCTTCCTCCCGGAGACCAAGAACGTGCCCATCGAGGAGATGACCGACAAGGTGTG  1460
ScLr_SUG4 (Lo7)            TGCCTTCTTCCTGCCGGAGACCAAGAACGTGCCCATCGAGGAGATGGTGCTC---GTGTG  1469
ScLr_SUG4_118_DANKO_APR    ------------------------------------------------------------  1250
ScLr_SUG4_120_DANKO_APR    ------------------------------------------------------------  1242
ScLr_SUG4_71_PHR_APR       ------------------------------------------------------------  1182
ScLr_SUG4_149_PHR_APR      ------------------------------------------------------------  1255
ScLr_SUG4_59_DANKO_non-APR ------------------------------------------------------------  1225
ScLr_SUG4_61_DANKO_non-APR ------------------------------------------------------------  1245
ScLr_SUG4_88_PHR_non-APR   ------------------------------------------------------------  1253
ScLr_SUG4_105_PHR_non-APR  ------------------------------------------------------------  1263

                           GAAG  GCACTGGT CTGGA   G TTCAT   CGACGA GAC   CAC  C  C  CG
Consensus                  ------------------------------------------------------------
                                    1510      1520      1530      1540      1550      1560
Lr67(sus)                  GAAGCAGCACTGGTTCTGGAAGAGATTCATGGACGACGACGACCACCACCACAACATCG-  1519
Lr67(res)                  GAAGCAGCACTGGTTCTGGAAGAGATTCATGGACGACGACGACCACCACCACAACATCG-  1519
ScLr_SUG4 (Lo7)            GAAGGCGCACTGGTACTGGAGCCGCTTCATCCGCGACGAGGACGTGCACGTCGGCGGCGG  1529
ScLr_SUG4_118_DANKO_APR    ------------------------------------------------------------  1250
ScLr_SUG4_120_DANKO_APR    ------------------------------------------------------------  1242
ScLr_SUG4_71_PHR_APR       ------------------------------------------------------------  1182
ScLr_SUG4_149_PHR_APR      ------------------------------------------------------------  1255
ScLr_SUG4_59_DANKO_non-APR ------------------------------------------------------------  1225
ScLr_SUG4_61_DANKO_non-APR ------------------------------------------------------------  1245
ScLr_SUG4_88_PHR_non-APR   ------------------------------------------------------------  1253
ScLr_SUG4_105_PHR_non-APR  ------------------------------------------------------------  1263

                                               CCAACGGCAAG  C    C G C         TGA
Consensus                  -------------------------------------------------------
                                    1570      1580      1590      1600      1610
Lr67(sus)                  --------------------CCAACGGCAAGAACGCCACCGTC---------TGA  1545
Lr67(res)                  --------------------CCAACGGCAAGAACGCCACCGTC---------TGA  1545
ScLr_SUG4 (Lo7)            CGCAGACGTCGAGATGCGCTCCAACGGCAAGGTCCAGGCTGCCAAGCTCCCGTGA  1584
ScLr_SUG4_118_DANKO_APR    -------------------------------------------------------  1250
ScLr_SUG4_120_DANKO_APR    -------------------------------------------------------  1242
ScLr_SUG4_71_PHR_APR       -------------------------------------------------------  1182
ScLr_SUG4_149_PHR_APR      -------------------------------------------------------  1255
ScLr_SUG4_59_DANKO_non-APR -------------------------------------------------------  1225
ScLr_SUG4_61_DANKO_non-APR -------------------------------------------------------  1245
ScLr_SUG4_88_PHR_non-APR   -------------------------------------------------------  1253
ScLr_SUG4_105_PHR_non-APR  -------------------------------------------------------  1263
```

Sequence Logo: 50% GC base composition

Consensus Threshold: >50%

Colors: 4-color highlighting

Created: 26 lis 2024

Last Modified: 26 lis 2024
