# Supplementary material for: Searching for genes determining the APR phenotype in rye
Source: BMC Plant Biol. 2025 Jul 19;25:935. doi: 10.1186/s12870-025-06920-0 (PMC12275401; doi:10.1186/s12870-025-06920-0)
Supplement: Supplementary file 9 — Supplementary Material 9. [file 12870_2025_6920_MOESM9_ESM.pdf]

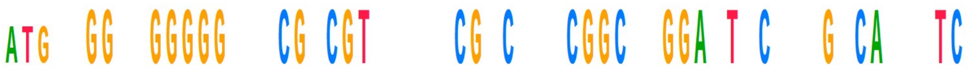

```
Consensus                   ------------------------------------------------------------
                                     10        20        30        40        50        60
Lr67(sus)                   ATGCCGGGCGGGGGGTTCGCCGTGTCGGCGCCGTCCGGCGTGGAGTTCGAGGCCAAGATC  60
Lr67(res)                   ATGCCGGGCGGGGGGTTCGCCGTGTCGGCGCCGTCCGGCGTGGAGTTCGAGGCCAAGATC  60
ScLr_SUG6 (Lo7)             ---ATGGCGGGGGGCGCCGTCGTCAACACGTCCGGCGGCAAGGACTACCCCGGCAGACTC  57
ScLr_SUG6_118_DANKO_APR     ------------------------------------------------------------
ScLr_SUG6_120_DANKO_APR     ------------------------------------------------------------
ScLr_SUG6_71_PHR_APR        ------------------------------------------------------------
ScLr_SUG6_149_PHR_APR       ------------------------------------------------------------
ScLr_SUG6_59_DANKO_non-APR  ------------------------------------------------------------
ScLr_SUG6_61_DANKO_non-APR  ------------------------------------------------------------
ScLr_SUG6_88_PHR_non-APR    ------------------------------------------------------------
ScLr_SUG6_105_PHR_non-APR   ------------------------------------------------------------
```

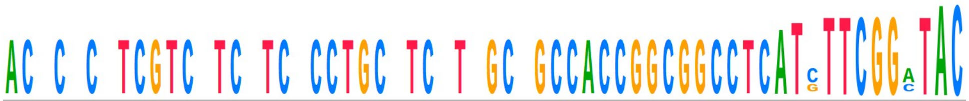

```
Consensus                   --------------------------------------------------NTTCGGNTAC
                                     70        80        90       100       110       120
Lr67(sus)                   ACGCCCATCGTCATCATCTCCTGCATCATGGCGGCCACCGGCGGCCTCATGTTCGGCTAC  120
Lr67(res)                   ACGCCCATCGTCATCATCTCCTGCATCATGGCGGCCACCGGCGGCCTCATGTTCGGCTAC  120
ScLr_SUG6 (Lo7)             ACCCTCTTCGTCTTCTTCACCTGCGTCGTCGCCGCCACCGGCGGCCTCATCTTCGGATAC  117
ScLr_SUG6_118_DANKO_APR     --------------------------------------------------CTTCGGATAC  10
ScLr_SUG6_120_DANKO_APR     ------------------------------------------------------------
ScLr_SUG6_71_PHR_APR        ------------------------------------------------ATCTTCGGATAC  12
ScLr_SUG6_149_PHR_APR       ----------------------------------------------------------AC  2
ScLr_SUG6_59_DANKO_non-APR  ----------------------------------------------------------AC  2
ScLr_SUG6_61_DANKO_non-APR  ----------------------------------------------------------AC  2
ScLr_SUG6_88_PHR_non-APR    ----------------------------------------------------------AC  2
ScLr_SUG6_105_PHR_non-APR   -------------------------------------------------TCTTCGGATAC  11
```

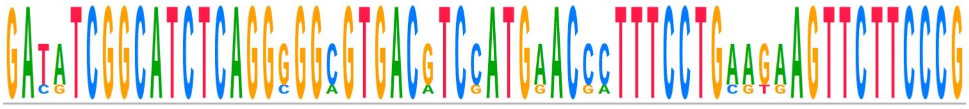

```
Consensus                   GATATCGGCATCTCAGGGGGCGTGACGTCCATGAACCCTTTCCTGAAGAAGTTCTTCCCG
                                    130       140       150       160       170       180
Lr67(sus)                   GACGTCGGCATCTCAGGCGGAGTGACATCGATGGACGATTTCCTGCGTGAGTTCTTCCCG  180
Lr67(res)                   GACGTCGGCATCTCAGGCGGAGTGACATCGATGGACGATTTCCTGCGTGAGTTCTTCCCG  180
ScLr_SUG6 (Lo7)             GATATCGGCATCTCAGGGGGCGTGACGTCCATGAACCCTTTCCTGAAGAAGTTCTTCCCG  177
ScLr_SUG6_118_DANKO_APR     GATATCGGCATCTCAGGGGGCGTGACGTCCATGAACCCTTTCCTGAAGAAGTTCTTCCCG  70
ScLr_SUG6_120_DANKO_APR     -ATATCGGCATCTCAGGGGGCGTGACGTCCATGAACCCTTTCCTGAAGAAGTTCTTCCCG  59
ScLr_SUG6_71_PHR_APR        GATATCGGCATCTCAGGGGGCGTGACGTCCATGAACCCTTTCCTGAAGAAGTTCTTCCCG  72
ScLr_SUG6_149_PHR_APR       GATATCGGCATCTCAGGGGGCGTGACGTCCATGAACCCTTTCCTGAAGAAGTTCTTCCCG  62
ScLr_SUG6_59_DANKO_non-APR  GATATCGGCATCTCAGGGGGCGTGACGTCCATGAACCCTTTCCTGAAGAAGTTCTTCCCG  62
ScLr_SUG6_61_DANKO_non-APR  GATATCGGCATCTCAGGGGGCGTGACGTCCATGAACCCTTTCCTGAAGAAGTTCTTCCCG  62
ScLr_SUG6_88_PHR_non-APR    GATATCGGCATCTCAGGGGGCGTGACGTCCATGAACCCTTTCCTGAAGAAGTTCTTCCCG  62
ScLr_SUG6_105_PHR_non-APR   GATATCGGCATCTCAGGGGGCGTGACGTCCATGAACCCTTTCCTGAAGAAGTTCTTCCCG  71
```

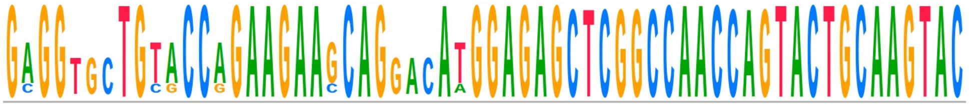

Sequence	190–240	End
**Consensus**	GAGG---TGTACCAGAAGAAGCAG---ATGGAGAGCTCGGCCAACCAGTACTGCAAGTAC	
Lr67(sus)	GCGGTGCTGCGCCGGAAGAACCAGGACAAGGAGAG------CAAC---TACTGCAAGTAC	231
Lr67(res)	GCGGTGCTGCGCCGGAAGAACCAGGACAAGGAGAG------CAAC---TACTGCAAGTAC	231
ScLr_SUG6 (Lo7)	GAGG---TGTACCAGAAGAAGCAG---ATGGAGAGCTCGGCCAACCAGTACTGCAAGTAC	231
ScLr_SUG6_118_DANKO_APR	GAGG---TGTACCAGAAGAAGCAG---ATGGAGAGCTCGGCCAACCAGTACTGCAAGTAC	124
ScLr_SUG6_120_DANKO_APR	GAGG---TGTACCAGAAGAAGCAG---ATGGAGAGCTCGGCCAACCAGTACTGCAAGTAC	113
ScLr_SUG6_71_PHR_APR	GAGG---TGTACCAGAAGAAGCAG---ATGGAGAGCTCGGCCAACCAGTACTGCAAGTAC	126
ScLr_SUG6_149_PHR_APR	GAGG---TGTACCAGAAGAAGCAG---ATGGAGAGCTCGGCCAACCAGTACTGCAAGTAC	116
ScLr_SUG6_59_DANKO_non-APR	GAGG---TGTACCAGAAGAAGCAG---ATGGAGAGCTCGGCCAACCAGTACTGCAAGTAC	116
ScLr_SUG6_61_DANKO_non-APR	GAGG---TGTACCAGAAGAAGCAG---ATGGAGAGCTCGGCCAACCAGTACTGCAAGTAC	116
ScLr_SUG6_88_PHR_non-APR	GAGG---TGTACCAGAAGAAGCAG---ATGGAGAGCTCGGCCAACCAGTACTGCAAGTAC	116
ScLr_SUG6_105_PHR_non-APR	GAGG---TGTACCAGAAGAAGCAG---ATGGAGAGCTCGGCCAACCAGTACTGCAAGTAC	125

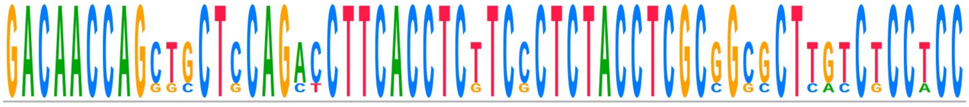

Sequence	250–300	End
**Consensus**	GACAACCAGCTGCTCCAGACCTTCACCTCTTCCCTCTACCTCGCGGCGCTTGTCTCCTCC	
Lr67(sus)	GACAACCAGGGCCTGCAGCTCTTCACCTCGTCGCTCTACCTCGCCGGCCTCACCGCCACC	291
Lr67(res)	GACAACCAGGGCCTGCAGCTCTTCACCTCGTCGCTCTACCTCGCCGGCCTCACCGCCACC	291
ScLr_SUG6 (Lo7)	GACAACCAGCTGCTCCAGACCTTCACCTCTTCCCTCTACCTCGCGGCGCTTGTCTCCTCC	291
ScLr_SUG6_118_DANKO_APR	GACAACCAGCTGCTCCAGACCTTCACCTCTTCCCTCTACCTCGCGGCGCTTGTCTCCTCC	184
ScLr_SUG6_120_DANKO_APR	GACAACCAGCTGCTCCAGACCTTCACCTCTTCCCTCTACCTCGCGGCGCTTGTCTCCTCC	173
ScLr_SUG6_71_PHR_APR	GACAACCAGCTGCTCCAGACCTTCACCTCTTCCCTCTACCTCGCGGCGCTTGTCTCCTCC	186
ScLr_SUG6_149_PHR_APR	GACAACCAGCTGCTCCAGACCTTCACCTCTTCCCTCTACCTCGCGGCGCTTGTCTCCTCC	176
ScLr_SUG6_59_DANKO_non-APR	GACAACCAGCTGCTCCAGACCTTCACCTCTTCCCTCTACCTCGCGGCGCTTGTCTCCTCC	176
ScLr_SUG6_61_DANKO_non-APR	GACAACCAGCTGCTCCAGACCTTCACCTCTTCCCTCTACCTCGCGGCGCTTGTCTCCTCC	176
ScLr_SUG6_88_PHR_non-APR	GACAACCAGCTGCTCCAGACCTTCACCTCTTCCCTCTACCTCGCGGCGCTTGTCTCCTCC	176
ScLr_SUG6_105_PHR_non-APR	GACAACCAGCTGCTCCAGACCTTCACCTCTTCCCTCTACCTCGCGGCGCTTGTCTCCTCC	185

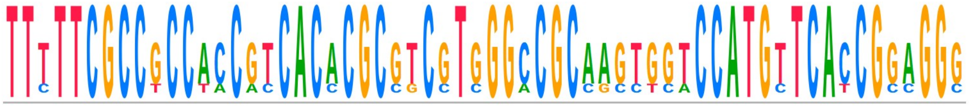

Sequence	310–360	End
**Consensus**	TTTTTCGCCGCCACCGTCACACGCGTCGTGGGCCGCAAGTGGTCCATGTTCACCGGAGGG	
Lr67(sus)	TTCTTCGCCTCCTACACCACCCGCCGCCTCGGACGCCGCCTCACCATGCTCATCGCCGGC	351
Lr67(res)	TTCTTCGCCTCCTACACCACCCGCCGCCTCGGACGCCGCCTCACCATGCTCATCGCCGGC	351
ScLr_SUG6 (Lo7)	TTTTTCGCCGCCACCGTCACACGCGTCGTGGGCCGCAAGTGGTCCATGTTCACCGGAGGG	351
ScLr_SUG6_118_DANKO_APR	TTTTTCGCCGCCACCGTCACACGCGTCGTGGGCCGCAAGTGGTCCATGTTCACCGGAGGG	244
ScLr_SUG6_120_DANKO_APR	TTTTTCGCCGCCACCGTCACACGCGTCGTGGGCCGCAAGTGGTCCATGTTCACCGGAGGG	233
ScLr_SUG6_71_PHR_APR	TTTTTCGCCGCCACCGTCACACGCGTCGTGGGCCGCAAGTGGTCCATGTTCACCGGAGGG	246
ScLr_SUG6_149_PHR_APR	TTTTTCGCCGCCACCGTCACACGCGTCGTGGGCCGCAAGTGGTCCATGTTCACCGGAGGG	236
ScLr_SUG6_59_DANKO_non-APR	TTTTTCGCCGCCACCGTCACACGCGTCGTGGGCCGCAAGTGGTCCATGTTCACCGGAGGG	236
ScLr_SUG6_61_DANKO_non-APR	TTTTTCGCCGCCACCGTCACACGCGTCGTGGGCCGCAAGTGGTCCATGTTCACCGGAGGG	236
ScLr_SUG6_88_PHR_non-APR	TTTTTCGCCGCCACCGTCACACGCGTCGTGGGCCGCAAGTGGTCCATGTTCACCGGAGGG	236
ScLr_SUG6_105_PHR_non-APR	TTTTTCGCCGCCACCGTCACACGCGTCGTGGGCCGCAAGTGGTCCATGTTCACCGGAGGG	245

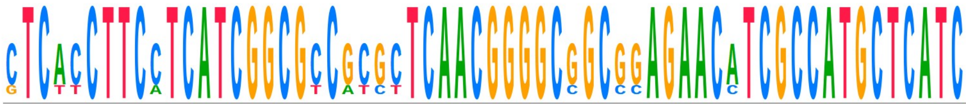

Sequence	361–420	End
**Consensus**	CTCACCTTCCTCATCGGCGCCGCGCTCAACGGGGCGGCGGAGAACATCGCCATGCTCATC	
	370 380 390 400 410 420	
Lr67(sus)	GTCTTCTTCATCATCGGCGTCATCTTCAACGGGGCCGCCCAGAACCTCGCCATGCTCATC	411
Lr67(res)	GTCTTCTTCATCATCGGCGTCATCTTCAACGGGGCCGCCCAGAACCTCGCCATGCTCATC	411
ScLr_SUG6 (Lo7)	CTCACCTTCCTCATCGGCGCCGCGCTCAACGGGGCGGCGGAGAACATCGCCATGCTCATC	411
ScLr_SUG6_118_DANKO_APR	CTCACCTTCCTCATCGGCGCCGCGCTCAACGGGGCGGCGGAGAACATCGCCATGCTCATC	304
ScLr_SUG6_120_DANKO_APR	CTCACCTTCCTCATCGGCGCCGCGCTCAACGGGGCGGCGGAGAACATCGCCATGCTCATC	293
ScLr_SUG6_71_PHR_APR	CTCACCTTCCTCATCGGCGCCGCGCTCAACGGGGCGGCGGAGAACATCGCCATGCTCATC	306
ScLr_SUG6_149_PHR_APR	CTCACCTTCCTCATCGGCGCCGCGCTCAACGGGGCGGCGGAGAACATCGCCATGCTCATC	296
ScLr_SUG6_59_DANKO_non-APR	CTCACCTTCCTCATCGGCGCCGCGCTCAACGGGGCGGCGGAGAACATCGCCATGCTCATC	296
ScLr_SUG6_61_DANKO_non-APR	CTCACCTTCCTCATCGGCGCCGCGCTCAACGGGGCGGCGGAGAACATCGCCATGCTCATC	296
ScLr_SUG6_88_PHR_non-APR	CTCACCTTCCTCATCGGCGCCGCGCTCAACGGGGCGGCGGAGAACATCGCCATGCTCATC	296
ScLr_SUG6_105_PHR_non-APR	CTCACCTTCCTCATCGGCGCCGCGCTCAACGGGGCGGCGGAGAACATCGCCATGCTCATC	305

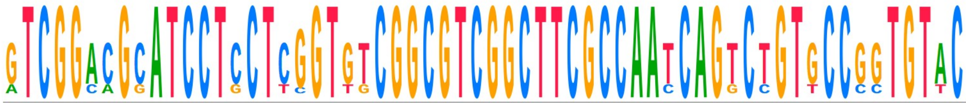

Sequence	421–480	End
**Consensus**	GTCGGACGCATCCTCCTCGGTGTCGGCGTCGGCTTCGCCAATCAGTCTGTGCCGGTGTAC	
	430 440 450 460 470 480	
Lr67(sus)	ATCGGCAGGATCCTGCTTGGTTGCGGCGTCGGCTTCGCCAACCAGGCCGTTCCCCTGTTC	471
Lr67(res)	ATCGGCAGGATCCTGCTTCGTTGCGGCGTCGGCTTCGCCAACCAGGCCGTTCCCCTGTTC	471
ScLr_SUG6 (Lo7)	GTCGGACGCATCCTCCTCGGTGTCGGCGTCGGCTTCGCCAATCAGTCTGTGCCGGTGTAC	471
ScLr_SUG6_118_DANKO_APR	GTCGGACGCATCCTCCTCGGTGTCGGCGTCGGCTTCGCCAATCAGTCTGTGCCGGTGTAC	364
ScLr_SUG6_120_DANKO_APR	GTCGGACGCATCCTCCTCGGTGTCGGCGTCGGCTTCGCCAATCAGTCTGTGCCGGTGTAC	353
ScLr_SUG6_71_PHR_APR	GTCGGACGCATCCTCCTCGGTGTCGGCGTCGGCTTCGCCAATCAGTCTGTGCCGGTGTAC	366
ScLr_SUG6_149_PHR_APR	GTCGGACGCATCCTCCTCGGTGTCGGCGTCGGCTTCGCCAATCAGTCTGTGCCGGTGTAC	356
ScLr_SUG6_59_DANKO_non-APR	GTCGGACGCATCCTCCTCGGTGTCGGCGTCGGCTTCGCCAATCAGTCTGTGCCGGTGTAC	356
ScLr_SUG6_61_DANKO_non-APR	GTCGGACGCATCCTCCTCGGTGTCGGCGTCGGCTTCGCCAATCAGTCTGTGCCGGTGTAC	356
ScLr_SUG6_88_PHR_non-APR	GTCGGACGCATCCTCCTCGGTGTCGGCGTCGGCTTCGCCAATCAGTCTGTGCCGGTGTAC	356
ScLr_SUG6_105_PHR_non-APR	GTCGGACGCATCCTCCTCGGTGTCGGCGTCGGCTTCGCCAATCAGTCTGTGCCGGTGTAC	365

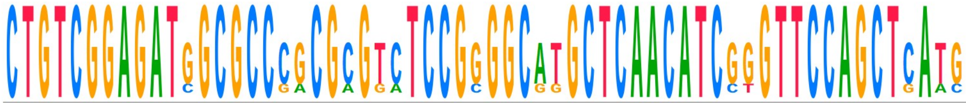

Sequence	481–540	End
**Consensus**	CTGTCGGAGATGGCGCCCGCGCGTCTCCGGGGCATGCTCAACATCGGGTTCCAGCTCATG	
	490 500 510 520 530 540	
Lr67(sus)	CTGTCGGAGATCGCGCCGACGAGGATCCGCGGCGGGCTCAACATCCTGTTCCAGCTGAAC	531
Lr67(res)	CTGTCGGAGATCGCGCCGACGAGGATCCGCGGCGGGCTCAACATCCTGTTCCAGCTGAAC	531
ScLr_SUG6 (Lo7)	CTGTCGGAGATGGCGCCCGCGCGTCTCCGGGGCATGCTCAACATCGGGTTCCAGCTCATG	531
ScLr_SUG6_118_DANKO_APR	CTGTCGGAGATGGCGCCCGCGCGTCTCCGGGGCATGCTCAACATCGGGTTCCAGCTCATG	424
ScLr_SUG6_120_DANKO_APR	CTGTCGGAGATGGCGCCCGCGCGTCTCCGGGGCATGCTCAACATCGGGTTCCAGCTCATG	413
ScLr_SUG6_71_PHR_APR	CTGTCGGAGATGGCGCCCGCGCGTCTCCGGGGCATGCTCAACATCGGGTTCCAGCTCATG	426
ScLr_SUG6_149_PHR_APR	CTGTCGGAGATGGCGCCCGCGCGTCTCCGGGGCATGCTCAACATCGGGTTCCAGCTCATG	416
ScLr_SUG6_59_DANKO_non-APR	CTGTCGGAGATGGCGCCCGCGCGTCTCCGGGGCATGCTCAACATCGGGTTCCAGCTCATG	416
ScLr_SUG6_61_DANKO_non-APR	CTGTCGGAGATGGCGCCCGCGCGTCTCCGGGGCATGCTCAACATCGGGTTCCAGCTCATG	416
ScLr_SUG6_88_PHR_non-APR	CTGTCGGAGATGGCGCCCGCGCGTCTCCGGGGCATGCTCAACATCGGGTTCCAGCTCATG	416
ScLr_SUG6_105_PHR_non-APR	CTGTCGGAGATGGCGCCCGCGCGTCTCCGGGGCATGCTCAACATCGGGTTCCAGCTCATG	425

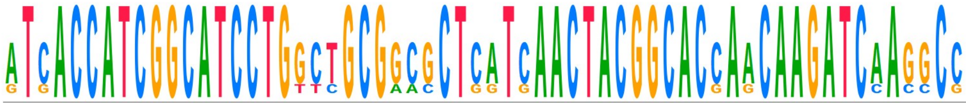

```
Consensus                    ATCACCATCGGCATCCTGGCTGCGGCGCTCATCAACTACGGCACCAACAAGATCAAGGCC
                                      550       560       570       580       590       600
Lr67(sus)                    GTGACCATCGGCATCCTGTTCGCGAACCTGGTGAACTACGGCACGAGCAAGATCCACCCG 591
Lr67(res)                    GTGACCATCGGCATCCTGTTCGCGAACCTGGTGAACTACGGCACGAGCAAGATCCACCCG 591
ScLr_SUG6 (Lo7)              ATCACCATCGGCATCCTGGCTGCGGCGCTCATCAACTACGGCACCAACAAGATCAAGGCC 591
ScLr_SUG6_118_DANKO_APR      ATCACCATCGGCATCCTGGCTGCGGCGCTCATCAACTACGGCACCAACAAGATCAAGGCC 484
ScLr_SUG6_120_DANKO_APR      ATCACCATCGGCATCCTGGCTGCGGCGCTCATCAACTACGGCACCAACAAGATCAAGGCC 473
ScLr_SUG6_71_PHR_APR         ATCACCATCGGCATCCTGGCTGCGGCGCTCATCAACTACGGCACCAACAAGATCAAGGCC 486
ScLr_SUG6_149_PHR_APR        ATCACCATCGGCATCCTGGCTGCGGCGCTCATCAACTACGGCACCAACAAGATCAAGGCC 476
ScLr_SUG6_59_DANKO_non-APR   ATCACCATCGGCATCCTGGCTGCGGCGCTCATCAACTACGGCACCAACAAGATCAAGGCC 476
ScLr_SUG6_61_DANKO_non-APR   ATCACCATCGGCATCCTGGCTGCGGCGCTCATCAACTACGGCACCAACAAGATCAAGGCC 476
ScLr_SUG6_88_PHR_non-APR     ATCACCATCGGCATCCTGGCTGCGGCGCTCATCAACTACGGCACCAACAAGATCAAGGCC 476
ScLr_SUG6_105_PHR_non-APR    ATCACCATCGGCATCCTGGCTGCGGCGCTCATCAACTACGGCACCAACAAGATCAAGGCC 485
```

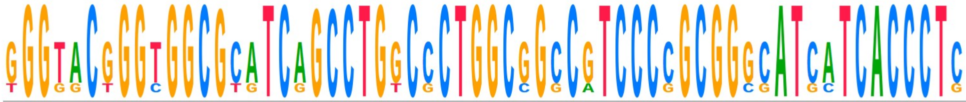

```
Consensus                    GGGTACGGGTGGCGCATCAGCCTGGCCCTGGCGGCCGTCCCCGCGGGCATCATCACCCTC
                                      610       620       630       640       650       660
Lr67(sus)                    TGGGGCTGGCGGC-TGTCG--CTGTCGCTGGCCGGCATCCCGGCGGCGATGCTCACCCTG 648
Lr67(res)                    TGGGGCTGGCGGC-TGTCG--CTGTCGCTGGCCGGCATCCCGGCGGCGATGCTCACCCTG 648
ScLr_SUG6 (Lo7)              GGGTACGGGTGGCGCATCAGCCTGGCCCTGGCGGCCGTCCCCGCGGGCATCATCACCCTC 651
ScLr_SUG6_118_DANKO_APR      GGGTACGGGTGGCGCATCAGCCTGGCCCTGGCGGCCGTCCCCGCGGGCATCATCACCCTC 544
ScLr_SUG6_120_DANKO_APR      GGGTACGGGTGGCGCATCAGCCTGGCCCTGGCGGCCGTCCCCGCGGGCATCATCACCCTC 533
ScLr_SUG6_71_PHR_APR         GGGTACGGGTGGCGCATCAGCCTGGCCCTGGCGGCCGTCCCCGCGGGCATCATCACCCTC 546
ScLr_SUG6_149_PHR_APR        GGGTACGGGTGGCGCATCAGCCTGGCCCTGGCGGCCGTCCCCGCGGGCATCATCACCCTC 536
ScLr_SUG6_59_DANKO_non-APR   GGGTACGGGTGGCGCATCAGCCTGGCCCTGGCGGCCGTCCCCGCGGGCATCATCACCCTC 536
ScLr_SUG6_61_DANKO_non-APR   GGGTACGGGTGGCGCATCAGCCTGGCCCTGGCGGCCGTCCCCGCGGGCATCATCACCCTC 536
ScLr_SUG6_88_PHR_non-APR     GGGTACGGGTGGCGCATCAGCCTGGCCCTGGCGGCCGTCCCCGCGGGCATCATCACCCTC 536
ScLr_SUG6_105_PHR_non-APR    GGGTACGGGTGGCGCATCAGCCTGGCCCTGGCGGCCGTCCCCGCGGGCATCATCACCCTC 545
```

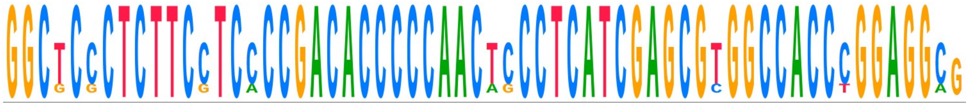

```
Consensus                    GGCTCCCTCTTCCTCCCCGACACCCCCAACTCCCTCATCGAGCGTGGCCACCCGGAGGC-
                                      670       680       690       700       710       720
Lr67(sus)                    GGCGCGCTCTTCGTCACCGACACCCCCAACAGCCTCATCGAGCGCGGCCACCTGGAGGAG 708
Lr67(res)                    GGCGCGCTCTTCGTCACCGACACCCCCAACAGCCTCATCGAGCGCGGCCACCTGGAGGAG 708
ScLr_SUG6 (Lo7)              GGCTCCCTCTTCCTCCCCGACACCCCCAACTCCCTCATCGAGCGTGGCCACCCGGAGGC- 710
ScLr_SUG6_118_DANKO_APR      GGCTCCCTCTTCCTCCCCGACACCCCCAACTCCCTCATCGAGCGTGGCCACCCGGAGGC- 603
ScLr_SUG6_120_DANKO_APR      GGCTCCCTCTTCCTCCCCGACACCCCCAACTCCCTCATCGAGCGTGGCCACCCGGAGGC- 592
ScLr_SUG6_71_PHR_APR         GGCTCCCTCTTCCTCCCCGACACCCCCAACTCCCTCATCGAGCGTGGCCACCCGGAGGC- 605
ScLr_SUG6_149_PHR_APR        GGCTCCCTCTTCCTCCCCGACACCCCCAACTCCCTCATCGAGCGTGGCCACCCGGAGGC- 595
ScLr_SUG6_59_DANKO_non-APR   GGCTCCCTCTTCCTCCCCGACACCCCCAACTCCCTCATCGAGCGTGGCCACCCGGAGGC- 595
ScLr_SUG6_61_DANKO_non-APR   GGCTCCCTCTTCCTCCCCGACACCCCCAACTCCCTCATCGAGCGTGGCCACCCGGAGGC- 595
ScLr_SUG6_88_PHR_non-APR     GGCTCCCTCTTCCTCCCCGACACCCCCAACTCCCTCATCGAGCGTGGCCACCCGGAGGC- 595
ScLr_SUG6_105_PHR_non-APR    GGCTCCCTCTTCCTCCCCGACACCCCCAACTCCCTCATCGAGCGTGGCCACCCGGAGGC- 604
```

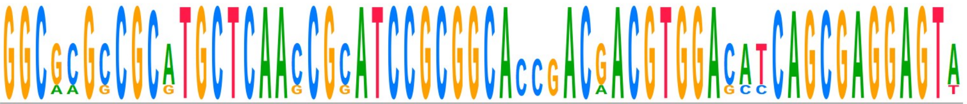

```
Consensus                 GGCGCGCCGCATGCTCAACCGCATCCGCGGCA---ACGACGTGGACATCAGCGAGGAGTA
                                 730       740       750       760       770       780
Lr67(sus)                 GGCAAGGCG-GTGCTCAAGCGGATCCGCGGCACCGACAACGTGGAGCC------GGAGTT  761
Lr67(res)                 GGCAAGGCG-GTGCTCAAGCGGATCCGCGGCACCGACAACGTGGAGCC------GGAGTT  761
ScLr_SUG6 (Lo7)           GGCGCGCCGCATGCTCAACCGCATCCGCGGCA---ACGACGTGGACATCAGCGAGGAGTA  767
ScLr_SUG6_118_DANKO_APR   GGCGCGCCGCATGCTCAACCGCATCCGCGGCA---ACGACGTGGACATCAGCGAGGAGTA  660
ScLr_SUG6_120_DANKO_APR   GGCGCGCCGCATGCTCAACCGCATCCGCGGCA---ACGACGTGGACATCAGCGAGGAGTA  649
ScLr_SUG6_71_PHR_APR      GGCGCGCCGCATGCTCAACCGCATCCGCGGCA---ACGACGTGGACATCAGCGAGGAGTA  662
ScLr_SUG6_149_PHR_APR     GGCGCGCCGCATGCTCAACCGCATCCGCGGCA---ACGACGTGGACATCAGCGAGGAGTA  652
ScLr_SUG6_59_DANKO_non-APR GGCGCGCCGCATGCTCAACCGCATCCGCGGCA---ACGACGTGGACATCAGCGAGGAGTA  652
ScLr_SUG6_61_DANKO_non-APR GGCGCGCCGCATGCTCAACCGCATCCGCGGCA---ACGACGTGGACATCAGCGAGGAGTA  652
ScLr_SUG6_88_PHR_non-APR  GGCGCGCCGCATGCTCAACCGCATCCGCGGCA---ACGACGTGGACATCAGCGAGGAGTA  652
ScLr_SUG6_105_PHR_non-APR GGCGCGCCGCATGCTCAACCGCATCCGCGGCA---ACGACGTGGACATCAGCGAGGAGTA  661
```

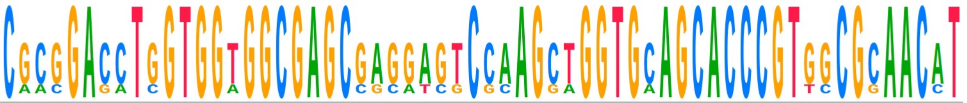

```
Consensus                 CGCGGACCTGGTGGTGGCGAGCGAGGAGTCCAAGCTGGTGCAGCACCCGTGGCGCAACAT
                                 790       800       810       820       830       840
Lr67(sus)                 CAACGAGATCGTGGAGGCGAGCCGCATCGCGCAGGAGGTGAAGCACCCGTTCCGGAACCT  821
Lr67(res)                 CAACGAGATCGTGGAGGCGAGCCGCATCGCGCAGGAGGTGAAGCACCCGTTCCGGAACCT  821
ScLr_SUG6 (Lo7)           CGCGGACCTGGTGGTGGCGAGCGAGGAGTCCAAGCTGGTGCAGCACCCGTGGCGCAACAT  827
ScLr_SUG6_118_DANKO_APR   CGCGGACCTGGTGGTGGCGAGCGAGGAGTCCAAGCTGGTGCAGCACCCGTGGCGCAACAT  720
ScLr_SUG6_120_DANKO_APR   CGCGGACCTGGTGGTGGCGAGCGAGGAGTCCAAGCTGGTGCAGCACCCGTGGCGCAACAT  709
ScLr_SUG6_71_PHR_APR      CGCGGACCTGGTGGTGGCGAGCGAGGAGTCCAAGCTGGTGCAGCACCCGTGGCGCAACAT  722
ScLr_SUG6_149_PHR_APR     CGCGGACCTGGTGGTGGCGAGCGAGGAGTCCAAGCTGGTGCAGCACCCGTGGCGCAACAT  712
ScLr_SUG6_59_DANKO_non-APR CGCGGACCTGGTGGTGGCGAGCGAGGAGTCCAAGCTGGTGCAGCACCCGTGGCGCAACAT  712
ScLr_SUG6_61_DANKO_non-APR CGCGGACCTGGTGGTGGCGAGCGAGGAGTCCAAGCTGGTGCAGCACCCGTGGCGCAACAT  712
ScLr_SUG6_88_PHR_non-APR  CGCGGACCTGGTGGTGGCGAGCGAGGAGTCCAAGCTGGTGCAGCACCCGTGGCGCAACAT  712
ScLr_SUG6_105_PHR_non-APR CGCGGACCTGGTGGTGGCGAGCGAGGAGTCCAAGCTGGTGCAGCACCCGTGGCGCAACAT  721
```

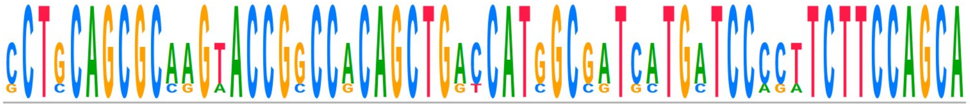

```
Consensus                 CCTGCAGCGCAAGTACCGGCCACAGCTGACCATGGCGATCATGATCCCCTTCTTCCAGCA
                                 850       860       870       880       890       900
Lr67(sus)                 GCTCCAGCGCCGGAACCGCCCGCAGCTGGTCATCGCCGTGCTGCTCCAGATCTTCCAGCA  881
Lr67(res)                 GCTCCAGCGCCGGAACCGCCCGCAGCTGGTCATCGCCGTGCTGCTCCAGATCTTCCAGCA  881
ScLr_SUG6 (Lo7)           CCTGCAGCGCAAGTACCGGCCACAGCTGACCATGGCGATCATGATCCCCTTCTTCCAGCA  887
ScLr_SUG6_118_DANKO_APR   CCTGCAGCGCAAGTACCGGCCACAGCTGACCATGGCGATCATGATCCCCTTCTTCCAGCA  780
ScLr_SUG6_120_DANKO_APR   CCTGCAGCGCAAGTACCGGCCACAGCTGACCATGGCGATCATGATCCCCTTCTTCCAGCA  769
ScLr_SUG6_71_PHR_APR      CCTGCAGCGCAAGTACCGGCCACAGCTGACCATGGCGATCATGATCCCCTTCTTCCAGCA  782
ScLr_SUG6_149_PHR_APR     CCTGCAGCGCAAGTACCGGCCACAGCTGACCATGGCGATCATGATCCCCTTCTTCCAGCA  772
ScLr_SUG6_59_DANKO_non-APR CCTGCAGCGCAAGTACCGGCCACAGCTGACCATGGCGATCATGATCCCCTTCTTCCAGCA  772
ScLr_SUG6_61_DANKO_non-APR CCTGCAGCGCAAGTACCGGCCACAGCTGACCATGGCGATCATGATCCCCTTCTTCCAGCA  772
ScLr_SUG6_88_PHR_non-APR  CCTGCAGCGCAAGTACCGGCCACAGCTGACCATGGCGATCATGATCCCCTTCTTCCAGCA  772
ScLr_SUG6_105_PHR_non-APR CCTGCAGCGCAAGTACCGGCCACAGCTGACCATGGCGATCATGATCCCCTTCTTCCAGCA  781
```

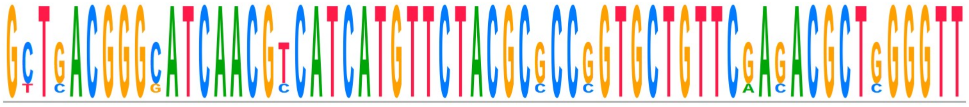

**Consensus**	GCTGACGGGCATCAACGTCATCATGTTCTACGCGCCGGTGCTGTTCGAGACGCTGGGGTT	
	910 920 930 940 950 960	
Lr67(sus)	GTTCACGGGGATCAACGCCATCATGTTCTACGCCCCCGTGCTGTTCAACACGCTCGGGTT	941
Lr67(res)	GTTCACGGGGATCAACGCCATCATGTTCTACGCCCCCGTGCTGTTCAACACGCTCGGGTT	941
ScLr_SUG6 (Lo7)	GCTGACGGGCATCAACGTCATCATGTTCTACGCGCCGGTGCTGTTCGAGACGCTGGGGTT	947
ScLr_SUG6_118_DANKO_APR	GCTGACGGGCATCAACGTCATCATGTTCTACGCGCCGGTGCTGTTCGAGACGCTGGGGTT	840
ScLr_SUG6_120_DANKO_APR	GCTGACGGGCATCAACGTCATCATGTTCTACGCGCCGGTGCTGTTCGAGACGCTGGGGTT	829
ScLr_SUG6_71_PHR_APR	GCTGACGGGCATCAACGTCATCATGTTCTACGCGCCGGTGCTGTTCGAGACGCTGGGGTT	842
ScLr_SUG6_149_PHR_APR	GCTGACGGGCATCAACGTCATCATGTTCTACGCGCCGGTGCTGTTCGAGACGCTGGGGTT	832
ScLr_SUG6_59_DANKO_non-APR	GCTGACGGGCATCAACGTCATCATGTTCTACGCGCCGGTGCTGTTCGAGACGCTGGGGTT	832
ScLr_SUG6_61_DANKO_non-APR	GCTGACGGGCATCAACGTCATCATGTTCTACGCGCCGGTGCTGTTCGAGACGCTGGGGTT	832
ScLr_SUG6_88_PHR_non-APR	GCTGACGGGCATCAACGTCATCATGTTCTACGCGCCGGTGCTGTTCGAGACGCTGGGGTT	832
ScLr_SUG6_105_PHR_non-APR	GCTGACGGGCATCAACGTCATCATGTTCTACGCGCCGGTGCTGTTCGAGACGCTGGGGTT	841

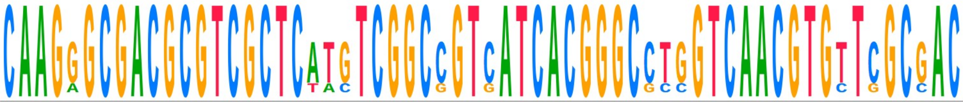

**Consensus**	CAAGGGCGACGCGTCGCTCATGTCGGCCGTCATCACGGGCCTGGTCAACGTGTTCGCGAC	
	970 980 990 1000 1010 1020	
Lr67(sus)	CAAGAGCGACGCGTCGCTCTACTCGGCGGTGATCACGGGCGCCGTCAACGTGCTGGCCAC	1001
Lr67(res)	CAAGAGCGACGCGTCGCTCTACTCGGCGGTGATCACGGGCGCCGTCAACGTGCTGGCCAC	1001
ScLr_SUG6 (Lo7)	CAAGGGCGACGCGTCGCTCATGTCGGCCGTCATCACGGGCCTGGTCAACGTGTTCGCGAC	1007
ScLr_SUG6_118_DANKO_APR	CAAGGGCGACGCGTCGCTCATGTCGGCCGTCATCACGGGCCTGGTCAACGTGTTCGCGAC	900
ScLr_SUG6_120_DANKO_APR	CAAGGGCGACGCGTCGCTCATGTCGGCCGTCATCACGGGCCTGGTCAACGTGTTCGCGAC	889
ScLr_SUG6_71_PHR_APR	CAAGGGCGACGCGTCGCTCATGTCGGCCGTCATCACGGGCCTGGTCAACGTGTTCGCGAC	902
ScLr_SUG6_149_PHR_APR	CAAGGGCGACGCGTCGCTCATGTCGGCCGTCATCACGGGCCTGGTCAACGTGTTCGCGAC	892
ScLr_SUG6_59_DANKO_non-APR	CAAGGGCGACGCGTCGCTCATGTCGGCCGTCATCACGGGCCTGGTCAACGTGTTCGCGAC	892
ScLr_SUG6_61_DANKO_non-APR	CAAGGGCGACGCGTCGCTCATGTCGGCCGTCATCACGGGCCTGGTCAACGTGTTCGCGAC	892
ScLr_SUG6_88_PHR_non-APR	CAAGGGCGACGCGTCGCTCATGTCGGCCGTCATCACGGGCCTGGTCAACGTGTTCGCGAC	892
ScLr_SUG6_105_PHR_non-APR	CAAGGGCGACGCGTCGCTCATGTCGGCCGTCATCACGGGCCTGGTCAACGTGTTCGCGAC	901

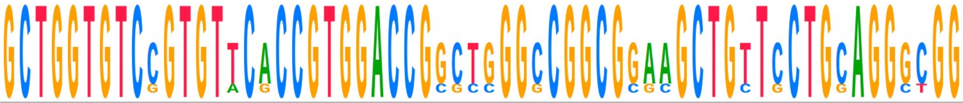

**Consensus**	GCTGGTGTCCGTGTTCACCGTGGACCGGCTGGGCCGGCGGAAGCTGTTCCTGCAGGGCGG	
	1030 1040 1050 1060 1070 1080	
Lr67(sus)	GCTGGTGTCGGTGTACGCCGTGGACCGCGCCGGGCGGCGCGCTGCTGCTGGAGGCTGG	1061
Lr67(res)	GCTGGTGTCGGTGTACGCCGTGGACCGCGCCGGGCGGCGCGCTGCTGCTGGAGGCTGG	1061
ScLr_SUG6 (Lo7)	GCTGGTGTCCGTGTTCACCGTGGACCGGCTGGGCCGGCGGAAGCTGTTCCTGCAGGGCGG	1067
ScLr_SUG6_118_DANKO_APR	GCTGGTGTCCGTGTTCACCGTGGACCGGCTGGGCCGGCGGAAGCTGTTCCTGCAGGGCGG	960
ScLr_SUG6_120_DANKO_APR	GCTGGTGTCCGTGTTCACCGTGGACCGGCTGGGCCGGCGGAAGCTGTTCCTGCAGGGCGG	949
ScLr_SUG6_71_PHR_APR	GCTGGTGTCCGTGTTCACCGTGGACCGGCTGGGCCGGCGGAAGCTGTTCCTGCAGGGCGG	962
ScLr_SUG6_149_PHR_APR	GCTGGTGTCCGTGTTCACCGTGGACCGGCTGGGCCGGCGGAAGCTGTTCCTGCAGGGCGG	952
ScLr_SUG6_59_DANKO_non-APR	GCTGGTGTCCGTGTTCACCGTGGACCGGCTGGGCCGGCGGAAGCTGTTCCTGCAGGGCGG	952
ScLr_SUG6_61_DANKO_non-APR	GCTGGTGTCCGTGTTCACCGTGGACCGGCTGGGCCGGCGGAAGCTGTTCCTGCAGGGCGG	952
ScLr_SUG6_88_PHR_non-APR	GCTGGTGTCCGTGTTCACCGTGGACCGGCTGGGCCGGCGGAAGCTGTTCCTGCAGGGCGG	952
ScLr_SUG6_105_PHR_non-APR	GCTGGTGTCCGTGTTCACCGTGGACCGGCTGGGCCGGCGGAAGCTGTTCCTGCAGGGCGG	961

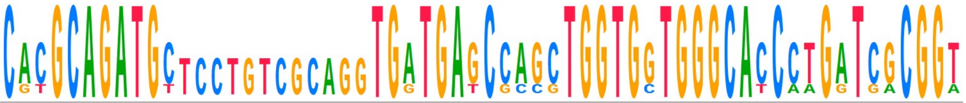

**Consensus**	CACGCAGATGC------------TGATGAGCCAGCTGGTGGTGGGCACCCTGATCGCGGT	
	1090 1100 1110 1120 1130 1140	
Lr67(sus)	CGTGCAGATGTTCCTGTCGCAGGTGGTGATCGCCGTGGTGCTGGGCATCAAGGTGACGGA	1121
Lr67(res)	CGTGCAGATGTTCCTGTCGCAGGTGGTGATCGCCGTGGTGCTGGGCATCAAGGTGACGGA	1121
ScLr_SUG6 (Lo7)	CACGCAGATGC------------TGATGAGCCAGCTGGTGGTGGGCACCCTGATCGCGGT	1115
ScLr_SUG6_118_DANKO_APR	CACGCAGATGC------------TGATGAGCCAGCTGGTGGTGGGCACCCTGATCGCGGT	1008
ScLr_SUG6_120_DANKO_APR	CACGCAGATGC------------TGATGAGCCAGCTGGTGGTGGGCACCCTGATCGCGGT	997
ScLr_SUG6_71_PHR_APR	CACGCAGATGC------------TGATGAGCCAGCTGGTGGTGGGCACCCTGATCGCGGT	1010
ScLr_SUG6_149_PHR_APR	CACGCAGATGC------------TGATGAGCCAGCTGGTGGTGGGCACCCTGATCGCGGT	1000
ScLr_SUG6_59_DANKO_non-APR	CACGCAGATGC------------TGATGAGCCAGCTGGTGGTGGGCACCCTGATCGCGGT	1000
ScLr_SUG6_61_DANKO_non-APR	CACGCAGATGC------------TGATGAGCCAGCTGGTGGTGGGCACCCTGATCGCGGT	1000
ScLr_SUG6_88_PHR_non-APR	CACGCAGATGC------------TGATGAGCCAGCTGGTGGTGGGCACCCTGATCGCGGT	1000
ScLr_SUG6_105_PHR_non-APR	CACGCAGATGC------------TGATGAGCCAGCTGGTGGTGGGCACCCTGATCGCGGT	1009

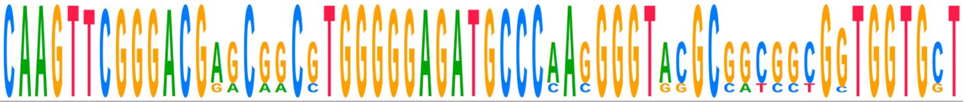

**Consensus**	CAAGTTCGGGACGAGCGGCGTGGGGGAGATGCCCAAGGGGTACGCGGCGGCGGTGGTGCT	
	1150 1160 1170 1180 1190 1200	
Lr67(sus)	CAAGT------CGGACAACCTGGG---------CCACGGGTGGCCATCCTGGTGGTGGT	1166
Lr67(res)	CAAGT------CGGACAACCTGGG---------CCACGGGTGGCCATCCTGCTGGTGGT	1166
ScLr_SUG6 (Lo7)	CAAGTTCGGGACGAGCGGCGTGGGGGAGATGCCCAAGGGGTACGCGGCGGCGGTGGTGCT	1175
ScLr_SUG6_118_DANKO_APR	CAAGTTCGGGACGAGCGGCGTGGGGGAGATGCCCAAGGGGTACGCGGCGGCGGTGGTGCT	1068
ScLr_SUG6_120_DANKO_APR	CAAGTTCGGGACGAGCGGCGTGGGGGAGATGCCCAAGGGGTACGCGGCGGCGGTGGTGCT	1057
ScLr_SUG6_71_PHR_APR	CAAGTTCGGGACGAGCGGCGTGGGGGAGATGCCCAAGGGGTACGCGGCGGCGGTGGTGCT	1070
ScLr_SUG6_149_PHR_APR	CAAGTTCGGGACGAGCGGCGTGGGGGAGATGCCCAAGGGGTACGCGGCGGCGGTGGTGCT	1060
ScLr_SUG6_59_DANKO_non-APR	CAAGTTCGGGACGAGCGGCGTGGGGGAGATGCCCAAGGGGTACGCGGCGGCGGTGGTGCT	1060
ScLr_SUG6_61_DANKO_non-APR	CAAGTTCGGGACGAGCGGCGTGGGGGAGATGCCCAAGGGGTACGCGGCGGCGGTGGTGCT	1060
ScLr_SUG6_88_PHR_non-APR	CAAGTTCGGGACGAGCGGCGTGGGGGAGATGCCCAAGGGGTACGCGGCGGCGGTGGTGCT	1060
ScLr_SUG6_105_PHR_non-APR	CAAGTTCGGGACGAGCGGCGTGGGGGAGATGCCCAAGGGGTACGCGGCGGCGGTGGTGCT	1069

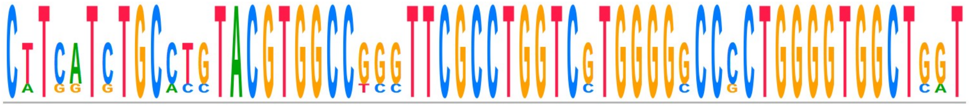

**Consensus**	CTTCATCTGCCTGTACGTGGCCGGGTTCGCCTGGTCGTGGGGGCCCCTGGGGTGGCTGGT	
	1210 1220 1230 1240 1250 1260	
Lr67(sus)	CATGGTGTGCACCTACGTGGCCTCCTTCGCCTGGTCCTGGGGCCCGCTGGGGTGGCTCAT	1226
Lr67(res)	CATGGTGTGCACCTACGTGGCCTCCTTCGCCTGGTCCTGGGGCCCGCTGGGGTGGCTCAT	1226
ScLr_SUG6 (Lo7)	CTTCATCTGCCTGTACGTGGCCGGGTTCGCCTGGTCGTGGGGGCCCCTGGGGTGGCTGGT	1235
ScLr_SUG6_118_DANKO_APR	CTTCATCTGCCTGTACGTGGCCGGGTTCGCCTGGTCGTGGGGGCCCCTGGGGTGGCTGGT	1128
ScLr_SUG6_120_DANKO_APR	CTTCATCTGCCTGTACGTGGCCGGGTTCGCCTGGTCGTGGGGGCCCCTGGGGTGGCTGGT	1117
ScLr_SUG6_71_PHR_APR	CTTCATCTGCCTGTACGTGGCCGGGTTCGCCTGGTCGTGGGGGCCCCTGGGGTGGCTGGT	1130
ScLr_SUG6_149_PHR_APR	CTTCATCTGCCTGTACGTGGCCGGGTTCGCCTGGTCGTGGGGGCCCCTGGGGTGGCTGGT	1120
ScLr_SUG6_59_DANKO_non-APR	CTTCATCTGCCTGTACGTGGCCGGGTTCGCCTGGTCGTGGGGGCCCCTGGGGTGGCTGGT	1120
ScLr_SUG6_61_DANKO_non-APR	CTTCATCTGCCTGTACGTGGCCGGGTTCGCCTGGTCGTGGGGGCCCCTGGGGTGGCTGGT	1120
ScLr_SUG6_88_PHR_non-APR	CTTCATCTGCCTGTACGTGGCCGGGTTCGCCTGGTCGTGGGGGCCCCTGGGGTGGCTGGT	1120
ScLr_SUG6_105_PHR_non-APR	CTTCATCTGCCTGTACGTGGCCGGGTTCGCCTGGTCGTGGGGGCCCCTGGGGTGGCTGGT	1129

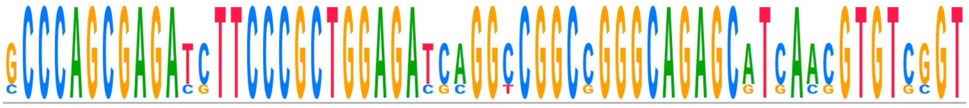

**Consensus**	GCCCAGCGAGATCTTCCCGCTGGAGATCAGGCCGGCCGGGCAGAGCATCAACGTGTCGGT	
	1270 1280 1290 1300 1310 1320	
Lr67(sus)	CCCCAGCGAGACGTTCCCGCTGGAGACGCGGTCGGCGGGGCAGAGCGTGACGGTGTGCGT	1286
Lr67(res)	CCCCAGCGAGACGTTCCCGCTGGAGACGCGGTCGGCGGGGCAGAGCGTGACGGTGTGCGT	1286
ScLr_SUG6 (Lo7)	GCCCAGCGAGATCTTCCCGCTGGAGATCAGGCCGGCCGGGCAGAGCATCAACGTGTCGGT	1295
ScLr_SUG6_118_DANKO_APR	GCCCAGCGAGATCTTCCCGCTGGAGATCAGGCCGGCCGGGCAGAGCATCAACGTGTCGGT	1188
ScLr_SUG6_120_DANKO_APR	GCCCAGCGAGATCTTCCCGCTGGAGATCAGGCCGGCCGGGCAGAGCATCAACGTGTCGGT	1177
ScLr_SUG6_71_PHR_APR	GCCCAGCGAGATCTTCCCGCTGGAGATCAGGCCGGCCGGGCAGAGCATCAACGTGTCGGT	1190
ScLr_SUG6_149_PHR_APR	GCCCAGCGAGATCTTCCCGCTGGAGATCAGGCCGGCCGGGCAGAGCATCAACGTGTCGGT	1180
ScLr_SUG6_59_DANKO_non-APR	GCCCAGCGAGATCTTCCCGCTGGAGATCAGGCCGGCCGGGCAGAGCATCAACGTGTCGGT	1180
ScLr_SUG6_61_DANKO_non-APR	GCCCAGCGAGATCTTCCCGCTGGAGATCAGGCCGGCCGGGCAGAGCATCAACGTGTCGGT	1180
ScLr_SUG6_88_PHR_non-APR	GCCCAGCGAGATCTTCCCGCTGGAGATCAGGCCGGCCGGGCAGAGCATCAACGTGTCGGT	1180
ScLr_SUG6_105_PHR_non-APR	GCCCAGCGAGATCTTCCCGCTGGAGATCAGGCCGGCCGGGCAGAGCATCAACGTGTCGGT	1189

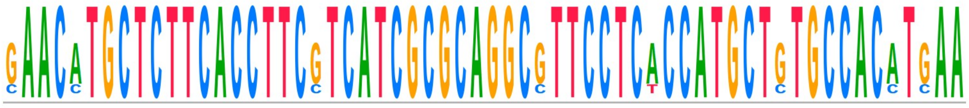

**Consensus**	GAACATGCTCTTCACCTTCGTCATCGCGCAGGCGTTCCTCACCATGCTGTGCCACATGAA	
	1330 1340 1350 1360 1370 1380	
Lr67(sus)	CAACCTGCTCTTCACCTTCCTCATCGCGCAGGCCTTCCTCTCCATGCTCTGCCACCTCAA	1346
Lr67(res)	CAACCTGCTCTTCACCTTCCTCATCGCGCAGGCCTTCCTCTCCATGCTCTGCCACCTCAA	1346
ScLr_SUG6 (Lo7)	GAACATGCTCTTCACCTTCGTCATCGCGCAGGCGTTCCTCACCATGCTGTGCCACATGAA	1355
ScLr_SUG6_118_DANKO_APR	GAACATGCTCTTCACCTTCGTCATCGCGCAGGCGTTCCTCACCATGCTGTGCCACATGAA	1248
ScLr_SUG6_120_DANKO_APR	GAACATGCTCTTCACCTTCGTCATCGCGCAGGCGTTCCTCACCATGCTGTGCCACATGAA	1237
ScLr_SUG6_71_PHR_APR	GAACATGCTCTTCACCTTCGTCATCGCGCAGGCGTTCCTCACCATGCTGTGCCACATGAA	1250
ScLr_SUG6_149_PHR_APR	GAACATGCTCTTCACCTTCGTCATCGCGCAGGCGTTCCTCACCATGCTGTGCCACATGAA	1240
ScLr_SUG6_59_DANKO_non-APR	GAACATGCTCTTCACCTTCGTCATCGCGCAGGCGTTCCTCACCATGCTGTGCCACATGAA	1240
ScLr_SUG6_61_DANKO_non-APR	GAACATGCTCTTCACCTTCGTCATCGCGCAGGCGTTCCTCACCATGCTGTGCCACATGAA	1240
ScLr_SUG6_88_PHR_non-APR	GAACATGCTCTTCACCTTCGTCATCGCGCAGGCGTTCCTCACCATGCTGTGCCACATGAA	1240
ScLr_SUG6_105_PHR_non-APR	GAACATGCTCTTCACCTTCGTCATCGCGCAGGCGTTCCTCACCATGCTGTGCCACATGAA	1249

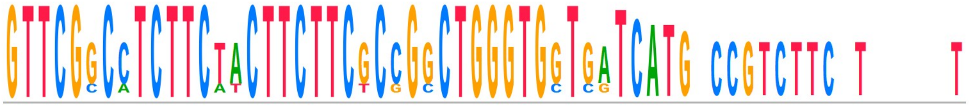

**Consensus**	GTTCGGCCTCTTCTACTTCTTCGCCGGCTGGGTGGTGATC--------------------	
	1390 1400 1410 1420 1430 1440	
Lr67(sus)	GTTCGCCATCTTCATCTTCTTCTCGGCCTGGGTGCTCGTCATGTCCGTCTTCGTGCTCTT	1406
Lr67(res)	GTTCGCCATCTTCATCTTCTTCTCGGCCTGGGTGCTCGTCATGTCCGTCTTCGTGCTCTT	1406
ScLr_SUG6 (Lo7)	GTTCGGCCTCTTCTACTTCTTCGCCGGCTGGGTGGTGATCATGACCGTCTTCATCGCGCT	1415
ScLr_SUG6_118_DANKO_APR	GTTCGGCCTCTTCTACTTCTTCGCCGGCTGGGTGGTG-----------------------	1285
ScLr_SUG6_120_DANKO_APR	GTTCGGCCTCTTCTACTTCTTCGCCGGCTGGGTGGTGA----------------------	1275
ScLr_SUG6_71_PHR_APR	GTTCGGCCTCTTCTACTTCTTCGCCGGCTGGGTGGTGAT---------------------	1289
ScLr_SUG6_149_PHR_APR	GTTCGGCCTCTTCTACTTCTTCGCCGGCTGGGTGGTGATCAT------------------	1282
ScLr_SUG6_59_DANKO_non-APR	GTTCGGCCTCTTCTACTTCTTCGCCGGCTGGGTGGTGAT---------------------	1279
ScLr_SUG6_61_DANKO_non-APR	GTTCGGCCTCTTCTACTTCTTCGCCGGCTGGGTGGTGATCATG-----------------	1283
ScLr_SUG6_88_PHR_non-APR	GTTCGGCCTCTTCTACTTCTTCGCCGGCTGGGTGGTGATC--------------------	1280
ScLr_SUG6_105_PHR_non-APR	GTTCGGCCTCTTCTACTTCTTCGCCGGCTGGGTGGTGAT---------------------	1288

```
                          CTTCCT CC GAGACCAAGAACGTGCCCATCGA GAGATG     C     G  G  GCA
Consensus                 ------------------------------------------------------------
                                   1450      1460      1470      1480      1490      1500
Lr67(sus)                 CTTCCTCCCGGAGACCAAGAACGTGCCCATCGAGGAGATGACCGACAAGGTGTGGAAGCA 1466
Lr67(res)                 CTTCCTCCCGGAGACCAAGAACGTGCCCATCGAGGAGATGACCGACAAGGTGTGGAAGCA 1466
ScLr_SUG6 (Lo7)           CTTCCTGCCCGAGACCAAGAACGTGCCCATCGAAGAGATGGTGCTCGTCTGGAAGGGGCA 1475
ScLr_SUG6_118_DANKO_APR   ------------------------------------------------------------ 1285
ScLr_SUG6_120_DANKO_APR   ------------------------------------------------------------ 1275
ScLr_SUG6_71_PHR_APR      ------------------------------------------------------------ 1289
ScLr_SUG6_149_PHR_APR     ------------------------------------------------------------ 1282
ScLr_SUG6_59_DANKO_non-APR ------------------------------------------------------------ 1279
ScLr_SUG6_61_DANKO_non-APR ------------------------------------------------------------ 1283
ScLr_SUG6_88_PHR_non-APR  ------------------------------------------------------------ 1280
ScLr_SUG6_105_PHR_non-APR ------------------------------------------------------------ 1288
```

```
                             T  T  G A G   TC   GACG GACG CCAC  C  C  CA C  C  C
Consensus                 ------------------------------------------------------------
                                   1510      1520      1530      1540      1550      1560
Lr67(sus)                 GCACTGGTTCTGGAAGAGATTCATGGACGACGACGACCACCACCACAACATCGCCAACGG 1526
Lr67(res)                 GCACTGGTTCTGGAAGAGATTCATGGACGACGACGACCACCACCACAACATCGCCAACGG 1526
ScLr_SUG6 (Lo7)           CTGGTTCTGGCGCAGGTACATCGGAGACGCTGACGTCCACGTCGGCGCCAACAACGGCAA 1535
ScLr_SUG6_118_DANKO_APR   ------------------------------------------------------------ 1285
ScLr_SUG6_120_DANKO_APR   ------------------------------------------------------------ 1275
ScLr_SUG6_71_PHR_APR      ------------------------------------------------------------ 1289
ScLr_SUG6_149_PHR_APR     ------------------------------------------------------------ 1282
ScLr_SUG6_59_DANKO_non-APR ------------------------------------------------------------ 1279
ScLr_SUG6_61_DANKO_non-APR ------------------------------------------------------------ 1283
ScLr_SUG6_88_PHR_non-APR  ------------------------------------------------------------ 1280
ScLr_SUG6_105_PHR_non-APR ------------------------------------------------------------ 1288
```

```
                                 CGCCA  G  T
Consensus                 -------------------
                                   1570
Lr67(sus)                 CAAGAACGCCACCGTCTGA 1545
Lr67(res)                 CAAGAACGCCACCGTCTGA 1545
ScLr_SUG6 (Lo7)           GGGCGCCGCCATTGCATAG 1554
ScLr_SUG6_118_DANKO_APR   ------------------- 1285
ScLr_SUG6_120_DANKO_APR   ------------------- 1275
ScLr_SUG6_71_PHR_APR      ------------------- 1289
ScLr_SUG6_149_PHR_APR     ------------------- 1282
ScLr_SUG6_59_DANKO_non-APR ------------------- 1279
ScLr_SUG6_61_DANKO_non-APR ------------------- 1283
ScLr_SUG6_88_PHR_non-APR  ------------------- 1280
ScLr_SUG6_105_PHR_non-APR ------------------- 1288
```

Sequence Logo: 50% GC base composition

Consensus Threshold: >50%

Colors: 4-color highlighting

Created: 26 lis 2024

Last Modified: 26 lis 2024
