# Supplementary material for: Searching for genes determining the APR phenotype in rye
Source: BMC Plant Biol. 2025 Jul 19;25:935. doi: 10.1186/s12870-025-06920-0 (PMC12275401; doi:10.1186/s12870-025-06920-0)
Supplement: Supplementary file 10 — Supplementary Material 10. [file 12870_2025_6920_MOESM10_ESM.docx]

Supplementary Figure S8. The disease symptoms observed on APR and non-APR rye inbreed lines from Polish Breeding company – Danko Plant Breeding infected with a mixture of *Puccinia recondite* f. sp*. secalis* spores collected in another location, in Poznań Plant Breeding company - PHR. Observations were taken 10 days after infection.


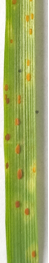

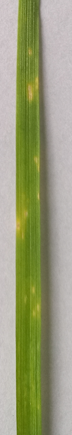

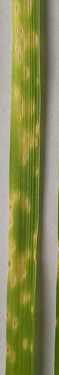

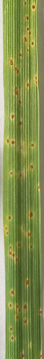


**APR rye lines**

**non APR rye lines**

61

59

120

118
